# Synthesis of Vicinal Carbocycles by Intramolecular Nickel-Catalyzed Conjunctive Cross-Electrophile Coupling Reaction

**DOI:** 10.1021/acs.orglett.2c02481

**Published:** 2022-08-04

**Authors:** Kirsten A. Hewitt, Claire A. Herbert, Elizabeth R. Jarvo

**Affiliations:** Department of Chemistry, University of California, Irvine, California 92617, United States

## Abstract

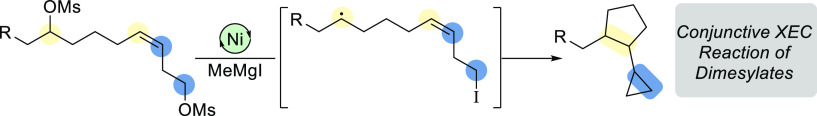

A nickel-catalyzed intramolecular conjunctive cross-electrophile coupling reaction has been established. This method enables the synthesis of 3,5-vicinal carbocyclic rings found in numerous biologically active compounds and natural products. We provide mechanistic experiments that indicate this reaction proceeds through alkyl iodides formed in situ, initiates at the secondary electrophilic center, and proceeds through radical intermediates.

Nickel-catalyzed conjunctive cross-electrophile coupling (XEC) reactions allow for the rapid and efficient synthesis of highly complex scaffolds, beginning with two electrophilic partners and an olefin (Scheme 1).^1,2^ These reactions pose major challenges in achieving high levels of stereo-, regio-, and chemoselectivity, particularly when performing three-component reactions (Scheme 1a). Building upon the mechanistic insights from XEC reactions,^3,4^ several strategies have been established to achieve selectivity, including mechanistic differentiation of the electrophiles or employing an excess of one reagent.^5−8^ In addition, the use of directing groups can also favor regio- and chemoselective reactions and allow for the use of unactivated conjunctive reagents.^9^ An additional strategy, tethering one electrophile and alkene together to afford a two-component reaction, also significantly addresses the selectivity challenges and at the same time constructs cyclic fragments (Scheme 1b).^10−12^ We envisioned a fully intramolecular nickel-catalyzed conjunctive XEC reaction to provide vicinal ring systems (Scheme 1c).^13,14^ This manifold engages two unactivated electrophiles and an internal olefin, and generates two carbocycles in a single step. Vicinal 3,5-carbocyclic motifs are present in a number of biologically active compounds and natural products, and cyclopropanes themselves are common in medicinal chemistry (Scheme 1d).^15−19^ We sought to prepare vicinal 3,5-carbocyclic motifs by a nickel-catalyzed conjunctive XEC reaction, where a single cascade reaction would construct both carbocyclic moieties.

**Scheme 1:**
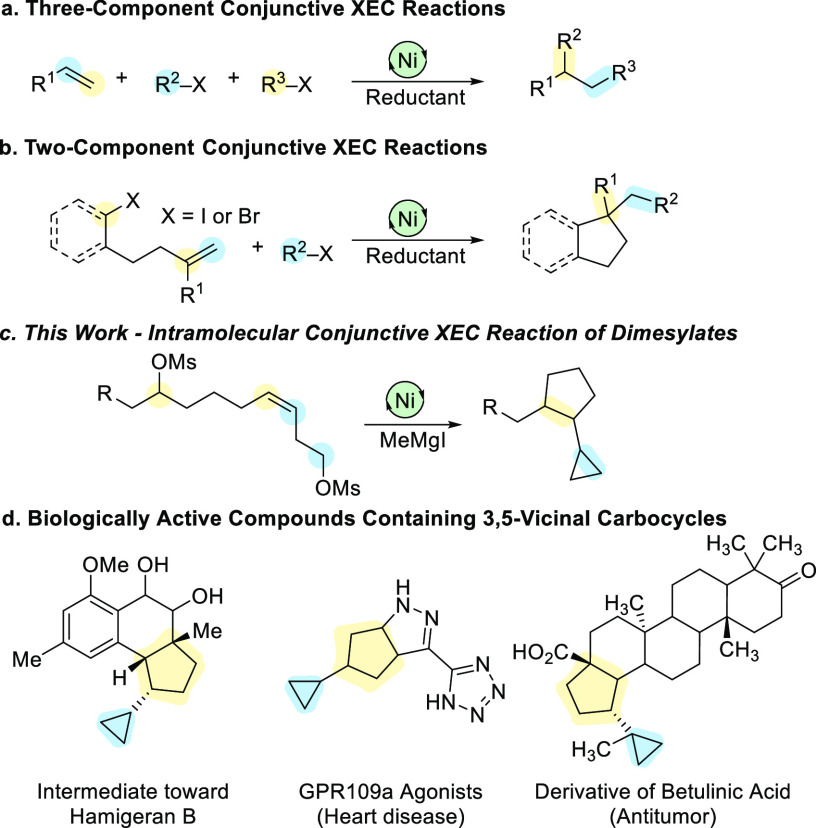
Previous Work in Conjunctive XEC Reactions and Medicinally Relevant Vicinal Carbocycles

In this manuscript, we report an intramolecular nickel-catalyzed conjunctive XEC reaction (Scheme 1c). The mechanistic framework of this reaction builds on our laboratory’s development of intramolecular XEC reactions of 1,3-dimesylates.^20^ In addition, it complements traditional radical reactions that initiate at alkyl halides and cascade forward to forge multiple ring systems.^21,22^ We provide preliminary mechanistic experiments to demonstrate that this reaction likely involves radical intermediates.

To begin, we designed a model substrate, dimesylate **1**, that contained two alkyl mesylates and an internal olefin. Based on previously developed cross-coupling and XEC reactions in our laboratory, we hypothesized that the secondary mesylate would be activated by the nickel catalyst and cascade forward through a 5-exo-trig cyclization.^19,23,24^ The proposed reaction would terminate by 3-exo-tet cyclization to afford the desired vicinal carbocycle **2**. First, we examined the previously developed conditions for the synthesis of cyclopropanes from 1,3-dimesylates, employing Ni(cod)_2_, racemic BINAP, and methylmagnesium iodide. We were delighted to observe the desired product in 85% yield (Table 1, entry 1). Next, to confirm that a *rac-*BINAP ligated nickel catalyst was responsible for the conjunctive XEC reaction, we performed the reaction without ligand; the yield decreased to 29% (entry 2). With these results, we then evaluated a series of ligands. While all ligands provided the desired product, *rac*-BINAP proved to be the optimal ligand (entries 1, 3–5). In the absence of the nickel catalyst and ligand, only diiodide (**3a**) was observed (entry 6). This result is consistent with formation of diiodides in situ as reactive intermediates.^19,25^ It also confirms that the nickel catalyst is necessary for the conjunctive XEC reaction to occur. Finally, we evaluated alternative reductants, including zinc, manganese, and TDAE, and found that the Grignard reagent provided the highest yield and minimal amounts of reduction product **4** (entries 7–10).

**Table 1:**
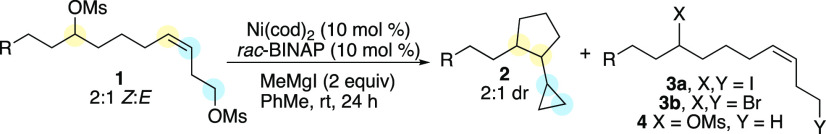
Cascade Reaction Optimization^a^

entry	deviation from standard conditions	recovered **1** (%)^b^	product **2** (%)^b^	dihalide **3** (%)^b^	reduction **4** (%)^b^
**1**	**None**	**0**	**85**	**0**	**0**
2	No Ligand	0	29	18	12
3	Dppm instead of *rac-*BINAP	0	49	0	0
4	BPhen instead of *rac*-BINAP	0	31	38	22
5	Bipy instead of *rac*-BINAP	0	46	0	0
6	No Nickel or Ligand	0	0	86	0
7^c^	Zn and NaI instead of MeMgI	15	<5	17	23
8^d^	Zn and MgBr_2_ instead of MeMgI	0	<5	54	19
9^e^	Mn and NaI/TMSCl instead of MeMgI	38	0	0	11
10^f^	TDAE and NaI instead of MeMgI	22	<5	0	12

a R = 4-MeO-C_6_H_4_.

b ^1^H NMR yield with PhTMS as standard.

c Zn (2 equiv), NaI (8 equiv).

d Zn, MgBr_2_ (2 equiv).

e Mn and NaI (2 equiv), TMSCl (1 equiv).

f TDAE, NaI (2 equiv).

With optimal reaction conditions in hand, we investigated the scope of the cascade reaction (Scheme 2). We were delighted to observe that both electron-donating and electron-withdrawing substituents were well tolerated under our standard reaction conditions (**2**, **5**–**9**). In addition, the cascade reaction with dimesylate **1** could be scaled 5-fold and retain similar yields. The cascade reaction also allowed for synthesis of a substituted tetrahydrofuran (**10**). Finally, a trisubstituted alkene was subjected to the reaction conditions and afforded adjacent quaternary and tertiary centers (**11**), albeit in moderate yield. For transformations where small amounts of olefinic byproducts were observed, dihydroxylation could be performed to ease purification of the desired product.^26^

**Scheme 2:**
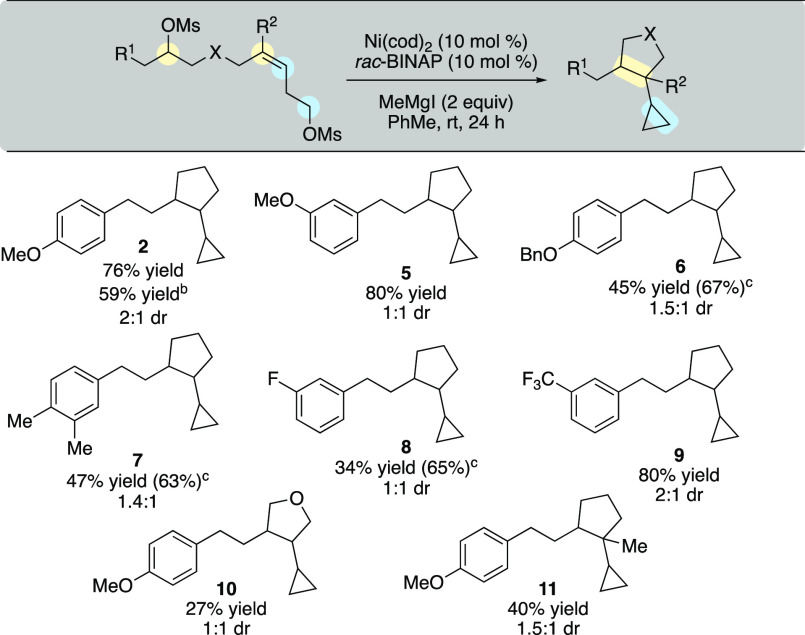
Conjunctive XEC Reaction Scope Reaction performed on 0.1 mmol scale unless otherwise noted. Reaction performed on 0.5 mmol scale. Yield in parentheses is ^1^H NMR yield compared to PhTMS as an internal standard.

Next, we turned our attention toward determining key features of the mechanism of this conjunctive XEC reaction. We hypothesized that the mechanism could proceed via two different pathways, involving either migratory insertion of an organonickel intermediate or a radical cyclization. Performing the cascade reaction with a single olefin isomer of dimesylate **1** provides a probe for radical versus organometallic cyclization (Scheme 3).^27^ Migratory insertion is a stereospecific process^28^ and would be expected to provide a single diastereomer of cyclopentane **2**. In contrast, radical cyclization would be stereoablative and lead to formation of both diastereomers. We separated the alkene diastereomers, employing silver impregnated silica gel, and subjected them separately to the cascade reaction. We observed that both (*E*)- and (*Z*)-**1** produced the same major diastereomer in 2:1 dr. This result is consistent with a radical exo-trig cyclization and not migratory insertion of an organonickel species.

**Scheme 3:**
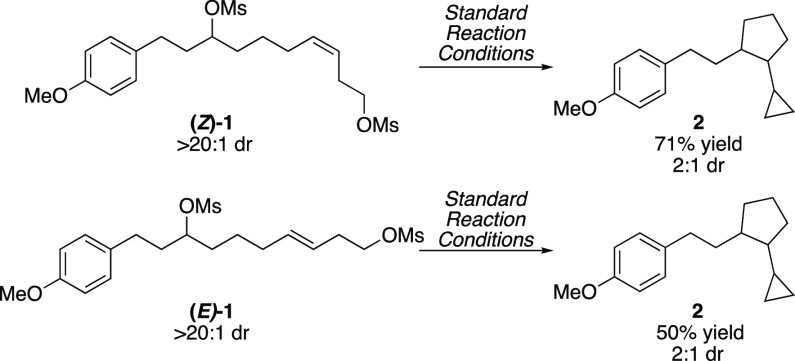
Control Reaction with Single Alkene Diastereomer

We aimed to further corroborate the proposed radical exo-trig cyclization by examining reactions of diiodide **3a**. First, to confirm that diiodide was a competent intermediate in the catalytic cycle, we subjected **3a** to the standard reaction conditions and observed product **2** in 73% yield (Table 2, entry 1). Therefore, we propose that dimesylate **1** is converted to diiodide **3a** in situ, and this intermediate engages the nickel catalyst by halogen atom transfer (XAT).^19,29^ If radical intermediates are operative, we should observe a decrease in yield with known radical inhibitors. Indeed, when 1 equiv of TEMPO was added to the standard reaction conditions, we observed a decrease in yield (Table 2, entry 2). In addition, we hypothesized that radical initiators, such as SmI_2_, should produce the desired carbocyclic system.^21^ Upon subjecting diiodide (**3a**) to a reaction with freshly prepared SmI_2_,^30^ we were excited to observe the desired cascade product in 56% yield and as a 1.5:1 mixture of diastereomers (Table 2, entry 3). These results are consistent with radical formation at one of the electrophilic centers and indicate that one or both cyclizations are radical mediated.

**Table 2:**
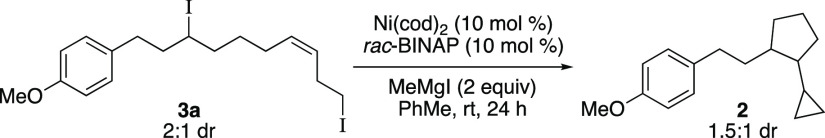
Control Reactions with Diiodide **3a**

entry	deviation from standard conditions	yield **2** (%)^a^
1	None	73
2	1 equiv of TEMPO added	48
3	SmI_2_ and THF instead of Ni, Ligand, and MeMgI	56^b^

a By ^1^H NMR compared to PhTMS standard.

b Isolated yield.

Finally, we aimed to understand which electrophile was activated first. Based on the selectivity of XAT reactions, we hypothesized that the reaction initiated at the secondary center.^19^ However, we had observed the formation of reduction product **4** in the optimization studies (vide supra) and considered that the primary iodide may engage the nickel complex first. We designed the following competition experiment to investigate the order of events. We synthesized two mesylates: one with a 2° mesylate (**12**) and one with a 1° mesylate (**15**). In a competition experiment between dimesylate **1** and 2° mesylate **12**, we observed a 1.5:1 ratio of products (Scheme 4a). However, in a similar competition experiment, now employing 1° mesylate **15**, the product ratio observed was 2.6:1 (Scheme 4b). The 2° mesylate **12** reacted at a competitive rate compared to dimesylate **1** and demonstrated that the 2° mesylate reacted faster than the 1° mesylate. These results indicate that the productive pathway for the conjunctive XEC reaction initiates at the secondary center. This selectivity is consistent with previous observations that secondary alkyl halides react at a faster rate than primary halides with nickel catalysts.^19,28^

**Scheme 4:**
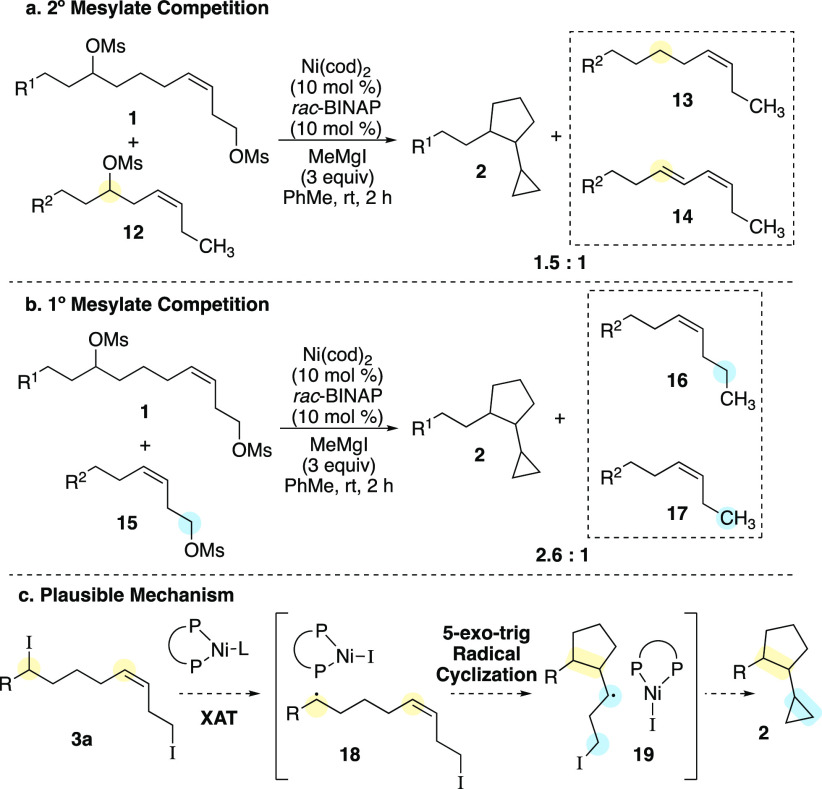
Competition Experiments and Proposed Reaction Mechanism (R^1^ = 4-MeO-C_6_H_4_, R^2^ = 4-BnO-C_6_H_4_)

Based on the mechanistic experiments, we proposed the following plausible reaction mechanism (Scheme 4c). Beginning from diiodide **3a**, generated in situ, halogen atom transfer occurs at the secondary alkyl iodide to generate alkyl radical **18**. This secondary alkyl radical cyclizes to afford the cyclopentane ring **19**.^31^ This radical cyclization is consistent with formation of a mixture of diastereomers from either *cis*- or *trans*-alkenes (vide supra). Following this step, radical or nickel-mediated 3-exo-tet cyclization would afford cyclopropane **2**. Cyclization could occur by direct S_H_2-type cyclization of **19** to generate iodine radical, or by formation of a nucleophilic alkylnickel(II) intermediate that undergoes S_N_2-type cyclization. Both pathways eventually lead to a nickel(II) complex, which is reduced by the Grignard reagent to regenerate the nickel(0) catalyst.^32^

In conclusion, we have developed a nickel-catalyzed cascade reaction for synthesis of 3,5-vicinal carbocyclic motifs. We have demonstrated the scope of the reaction to include electron-donating and -withdrawing groups. In addition, we have provided preliminary mechanistic experiments to demonstrate that alkyl iodides are likely generated in situ and that the cascade reaction likely proceeds through radical intermediates. Future work includes delineating the remaining steps of the reaction mechanism, including the nature of the 3-exo-tet cyclization, and development of related cascade reactions.

## Supporting Information Available

Experimental details and NMR spectra (PDF)
