# Supplementary material for: Synthesis of Vicinal Carbocycles by Intramolecular Nickel-Catalyzed Conjunctive Cross-Electrophile Coupling Reaction
Source: Org Lett. 2022 Aug 4;24(32):6093–8. doi: 10.1021/acs.orglett.2c02481 (PMC9396665; doi:10.1021/acs.orglett.2c02481)
Supplement: Supplementary file 1 — ol2c02481_si_001.pdf [file ol2c02481_si_001.pdf]

Supporting Information

Synthesis of Vicinal Carbocycles by Intramolecular Nickel-Catalyzed Conjunctive Cross-Electrophile Coupling Reaction

Kirsten A. Hewitt, Claire A. Herbert, Elizabeth R. Jarvo*

Department of Chemistry, University of California, Irvine, California, 92697
*Corresponding Author: erjarvo@uci.edu

Table of Contents

I.	General Procedures	SI-2
II.	Experimental	SI-3
	a. Conjunctive XEC Reaction Procedures	SI-3
	Method A: Nickel-Catalyzed Conjunctive XEC Reactions	SI-3
	1) Preparation of Grignard Reagent	SI-3
	b. Characterization Data for Conjunctive XEC Reaction Products	SI-4
	c. Substrates that Provide Low Yields of Desired Product	SI-12
	d. Characterization Data for By-Products Formed from Optimization Studies	SI-12
	e. General Procedures for the Synthesis of Dimesylate Starting Materials	SI-13
	Method B: Pd-Catalyzed Heck Reaction	SI-13
	Method C: Preparation of Grignard Reagent	SI-13
	Method D: Nucleophilic Grignard Addition into Aldehyde	SI-13
	Method E: Ozonolysis of Terminal Alkenes	SI-14
	Method F: Preparation of Wittig Salt **SI-11**	SI-14
	Method G: Wittig Reaction	SI-14
	Method H: Mesylation Reaction	SI-14
	Method I: TBS Protection of Alcohols	SI-15
	Method J: Hydroboration Oxidation of Terminal Alkenes	SI-15
	Method K: Parikh-Doering Oxidation	SI-15
	Method L: Corey-Fuchs Reaction	SI-16
	Method M: Silyl Deprotection with Tetrabutylammonium Fluoride	SI-16
	f. Synthesis and Characterization Data for Dimesylate Starting Materials	SI-17
	g. Synthesis and Characterization Data for Mechanistic Experiments	SI-36
	1) Mechanistic Experiment with Single Alkene Diastereomer	SI-36
	2) Separation of Alkene Diastereomers on Silver Impregnated Silica Gel	SI-36
	3) SmI_2 Control Reaction	SI-36
	4) TEMPO Control Reaction	SI-37
	h. Competition Experiment Data	SI-38
	i. Synthesis and Characterization for Competition Experiment Starting Materials	SI-39
III.	References for Supporting Information	SI-42
IV.	Gas Chromatography Spectra for Competition Experiment	SI-44
V.	^{1}H, ^{13}C, ^{19}F, COSY, HMQC NMR Spectra	SI-48

I. **General Procedures**

All reactions were carried out under an atmosphere of N_2 when noted. All glassware was oven- or flame-dried prior to use. Tetrahydrofuran (THF), diethyl ether (Et_2O), dichloromethane (DCM), methanol (MeOH), triethylamine (Et_3N), dimethylformamide (DMF), and toluene (PhMe) were degassed with Ar and then passed through two 4 x 36 inch columns of anhydrous neutral A-2 alumina (8 x 14 mesh; LaRoche Chemicals; activated under a flow of argon at 350 ºC for 12 h) to remove H_2O.[1] All other solvents utilized were purchased "anhydrous" commercially, or purified as described. ^{1}H NMR spectra were recorded on Bruker DRX-400 (400 MHz ^{1}H, 100 MHz ^{13}C), GN-500 (500 MHz ^{1}H, 125.7 MHz ^{13}C), CRYO-500 (500 MHz ^{1}H, 125.7 MHz ^{13}C), or AVANCE-600 (600 MHz ^{1}H, 150 MHz ^{13}C) spectrometers. Proton chemical shifts are reported in ppm (δ) relative to internal tetramethylsilane (TMS, δ 0.00). Data are reported as follows: chemical shift (multiplicity [singlet (s), broad singlet (br s), doublet (d), doublet of doublets (dd), doublet of doublet of doublets (ddd), doublet of doublet of triplets (ddt), doublet of triplets (dt), triplet (t), triplet of doublets (td), triplet of triplets (tt), quartet (q), quartet of doublets (qd), quintet (quin), apparent singlet (as), apparent doublet (ad), apparent doublets of doublets (add), apparent doublet of triplets (adt), apparent doublet of quintets (adquin), apparent doublet of septets (adsept), apparent triplet (at), apparent triplet of doublets (atd), apparent triplet of triplets (att), apparent quartet (aq), apparent quartet of doublets (aqd), apparent quartet of triplets (aqt), apparent quintet of doublets (aquin d), apparent sextet (asext), apparent septet (asept), multiplet (m)], coupling constants [Hz], integration). Carbon chemical shifts are reported in ppm (δ) relative to TMS with the respective solvent resonance as the internal standard ($CDCl_3$, δ 77.16 ppm). Unless otherwise indicated, NMR data were collected at 25 ºC. Structural assignments were made with additional information from gCOSY, gHSQC, and gHMBC experiments. Infrared (IR) spectra were obtained on a Thermo Scientific Nicolet iS5 spectrometer with an iD5 ATR tip (neat) and are reported in terms of frequency of absorption (cm^{-1}). Analytical thin-layer chromatography (TLC) was performed using Silica Gel 60 F254 precoated plates (0.25 mm thickness). Visualization was accomplished by irradiation with a UV lamp. Flash chromatography was performed using SiliaFlash F60 (40-63 μm, 60 Å) from SiliCycle. Automated chromatography was carried out on a Teledyne Isco CombiFlash Rf Plus. Melting points (M.p.) were obtained using a MelTemp melting point apparatus and are uncorrected. High resolution mass spectrometry was performed by the University of California, Irvine Mass Spectrometry Center.

Bis(1,5-cyclooctadiene)nickel was purchased from Strem, stored in a glove box freezer (–20 °C) under an atmosphere of N_2 and used as received. All ligands were purchased from Strem or Sigma Aldrich and were stored under N_2 atmosphere and used as received. The methylmagnesium iodide was titrated with iodine prior to use.[2] All other chemicals were purchased commercially and used as received, unless otherwise noted.

II Experimental

a. Conjunctive XEC Reaction Procedures

Method A: Nickel-Catalyzed Conjunctive XEC Reaction

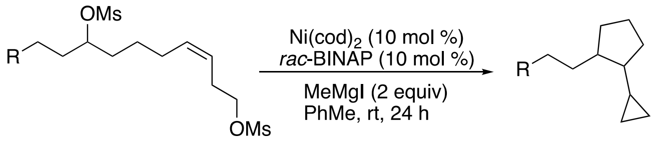

(SI-1)

In a glovebox, an oven-dried 7 mL dram vial equipped with a stir bar was charged with dimesylate (1.0 equiv), $Ni(cod)_2$ (10. mol %), *rac*-BINAP (10. mol %) and PhMe (0.20 M in substrate). A solution of MeMgI in Et_2O (2.0 equiv) was then added dropwise via syringe. After 24 h, the reaction vial was removed from the glovebox, quenched with MeOH, filtered through a plug of silica gel eluting with Et_2O, and concentrated in vacuo. Phenyltrimethylsilane (PhTMS; 8.6 μL, 50. μmol) was added and the yield was determined by ^1H NMR based on comparison to PhTMS as internal standard before purification by column chromatography.

To remove olefin by-products resulting from β-hydride elimination, a Sharpless asymmetric dihydroxylation was performed.[3] To a flame-dried 7-mL dram vial was added AD-mix-β (1.4 g/mmol). The flask was sealed with a septum cap and placed under an atmosphere of N_2. Then *t*-BuOH (0.50 mL) and H_2O (0.50 mL) were added via syringe. The vial was cooled to 0 ºC and then unpurified reaction mixture was added dropwise as a solution in *t*-BuOH (0.50 mL) and H_2O (0.5 mL) via syringe. The mixture was allowed to stir at 0 ºC for 24 h. To quench, saturated aq. $Na_2S_2O_3$ (1.0 mL) was added and the mixture was allowed to warm to rt and stir for 1 h. Then the mixture was transferred to a separatory funnel, and the organic layer was extracted with EtOAc (x 3). The combined organic layers were washed with brine, dried over Na_2SO_4, filtered and concentrated in vacuo.

1) Preparation of Grignard Reagent

$$\text{MeI} \xrightarrow[\text{Et}_2\text{O}]{\text{Mg}^0} \text{MeMgI} \qquad \text{(SI-2)}$$

Under a N_2 atmosphere, a three-necked flask equipped with a stir bar, reflux condenser, and Schlenk filtration apparatus was charged with magnesium turnings (1.1 g, 45 mmol, 1.5 equiv). The flask and magnesium turnings were then flame-dried under vacuum and the flask was back-filled with N_2. Anhydrous Et_2O (7.0 mL) and a crystal of iodine (ca. 2.0 mg) were added to the flask. Freshly distilled iodomethane (1.9 mL, 31 mmol, 1.0 equiv) was slowly added over 30 min to maintain a gentle reflux. The mixture was stirred for 2 h at room temperature then filtered through the fritted Schlenk filter into a Schlenk flask under N_2 atmosphere. The magnesium turnings were washed with Et_2O (2 x 1.0 mL) then the Schlenk flask was sealed, removed, and placed under an N_2 atmosphere. The resulting methylmagnesium iodide was typically between 2.4 and 3.0 M as titrated by Knochel's method[2] and could be stored, sealed under N_2 atmosphere or in a glovebox, for up to 4 weeks.

b. Characterization Data for Conjunctive XEC Reaction Products

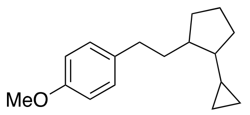

Vicinal carbocycle (2) was prepared according to Method A. The following amounts of reagents were used: dimesylate **1** (42 mg, 97 µmol, 1.0 equiv), $Ni(cod)_2$ (2.7 mg, 9.7 µmol, 10. mol %), *rac*-BINAP (6.0 mg, 9.7 µmol, 10. mol %), MeMgI (70. µL, 0.19 mmol, 2.0 equiv, 2.9 M in Et_2O), PhMe (0.48 mL), AD-mix-β (0.14 g, 1.4 g/mmol of dimesylate), *t*-BuOH (2.0 mL), and H_2O (1.5 mL). Purification by column chromatography (100% hexanes) afforded the title compound as a bright yellow oil (18 mg, 74 µmol, 76% yield, 2:1 dr). The dr was determined by the integration of resonances attributed to the aromatic carbons in ^{13}C NMR. **TLC R$_f$** = 0.7 (5% EtOAc/hexanes); **^{1}H NMR** (500 MHz, $CDCl_3$) δ 7.08–6.98 (m, 4H, both diastereomers), 6.75 (d, *J* = 8.1 Hz, 4H, both diastereomers), 3.70 (d, *J* = 1.6 Hz, 6H, both diastereomers), 2.60–2.38 (m, 4H, both diastereomers), 1.92–1.80 (m, 2H, both diastereomers), 1.79–1.56 (m, 4H, both diastereomers), 1.55–1.37 (m, 6H, both both diastereomers), 1.38–1.00 (m, 6H, both both diastereomers), 0.94–0.68 (m, 2H, both diastereomers), 0.53–0.43 (m, 2H, both diastereomers), 0.37 (ddq, *J* = 21.2, 8.5, 4.3 Hz, 2H, both diastereomers), 0.26 (dtt, *J* = 22.5, 8.9, 4.7 Hz, 2H, both diastereomers), 0.05 (tq, *J* = 9.4, 4.6 Hz, 2H, both diastereomers), -0.10 (tq, *J* = 9.5, 4.6 Hz, 2H, both diastereomers); **^{13}C NMR** (125 MHz, $CDCl_3$) δ 157.7 (2C, both diastereomers), 135.6 (major diastereomer), 135.4 (minor diastereomer), 129.32 (2C, major diastereomer), 129.26 (2C, minor diastereomer), 113.8 (4C, both diastereomers), 55.4 (2C, both diastereomers), 51.0 (minor diastereomer), 48.5 (major diastereomer), 46.2 (minor diastereomer), 43.1 (major diastereomer), 37.6 (minor diastereomer), 34.6 (major diastereomer), 34.2 (minor diastereomer), 33.5 (major diastereomer), 32.6 (minor diastereomer), 32.1 (minor diastereomer), 31.8 (major diastereomer), 30.7 (major diastereomer), 23.6 (minor diastereomer), 23.0 (major diastereomer), 15.4 (minor diastereomer), 11.8 (major diastereomer), 5.5 (major diastereomer), 4.4 (minor diastereomer), 3.0 (major diastereomer), 2.3 (major diastereomer); **HRMS** (TOF MS CI+) *m/z*: $[M]^+$ calcd for $C_{17}H_{24}O$, 244.1827; found 244.1831.

Scale Up Reaction: This scale up reaction was performed according to Method A. The following amounts of reagents were used: dimesylate **1** (217 mg, 0.50 mmol, 1.0 equiv), $Ni(cod)_2$ (14 mg, 0.050 mol, 10. mol %), *rac*-BINAP (31 mg, 0.050 mmol, 10. mol %), MeMgI (0.34 mL, 1.0 mmol, 2.0 equiv, 2.9 M in Et_2O), PhMe (2.5 mL), AD-mix-β (0.70 g, 1.4 g/mmol of dimesylate), *t*-BuOH (7.0 mL), and H_2O (5.0 mL). Purification by column chromatography (100% hexanes) afforded the title compound as a bright yellow oil as a mixture with 3% beta-hydride elimination (71 mg, 0.29 mmol, 59% yield). To remove residual beta-hydride elimination, we performed an additional dihydroxylation reaction with AD-mix-β (0.70 g, 1.4 g/mmol of dimesylate), *t*-BuOH (5.0 mL), and H_2O (5.0 mL) to afford the title compound as a bright yellow oil (51 mg, 0.21 mmol, 42% yield, 2:1 dr). The dr was determined by the integration of resonances attributed to the aromatic carbons in ^{13}C NMR.

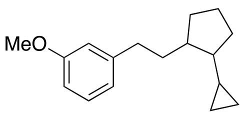

Vicinal carbocycle (5) was prepared according to Method A. The following amounts of reagents were used: dimesylate **SI-1** (50 mg, 0.11 mmol, 1.0 equiv), Ni(cod)$_2$ (3.0 mg, 10. µmol, 10. mol %), *rac*-BINAP (6.0 mg, 10. µmol, 10 mol %), MeMgI (70. µL, 0.22 mmol, 2.0 equiv, 2.9 M in Et_2O), PhMe (0.55 mL), AD-mix-β (154 mg, 1.4 g/mmol), *t*-BuOH (1.0 mL) and H_2O (1.0 mL). Purification by column chromatography (0–1% Et_2O/pentanes) afforded the title compound as a yellow oil (22 mg, 90. µmol, 80% yield, 1:1 dr). The dr was determined by the integration of resonances attributed to the aromatic carbons in ^{13}C NMR. **TLC R$_f$** = 0.3 (100% hexanes); **^{1}H NMR** (500 MHz, $CDCl_3$) δ 7.19 (t, *J* = 7.8 Hz, 2H, both diastereomers), 6.80 (d, *J* = 7.50 Hz, 1H, one diastereomer), 6.79 (d, *J* = 7.50 Hz, 1H, other diastereomer), 6.76 (aq, *J* = 1.39 Hz, 2H, both diastereomers), 6.73 (dd, *J* = 7.96, 1.34 Hz, 2H, both diastereomers), 3.80 (s, 6H, both diastereomers), 2.69–2.62 (m, 2H, both diastereomers), 2.60–2.54 (m, 2H, both diastereomers), 2.23 (aq, *J* = 5.39 Hz, 1H, one diastereomers), 2.00–1.48 (m, 14H, both diastereomers), 1.44–1.11 (m, 5H, both diastereomers), 0.59–0.53 (m, 2H, both diastereomers), 0.50–0.30 (m, 4H, both diastereomers), 0.15–0.10 (m, 2H, both diastereomers), 0.01–-0.05 (m, 2H, both diastereomers); **^{13}C NMR** (125 MHz, $CDCl_3$) δ 159.7 (2C, both diastereomers), 145.2 (one diastereomer), 145.0 (other diastereomer), 129.3 (2C, both diastereomers), 121.0 (one diastereomer), 120.9 (other diastereomer), 114.4 (one diastereomer), 114.3 (other diastereomer), 110.9 (one diastereomer), 110.8 (other diastereomer), 55.3 (2C, both diastereomers), 51.0 (one diastereomer), 48.5 (other diastereomer), 46.3 (one diastereomer), 43.2 (other diastereomer), 37.3 (one diastereomer), 35.6 (other diastereomer), 35.2 (one diastereomer), 33.1 (other diastereomer), 32.6 (one diastereomer), 32.1 (other diastereomer), 31.7 (one diastereomer), 30.7 (other diastereomer), 23.6 (one diastereomer), 23.0 (other diastereomer), 15.4 (one diastereomer), 11.8 (other diastereomer), 5.5 (one diastereomer), 4.4 (other diastereomer), 3.0 (one diastereomer), 2.2 (other diastereomer); **HRMS** (TOF MS CI+) *m/z*: $[M]^+$ calcd for $C_{17}H_{24}O$, 244.1827; found 244.1821.

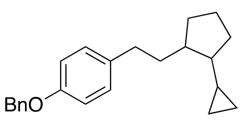

Vicinal carbocycle (6) was prepared according to Method A. The following amounts of reagents were used: dimesylate **SI-2** (32 mg, 60. μmol, 1.0 equiv), $Ni(cod)_2$ (1.7 mg, 6.0 μmol, 10 mol %), *rac*-BINAP (3.7 mg, 6.0 μmol, 10 mol %), MeMgI (40. μL, 0.12 mmol, 2.0 equiv, 3.0 M in Et_2O), PhMe (0.30 mL), AD-mix-β (84 mg, 1.4 g/mmol), *t*-BuOH (1.0 mL) and H_2O (1.0 mL). Purification by column chromatography (0–10% EtOAc/hexanes) afforded the title compound as a pale yellow oil (8.6 mg, 28 μmol, 45% yield, 1.5:1 dr). The dr was determined by the integration of resonances attributed to the aromatic carbons in ^{13}C NMR. **TLC** $\mathbf{R_f}$ = 0.8 (10% EtOAc/hexanes); ^{1}**H NMR** (600 MHz, $CDCl_3$) δ 7.43 (d, *J* = 7.3 Hz, 4H, both diastereomers), 7.38 (t, *J* = 7.4 Hz, 4H, both diastereomers), 7.34–7.29 (m, 2H, both diastereomers), 7.14–7.09 (m, 4H, both diastereomers), 6.92–6.87 (m, 4H, both diastereomers), 5.04 (s, 4H, both diastereomers), 2.62 (dddd, *J* = 13.6, 10.4, 5.3, 2.8 Hz, 2H, both diastereomers), 2.52 (dtd, *J* = 13.9, 10.0, 6.1, 2H, both diastereomers), 1.99–1.86 (m, 2H, both diastereomers), 1.86–1.61 (m, 6H, both diastereomers), 1.62–1.55 (m, 2H, both diastereomers), 1.54–1.46 (m, 2H, both diastereomers), 1.45–1.27 (m, 2H, both diastereomers), 1.21 (dq, *J* = 12.5, 8.5 Hz, 1H, minor diastereomer), 1.13 (dq, *J* = 11.3, 6.0, 1H, major diastereomer), 0.99–0.76 (m, 4H, both diastereomers), 0.55 (dddt, *J* = 13.1, 7.9, 5.3, 2.9 Hz, 2H, both diastereomers), 0.47 (dddd, *J* = 9.4, 8.1, 5.5, 4.2 Hz, 1H, major diastereomer), 0.44–0.39 (m, 1H, minor diastereomer), 0.40–0.34 (m, 1H, major diastereomer), 0.34–0.29 (m, 1H, minor diastereomer), 0.12 (tdd, *J* = 9.3, 5.2, 4.1 Hz, 2H, both diastereomers), -0.01—-0.06 (m, 2H, both diastereomers); ^{13}**C NMR** (151 MHz, $CDCl_3$) δ 156.96 (major diastereomer) 156.95 (minor diastereomer), 137.4 (2C, both diastereomers), 135.9 (minor diastereomer), 135.8 (major diastereomer), 129.4 (2C, major diastereomer), 129.3 (2C, minor diastereomer), 128.7 (4C, both diastereomers), 128.0 (2C, both diastereomers), 127.6 (4C, both diastereomers), 114.78 (2C, minor diastereomer), 114.76 (2C, major diastereomer), 70.20 (2C, both diastereomers), 51.0 (minor diastereomer), 48.5 (major diastereomer), 46.2 (minor diastereomer), 43.1 (major diastereomer), 37.6 (minor diastereomer), 34.6 (major diastereomer), 34.2 (minor diastereomer), 33.5 (major diastereomer), 32.6 (minor diastereomer) 32.1 (minor diastereomer), 31.8 (major diastereomer), 30.7 (major diastereomer), 23.6 (minor diastereomer), 23.0 (major diastereomer), 15.4 (minor diastereomer), 11.8 (major diastereomer), 5.5 (major diastereomer), 4.4 (minor diastereomer), 3.0 (major diastereomer), 2.3 (minor diastereomer); **HRMS** (TOF MS CI+) *m/z*: $[M]^+$ calcd for $C_{23}H_{28}O$, 320.2140, found 320.2154.

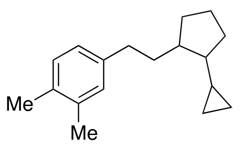

Vicinal carbocycle (7) was prepared according to Method A. The following amounts of reagents were used: dimesylate **SI-3** (89 mg, 0.20 mmol, 1.0 equiv), $Ni(cod)_2$ (5.5 mg, 20. µmol, 10 mol %), *rac*-BINAP (12 mg, 20. µmol, 10 mol %), MeMgI (0.14 mL, 0.40 mmol, 2.0 equiv, 2.9 M in Et_2O), PhMe (1.0 mL), AD-mix-β (430 mg, 2.1 g/mmol), *t*-BuOH (1.5 mL) and H_2O (1.5 mL). Before purification, a ^{1}H NMR yield of 63% was obtained based on comparison to PhTMS as an internal standard. Purification by column chromatography (100% hexanes) afforded the title compound as a colorless oil (21 mg, 90. µmol, 44% yield, 1.4:1 dr). The dr was determined by the integration of resonances attributed to the aromatic carbons in ^{13}C NMR. For clarity, the ^{13}C data is tabulated individually.

TLC $\mathbf{R_f}$ = 0.8 (100% hexanes); **HRMS** (TOF MS CI+) *m/z*: $[M]^+$ calcd for $C_{18}H_{26}$, 242.2034; found 242.2027.

^{1}H NMR (500 MHz, $CDCl_3$) δ 6.97 (d, *J* = 7.6 Hz, 2H, both diastereomers), 6.91 (s, 2H, both diastereomers), 6.87 (d, *J* = 6.96 Hz, 2H, both diastereomers), 2.57–2.51 (m, 2H, both diastereomers), 2.47–2.40 (m, 2H, both diastereomers) 2.17 (s, 6H, both diastereomers), 2.15 (s, 6H, both diastereomers), 1.91–1.58 (m, 9H, both diastereomers), 1.55-1.47 (m, 3H, both diastereomers), 1.44–1.39 (m, 2H, both diastereomers), 1.34–1.22 (m, 4H, both diastereomers), 1.19–1.12 (m, 1H, one diastereomer), 1.09–1.03 (m, 1H, one diastereomer), 0.52–0.45 (m, 2H, both diastereomers), 0.42–0.22 (m, 4H, both diastereomers), 0.08–0.03 (m, 2H, both diastereomers), -0.06–-0.13 (m, 2H, both diastereomers).

Major Diastereomer: ^{13}C NMR (125 MHz, $CDCl_3$) δ 140.9, 136.3, 133.6, 129.8, 129.6, 125.7, 48.4, 43.2, 35.0, 33.4, 31.7, 30.7 (2C), 22.9, 19.4, 11.8, 5.4, 3.0.

Minor Diastereomer: ^{13}C NMR (125 MHz, $CDCl_3$) δ 140.7, 136.3, 133.6, 129.8, 129.6, 125.7, 50.9, 46.3, 37.5, 34.6, 32.5 (2C), 32.1, 22.9, 19.8, 15.4, 4.3, 2.1.

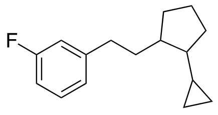

Vicinal carbocycle (8) was prepared according to Method A. The following amounts of reagents were used: dimesylate **SI-4** (67 mg, 0.16 mmol, 1.0 equiv), Ni(cod)$_2$ (4.4 mg, 16. µmol, 10 mol %), *rac*-BINAP (10. mg, 16. µmol, 10 mol %), MeMgI (0.11 mL, 0.32 mmol, 2.0 equiv, 2.9 M in Et$_2$O), PhMe (1.0 mL). Before purification, a ^{1}H NMR yield of 65% was obtained based on comparison of PhTMS as an internal standard. Purification by column chromatography (100% pentanes) afforded the title compound as a colorless oil (13 mg, 50. µmol, 34% yield, 1:1 dr). The dr was determined by the integration of resonances attributed to the aromatic carbons in ^{13}C NMR. **TLC R$_f$** = 0.8 (100% hexanes); **^{1}H NMR** (500 MHz, CDCl$_3$) δ 7.22 (aq, *J* = 7.3 Hz, 2H, both diastereomers), 6.97 (d, *J* = 7.5 Hz, 2H, both diastereomers), 6.91–6.83 (m, 4H, both diastereomers), 2.71–2.54 (m, 4H, both diastereomers), 2.00–1.12 (m, 20H, both diastereomers), 0.59–0.29 (m, 6H, both diastereomers), 0.15–0.09 (m, 2H, both diastereomers), 0.01–0.05 (m, 2H); **^{13}C NMR** (125 MHz, CDCl$_3$) δ 163.0 (d, *J* = 245.1 Hz, 2C, both diastereomers), 146.1 (d, *J* = 7.1 Hz, one diastereomer), 145.9 (d, *J* = 7.2 Hz, other diastereomer), 129.7 (d, *J* = 8.3 Hz, 2C, both diastereomers), 124.1 (d, *J* = 2.6 Hz, 2C, both diastereomers), 115.27 (d, *J* = 20.9 Hz, one diastereomer), 115.24 (d, *J* = 20.5 Hz, other diastereomer), 112.5 (d, *J* = 21.0 Hz, 2C, both diastereomers), 51.0 (one diastereomer), 48.4 (other diastereomer), 46.1 (one diastereomer), 43.1 (other diastereomer), 37.0 (one diastereomer), 35.24 (other diastereomer), 34.89 (one diastereomer), 33.0 (other diastereomer), 32.5 (one diastereomer), 32.1 (other diastereomer), 31.7 (one diastereomer). 30.7 (other diastereomer), 23.6 (one diastereomer), 23.0 (other diastereomer), 15.4 (one diastereomer), 11.8 (other diastereomer), 5.5 (one diastereomer), 4.4 (other diastereomer), 3.0 (one diastereomer), 2.2 (other diastereomer); **^{19}F NMR** (565 MHz, CDCl$_3$) δ -114.1 (aq, *J* = 8.2 Hz, 2F, both diastereomers); **HRMS** (TOF MS CI+) *m/z*: [M]$^+$ calcd for $C_{16}H_{21}F$, 232.1627; found 232.1635.

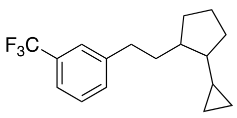

Vicinal carbocycle (9) was prepared according to Method A. The following amounts of reagents were used: dimesylate **SI-5** (43 mg, 92 µmol, 1.0 equiv), Ni(cod)$_2$ (2.5 mg, 9.2 µmol, 10 mol %), *rac*-BINAP (5.7 mg, 9.2 µmol, 10 mol %), MeMgI (70. µL, 0.18 mmol, 2.0 equiv, 2.5 M in Et$_2$O), and PhMe (0.50 mL). Purification by column chromatography (100% hexanes) afforded the title compound as a clear and colorless oil (21 mg, 74 µmol, 80% yield, 2:1 dr). The dr was determined by the integration of resonances attributed to the aromatic carbons in ^{13}C NMR. **TLC R$_f$** = 0.8 (10% EtOAc/hexanes); **^{1}H NMR** (600 MHz, CDCl$_3$) δ 7.47–7.41 (m, 4H, both diastereomers), 7.40–7.36 (m, 4H, both diastereomers), 2.74 (ddt, *J* = 13.7, 10.3, 5.1 Hz, 2H, both diastereomers), 2.65 (ddt, *J* = 13.8, 10.5, 6.8 Hz, 2H, both diastereomers), 2.07–1.88 (m, 4H, both diastereomers), 1.87–1.67 (m, 4H, diastereomers), 1.66–1.55 (m, 2H, both diastereomers), 1.54–1.48 (m, 2H, both diastereomers), 1.47–1.30 (m, 4H, both diastereomers), 1.28–1.19 (m, 1H, major diastereomer), 1.15 (dq, *J* = 11.5, 6.2 Hz, 1H, minor diastereomer), 1.00–0.79 (m, 2H, both diastereomers), 0.56 (dddd, *J* = 14.9, 8.1, 5.7, 4.0 Hz, 2H, both diastereomers), 0.52–0.46 (m, 1H, major diastereomers), 0.44 (ddd, *J* = 8.2, 5.4, 4.2 Hz, 1H, minor diastereomer), 0.39 (dddd, *J* = 9.3, 8.2, 5.4, 4.1 Hz, 1H, major diastereomer), 0.34 (dddd, *J* = 9.4, 8.3, 5.4, 4.2 Hz, 1H, minor diastereomer), 0.13 (dt, *J* = 9.8, 4.9 Hz, 2H, both diastereomers), 0.04 to -0.04 (m, 2H, both diastereomers); **^{13}C NMR** (151 MHz, CDCl$_3$) δ 144.3 (major diastereomer), 144.2 (minor diastereomer), 131.91 (q, *J* = 1.7 Hz, major diastereomer), 131.89 (q, *J* = 1.7 Hz, minor diastereomer), 130.7 (q, *J* = 31.5 Hz, 2C, both diastereomers), 128.8 (2C, both diastereomers), 125.2 (q, *J* = 3.3 Hz, major diastereomer), 125.1 (q, *J* = 3.9 Hz, minor diastereomer), 124.5 (q, *J* = 272.6 Hz, 2C, both diastereomers), 122.6 (q, *J* = 3.9 Hz, 2C, both diastereomers), 50.9 (minor diastereomer), 48.4 (major diastereomer), 46.2 (minor diastereomer), 43.1 (major diastereomer), 37.1 (minor diastereomer), 35.3 (major diastereomer), 35.0 (minor diastereomer), 33.1 (major diastereomer), 32.6 (minor diastereomer), 32.1 (minor diastereomer), 31.7 (major diastereomer), 30.7 (major diastereomer), 23.6 (minor diastereomer), 23.0 (major diastereomer), 15.4 (minor diastereomer), 11.8 (major diastereomer), 5.5 (major diastereomer), 4.4 (minor diastereomer), 3.1 (major diastereomer), 2.3 (minor diastereomer);**^{19}F NMR** (565 MHz, CDCl$_3$) δ -62.5 (3F, major diastereomer), -62.6 (3F, minor diastereomer); **HRMS** (TOF MS CI+) *m/z*: $[M]^+$ calcd for $C_{17}H_{21}F_3$, 282.1595, found 282.1595.

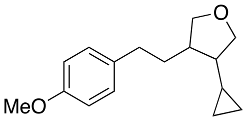

Vicinal carbocycle (10) was prepared according to Method A. The following amounts of reagents were used: dimesylate **SI-6** (58 mg, 0.13 mmol, 1.0 equiv), Ni(cod)$_2$ (3.6 mg, 13 µmol, 10. mol %), *rac*-BINAP (8.1 mg, 13 µmol, 10 mol %), MeMgI (90. µL, 0.26 mmol, 2.0 equiv, 2.9 M in Et_2O), and PhMe (0.70 mL). Before purification a ^{1}H NMR yield of 28% yield was obtained based on comparison to PhTMS as an internal standard. The residue was purified by column chromatography (0–10% EtOAc/hexanes) to afford the title compound as a clear and colorless oil (8.2 mg, 33 µmol, 27% yield, 1:1 dr). The dr was determined by the integration of resonances attributed to the aromatic carbons in ^{13}C NMR. **TLC R$_f$** = 0.2 (10% EtOAc/Hexanes, CAM Stain); **^{1}H NMR** (600 MHz, $CDCl_3$) δ 7.10 (at, *J* = 8.9 Hz, 4H, both diastereomers), 6.84 (dd, *J* = 8.9, 3.4 Hz, 4H, both diastereomers), 4.03 (dd, *J* = 8.4, 7.4 Hz, 1H, one diastereomer), 3.93 (dt, *J* = 20.4, 7.6 Hz, 2H, both diastereomers), 3.88 (dd, *J* = 8.3, 6.4 Hz, 1H, one diastereomer), 3.79 (s, 6H, both diastereomers), 3.69 (dd, *J* = 8.3, 4.6 Hz, 1H, one diastereomer), 3.59–3.53 (m, 2H, both diastereomers), 3.44 (dd, *J* = 8.4, 7.7 Hz, 1H, one diastereomer), 2.62–2.52 (m, 4H, both diastereomers), 2.23 (dq, *J* = 12.9, 7.6 Hz, 1H, one diastereomer), 2.07–1.87 (m, 3H, both diastereomer), 1.70 (dtd, *J* = 13.4, 9.1, 6.5 Hz, 1H, one diastereomer), 1.56–1.50 (m, 1H, one diastereomer), 1.49–1.42 (m, 1H, one diastereomer), 1.20 (dq, *J* = 9.0, 7.4 Hz, 1H, one diastereomer), 0.74–0.67 (m, 1H, one diastereomer), 0.67–0.61 (m, 1H, one diastereomer), 0.57 (dddd, *J* = 9.7, 8.2, 5.5, 4.4 Hz, 1H, one diastereomer), 0.52–0.44 (m, 2H, both diastereomers), 0.41 (dddd, *J* = 9.3, 8.0, 5.4, 4.4 Hz, 1H, one diastereomer), 0.18 (dquint *J* = 10.1, 5.2 Hz, 2H, both diastereomers), 0.05 (td, *J* = 9.6, 4.8 Hz, 2H, both diastereomers); **^{13}C NMR** (151 MHz, $CDCl_3$) δ 157.93 (one diastereomer), 157.92 (one diastereomer), 134.5 (one diastereomer), 134.4 (one diastereomer), 129.33 (2C, one diastereomer), 129.29 (2C, one diastereomer), 113.9 (4C, both diastereomers), 74.2 (one diastereomer), 73.7 (one diastereomer), 73.4 (one diastereomer), 72.7 (one diastereomer), 55.4 (2C, both diastereomers), 50.8 (one diastereomer), 47.9 (one diastereomer), 45.7 (one diastereomer), 42.4 (one diastereomer), 35.4 (one diastereomer), 34.4 (one diastereomer), 34.1 (one diastereomer), 30.6 (one diastereomer), 13.3 (one diastereomer), 10.0 (one diastereomer), 5.0 (one diastereomer), 3.7 (one diastereomer), 3.1 (one diastereomer), 2.7 (one diastereomer); **HRMS** (TOF MS CI+) *m/z*: $[M]^+$ calcd for $C_{16}H_{22}O_2$, 246.1620, found 246.1614.

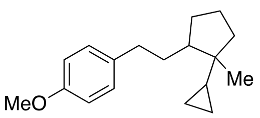

Vicinal carbocycle (11) was prepared according to Method A. The following amount of reagents were used: dimesylate **SI-7** (27 mg, 60. µmol, 1.0 equiv), Ni(cod)$_2$ (1.7 mg, 6.0 µmol, 10 mol %), *rac*-BINAP (3.7 mg, 6.0 µmol, 10 mol %), MeMgI (40. µL, 0.12 mmol, 2.0 equiv, 3.0 M in Et_2O), PhMe (0.30 mL), AD-mix-β (84 mg, 1.4 g/mmol), *t*-BuOH (1.0 mL) and H_2O (1.0 mL). Purification by column chromatography (0–10% EtOAc/hexanes) afforded the title compound as a pale yellow oil (6.0 mg, 23 µmol, 40% yield, 1.5:1 dr). The dr was determined by the integration of resonances attributed to the aromatic carbons in ^{13}C NMR. **TLC R$_f$** = 0.7 (10% EtOAc/hexanes, CAM Stain); **^{1}H NMR** (600 MHz, $CDCl_3$) δ 7.12 (d, *J* = 8.4 Hz, 4H, both diastereomers), 6.83 (dd, *J* = 9.1, 3.0 Hz, 4H, both diastereomers), 3.79 (s, 6H, both diastereomers), 2.66 (ddd, *J* = 15.0, 10.6, 4.9 Hz, 1H, minor diastereomer), 2.45 (ddd, *J* = 13.8, 10.2, 6.4 Hz, 1H, major diastereomers), 1.94 (qd, *J* = 10.1, 9.0, 1H, major diastereomer), 1.83 (dddd, *J* = 13.3, 9.9, 6.4, 3.0 1H, minor diastereomer), 1.70–1.55 (m, 2H, both diastereomers), 1.52–1.43 (m, 2H, both diastereomers), 1.44–1.36 (m, 2H, both diastereomers), 1.35–1.24 (m, 6H, both diastereomers), 1.21 (ddd, *J* = 12.8, 6.4, 3.3 Hz, 2H, both diastereomers), 0.91–0.79 (m, 4H, both diastereomers), 0.73 (s, 3H, major diastereomer), 0.69 (s, 3H, minor diastereomer), 0.67–0.62 (m, 2H, both diastereomers), 0.24 (dqd, *J* = 13.0, 8.9, 4.2 Hz, 4H, both diastereomers), 0.18–0.08 (m, 4H, both diastereomers); **^{13}C NMR** (151 MHz, $CDCl_3$) δ 157.72 (minor diastereomer), 157.70 (major diastereomer), 135.59 (major diastereomer), 135.58 (minor diastereomer), 129.4 (2C, minor diastereomer), 129.3 (2C, major diastereomer), 113.80 (2C, major diastereomer), 113.78 (2C, minor diastereomer), 55.4 (2C, both diastereomers) 51.1 (2C, both diastereomers), 43.1 (2C, both diastereomers), 38.1 (2C, both diastereomers), 34.8 (2C, both diastereomers), 32.7 (2C, both diastereomers), 31.1 (2C, both diastereomers), 23.4 (2C, both diastereomers), 22.0 (2C, both diastereomers), 15.6 (2C, both diastereomers) 1.2 (minor diastereomer), 1.1 (major diastereomer), 0.3 (major diastereomer), 0.2 (minor diastereomer); **HRMS** (TOF MS CI+) *m/z*: [M]$^+$ calcd for $C_{18}H_{26}O$, 258.1984, found 258.1980.

c. Substrates that Provide Low Yields of Desired Product

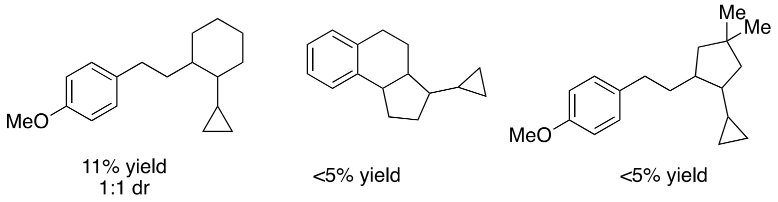

d. Characterization Data for By-Products Formed from Optimization Studies

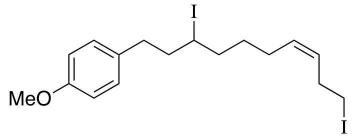

Diiodide (3a) was observed as a by-product from the optimization studies as a 2:1 mixture of alkene diastereomers (see Table 1). Below is the characterization of the title compound. For clarity the ^{1}H and ^{13}C data are tabulated separately. **TLC R$_f$** = 0.7 (10% EtOAc/hexanes); **HRMS** (TOF MS CI+) *m/z*: $[M]^+$ calcd for $C_{17}H_{24}I_2O$, 497.9917; found 497.9906.

Major Diastereomer: ^{1}H NMR (400 MHz, $CDCl_3$) δ 7.12 (d, *J* = 8.7 Hz, 2H), 6.85 (d, *J* = 8.6 Hz, 2H), 5.59–5.42 (m, 1H), 5.40–5.28 (m, 1H), 4.04 (tt, *J* = 8.8, 4.3 Hz, 1H), 3.80 (s, 3H), 3.15 (td, *J* = 7.2, 4.0 Hz, 2H), 2.84 (ddd, *J* = 13.9, 8.9, 5.0 Hz, 1H), 2.74–2.65 (m, 1H), 2.62 (aq, *J* = 7.2, 6.1 Hz, 2H), 2.15 (dtd, *J* = 14.3, 9.1, 5.0 Hz, 1H), 2.09–1.84 (m, 4H), 1.81–1.69 (m, 1H), 1.68–1.57 (m, 1H), 1.54–1.42 (m, 1H); **^{13}C NMR** (101 MHz, $CDCl_3$) δ 158.1, 133.0, 131.8, 129.6 (2C), 128.7, 114.1 (2C), 55.4, 42.6, 40.3, 39.0, 34.8, 31.6, 29.4, 26.7, 5.5.

Minor Diastereomer: ^{1}H NMR (400 MHz, $CDCl_3$) δ 7.12 (d, *J* = 8.7 Hz, 2H), 6.85 (d, *J* = 8.6 Hz, 2H), 5.59–5.42 (m, 1H), 5.40–5.28 (m, 1H), 4.04 (tt, *J* = 8.8, 4.3 Hz, 1H), 3.80 (s, 3H), 3.15 (td, *J* = 7.2, 4.0 Hz, 2H), 2.84 (ddd, *J* = 13.9, 8.9, 5.0 Hz, 1H), 2.74–2.65 (m, 1H), 2.55 (aq, *J* = 7.2, 6.7 Hz, 2H), 2.15 (dtd, *J* = 14.3, 9.1, 5.0 Hz, 1H), 2.09–1.84 (m, 4H), 1.81–1.69 (m, 1H), 1.68–1.57 (m, 1H), 1.54–1.42 (m, 1H); **^{13}C NMR** (101 MHz, $CDCl_3$) δ 158.1, 132.8, 131.8, 129.6 (2C), 129.2, 114.0 (2C), 55.4, 42.6, 40.1, 39.2, 36.7, 31.7, 29.1, 26.7, 6.3.

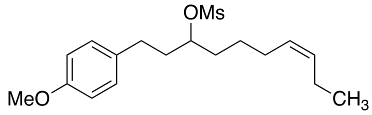

Reduction Product (4) was observed as a by-product from the optimization studies (see Table 1). Below is the characterization for the major diastereomer. **^{1}H NMR** (500 MHz, $CDCl_3$) δ 7.11 (d, *J* = 8.2 Hz, 2H), 6.84 (d, *J* = 8.4 Hz, 2H), 5.57–5.22 (m, 2H), 4.75 (sextet, *J* = 6.1 Hz, 1H), 3.79 (s, 3H), 2.99 (s, 3H), 2.67 (qdd, *J* = 14.1, 9.5, 6.1 Hz, 2H), 2.15–1.87 (m, 4H), 1.74 (tt, *J* = 9.1, 6.0 Hz, 2H), 1.68–1.52 (m, 1H), 1.52–1.14 (m, 3H), 0.96 (tdd, *J* = 7.5, 3.5, 1.1 Hz, 3H); **^{13}C NMR** (126 MHz, $CDCl_3$) δ 158.1, 133.1, 132.7, 129.4 (2C), 128.2, 114.1 (2C), 83.4, 55.4, 38.9, 36.5, 32.2, 30.5, 26.8, 24.9, 20.7, 14.1; **HRMS** (TOF MS ES+) *m/z*: [M+Na] calcd for $C_{18}H_{28}O_4S$, 363.1606; found 363.1615.

e. General Procedures for the Synthesis of Dimesylate Starting Materials

Method B: Pd-Catalyzed Heck Reaction

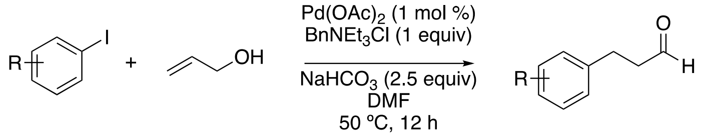

(SI-3)

This method was adapted from a procedure reported by Su.[4] A flame dried pressure tube equipped with a stir bar was charged with aryl iodide[5] (1.0 equiv), $NaHCO_3$ (2.5 equiv), and $BnNEt_3Cl$ (1.0 equiv). The flask was sealed with a septum and pumped into a glovebox. Then the pressure tube was charged with $Pd(OAc)_2$ (1.0 mol %), sealed with septum, removed from the glovebox and placed under an atmosphere of N_2. Then DMF (0.20 M) was added via syringe followed by allyl alcohol (1.5 equiv). The pressure tube was sealed with a teflon cap, heated in an oil bath to 50 ºC and allowed to stir at that temperature for at least 5 h. After the reaction was complete, the flask was cooled to rt and allowed to stir at that temperature for 1 h. The mixture was then filtered through a pad of silica gel eluting with EtOAc. The filtrate was concentrated to remove the excess EtOAc and then transferred to a separatory funnel. The organic layer was washed with excess H_2O (x 5) and then brine. The combined organic layers were dried over Na_2SO_4, filtered, and concentrated in vacuo. The product was purified by column chromatography.

Method C: Preparation of Grignard Reagent

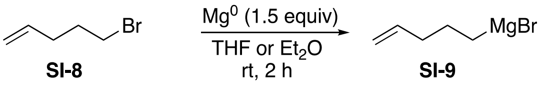

(SI-4)

To a flame dried two-neck round bottom flask equipped with a stir bar, was added magnesium turnings (1.5 equiv). The flask and magnesium turnings were then flame-dried under vacuum and the flask was back-filled with N_2. Anhydrous Et_2O or THF (2.0 M) and a crystal of iodine (ca. 2.0 mg) were added to the flask. Then alkyl bromide **SI-8** (1.0 equiv) was slowly added neat over 30 min to maintain a gentle reflux. The mixture was stirred for 2 h at room temperature The resulting Grignard reagent was titrated according to Knochel's method and used immediately.[2]

Method D: Nucleophilic Grignard Addition into Aldehyde

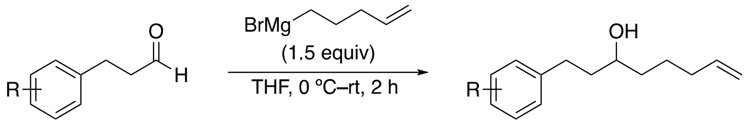

(SI-5)

To a flame dried round bottom flask equipped with a stir bar, was added aldehyde (1.0 equiv) as a solution in THF or Et_2O (0.20 M). The flask was cooled to 0 ºC and the freshly prepared Grignard reagent (1.5 equiv) was added dropwise via syringe. The flask was warmed to rt and allowed to stir for 2 h. To quench, saturated aq. NH_4Cl was added to the reaction mixture. The biphasic solution was transferred to a separatory funnel and the organic layer was extracted with EtOAc. The combined organic layers were washed with H_2O, brine, dried over Na_2SO_4, filtered, and concentrated in vacuo.

Method E: Ozonolysis of Terminal Alkenes

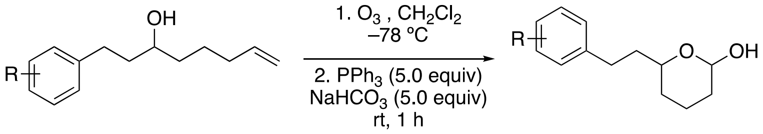

(SI-6)

This method was adapted from a procedure reported by Taber.[6] A round-bottom flask equipped with stir bar was charged with alkene (1.0 equiv), and CH_2Cl_2 (0.10 M). The reaction was then cooled to –78 ºC, and ozone was bubbled through mixture until the solution turned blue. Ozone flow was then discontinued, and O_2 was passed through the solution for 5 min. PPh_3 (5.0 equiv) and $NaHCO_3$ (5.0 equiv) were then added to flask and the mixture was allowed to stir for at least 1 h. Then H_2O was added and the biphasic mixture was transferred to a separatory funnel. The organic layer was extracted with CH_2Cl_2 (x 3). The combined organic layers were washed with brine, dried over Na_2SO_4, filtered, and concentrated in vacuo.

Method F: Preparation of Wittig Salt SI-11

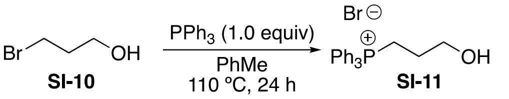

(SI-7)

To a flame dried round bottom flask equipped with a stir bar and reflux condenser was added 3-bromopropanol **SI-10** (1.0 equiv) and PhMe (1.1 M). Then PPh_3 (1.0 equiv) was added, the reaction mixture was heated in an oil bath to reflux and allowed to stir until white precipitate formed (~15–24 h). The flask was cooled to rt, the precipitate was filtered and washed with hexanes. The white solids were collected and dried under vacuum overnight.

Method G: Wittig Reaction

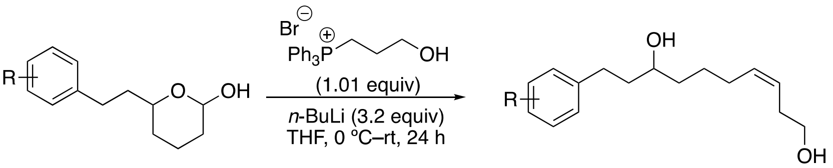

(SI-8)

This method was adapted from a procedure reported by Taber.[6] To a flame dried round bottom flask equipped with stir bar was added the Wittig salt **SI-11** (1.01 equiv) and THF (0.2 M). The flask is cooled to 0 ºC and *n*-BuLi (3.2 equiv, 2.5 M in hexanes) was added dropwise. The dark red solution was allowed to stir at 0 ºC for 1 h. Then, a solution of lactol (1.0 equiv) in THF (1.0 M) was added and the reaction mixture was warmed to rt. The resulting orange solution was allowed to stir at rt for 24 h. To quench, saturated aq. NH_4Cl was added dropwise. The biphasic mixture was transferred to a separatory funnel. The organic layer was extracted with EtOAc (x 3). The combined organic layers were washed with brine, dried over Na_2SO_4, filtered, and concentrated in vacuo.

Method H: Mesylation Reaction

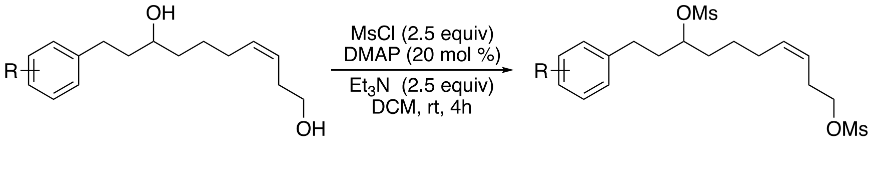

(SI-9)

A flame dried round bottom flask equipped with a stir bar was charged with diol (1.0 equiv) and CH_2Cl_2 (0.20 M) under N_2. Then, Et_3N (1.5 equiv), DMAP (20 mol %), and MsCl (2.5 equiv) were added in that order. The reaction mixture was then stirred at rt for at least 4 h. Once complete, sat. $NaHCO_3$ was added

and the reaction mixture was extracted with CH_2Cl_2 (x 3). The combined organic layers were washed with brine, dried over Na_2SO_4, and concentrated in vacuo.

Method I: TBS Protection of Alcohols

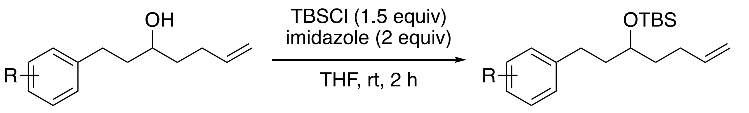

(SI-10)

This method was adapted from a procedure reported by Jia.[7] To a flame dried round bottom flask equipped with a stir bar was added alcohol (1.0 equiv), imidazole (2.0 equiv), *tert*-butyldimethylsilyl chloride (1.5 equiv), and CH_2Cl_2 (0.20 M). The reaction mixture was allowed to stir at rt for 24 h. After the reaction was complete, H_2O was added and the mixture was transferred to a separatory funnel. The mixture was extracted with CH_2Cl_2 (x 3). The combined organic layers were washed with brine, dried over Na_2SO_4, and concentrated in vacuo.

Method J: Hydroboration Oxidation of Terminal Alkenes

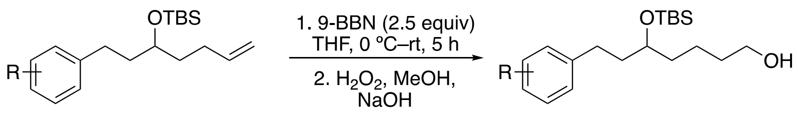

(SI-11)

This method was adapted from a procedure reported by Hartwig.[8] To a flame dried round bottom flask equipped with a stir bar was added silyl protected alcohol (1.0 equiv) and THF (0.20 M). The reaction mixture was cooled to 0 ºC and 9-BBN (2.5 equiv) was added dropwise via syringe. The mixture was warmed to rt and allowed to stir for at least 5 h. Then MeOH (3 mL/mmol), H_2O_2 (30%, 1 mL/mmol), and NaOH (1.0 M, 1 mL/mmol) were added, and the reaction was allowed to stir for at least 3 h. Once complete, H_2O (10 mL) was added. The reaction mixture was then extracted with EtOAc (x 3) and combined organic layers were washed with brine, dried over Na_2SO_4, and concentrated in vacuo.

Method K: Parikh–Doering Oxidation

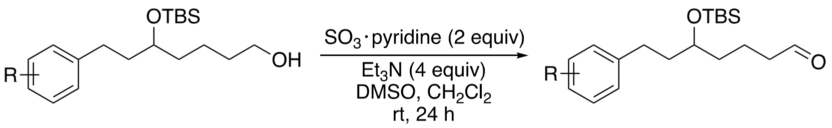

(SI-12)

This method was adapted from a procedure reported by Nicolaou.[9] To a flame dried round bottom flask equipped with a stir bar was added primary alcohol (1.0 equiv), SO_3·pyridine (2.0 equiv), Et_3N (4.0 equiv), DMSO (0.50 M), and CH_2Cl_2 (0.50 M). The reaction mixture was allowed to stir at rt for 24 h. Once complete, saturated aq. NH_4Cl (10 mL) was added. The reaction mixture was then extracted with CH_2Cl_2 (x 3) and combined organic layers were washed with brine, dried over Na_2SO_4, and concentrated in vacuo.[10]

Method L: Corey-Fuchs Reaction

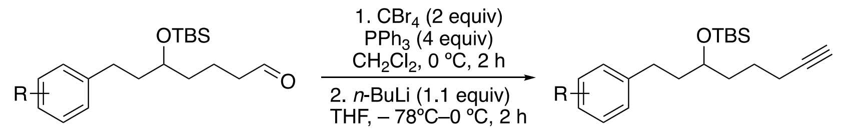

(SI-13)

This method was adapted from a procedure reported by Fürstner and Ghosh.[11] To a flame dried round bottom flask equipped with a stir bar was added aldehyde (1.0 equiv), CBr_4 (2.0 equiv), and CH_2Cl_2 (0.20 M). The reaction mixture was cooled to 0 ºC and then PPh_3 (4.0 equiv) was added dropwise as a solution in CH_2Cl_2 (0.60 M). The reaction was allowed to stir at 0 ºC for 2 h. To quench, hexanes was added and the reaction mixture was filtered through a pad of silica gel eluting with 50% Et_2O in hexanes. The filtrate was concentrated and carried into the next step without further purification.

The crude dibromide (1.0 equiv) was dissolved in THF (0.20 M) and the mixture was cooled to – 78 ºC. Then *n*-BuLi (1.1 equiv) was added dropwise and the mixture was allowed to slowly warm to 0 ºC over 2 h. To quench, saturated aq. NH_4Cl was added dropwise and the biphasic mixture was transferred to separatory funnel. The organic layer was extracted with EtOAc (x 3). The combined organic layers were washed sequentially with H_2O and brine, dried over Na_2SO_4, filtered, and concentrated in vacuo.

Method M: Silyl Deprotection with Tetrabutylammonium fluoride (TBAF)

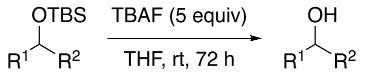

(SI-14)

This method was adapted from a procedure reported by Knochel.[12] To a flame dried round bottom flask equipped with a stir bar was added silyl protected alcohol (1.0 equiv) and THF (0.20 M). Then, tetrabutyl ammonium fluoride (TBAF, 5.0 equiv, 1.0 M in THF) was added dropwise at rt. The reaction mixture was allowed to stir at rt for 72 h. The reaction was quenched with H_2O. The biphasic mixture was transferred to a separatory funnel and the product was extracted with EtOAc (x 3). The combined organic layers were washed with brine, dried over Na_2SO_4, filtered, and concentrated in vacuo.

f. Synthesis and Characterization Data for Dimesylate Starting Materials

Scheme SI-1 General Synthesis of Dimesylate Starting Material

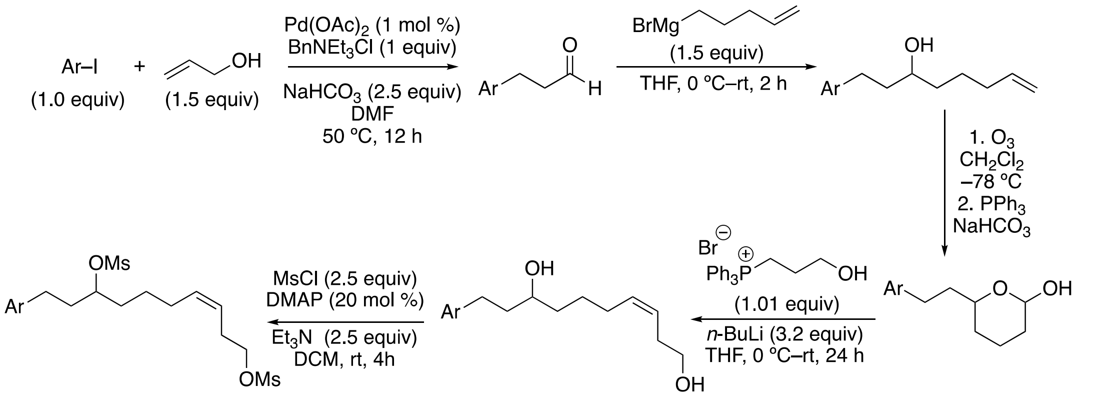

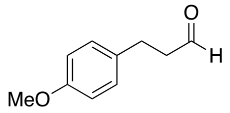

Aldehyde (SI-12) was prepared according to Method B. The following amounts of reagents were used: 4-iodoanisole (4.9 g, 20. mmol, 1.0 equiv), allyl alcohol (2.0 mL, 30. mmol, 1.5 equiv), $Pd(OAc)_2$ (45 mg, 0.20 mmol, 1.0 mol %), $BnEt_3NCl$ (4.6 g, 20. mmol, 1.0 equiv), $NaHCO_3$ (4.3 g, 50. mmol, 2.5 equiv), and DMF (80 mL). Purification by column chromatography (0–10% EtOAc/hexanes) afforded the title compound as a clear and colorless oil (2.6 g, 16 mmol, 81% yield). Analytical data is consistent with literature values.[13] **^{1}H NMR** (400 MHz, $CDCl_3$) δ 9.75 (t, J = 1.5 Hz, 1H), 7.07 (d, J = 8.9 Hz, 2H), 6.81 (d, J = 8.7 Hz, 2H), 3.74 (s, 3H), 2.86 (t, J = 7.6 Hz, 2H), 2.73 – 2.65 (m, 2H).

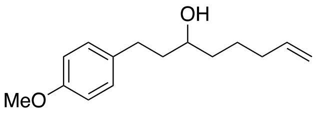

Alcohol (SI-13) was prepared according to Method D. The following amount of reagents were used: aldehyde **SI-12** (1.4 g, 8.6 mmol, 1.0 equiv), Grignard reagent **SI-9** (10. mL, 13 mmol, 1.5 equiv, 1.3 M in Et_2O) and Et_2O (43 mL, 0.20 M). Purification by column chromatography (0–30% EtOAc/hexanes) afforded the title compound as a pale yellow oil (1.9 g, 8.1 mmol, 94% yield). Analytical data consistent with literature values.[6] **TLC R$_f$** = 0.3 (20% EtOAc/hexanes, $KMnO_4$ stain); **^{1}H NMR** (400 MHz, $CDCl_3$) δ 7.15–7.10 (m, 2H), 6.87–6.81 (m, 2H), 5.81 (ddt, J = 16.9, 10.1, 6.6 Hz, 1H), 5.02 (dq, J = 17.1, 1.7 Hz, 1H), 4.97 (dq, J = 10.2, 1.7 Hz, 1H), 3.79 (s, 3H), 3.68–3.57 (m, 1H), 2.68 (dddd, J = 46.8, 13.8, 9.5, 6.3 Hz, 2H), 2.12–2.04 (m, 2H), 1.82–1.66 (m, 2H), 1.60–1.39 (m, 4H).

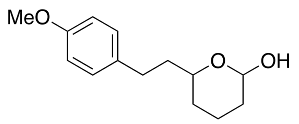

Lactol (SI-14) was prepared according to Method E. The following amounts of reagents were used: alcohol **SI-13** (0.42 g, 1.8 mmol, 1.0 equiv), ozone (excess), CH_2Cl_2 (18 mL), PPh_3 (0.85 g, 2.0 g/1g of alcohol), and $NaHCO_3$ (0.85 g, 2.0 g/1g of alcohol). Purification by column chromatography (0–50% EtOAc/hexanes) afforded the title compound as a white solid (0.19 g, 0.83 mmol, 46% yield, 1.5:1 mixtures of diastereomers). For clarity, the ^{1}H and ^{13}C of the major and minor diastereomers have been tabulated individually. The analytical data is consistent with literature values.[6]

TLC R_f = 0.3 (20% EtOAc/hexanes, CAM stain); **HRMS** (TOF MS ES+) *m/z*: $[M+Na]^+$ calcd for $C_{14}H_{20}O_3Na$, 259.1310; found 259.1309.

Major diastereomer: ^{1}H NMR (500 MHz, $CDCl_3$) δ 7.09 (d, *J* = 8.5 Hz, 2H), 6.82 (d, *J* = 8.4 Hz, 2H), 4.69 (t, *J* = 7.7 Hz, 1H), 3.78 (s, 3H), 3.46–3.34 (m, 1H), 3.19 (s, 1H), 2.65–2.51 (m, 2H), 1.92–1.79 (m, 2H), 1.79–1.55 (m, 3H), 1.54–1.43 (m, 1H), 1.38–1.15 (m, 2H); **^{13}C NMR** (126 MHz, $CDCl_3$) δ 157.8, 134.3, 129.4 (2C), 113.9 (2C), 96.6, 75.6, 55.4, 38.0, 33.1, 30.9, 30.6, 22.2.

Minor Diastereomer: ^{1}H NMR (500 MHz, $CDCl_3$) δ 7.09 (d, *J* = 8.5 Hz, 2H), 6.82 (d, *J* = 8.4 Hz, 2H), 5.33 (s, 1H), 3.98–3.90 (m, 1H), 3.78 (s, 3H), 3.19 (s, 1H), 3.14 (d, *J* = 6.2 Hz, 1H), 2.79–2.66 (m, 1H), 1.92–1.79 (m, 2H), 1.79–1.55 (m, 3H), 1.56–1.42 (m, 1H), 1.38–1.15 (m, 2H); **^{13}C NMR** (126 MHz, $CDCl_3$) δ 157.8, 134.5, 129.4 (2C), 113.9 (2C), 92.1, 68.1, 55.4, 38.2, 31.3, 30.9, 30.0, 17.6.

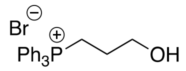

Wittig Salt (SI-11) was prepared according to Method F. The following amounts of reagents were used: 4-bromoproanol **SI-10** (0.89 mL, 10. mmol, 1.0 equiv), PPh_3 (2.6 g, 10. mmol, 1.0 equiv), and PhMe (9.1 mL). Analytical data is consistent with literature data.[14] **^{1}H NMR** (400 MHz, $CDCl_3$) δ 7.84–7.75 (m, 9H), 7.74–7.67 (m, 6H), 4.93 (s, 1H) 3.94–3.62 (m, 4H), 1.95–1.74 (m, 2H).

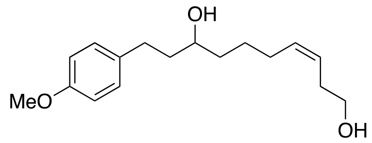

Diol (SI-15) was prepared according to Method G. The following amounts of reagents were used: lactol **SI-14** (0.19, 0.83 mmol, 1.0 equiv), Wittig salt **SI-11** (0.34 g, 0.84 mmol, 1.0 equiv), *n*-BuLi (1.1 mL, 2.7 mmol, 3.2 equiv, 2.5 M in hexane) and THF (4.2 mL). Purification by column chromatography (0–70% EtOAc/hexanes) afforded the title compound as a yellow oil (0.16 g, 0.57 mmol, 69% yield, 2:1 mixture of diastereomers) with a small amount of triphenylphosphine oxide. For clarity, the ^{1}H and ^{13}C of the major and minor diastereomers have been tabulated individually.

TLC R_f = 0.2 (20% EtOAc/hexanes, $KMnO_4$ stain); **HRMS** (TOF MS ES+) *m/z*: $[M+Na]^+$ calcd for $C_{17}H_{26}O_3Na$, 301.1780; found 301.1788.

Major diastereomer: ^{1}H NMR (500 MHz, $CDCl_3$) δ 7.18 (d, *J* = 8.4 Hz, 2H), 6.89 (d, *J* = 8.5 Hz, 2H), 5.62 (dt, *J* = 10.7, 7.5 Hz, 1H), 5.45 (dtt, *J* = 10.5, 7.3, 1.5 Hz, 1H), 3.85 (s, 3H), 3.70 (t, *J* = 6.5 Hz, 2H), 2.79 (ddd, *J* = 14.8, 9.5, 5.8 Hz, 1H), 2.68 (ddd, *J* = 13.9, 9.4, 6.9 Hz, 1H), 2.37 (q, *J* = 7.0 Hz, 2H), 2.14 (tquint, *J* = 13.9, 7.5 Hz, 2H), 1.89–1.71 (m, 2H), 1.66 (br s, 5H), 1.61–1.41 (m, 2H); **^{13}C NMR** (126 MHz, $CDCl_3$) δ 157.9, 134.3, 133.0, 129.4 (2C), 125.6, 114.0 (2C), 71.3, 62.4, 55.4, 39.5, 37.1, 31.3, 30.9, 27.4, 25.8.

Minor Diastereomer: **^{1}H NMR** (500 MHz, $CDCl_3$) δ 7.18 (d, J = 8.5 Hz, 2H), 6.89 (d, J = 8.6 Hz, 2H), 5.60 (dt, J = 15.3, 6.7 Hz, 1H), 5.45 (dt, J = 15.3, 6.9 Hz, 1H), 3.85 (s, 3H), 3.69 (t, J = 6.3 Hz, 2H), 2.79 (ddd, J = 14.9, 9.5, 5.8 Hz, 1H), 2.68 (ddd, J = 13.9, 9.4, 6.8 Hz, 1H), 2.33 (q, J = 6.4 Hz, 2H), 2.10 (d, J = 7.1 Hz, 2H), 1.91–1.70 (m, 2H), 1.67–1.39 (m, 5H), 1.03–0.80 (m, 2H); **^{13}C NMR** (126 MHz, $CDCl_3$) δ 157.9, 134.3, 133.9, 129.4 (2C), 126.4, 114.0 (2C), 71.3, 62.2, 55.4, 39.5, 37.2, 36.1, 32.7, 31.3, 25.5.

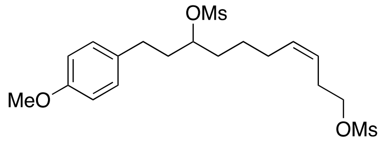

Dimesylate (1) was prepared according to Method H. The following amounts of reagents were used: diol **SI-15** (0.59 g, 2.1 mmol, 1.0 equiv), MsCl (0.41 mL, 5.3 mmol, 2.5 equiv), Et_3N (0.73 mL, 5.3 mmol, 2.5 equiv), DMAP (51 mg, 0.42 mmol, 20 mol %), and CH_2Cl_2 (11 mL). Purification by column chromatography (0–50% EtOAc/hexanes) afforded the title compound as a yellow oil (0.76 g, 1.7 mmol, 83% yield, 2:1 dr). For clarity, the ^{1}H and ^{13}C of the major and minor diastereomers have been tabulated individually.

TLC R$_f$ = 0.4 (50% EtOAc/hexanes, CAM stain); **HRMS** (TOF MS ES+) *m/z*: $[M+Na]^+$ calcd for $C_{19}H_{30}O_7S_2Na$, 457.1331; found 457.1314.

Major Diastereomer: **^{1}H NMR** (400 MHz, $CDCl_3$) δ 7.11 (d, J = 8.5 Hz, 2H), 6.83 (d, J = 8.7 Hz, 2H), 5.54 (dt, J = 10.7, 7.4 Hz, 1H), 5.38 (dt, J = 10.8, 7.2 Hz, 1H), 4.75 (quint, J = 6.0 Hz, 1H), 4.20 (t, J = 6.8 Hz, 2H), 3.78 (s, 3H), 2.99 (d, J = 1.8 Hz, 6H), 2.76–2.58 (m, 2H), 2.49 (q, J = 6.8 Hz, 2H), 2.18–1.88 (m, 4H), 1.75 (dtd, J = 9.4, 6.2, 3.9 Hz, 2H), 1.57–1.39 (m, 2H); **^{13}C NMR** (101 MHz, $CDCl_3$) δ 158.2, 133.2, 133.0, 129.5 (2C), 124.0, 114.2 (2C), 83.1, 69.4, 55.5, 39.0, 37.7, 36.6, 34.2, 30.6, 27.6, 27.1, 24.9.

Minor Diastereomer: **^{1}H NMR** (400 MHz, $CDCl_3$) δ 7.12 (d, J = 8.6 Hz, 2H), 6.85 (d, J = 8.7 Hz, 2H), 5.55 (dt, J = 15.4, 6.7 Hz, 1H), 5.40 (dt, J = 15.3, 6.6 Hz, 1H), 4.75 (quint, J = 6.1 Hz, 1H), 4.22 (t, J = 6.7 Hz, 2H), 3.79 (s, 3H), 3.00 (d, J = 1.1 Hz, 6H), 2.76–2.59 (m, 2H), 2.45 (q, J = 6.7 Hz, 2H), 2.10–1.94 (m, 3H), 1.81–1.66 (m, 2H), 1.49 (dt, J = 15.7, 7.4 Hz, 1H), 0.96 – 0.79 (m, 2H); **^{13}C NMR** (126 MHz, $CDCl_3$) δ 158.1, 133.8, 132.9, 129.4 (2C), 124.7, 114.1 (2C), 83.2, 69.6, 55.4, 38.9, 37.6, 36.5, 34.0, 32.5, 32.2, 30.5, 24.5.

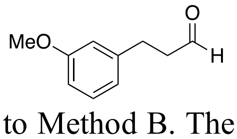

Aldehyde (SI-16) was prepared according to Method B. The following amounts of reagents were used: 3-iodoanisole (0.59 mL, 5.0 mmol, 1.0 equiv), allyl alcohol (0.51 mL, 7.5 mmol, 1.5 equiv), $Pd(OAc)_2$ (22 mg, 0.10 mmol, 2.0 mol %), $BnEt_3NCl$ (1.1 g, 5.0 mmol, 1.0 equiv), $NaHCO_3$ (1.0 g, 13 mmol, 2.5 equiv), and DMF (20 mL). Purification by column chromatography (0–10% EtOAc/hexanes) afforded the title compound as a yellow oil (0.57 g, 3.4 mmol, 69% yield). Analytical data is consistent with literature values.[15] **^{1}H NMR** (500 MHz, $CDCl_3$) δ 9.83 (t, J = 1.4 Hz, 1H), 7.22 (td, J = 7.5, 1.0 Hz, 1H), 6.80–6.75 (m, 3H), 3.81 (s, 3H), 2.95 (t, J = 7.5 Hz, 2H), 2.79 (t, J = 8.1Hz, 2H).

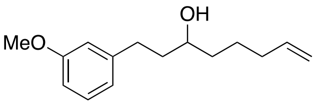

Alcohol (SI-17) was prepared according to Method D. The following amounts of reagents were used: aldehyde **SI-16** (0.57 g, 3.4 mmol, 1.0 equiv), Grignard reagent **SI-9** (5.1 mL, 5.1 mmol, 1.5 equiv, 1.0 M in THF), and THF (17 mL). Purification by column chromatography (0–10% EtOAc/hexanes) afforded the title compound as a brown oil (0.49 g, 2.1 mmol, 61% yield). **^{1}H NMR** (500 MHz, $CDCl_3$) δ 7.20 (t, *J* = 8.0 Hz, 1H), 6.79 (d, *J* = 7.5 Hz, 1H), 6.75–6.72 (m, 2H), 5.80 (ddt, *J* = 17.8, 10.1, 6.4 Hz, 1H), 4.98 (ddt, *J* = 17.1, 2.0, 1.6 Hz, 1H), 4.95 (ddt, *J* = 10.2, 2.0, 1.0 Hz, 1H), 3.80 (s, 3H), 3.63 (br s, 1H), 2.77 (ddd, *J* = 13.8, 9.7, 5.7 Hz, 1H), 2.65 (ddd, *J* = 14.0, 9.1, 7.0 Hz, 1H), 2.07 (aq, *J* = 6.1 Hz, 2H), 1.84–1.70 (m, 2H), 1.57–1.40 (m, 4H), 1.33 (br s, 1H).

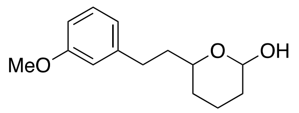

Lactol (SI-18) was prepared according to Method E. The following amounts of reagents were used: alkene **SI-17** (0.46 g, 2.0 mmol, 1.0 equiv), ozone (excess), $NaHCO_3$ (0.28 g, 3.4 mmol, 1.7 equiv), PPh_3 (0.89 g. 3.4 mmol, 1.7 equiv), and CH_2Cl_2 (2.0 mL). The crude residue was taken on with minimal purification.

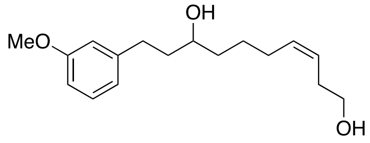

Diol (SI-19) was prepared according to method G. The following amounts of reagents were used: lactol **SI-18** (0.32 g, 1.3 mmol, 1.0 equiv), Wittig salt **SI-11** (0.53 g, 0.37 mmol, 1.01 equiv), *n*-BuLi (1.7 mL, 4.2 mmol, 3.2 equiv, 2.5 M in hexane), and THF (6.5 mL). The crude residue was taken on with minimal purification.

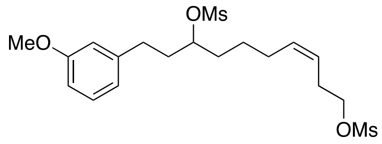

Dimesylate (SI-1) was prepared according to Method H. The following amounts of reagents were used: diol **SI-19** (190 mg, 0.67 mmol, 1.0 equiv), MsCl (0.15 mL, 2.0 mmol, 3.0 equiv), DMAP (16 mg, 0.13 mmol, 0.20 equiv), Et_3N (0.28 mL, 2.0 mmol, 3.0 equiv), and CH_2Cl_2 (3.4 mL). Purification by column chromatography (0–50% EtOAc/hexanes) afforded the title compound as a yellow oil (170 mg, 0.39 mmol, 19% yield over 3 steps, 1.5:1 dr). For clarity, the ^{1}H and ^{13}C data are tabulated individually.

TLC R_f = 0.2 (50% EtOAc/hexanes); **HRMS** (TOF MS ES+) *m/z*: $[M+Na]^+$ calcd for $C_{19}H_{30}O_7S_2Na$, 457.1331; found 457.1336.

Major Diastereomer ^{1}H NMR (600 MHz, $CDCl_3$) δ 7.21 (d, *J* = 8.2 Hz, 1H), 6.79 (d, *J* = 7.4 Hz, 1H), 6.75–6.74 (m, 2H), 5.56–5.52 (m, 1H), 5.41–5.36 (m, 1H), 4.76 (quin, *J* = 6.0 Hz, 1H), 4.20 (t, *J* = 6.7 Hz, 2H), 3.80 (s, 3H), 3.01 (s, 3H), 3.00 (s, 3H), 2.76–2.71(m, 1H), 2.70–2.65 (m,1H), 2.49 (dt, *J* = 7.1, 6.3 Hz, 2H), 2.15–1.95 (m, 4H), 1.80–1.70 (m, 2H), 1.54–1.43 (m, 2H); **^{13}C NMR** (125 MHz, $CDCl_3$) δ 159.9, 142.6, 133.1, 129.7, 123.9, 120.8, 114.3, 111.6, 82.9, 69.3, 55.3, 37.6, 36.1, 34.2, 32.5, 21.5, 27.5, 27.0, 24.8.

Minor Diastereomer ^{1}H NMR (600 MHz, $CDCl_3$) δ 7.21 (d, *J* = 8.2 Hz, 1H), 6.79 (d, *J* = 7.4 Hz, 1H), 6.75–6.74 (m, 2H), 5.56–5.52 (m, 1H), 5.41–5.36 (m, 1H), 4.76 (quint, *J* = 6.0 Hz, 1H), 4.22 (t, *J* = 6.5 Hz, 2H), 3.80 (s, 3H), 3.005 (s, 3H), 2.997 (s, 3H), 2.76–2.71(m, 1H), 2.70–2.65 (m,1H), 2.44 (dt, *J* = 6.7, 6.2 Hz, 2H), 2.15–1.95 (m, 4H), 1.80–1.70 (m, 2H), 1.54–1.43 (m, 2H); **^{13}C NMR** (125 MHz, $CDCl_3$)

δ 159.9, 142.6, 133.8, 129.7, 124.8, 120.8, 114.3, 111.6, 83.1, 69.5, 55.3, 37.6, 36.1, 34.0, 32.5, 31.5, 27.5, 27.0, 24.5.

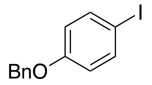

Iodide (SI-20) was prepared according to a procedure reported by Fukuyama.[16] To a round bottom flask equipped with a stir bar was added 4-iodophenol (1.1 g, 5.0 mmol, 1.0 equiv), K_2CO_3 (0.89 g, 6.5 mmol, 1.3 equiv), benzyl bromide (0.65 mL, 5.5 mmol, 1.1 equiv), and DMF (2.5 mL). The reaction flask was fitted with a reflux condenser, and the mixture was heated in an oil bath to 60 ºC and allowed to stir overnight. To quench, H_2O was added at rt and the solution was transferred to a separatory funnel. The aqueous layer was extracted with Et_2O (x 3). The combined organic layers were washed with brine, dried over Na_2SO_4, filtered, and concentrated in vacuo. Purification by column chromatography (0–10% EtOAc/hexanes) afforded the title compound as a white solid (1.2 g, 3.7 mmol, 74% yield). Analytical data is consistent with literature data.[16] **TLC R$_f$** = 0.7 (10% EtOAc/hexanes); **^{1}H NMR** (500 MHz, $CDCl_3$) δ 7.65 (d, *J* = 9.0 Hz, 2H), 7.56–7.47 (m, 4H), 7.47–7.40 (m, 1H), 6.85 (d, *J* = 8.9 Hz, 2H), 5.12 (s, 2H).

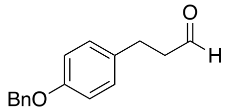

Aldehyde (SI-21) was prepared according to Method B. The following amounts of reagents were used: iodide **SI-20** (1.0 g, 3.2 mmol, 1.0 equiv), allyl alcohol (0.33 mL, 4.8 mmol, 1.5 equiv), $Pd(OAc)_2$ (7.0 mg, 32 µmol, 1.0 mol %), $BnEt_3NCl$ (0.73 g, 3.2 mmol, 1 equiv), $NaHCO_3$ (0.67 g, 8.0 mmol, 2.5 equiv), and DMF (13 mL). Purification by column chromatography (0–10% EtOAc/hexanes) afforded the title compound as a white solid (0.74 g, 3.1 mmol, 97% yield). **TLC R$_f$** = 0.3 (10% EtOAc/hexanes); **^{1}H NMR** (400 MHz, $CDCl_3$) δ 9.82 (t, *J* = 1.5 Hz, 1H), 7.54–7.34 (m, 5H), 7.24–7.11 (m, 2H), 7.02–6.95 (m, 2H), 5.09 (s, 2H), 2.95 (t, *J* = 7.5 Hz, 2H), 2.75 (t, *J* = 7.7 Hz, 2H); **^{13}C NMR** (101 MHz, $CDCl_3$) δ 201.5, 157.3, 132.7, 129.2 (2C), 128.5 (2C), 127.9, 127.4 (2C), 114.9 (2C), 70.0, 45.4, 27.2; **HRMS** (TOF MS ES+) *m/z*: $[M+Na]^+$ calcd for $C_{15}H_{16}O_2Na$, 263.1048; found 262.1051.

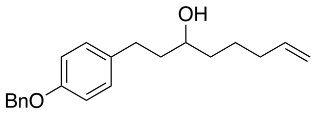

Alcohol (SI-22) was prepared according to Method D. The following amounts of reagents were used: aldehyde **SI-21** (0.87 g, 3.6 mmol, 1.0 equiv), Grignard reagent **SI-9** (5.0 mL, 1.5 mmol, 1.5 equiv, 1.1 M in THF), and THF (19 mL). The crude reaction mixture was filtered through a pad of silica gel eluting with 100% EtOAc to remove excess magnesium salts. The title compound was afforded as a white solid (1.1 g, 3.5 mmol, 97 % yield). **^{1}H NMR** (400 MHz, $CDCl_3$) δ 7.47 (d, *J* = 7.3 Hz, 2H), 7.42 (t, *J* = 7.1 Hz, 2H), 7.36 (t, *J* = 7.3 Hz, 1H), 7.16 (d, *J* = 8.3 Hz, 2H), 6.95 (d, *J* = 8.5 Hz, 2H), 5.86 (ddtd, *J* = 16.9, 10.2, 6.7, 1.5 Hz, 1H), 5.11–4.97 (m, 4H), 3.73–3.59 (m, 1H), 2.83–2.72 (m, 1H), 2.66 (ddd, *J* = 14.0, 9.4, 6.8 Hz, 1H), 2.18–2.03 (m, 2H), 1.85–1.66 (m, 3H), 1.64–1.40 (m, 3H); **^{13}C NMR** (101 MHz, $CDCl_3$) δ 157.1, 138.7, 137.3, 134.6, 129.4 (2C), 128.6 (2C), 127.9, 127.5 (2C), 122.7, 114.9 (2C), 71.2, 70.1, 39.3, 37.0, 33.8, 31.2, 24.9; **HRMS** (TOF MS ES+) *m/z*: $[M+Na]^+$ calcd for $C_{21}H_{26}O_2Na$, 333.1830; found 333.1828.

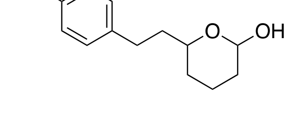

Lactol (SI-23) was prepared according to a modified version of Method E. The following amounts of reagents were used: alcohol **SI-22** (1.1 g, 3.5 mmol, 1.0 equiv), ozone (excess), CH_2Cl_2 (3.5 mL), MeOH (7.0 mL), PPh_3 (2.2 g, 2.0 g/1.0 mmol of alcohol) and $NaHCO_3$ (2.2 g, 2.0 g/1.0 g of alcohol). Purification by column chromatography (0–30% EtOAc/hexanes) afforded the title compound as a white solid (0.36 g, 1.2 mmol, 33% yield, 2:1 dr). For clarity, the ^{1}H and ^{13}C of the major and minor diastereomers have been tabulated individually.

TLC R$_f$ = 0.3 (50% EtOAc/hexanes); **HRMS** (TOF MS ES+) *m/z*: $[M+Na]^+$ calcd for $C_{20}H_{24}O_3Na$, 335.1623; found 335.1610.

Major Diastereomer: **^{1}H NMR** (400 MHz, $CDCl_3$) δ 7.49–7.32 (m, 5H), 7.13 (d, *J* = 8.4 Hz, 2H), 6.93 (d, *J* = 8.0 Hz, 2H), 5.40–5.32 (m, 1H) 5.06 (s, 2H), 4.73 (d, *J* = 9.5 Hz, 1H), 3.44 (dtd, *J* = 10.4, 5.3, 2.6 Hz, 1H), 2.83–2.54 (m, 2H), 1.98–1.82 (m, 2H), 1.82–1.58 (m, 2H), 1.58–1.44 (m, 2H), 1.42–1.18 (m, 2H); **^{13}C NMR** (101 MHz, $CDCl_3$) δ 157.1, 137.3, 134.6, 129.4 (2C), 128.6 (2C), 127.9, 127.5 (2C), 114.8 (2C), 96.6, 75.6, 70.1, 37.8, 33.0, 30.9, 30.5, 22.2.

Minor Diastereomer: **^{1}H NMR** (400 MHz, $CDCl_3$) δ 7.49–7.32 (m, 5H), 7.13 (d, *J* = 8.4 Hz, 2H), 6.93 (d, *J* = 8.0 Hz, 2H), 5.32–5.13 (m, 1H), 5.06 (s, 2H), 4.73 (d, *J* = 9.5 Hz, 1H), 4.08–3.92 (m, 1H), 2.83–2.54 (m, 2H), 1.98–1.82 (m, 2H), 1.82–1.58 (m, 2H), 1.58–1.44 (m, 2H), 1.42–1.18 (m, 2H); **^{13}C NMR** (101 MHz, $CDCl_3$) δ 157.1, 137.3, 134.7, 129.4 (2C), 128.6 (2C), 127.9, 127.5 (2C), 114.8 (2C), 91.9, 75.6, 68.2, 38.1, 33.0, 31.2, 30.0, 17.5.

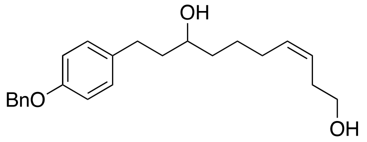

Diol (SI-24) was prepared according to Method G. The following amounts of reagents were used: lactol **SI-23** (56 mg, 0.18 mmol, 1.0 equiv), Wittig salt **SI-11** (73 mg, 0.18 mmol, 1.0 equiv), *n*-BuLi (0.30 mL, 0.58 mmol, 3.2 equiv, 2.5 M in hexanes), and THF (0.90 mL). Purification by column chromatography (0–50% yield) afforded the title compound as a white solid (27 mg, 80. µmol, 42% yield). For clarity, the ^{1}H and ^{13}C of the major and minor diastereomers have been tabulated individually.

TLC R$_f$ = 0.2 (50% EtOAc/hexanes, $KMnO_4$ stain); **HRMS** (TOF MS ES+) *m/z*: calcd for $C_{23}H_{30}O_3Na$, 377.2093; found 377.3075

Major Diastereomer: **^{1}H NMR** (600 MHz, $CDCl_3$) δ 7.49 (d, *J* = 7.0 Hz, 2H), 7.44 (dd, *J* = 8.5, 6.7 Hz, 2H), 7.38 (tt, *J* = 7.3, 1.4 Hz, 1H), 7.17 (d, *J* = 8.4 Hz, 2H), 6.96 (d, *J* = 8.6 Hz, 2H), 5.64–5.57 (m, 1H), 5.49–5.41 (m, 1H), 5.10 (s, 2H), 3.73–3.64 (m, 3H), 2.79 (ddd, *J* = 14.8, 9.5, 5.7 Hz, 1H), 2.67 (ddd, *J* = 13.9, 9.5, 6.8 Hz, 1H), 2.37 (qt, *J* = 6.8, 1.9 Hz, 1H), 2.21–2.05 (m, 2H), 1.87–1.70 (m, 2H), 1.62–1.40 (m, 7H); **^{13}C NMR** (151 MHz, $CDCl_3$) δ 157.2, 137.3, 134.6, 133.1, 129.4 (2C), 128.7 (2C), 128.0, 127.6 (2C), 125.7, 115.0 (2C), 71.3, 70.2, 62.4, 39.4, 37.2, 31.3, 30.9, 27.4, 25.8.

Minor Diastereomer: **^{1}H NMR** (600 MHz, $CDCl_3$) δ 7.49 (d, *J* = 7.0 Hz, 2H), 7.44 (dd, *J* = 8.5, 6.7 Hz, 2H), 7.38 (tt, *J* = 7.3, 1.4 Hz, 1H), 7.17 (d, *J* = 8.4 Hz, 2H), 6.96 (d, *J* = 8.6 Hz, 2H), 5.64–5.57 (m, 1H), 5.49–5.41 (m, 1H), 5.10 (s, 2H), 3.73–3.64 (m, 3H), 2.79 (ddd, *J* = 14.8, 9.5, 5.7 Hz, 1H), 2.67 (ddd, *J* = 13.9, 9.5, 6.8 Hz, 1H), 2.32 (qd, *J* = 6.3, 1.2 Hz, 1H), 2.21–2.05 (m, 2H), 1.87–1.70 (m, 2H), 1.62–1.40 (m, 7H); **^{13}C NMR** (151 MHz, $CDCl_3$) δ 157.2, 137.3, 134.6, 133.9, 129.4 (2C), 128.7 (2C), 128.0, 127.6 (2C), 126.4, 115.0 (2C), 71.3, 70.2, 62.2, 39.4, 37.2, 36.1, 32.7, 27.4, 25.5.

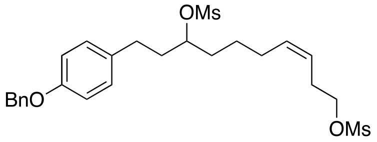

Dimesylate (SI-2) was prepared according to Method H. The following amounts of reagents were used: diol **SI-24** (27 mg, 80. µmol, 1.0 equiv), MsCl (20. µL, 0.20 mmol, 2.5 equiv), Et_3N (30. µL, 0.20 mmol, 2.5 equiv), and CH_2Cl_2 (0.50 mL). The crude reaction mixture was filtered through a pad a silica gel eluting with 100% CH_2Cl_2 and the title compound was isolated as a pale yellow oil (35 mg, 68 µmol, 85% yield, 2:1 dr). For clarity, the ^{1}H and ^{13}C of the major and minor diastereomers have been tabulated individually.

TLC R$_f$ = 0.3 (30% EtOAc/hexanes); **HRMS** (TOF MS ES+) *m/z*: [M+Na] calcd for $C_{25}H_{34}O_7S_2Na$, 533.1644; found 533.1658.

Major Diastereomer: ^{1}H NMR (500 MHz, $CDCl_3$) δ 7.49 (d, *J* = 7.3 Hz, 2H), 7.44 (t, *J* = 7.5 Hz, 2H), 7.38 (dd, *J* = 8.5, 5.9 Hz, 1H), 7.17 (d, *J* = 8.4 Hz, 2H), 6.97 (d, *J* = 8.5 Hz, 2H), 5.60 (q, *J* = 8.5, 7.4 Hz, 1H), 5.51 – 5.40 (m, 1H), 5.10 (d, *J* = 3.3 Hz, 2H), 4.81 (quint, *J* = 6.0 Hz, 1H), 4.30–4.24 (m, 2H), 3.72 (d, *J* = 1.0 Hz, 2H), 3.05 (s, 6H), 2.73 (qdd, *J* = 14.3, 9.6, 6.3 Hz, 2H), 2.55 (q, *J* = 7.0 Hz, 2H), 2.22–1.94 (m, 2H), 1.90–1.71 (m, 2H), 1.61–1.45 (m, 2H); **^{13}C NMR** (126 MHz, $CDCl_3$) δ 157.3, 137.2, 133.2, 133.1, 129.4 (2C), 128.7 (2C), 128.0, 127.6 (2C), 123.9, 115.0 (2C), 83.0, 70.1, 69.3, 38.8, 37.6, 36.4, 34.1, 31.7, 27.5, 27.0, 24.8.

Minor Diastereomer: ^{1}H NMR (500 MHz, $CDCl_3$) δ 7.49 (d, *J* = 7.3 Hz, 2H), 7.44 (t, *J* = 7.5 Hz, 2H), 7.38 (dd, *J* = 8.5, 5.9 Hz, 1H), 7.17 (d, *J* = 8.4 Hz, 2H), 6.97 (d, *J* = 8.5 Hz, 2H), 5.60 (q, *J* = 8.5, 7.4 Hz, 1H), 5.51–5.40 (m, 1H), 5.10 (d, *J* = 3.3 Hz, 2H), 4.81 (p, *J* = 6.0 Hz, 1H), 4.30–4.24 (m, 2H), 3.19 (d, *J* = 1.0 Hz, 1H), 3.05 (s, 6H), 2.73 (qdd, *J* = 14.3, 9.6, 6.3 Hz, 2H), 2.50 (q, *J* = 7.0 Hz, 2H), 2.22–1.94 (m, 2H), 1.90–1.71 (m, 2H), 1.61–1.45 (m, 2H); **^{13}C NMR** (126 MHz, $CDCl_3$) δ 157.3, 133.8, 133.2, 133.1, 129.4 (2C), 128.7 (2C), 128.0, 127.6 (2C), 124.7, 115.0 (2C), 83.1, 70.1, 69.6, 38.8, 34.0, 32.5, 32.2, 31.7, 27.5, 27.0, 24.5.

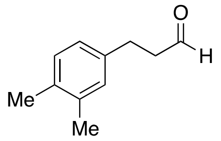

Aldehyde (SI-25) was prepared according to Method B. The following amounts of reagents were used: 4-iodo-*o*-xylene (0.85 mL, 6.0 mmol, 1.0 equiv), allyl alcohol (0.61 mL, 9.0 mmol, 1.5 equiv), $Pd(OAc)_2$ (27 mg, 0.12 mmol, 2.0 mol %), $BnEt_3NCl$ (1.4 g, 6.0 mmol, 1.0 equiv), $NaHCO_3$ (1.3 g, 15 mmol, 2.5 equiv), and DMF (24 mL). Purification by column chromatography (0–5% EtOAc/hexanes) afforded the title compound as a yellow oil (0.56 g, 3.4 mmol, 57% yield). For clarity, the ^{1}H and ^{13}C data are tabulated individually. **TLC R$_f$** = 0.7 (50% EtOAc/hexanes); **^{1}H NMR** (500 MHz, $CDCl_3$) δ 9.81 (t, *J* = 1.5 Hz, 1H), 7.05 (d, *J* = 7.6 Hz, 1H), 6.96 (s, 1H), 6.92 (d, *J* = 7.6 Hz, 1H), 2.89 (t, *J* = 7.5 Hz, 2H), 2.75 (t, *J* = 8.0 Hz, 2H), 2.23 (s, 3H), 2.22 (s, 3H); **^{13}C NMR** (126 MHz, $CDCl_3$) δ 201.9, 137.7, 136.8, 134.5, 129.9, 129.7, 125.6, 45.5, 27.7, 19.8, 19.3; **HRMS** (TOF MS CI+) *m/z*: $[M]^+$ calcd for $C_{11}H_{14}O$, 162.1045; found 162.1044.

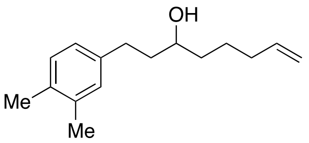

Alcohol (SI-26) was prepared according to Method D. The following amounts of reagents were used: aldehyde **SI-25** (0.56 g, 3.4 mmol, 1.0 equiv), Grignard reagent **SI-9** (3.9 mL, 5.1 mmol, 1.5 equiv, 1.3 M in Et_2O), Et_2O (17 mL). Purification by column chromatography (0–20% EtOAc/hexanes) afforded the title compound as a yellow oil (0.43 g, 1.8 mmol, 54% yield). **TLC** $\mathbf{R_f}$ = 0.7 (20% EtOAc/hexanes); ^{1}**H NMR** (500 MHz, $CDCl_3$) δ 7.04 (d, *J* = 7.6 Hz, 1H), 6.97 (s, 1H), 6.93 (d, *J* = 7.6 Hz, 1H), 5.80 (ddt, *J* = 17.0, 10.3, 6.6 Hz, 1H), 4.98 (ddt, *J* = 17.1, 2.0, 1.5 Hz, 1H), 4.95 (ddt, *J* = 10.2, 2.1, 1.1 Hz, 1H), 3.63 (br s, 1H), 2.72 (ddd, *J* = 13.8, 9.3, 6.0 Hz, 1H), 2.60 (ddd, *J* = 13.7, 8.9, 6.1 Hz, 1H), 2.23 (s, 3H), 2.22 (s, 3H), 2.07 (aq, *J* = 5.6 Hz, 2H), 1.81–1.68 (m, 2H), 1.56–1.40 (m, 4H), 1.32 (d, *J* = 4.3 Hz, 1H); ^{13}**C NMR** (126 MHz, $CDCl_3$) δ 139.6, 138.7, 136.5, 134.0, 129.8, 129.7, 125.8, 114.7, 71.4, 39.3, 37.0, 33.7, 31.6, 24.9, 19.8, 19.3; **HRMS** (TOF MS CI+) *m/z*: $[M+Na]^+$ calcd for $C_{16}H_{24}ONa$, 255.1725; found 255.1723.

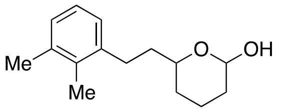

Lactol (SI-27) was prepared according to Method E. The following amounts of reagents were used: alkene **SI-26** (0.42 g, 1.3 mmol, 1.0 equiv), ozone (excess), $NaHCO_3$ (190 mg, 2.2 mmol, 1.7 equiv), PPh_3 (0.58 g. 2.2 mmol, 1.7 mmol), CH_2Cl_2 (1.3 mL). Purification by column chromatography (0–20% EtOAc/hexanes) afforded the title compound as a colorless oil (240 mg, 1.0 mmol, 80% yield, 1.5:1 dr). For clarity, the ^{1}H and ^{13}C data are tabulated individually.

TLC $\mathbf{R_f}$ = 0.6 (30% EtOAc/hexanes); **HRMS** (TOF MS CI+) *m/z*: $[M+Na]^+$ calcd for $C_{15}H_{22}O_2Na$, 257.1518; found 257.1523.

Major Diastereomer ^{1}**H NMR** (500 MHz, $CDCl_3$) δ 7.04 (d, *J* = 7.6 Hz, 1H), 6.97 (s, 1H), 6.93 (d, *J* = 7.5 Hz, 1H), 4.70 (t, *J* = 7.7 Hz, 1H), 3.44–3.39 (m, 1H), 2.84 (d, *J* = 6.1 Hz, 1H), 2.76–2.52 (m, 2H), 2.23 (s, 3H), 1.92–1.80 (m, 3H), 1.78–1.61 (m, 3H), 1.54–1.48 (m, 2H), 1.36–1.20 (m, 3H); ^{13}**C NMR** (126 MHz, $CDCl_3$) δ 139.6, 136.5, 133.9, 129.9, 129.6, 125.8, 96.5, 75.5, 37.8, 33.0, 31.3, 30.5, 29.8, 22.1, 19.3.

Minor Diastereomer ^{1}**H NMR** (500 MHz, $CDCl_3$) δ 7.04 (d, *J* = 7.6 Hz, 1H), 6.97 (s, 1H), 6.93 (d, *J* = 7.5 Hz, 1H), 5.33 (s, 1H), 3.96–3.91 (m, 1H), 2.76–2.52 (m, 2H), 2.33 (brs, 1H), 2.22 (s, 3H), 1.92–1.80 (m, 3H), 1.78–1.61 (m, 3H), 1.54–1.48 (m, 2H), 1.36–1.20 (m, 3H); ^{13}**C NMR** (126 MHz, $CDCl_3$) δ 139.8, 136.5, 133.9, 129.8, 129.6, 125.8, 92.0, 68.1, 38.1, 33.0, 31.3, 31.2, 29.8, 22.1, 19.3.

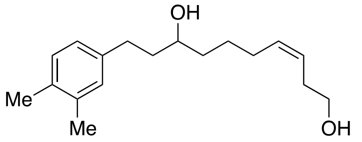

Diol (SI-28) was prepared according to Method G. The following amounts of reagents were used: lactol **SI-27** (240 mg, 1.0 mmol. 1.0 equiv), Wittig salt **SI-11** (0.41 g, 1.01 mmol, 1.01 equiv), *n*-BuLi (1.3 mL, 3.2 mmol, 3.2 equiv, 2.5 M in hexanes) and THF (5.0 mL). Purification by column chromatography (0–50% EtOAc/hexanes) afforded the title compound as a yellow oil (0.16 g, 0.59 mmol, 59% yield, 1.5:1 dr). Diastereomers were separated on Ag impregnated silica[17] by column chromatography to afford the title compound as a brown oil (95 mg, 0.34 mmol, >20:1 dr *Z:E*). **TLC** $\mathbf{R_f}$ = 0.4 (50% EtOAc/hexanes); ^{1}**H NMR** (500 MHz, $CDCl_3$) δ 7.04 (d, *J* = 7.6 Hz, 1H), 6.98 (s, 1H), 6.93 (d, *J* = 7.5 Hz, 1H), 5.55 (dt, *J* = 10.8, 8.0 Hz, 1H), 5.38 (dt, *J* = 10.0, 8.3 Hz, 1H), 3.64 (t, *J* = 6.4 Hz, 3H), 2.75–2.69 (m, 1H), 2.63–2.57 (m, 1H), 2.30 (dt, *J* = 6.7, 6.2 Hz, 2H), 2.24 (s, 3H), 2.22 (s, 3H), 2.09 (quint, 6.6 Hz, 2H), 1.80–1.67 (m, 2H), 1.54–1.37 (m, 6H); ^{13}**C NMR** (125 MHz, $CDCl_3$) δ 139.5, 136.6, 134.0, 133.0, 129.8, 129.7,

125.8, 125.6, 71.3, 62.3, 39.3, 37.0, 31.6, 30.8, 27.3, 25.7, 19.8, 19.3. **HRMS** (TOF MS CI+) *m/z*: $[M+Na]^+$ calcd for $C_{18}H_{28}O_2Na$, 299.1987; found 299.1995.

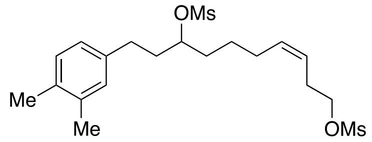

Dimesylate (SI-3) was prepared according to Method H. The following amounts of reagents were used: diol **SI-28** (89 mg, 0.32 mmol, 1.0 equiv), MsCl (70. µL, 0.96 mmol, 3.0 equiv), DMAP (8.0 mg, 60. µmol, 0.20 equiv), Et_3N (0.13 mL, 0.96 mmol, 3.0 equiv), and CH_2Cl_2 (1.6 mL). Purification by column chromatography (0–50% EtOAc/hexanes) afforded the title compound as a yellow oil (95 mg, 0.22 mmol, 69% yield, >20:1 dr). **TLC R$_f$** = 0.7 (50% EtOAc/hexanes); **^{1}H NMR** (400 MHz, $CDCl_3$) δ 7.05 (d, *J* = 7.7 Hz, 1H), 6.96 (s, 1H), 6.92 (d, *J* = 7.6 Hz, 1H), 5.53 (dtt, *J* = 10.8, 7.3, 1.4 Hz, 1H), 5.38 (dtt, *J* = 10.8, 7.3, 1.5 Hz, 1H), 4.76 (quin, *J* = 6.1 Hz, 1H), 4.20 (t, *J* = 6.8 Hz, 2H), 3.000 (s, 3H), 2.996 (s, 3H), 2.73–2.58 (m, 2H), 2.49 (dt, *J* = 7.3, 7.0 Hz, 2H), 2.24 (s, 3H), 2.23 (s, 3H), 2.09 (quin, *J* = 6.3 Hz, 2H), 2.04–1.95 (m, 2H), 1.79–1.72 (m, 2H), 1.54–1.46 (m, 2H); **^{13}C NMR** (125 MHz, $CDCl_3$) δ 138.3, 136.8, 134.4, 133.2, 129.9, 129.8, 125.7, 123.9, 83.2, 69.3, 38.9, 37.6, 36.4, 34.2, 30.9, 27.5, 27.1, 24.8, 19.9, 19.4; **HRMS** (TOF MS CI+) *m/z*: $[M+Na]^+$ calcd for $C_{20}H_{32}O_6S_2Na$, 455.1538; found 455.1523.

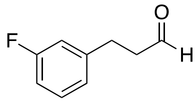

Aldehyde (SI-29) was prepared according to Method B. The following amounts of reagents were used: 3-fluoroiodobenzene (0.70 mL, 6.0 mmol, 1.0 equiv), allyl alcohol (0.61 mL, 9.0 mmol, 1.5 equiv), $Pd(OAc)_2$ (27 mg, 0.12 mmol, 2.0 mol %), $BnEt_3NCl$ (1.4 g, 6.0 mmol, 1.0 equiv), $NaHCO_3$ (1.3 g, 15 mmol, 2.5 equiv), and DMF (24 mL). Purification by column chromatography (0–10% EtOAc/hexanes) afforded the title compound as a yellow oil (470 mg, 3.1 mmol, 51% yield). Analytical data is consistent with literature values.[18] **^{1}H NMR** (500 MHz, $CDCl_3$) δ 9.82 (d, *J* = 1.1 Hz, 1H), 7.25 (dd, *J* = 14.6, 7.4 Hz, 1H), 6.97 (d, *J* = 7.6 Hz, 1H), 6.91–6.88 (m, 2H), 2.96 (t, *J* = 7.5 Hz, 2H), 2.78 (t, *J* = 7.5 Hz, 2H).

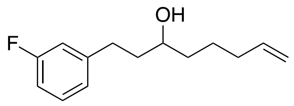

Alcohol (SI-30) was prepared according to Method D. The following amounts of reagents were used: aldehyde **SI-27** (0.47 g, 3.1 mmol, 1.0 equiv), Grignard reagent **SI-9** (4.5 mL, 5.8 mmol, 0.97 equiv, 1.3 M in THF), and THF (16 mL). The crude residue was taken on without further purification.

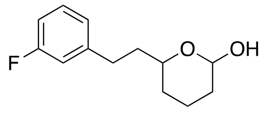

Lactol (SI-31) was prepared according to Method E. The following amounts of reagents were used: alkene **SI-30** (0.69 g, 3.1 mmol, 1.0 equiv), $NaHCO_3$ (0.44 g, 5.3 mmol, 1.7 equiv), PPh_3 (1.4 g, 5.3 mmol, 1.7 equiv), $NaHCO_3$ (0.44 g, 5.3 mmol, 1.7 equiv), and CH_2Cl_2 (3.1 mL). Purification by column chromatography (0–30% EtOAc/hexanes) afforded the title compound as a white solid (0.35 g, 1.6 mmol, 50% yield over two steps, 1.6:1 dr) containing CH_2Cl_2 (12 mg, 0.15 mmol, 5%). For clarity, the ^{1}H and ^{13}C data are tabulated individually.

TLC R$_f$ = 0.7 (50% EtOAc/hexanes); **HRMS** (TOF MS CI+) *m/z*: [M+Na]$^+$ calcd for $C_{13}H_{17}FO_2Na$, 247.1110; found 247.1098.

Major Diastereomer ^{1}H NMR (400 MHz, $CDCl_3$) δ 7.23 (dd, *J* = 14.3, 7.8 Hz, 1H), 6.96 (d, *J* = 7.7 Hz, 1H), 6.90–6.85 (m, 2H), 4.70 (ddd, *J* = 9.5, 6.3, 1.5 Hz, 1H), 3.43–3.37 (m, 1H), 2.85 (d, *J* = 6.4 Hz, 1H), 2.82–2.62 (m, 1H), 1.94–1.83 (m, 2H), 1.80–1.56 (m, 5H), 1.37–1.20 (m, 2H); **^{13}C NMR** (151 MHz, $CDCl_3$) δ 163.1 (d, *J* = 244.1 Hz), 144.9 (d, *J* = 7.2 Hz), 129.8 (d, *J* = 8.3 Hz), 124.2 (d, *J* = 2.7 Hz), 115.4 (d, *J* = 20.7 Hz), 112.8 (d, *J* = 21.0 Hz), 96.6, 75.4, 37.4, 33.0, 31.6, 30.6, 22.2; **^{19}F NMR** (565 MHz, $CDCl_3$) δ -113.8–-113.9 (m, 1F)

Minor Diastereomer ^{1}H NMR (400 MHz, $CDCl_3$) δ 7.23 (dd, *J* = 14.3, 7.8 Hz, 1H), 6.96 (d, *J* = 7.7 Hz, 1H), 6.90–6.85 (m, 2H), (5.33 (br s, 1H), 3.96–3.90 (m, 1H), 2.85 (d, *J* = 6.4 Hz, 1H), 2.82–2.62 (m, 1H), 1.94–1.83 (m, 2H), 1.80–1.56 (m, 5H), 1.37–1.20 (m, 2H); **^{13}C NMR** (151 MHz, $CDCl_3$) δ 163.1 (d, *J* = 244.1 Hz), 145.1 (d, *J* = 7.2 Hz), 129.8 (d, *J* = 8.5 Hz), 124.2 (d, *J* = 2.6 Hz), 115.4 (d, *J* = 20.7 Hz), 112.8 (d, *J* = 21.0 Hz), 92.1, 68.0, 37.7, 31.6, 31.4, 29.9, 17.5; **^{19}F NMR** (565 MHz, $CDCl_3$) δ -113.8–-113.9 (m, 1F)

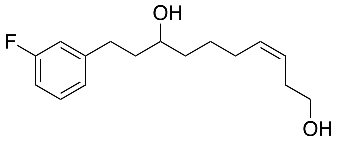

Diol (SI-32) was prepared according to Method G. The following amounts of reagents were used: lactol **SI-31** (0.35 g, 1.6 mmol, 1.0 equiv), Wittig salt **SI-11** (0.63 g, 1.6 mmol, 1.0 equiv), *n*-BuLi (2.0 mL, 5.0 mmol, 3.2 equiv), and THF (8.0 mL). Purification by column chromatography (0–50% EtOAc/hexanes) afforded the title compound as a yellow oil (200 mg, 0.75 mmol, 48% yield, 1:1 dr). **TLC R$_f$** = 0.4 (50% EtOAc/hexanes); **^{1}H NMR** (500 MHz, $CDCl_3$) δ 7.23 (aq, *J* = 7.3 Hz, 2H, both diastereomers), 6.97 (d, *J* = 7.5 Hz, 2H, both diastereomers), 6.91–6.85 (m, 4H, both diastereomers), 5.57–5.52 (m, 2H, both diastereomers), 5.42–5.37 (m, 2H, both diastereomers), 3.65–3.62 (m, 6H, both diastereomers), 2.82–2.77 (m, 2H, both diastereomers), 2.69–2.64 (m, 2H, both diastereomers), 2.31 (dt (*J* = 7.2, 6.2 Hz, 2H, one diastereomer), 2.26 (dt, *J* = 6.5, 6.1 Hz, 2H, other diastereomer), 2.12–2.02 (m, 4H, both diastereomers), 1.80–1.68 (m, 4H, both diastereomers), 1.61 (brs, 2H, both diastereomers), 1.53–1.38 (m, 10H, both diastereomers); **^{13}C NMR** (125 MHz, $CDCl_3$) δ 163.0 (d, *J* = 245.3 Hz, 2C, both diastereomers), 144.82 (d, *J* = 7.0 Hz, one diastereomer), 144.79 (d, *J* = 7.1 Hz, other diastereomer), 133.7 (one diastereomer), 132.9 (other diastereomer), 129.8 (d, *J* = 8.4 Hz, 2C, both diastereomers), 126.4 (one diastereomer), 125.7 (other diastereomer), 124.1 (d, *J* = 2.6 Hz, 2C, both diastereomers), 115.3 (d, *J* = 20.8 Hz, one diastereomer), 112.7 (d, *J* = 21.0 Hz, other diastereomer), 71.03 (one diastereomer), 71.02 (other diastereomer), 63.3 (one diastereomer), 62.1 (other diastereomer), 38.81 (one diastereomer), 38.79 (other diastereomer), 37.11 (one diastereomer), 37.09 (other diastereomer), 36.0 (2C, both diastereomers), 32.6 (one diastereomer), 31.83 (one diastereomer), 31.82 (other diastereomer), 30.82 (other diastereomer), 27.2 (2C, both diastereomers), 25.7 (one diastereomers), 25.4 (other diastereomers); **^{19}F NMR** (565 MHz, $CDCl_3$) δ -113.7 (apparq, *J* = 8.2 Hz, 2F, both diastereomers); **HRMS** (TOF MS CI+) *m/z*: [M+Na]$^+$ calcd for $C_{16}H_{23}FO_2Na$, 289.1580; found 289.1569.

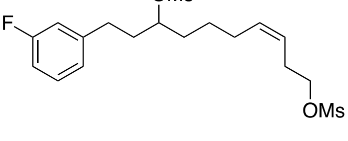

Dimesylate (SI-4) was prepared according to Method H. The following amounts of reagents were used: diol **SI-32** (197 mg, 0.74 mmol, 1.0 equiv), MsCl (0.17 mL, 2.2 mmol, 3.0 equiv), DMAP (18 mg, 0.15 mmol, 0.20 equiv), Et_3N (0.31 mL, 2.0 mmol, 3.0 equiv), and CH_2Cl_2 (3.7 mL). Purification by column chromatography (0–30% EtOAc/hexanes) afforded the title compound as a yellow oil (0.24 g, 0.56 mmol,

75% yield, 1:1 dr). **TLC R_f** = 0.3 (50% EtOAc/hexanes); **^{1}H NMR** (600 MHz, $CDCl_3$) δ 7.27–7.23 (m, 2H, both diastereomers), 6.98 (d, *J* = 7.2 Hz, 2H, both diastereomers), 6.91–6.88 (m, 4H, both diastereomers), 5.56–5.52 (m, 2H), 5.42–5.38 (m, 2H), 4.78–4.75 (m, 2H, both diastereomers), 4.21 (aq, *J* = 6.2 Hz, 4H, both diastereomers), 3.02 (s, 3H, one diastereomer), 3.01 (s, 3H, other diastereomer), 3.001 (s, 3H, one diastereomer), 2.999 (s, 3H, other diastereomer), 2.79–2.68 (m, 4H, both diastereomers), 2.50 (dt *J* = 7.2, 6.2 Hz, 2H, one diastereomer), 2.44 (dt *J* = 6.9, 6.2 Hz, 2H, other diastereomer), 2.13–1.95 (m, 8H, both diastereomers), 1.79–1.70 (m, 4H, both diastereomers), 1.53–1.44 (m, 4H, both diastereomers); **^{13}C NMR** (125 MHz, $CDCl_3$) δ 163.0 (d, *J* = 242.4 Hz, 2C, both diastereomers), 143.6 (d, *J* = 2.1 Hz, one diastereomer), 143.5 (d, *J* = 2.0 Hz, other diastereomer), 133.8 (one diastereomer) 133.1 (other diastereomer), 130.1 (d, *J* = 8.4 Hz, 2C, both diastereomers), 124.8 (one diastereomer), 124.2 (d, *J* = 2.4 Hz, 2C, both diastereomers), 124.0 (other diastereomer), 115.34 (d, *J* = 21.3 Hz, one diastereomer), 115.33 (d, *J* = 21.2 Hz, other diastereomer), 113.24 (d, *J* = 21.2 Hz, one diastereomer), 113.23 (d, *J* = 21.2 Hz, other diastereomer), 82.7 (one diastereomer), 82.6 (other diastereomer), 69.5 (one diastereomer), 69.3 (other diastereomer), 38.9 (2C, both diastereomers), 37.63 (one diastereomer), 37.64 (other diastereomer), 36.0 (2C, both diastereomers), 34.2 (2C, both diastereomers), 34.0 (2C, both diastereomers), 32.5 (one diastereomer), 32.2 (other diastereomer), 31.1 (2C, both diastereomers), 27.6 (one diastereomer), 27.0 (other diastereomer); **^{19}F NMR** (565 MHz, $CDCl_3$) δ -113.3 to -113.4 (m, 2F, both diastereomers); **HRMS** (TOF MS ES+) *m/z*: $[M+Na]^+$ calcd for $C_{18}H_{27}FO_6S_2Na$, 445.1131; found 445.1118.

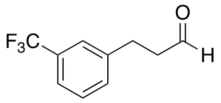

Aldehyde (SI-33) was prepared according to Method B. The following amounts of reagents were used: 1-iodo-3-(trifluoromethyl)benzene (0.72 mL, 5.0 mmol, 1.0 equiv), allyl alcohol (0.51 mL, 7.5 mmol, 1.5 equiv), $Pd(OAc)_2$ (11 mg, 5.0 µmol, 1.0 mol %), $BnEt_3NCl$ (1.1 g, 5.0 mmol, 1.0 equiv), $NaHCO_3$ (1.1 g, 13 mmol, 2.5 equiv), and DMF (20. mL). **TLC R_f** = 0.2 (10% EtOAc/hexanes, CAM stain); **^{1}H NMR** (500 MHz, $CDCl_3$) δ 9.82 (s, 1H), 7.49–7.44 (m, 2H), 7.43–7.37 (m, 2H), 3.01 (t, *J* = 7.6 Hz, 2H), 2.82 (t, *J* = 7.6 Hz, 2H); **^{13}C NMR** (126 MHz, $CDCl_3$) δ 200.8, 141.5, 131.9 (q, *J* = 1.4 Hz), 131.0 (q, *J* = 32.4 Hz), 129.2, 125.2 (q, *J* = 3.7 Hz), 124.3 (q, *J* = 272.4 Hz), 123.3 (q, *J* = 3.7 Hz), 45.1, 27.9; **^{19}F NMR** (565 MHz, $CDCl_3$) δ –62.7 (3F); **HRMS** (TOF MS ES+) *m/z*: $[M+Na]^+$ calcd for $C_{10}H_9F_3Na$, 202.0605; found 202.0615.

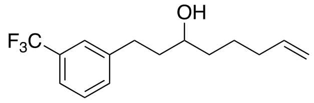

Alcohol (SI-34) was prepared according to Method D. The following amounts of reagents were used: aldehyde **SI-33** (0.43 g, 2.1 mmol, 1.0 equiv), Grignard reagent **SI-9** (6.0 mL, 3.2 mmol, 1.5 equiv, 0.50 M in Et_2O), and Et_2O (11 mL). Purification by column chromatography (0–20% EtOAc/hexanes) afforded the title compound as a yellow oil (0.44 g, 1.6 mmol, 75% yield). **TLC R_f** = 0.6 (20% EtOAc/hexanes, $KMnO_4$ stain); **^{1}H NMR** (600 MHz, $CDCl_3$) δ 7.47–7.42 (m, 2H), 7.38 (d, *J* = 4.8 Hz, 2H), 5.79 (ddt, *J* = 16.9, 10.2, 6.7 Hz, 1H), 5.04–4.92 (m, 2H), 3.62 (tt, *J* = 7.9, 4.2 Hz, 1H), 2.86 (ddd, *J* = 15.0, 10.1, 5.5 Hz, 1H), 2.72 (ddd, *J* = 13.8, 10.0, 6.6 Hz, 1H), 2.19–2.00 (m, 2H), 1.85–1.66 (m, 3H), 1.62–1.37 (m, 3H); **^{13}C NMR** (151 MHz, $CDCl_3$) δ 143.2, 138.6, 132.0 (q, *J* = 1.2, 0.7 Hz), 130.8 (q, *J* = 31.8 Hz), 128.9, 124.4 (q, *J* = 272.1 Hz), 125.17 (q, *J* = 3.8 Hz), 122.79 (q, *J* = 3.8 Hz), 114.8, 71.1, 40.0, 37.1, 33.8, 32.0, 25.0; **^{19}F NMR** (565 MHz, $CDCl_3$) δ –62.6 (3F); **HRMS** (TOF MS CI+) *m/z*: $[M+NH_4]^+$ calcd for $C_{15}H_{19}F_3ONH_4$ 290.1732, found 290.1728.

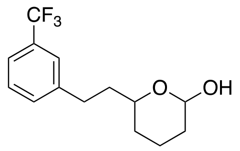

Lactol (SI-35) was prepared according to Method E. The following amount of reagents were used: alkene **SI-34** (0.72 g, 2.6 mmol, 1.0 equiv), ozone (excess), CH_2Cl_2 (20 mL), PPh_3 (1.7 g, 2.0 g/1.0 g of alcohol), $NaHCO_3$ (1.7 g. 2.0 g/1.0 g of alcohol). Purification by column chromatography (0–30% EtOAc/hexanes) to afford the title compound as a white solid (0.47 g, 1.7 mmol, 65% yield, 2:1 dr). For clarity, the ^{1}H and ^{13}C of the major and minor diastereomers have been tabulated individually.

TLC R_f = 0.2 (20% EtOAc/hexanes, CAM Stain); **HRMS** (TOF MS ES+) *m/z*: $[M+Na]^+$ calcd for $C_{14}H_{17}F_3O_2Na$, 297.1078; found 297.1077.

Major Diastereomer: ^{1}H NMR (600 MHz, $CDCl_3$) δ 7.46–7.41 (m, 2H), 7.40–7.33 (m, 2H, 4.70 (ddd, *J* = 9.0, 6.4, 2.1 Hz, 1H), 3.56 (br s, 1H), 3.41 (dddd, *J* = 10.5, 8.1, 4.4, 2.0 Hz, 1H), 2.91–2.63 (m, 2H), 1.98–1.42 (m, 6H), 1.41–1.15 (m, 2H); **^{13}C NMR** (151 MHz, $CDCl_3$) δ 143.1, 132.0 (q, *J* = 1.1 Hz), 130.72 (q, *J* = 32.1 Hz), 128.9, 125.3 (q, *J* = 3.8 Hz), 124.4 (q, *J* = 272.6 Hz), 122.78 (q, *J* = 3.9 Hz), 96.7, 75.5, 37.5, 33.0, 31.6, 30.5, 22.1; **^{19}F NMR** (565 MHz, $CDCl_3$) δ –62.6 (3F).

Minor Diastereomer: ^{1}H NMR (600 MHz, $CDCl_3$) δ 7.46–7.41 (m, 2H), 7.40–7.33 (m, 2H), 5.34 (s, 1H), 4.02–3.90 (m, 1H), 3.56 (br s, 1H), 2.91–2.63 (m, 2H), 1.98–1.42 (m, 6H), 1.41–1.15 (m, 2H); **^{13}C NMR** (151 MHz, $CDCl_3$) δ 143.3, 131.9 (q, *J* = 1.1 Hz), 130.69 (q, *J* = 31.5 Hz), 128.8, 125.3 (q, *J* = 3.3

Hz), 124.4 (q, J = 272.6 Hz), 122.75 (q, J = 3.9 Hz), 92.0, 68.0, 37.8, 31.2 30.0, 22.1, 17.5; **^{19}F NMR** (565 MHz, $CDCl_3$) δ –62.6 (3F).

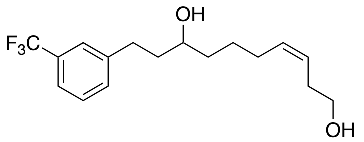

Diol (SI-36) was prepared according to Method G. The following amounts of reagents were used: lactol **SI-35** (0.47 g, 1.7 mmol, 1.0 equiv), Wittig salt **SI-11** (0.69 g, 1.7 mmol, 1.0 equiv), *n*-BuLi (2.2 mL, 5.4 mmol, 3.2 equiv, 2.5 M in hexanes), and THF (8.5 mL). Purification by column chromatography (0–5% MeOH/CH_2Cl_2) afforded a mixture of the title compound and triphenylphosphine oxide. This mixture was carried onto the next step without further purification.

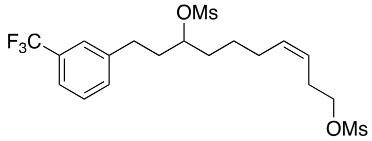

Dimesylate (SI-5) was prepared according to Method H. The following amounts of reagents were used: diol **SI-36** (0.34 g, 1.1 mmol, 1.0 equiv), methanesulfonyl chloride (0.21 mL, 2.7 mmol, 2.5 equiv), DMAP (26 mg, 0.22 mmol, 20. mol %), Et_3N (0.38 mL, 2.7 mmol, 2.5 equiv), and CH_2Cl_2 (5.4 mL). Purification by column chromatography (0–30% EtOAc/hexanes) afforded the title compound as a pale yellow oil (0.40 g, 0.94 mmol, 78% yield, 2:1 dr). For clarity, the ^{1}H and ^{13}C of the major and minor diastereomers have been tabulated individually.

TLC R_f = 0.6 (50% EtOAc/hexanes, CAM Stain); **HRMS** (TOF MS ES+) *m/z*: $[M+Na]^+$ calcd for $C_{19}H_{27}F_3O_6S_2Na$, 495.1099; found 495.1080.

Major Diastereomer: ^{1}H NMR (600 MHz, $CDCl_3$) δ 7.47 (dd, J = 6.6, 1.7 Hz, 2H), 7.42–7.39 (m, 2H), 5.59–5.51 (m, 1H), 5.44–5.36 (m, 1H), 5.30 (s, 1H), 4.82–4.74 (m, 1H), 4.28–4.16 (m, 2H), 3.03 (s, 3H), 3.00 (s, 3H), 2.84 (ddd, J = 14.0, 10.2, 6.0 Hz, 1H), 2.77 (ddd, J = 14.2, 10.1, 6.4 Hz, 1H), 2.50 (q, J = 7.0 Hz, 1H), 2.16–1.97 (m, 4H), 1.85–1.69 (m, 2H), 1.49 (dddd, J = 20.3, 17.1, 13.5, 6.7 Hz, 2H); **^{13}C NMR** (151 MHz, $CDCl_3$) δ 141.9, 133.6, 132.9, 130.7 (q, J = 31.5 Hz), 129.0, 125.0 (q, J = 3.9 Hz), 124.8, 124.2 (q, J = 272.4), 123.1 (q, J = 3.9 Hz), 82.5, 69.3, 60.4, 38.7, 35.9 (2C), 34.0, 31.1, 26.9, 24.7; **^{19}F NMR** (565 MHz, $CDCl_3$) δ –62.5 (3F).

Minor Diastereomer: ^{1}H NMR (600 MHz, $CDCl_3$) δ 7.47 (dd, J = 6.6, 1.7 Hz, 2H), 7.42–7.39 (m, 2H), 5.59–5.51 (m, 1H), 5.44–5.36 (m, 1H), 5.30 (s, 1H), 4.82–4.74 (m, 1H), 4.28–4.16 (m, 2H), 3.03 (s, 3H), 3.00 (s, 3H), 2.84 (ddd, J = 14.0, 10.2, 6.0 Hz, 1H), 2.77 (ddd, J = 14.2, 10.1, 6.4 Hz, 1H), 2.44 (q, J = 6.7 Hz, 1H), 2.16–1.97 (m, 4H), 1.85–1.69 (m, 2H), 1.49 (dddd, J = 20.3, 17.1, 13.5, 6.7 Hz, 2H); **^{13}C NMR** (151 MHz, $CDCl_3$) δ 141.9, 133.6, 131.9, 130.7 (q, J = 31.5 Hz), 129.0, 125.0 (q, J = 3.9 Hz), 124.8, 124.2 (q, J = 272.4), 123.1 (q, J = 3.9 Hz), 82.6, 69.6, 60.4, 37.4 (2C), 33.8, 32.4, 32.0, 27.4, 24.5; **^{19}F NMR** (565 MHz, $CDCl_3$) δ –62.5 (3F).

Scheme SI-2 Synthesis of Ether Containing Dimesylate Starting Material

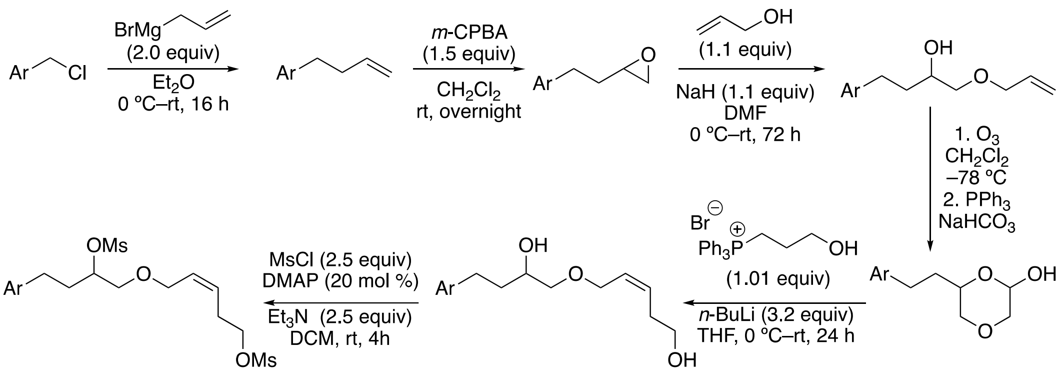

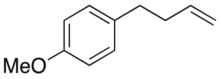

Alkene (SI-37) was prepared according to a procedure reported by Taber.[6] To a flame dried round bottom flask equipped with a stir bar was added 4-methoxybenzyl chloride (1.4 mL, 10. mmol, 1.0 equiv), and THF (50 mL, 0.20 M). The flask was cooled to 0 ºC and allylmagnesium bromide (20. mL, 20. mmol, 2.0 equiv, 1.0 M in Et_2O) was added dropwise. The flask was warmed to rt and allowed to stir overnight. To quench, saturated aq. NH_4Cl was added dropwise. The biphasic mixture was transferred to a separatory funnel and extracted with EtOAc (x 3). The combined organic layers were washed with H_2O, brine, dried over Na_2SO_4, filtered, and concentrated in vacuo. Purification by column chromatography (0–10% EtOAc/hexanes) afforded the desired product as a clear and colorless oil (1.5 g, 9.3 mmol, 93% yield). Analytical data is consistent with literature values.[19] **TLC R$_f$** = 0.8 (5% EtOAc/hexanes); **^{1}H NMR** (400 MHz, $CDCl_3$) δ 7.16 (d, J = 8.6 Hz, 2H), 6.87 (d, J = 8.6 Hz, 2H), 5.90 (ddt, J = 16.9, 10.2, 6.6 Hz, 1H), 5.16 – 4.95 (m, 2H), 3.83 (s, 3H), 2.71 (t, J = 8.4 Hz, 2H), 2.40 (tdt, J = 7.8, 6.6, 1.4 Hz, 2H); **^{13}C NMR** (101 MHz, $CDCl_3$) δ 158.0, 138.4, 134.2, 129.5 (2C), 115.1, 114.0 (2C), 55.4, 36.0, 34.7.

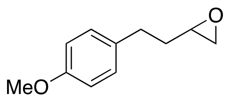

Epoxide (SI-38) was prepared according to a procedure reported by Taber.[6] A flame dried round bottom flask equipped with a stir bar is charged with alkene **SI-37** (1.5 g, 9.3 mmol, 1.0 equiv), CH_2Cl_2 (93 mL, 0.1 M), and *m*-CPBA (3.4 g, 14 mmol, 1.5 equiv, 70% w/w in H_2O). The reaction mixture was allowed to stir at rt for 16 h. Upon completion the reaction mixture was diluted with CH_2Cl_2 and quenched with saturated aq. $NaHSO_3$. The biphasic mixture was transferred to a separatory funnel and the layers were separated. The product was extracted with CH_2Cl_2 (x 3). The combined organic layers were washed with 1M NaOH, then brine, dried over Na_2SO_4, filtered, and concentrated in vacuo. Purification by column chromatography (0–20% EtOAc/hexanes) afforded the title compound as a yellow oil (1.3 g, 7.0 mmol, 75% yield). **^{1}H NMR** (400 MHz, $CDCl_3$) δ 7.13 (d, J = 8.5 Hz, 2H), 6.84 (d, J = 8.7 Hz, 2H), 3.79 (s, 3H), 2.95 (dddd, J = 6.4, 5.1, 4.0, 2.8 Hz, 1H), 2.84–2.66 (m, 3H), 2.47 (dd, J = 5.0, 2.7 Hz, 1H), 1.92–1.76 (m, 2H); **^{13}C NMR** (101 MHz, $CDCl_3$) δ 157.9, 133.3, 129.3 (2C), 113.9 (2C), 55.2, 51.7, 47.2, 34.5, 31.3.

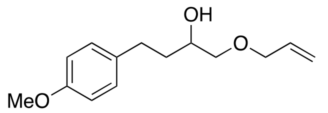

Alkene (SI-39) was prepared according to a procedure reported by Isaacs.[20] In a glovebox, a flame dried round bottom flask equipped with a stir bar was charged with NaH (66 mg, 2.8 mol, 1.1 equiv). The flask was sealed with a septum, removed from the glovebox and placed under an atmosphere of N_2. Next, DMF (13 mL, 0.2 M) was added via syringe and the reaction mixture was cooled to 0 ºC. To the resulting

suspension was added a solution of epoxide **SI-38** (0.45 g, 2.5 mmol, 1.0 equiv) in DMF (2.5 mL, 1.0 M) dropwise via syringe. The reaction mixture was warmed to rt and allowed to stir for 72 h. The reaction was quenched with addition of H_2O and extracted with EtOAc (x 3). The combined organic layers were washed with excess H_2O (x 5) and brine. The organic layer was dried over Na_2SO_4, filtered, and concentrated in vacuo. Purification by column chromatography (0–20% EtOAc/hexanes) afforded the title compound as a pale yellow oil (0.29 g, 1.2 mmol, 50% yield). **TLC R$_f$** = 0.5 (20% EtOAc/hexanes); **^{1}H NMR** (500 MHz, $CDCl_3$) δ 7.11 (d, *J* = 8.4 Hz, 2H), 6.81 (d, *J* = 8.3 Hz, 2H), 5.89 (ddt, *J* = 16.4, 10.8, 5.6 Hz, 1H), 5.26 (d, *J* = 17.2 Hz, 1H), 5.18 (d, *J* = 10.4 Hz, 1H), 4.03–3.97 (m, 2H), 3.76 (s, 3H), 3.44 (dd, *J* = 9.6, 3.1 Hz, 1H), 3.30 (t, *J* = 9.0 Hz, 1H), 2.83–2.70 (m, 1H), 2.68–2.49 (m, 2H), 1.81–1.62 (m, 2H); **^{13}C NMR** (126 MHz, $CDCl_3$) δ 157.9, 134.6, 134.1, 129.4 (2C), 117.3, 113.9 (2C), 74.6, 72.3, 69.7, 55.3, 35.1, 30.9; **HRMS** (TOF MS ES+) *m/z*: $[M+Na]^+$ calcd for $C_{14}H_{20}O_3$, 259.1310; found 259.1322.

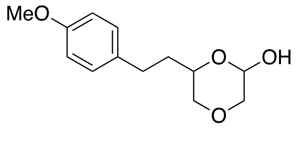

Lactol (SI-40) was prepared according to Method E. The following amounts of reagents were used: alkene **SI-39** (0.16 g, 0.67 mmol, 1.0 equiv), ozone (excess), CH_2Cl_2 (25 mL), triphenylphosphine (0.32 g, 2.0 g/1.0 g of alcohol), $NaHCO_3$ (0.32 g, 2.0 g/1.0 g of alcohol). Purification by column chromatography (0–70% EtOAc/hexanes) afforded the desired product as a white solid (79 mg, 0.33 mmol, 50% yield, 1.5:1 dr). For clarity, the ^{1}H and ^{13}C are tabulated individually.

m.p. = 106–108 ºC; **TLC R$_f$** = 0.5 (60% EtOAc/hexanes); **HRMS** (TOF MS CI+) *m/z*: $[M]^+$ calcd for $C_{13}H_{18}O_4$, 238.1200; found 238.1205.

Major Diastereomer: ^{1}H NMR (400 MHz, $CDCl_3$) δ 7.09 (d, *J* = 8.4 Hz, 2H), 6.82 (d, *J* = 8.6 Hz, 2H), 5.04 (d, *J* = 6.2 Hz, 1H), 4.14 (dddd, *J* = 10.7, 7.9, 4.7, 2.8 Hz, 1H), 3.78 (s, 3H), 3.73 (ddd, *J* = 11.9, 6.4, 1.8 Hz, 2H), 3.62 (dd, *J* = 11.8, 2.0 Hz, 1H), 3.33 (dd, *J* = 11.5, 10.4 Hz, 1H), 3.27 (s, 1H), 2.78–2.51 (m, 2H), 1.84–1.52 (m, 2H); **^{13}C NMR** (101 MHz, $CDCl_3$) δ 158.0, 133.7, 129.4 (2C), 114.0 (2C), 89.8, 71.4, 69.3, 66.6, 55.4, 33.4, 30.3.

Minor Diastereomer: ^{1}H NMR (400 MHz, $CDCl_3$) δ 7.09 (d, *J* = 8.4 Hz, 2H), 6.82 (d, *J* = 8.6 Hz, 2H), 4.87 (ddd, *J* = 9.0, 6.7, 2.6 Hz, 1H), 3.83 (dd, *J* = 11.2, 2.6 Hz, 1H), 3.78 (s, 3H), 3.73 (ddd, *J* = 11.9, 6.4, 1.8 Hz, 2H), 3.62 (dd, *J* = 11.8, 2.0 Hz, 1H), 3.27 (s, 1H), 3.20–3.06 (m, 1H), 2.78–2.51 (m, 2H), 1.84–1.52 (m, 2H); **^{13}C NMR** (101 MHz, $CDCl_3$) δ 158.0, 133.6, 129.4 (2C), 114.0 (2C), 92.3, 73.9, 70.2, 69.8, 55.4, 33.0, 30.4.

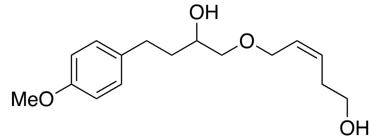

Diol (SI-41) was prepared according to Method G. The following amounts of reagents were used: lactol **SI-40** (0.21 g, 0.88 mmol, 1.0 equiv), Wittig salt **SI-11** (0.36 g, 0.89 mmol, 1.0 equiv), *n*-BuLi (1.2 mL, 2.8 mmol, 3.2 equiv, 2.5 M in hexanes), and THF (4.4 mL). Purification by column chromatography afforded a mixture of the title compound with triphenylphosphine oxide. This mixture was carried onto the next step without further purification.

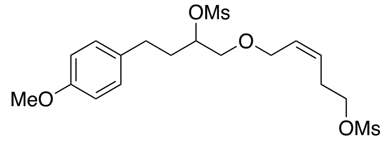

Dimesylate (SI-6) was prepared according to Method H. The following amounts of reagents were used: diol **SI-41** (0.29 g, 1.0 mmol, 1.0 equiv), MsCl (0.20 mL, 2.6 mmol, 2.5 equiv), DMAP (25 mg, 0.21 mmol, 20 mol %), Et_3N (0.36 mL, 2.6 mmol, 2.5 equiv), and DCM (5.2 mL). Purification by column chromatography (0–60% EtOAc/hexanes) afforded the desired product as a pale yellow oil (197 mg, 0.45 mmol, 44% yield, 2:1 dr). For clarity, the ^{1}H and ^{13}C are tabulated individually.

TLC R$_f$ = 0.6 (60% EtOAc/hexanes); **HRMS** (TOF MS ES+) *m/z*: [M + NH_4] calcd for $C_{18}H_{32}NO_8S_2$ 454.1569, found 454.1582.

Major Diastereomer: ^{1}H NMR (400 MHz, $CDCl_3$) δ 7.11 (d, *J* = 8.6 Hz, 2H), 6.83 (d, *J* = 8.7 Hz, 2H), 5.75–5.54 (m, 2H), 4.80 (dddd, *J* = 9.1, 7.8, 4.5, 1.2 Hz, 1H), 4.23 (dt, *J* = 9.7, 6.6 Hz, 2H), 4.15–3.92 (m, 2H), 3.77 (s, 3H), 3.64–3.52 (m, 2H), 3.06 (s, 3H), 2.99 (s, 3H), 2.81–2.60 (m, 2H), 2.57–2.44 (m, 2H), 1.95 (tddt, *J* = 14.4, 9.8, 6.7, 4.9 Hz, 2H); **^{13}C NMR** (101 MHz, $CDCl_3$) δ 158.0, 132.7, 129.3 (2C), 128.2, 127.4, 114.0 (2C), 81.7, 71.7, 68.9, 66.6, 55.3, 38.7, 37.4, 33.6, 30.3, 27.8.

Minor Diastereomer: ^{1}H NMR (400 MHz, $CDCl_3$) δ 7.11 (d, *J* = 8.6 Hz, 2H), 6.83 (d, *J* = 8.7 Hz, 2H), 5.75–5.54 (m, 2H), 4.80 (dddd, *J* = 9.1, 7.8, 4.5, 1.2 Hz, 1H), 4.23 (dt, *J* = 9.7, 6.6 Hz, 2H), 4.15–3.92 (m, 2H), 3.77 (s, 3H), 3.64–3.52 (m, 2H), 3.06 (s, 3H), 2.99 (s, 3H), 2.81–2.60 (m, 2H), 2.57–2.44 (m, 2H), 1.95 (tddt, *J* = 14.4, 9.8, 6.7, 4.9 Hz, 2H); **^{13}C NMR** (101 MHz, $CDCl_3$) δ 158.0, 132.7, 129.7, 129.4 (2C), 127.4, 114.1 (2C), 81.7, 71.7, 68.8, 66.6, 55.3, 38.7, 37.5, 33.6, 30.3, 27.8.

Scheme SI-3 Synthesis of Trisubstituted Alkene Dimesylate Starting Material

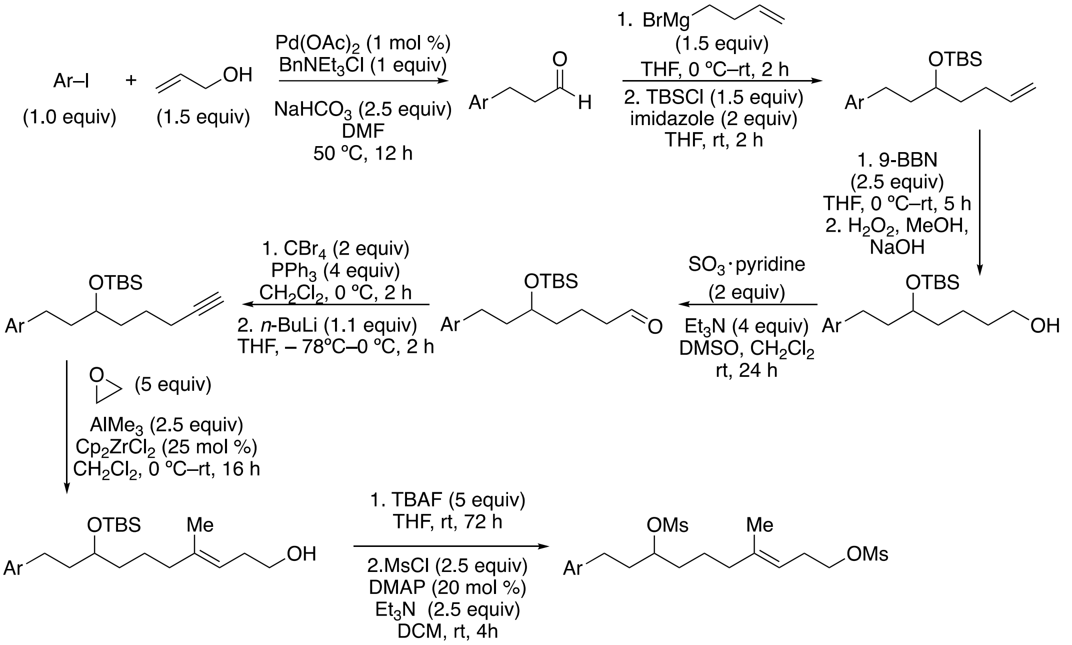

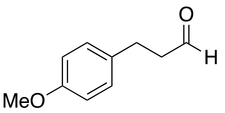

Aldehyde (SI-12) was prepared according to Method B. Refer above for analytical data.

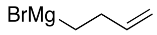

But-3-en-1-ylmagnesium bromide (SI-42) was prepared according to Method C. The following amounts of reagents were used: 4-bromo-butene (4.1 mL, 40. mmol, 1.0 equiv), Mg^0 (1.4 g, 60. mmol, 1.5 equiv), and THF (20 mL). The resulting Grignard reagent was titrated to be 1.3 M.

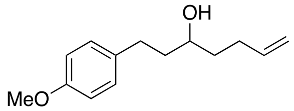

Alcohol (SI-43) was prepared according to a modified version of Method D. The following amounts of reagents were used: aldehyde **SI-12** (2.6 g, 16 mmol, 1.0 equiv), Grignard reagent **SI-42** (19 mL, 24 mmol, 1.5 equiv, 1.3 M in THF), and THF (80 mL). The crude reaction mixture was passed through a plug of silica gel eluting with 100% EtOAc to remove any remaining magnesium salts and was carried into the next step without further purification.

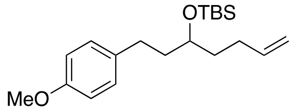

TBS Ether (SI-44) was prepared according to Method I. The following amount of reagents were used: alcohol **SI-43** (3.5 g, 16 mmol, 1.0 equiv), TBSCl (3.6 g, 24 mmol, 1.5 equiv), imidazole (2.2 g, 32 mmol, 2.0 equiv), and CH_2Cl_2 (81 mL, 0.20 M). Purification by column chromatography (0–30% EtOAc/hexanes) afforded the title compound as a pale yellow oil (4.6 g, 13 mmol, 86% yield). **TLC R$_f$** = 0.8 (20% EtOAc/hexanes); **^{1}H NMR** (400 MHz, $CDCl_3$) δ 7.17 (d, *J* = 7.9 Hz, 2H), 6.90 (d, *J* = 8.1 Hz, 2H), 5.99–5.83 (m, 1H), 5.17–4.98 (m, 2H), 3.84 (s, 4H), 2.80–2.58 (m, 2H), 2.29–2.13 (m, 2H), 1.83 (aq, *J* = 7.8, 7.3, 6.6 Hz, 2H), 1.69 (aq, *J* = 7.9, 7.4, 6.7 Hz, 2H), 1.05–1.02 (m, 9H), 0.16 (dd, *J* = 3.3, 1.5 Hz, 6H); **^{13}C NMR** (101 MHz, $CDCl_3$) δ 158.0, 139.1, 134.9, 129.4 (2C), 114.6, 114.1 (2C), 71.6, 55.4,

39.5, 36.5, 31.0, 29.9, 26.2 (3C), 18.4, -4.1 (2C); **HRMS** (TOF MS CI+) *m/z*: [M+H] calcd for $C_{20}H_{35}O_2Si$ 335.2406, found 335.2402.

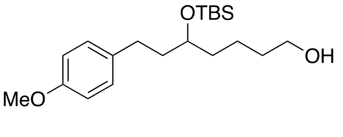

Alcohol (SI-45) was prepared according to Method J. The following amounts of reagents were used: alkene **SI-44** (4.6 g, 14 mmol, 1.0 equiv), 9-BBN (69 mL, 35 mmol, 1.5 equiv, 0.50 M in THF), THF (36 mL), MeOH (42 mL), H_2O_2 (14 mL), and NaOH (42 mL, 1.0 M). Purification by column chromatography (0–30% EtOAc/hexanes) afforded a mixture of the title compound and unreacted 9-BBN as a pale yellow oil. This mixture was carried into the next step without further purification.

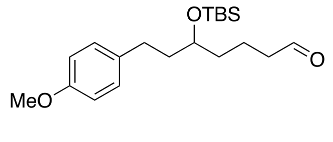

Aldehyde (SI-46) was prepared according to Method K. The following amounts of reagents were used: alcohol **SI-45** (1.1 g, 3.0 mmol, 1.0 equiv), SO_3·pyridine (0.96 g, 6.0 mmol, 2.0 equiv), Et_3N (1.7 mL, 12 mmol, 4.0 equiv), DMSO (3.0 mL), and CH_2Cl_2 (3.0 mL). Purification by column chromatography (0–10% EtOAc/hexanes) afforded the title compound as a yellow oil (0.80 g, 2.3 mmol, 76% yield). **TLC R_f** = 0.6 (20% EtOAc/hexanes, CAM stain); **^{1}H NMR (**400 MHz, $CDCl_3$) δ 9.76 (t, *J* = 1.7 Hz, 1H), 7.08 (d, *J* = 8.7 Hz, 2H), 6.82 (d, *J* = 8.6 Hz, 2H), 3.78 (s, 3H), 3.72 (p, *J* = 5.7 Hz, 1H), 2.57 (qt, *J* = 13.8, 8.1 Hz, 2H), 2.46–2.37 (m, 2H), 1.95–1.82 (m, 2H), 1.79–1.59 (m, 2H), 1.59–1.46 (m, 2H), 0.91 (s, 9H), 0.05 (d, *J* = 5.6 Hz, 6H); **^{13}C NMR** (101 MHz, $CDCl_3$) δ 202.6, 157.9, 134.7, 129.3 (2C), 113.9 (2C), 71.6, 55.4, 44.1, 42.1, 39.2, 36.4, 30.8, 26.1 (3C), 18.0, -4.3 (2C); **HRMS** (TOF MS ES+) *m/z*: $[M+H]^+$ calcd for $C_{20}H_{35}O_3Si$, 351.2355; found 351.2349.

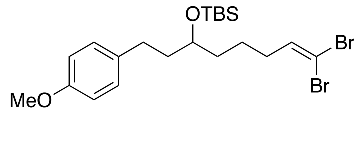

Gemdibromide (SI-47) was prepared according to Method L. The following amounts of reagents were used: aldehyde **SI-46** (0.31 g, 0.88 mmol, 1.0 equiv), CBr_4 (0.58 g, 1.8 mmol, 2.0 equiv), PPh_3 (0.92 g, 3.5 mmol, 4.0 equiv), and CH_2Cl_2 (10. mL). The resulting crude reaction mixture was carried into the next step without further purification (0.38 g, 0.70 mmol, 84% yield). **TLC R_f** = 0.6 (20% EtOAc/hexanes, CAM stain); **^{1}H NMR** (400 MHz, $CDCl_3$) δ 7.12 (d, *J* = 8.6 Hz, 2H), 6.84 (d, *J* = 8.6 Hz, 2H), 6.40 (t, *J* = 7.2 Hz, 1H), 3.80 (s, 3H), 3.78–3.71 (m, 1H), 2.70–2.45 (m, 3H), 2.45–2.37 (m, 1H), 2.16–2.08 (m, 2H), 1.90 (dtt, *J* = 9.0, 6.2, 2.6 Hz, 2H), 1.75 (tdd, *J* = 8.5, 5.7, 1.2 Hz, 2H), 0.95 (s, 9H), 0.11–0.06 (m, 6H); **^{13}C NMR** (101 MHz, $CDCl_3$) δ 157.9, 138.8, 134.7, 129.3 (2C), 113.9 (2C), 89.0, 71.6, 55.4, 39.3, 36.4, 33.3, 30.9, 26.1 (3C), 23.6, 18.3, -4.2 (2C).

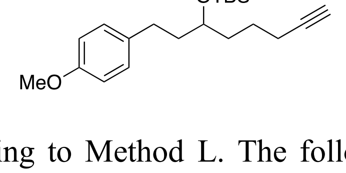

Alkyne (SI-48) was prepared according to Method L. The following amount of reagents were used: gemdibromide **SI-47** (1.1 g, 2.1 mmol, 1.0 equiv), *n*-BuLi (2.3 mL, 3.2 mmol, 1.5 equiv, 1.4 M in hexanes), and THF (10. mL). Purification by column chromatography (0–10% EtOAc/hexanes) afforded the title compound as a pale yellow oil (0.43 g, 1.2 mmol, 53% yield). **TLC R_f** = 0.6 (20% EtOAc/hexanes, CAM Stain); **^{1}H NMR** (400 MHz, $CDCl_3$) δ 7.10 (d, *J* = 8.8 Hz, 2H), 6.84 (d, *J* = 8.6 Hz, 2H), 3.80 (s,

3H), 3.74 (p, *J* = 5.4 Hz, 1H), 2.69–2.49 (m, 2H), 2.25–2.15 (m, 2H), 1.96 (t, *J* = 2.6 Hz, 1H), 1.74 (ddd, *J* = 9.1, 7.9, 5.7 Hz, 2H), 1.67–1.55 (m, 3H), 0.92 (s, 10H), 0.07 (d, *J* = 2.8 Hz, 6H); **^{13}C NMR** (101 MHz, $CDCl_3$) δ 157.8, 134.8, 129.3 (2C), 113.9 (2C), 84.6, 71.6, 68.5, 55.4, 39.4, 36.1, 30.9, 26.1 (3C), 24.4, 18.8, 18.3, -4.2 (2C).

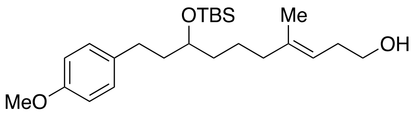

Alcohol (SI-49) was prepared according to a procedure reported by Sigman.[21] A flame dried round bottom flask equipped with a stir bar is charged with Cp_2ZrCl_2 (31 mg, 0.11 mmol, 25 mol %) and CH_2Cl_2 (0.21 mL, 2.0 M). The flask was cooled to 0 ºC and $AlMe_3$ (0.54 mL, 1.1 mmol, 2.5 equiv, 2.0 M in PhMe) was added dropwise via syringe and the mixture was allowed to stir for 30 mins. Then, a solution of alkyne **SI-48** (150 mg, 0.43 mmol, 1.0 equiv) in CH_2Cl_2 (0.45 mL, 0.25 M) was added via syringe. The reaction mixture was warmed to rt and allowed to stir overnight. Then the mixture was cooled to 0 ºC and ethylene oxide (1.1 mL, 2.2 mmol, 5.0 equiv, 2.5–3.3 M in THF) was added in one portion via syringe. The mixture was allowed to stir for an additional 3 h. To quench, the reaction mixture was cooled to 0 ºC and 1 M HCl was added dropwise. The mixture was then passed through a pad of Celite eluting with 100% CH_2Cl_2. The biphasic mixture was transferred to a separatory funnel and extracted with CH_2Cl_2 (x 3). The combined organic layers were washed with 1 M HCl and then brine. Then the organic layer was dried over Na_2SO_4, filtered, and concentrated in vacuo. Purification by column chromatography (0–30% EtOAc/hexanes) afforded the desired product as a pale yellow oil (69 mg, 0.17 mmol, 40% yield, >20:1 dr). **TLC R_f** = 0.3 (30% EtOAc/hexanes); **^{1}H NMR** (400 MHz, $CDCl_3$) δ 7.10 (d, *J* = 8.4 Hz, 2H), 6.83 (d, *J* = 8.4 Hz, 2H), 5.14 (t, *J* = 7.2 Hz, 1H), 3.79 (s, 3H), 3.71 (t, *J* = 5.0 Hz, 1H), 3.66–3.60 (m, 3H), 3.58 (t, *J* = 5.3 Hz, 1H), 2.75–2.46 (m, 2H), 2.30 (q, *J* = 6.8 Hz, 2H), 2.00 (d, *J* = 9.0 Hz, 2H), 1.80–1.70 (m, 4H), 1.46 (qd, *J* = 11.9, 7.2 Hz, 4H), 0.92 (s, 9H), 0.07 (d, *J* = 4.4 Hz, 6H); **^{13}C NMR** (101 MHz, $CDCl_3$) δ 157.8, 138.8, 134.9, 129.3 (2C), 120.0, 113.9 (2C), 71.9, 69.3, 62.6, 55.4, 40.0, 36.7, 31.7, 31.0, 26.1 (3C), 23.6, 18.3, 16.2, -4.2 (2C).

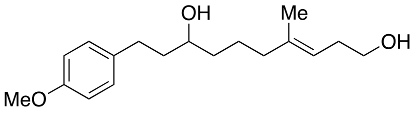

Diol (SI-50) was prepared according to Method M. The following amount of reagents were used: silylprotected alcohol **SI-49** (69 mg, 0.17 mmol, 1.0 equiv), TBAF (0.85 mL, 0.85 mmol, 5.0 equiv, 1.0 M in THF), and THF (0.85 mL, 0.2 M). Purification by column chromatography (0–30% EtOAc/hexanes) afforded the title compound as a pale yellow oil (40. mg, 0.14 mmol, 80% yield).

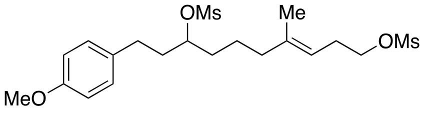

Dimesylate (SI-7) was prepared according to Method H. The following amounts of reagents were used: diol **SI-50** (40 mg, 0.14 mmol, 1.0 equiv), MsCl (30. µL, 0.35 mmol, 2.5 equiv), DMAP (3.4 mg, 28 µmol, 20 mol %), Et_3N (50. µL, 0.35 mmol, 2.5 equiv), and CH_2Cl_2 (0.70 mL). Purification by column chromatography (0–50% EtOAc/hexanes) afforded the desired product as a pale yellow oil (27 mg, 61 µmol, 44% yield). **TLC R_f** = 0.4 (50% EtOAc/hexanes, CAM stain); **^{1}H NMR** (400 MHz, $CDCl_3$) δ 7.11 (d, *J* = 8.3 Hz, 2H), 6.83 (d, *J* = 8.4 Hz, 2H), 5.12 (t, *J* = 6.8 Hz, 1H), 4.76 (h, *J* = 6.5, 6.0 Hz, 1H), 4.18 (t, *J* = 6.9 Hz, 2H), 3.78 (s, 3H), 3.06–2.92 (m, 6H), 2.78–2.58 (m, 2H), 2.46 (q, *J* = 7.0 Hz, 1H), 2.09–1.89 (m, 4H), 1.80–1.65 (m, 2H), 1.60–1.43 (m, 3H), 1.09 (d, *J* = 1.4 Hz, 3H); **^{13}C NMR** (101 MHz, $CDCl_3$) δ 158.2, 138.9, 133.0, 129.4 (2C), 118.5, 114.1 (2C), 83.2, 69.6, 55.4, 39.2, 38.9, 37.6, 36.5, 34.1, 30.5, 28.2, 23.0, 16.2.

g. Synthesis and Characterization Data for Mechanistic Experiments

1) Mechanistic Experiment with Single Alkene Diastereomer

Scheme SI-4 Results from Single Alkene Diastereomer Experiment

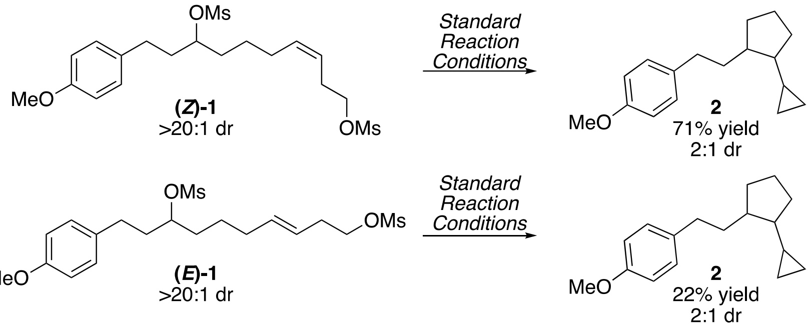

The control reaction was performed according to Method A. Two separate reactions were set up in 7-mL dram vials. The following amounts of reagents were used:

Vial 1: (*Z*)-**1** (61 mg, 0.14 mmol, 1.0 equiv), Ni(cod)$_2$ (3.9 mg, 14 μmol, 10 mol %), *rac*-BINAP (8.7 mg, 14 μmol, 10 mol %), MeMgI (0.11 mL, 0.28 mmol, 2.0 equiv, 2.9 M in Et$_2$O), PhMe (0.70 mL), AD-mix-β (196 mg, 1.4 g/mol substrate), *t*-BuOH (0.50 mL), and H_2O (0.50 mL). Purification by column chromatography (0–10% EtOAc/hexanes) afforded the desired product **2** (24 mg, 98 μmol, 71% yield, 2:1 dr).

Vial 2: (*E*)-**1** (17 mg, 40 μmol, 1.0 equiv), Ni(cod)$_2$ (1.1 mg, 4.0 μmol, 10 mol %), *rac*-BINAP (2.5 mg, 4.0 μmol, 10 mol %), MeMgI (30. μL, 80. μmol, 2.0 equiv, 2.9 M in Et$_2$O), PhMe (0.20 mL), AD-mix-β (56 mg, 1.4 g/mol substrate), *t*-BuOH (0.30 mL), and H_2O (0.30 mL). Purification by column chromatography (0–10% EtOAc/hexanes) afforded the desired product **2** (2.1 mg, 8.5 μmol, 22% yield, 2:1 dr).

2) Separation of Alkene Diastereomers on Silver Impregnated Silica

$AgNO_3$ (10 g) was dissolved in H_2O (2.5 mL) and MeCN (100 mL) in a 500 mL erlenmeyer flask. The stock solution was poured into a 500 mL round bottom flask that contained 100 g of silica gel and a stir bar. The slurry was allowed to stir at rt for 2 h and then was concentrated in vacuo in the dark. The silica gel was dried on a high vacuum manifold overnight and resulted in a cream colored powder. The silica gel was then used to separate the alkene diastereomers.[22]

3) SmI_2 Control Reaction

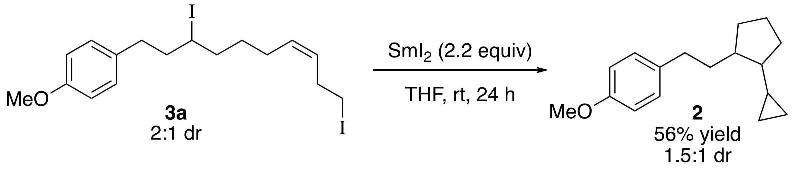

(SI-15)

Preparation of SmI_2 in THF: SmI_2 was prepared as a solution in THF according to a procedure reported by Procter.[23] In a glovebox, Sm metal (0.60 g, 4.0 mmol, 2.0 equiv) was added to a flame dried Schlenk flask equipped with a stir bar. The flask was sealed with a septum, removed from the glovebox, and placed under an N_2 atmosphere. Then, freshly washed 1,2-diiodoethane (0.56 g, 2.0 mmol, 1.0 equiv) was added. The flask was evacuated and backfilled with N_2 (x 3). Then THF (20. mL) was added via syringe and the resulting yellow solution was evacuated until the solvent began to bubble and backfilled with N_2 (x 3).

Then the mixture was allowed to stir overnight at rt in the dark and resulted in a dark blue solution. The SmI_2 was titrated according to a procedure reported by Hilmersson and used immediately.[24]

Control Reaction with SmI_2: To a flame dried round bottom flask equipped with a stir bar was added diiodide **3a** (27 mg, 54 µmol, 1.0 equiv) and THF (0.27 mL). Then SmI_2 (3.0 mL, 0.12 mmol, 2.2 equiv, 42 µM in THF) was added dropwise via syringe. The reaction mixture was stirred at rt overnight. MeOH was added to quench and the reaction mixture was filtered through a pad of silica gel eluting with 100% Et_2O. Purification by column chromatography (100% hexanes) afforded the cascade product **2** (7.4 mg, 30 µmol, 56% yield, 1.5:1 dr). Refer above for characterization data.

4) TEMPO Control Reaction

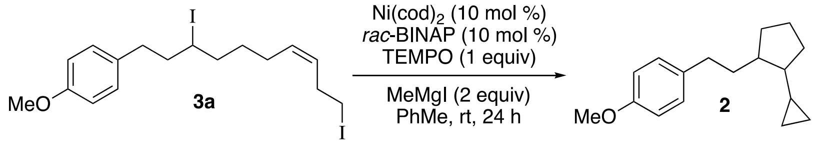

(SI-16)

The mechanistic experiment with TEMPO as an additive was performed according to a modified version of Method A. The following amounts of reagents were used: diiodide **3a** (25 mg, 50. µmol, 1.0 equiv), $Ni(cod)_2$ (1.4 mg, 5.0 µmol, 10. mol %), *rac*-BINAP (3.1 mg, 5.0 µmol, 10. mol %), MeMgI (30. µL, 0.10 mmol, 2.0 equiv, 2.9 M in Et_2O), TEMPO (7.8 mg, 50. µmol, 1.0 equiv), and PhMe (0.25 mL). The reaction was quenched with MeOH and filtered through a pad of silica gel eluting with 100% Et_2O. A 48% ^1H NMR yield of **2** was observed compared to PhTMS as an internal standard.

h. Competition Experiment

The competition experiment was performed according to a modified version of Method A. Two separate reactions were set up for each additive in different 7-mL dram vials. The following amounts of reagents were used:

Vial 1: dimesylate **1** (47 mg, 0.11 mmol, 1.0 equiv), secondary mesylate **12** (45 mg, 0.11 mmol, 1.0 equiv), Ni(cod)$_2$ (3.1 mg, 11 µmol, 10. mol %), *rac*-BINAP (6.8 mg, 11 µmol, 10. mol %), MeMgI (0.11 mL, 0.33 mmol, 3.0 equiv, 3.0 M in Et$_2$O), and PhMe (0.55 mL). The crude reaction mixture was concentrated to remove PhMe and was diluted to 300 µM in DCM and analyzed by GCMS. All area percent values were obtained from GCMS spectrum.

Vial 2: dimesylate **1** (43 mg, 0.10 mmol, 1.0 equiv), primary mesylate **15** (36 mg, 0.10 mmol, 1.0 equiv), Ni(cod)$_2$ (2.8 mg, 10. µmol, 10. mol %), *rac*-BINAP (6.2 mg, 10. µmol, 10. mol %), MeMgI (0.10 mL, 0.30 mmol, 3.0 equiv, 3.0 M in Et$_2$O), and PhMe (0.50 mL). The crude reaction mixture was concentrated to remove PhMe and was diluted to 300 µM in DCM and analyzed by GCMS. All area percent values were obtained from GCMS spectrum.

Table SI-1. Area Percent Values and Ratio Calculations for the Competition Experiment

Compound	Area	Area Percent	
PhTMS	16627132	16.692311	
Trimethoxybenzene	33349020	33.479750	ratio
Product **2**	30001111	30.118717	1.5281535
Products from 2º mesylate (**13**,**14**)	19632263	19.709222	
**Total**	99609526		

Compound	Area	Area Percent	
PhTMS	28142418	16.813243	
Trimethoxybenzene	48364556	28.894640	
Product **2**	37642540	22.488941	
Products from 1º mesylate (**17**)	5668865	3.3867739	ratio
Kumada (**16**)	8612848	5.1456100	2.6357160
Iodide (**SI-51**)	38951221	23.270792	
**Total**	167382448		

i. Synthesis and Characterization Data for Competition Experiment

Scheme SI-5 Synthesis of Secondary Mesylate for Competition Experiment

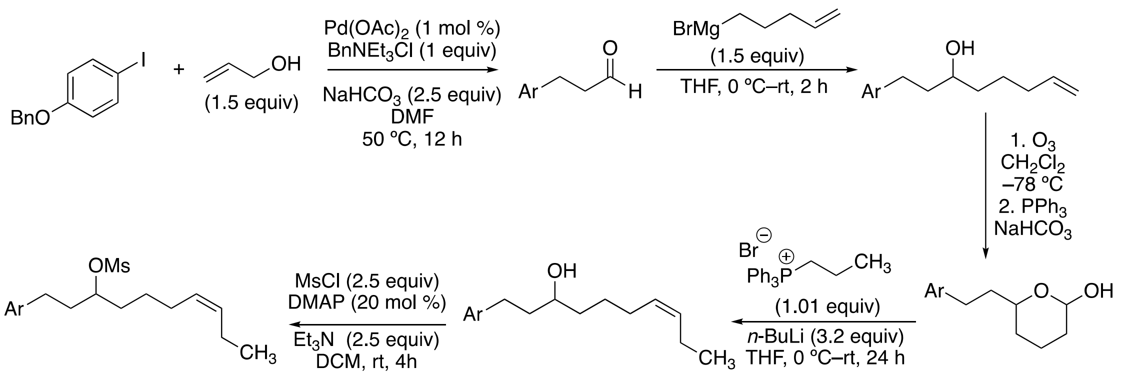

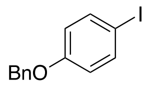

Iodide (SI-20) refer above for synthesis and characterization data.

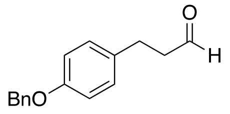

Aldehyde (SI-21) refer above for synthesis and characterization data.

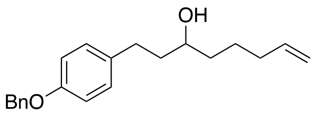

Alcohol (SI-22) refer above for synthesis and characterization data.

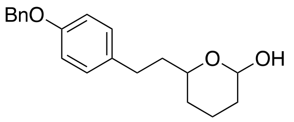

Lactol (SI-23) refer above for synthesis and characterization data.

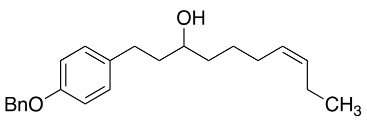

Alcohol (SI-52) was prepared according to Method G. The following amounts of reagents were used: lactol **SI-23** (0.10 g, 0.32 mmol, 1.0 equiv), propyltriphenylphosphonium bromide (0.13 g, 0.32 mmol, 1.0 equiv), *n*-BuLi (0.40 mL, 0.96 mmol, 3.0 equiv, 2.5 M in hexanes), and THF (1.6 mL). Purification by column chromatography (0–50% EtOAc/hexanes) afforded the title compound as a white solid (59 mg, 0.17 mmol, 55% yield, 2:1 dr). **TLC R$_f$** = 0.3 (30% EtOAc/hexanes, $KMnO_4$ stain); **^{1}H NMR** (500 MHz,

$CDCl_3$) δ 7.45–7.40 (m, 4H, both diastereomers), 7.37 (ddd, *J* = 9.9, 5.6, 1.6 Hz, 4H, both diastereomers), 7.33–7.28 (m, 2H, both diastereomers), 7.11 (d, *J* = 8.5 Hz, 4H, both diastereomers), 6.90 (d, *J* = 8.5 Hz, 4H, both diastereomers), 4.68 (br s, 2H, both diastereomers), 5.47–5.25 (m, 4H, both diastereomers), 5.03 (s, 4H, both diastereomers), 3.60 (tt, *J* = 8.1, 4.0 Hz, 2H, both diastereomers), 2.72 (ddd, *J* = 14.9, 9.6, 5.7 Hz, 2H, both diastereomers), 2.60 (ddd, *J* = 13.9, 9.5, 6.8 Hz, 2H, both diastereomers), 2.09–1.93 (m, 8H, both diastereomers), 1.81–1.63 (m, 4H, both diastereomers), 1.54–1.32 (m, 8H, both diastereomers), 0.95 (td, *J* = 7.4, 4.4 Hz, 6H, both diastereomers); **^{13}C NMR** (126 MHz, $CDCl_3$) δ 157.1 (2C, both diastereomers), 137.3 (2C, both diastereomer), 134.6 (2C, both diastereomers), 132.5 (minor diastereomer), 132.1 (major diastereomer), 129.4 (4C, both diastereomers), 128.9 (2C, both diastereomers), 128.7 (4C, both diastereomers), 128.0 (2C, both diastereomer), 127.6 (4C, both diastereomers), 114.9 (4C, both diastereomers), 71.3 (2C, both diastereomers), 70.1 (2C, both diastereomers), 39.4 (2C, both diastereomers), 37.2 (major diastereomers), 37.1 (minor diastereomer), 31.2 (2C, both diastereomers), 27.1 (2C, both diastereomers), 25.8 (major diastereomer), 25.7 (minor diastereomers), 20.6 (2C, both diastereomers), 14.5 (major diastereomer), 14.1 (minor diastereomer); **HRMS** (TOF MS ES+) *m/z*: [M+Na] calcd for $C_{23}H_{30}O_2$, 361.2144; found 361.2129.

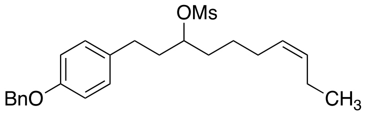

Mesylate (12) was prepared according to Method H. The following amounts of reagents were used: alcohol **SI-52** (0.12 g, 0.34 mmol, 1.0 equiv), MsCl (40. µL, 0.51 mmol, 1.5 equiv), Et_3N (70. µL, 0.51 mmol, 1.5 equiv), and CH_2Cl_2 (1.7 mL). Purification by column chromatography (0–30% EtOAc/hexanes) afforded the title compound as a yellow oil (120 mg, 0.30 mmol, 87% yield). **TLC R_f** = 0.8 (50% EtOAc/hexanes, CAM Stain); **^{1}H NMR** (600 MHz, $CDCl_3$) δ 7.42 (d, *J* = 6.9 Hz, 4H, both diastereomers), 7.38 (t, *J* = 7.7 Hz, 4H, both diastereomers), 7.34–7.29 (m, 2H, both diastereomers), 7.11 (d, *J* = 8.5 Hz, 4H, both diastereomers), 6.90 (d, *J* = 8.5 Hz, 4H, both diastereomers), 5.54–5.21 (m, 4H, both diastereomers), 5.04 (s, 4H, both diastereomers), 4.75 (quint, *J* = 6.0 Hz, 2H, both diastereomers), 2.98 (s, 6H, both diastereomers), 2.75–2.58 (m, 4H, both diastereomers), 2.11–1.91 (m, 12H, both diastereomers), 1.80–1.68 (m, 4H, both diastereomers), 1.53–1.39 (m, 4H, both diastereomers), 0.96 (td, *J* = 7.4, 4.1 Hz, 6H, both diastereomers); **^{13}C NMR** (151 MHz, $CDCl_3$) δ 157.35 (major diastereomer), 157.34 (minor diastereomer), 137.2 (2C, both diastereomers), 133.4 (minor diastereomer), 133.1 (major diastereomer), 132.7 (2C, both diastereomers), 129.4 (4C, both diastereomers), 128.7 (4C, both diastereomers), 128.3 (minor diastereomer), 128.2 (major diastereomer), 128.1 (2C, both diastereomers), 127.6 (4C, both diastereomers), 115.1 (4C, both diastereomers), 83.43 (minor diastereomer), 84.36 (major diastereomer), 70.2 (2C, both diastereomers), 38.9 (2C both diastereomers), 36.49 (major diastereomer), 36.47 (minor diastereomer), 34.13 (major diastereomer), 34.00 (minor diastereomer), 32.24 (minor diastereomer), 30.51 (minor diastereomer), 30.50 (major diastereomer), 26.8 (major diastereomer), 25.7 (minor diastereomer), 25.1 (major diastereomer), 24.9 (minor diastereomer), 20.69 (major diastereomers), 14.47 (major diastereomer), 14.06 (minor diastereomer); **HRMS** (TOF MS ES+) *m/z*: [M+Na] calcd for $C_{24}H_{32}O_4SNa$, 439.1919; found 439.1915.

Scheme SI-6 Synthesis of Primary Mesylate for Competition Experiment

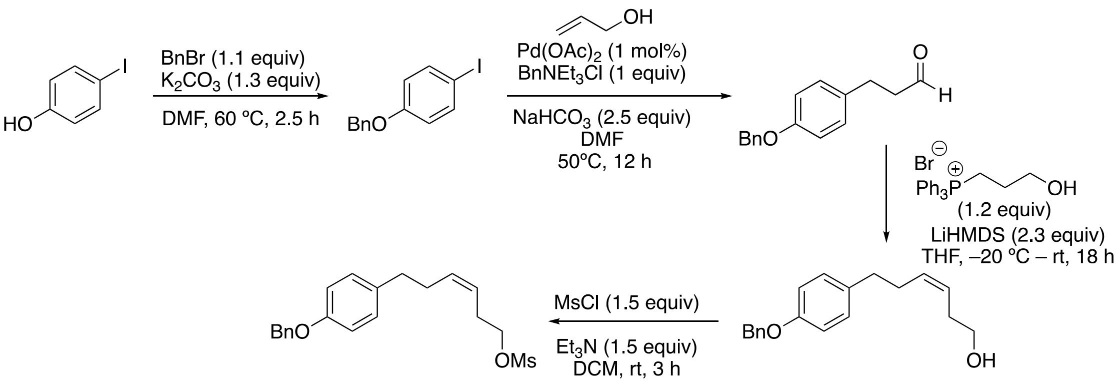

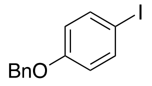

Iodide (SI-20) refer above for synthesis and characterization data.

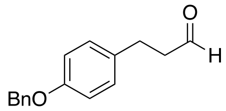

Aldehyde (SI-21) refer above for synthesis and characterization data.

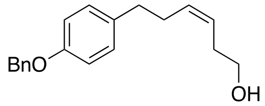

Alcohol (SI-53) was prepared according to a procedure reported by Liu.[25] To a flame dried round bottom flask was added Wittig salt **SI-11** (0.45 g, 1.1 mmol, 1.2 equiv) and THF (2.4 mL). The flask was cooled to – 20 ºC[26] and LiHMDS (0.36 mg, 2.2 mmol, 2.3 equiv) was added as a solution in PhMe (1.0 M) dropwise via syringe. The dark orange suspension was allowed to stir at – 20 ºC for 1 h, then aldehyde **SI-21** (0.27 g, 1.1 mmol, 1.0 equiv) was added as a solution in THF (1.0 mL) dropwise. The reaction mixture was allowed to stir at – 20 ºC for 2 h and then slowly allowed to warm to rt overnight. To quench, saturated aq NH_4Cl was added dropwise. The biphasic mixture was transferred to a separatory funnel and extracted with EtOAc (x 3). The combined organic layers were washed with H_2O and brine, dried over Na_2SO_4, filtered, and concentrated in vacuo. Purification by column chromatography (0–20% EtOAc/hexanes) afforded the title compound as a white solid (140 mg, 0.50 mmol, 45% yield, 1.5:1 dr). **TLC R$_f$** = 0.3 (30% EtOAc/hexanes, CAM Stain); **^{1}H NMR** (400 MHz, $CDCl_3$) δ 7.50–7.29 (m, 10H, both diastereomers), 7.11 (dd, *J* = 8.7, 7.1 Hz, 4H, both diastereomers), 6.93 (d, *J* = 8.4 Hz, 4H, both diastereomers), 5.66–5.53 (m, 2H, both diastereomers), 5.49–5.32 (m, 2H, both diastereomers), 5.06 (s, 4H, both diastereomers), 3.59 (dt, *J* = 10.5, 6.4 Hz, 4H, both diastereomers), 2.65 (td, *J* = 7.6, 4.9 Hz, 4H, both diastereomers), 2.32 (ddt, *J* = 37.5, 12.4, 6.8 Hz, 8H, both diastereomers); **^{13}C NMR** (101 MHz, $CDCl_3$) δ 157.2 (2C, both diastereomers), 137.4 (2C, both diastereomers), 137.4 (minor diastereomer), 133.3 (major diastereomer), 132.2 (2C, both diastereomers), 129.5 (4C, both diastereomers), 128.7 (4C, both diastereomers), 128.0 (2C, both diastereomer), 127.6 (4C, both diastereomers), 126.8 (major diastereomer), 126.0 (minor diastereomer), 114.9 (4C, both diastereomers), 70.2 (2C, both diastereomers), 62.3 (minor diastereomer), 62.1 (major diastereomer), 36.1 (2C, both diastereomers), 35.1 (major diastereomer), 34.8 (minor diastereomer), 30.9 (minor diastereomer), 29.6 (major diastereomer); **HRMS** (TOF MS ES+) *m/z*: [M+Na] calcd for $C_{19}H_{22}O_2Na$, 305.1518; found 305.1521.

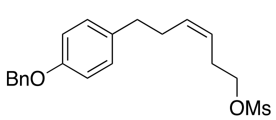

Mesylate (15) was prepared according to Method H. The following amounts of reagents were used: alcohol **SI-53** (140 mg, 0.50 mmol, 1.0 equiv), MsCl (60. µL, 0.74 mmol, 1.5 equiv), Et_3N (0.10 mL, 0.74 mmol, 1.5 equiv) and CH_2Cl_2 (2.5 mL). Purification by column chromatography (0–30% EtOAc/hexanes) afforded the title compound as a yellow oil (120 mg, 0.33 mmol, 68% yield, 1.5:1 dr). **TLC** $\mathbf{R_f}$ = 0.5 (50% EtOAc/hexanes, CAM Stain); **^{1}H NMR** (400 MHz, $CDCl_3$) δ 7.43 (d, J = 7.2 Hz, 4H, both diastereomers), 7.38 (t, J = 7.2 Hz, 4H, both diastereomers), 7.34–7.28 (m, 2H, both diastereomers), 7.08 (dd, J = 7.2, 4.8 Hz, 4H, both diastereomers), 6.90 (d, J = 8.5 Hz, 4H, both diastereomers), 5.66–5.53 (m, 2H, both diastereomers), 5.43–5.31 (m, 2H, both diastereomers), 5.04 (s, 4H, both diastereomers), 4.18 (t, J = 6.8 Hz, 2H, both diastereomers), 4.05 (t, J = 6.9 Hz, 2H, both diastereomers), 2.95 (s, 3H, major diastereomer), 2.94 (s, 3H, minor diastereomer), 2.62 (t, J = 7.6 Hz, 4H, both diastereomers), 2.46–2.25 (m, 8H, both diastereomers); **^{13}C NMR** (101 MHz, $CDCl_3$) δ 157.23 (minor diastereomer), 157.17 (major diastereomer), 137.34 (major diastereomer), 137.31 (minor diastereomer), 134.2 (major diastereomer), 134.1 (minor diastereomer), 134.0 (major diastereomer), 133.0 (minor diastereomer), 129.6 (2C, minor diastereomer), 129.5 (2C, major diastereomers), 128.7 (4C, both diastereomers), 128.01 (minor diastereomer), 128.00 (major diastereomer), 127.59 (2C, minor diastereomer), 127.58 (2C, minor diastereomer), 124.4 (major diastereomer), 123.7 (minor diastereomer), 114.9 (2C, minor diastereomer), 114.8 (2C, major diastereomer), 70.2 (2C, both diastereomers), 69.6 (major diastereomer), 69.3 (minor diastereomer), 37.56 (minor diastereomer), 37.53 (minor diastereomer), 34.89 (major diastereomer), 34.81 (major diastereomer), 34.6 (major diastereomer), 32.5 (minor diastereomer), 29.6 (major diastereomer), 27.43 (minor diastereomer); **HRMS** (TOF MS ES+) *m/z*: [M+Na] calcd for $C_{20}H_{24}O_4S$, 378.1739; found 378.1747.

IV. References for Supporting Information

[1] Pangborn, A. B.; Giardello, M. A.; Grubbs, R. H.; Rosen, R. K.; Timmers, F. J. Safe and Convenient Procedure for Solvent Purification. *Organometallics* **1996**, *15*, 1518–1520.
[2] Krasoviskiy, A.; Knochel, P. Convenient Titration Method for Organometallic Zinc, Magnesium, and Lanthanide Reagents. *Synthesis*, **2006**, *5,* 890–891.
[3] For the orginal report of the asymmetric dihydroxylation, see: Jacobsen, E. N.; Marko, I.; Mungall, W. S.; Schroeder, G.; Sharpless, K. B. Asymmetric Dihydroxylation via Ligand-Accelerated Catalysis. *J. Am. Chem. Soc.* **1988**, *110,* 1968–1970. For the procedure that was employed, see: Holl, K.; Schepmann, D.; Daniliuc, C. G.; Wünsch, B. Sharpless Asymmetric Dihydroxylation as the Key Step in the Enantioselective Synthesis of Spirocyclic σ1 Receptor Ligands. *Tetrahedron Asymmetry* **2014**, *25*, 268–277.
[4] Shang, Y.; Jie, X.; Jonnada, K.; Zafar, S. N.; Su, W. Dehydrogenative Desaturation-Relay via Formation of Multicenter-Stabilized Radical Intermediates. *Nat Commun*. **2017**, *8*, 2273.
[5] Note: if the aryl iodide starting material is a liquid, it was added via syringe after the flask was removed from the glovebox.
[6] Taber, D. F.; Guo, P. Convenient Access to Bicyclic and Tricyclic Diazenes. *J. Org. Chem.* **2008**, *73*, 9479–9481.
[7] Zhou, S.; Jia, Y. Total Synthesis of (–)-Goniomitine. *Org. Lett.* **2014**, *16*, 3416–3418.
[8] Stanley, L. M.; Hartwig, J. F. Regio- and Enantioselective *N*-Allylations of Imidazole, Benzimidazole, and Purine Heterocycles Catalyzed by Single-Component Metallacyclic Iridium Complexes. *J. Am. Chem. Soc.* **2009**, *131*, 8971–8983.
[9] Nicolaou, K. C.; Peng, X.-S.; Sun, Y.-P.; Polet, D.; Zou, B.; Lim, C. S.; Chen, D. Y.-K. Total Synthesis and Biological Evaluation of Cortistatins A and J and Analogues Thereof. *J. Am. Chem. Soc.* **2009**, *131*, 10587–10597.
[10] Note: We observed that the yield of the Parikh-Doering oxidation decreases with increasing scale. We typically performed this reaction on less than 5.0 mmol of primary alcohol.
[11] (a) For the preparation of the geminal dibromide, see: Wölfl, B.; Mata, G.; Fürstner, A. Total Synthesis of Callyspongiolide, Part 2: The Ynoate Matathesis/*cis*-Reduction Strategy. *Chem. Eur. J.* **2019**, *25*, 255–259. (b) For the preparation of the alkyne, see: Chandrasekhar, B.; Athe, S.; Reddy, P. P.; Ghosh, S. Synthesis of Fully Functionalized Aglycone of Lycoperdinoside A and B. *Org. Biomol. Chem.* **2015**, *13*, 115–124.
[12] Seel, S.; Thaler, T.; Takatsu, K.; Zhang, C.; Zipse, H.; Straub, B. F.; Mayer, P.; Knochel, P. Highly Diastereoselective Arylations of Substituted Piperidines. *J. Am. Chem. Soc.* **2011**, *133*, 4774–4777.

[13] Frost, C. C.; Hartley, B. C. Tandem Molybdenum Catalyzed Hydrosilylations: An Expedient Synthesis of β-Aryl Aldhydes. *Org. Lett.* **2007**, *9*, 4259–4261.
[14] Kandula, S. R. V.; Kumar, P. Asymmetric Synthesis of (–)-α-Conhydrine. *Tetrahedron Asymmetry* **2005**, *16*, 3268–3274.
[15] Huang, H.; Yu, C.; Zhang, Y.; Zhang, Y.; Mariano, P. S.; Wang, W. Chemo- and Regioselective Organo-Photoredox Catalyzed Hydroformylation of Styrenes via a Radical Pathway. *J. Am. Chem. Soc.* **2017**, *139*, 9799–9802.
[16] Kurosawa, W.; Kan, T.; Fukuyama, T. Stereocontrolled Total Synthesis of (–)-Ephedradine A (Orantine). *J. Am. Chem. Soc.* **2003**, *125*, 8112–8113.
[17] Lawrence, B. M. The Use of Silver Nitrate Impregnated Silica Gel Layers in the Separation of Monoterpene Hydrocarbons. *J. Chromatog.* **1968**, *38*, 535–537.
[18] Fujihara, T.; Cong, C.; Iwai, T.; Terao, T.; Tsuji, Y. Palladium-Catalyzed Reduction of Acid Chlorides to Aldehydes with Hydrosilanes. *Synlett* **2012**, *23*, 2389–2392.
[19] Datta, S.; Chang, C.-L.; Yeh, K.-L.; Liu, R.-S. A New Ruthenium-Catalyzed Cleavage of a Carbon-Carbo Triple Bond: Efficient Transformation of Ethynyl Alcohol into Alkene and Carbon Monoxide. *J. Am. Chem. Soc.* **2003**, *125*, 9294–9295.
[20] Isaacs, R. C. A.; Thompson, W. J.; Williams, P. D.; Su, D.-S.; Wenkatraman, S.; Embrey, M. W.; Fisher, T. E.; Wai, J. S.; Dubost, D. C.; Ball, R. C.; Choi, E. J.; Pei, T.; Trice, S. L.; Campbell, N.; Maddess, M.; Maligres, P. E.; Shevlin. M.; Song, Z. J.; Steinhuebel, D. P.; Strotman, N. A.; Yin, J. HIV Integrase Inhibitors. US 2010.0087419 A1, **2010**.
[21] Patel, H. H.; Sigman, M. S. *J. Am. Chem. Soc.* **2016**, *138*, 14226–14229.
[22] The silver impregnated silica gel was sensitive to light. All preparations of the silica gel were performed in the dark. The column was performed by wrapping the glassware in aluminum foil and in the dark.
[23] Szostak, M.; Spain, M.; Procter, D. J. Preparation of Samarium(II) IodideL Quantitative Evaluation of the Effect of Water, Oxygen, and Peroxide Content, Preparative Methods, and the Activation of Samarium Metal. *J. Org. Chem.* **2012**, *77*, 3049–3059.
[24] Dahlén, A.; Hilmersson, G. Microwave-Assisted Generation of Lanthanide(II) Halides in THF and Simple Quantitative Determination. *Eur. J. Inorg. Chem.* **2004**, *15*, 3020–3024.
[25] Wang, Y.; Jiang, M.; Liu, J.-T. Copper-Catalyzed Diastereoselective Synthesis of Trifluoromethylated Tetrahydrofurans. *Adv. Synth. Catal.* **2016**, *358*, 1322–1327.
[26] A – 20 ºC bath was prepared using ice water and NaCl.

Sequence: Sequence_khewitt1_ISQD_20201216
Injection #169: KAH-VI-060-crude

Chromatogram

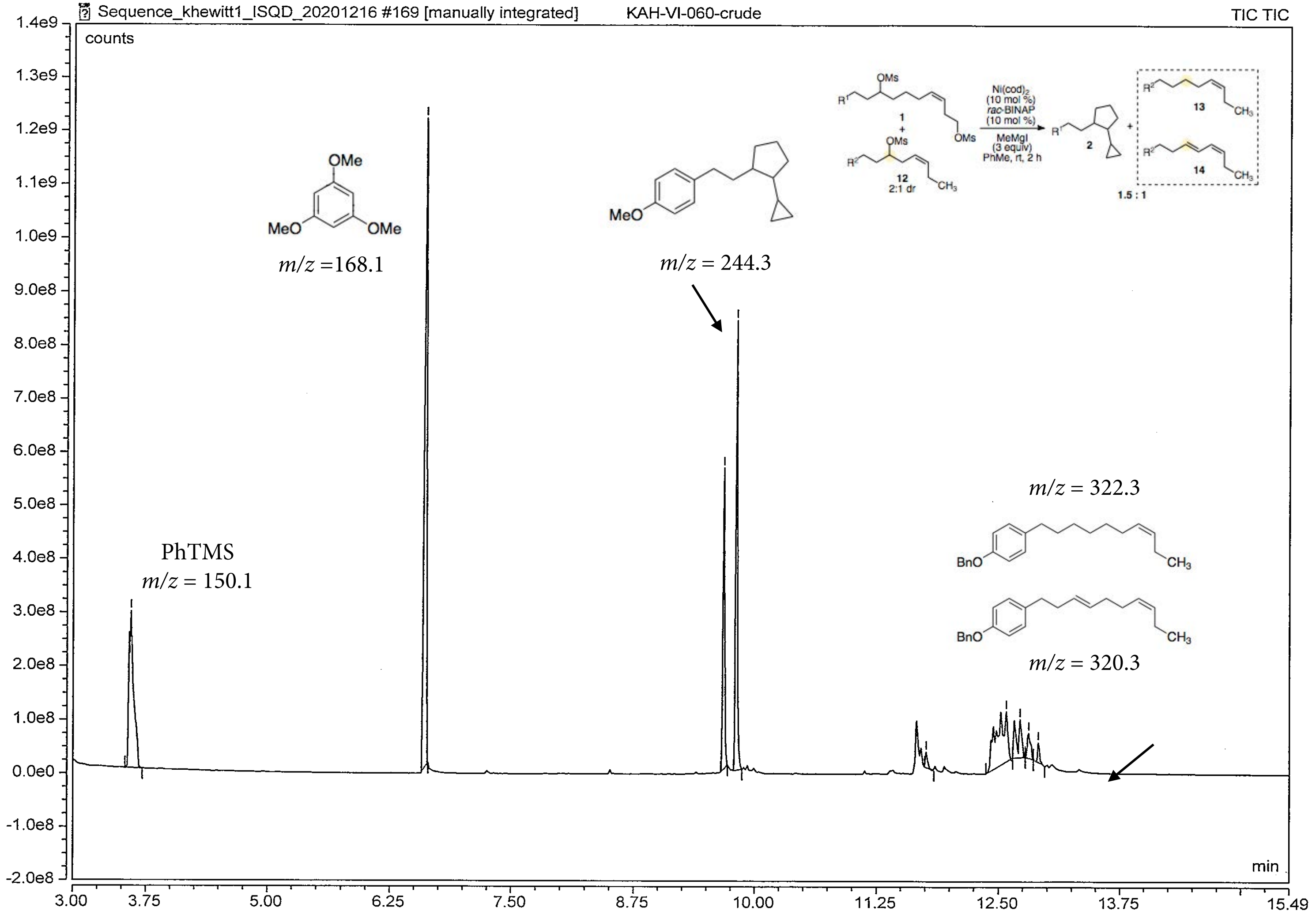

Chromeleon 7,
Version 7.2.9.11323, Thermo Fisher Scientific

Page 1 of 1

Printed by khewitt1
14/04/22 09:34

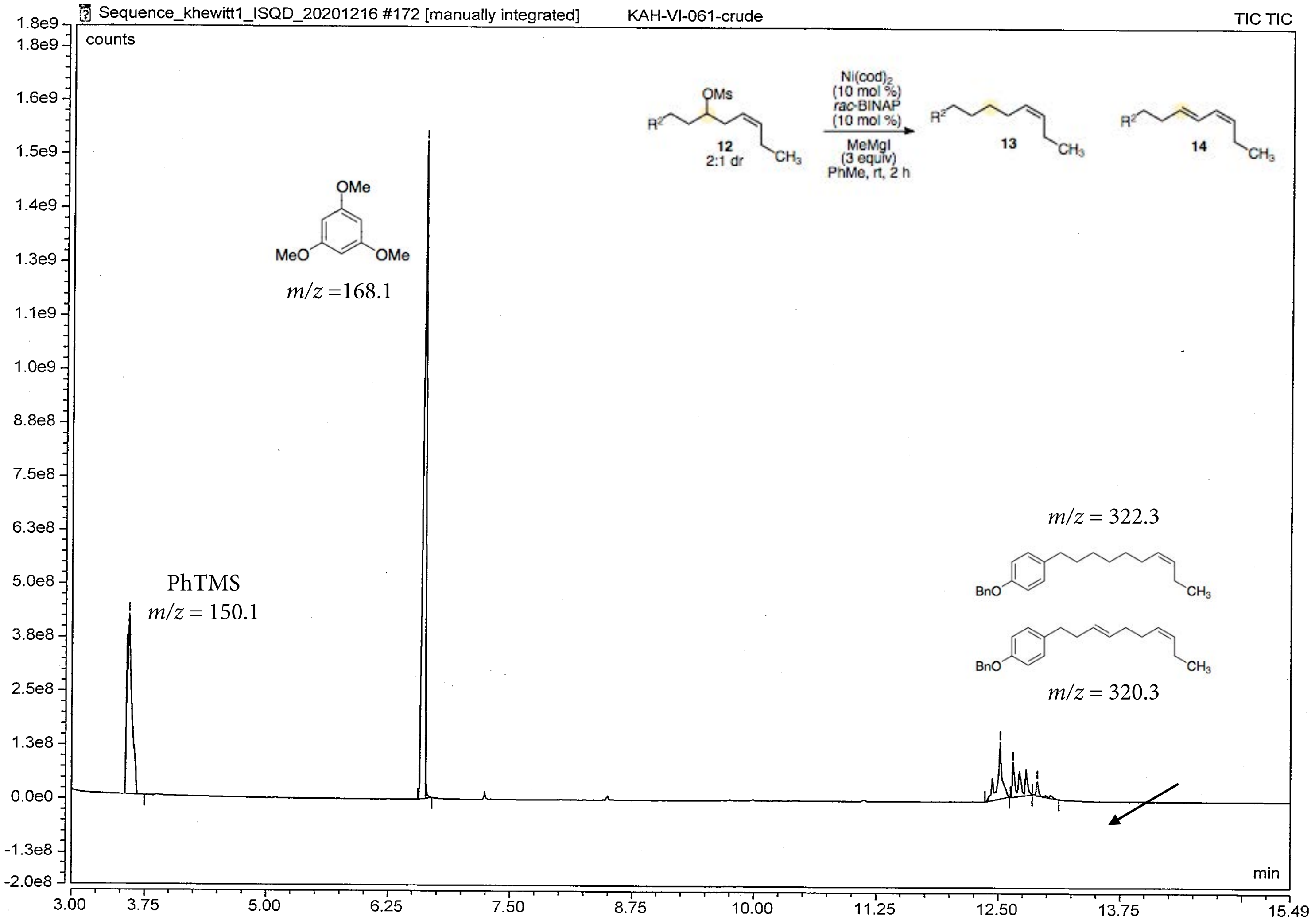
Sequence_khewitt1_ISQD_20201216 #172 [manually integrated]
KAH-VI-061-crude
TIC TIC
counts
Ni(cod)2 (10 mol %) rac-BINAP (10 mol %)
MeMgI (3 equiv) PhMe, rt, 2 h
12
2:1 dr
13
14
m/z =168.1
PhTMS
m/z = 150.1
m/z = 322.3
m/z = 320.3
min

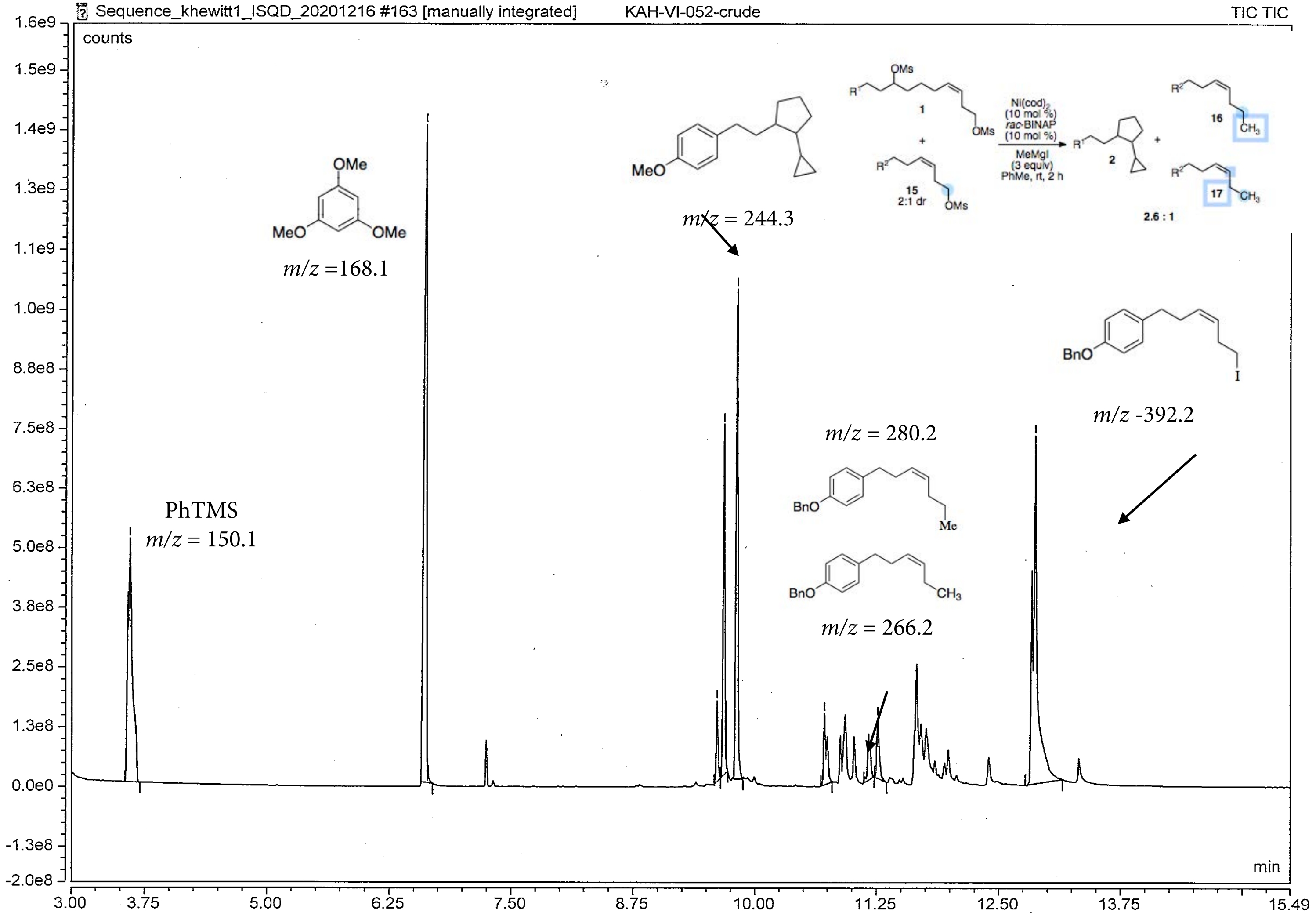
Sequence_khewitt1_ISQD_20201216 #163 [manually integrated]
KAH-VI-052-crude
TIC TIC
counts
PhTMS
m/z = 150.1
m/z =168.1
m/z = 244.3
m/z = 280.2
m/z = 266.2
m/z -392.2
min

Sequence: Sequence_khewitt1_ISQD_20201216
Injection #166: KAH-VI-057-crude

Chromatogram

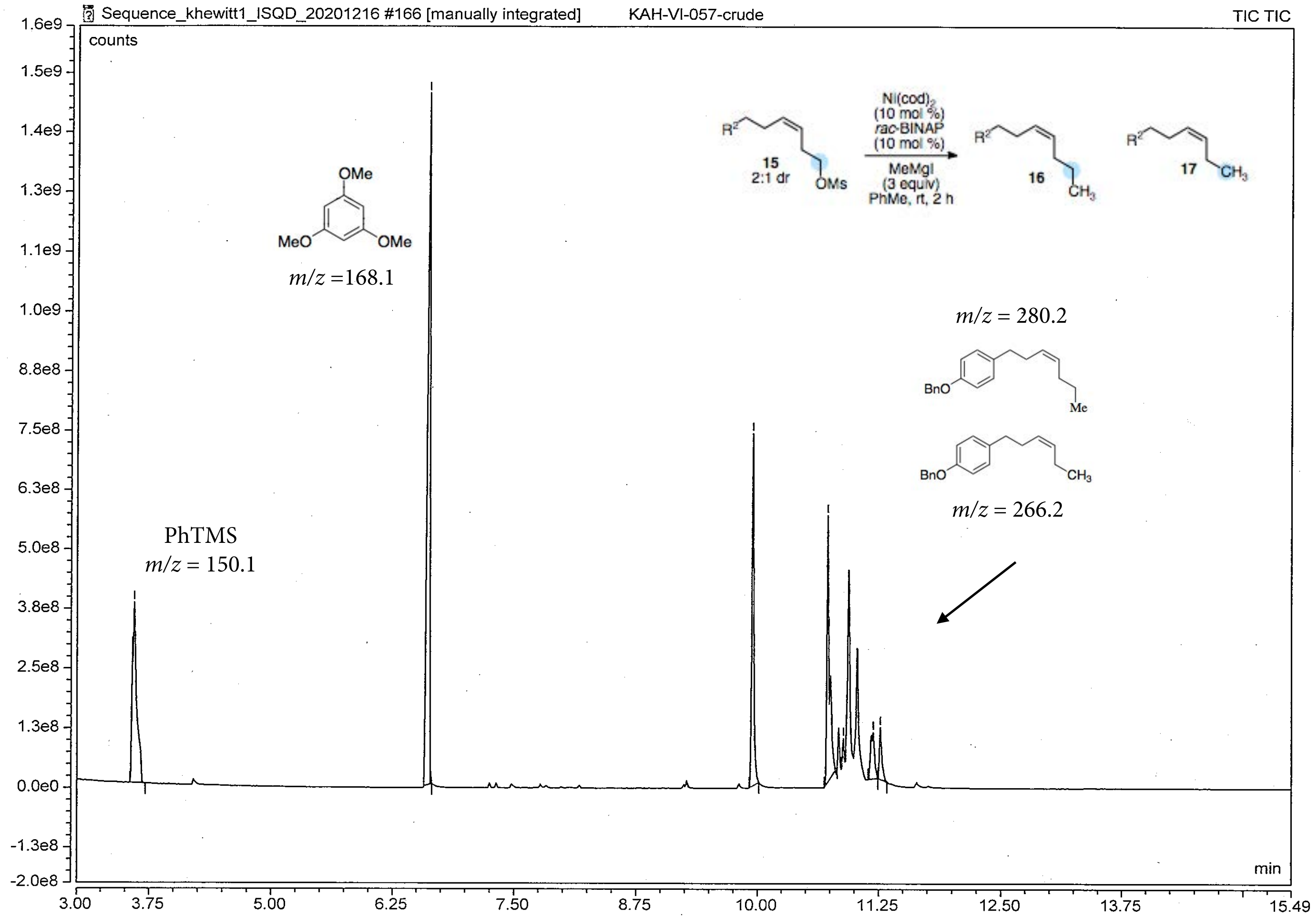

Chromeleon 7,
Version 7.2.9.11323, Thermo Fisher Scientific

Page 1 of 1

Printed by khewitt1
14/04/22 09:30

1H spectrum

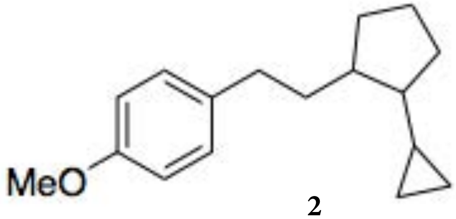

2

Unpurified Reaction Mixture

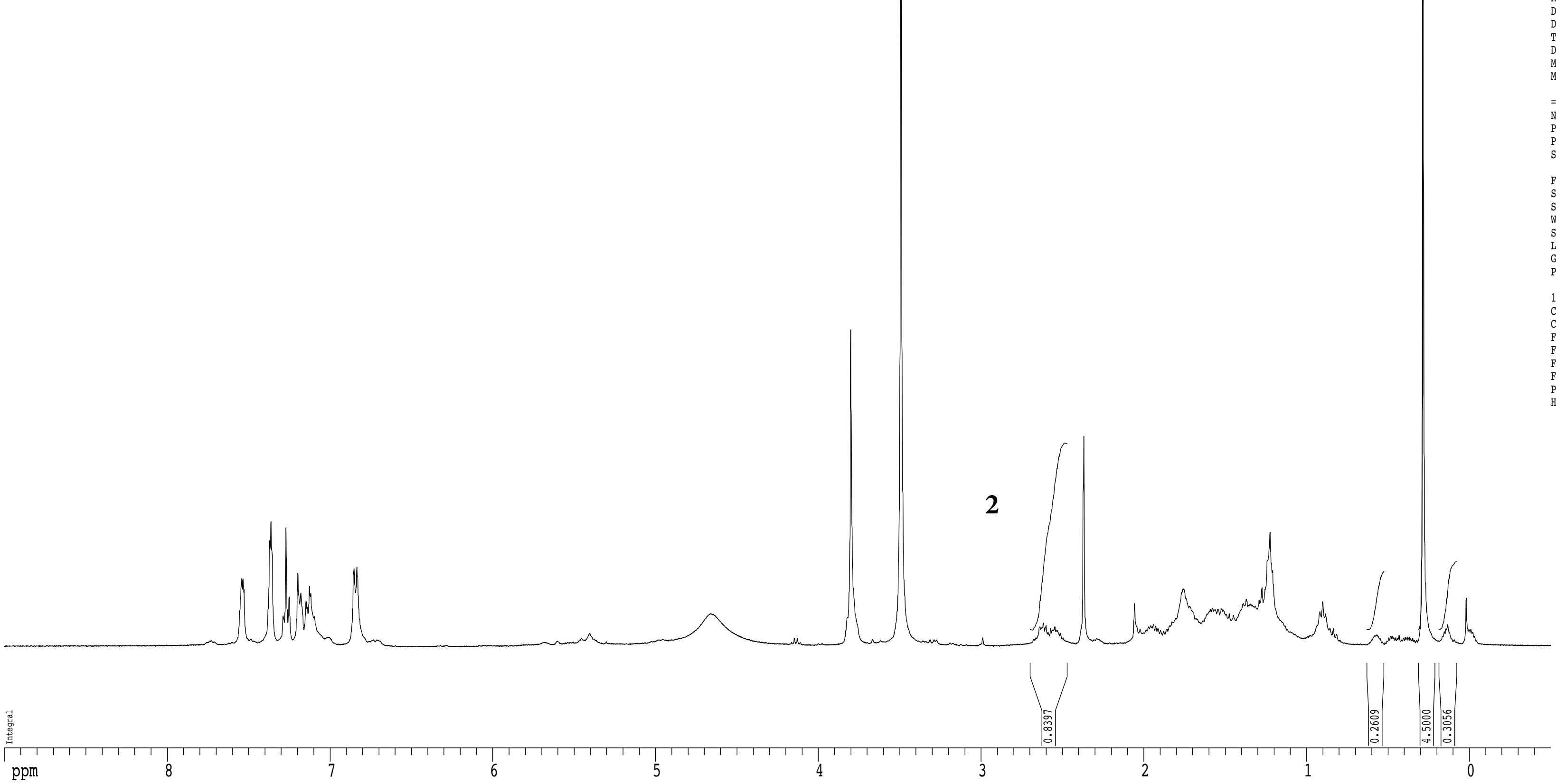


```
Current Data Parameters
USER            khewitt1
NAME     KAH-III-064 crude
EXPNO                  1
PROCNO                 1

F2 - Acquisition Parameters
Date_           20200129
Time               14.31
INSTRUM           drx400
PROBHD   5 mm QNP H/F/P
PULPROG             zg30
TD                 38460
SOLVENT           CDCl3T
NS                     8
DS                     2
SWH             6410.256 Hz
FIDRES          0.166673 Hz
AQ             2.9999299 sec
RG                  90.5
DW                78.000 usec
DE                  4.50 usec
TE                 298.1 K
D1            0.10000000 sec
MCREST        0.00000000 sec
MCWRK         0.01500000 sec

======== CHANNEL f1 ========
NUC1                  1H
P1                 12.00 usec
PL1                -1.10 dB
SFO1         400.1328009 MHz

F2 - Processing parameters
SI                 65536
SF           400.1300175 MHz
WDW                   no
SSB                    0
LB                  0.00 Hz
GB                     0
PC                  2.00

1D NMR plot parameters
CX                 22.80 cm
CY                 15.00 cm
F1P                9.000 ppm
F1               3601.17 Hz
F2P               -0.500 ppm
F2               -200.06 Hz
PPMCM            0.41667 ppm/cm
HZCM           166.72084 Hz/cm
```

1H spectrum

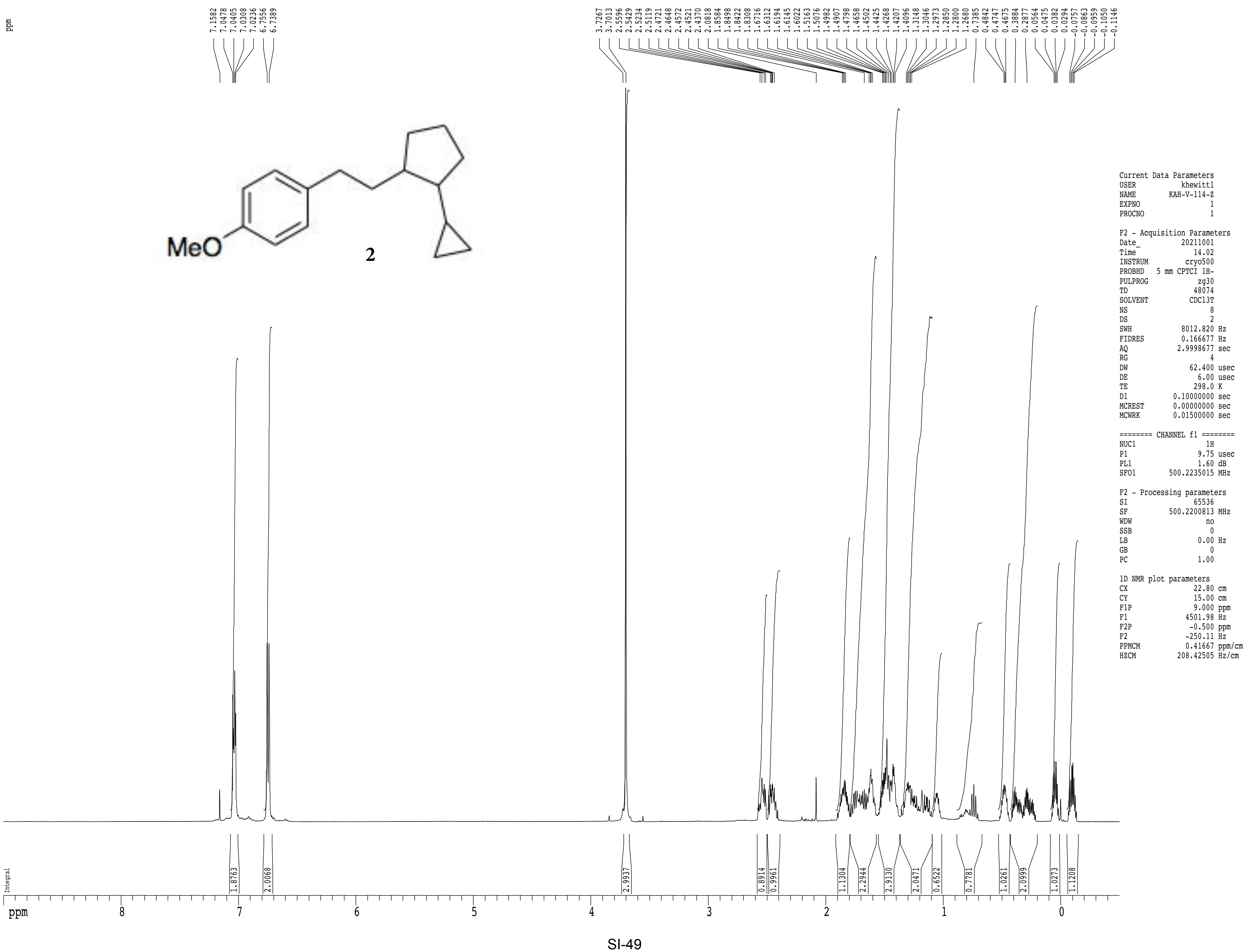
ppm
7.1582
7.0478
7.0405
7.0308
7.0236
6.7556
6.7389
3.7267
3.7013
2.5596
2.5429
2.5234
2.5119
2.4721
2.4648
2.4572
2.4521
2.4370
2.0818
1.8584
1.8498
1.8422
1.8308
1.6716
1.6312
1.6194
1.6145
1.6022
1.5163
1.5076
1.4982
1.4907
1.4798
1.4658
1.4502
1.4425
1.4268
1.4207
1.4096
1.3148
1.3046
1.2973
1.2850
1.2800
1.2680
0.7385
0.4842
0.4747
0.4675
0.3884
0.2877
0.0564
0.0475
0.0382
0.0294
-0.0757
-0.0863
-0.0959
-0.1050
-0.1146
MeO
2
Current Data Parameters
USER khewitt1
NAME KAH-V-114-Z
EXPNO 1
PROCNO 1
F2 - Acquisition Parameters
Date_ 20211001
Time 14.02
INSTRUM cryo500
PROBHD 5 mm CPTCI 1H-
PULPROG zg30
TD 48074
SOLVENT CDCl3T
NS 8
DS 2
SWH 8012.820 Hz
FIDRES 0.166677 Hz
AQ 2.9998677 sec
RG 4
DW 62.400 usec
DE 6.00 usec
TE 298.0 K
D1 0.10000000 sec
MCREST 0.00000000 sec
MCWRK 0.01500000 sec
======== CHANNEL f1 ========
NUC1 1H
P1 9.75 usec
PL1 1.60 dB
SFO1 500.2235015 MHz
F2 - Processing parameters
SI 65536
SF 500.2200813 MHz
WDW no
SSB 0
LB 0.00 Hz
GB 0
PC 1.00
1D NMR plot parameters
CX 22.80 cm
CY 15.00 cm
F1P 9.000 ppm
F1 4501.98 Hz
F2P -0.500 ppm
F2 -250.11 Hz
PPMCM 0.41667 ppm/cm
HZCM 208.42505 Hz/cm
Integral
1.8763
2.0068
2.9937
0.8914
0.9961
1.1304
2.2944
2.9130
2.0471
0.6522
0.7781
1.0261
2.0999
1.0273
1.1208
ppm
8
7
6
5
4
3
2
1
0

13C spectrum with 1H decoupling

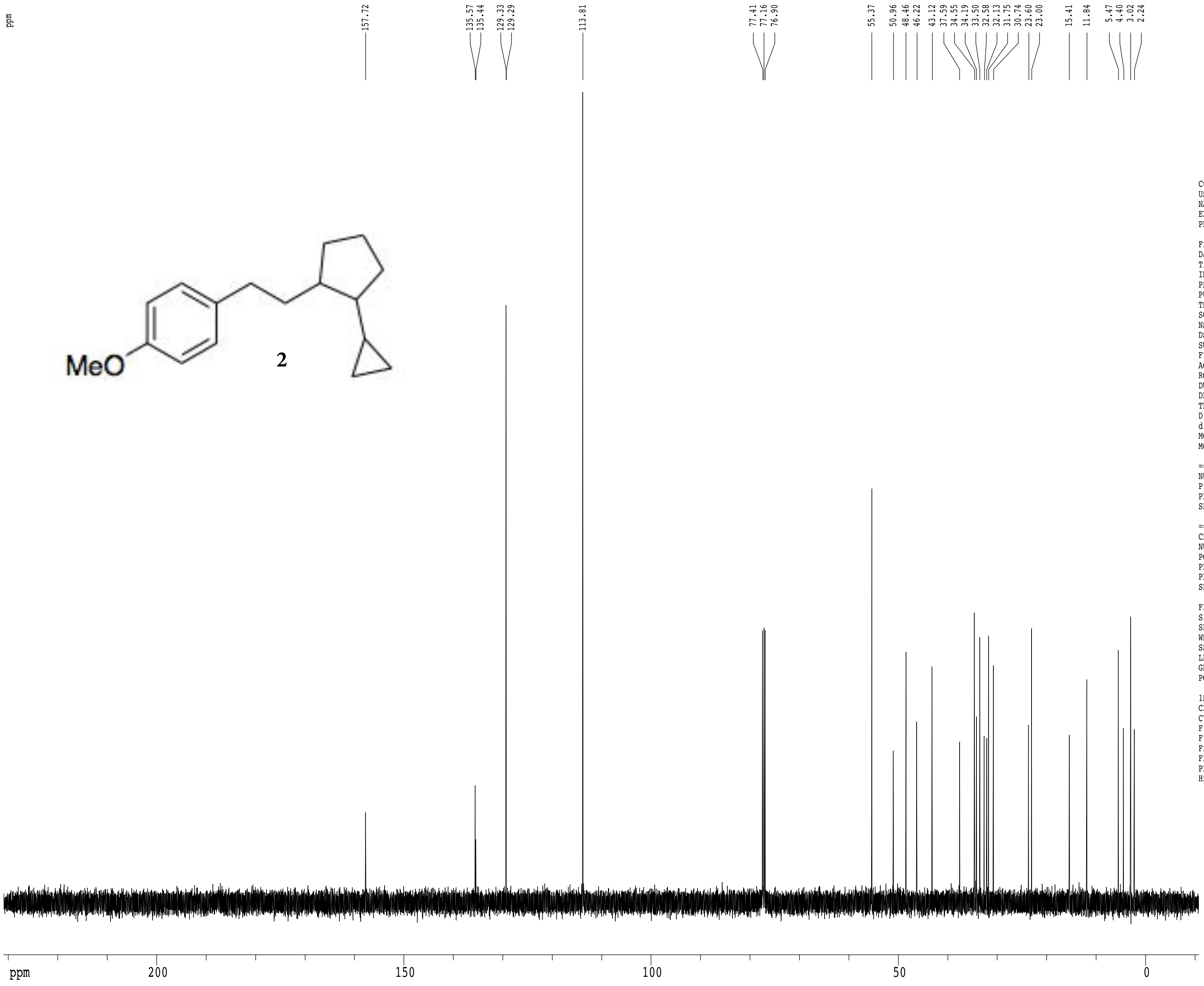

2


```
Current Data Parameters
USER          khewitt1
NAME     KAH-V-114-115-chk
EXPNO                2
PROCNO               1

F2 - Acquisition Parameters
Date_          20210929
Time              16.34
INSTRUM           gn500
PROBHD   5 mm broadband
PULPROG         zgdc30
TD                65536
SOLVENT           CDCl3
NS                  120
DS                    4
SWH           30303.031 Hz
FIDRES         0.462388 Hz
AQ            1.0813940 sec
RG                46341
DW               16.500 usec
DE                 6.00 usec
TE                298.0 K
D1           0.25000000 sec
d11          0.03000000 sec
MCREST       0.00000000 sec
MCWRK        0.01500000 sec

======== CHANNEL f1 ========
NUC1                13C
P1                14.20 usec
PL1               -6.00 dB
SFO1        125.3994349 MHz

======== CHANNEL f2 ========
CPDPRG2         waltz16
NUC2                 1H
PCPD2            100.00 usec
PL2               -6.00 dB
PL12              12.30 dB
SFO2        498.6524933 MHz

F2 - Processing parameters
SI                65536
SF          125.3856329 MHz
WDW                  no
SSB                   0
LB                 0.00 Hz
GB                    0
PC                 2.00

1D NMR plot parameters
CX                22.80 cm
CY                15.65 cm
F1P             230.956 ppm
F1             28958.52 Hz
F2P             -10.723 ppm
F2             -1344.51 Hz
PPMCM          10.59994 ppm/cm
HZCM         1329.08032 Hz/cm
```

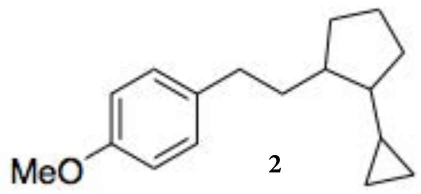

2

gcosy60

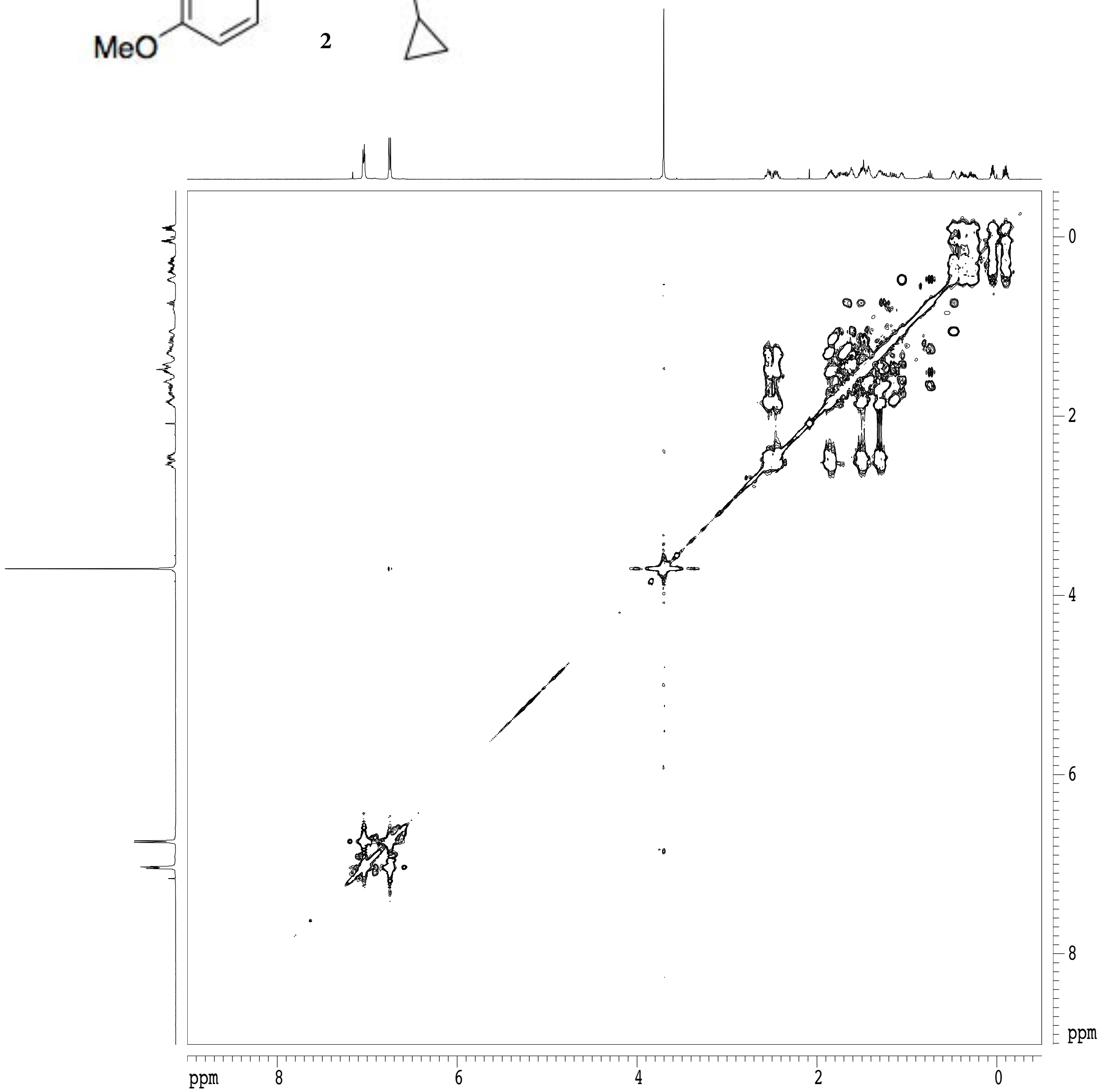

Current Data Parameters
USER khewitt1
NAME KAH-V-114-Z
EXPNO 2
PROCNO 1

F2 - Acquisition Parameters
Date_ 20211001
Time 14.04
INSTRUM cryo500
PROBHD 5 mm CPTCI 1H-
PULPROG cosygp60.prd
TD 2048
SOLVENT CDCl3
NS 1
DS 16
SWH 8012.820 Hz
FIDRES 3.912510 Hz
AQ 0.1278452 sec
RG 35.9
DW 62.400 usec
DE 6.00 usec
TE 298.0 K
d0 0.00000300 sec
D1 1.00000000 sec
d13 0.00000300 sec
D16 0.00020000 sec
IN0 0.00012480 sec

======== CHANNEL f1 ========
NUC1 1H
P1 9.75 usec
PL1 1.60 dB
SFO1 500.2235015 MHz

====== GRADIENT CHANNEL =====
GPNAM1 SMSQ10.100
GPNAM2 SMSQ10.100
GPX1 0.00 %
GPX2 0.00 %
GPY1 0.00 %
GPY2 0.00 %
GPZ1 17.00 %
GPZ2 17.00 %
P16 1000.00 usec

F1 - Acquisition parameters
ND0 1
TD 512
SFO1 500.2235 MHz
FIDRES 15.650040 Hz
SW 16.018 ppm
FnMODE QF

F2 - Processing parameters
SI 1024
SF 500.2200813 MHz
WDW SINE
SSB 0
LB 0.00 Hz
GB 0
PC 1.00

F1 - Processing parameters
SI 1024
MC2 QF
SF 500.2200813 MHz
WDW SINE
SSB 0
LB 0.00 Hz
GB 0

2D NMR plot parameters
CX2 15.00 cm
CX1 15.00 cm
F2PLO 9.000 ppm
F2LO 4501.98 Hz
F2PHI -0.500 ppm
F2HI -250.11 Hz
F1PLO 9.012 ppm
F1LO 4507.95 Hz
F1PHI -0.515 ppm
F1HI -257.49 Hz
F2PPMCM 0.63333 ppm/cm
F2HZCM 316.80606 Hz/cm
F1PPMCM 0.63511 ppm/cm
F1HZCM 317.69580 Hz/cm

ghmqc

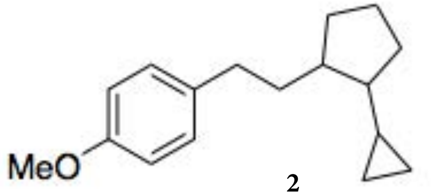

2

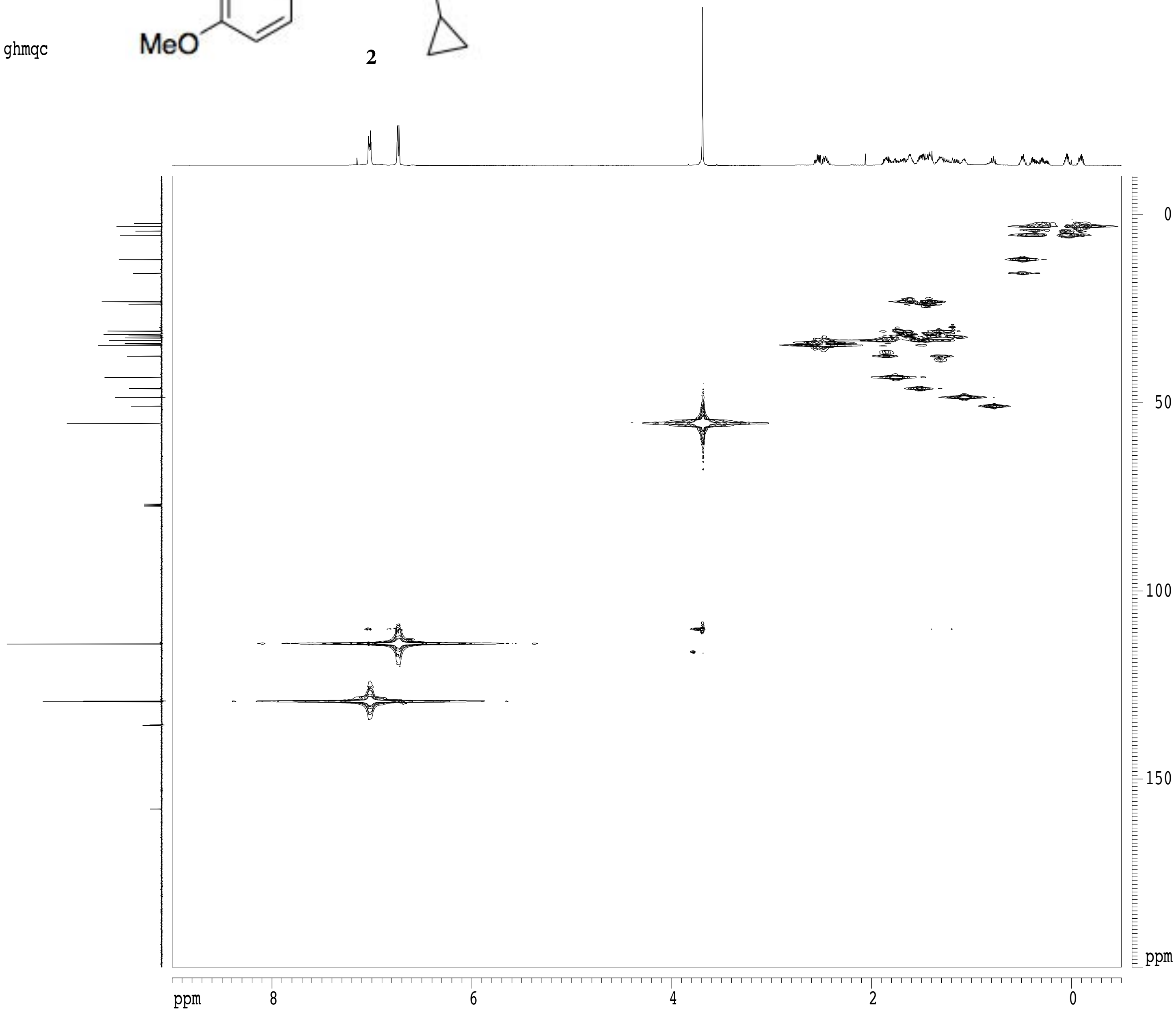


```
Current Data Parameters
USER          khewitt1
NAME        KAH-V-114-Z
EXPNO                 6
PROCNO                1

F2 - Acquisition Parameters
Date_          20211004
Time              17.40
INSTRUM         cryo500
PROBHD   5 mm CPTCI 1H-
PULPROG       inv4gp.wu
TD                 2048
SOLVENT           CDCl3
NS                    1
DS                   16
SWH            8012.820 Hz
FIDRES         3.912510 Hz
AQ            0.1278452 sec
RG              11585.2
DW               62.400 usec
DE                 6.50 usec
TE                323.0 K
CNST2       145.0000000
d0           0.00000300 sec
D1           1.00000000 sec
d2           0.00344828 sec
d12          0.00002000 sec
d13          0.00000300 sec
D16          0.00020000 sec
d20          0.00242528 sec
IN0          0.00001650 sec

======== CHANNEL f1 ========
NUC1                 1H
P1                 9.75 usec
p2                19.50 usec
PL1                1.60 dB
SFO1        500.2235015 MHz

======== CHANNEL f2 ========
CPDPRG2            garp
NUC2                13C
P3                18.85 usec
PCPD2             70.00 usec
PL2               -1.00 dB
PL12              10.40 dB
SFO2        125.7942548 MHz

====== GRADIENT CHANNEL =====
GPNAM1       SMSQ10.100
GPNAM2       SMSQ10.100
GPNAM3       SMSQ10.100
GPX1               0.00 %
GPX2               0.00 %
GPX3               0.00 %
GPY1               0.00 %
GPY2               0.00 %
GPY3               0.00 %
GPZ1              30.00 %
GPZ2              18.00 %
GPZ3              24.00 %
P16             1000.00 usec

F1 - Acquisition parameters
ND0                   2
TD                  512
SFO1           125.7943 MHz
FIDRES        59.185608 Hz
SW              240.894 ppm
FnMODE               QF

F2 - Processing parameters
SI                 1024
SF          500.2200860 MHz
WDW                  EM
SSB                   0
LB                 5.00 Hz
GB                    0
PC                 2.00

F1 - Processing parameters
SI                 1024
MC2                  QF
SF          125.7804002 MHz
WDW               QSINE
SSB                   3
LB                 0.00 Hz
GB                    0

2D NMR plot parameters
CX2               18.00 cm
CX1               15.00 cm
F2PLO             9.000 ppm
F2LO            4501.98 Hz
F2PHI            -0.500 ppm
F2HI            -250.11 Hz
F1PLO           200.000 ppm
F1LO           25156.08 Hz
F1PHI           -10.310 ppm
F1HI           -1296.84 Hz
F2PPMCM         0.52778 ppm/cm
F2HZCM        264.00504 Hz/cm
F1PPMCM        14.02069 ppm/cm
F1HZCM       1763.52795 Hz/cm
```

1H spectrum

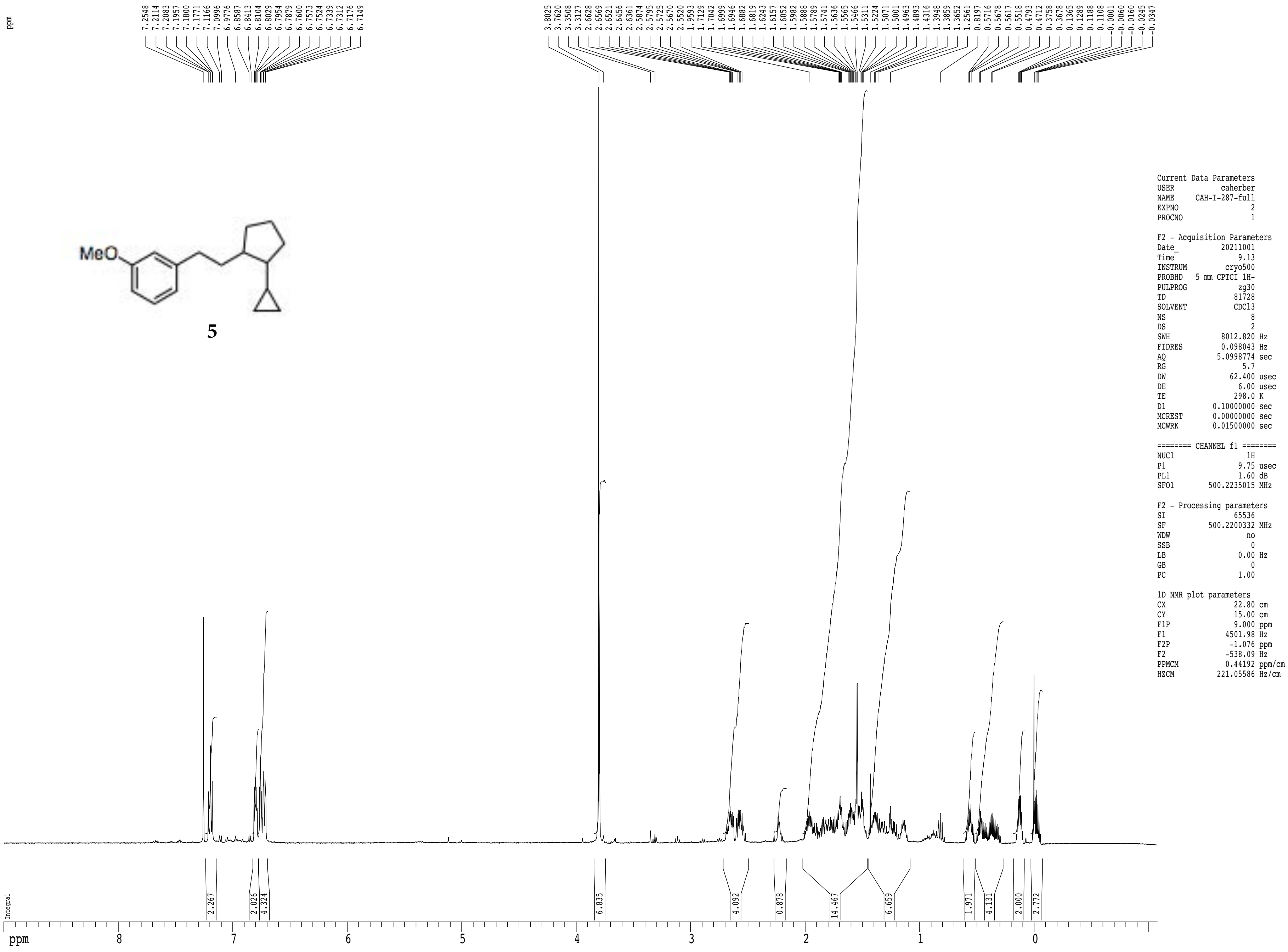

5

Z-restored spin-echo 13C spectrum with 1H decoupling

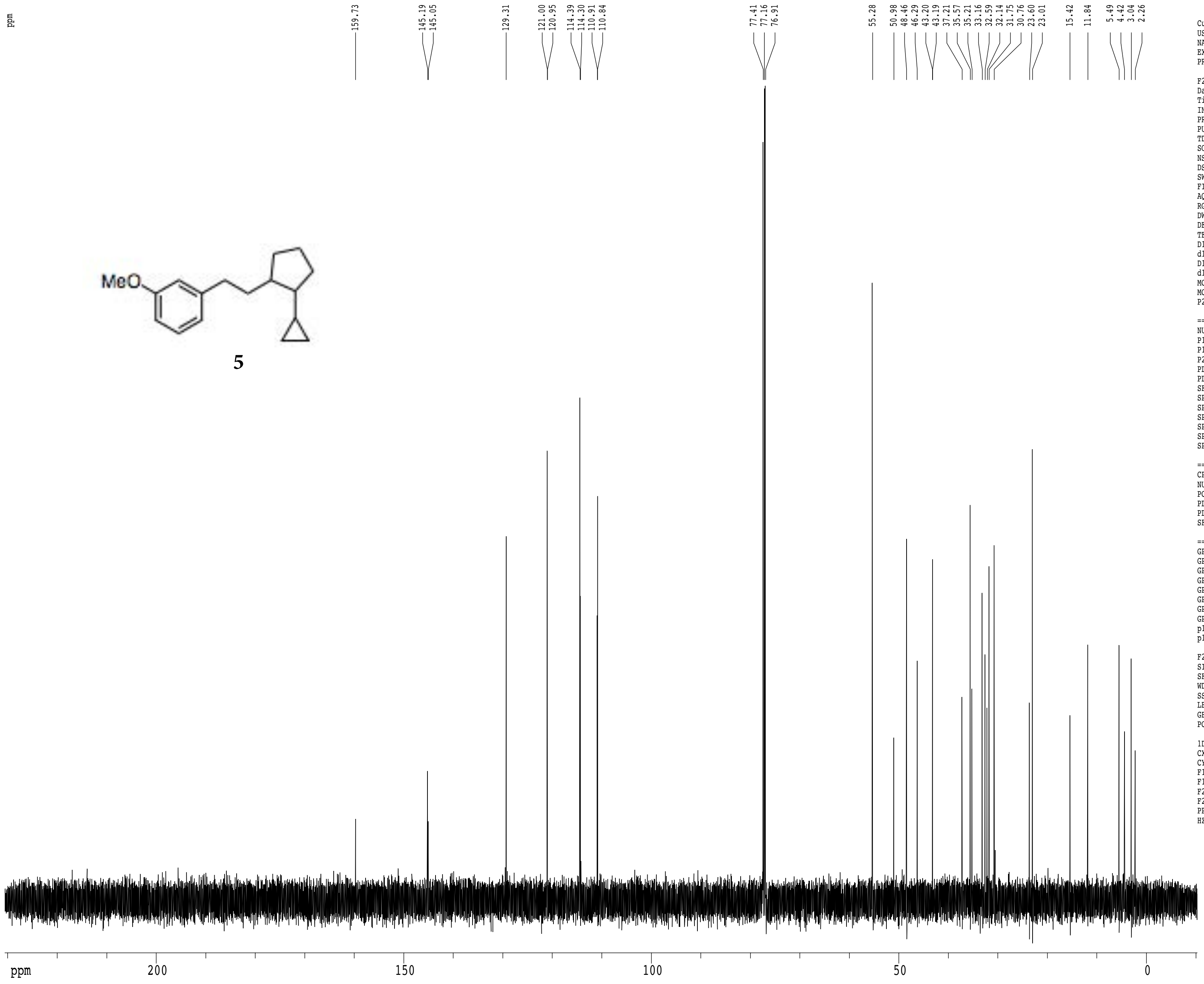


```
Current Data Parameters
USER          caherber
NAME     CAH-I-287-full
EXPNO                 3
PROCNO                1

F2 - Acquisition Parameters
Date_          20211001
Time               9.18
INSTRUM         cryo500
PROBHD   5 mm CPTCI 1H-
PULPROG  SpinEchopg30gp2.prd
TD                65536
SOLVENT           CDCl3
NS                  232
DS                   16
SWH           30303.031 Hz
FIDRES         0.462388 Hz
AQ            1.0813940 sec
RG                 2048
DW               16.500 usec
DE                 6.00 usec
TE                298.0 K
D1           0.25000000 sec
d11          0.03000000 sec
D16          0.00020000 sec
d17          0.00019600 sec
MCREST       0.00000000 sec
MCWRK        0.01500000 sec
P2                37.70 usec

======== CHANNEL f1 ========
NUC1                13C
P1                18.85 usec
P12             2000.00 usec
P20              500.00 usec
PL0              120.00 dB
PL1               -1.00 dB
SFO1        125.7942548 MHz
SP2                1.55 dB
SP4                1.55 dB
SPNAM2      Crp60comp.4
SPNAM4   Crp60,0.5,20.1
SPOFF2             0.00 Hz
SPOFF4             0.00 Hz

======== CHANNEL f2 ========
CPDPRG2         waltz16
NUC2                 1H
PCPD2            100.00 usec
PL2                1.60 dB
PL12              22.00 dB
SFO2        500.2225011 MHz

====== GRADIENT CHANNEL =====
GPNAM1         SINE.100
GPNAM2         SINE.100
GPX1               0.00 %
GPX2               0.00 %
GPY1               0.00 %
GPY2               0.00 %
GPZ1              30.00 %
GPZ2              50.00 %
p15              500.00 usec
p16             1000.00 usec

F2 - Processing parameters
SI                65536
SF          125.7804062 MHz
WDW                  no
SSB                   0
LB                 0.00 Hz
GB                    0
PC                 2.00

1D NMR plot parameters
CX                22.80 cm
CY                15.65 cm
F1P             230.637 ppm
F1             29009.68 Hz
F2P             -10.287 ppm
F2             -1293.96 Hz
PPMCM          10.56688 ppm/cm
HZCM         1329.10693 Hz/cm
```

1H spectrum

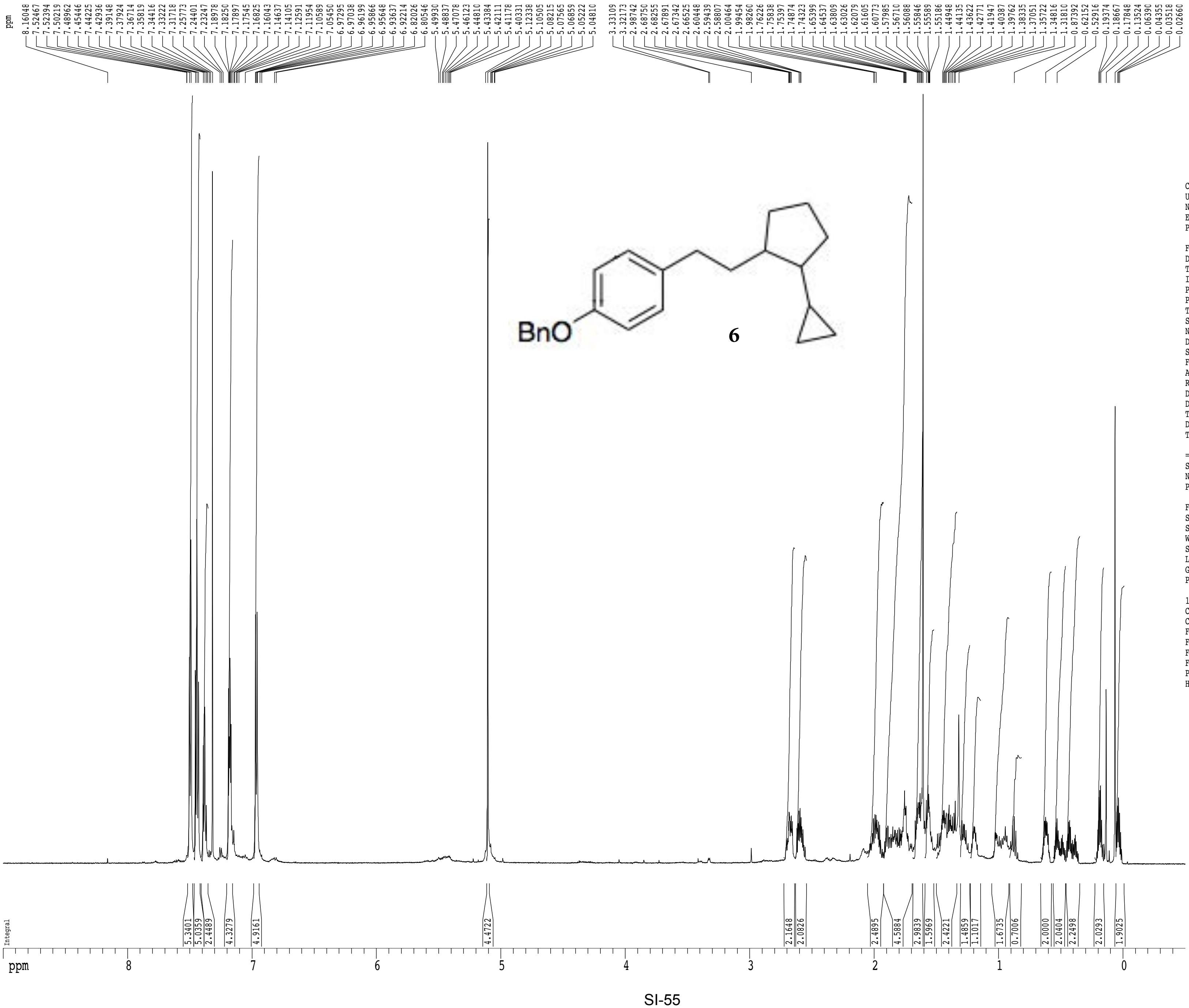


```
Current Data Parameters
USER          khewitt1
NAME     KAH-V-304-z-600
EXPNO                  3
PROCNO                 1

F2 - Acquisition Parameters
Date_           20220313
Time               16.50
INSTRUM            av600
PROBHD   5 mm CPBBO BB-
PULPROG             zg30
TD                 98074
SOLVENT           CDCl3T
NS                    32
DS                     2
SWH             9615.385 Hz
FIDRES          0.098042 Hz
AQ             5.0998979 sec
RG                    10
DW                52.000 usec
DE                 14.23 usec
TE                 298.0 K
D1            0.10000000 sec
TD0                    1

======== CHANNEL f1 ========
SFO1         600.1342009 MHz
NUC1                  1H
P1                  9.50 usec

F2 - Processing parameters
SI                 65536
SF           600.1300000 MHz
WDW                   no
SSB                    0
LB                  0.00 Hz
GB                     0
PC                  1.00

1D NMR plot parameters
CX                 22.80 cm
CY                 15.00 cm
F1P                9.000 ppm
F1               5401.17 Hz
F2P               -0.500 ppm
F2               -300.06 Hz
PPMCM            0.41667 ppm/cm
HZCM           250.05418 Hz/cm
```

13C spectrum

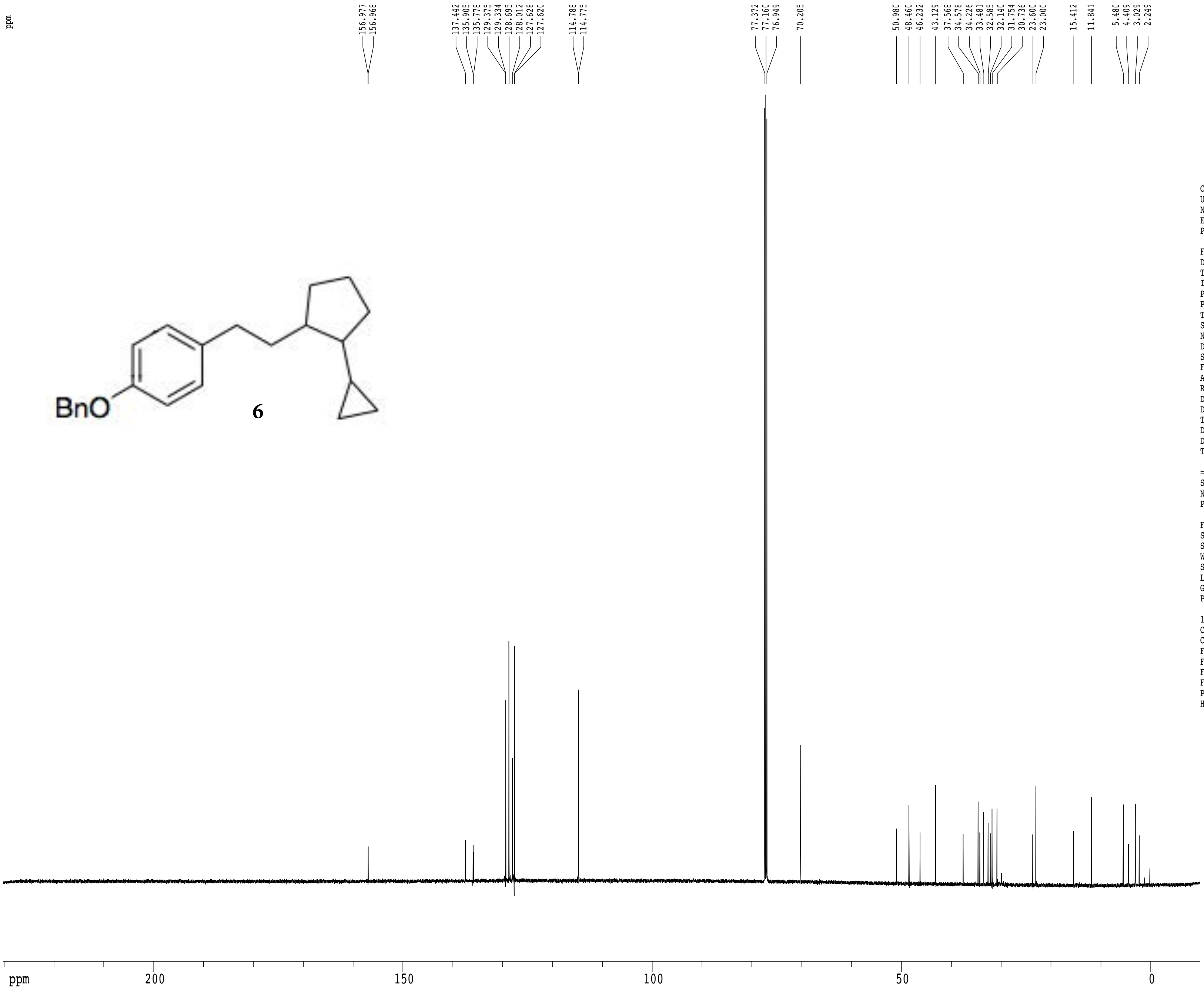

Current Data Parameters
USER khewitt1
NAME KAH-V-304-z-600
EXPNO 4
PROCNO 1

F2 - Acquisition Parameters
Date_ 20220313
Time 16.56
INSTRUM av600
PROBHD 5 mm CPBBO BB-
PULPROG zgdc30
TD 65536
SOLVENT CDCl3T
NS 814
DS 4
SWH 36231.883 Hz
FIDRES 0.552855 Hz
AQ 0.9044468 sec
RG 2050
DW 13.800 usec
DE 19.63 usec
TE 297.9 K
D1 0.40000001 sec
D11 0.03000000 sec
TD0 1

======== CHANNEL f1 ========
SFO1 150.9194080 MHz
NUC1 13C
P1 10.10 usec

F2 - Processing parameters
SI 65536
SF 150.9027942 MHz
WDW no
SSB 0
LB 0.00 Hz
GB 0
PC 1.00

1D NMR plot parameters
CX 22.80 cm
CY 15.00 cm
F1P 230.147 ppm
F1 34729.77 Hz
F2P -9.954 ppm
F2 -1502.12 Hz
PPMCM 10.53074 ppm/cm
HZCM 1589.11780 Hz/cm

1H spectrum

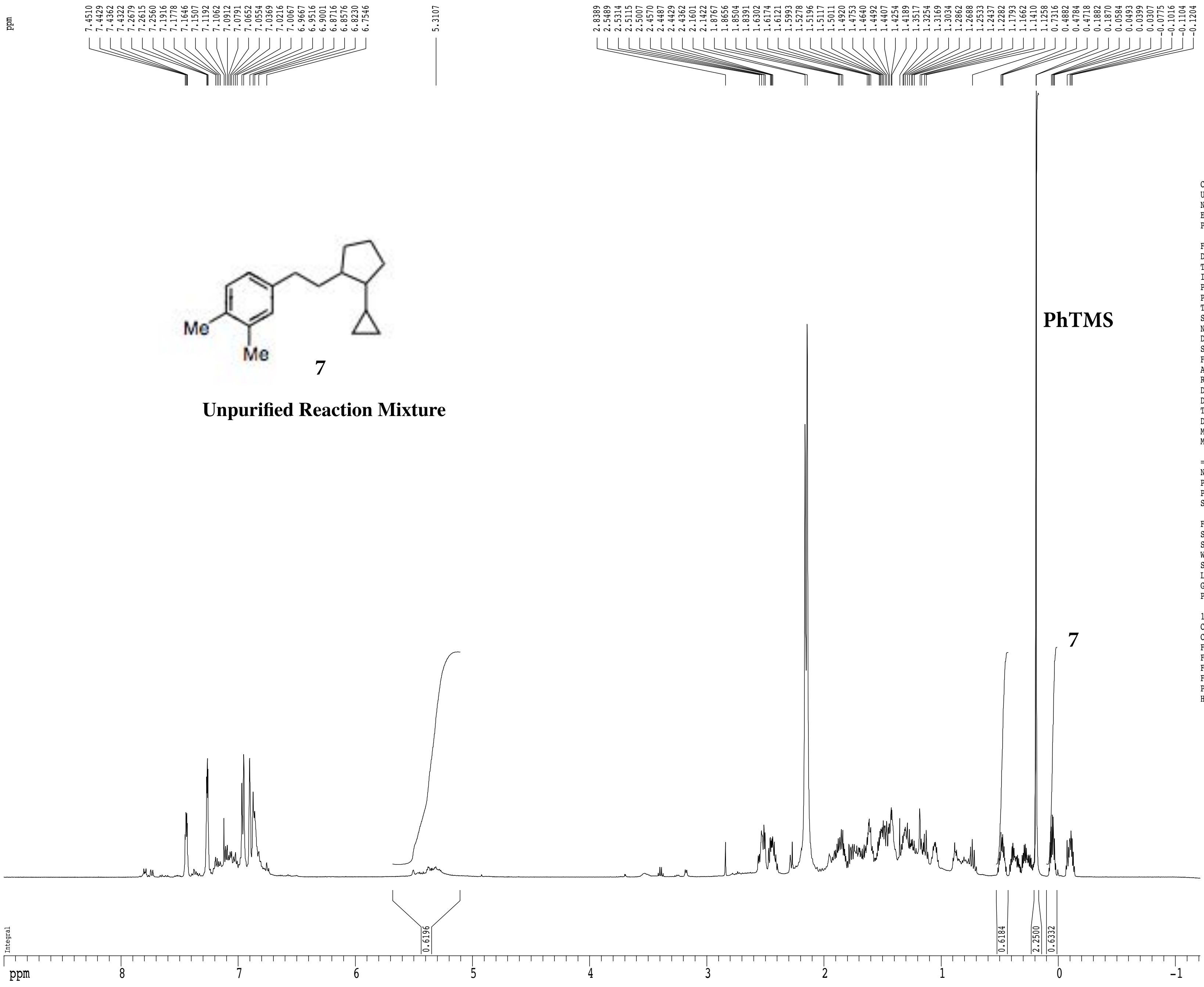


```
Current Data Parameters
USER          caherber
NAME     CAH-II-029-Crude
EXPNO                1
PROCNO               1

F2 - Acquisition Parameters
Date_          20211111
Time              17.12
INSTRUM         cryo500
PROBHD   5 mm CPTCI 1H-
PULPROG            zg30
TD                81728
SOLVENT           CDCl3
NS                    8
DS                    2
SWH            8012.820 Hz
FIDRES         0.098043 Hz
AQ            5.0998774 sec
RG                  3.6
DW               62.400 usec
DE                 6.00 usec
TE                298.0 K
D1           0.10000000 sec
MCREST       0.00000000 sec
MCWRK        0.01500000 sec

======== CHANNEL f1 ========
NUC1                 1H
P1                 9.75 usec
PL1                1.60 dB
SFO1        500.2235015 MHz

F2 - Processing parameters
SI                65536
SF          500.2201016 MHz
WDW                  no
SSB                   0
LB                 0.00 Hz
GB                    0
PC                 1.00

1D NMR plot parameters
CX                22.80 cm
CY                15.00 cm
F1P               9.000 ppm
F1              4501.98 Hz
F2P              -1.212 ppm
F2              -606.44 Hz
PPMCM           0.44791 ppm/cm
HZCM          224.05360 Hz/cm
```

1H spectrum

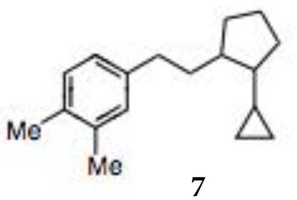

7

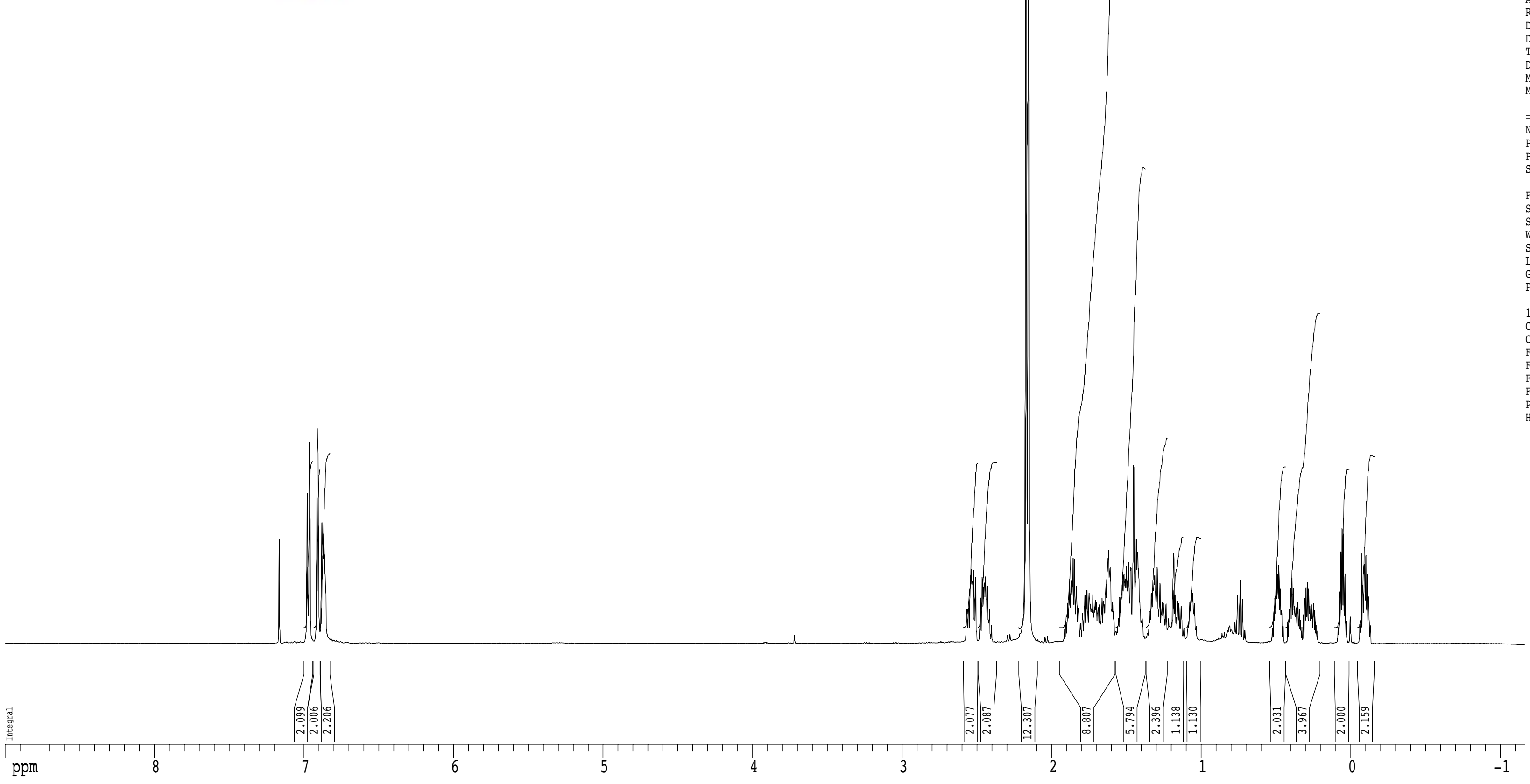

Current Data Parameters
USER caherber
NAME CAH-II-029-full
EXPNO 1
PROCNO 1

F2 - Acquisition Parameters
Date_ 20211113
Time 12.39
INSTRUM cryo500
PROBHD 5 mm CPTCI 1H-
PULPROG zg30
TD 81728
SOLVENT CDCl3
NS 8
DS 2
SWH 8012.820 Hz
FIDRES 0.098043 Hz
AQ 5.0998774 sec
RG 6.3
DW 62.400 usec
DE 6.00 usec
TE 298.0 K
D1 0.10000000 sec
MCREST 0.00000000 sec
MCWRK 0.01500000 sec

======== CHANNEL f1 ========
NUC1 1H
P1 9.75 usec
PL1 1.60 dB
SFO1 500.2235015 MHz

F2 - Processing parameters
SI 65536
SF 500.2200792 MHz
WDW no
SSB 0
LB 0.00 Hz
GB 0
PC 1.00

1D NMR plot parameters
CX 22.80 cm
CY 15.00 cm
F1P 9.000 ppm
F1 4501.98 Hz
F2P -1.168 ppm
F2 -584.06 Hz
PPMCM 0.44595 ppm/cm
HZCM 223.07208 Hz/cm

Z-restored spin-echo 13C spectrum with 1H decoupling

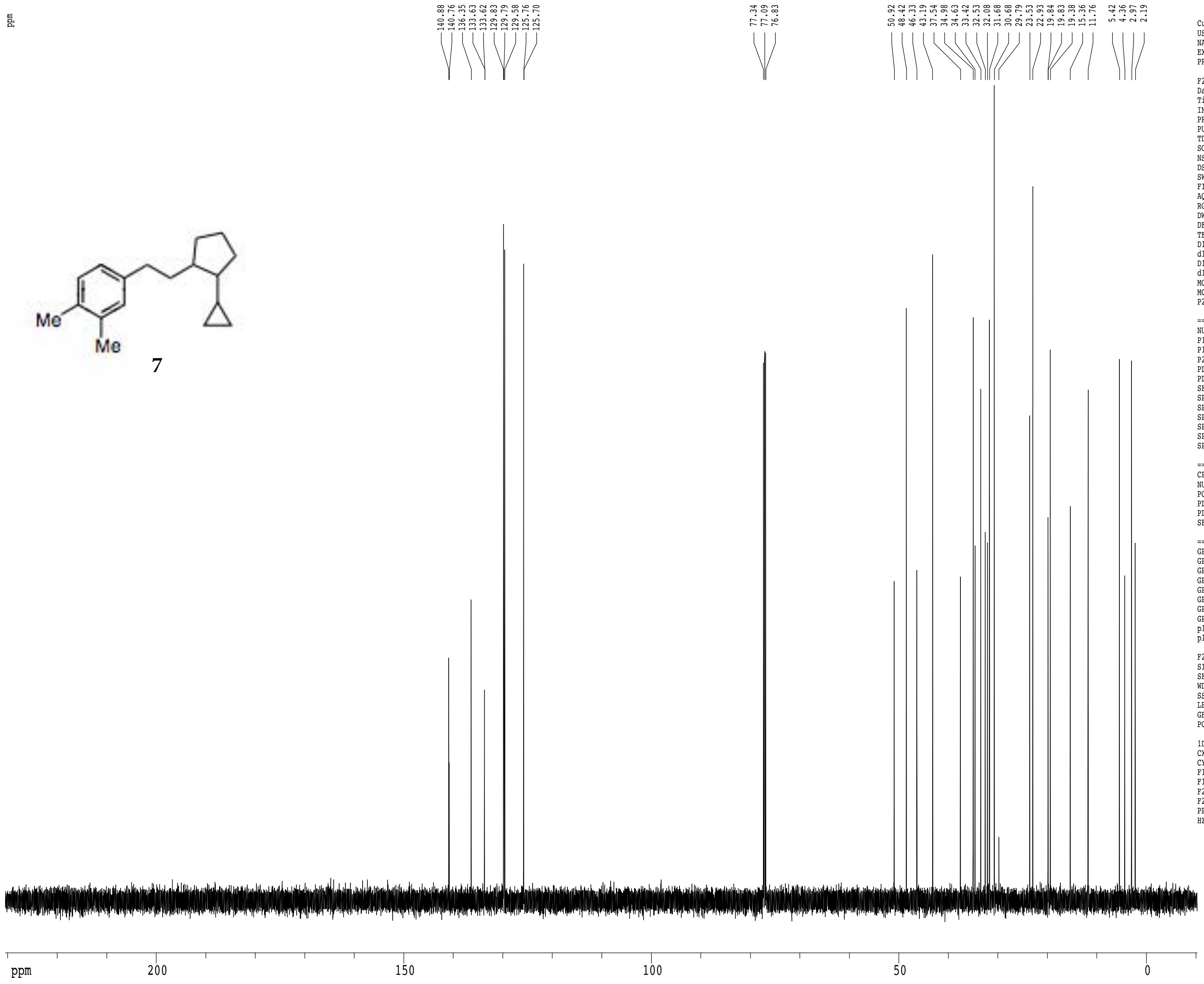

Current Data Parameters
USER caherber
NAME CAH-II-029-full
EXPNO 2
PROCNO 1

F2 - Acquisition Parameters
Date_ 20211115
Time 10.58
INSTRUM cryo500
PROBHD 5 mm CPTCI 1H-
PULPROG SpinEchopg30gp2.prd
TD 65536
SOLVENT CDCl3
NS 216
DS 16
SWH 30303.031 Hz
FIDRES 0.462388 Hz
AQ 1.0813940 sec
RG 7298.2
DW 16.500 usec
DE 6.00 usec
TE 298.0 K
D1 0.25000000 sec
d11 0.03000000 sec
D16 0.00020000 sec
d17 0.00019600 sec
MCREST 0.00000000 sec
MCWRK 0.01500000 sec
P2 37.70 usec

======== CHANNEL f1 ========
NUC1 13C
P1 18.85 usec
P12 2000.00 usec
P20 500.00 usec
PL0 120.00 dB
PL1 -1.00 dB
SFO1 125.7942548 MHz
SP2 1.55 dB
SP4 1.55 dB
SPNAM2 Crp60comp.4
SPNAM4 Crp60,0.5,20.1
SPOFF2 0.00 Hz
SPOFF4 0.00 Hz

======== CHANNEL f2 ========
CPDPRG2 waltz16
NUC2 1H
PCPD2 100.00 usec
PL2 1.60 dB
PL12 22.00 dB
SFO2 500.2225011 MHz

====== GRADIENT CHANNEL =====
GPNAM1 SINE.100
GPNAM2 SINE.100
GPX1 0.00 %
GPX2 0.00 %
GPY1 0.00 %
GPY2 0.00 %
GPZ1 30.00 %
GPZ2 50.00 %
p15 500.00 usec
p16 1000.00 usec

F2 - Processing parameters
SI 65536
SF 125.7804190 MHz
WDW no
SSB 0
LB 0.00 Hz
GB 0
PC 2.00

1D NMR plot parameters
CX 22.80 cm
CY 15.65 cm
F1P 230.637 ppm
F1 29009.68 Hz
F2P -10.287 ppm
F2 -1293.96 Hz
PPMCM 10.56688 ppm/cm
HZCM 1329.10706 Hz/cm

1H spectrum

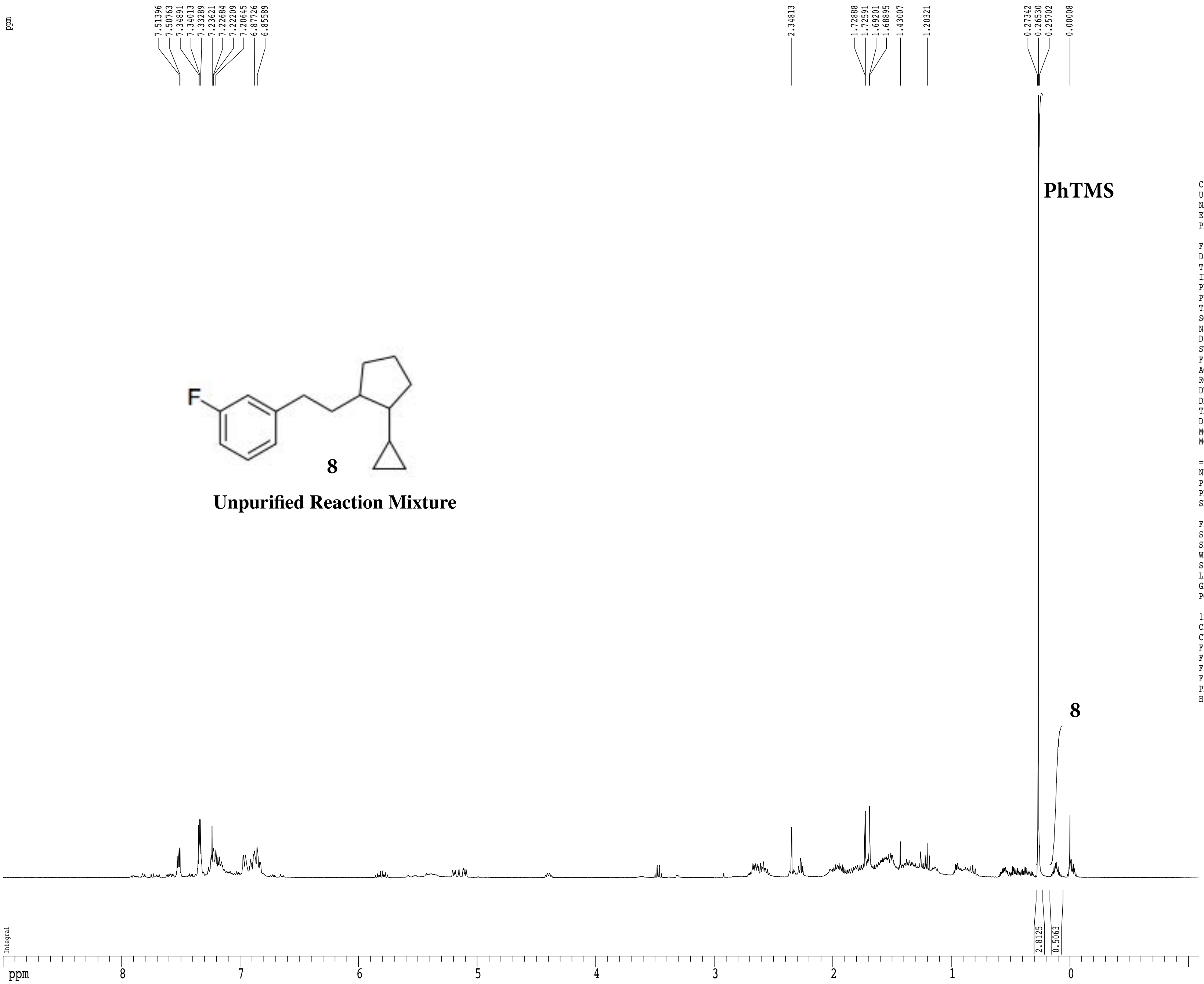


```
Current Data Parameters
USER          caherber
NAME     CAH-I-258-Crude
EXPNO                1
PROCNO               1

F2 - Acquisition Parameters
Date_          20210804
Time              15.46
INSTRUM          drx400
PROBHD   5 mm QNP H/F/P
PULPROG            zg30
TD                65536
SOLVENT           CDCl3
NS                    8
DS                    2
SWH            6410.256 Hz
FIDRES         0.097813 Hz
AQ            5.1118579 sec
RG                 90.5
DW               78.000 usec
DE                 4.50 usec
TE                298.0 K
D1           0.10000000 sec
MCREST       0.00000000 sec
MCWRK        0.01500000 sec

======== CHANNEL f1 ========
NUC1                 1H
P1                12.00 usec
PL1               -1.60 dB
SFO1        400.1328009 MHz

F2 - Processing parameters
SI                65536
SF          400.1300308 MHz
WDW                  no
SSB                   0
LB                 0.00 Hz
GB                    0
PC                 2.00

1D NMR plot parameters
CX                22.80 cm
CY                15.00 cm
F1P               9.000 ppm
F1              3601.17 Hz
F2P              -1.087 ppm
F2              -434.97 Hz
PPMCM           0.44242 ppm/cm
HZCM          177.02393 Hz/cm
```

1H spectrum

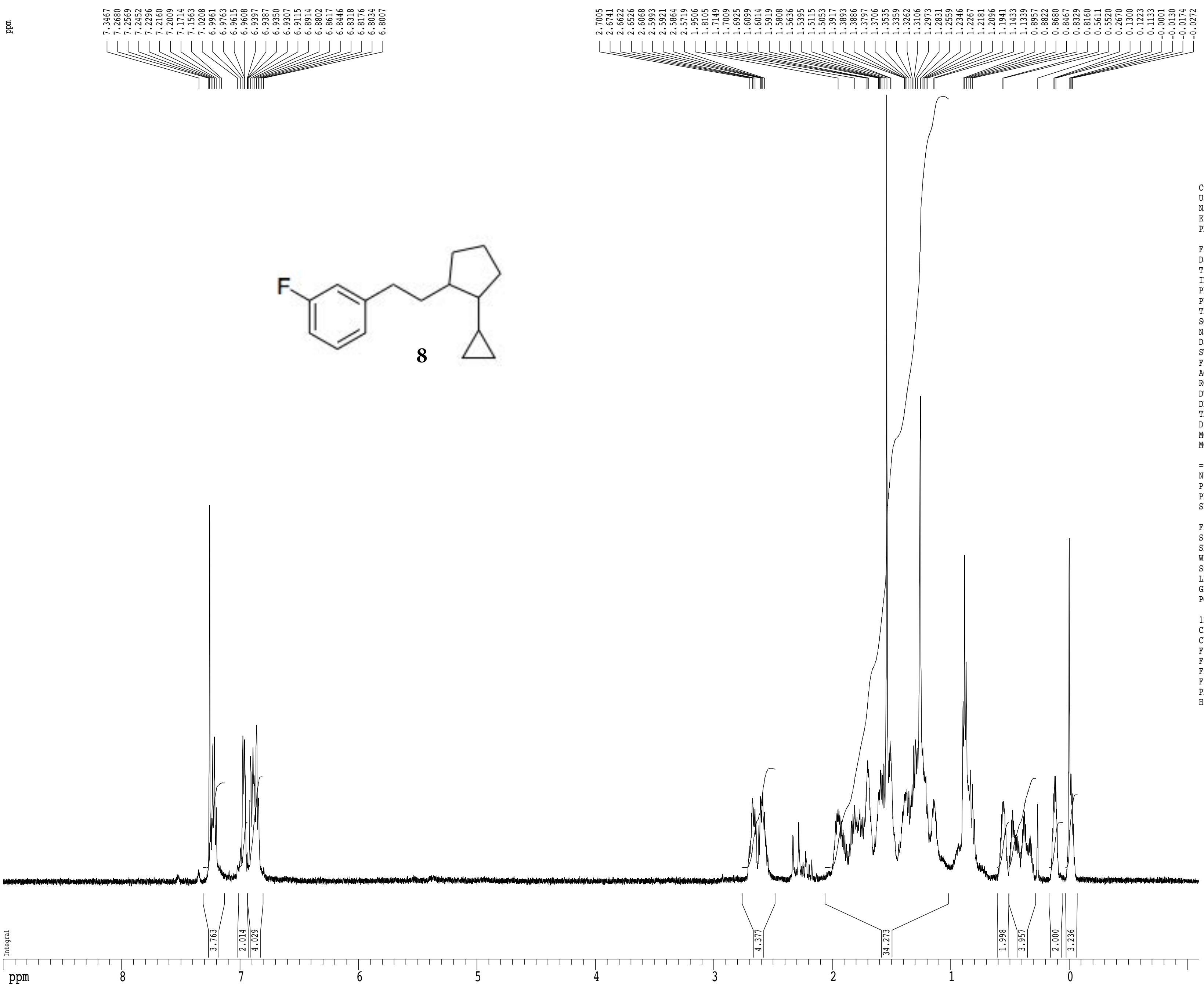

Current Data Parameters
USER caherber
NAME CAH-I-290
EXPNO 1
PROCNO 1

F2 - Acquisition Parameters
Date_ 20211005
Time 16.58
INSTRUM gn500
PROBHD 5 mm broadband
PULPROG zg30
TD 81728
SOLVENT CDCl3
NS 8
DS 2
SWH 8012.820 Hz
FIDRES 0.098043 Hz
AQ 5.0998774 sec
RG 912.3
DW 62.400 usec
DE 6.00 usec
TE 298.0 K
D1 0.10000000 sec
MCREST 0.00000000 sec
MCWRK 0.01500000 sec

======== CHANNEL f1 ========
NUC1 1H
P1 12.00 usec
PL1 -6.00 dB
SFO1 498.6534906 MHz

F2 - Processing parameters
SI 65536
SF 498.6500301 MHz
WDW no
SSB 0
LB 0.00 Hz
GB 0
PC 1.00

1D NMR plot parameters
CX 22.80 cm
CY 15.00 cm
F1P 9.000 ppm
F1 4487.85 Hz
F2P -1.095 ppm
F2 -545.92 Hz
PPMCM 0.44275 ppm/cm
HZCM 220.77931 Hz/cm

@c13.c

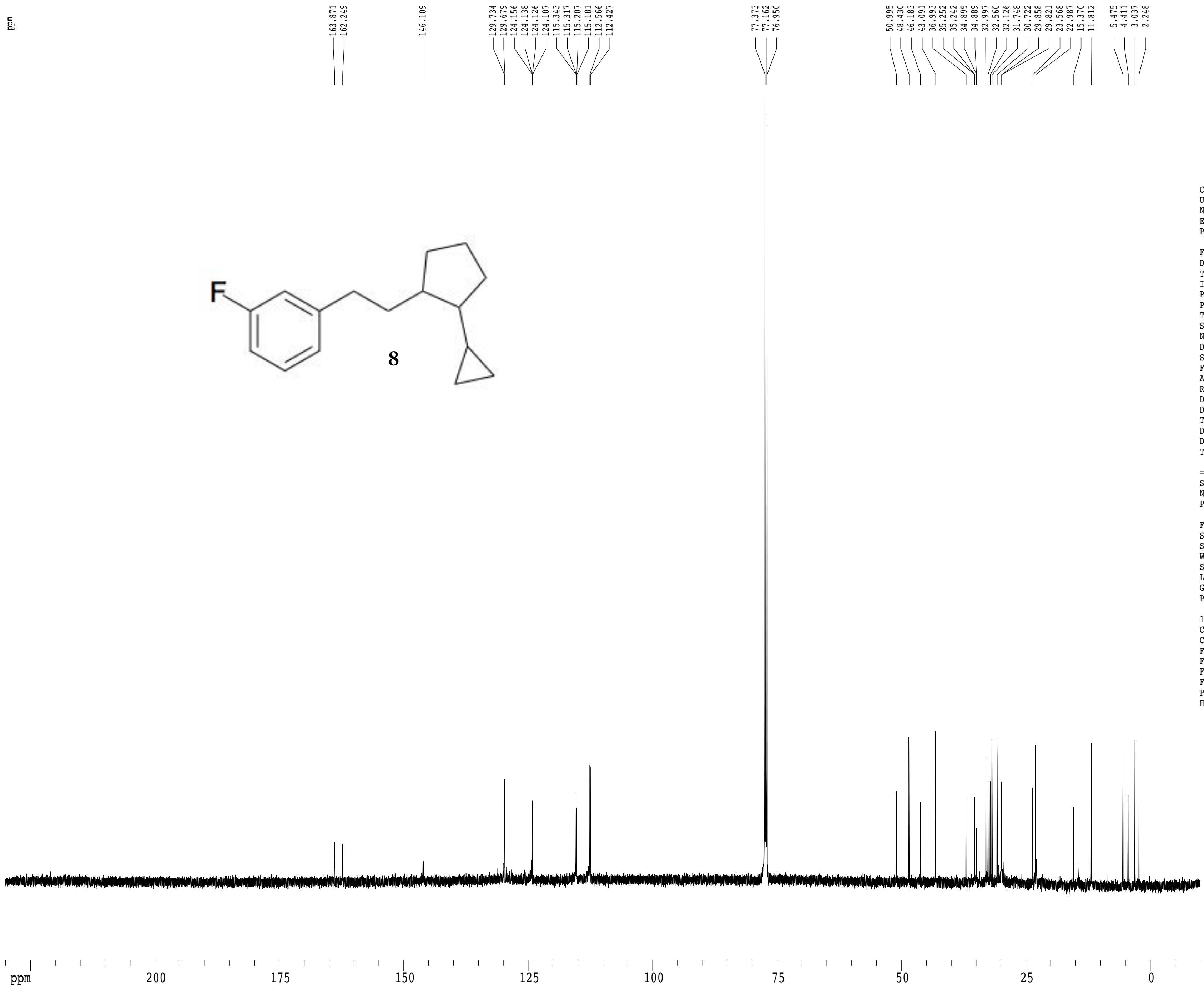

Current Data Parameters
USER caherber
NAME CAH-I-258-full
EXPNO 3
PROCNO 1

F2 - Acquisition Parameters
Date_ 20210811
Time 13.56
INSTRUM av600
PROBHD 5 mm CPBBO BB-
PULPROG zgdc30
TD 65536
SOLVENT CDCl3
NS 404
DS 4
SWH 36231.883 Hz
FIDRES 0.552855 Hz
AQ 0.9044468 sec
RG 2050
DW 13.800 usec
DE 19.63 usec
TE 298.0 K
D1 0.40000001 sec
D11 0.03000000 sec
TD0 1

======== CHANNEL f1 ========
SFO1 150.9194080 MHz
NUC1 13C
P1 10.10 usec

F2 - Processing parameters
SI 65536
SF 150.9027942 MHz
WDW no
SSB 0
LB 0.00 Hz
GB 0
PC 1.00

1D NMR plot parameters
CX 22.80 cm
CY 15.00 cm
F1P 230.147 ppm
F1 34729.77 Hz
F2P -9.954 ppm
F2 -1502.12 Hz
PPMCM 10.53074 ppm/cm
HZCM 1589.11780 Hz/cm

@f19.c

ppm

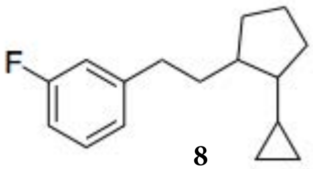

8

-113.76
-113.82
-113.84
-113.85
-113.87
-113.91
-113.99
-113.99
-114.00
-114.04
-114.06
-114.08
-114.09
-114.10
-114.12

```
Current Data Parameters
USER            caherber
NAME      CAH-I-258-full
EXPNO                  2
PROCNO                 1

F2 - Acquisition Parameters
Date_           20210811
Time               13.43
INSTRUM            av600
PROBHD   5 mm CPBBO BB-
PULPROG         zgflqn30
TD                131072
SOLVENT            CDCl3
NS                    16
DS                     2
SWH           178571.422 Hz
FIDRES          1.362392 Hz
AQ             0.3670516 sec
RG                   575
DW                 2.800 usec
DE                 18.00 usec
TE                 298.0 K
D1            3.00000000 sec
TD0                    1

======== CHANNEL f1 ========
SFO1         564.6299196 MHz
NUC1                 19F
P1                 18.25 usec

F2 - Processing parameters
SI                131072
SF           564.6863882 MHz
WDW                   no
SSB                    0
LB                  0.00 Hz
GB                     0
PC                  1.00

1D NMR plot parameters
CX                 22.80 cm
CY                 15.00 cm
F1P               58.116 ppm
F1              32817.10 Hz
F2P             -258.116 ppm
F2            -145754.33 Hz
PPMCM           13.86979 ppm/cm
HZCM          7832.08057 Hz/cm
```

ppm 0 -50 -100 -150 -200 -250

h1.c

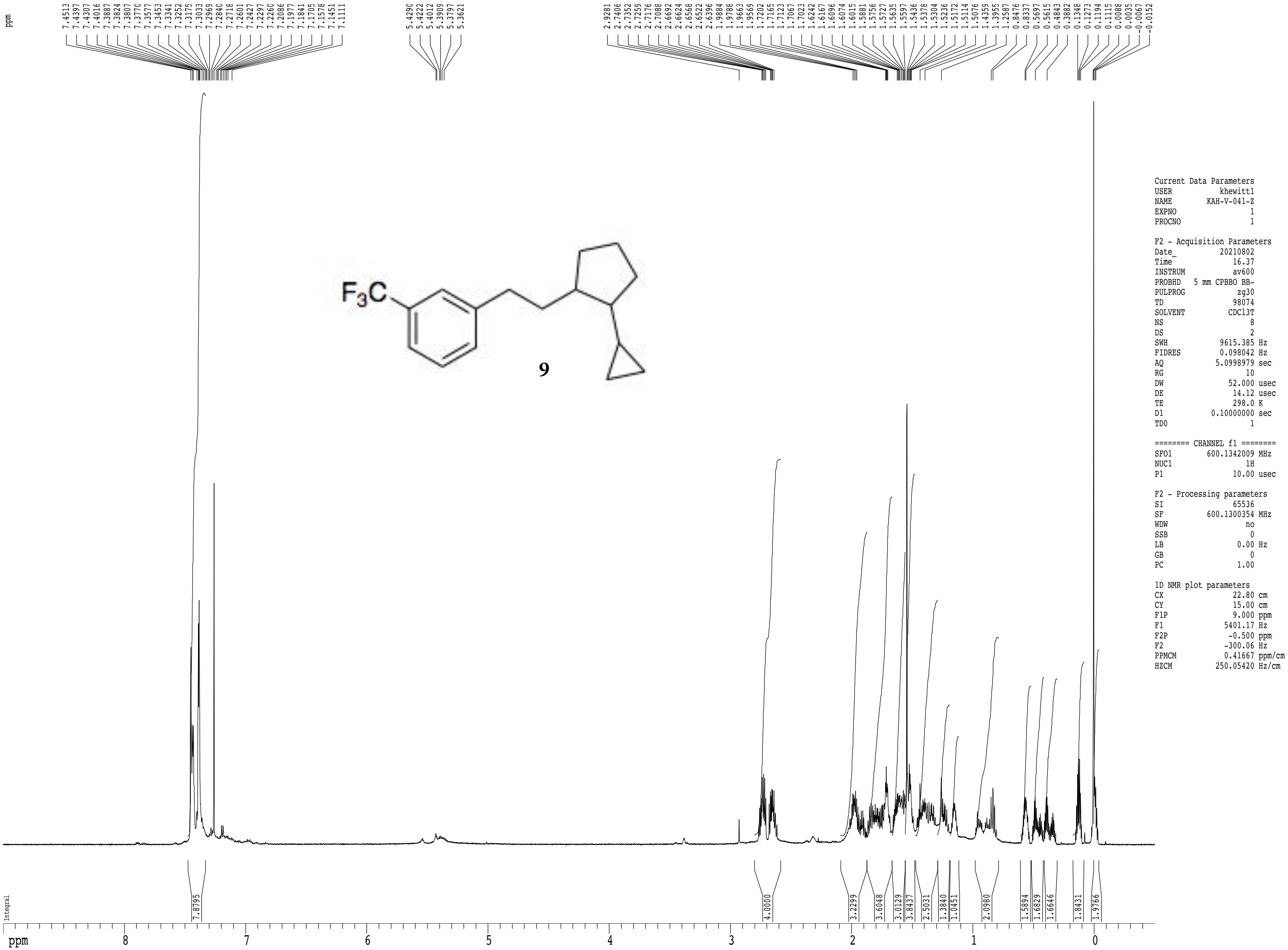

Current Data Parameters
USER khewitt1
NAME KAH-V-041-Z
EXPNO 1
PROCNO 1

F2 - Acquisition Parameters
Date_ 20210802
Time 16.37
INSTRUM av600
PROBHD 5 mm CPBBO BB-
PULPROG zg30
TD 98074
SOLVENT CDCl3T
NS 8
DS 2
SWH 9615.385 Hz
FIDRES 0.098042 Hz
AQ 5.0998979 sec
RG 10
DW 52.000 usec
DE 14.12 usec
TE 298.0 K
D1 0.10000000 sec
TD0 1

======== CHANNEL f1 ========
SFO1 600.1342009 MHz
NUC1 1H
P1 10.00 usec

F2 - Processing parameters
SI 65536
SF 600.1300354 MHz
WDW no
SSB 0
LB 0.00 Hz
GB 0
PC 1.00

1D NMR plot parameters
CX 22.80 cm
CY 15.00 cm
F1P 9.000 ppm
F1 5401.17 Hz
F2P -0.500 ppm
F2 -300.06 Hz
PPMCM 0.41667 ppm/cm
HZCM 250.05420 Hz/cm

c13.c

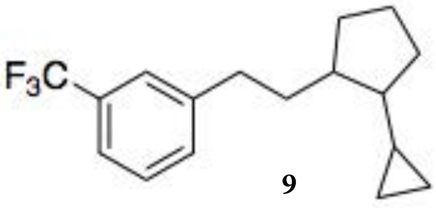
F3C

9

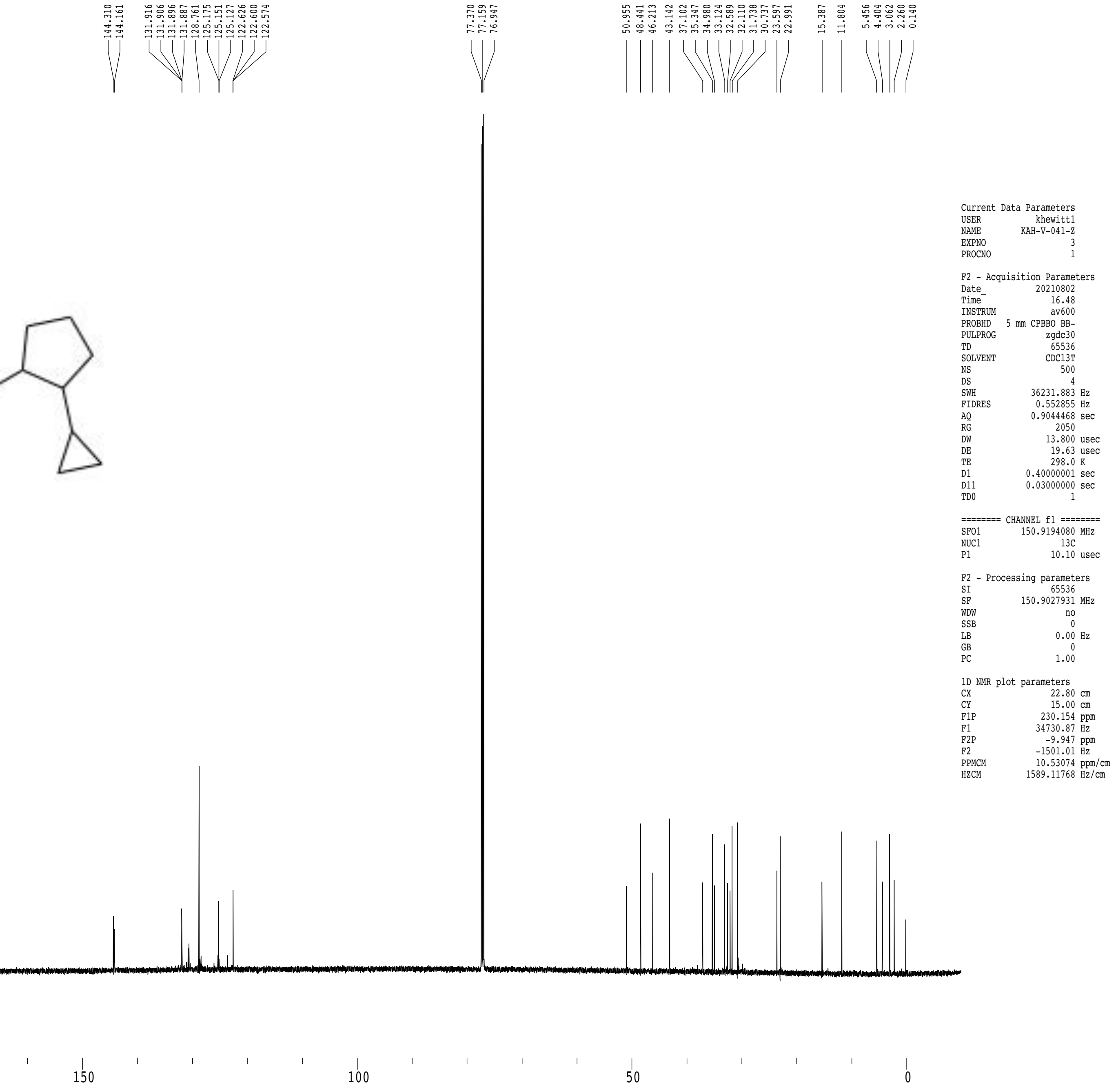
ppm
144.310
144.161
131.916
131.906
131.896
131.887
128.761
125.175
125.151
125.127
122.626
122.600
122.574
77.370
77.159
76.947
50.955
48.441
46.213
43.142
37.102
35.347
34.980
33.124
32.589
32.110
31.738
30.737
23.597
22.991
15.387
11.804
5.456
4.404
3.062
2.260
0.140
Current Data Parameters
USER khewitt1
NAME KAH-V-041-Z
EXPNO 3
PROCNO 1
F2 - Acquisition Parameters
Date_ 20210802
Time 16.48
INSTRUM av600
PROBHD 5 mm CPBBO BB-
PULPROG zgdc30
TD 65536
SOLVENT CDCl3T
NS 500
DS 4
SWH 36231.883 Hz
FIDRES 0.552855 Hz
AQ 0.9044468 sec
RG 2050
DW 13.800 usec
DE 19.63 usec
TE 298.0 K
D1 0.40000001 sec
D11 0.03000000 sec
TD0 1
======== CHANNEL f1 ========
SFO1 150.9194080 MHz
NUC1 13C
P1 10.10 usec
F2 - Processing parameters
SI 65536
SF 150.9027931 MHz
WDW no
SSB 0
LB 0.00 Hz
GB 0
PC 1.00
1D NMR plot parameters
CX 22.80 cm
CY 15.00 cm
F1P 230.154 ppm
F1 34730.87 Hz
F2P -9.947 ppm
F2 -1501.01 Hz
PPMCM 10.53074 ppm/cm
HZCM 1589.11768 Hz/cm
ppm
200
150
100
50
0

f19.c

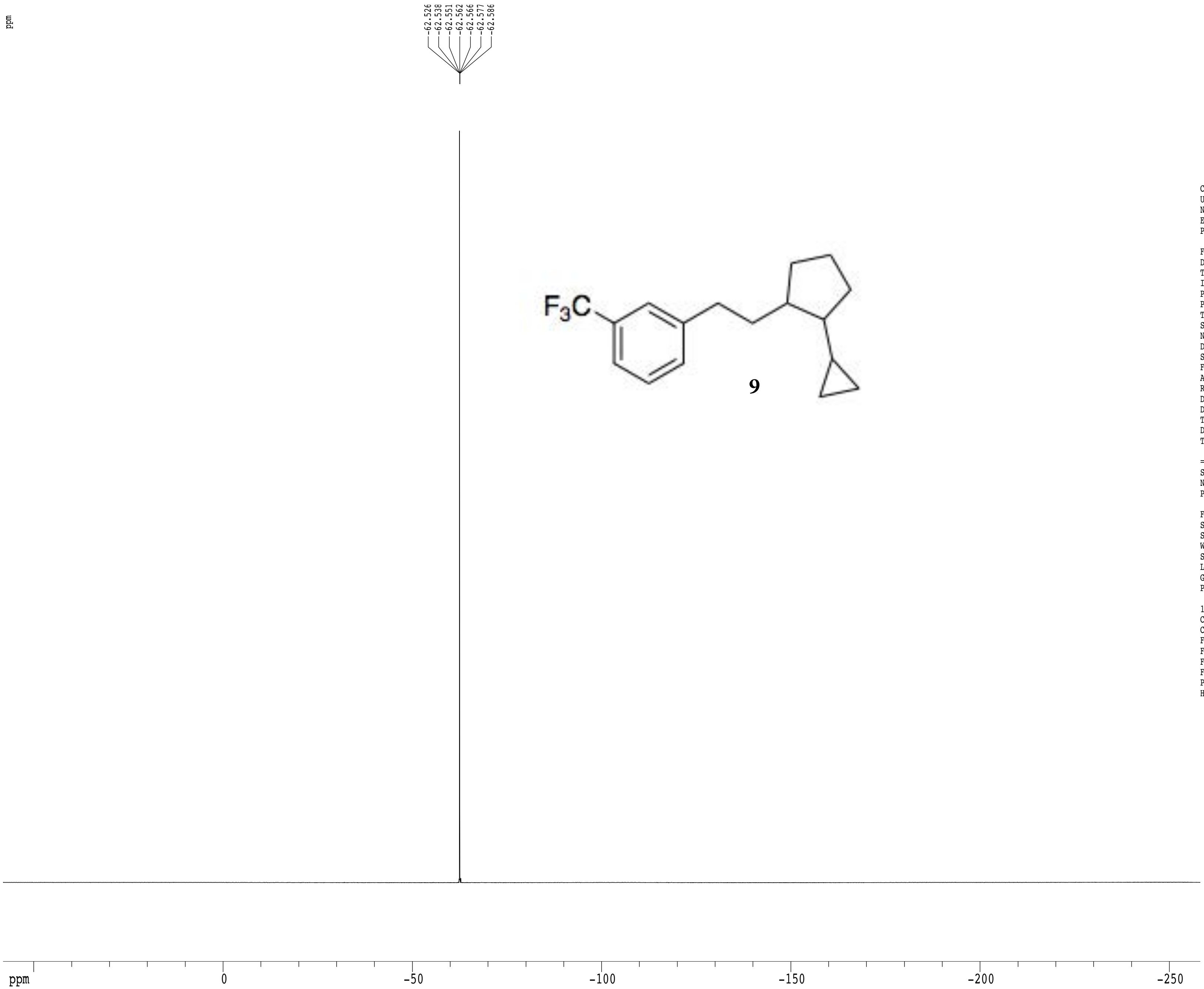

9

```
Current Data Parameters
USER           khewitt1
NAME        KAH-V-041-Z
EXPNO                 2
PROCNO                1

F2 - Acquisition Parameters
Date_          20210802
Time              16.43
INSTRUM           av600
PROBHD   5 mm CPBBO BB-
PULPROG        zgflqn30
TD               131072
SOLVENT          CDCl3T
NS                   16
DS                    2
SWH          178571.422 Hz
FIDRES         1.362392 Hz
AQ            0.3670516 sec
RG                  575
DW                2.800 usec
DE                18.00 usec
TE                298.0 K
D1           3.00000000 sec
TD0                   1

======== CHANNEL f1 ========
SFO1        564.6299196 MHz
NUC1                19F
P1                18.25 usec

F2 - Processing parameters
SI               131072
SF          564.6864234 MHz
WDW                  no
SSB                   0
LB                 0.00 Hz
GB                    0
PC                 1.00

1D NMR plot parameters
CX                22.80 cm
CY                15.00 cm
F1P              58.053 ppm
F1             32781.88 Hz
F2P            -258.178 ppm
F2           -145789.55 Hz
PPMCM          13.86979 ppm/cm
HZCM         7832.08008 Hz/cm
```

1H spectrum

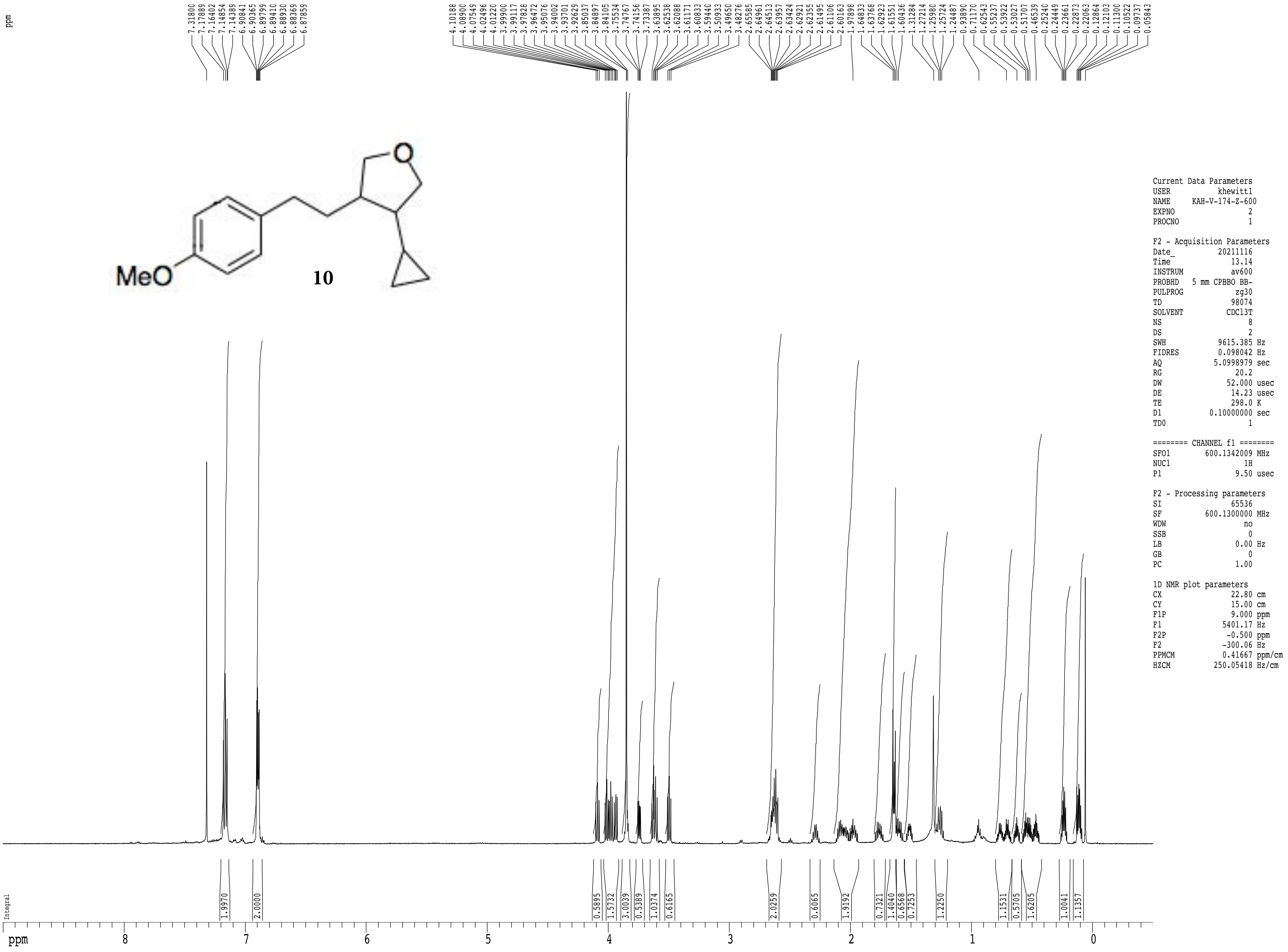
MeO
10
Current Data Parameters
USER khewitt1
NAME KAH-V-174-Z-600
EXPNO 2
PROCNO 1
F2 - Acquisition Parameters
Date_ 20211116
Time 13.14
INSTRUM av600
PROBHD 5 mm CPBBO BB-
PULPROG zg30
TD 98074
SOLVENT CDCl3T
NS 8
DS 2
SWH 9615.385 Hz
FIDRES 0.098042 Hz
AQ 5.0998979 sec
RG 20.2
DW 52.000 usec
DE 14.23 usec
TE 298.0 K
D1 0.10000000 sec
TD0 1
======== CHANNEL f1 ========
SFO1 600.1342009 MHz
NUC1 1H
P1 9.50 usec
F2 - Processing parameters
SI 65536
SF 600.1300000 MHz
WDW no
SSB 0
LB 0.00 Hz
GB 0
PC 1.00
1D NMR plot parameters
CX 22.80 cm
CY 15.00 cm
F1P 9.000 ppm
F1 5401.17 Hz
F2P -0.500 ppm
F2 -300.06 Hz
PPMCM 0.41667 ppm/cm
HZCM 250.05418 Hz/cm
ppm
Integral

13C spectrum

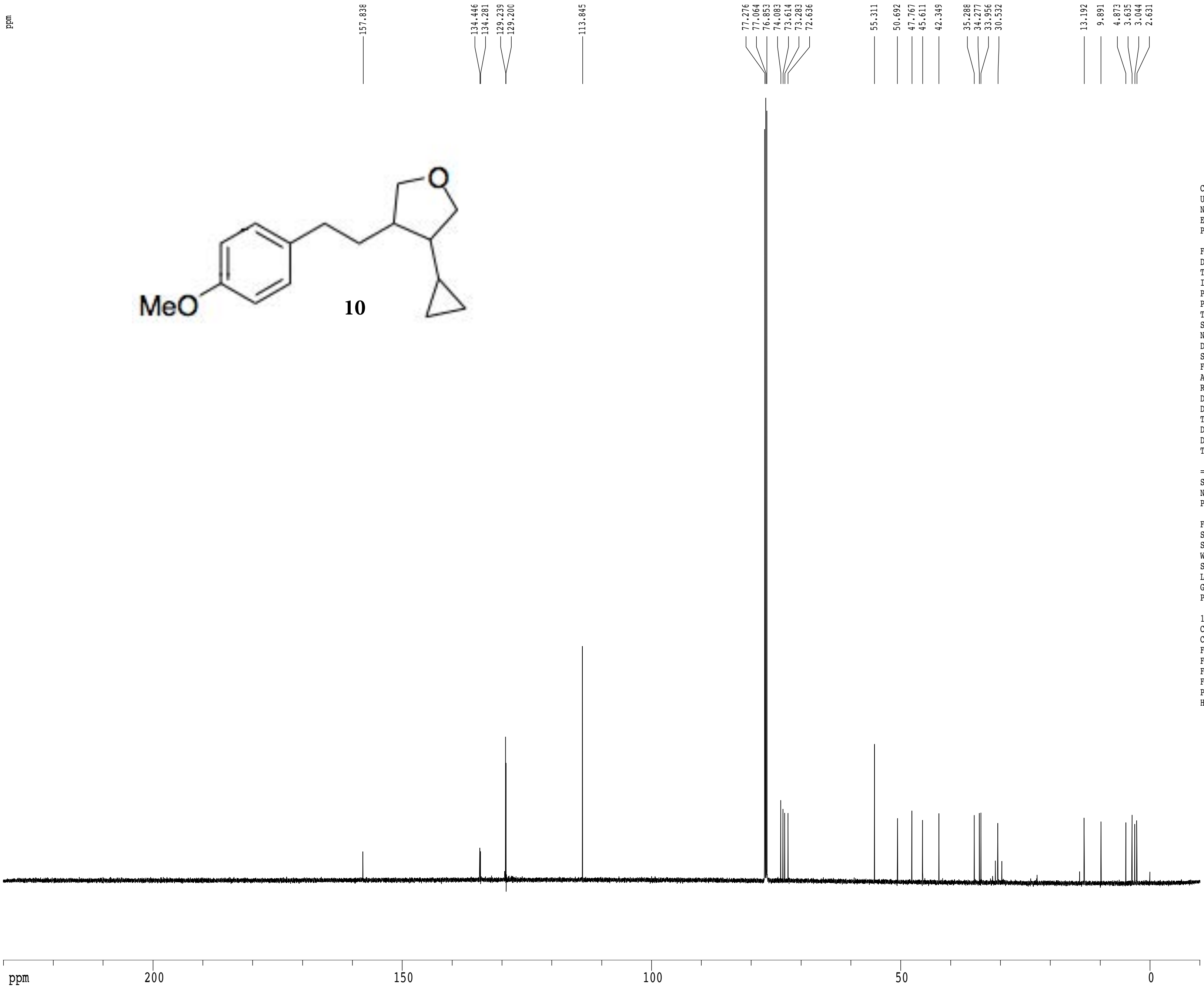

10

```
Current Data Parameters
USER            khewitt1
NAME         KAH-V-174-Z
EXPNO                  2
PROCNO                 1

F2 - Acquisition Parameters
Date_           20211207
Time               12.26
INSTRUM            av600
PROBHD   5 mm CPBBO BB-
PULPROG           zgdc30
TD                 65536
SOLVENT           CDCl3T
NS                   283
DS                     4
SWH            36231.883 Hz
FIDRES          0.552855 Hz
AQ             0.9044468 sec
RG                  2050
DW                13.800 usec
DE                 19.63 usec
TE                 297.9 K
D1            0.40000001 sec
D11           0.03000000 sec
TD0                    1

======== CHANNEL f1 ========
SFO1         150.9194080 MHz
NUC1                 13C
P1                 10.10 usec

F2 - Processing parameters
SI                 65536
SF           150.9028085 MHz
WDW                   no
SSB                    0
LB                  0.00 Hz
GB                     0
PC                  1.00

1D NMR plot parameters
CX                 22.80 cm
CY                 15.00 cm
F1P              230.051 ppm
F1              34715.41 Hz
F2P              -10.049 ppm
F2              -1516.47 Hz
PPMCM           10.53074 ppm/cm
HZCM          1589.11755 Hz/cm
```

1H spectrum

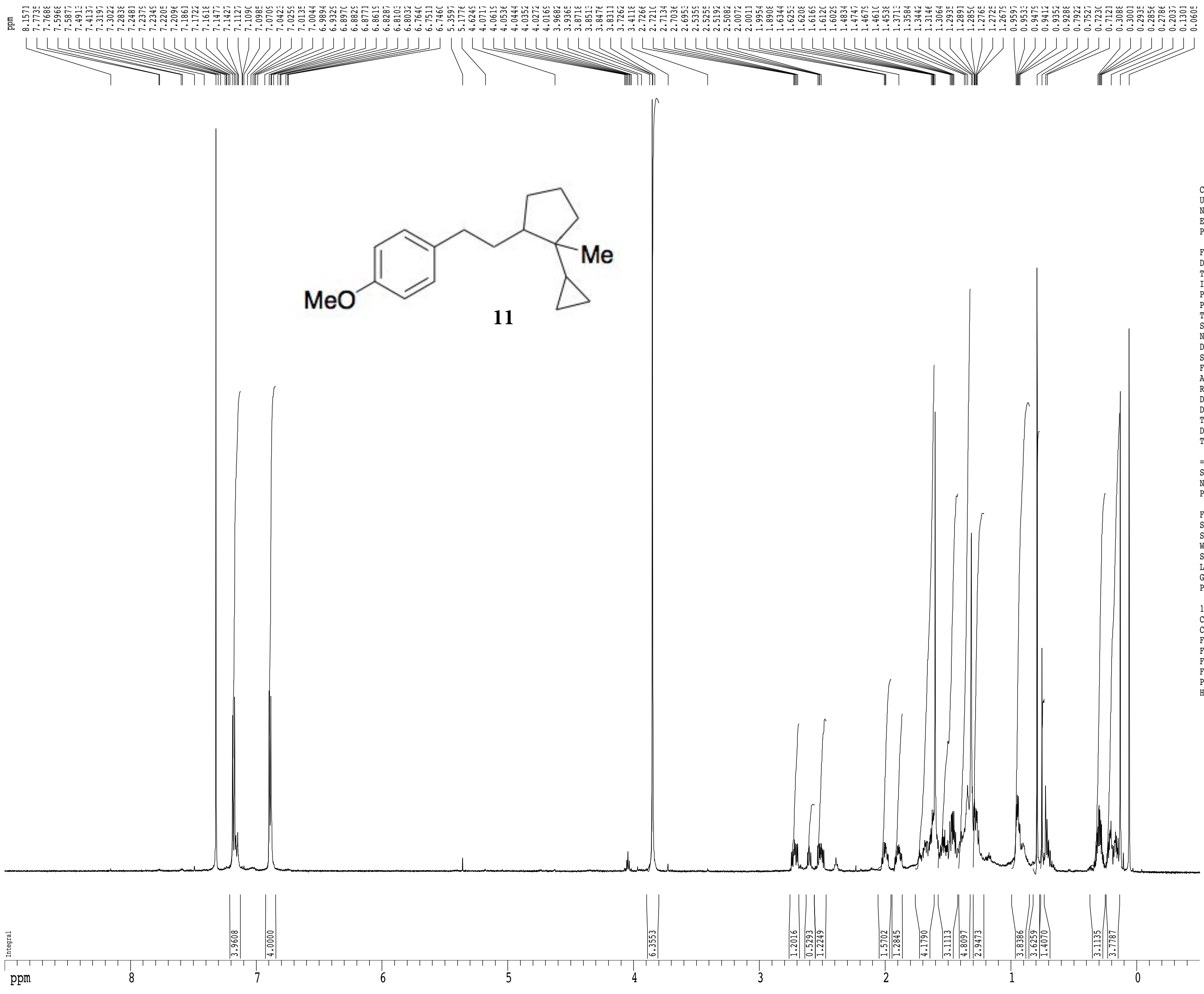

Current Data Parameters
USER khewitt1
NAME KAH-V-211-Z
EXPNO 2
PROCNO 1

F2 - Acquisition Parameters
Date_ 20220110
Time 16.17
INSTRUM av600
PROBHD 5 mm CPBBO BB-
PULPROG zg30
TD 98074
SOLVENT CDCl3T
NS 24
DS 2
SWH 9615.385 Hz
FIDRES 0.098042 Hz
AQ 5.0998979 sec
RG 10
DW 52.000 usec
DE 14.23 usec
TE 297.9 K
D1 0.10000000 sec
TD0 1

======== CHANNEL f1 ========
SFO1 600.1342009 MHz
NUC1 1H
P1 9.50 usec

F2 - Processing parameters
SI 65536
SF 600.1300000 MHz
WDW no
SSB 0
LB 0.00 Hz
GB 0
PC 1.00

1D NMR plot parameters
CX 22.80 cm
CY 15.00 cm
F1P 9.000 ppm
F1 5401.17 Hz
F2P -0.500 ppm
F2 -300.06 Hz
PPMCM 0.41667 ppm/cm
HZCM 250.05418 Hz/cm

1H spectrum

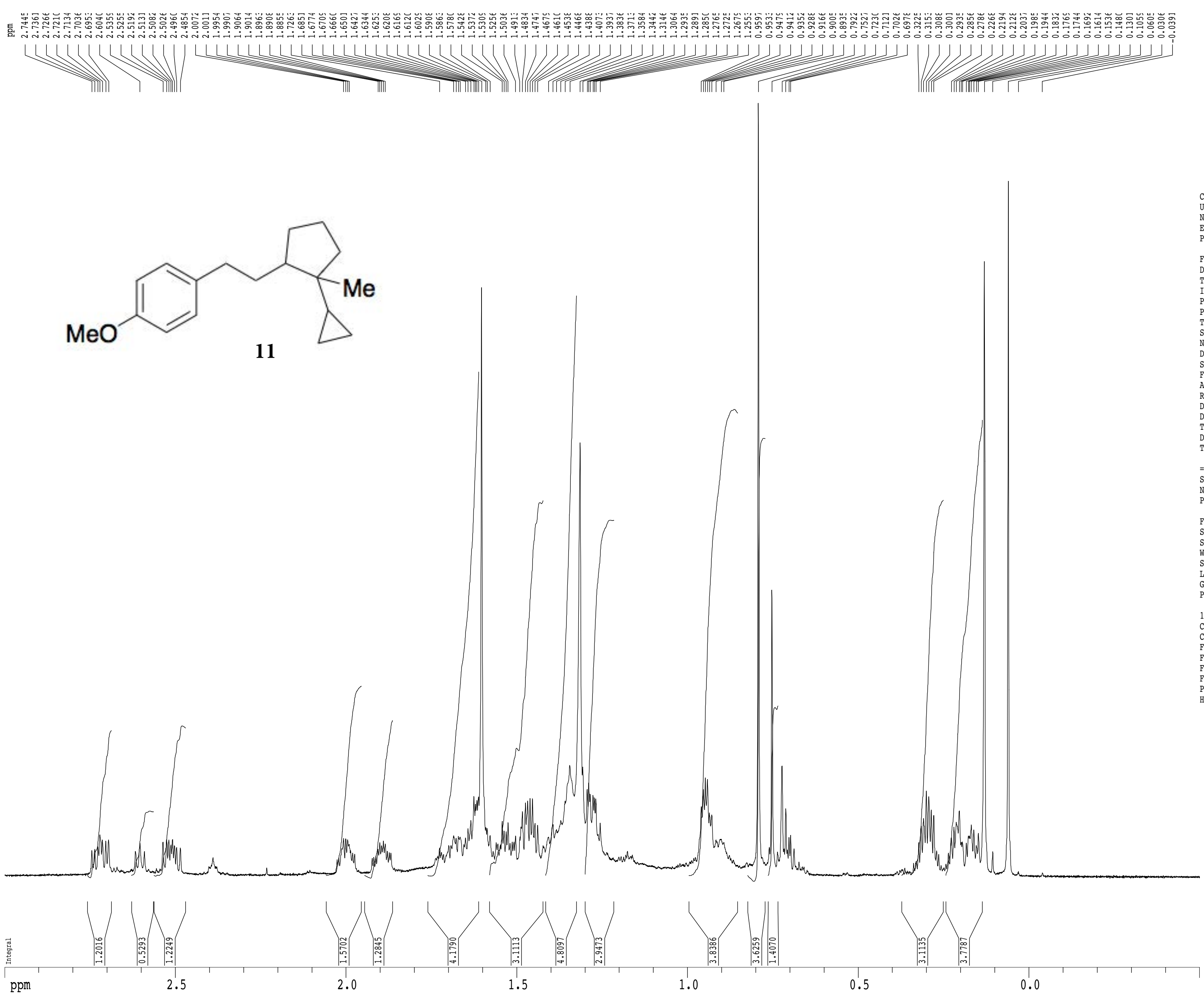


```
Current Data Parameters
USER            khewitt1
NAME          KAH-V-211-Z
EXPNO                   2
PROCNO                  1

F2 - Acquisition Parameters
Date_            20220110
Time                16.17
INSTRUM             av600
PROBHD   5 mm CPBBO BB-
PULPROG              zg30
TD                  98074
SOLVENT             CDCl3T
NS                     24
DS                      2
SWH             9615.385 Hz
FIDRES          0.098042 Hz
AQ             5.0998979 sec
RG                     10
DW                 52.000 usec
DE                  14.23 usec
TE                  297.9 K
D1            0.10000000 sec
TD0                     1

======== CHANNEL f1 ========
SFO1         600.1342009 MHz
NUC1                   1H
P1                   9.50 usec

F2 - Processing parameters
SI                  65536
SF           600.1300000 MHz
WDW                    no
SSB                     0
LB                   0.00 Hz
GB                      0
PC                   1.00

1D NMR plot parameters
CX                  22.80 cm
CY                  15.00 cm
F1P                 3.000 ppm
F1                1800.39 Hz
F2P                -0.500 ppm
F2                -300.07 Hz
PPMCM             0.15351 ppm/cm
HZCM             92.12522 Hz/cm
```

13C spectrum

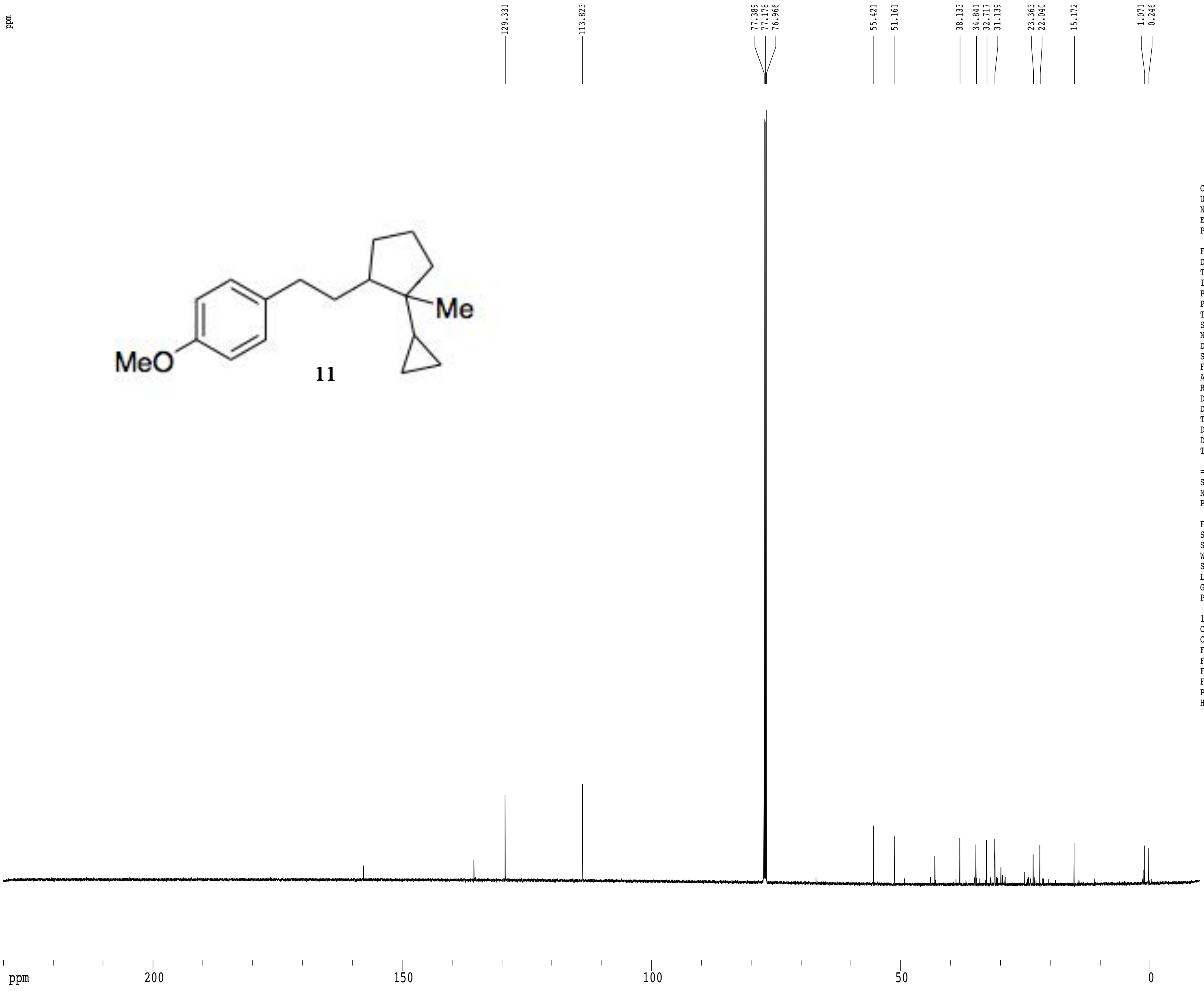

Current Data Parameters
USER khewitt1
NAME KAH-V-211-Z
EXPNO 3
PROCNO 1

F2 - Acquisition Parameters
Date_ 20220110
Time 16.22
INSTRUM av600
PROBHD 5 mm CPBBO BB-
PULPROG zgdc30
TD 65536
SOLVENT CDCl3T
NS 1024
DS 4
SWH 36231.883 Hz
FIDRES 0.552855 Hz
AQ 0.9044468 sec
RG 2050
DW 13.800 usec
DE 19.63 usec
TE 297.9 K
D1 0.40000001 sec
D11 0.03000000 sec
TD0 1

======== CHANNEL f1 ========
SFO1 150.9194080 MHz
NUC1 13C
P1 10.10 usec

F2 - Processing parameters
SI 65536
SF 150.9027907 MHz
WDW no
SSB 0
LB 0.00 Hz
GB 0
PC 1.00

1D NMR plot parameters
CX 22.80 cm
CY 15.00 cm
F1P 230.051 ppm
F1 34715.41 Hz
F2P -10.049 ppm
F2 -1516.47 Hz
PPMCM 10.53074 ppm/cm
HZCM 1589.11743 Hz/cm

gcosy60

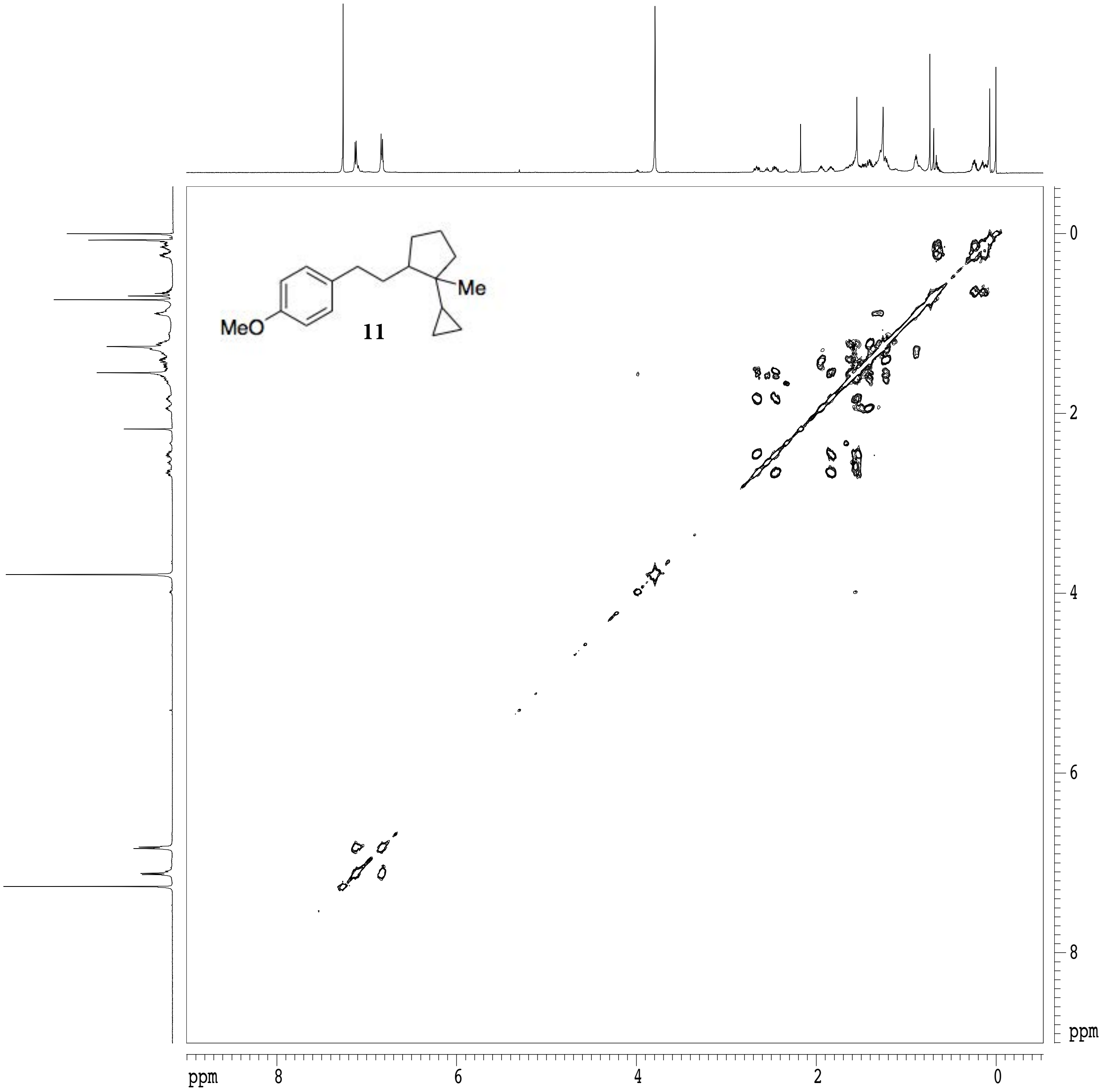


```
        Current Data Parameters
USER              khewitt1
NAME           KAH-V-211-2D
EXPNO                    2
PROCNO                   1

      F2 - Acquisition Parameters
Date_             20220111
Time                 11.06
INSTRUM            cryo500
PROBHD   5 mm CPTCI 1H-
PULPROG      cosygp60.prd
TD                    2048
SOLVENT              CDCl3
NS                       1
DS                      16
SWH               8012.820 Hz
FIDRES            3.912510 Hz
AQ               0.1278452 sec
RG                   724.1
DW                  62.400 usec
DE                    6.00 usec
TE                   298.0 K
d0              0.00000300 sec
D1              1.00000000 sec
d13             0.00000300 sec
D16             0.00020000 sec
IN0             0.00012480 sec

======== CHANNEL f1 ========
NUC1                    1H
P1                    9.75 usec
PL1                   1.60 dB
SFO1           500.2235015 MHz

====== GRADIENT CHANNEL =====
GPNAM1          SMSQ10.100
GPNAM2          SMSQ10.100
GPX1                  0.00 %
GPX2                  0.00 %
GPY1                  0.00 %
GPY2                  0.00 %
GPZ1                 17.00 %
GPZ2                 17.00 %
P16                1000.00 usec

      F1 - Acquisition parameters
ND0                      1
TD                     512
SFO1              500.2235 MHz
FIDRES           15.650040 Hz
SW                  16.018 ppm
FnMODE                  QF

       F2 - Processing parameters
SI                    1024
SF             500.2200307 MHz
WDW                   SINE
SSB                      0
LB                    0.00 Hz
GB                       0
PC                    1.00

       F1 - Processing parameters
SI                    1024
MC2                     QF
SF             500.2200307 MHz
WDW                   SINE
SSB                      0
LB                    0.00 Hz
GB                       0

         2D NMR plot parameters
CX2                  15.00 cm
CX1                  15.00 cm
F2PLO                9.004 ppm
F2LO               4503.78 Hz
F2PHI               -0.523 ppm
F2HI               -261.66 Hz
F1PLO                9.004 ppm
F1LO               4503.78 Hz
F1PHI               -0.523 ppm
F1HI               -261.66 Hz
F2PPMCM            0.63511 ppm/cm
F2HZCM           317.69580 Hz/cm
F1PPMCM            0.63511 ppm/cm
F1HZCM           317.69580 Hz/cm
```

ghmqc

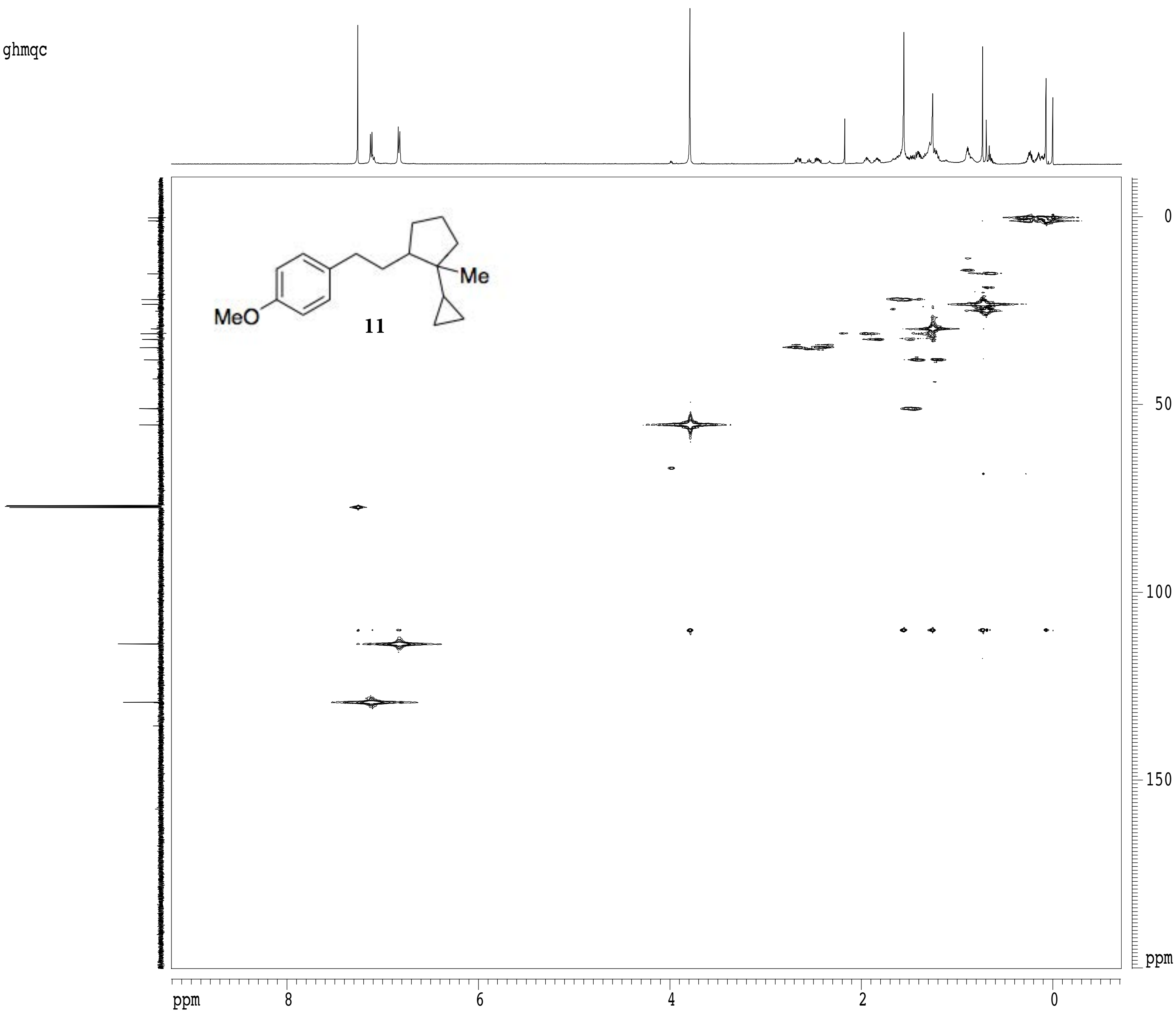


```
Current Data Parameters
USER            khewitt1
NAME         KAH-V-211-2D
EXPNO                  6
PROCNO                 1

F2 - Acquisition Parameters
Date_           20220117
Time               16.45
INSTRUM          cryo500
PROBHD   5 mm CPTCI 1H-
PULPROG       inv4gp.wu
TD                  2048
SOLVENT            CDCl3
NS                     1
DS                    16
SWH             4960.317 Hz
FIDRES          2.422030 Hz
AQ             0.2064884 sec
RG                 26008
DW               100.800 usec
DE                  6.50 usec
TE                 298.0 K
CNST2        145.0000000
d0            0.00000300 sec
D1            1.00000000 sec
d2            0.00344828 sec
d12           0.00002000 sec
d13           0.00000300 sec
D16           0.00020000 sec
d20           0.00242528 sec
IN0           0.00001650 sec

======== CHANNEL f1 ========
NUC1                  1H
P1                  9.75 usec
p2                 19.50 usec
PL1                 1.60 dB
SFO1         500.2221566 MHz

======== CHANNEL f2 ========
CPDPRG2             garp
NUC2                 13C
P3                 18.85 usec
PCPD2              70.00 usec
PL2                -1.00 dB
PL12               10.40 dB
SFO2         125.7942548 MHz

====== GRADIENT CHANNEL =====
GPNAM1        SMSQ10.100
GPNAM2        SMSQ10.100
GPNAM3        SMSQ10.100
GPX1                0.00 %
GPX2                0.00 %
GPX3                0.00 %
GPY1                0.00 %
GPY2                0.00 %
GPY3                0.00 %
GPZ1               30.00 %
GPZ2               18.00 %
GPZ3               24.00 %
P16              1000.00 usec

F1 - Acquisition parameters
ND0                    2
TD                   512
SFO1            125.7943 MHz
FIDRES         59.185608 Hz
SW               240.894 ppm
FnMODE                QF

F2 - Processing parameters
SI                  1024
SF           500.2200307 MHz
WDW                   EM
SSB                    0
LB                  5.00 Hz
GB                     0
PC                  2.00

F1 - Processing parameters
SI                  1024
MC2                   QF
SF           125.7804071 MHz
WDW                QSINE
SSB                    3
LB                  0.00 Hz
GB                     0

2D NMR plot parameters
CX2                18.00 cm
CX1                15.00 cm
F2PLO              9.208 ppm
F2LO             4606.09 Hz
F2PHI             -0.718 ppm
F2HI             -359.07 Hz
F1PLO            200.204 ppm
F1LO            25181.78 Hz
F1PHI            -10.601 ppm
F1HI            -1333.37 Hz
F2PPMCM          0.55144 ppm/cm
F2HZCM         275.84232 Hz/cm
F1PPMCM         14.05367 ppm/cm
F1HZCM        1767.67676 Hz/cm
```

1H spectrum

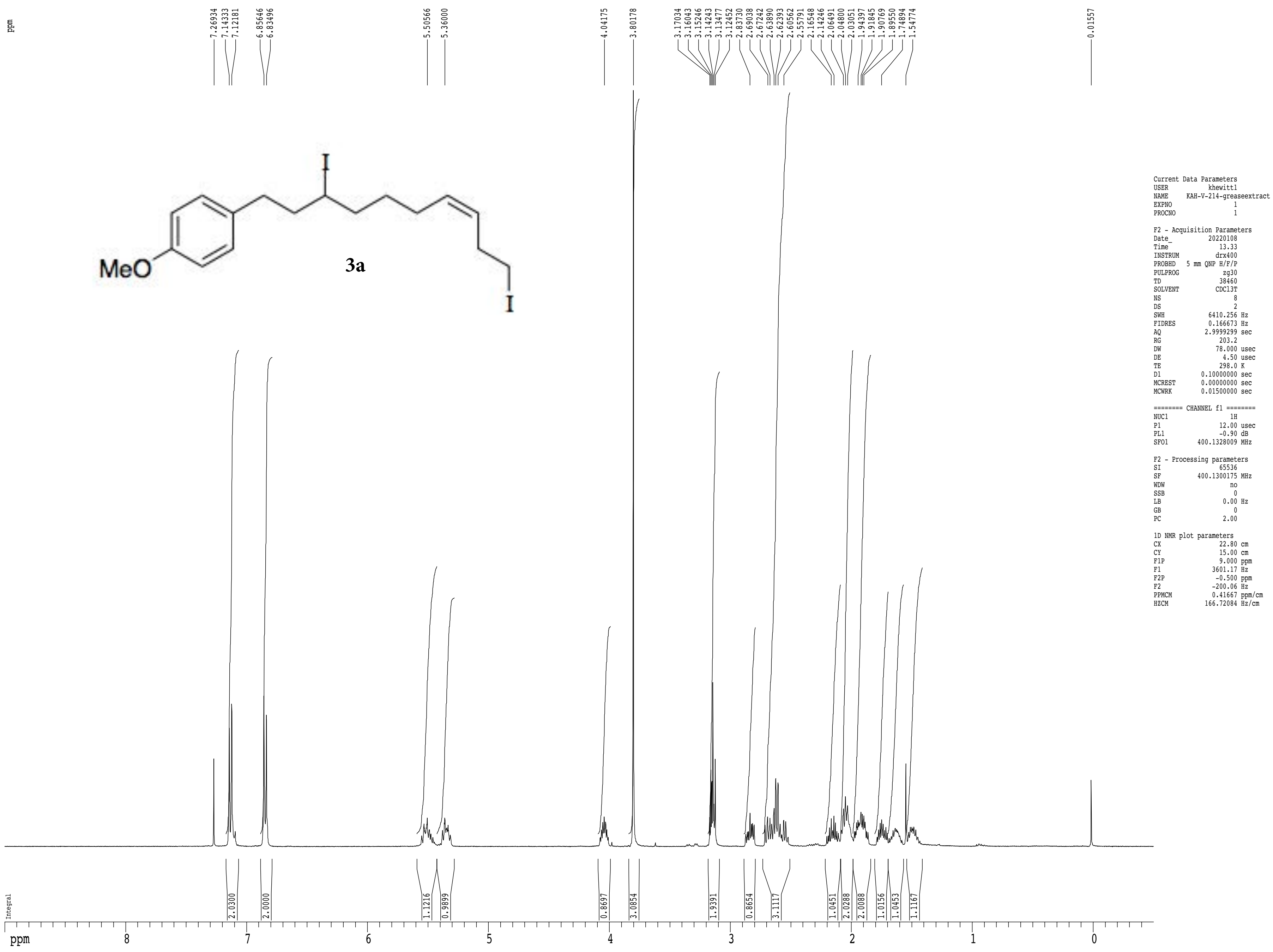
ppm
7.26934
7.14333
7.12181
6.85646
6.83496
5.50566
5.36000
4.04175
3.80178
3.17034
3.16043
3.15246
3.14243
3.13477
3.12452
2.83730
2.69038
2.67242
2.63890
2.62393
2.60562
2.55791
2.16548
2.14246
2.06491
2.04800
2.03051
1.94397
1.91845
1.90769
1.89550
1.74894
1.54774
0.01557
MeO
I
I
3a
Current Data Parameters
USER khewitt1
NAME KAH-V-214-greaseextract
EXPNO 1
PROCNO 1
F2 - Acquisition Parameters
Date_ 20220108
Time 13.33
INSTRUM drx400
PROBHD 5 mm QNP H/F/P
PULPROG zg30
TD 38460
SOLVENT CDCl3T
NS 8
DS 2
SWH 6410.256 Hz
FIDRES 0.166673 Hz
AQ 2.9999299 sec
RG 203.2
DW 78.000 usec
DE 4.50 usec
TE 298.0 K
D1 0.10000000 sec
MCREST 0.00000000 sec
MCWRK 0.01500000 sec
======== CHANNEL f1 ========
NUC1 1H
P1 12.00 usec
PL1 -0.90 dB
SFO1 400.1328009 MHz
F2 - Processing parameters
SI 65536
SF 400.1300175 MHz
WDW no
SSB 0
LB 0.00 Hz
GB 0
PC 2.00
1D NMR plot parameters
CX 22.80 cm
CY 15.00 cm
F1P 9.000 ppm
F1 3601.17 Hz
F2P -0.500 ppm
F2 -200.06 Hz
PPMCM 0.41667 ppm/cm
HZCM 166.72084 Hz/cm
Integral
2.0300
2.0000
1.1216
0.9899
0.8697
3.0854
1.9391
0.8654
3.1117
1.0451
2.0288
2.0088
1.0156
1.0453
1.1167
ppm
8
7
6
5
4
3
2
1
0

13C spectrum with 1H decoupling

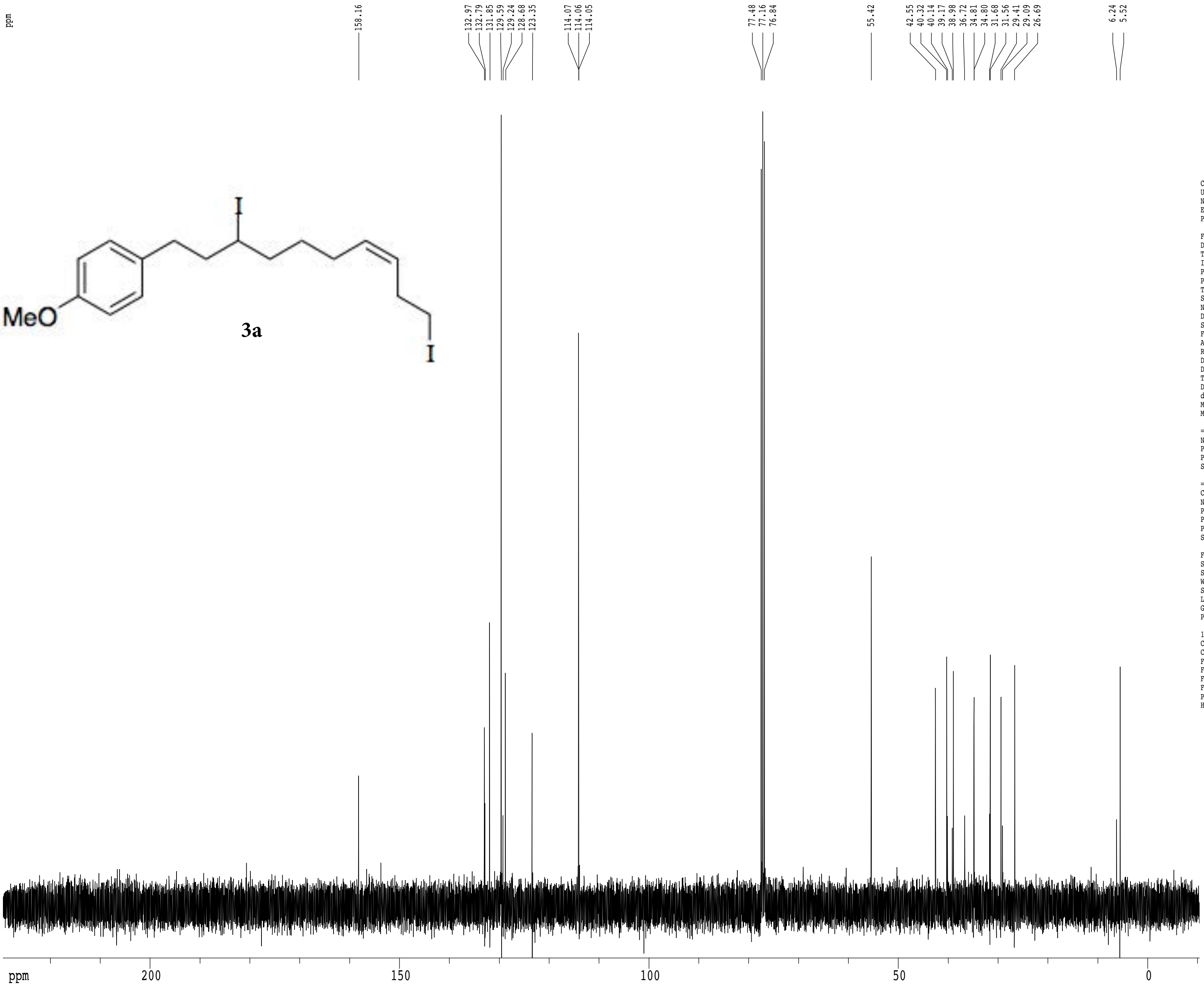


```
Current Data Parameters
USER            khewitt1
NAME     KAH-V-214-greaseextract
EXPNO                  2
PROCNO                 1

F2 - Acquisition Parameters
Date_           20220108
Time               13.37
INSTRUM           drx400
PROBHD   5 mm QNP H/F/P
PULPROG          zgdc30
TD                 65536
SOLVENT            CDCl3
NS                   240
DS                     4
SWH            24154.590 Hz
FIDRES          0.368570 Hz
AQ             1.3566452 sec
RG                9195.2
DW                20.700 usec
DE                 20.39 usec
TE                 298.0 K
D1            0.10000000 sec
d11           0.03000000 sec
MCREST        0.00000000 sec
MCWRK         0.01500000 sec

======== CHANNEL f1 ========
NUC1                 13C
P1                  7.90 usec
PL1                -3.00 dB
SFO1         100.6237964 MHz

======== CHANNEL f2 ========
CPDPRG2          waltz16
NUC2                  1H
PCPD2              90.00 usec
PL2                -0.90 dB
PL12               17.00 dB
SFO2         400.1328009 MHz

F2 - Processing parameters
SI                 65536
SF           100.6127587 MHz
WDW                   no
SSB                    0
LB                  0.00 Hz
GB                     0
PC                  1.00

1D NMR plot parameters
CX                 22.80 cm
CY                 15.50 cm
F1P              229.496 ppm
F1              23090.21 Hz
F2P              -10.579 ppm
F2              -1064.37 Hz
PPMCM           10.52959 ppm/cm
HZCM          1059.41150 Hz/cm
```

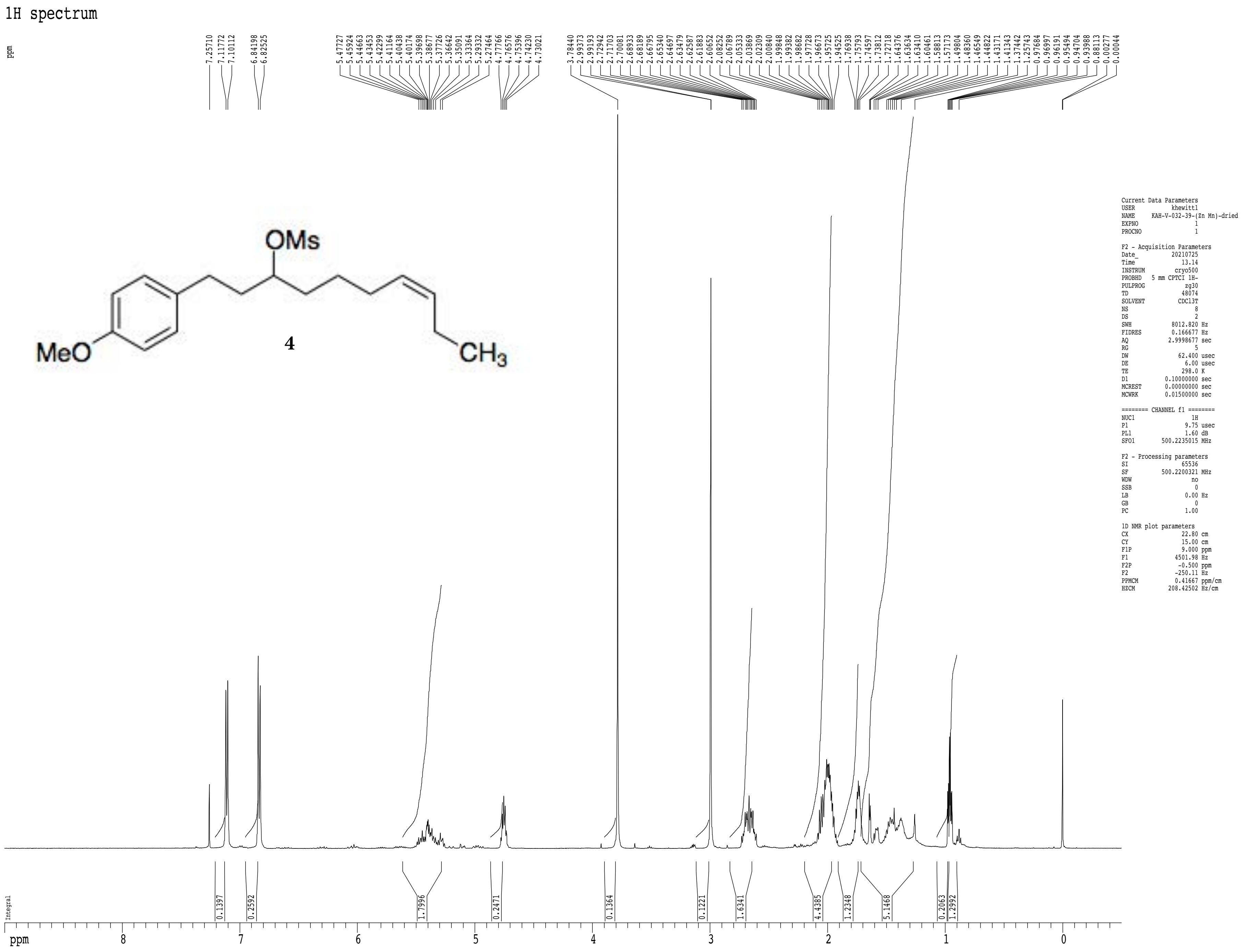
1H spectrum
OMs
MeO
4
CH3
7.25710 7.11772 7.10112 6.84198 6.82525
5.47727 5.45924 5.44663 5.43453 5.42299 5.41164 5.40438 5.40174 5.39698 5.38677 5.37726 5.36642 5.35091 5.33364 5.29332 5.27464 4.77766 4.76576 4.75396 4.74230 4.73021
3.78440 2.99373 2.99193 2.72942 2.71703 2.70081 2.68933 2.68189 2.66795 2.65340 2.64697 2.63479 2.62587 2.61883 2.60652 2.08252 2.06789 2.05333 2.03869 2.02309 2.00840 1.99848 1.99382 1.98682 1.97728 1.96673 1.95725 1.94525 1.76938 1.75793 1.74597 1.73812 1.72718 1.64376 1.63634 1.63410 1.60461 1.58813 1.57173 1.49804 1.48360 1.46549 1.44822 1.43171 1.41343 1.37442 1.25743 0.97684 0.96997 0.96191 0.95494 0.94704 0.93988 0.88113 0.00277 0.00044
Current Data Parameters
USER khewitt1
NAME KAH-V-032-39-(Zn Mn)-dried
EXPNO 1
PROCNO 1
F2 - Acquisition Parameters
Date_ 20210725
Time 13.14
INSTRUM cryo500
PROBHD 5 mm CPTCI 1H-
PULPROG zg30
TD 48074
SOLVENT CDCl3T
NS 8
DS 2
SWH 8012.820 Hz
FIDRES 0.166677 Hz
AQ 2.9998677 sec
RG 5
DW 62.400 usec
DE 6.00 usec
TE 298.0 K
D1 0.10000000 sec
MCREST 0.00000000 sec
MCWRK 0.01500000 sec
======== CHANNEL f1 ========
NUC1 1H
P1 9.75 usec
PL1 1.60 dB
SFO1 500.2235015 MHz
F2 - Processing parameters
SI 65536
SF 500.2200321 MHz
WDW no
SSB 0
LB 0.00 Hz
GB 0
PC 1.00
1D NMR plot parameters
CX 22.80 cm
CY 15.00 cm
F1P 9.000 ppm
F1 4501.98 Hz
F2P -0.500 ppm
F2 -250.11 Hz
PPMCM 0.41667 ppm/cm
HZCM 208.42502 Hz/cm
Integral
0.1397 0.2592 1.7996 0.2471 0.1364 0.1221 1.6341 4.4385 1.2348 5.1468 0.2063 1.2992
ppm
8 7 6 5 4 3 2 1 0

Z-restored spin-echo 13C spectrum with 1H decoupling

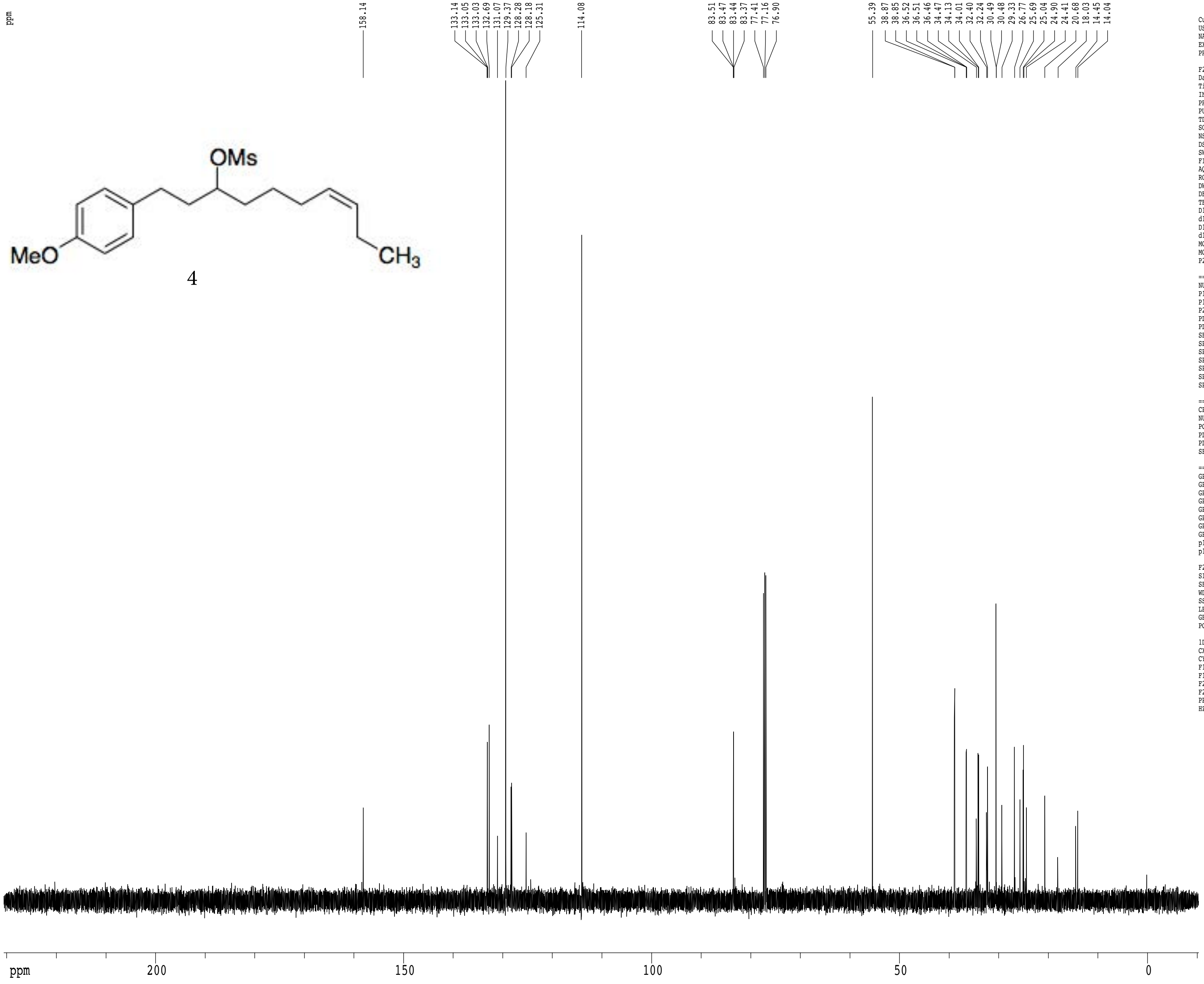


```
Current Data Parameters
USER              khewitt1
NAME     KAH-V-032-39-(Zn Mn)-dried
EXPNO                    2
PROCNO                   1

F2 - Acquisition Parameters
Date_             20210725
Time                 13.17
INSTRUM            cryo500
PROBHD   5 mm CPTCI 1H-
PULPROG  SpinEchopg30gp2.prd
TD                   65536
SOLVENT              CDCl3
NS                     104
DS                      16
SWH              30303.031 Hz
FIDRES            0.462388 Hz
AQ               1.0813940 sec
RG                  7298.2
DW                  16.500 usec
DE                    6.00 usec
TE                   298.0 K
D1              0.25000000 sec
d11             0.03000000 sec
D16             0.00020000 sec
d17             0.00019600 sec
MCREST          0.00000000 sec
MCWRK           0.01500000 sec
P2                   37.70 usec

======== CHANNEL f1 ========
NUC1                   13C
P1                   18.85 usec
P12                2000.00 usec
P20                 500.00 usec
PL0                 120.00 dB
PL1                  -1.00 dB
SFO1           125.7942548 MHz
SP2                   1.55 dB
SP4                   1.55 dB
SPNAM2         Crp60comp.4
SPNAM4      Crp60,0.5,20.1
SPOFF2                0.00 Hz
SPOFF4                0.00 Hz

======== CHANNEL f2 ========
CPDPRG2            waltz16
NUC2                    1H
PCPD2               100.00 usec
PL2                   1.60 dB
PL12                 22.00 dB
SFO2           500.2225011 MHz

====== GRADIENT CHANNEL =====
GPNAM1            SINE.100
GPNAM2            SINE.100
GPX1                  0.00 %
GPX2                  0.00 %
GPY1                  0.00 %
GPY2                  0.00 %
GPZ1                 30.00 %
GPZ2                 50.00 %
p15                 500.00 usec
p16                1000.00 usec

F2 - Processing parameters
SI                   65536
SF             125.7804090 MHz
WDW                     no
SSB                      0
LB                    0.00 Hz
GB                       0
PC                    2.00

1D NMR plot parameters
CX                   22.80 cm
CY                   15.65 cm
F1P                230.637 ppm
F1                29009.68 Hz
F2P                -10.287 ppm
F2                -1293.96 Hz
PPMCM             10.56688 ppm/cm
HZCM            1329.10693 Hz/cm
```

1H spectrum

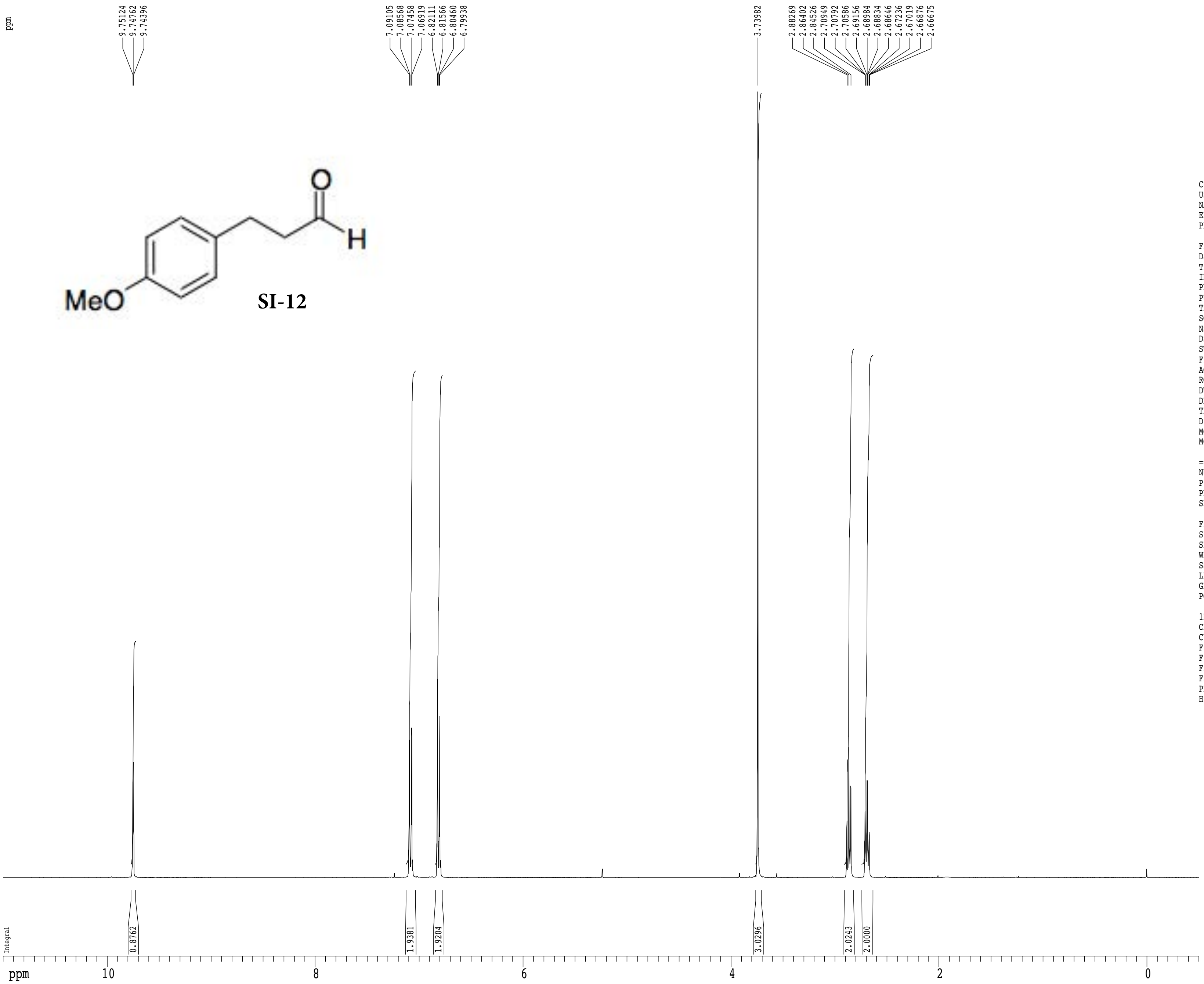

SI-12

Current Data Parameters
USER khewitt1
NAME KAH-V-054-1
EXPNO 1
PROCNO 1

F2 - Acquisition Parameters
Date_ 20210805
Time 15.42
INSTRUM drx400
PROBHD 5 mm QNP H/F/P
PULPROG zg30
TD 38460
SOLVENT CDCl3T
NS 8
DS 2
SWH 6410.256 Hz
FIDRES 0.166673 Hz
AQ 2.9999299 sec
RG 32
DW 78.000 usec
DE 4.50 usec
TE 298.0 K
D1 0.10000000 sec
MCREST 0.00000000 sec
MCWRK 0.01500000 sec

======== CHANNEL f1 ========
NUC1 1H
P1 12.00 usec
PL1 -1.60 dB
SFO1 400.1328009 MHz

F2 - Processing parameters
SI 65536
SF 400.1300301 MHz
WDW no
SSB 0
LB 0.00 Hz
GB 0
PC 2.00

1D NMR plot parameters
CX 22.80 cm
CY 15.00 cm
F1P 11.000 ppm
F1 4401.43 Hz
F2P -0.500 ppm
F2 -200.07 Hz
PPMCM 0.50439 ppm/cm
HZCM 201.81998 Hz/cm

1H spectrum

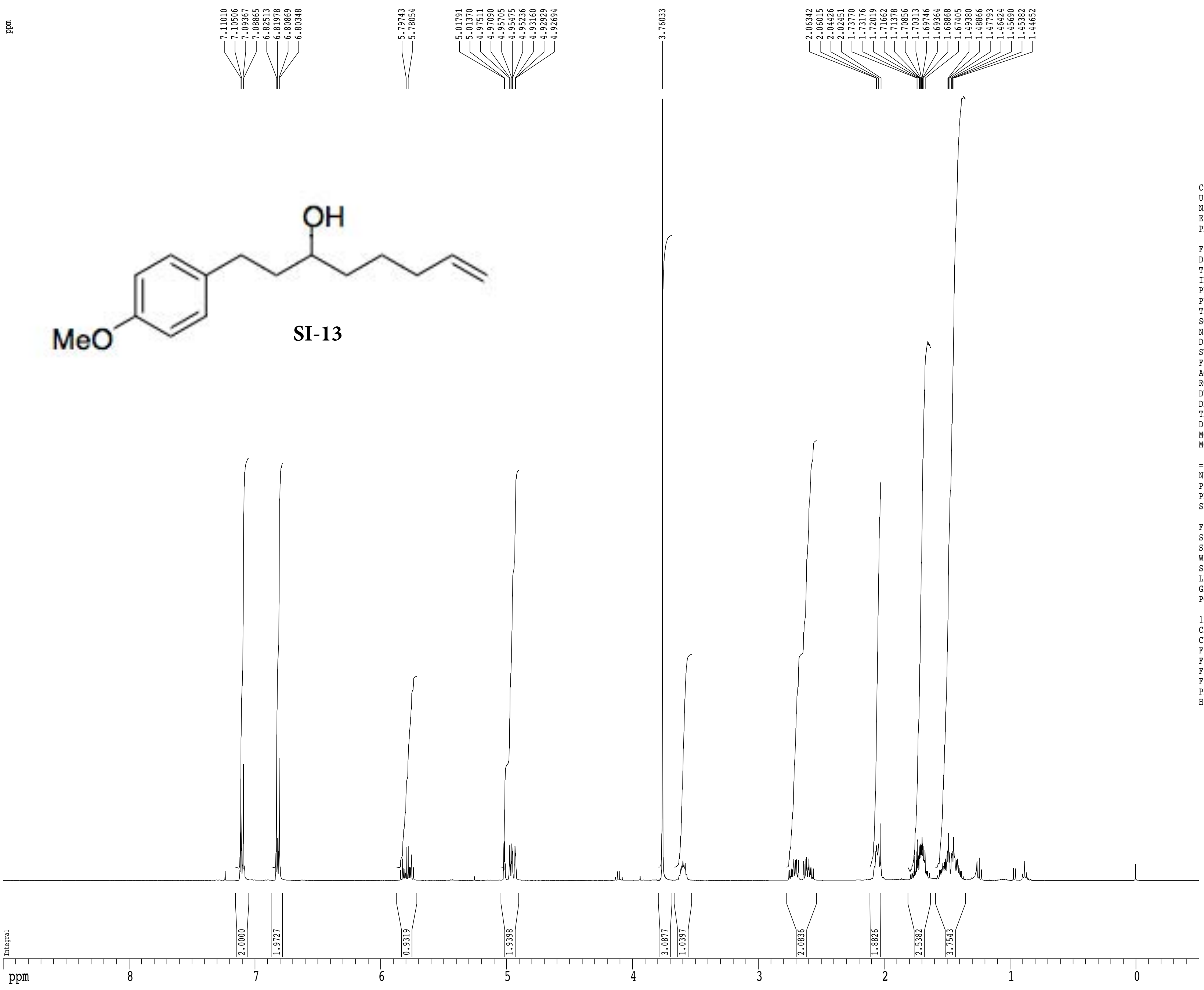

Current Data Parameters
USER khewitt1
NAME KAH-V-057-1
EXPNO 1
PROCNO 1

F2 - Acquisition Parameters
Date_ 20210806
Time 17.51
INSTRUM drx400
PROBHD 5 mm QNP H/F/P
PULPROG zg30
TD 38460
SOLVENT CDCl3T
NS 8
DS 2
SWH 6410.256 Hz
FIDRES 0.166673 Hz
AQ 2.9999299 sec
RG 32
DW 78.000 usec
DE 4.50 usec
TE 298.0 K
D1 0.10000000 sec
MCREST 0.00000000 sec
MCWRK 0.01500000 sec

======== CHANNEL f1 ========
NUC1 1H
P1 12.00 usec
PL1 -1.60 dB
SFO1 400.1328009 MHz

F2 - Processing parameters
SI 65536
SF 400.1300311 MHz
WDW no
SSB 0
LB 0.00 Hz
GB 0
PC 2.00

1D NMR plot parameters
CX 22.80 cm
CY 15.00 cm
F1P 9.000 ppm
F1 3601.17 Hz
F2P -0.500 ppm
F2 -200.06 Hz
PPMCM 0.41667 ppm/cm
HZCM 166.72086 Hz/cm

13C spectrum with 1H decoupling

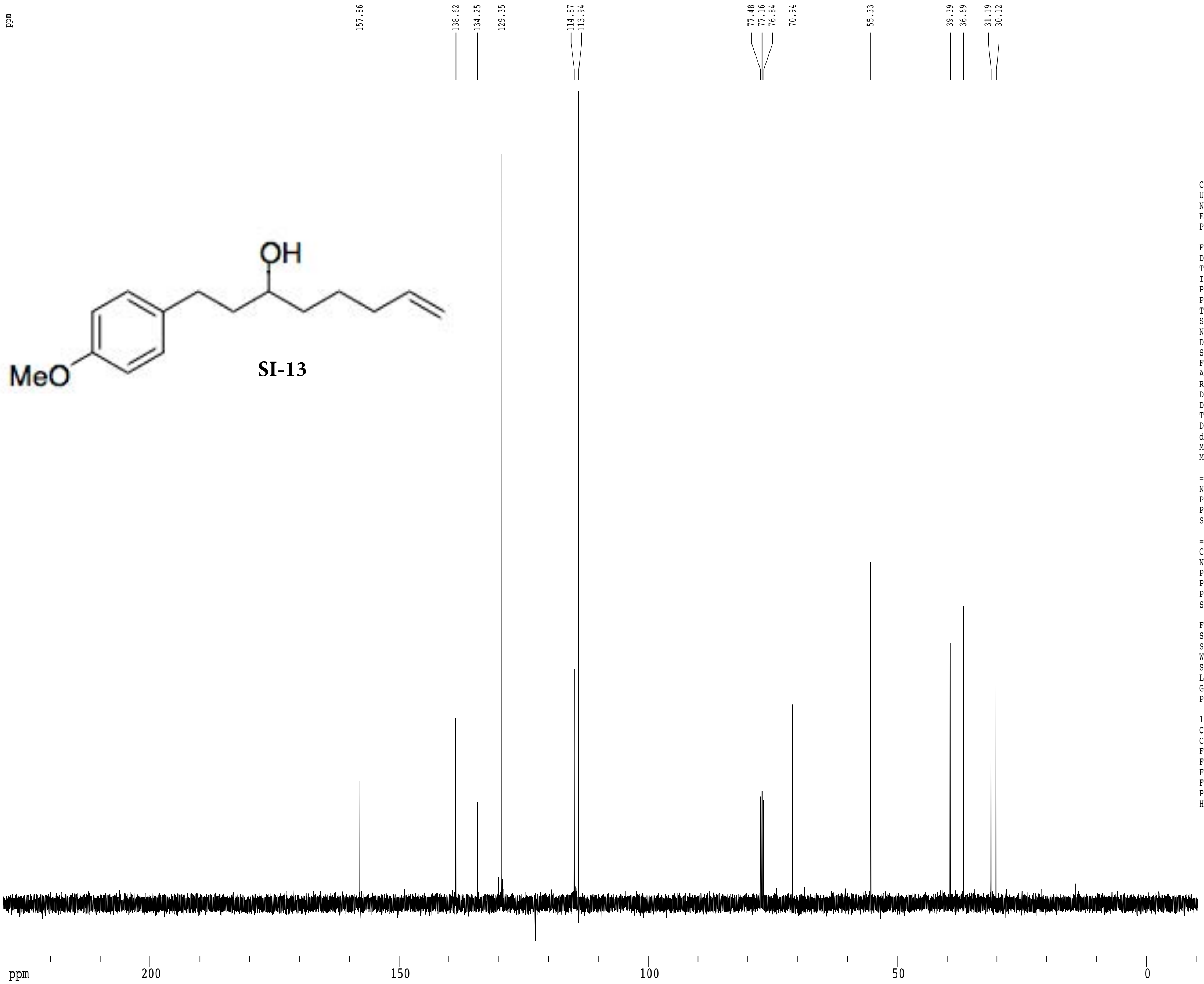

SI-13

```
Current Data Parameters
USER          khewitt1
NAME        KAH-V-248-1
EXPNO                2
PROCNO               1

F2 - Acquisition Parameters
Date_         20220126
Time             18.37
INSTRUM         drx400
PROBHD   5 mm QNP H/F/P
PULPROG        zgdc30
TD               65536
SOLVENT          CDCl3
NS                  64
DS                   4
SWH          24154.590 Hz
FIDRES        0.368570 Hz
AQ           1.3566452 sec
RG              9195.2
DW              20.700 usec
DE               20.39 usec
TE               298.0 K
D1          0.10000000 sec
d11         0.03000000 sec
MCREST      0.00000000 sec
MCWRK       0.01500000 sec

======== CHANNEL f1 ========
NUC1               13C
P1                7.90 usec
PL1              -3.00 dB
SFO1       100.6237964 MHz

======== CHANNEL f2 ========
CPDPRG2        waltz16
NUC2                1H
PCPD2            90.00 usec
PL2              -0.90 dB
PL12             17.00 dB
SFO2       400.1328009 MHz

F2 - Processing parameters
SI               65536
SF         100.6127658 MHz
WDW                 no
SSB                  0
LB                0.00 Hz
GB                   0
PC                1.00

1D NMR plot parameters
CX               22.80 cm
CY               15.50 cm
F1P            229.496 ppm
F1            23090.21 Hz
F2P            -10.579 ppm
F2            -1064.37 Hz
PPMCM         10.52959 ppm/cm
HZCM        1059.41150 Hz/cm
```

1H spectrum

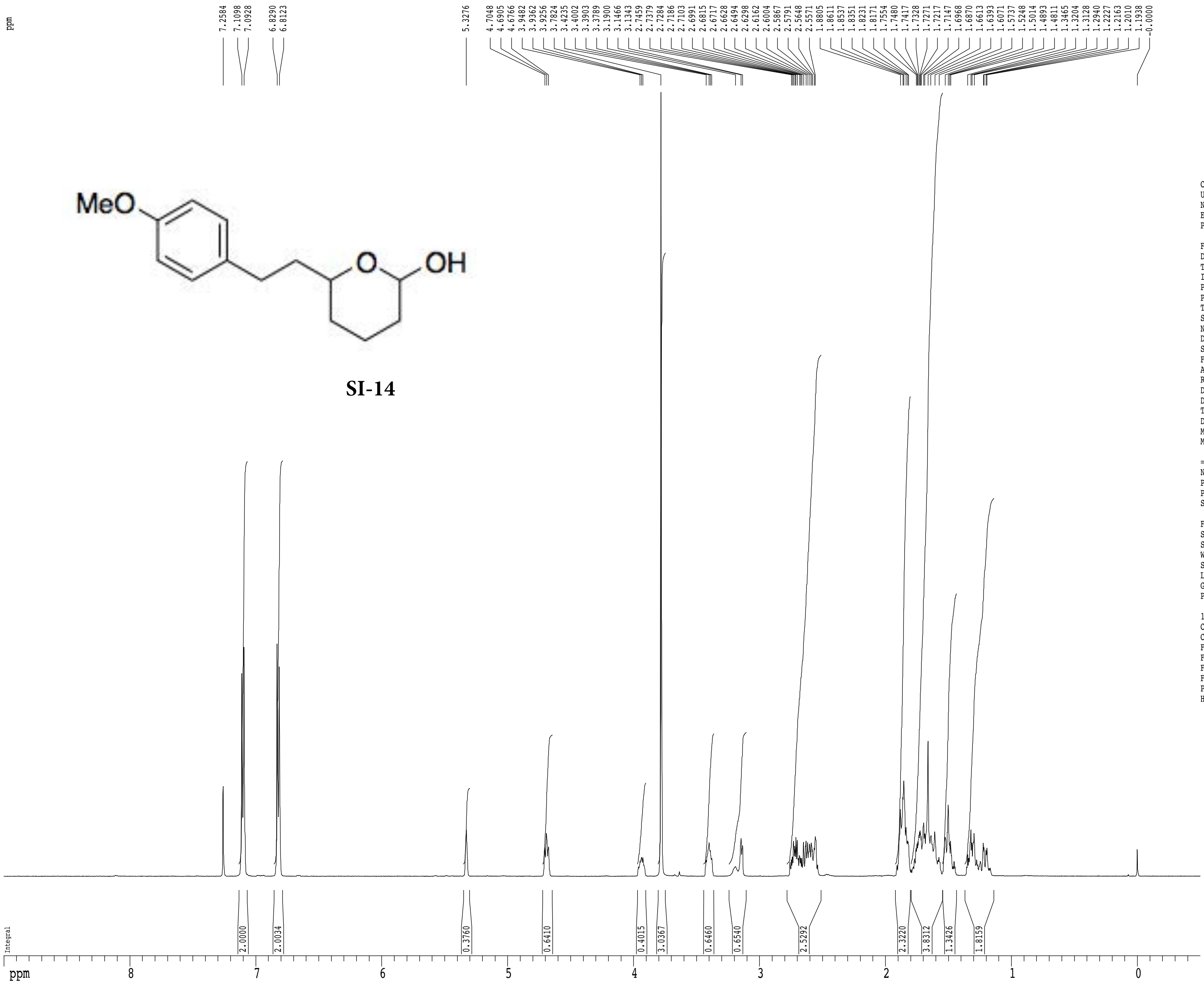

SI-14

```
Current Data Parameters
USER          khewitt1
NAME      KAH-IV-286-Z
EXPNO                1
PROCNO               1

F2 - Acquisition Parameters
Date_         20210616
Time             16.13
INSTRUM        cryo500
PROBHD   5 mm CPTCI 1H-
PULPROG           zg30
TD               48074
SOLVENT         CDCl3T
NS                   8
DS                   2
SWH           8012.820 Hz
FIDRES        0.166677 Hz
AQ           2.9998677 sec
RG                 6.3
DW              62.400 usec
DE                6.00 usec
TE               298.0 K
D1          0.10000000 sec
MCREST      0.00000000 sec
MCWRK       0.01500000 sec

======== CHANNEL f1 ========
NUC1                1H
P1                9.75 usec
PL1               1.60 dB
SFO1       500.2235015 MHz

F2 - Processing parameters
SI               65536
SF         500.2200321 MHz
WDW                 no
SSB                  0
LB                0.00 Hz
GB                   0
PC                1.00

1D NMR plot parameters
CX               22.80 cm
CY               15.00 cm
F1P              9.000 ppm
F1             4501.98 Hz
F2P             -0.500 ppm
F2             -250.11 Hz
PPMCM          0.41667 ppm/cm
HZCM         208.42502 Hz/cm
```

Z-restored spin-echo 13C spectrum with 1H decoupling

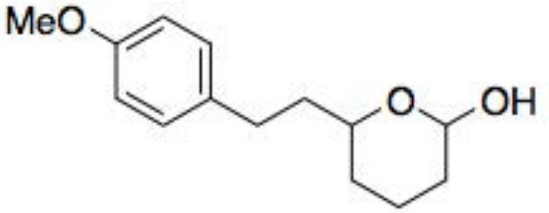

SI-14

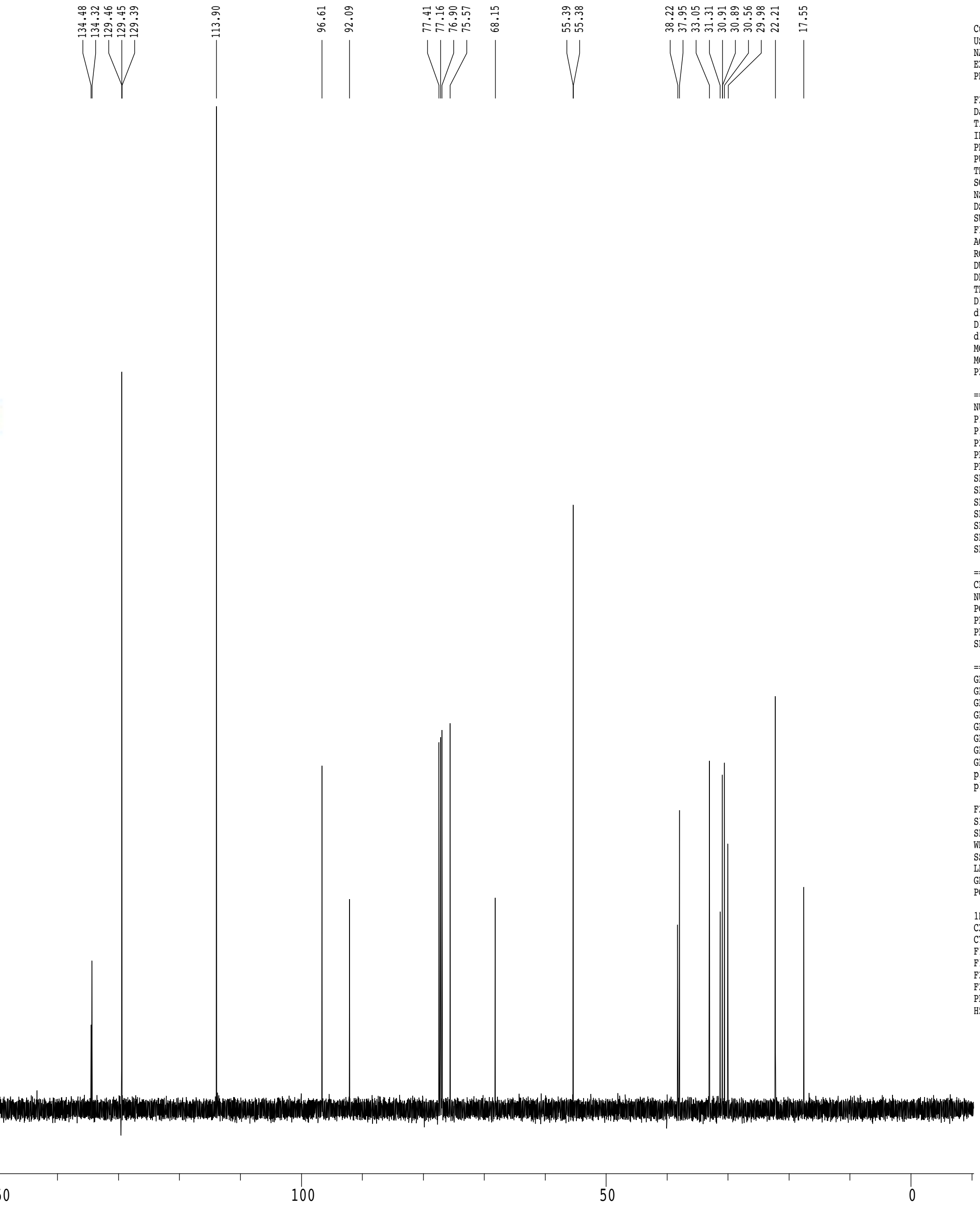


```
Current Data Parameters
USER          khewitt1
NAME      KAH-IV-286-Z
EXPNO                2
PROCNO               1

F2 - Acquisition Parameters
Date_         20210616
Time             16.15
INSTRUM        cryo500
PROBHD   5 mm CPTCI 1H-
PULPROG  SpinEchopg30gp2.prd
TD               65536
SOLVENT          CDCl3
NS                 144
DS                  16
SWH          30303.031 Hz
FIDRES        0.462388 Hz
AQ           1.0813940 sec
RG              7298.2
DW              16.500 usec
DE                6.00 usec
TE               298.0 K
D1          0.25000000 sec
d11         0.03000000 sec
D16         0.00020000 sec
d17         0.00019600 sec
MCREST      0.00000000 sec
MCWRK       0.01500000 sec
P2               37.70 usec

======== CHANNEL f1 ========
NUC1               13C
P1               18.85 usec
P12            2000.00 usec
P20             500.00 usec
PL0             120.00 dB
PL1              -1.00 dB
SFO1       125.7942548 MHz
SP2               1.55 dB
SP4               1.55 dB
SPNAM2      Crp60comp.4
SPNAM4   Crp60,0.5,20.1
SPOFF2            0.00 Hz
SPOFF4            0.00 Hz

======== CHANNEL f2 ========
CPDPRG2        waltz16
NUC2                1H
PCPD2           100.00 usec
PL2               1.60 dB
PL12             22.00 dB
SFO2       500.2225011 MHz

====== GRADIENT CHANNEL =====
GPNAM1        SINE.100
GPNAM2        SINE.100
GPX1              0.00 %
GPX2              0.00 %
GPY1              0.00 %
GPY2              0.00 %
GPZ1             30.00 %
GPZ2             50.00 %
p15             500.00 usec
p16            1000.00 usec

F2 - Processing parameters
SI               65536
SF         125.7804090 MHz
WDW                 no
SSB                  0
LB                0.00 Hz
GB                   0
PC                2.00

1D NMR plot parameters
CX               22.80 cm
CY               15.65 cm
F1P            230.637 ppm
F1            29009.68 Hz
F2P            -10.287 ppm
F2            -1293.96 Hz
PPMCM         10.56688 ppm/cm
HZCM        1329.10693 Hz/cm
```

1H spectrum

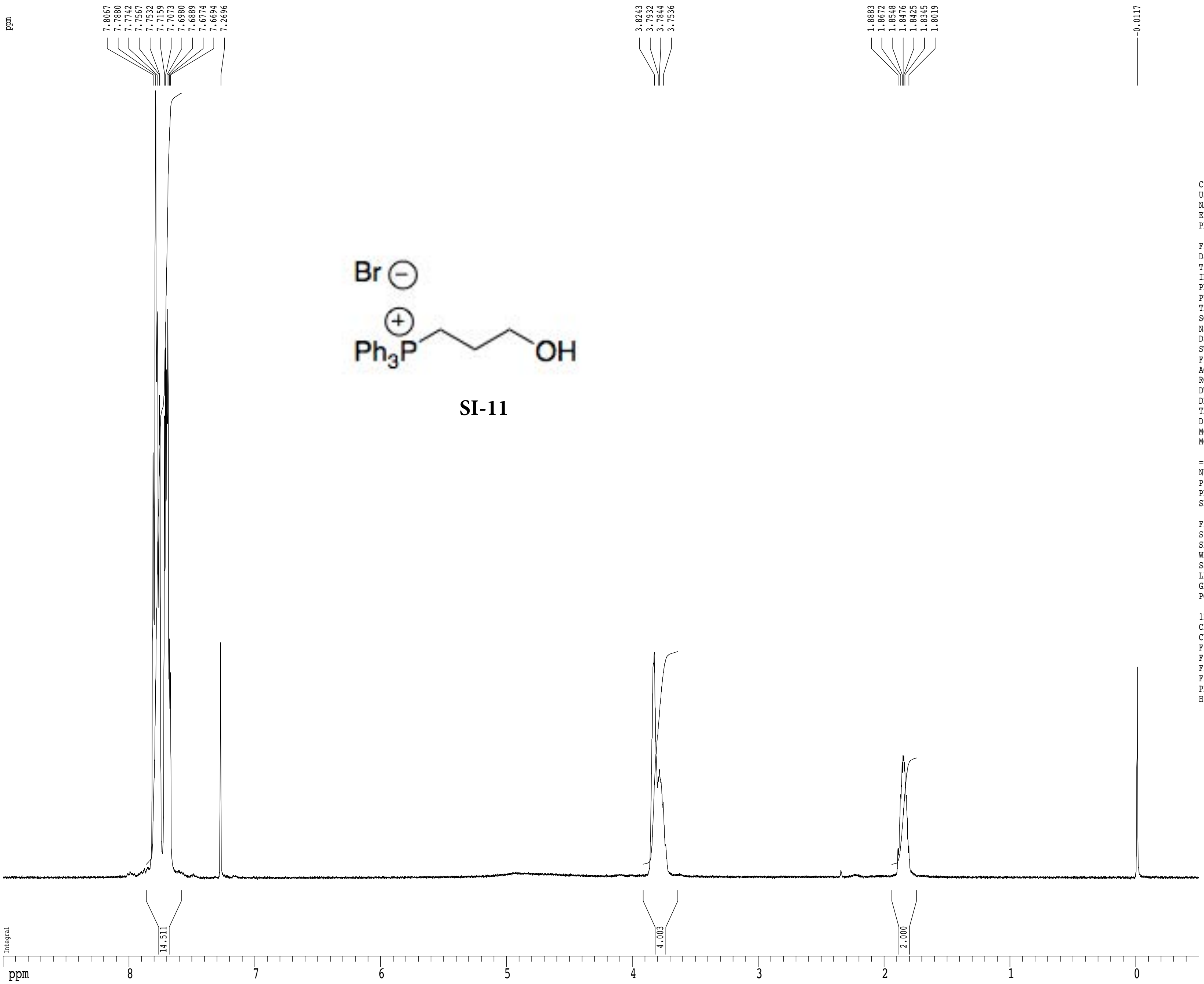

SI-11

```
Current Data Parameters
USER          khewitt1
NAME       KAH-IV-290-1
EXPNO                 1
PROCNO                1

F2 - Acquisition Parameters
Date_          20210622
Time              14.37
INSTRUM          drx400
PROBHD   5 mm QNP H/F/P
PULPROG            zg30
TD                38460
SOLVENT          CDCl3T
NS                    8
DS                    2
SWH            6410.256 Hz
FIDRES         0.166673 Hz
AQ            2.9999299 sec
RG                  362
DW               78.000 usec
DE                 4.50 usec
TE                298.0 K
D1           0.10000000 sec
MCREST       0.00000000 sec
MCWRK        0.01500000 sec

======== CHANNEL f1 ========
NUC1                 1H
P1                12.00 usec
PL1               -1.60 dB
SFO1        400.1328009 MHz

F2 - Processing parameters
SI                65536
SF          400.1300175 MHz
WDW                  no
SSB                   0
LB                 0.00 Hz
GB                    0
PC                 2.00

1D NMR plot parameters
CX                22.80 cm
CY                15.00 cm
F1P               9.000 ppm
F1              3601.17 Hz
F2P              -0.500 ppm
F2              -200.06 Hz
PPMCM           0.41667 ppm/cm
HZCM          166.72084 Hz/cm
```

1H spectrum

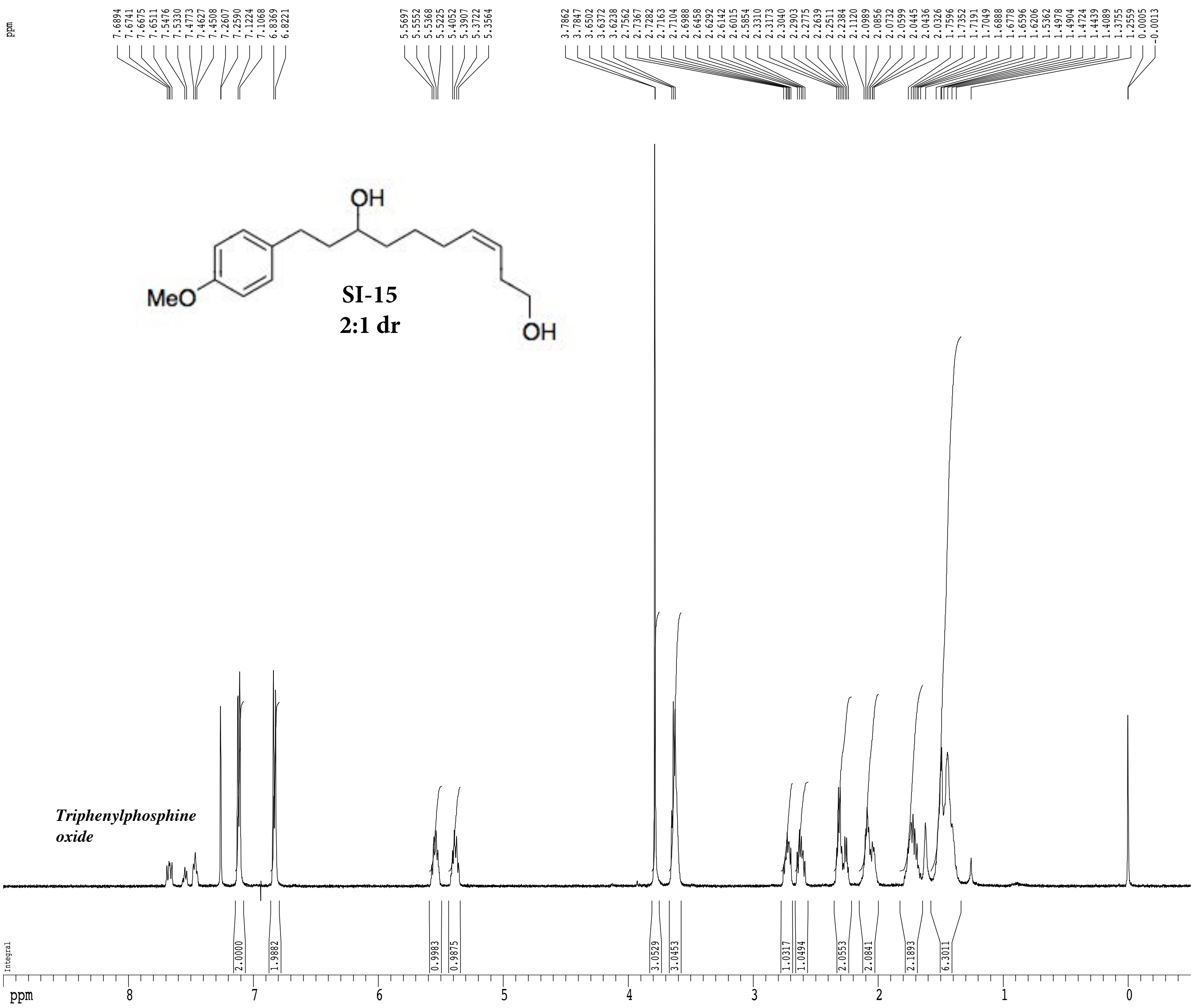


```
Current Data Parameters
USER             khewitt1
NAME        KAH-IV-288-chk
EXPNO                   1
PROCNO                  1

F2 - Acquisition Parameters
Date_            20210621
Time                14.21
INSTRUM             gn500
PROBHD   5 mm broadband
PULPROG              zg30
TD                  48074
SOLVENT            CDCl3T
NS                      8
DS                      2
SWH             8012.820 Hz
FIDRES          0.166677 Hz
AQ             2.9998677 sec
RG                 1448.2
DW                 62.400 usec
DE                   6.00 usec
TE                  298.0 K
D1            0.10000000 sec
MCREST        0.00000000 sec
MCWRK         0.01500000 sec

======== CHANNEL f1 ========
NUC1                   1H
P1                  12.00 usec
PL1                 -6.00 dB
SFO1          498.7534913 MHz

F2 - Processing parameters
SI                  65536
SF            498.7500314 MHz
WDW                    no
SSB                     0
LB                   0.00 Hz
GB                      0
PC                   1.00

1D NMR plot parameters
CX                  20.00 cm
CY                  12.50 cm
F1P                 9.000 ppm
F1                4488.75 Hz
F2P                -0.500 ppm
F2                -249.38 Hz
PPMCM             0.47500 ppm/cm
HZCM            236.90627 Hz/cm
```

13C spectrum with 1H decoupling

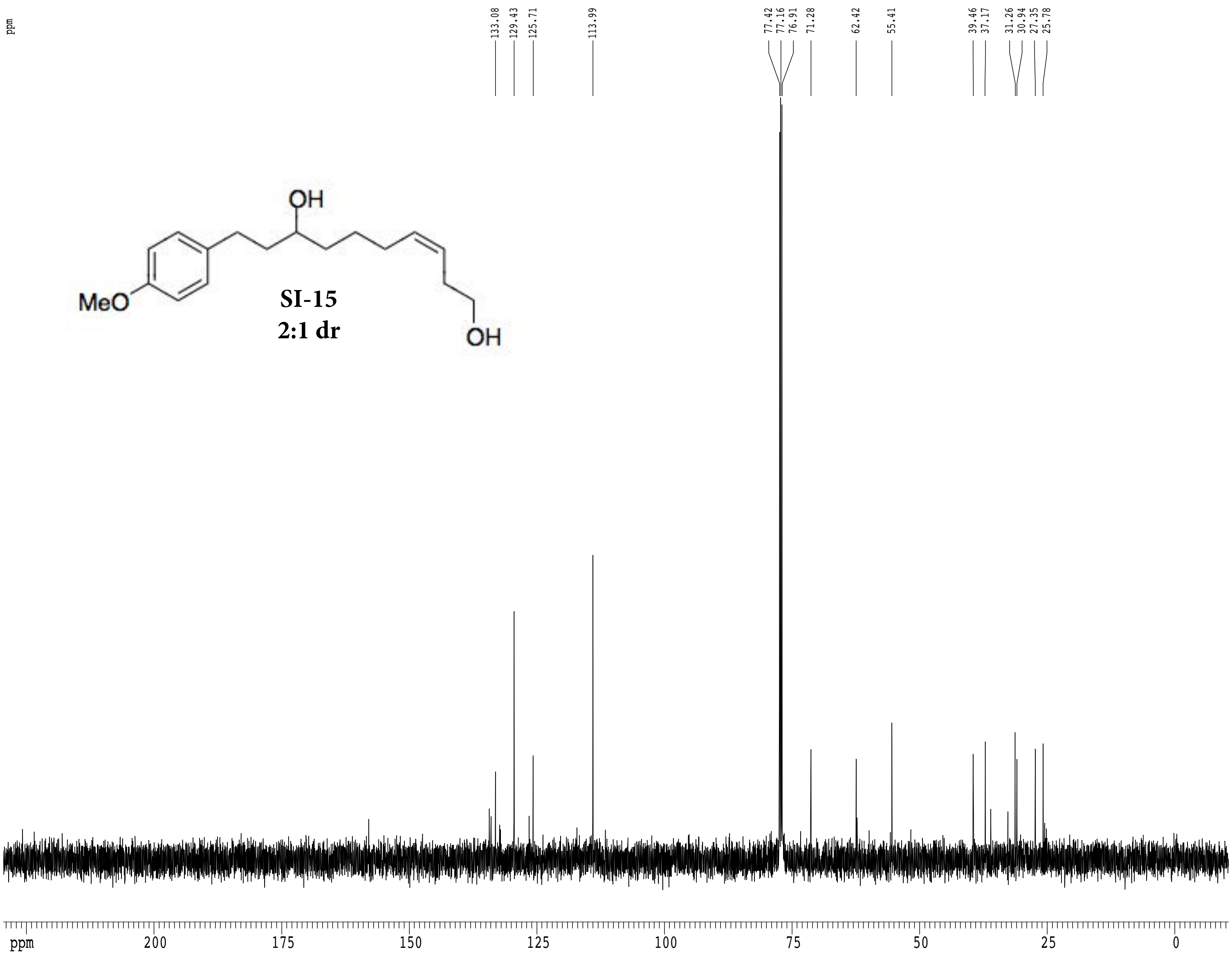

Current Data Parameters
USER khewitt1
NAME KAH-IV-288-chk
EXPNO 2
PROCNO 1

F2 - Acquisition Parameters
Date_ 20210621
Time 14.23
INSTRUM gn500
PROBHD 5 mm broadband
PULPROG zgdc30
TD 65536
SOLVENT CDCl3
NS 144
DS 4
SWH 30303.031 Hz
FIDRES 0.462388 Hz
AQ 1.0813940 sec
RG 5792.6
DW 16.500 usec
DE 6.00 usec
TE 298.0 K
D1 0.25000000 sec
d11 0.03000000 sec
MCREST 0.00000000 sec
MCWRK 0.01500000 sec

======== CHANNEL f1 ========
NUC1 13C
P1 14.20 usec
PL1 -6.00 dB
SFO1 125.4245824 MHz

======== CHANNEL f2 ========
CPDPRG2 waltz16
NUC2 1H
PCPD2 80.00 usec
PL2 -6.00 dB
PL12 12.30 dB
SFO2 498.7524937 MHz

F2 - Processing parameters
SI 65536
SF 125.4107757 MHz
WDW EM
SSB 0
LB 1.00 Hz
GB 0
PC 2.00

1D NMR plot parameters
CX 20.00 cm
CY 12.50 cm
F1P 229.520 ppm
F1 28784.24 Hz
F2P -10.507 ppm
F2 -1317.64 Hz
PPMCM 12.00131 ppm/cm
HZCM 1505.09399 Hz/cm

1H spectrum

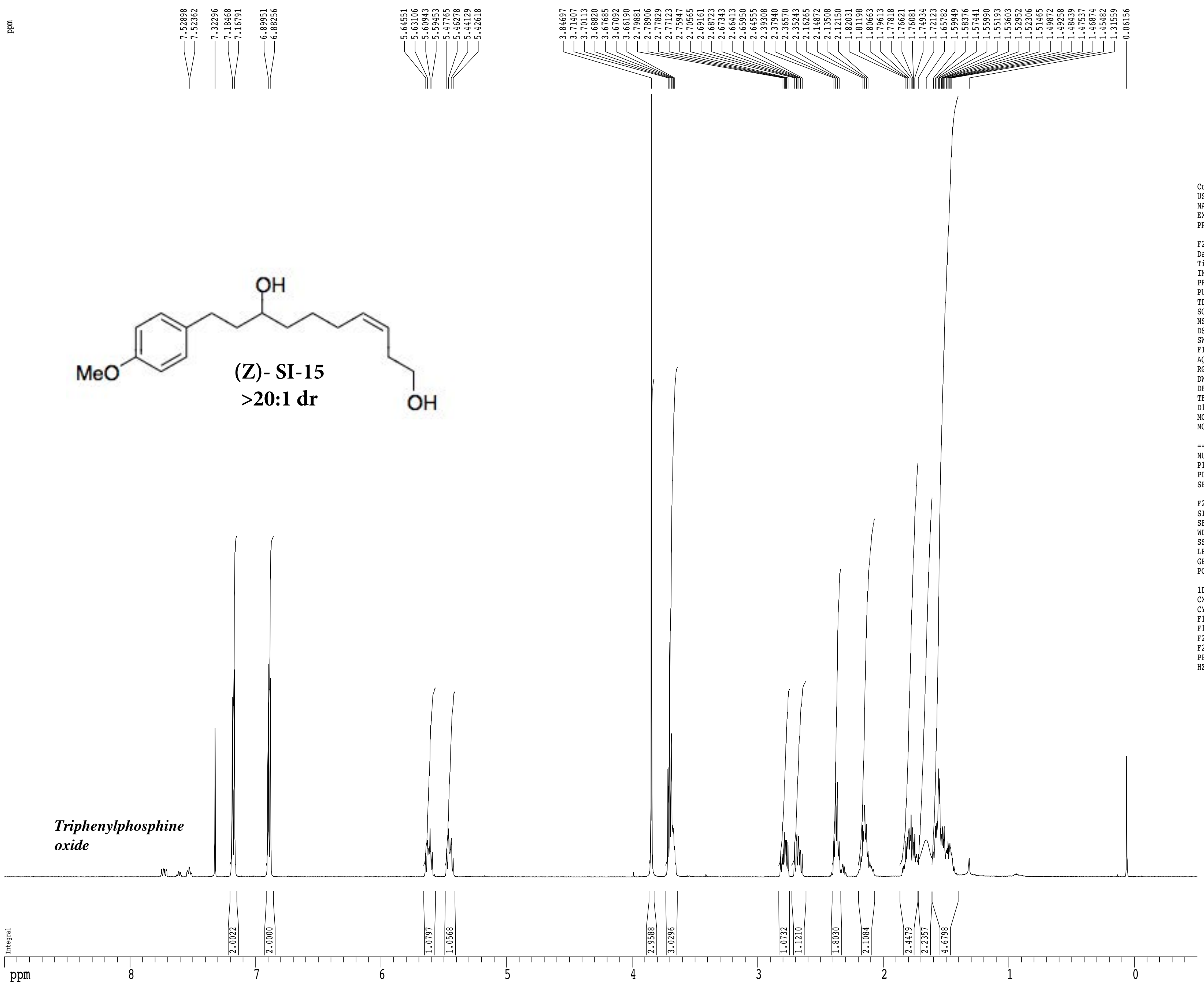

(Z)- SI-15
>20:1 dr

```
Current Data Parameters
USER            khewitt1
NAME     KAH-V-155-major-Z-Z
EXPNO                  1
PROCNO                 1

F2 - Acquisition Parameters
Date_           20211105
Time               11.39
INSTRUM          cryo500
PROBHD   5 mm CPTCI 1H-
PULPROG             zg30
TD                 48074
SOLVENT           CDCl3T
NS                     8
DS                     2
SWH            8012.820 Hz
FIDRES         0.166677 Hz
AQ            2.9998677 sec
RG                   7.1
DW               62.400 usec
DE                 6.00 usec
TE                298.0 K
D1           0.10000000 sec
MCREST       0.00000000 sec
MCWRK        0.01500000 sec

======== CHANNEL f1 ========
NUC1                  1H
P1                  9.75 usec
PL1                 1.60 dB
SFO1        500.2235015 MHz

F2 - Processing parameters
SI                 65536
SF           500.2200000 MHz
WDW                   no
SSB                    0
LB                  0.00 Hz
GB                     0
PC                  1.00

1D NMR plot parameters
CX                 22.80 cm
CY                 15.00 cm
F1P                9.000 ppm
F1               4501.98 Hz
F2P               -0.500 ppm
F2               -250.11 Hz
PPMCM            0.41667 ppm/cm
HZCM           208.42500 Hz/cm
```

Z-restored spin-echo 13C spectrum with 1H decoupling

(Z)- SI-15
>20:1 dr

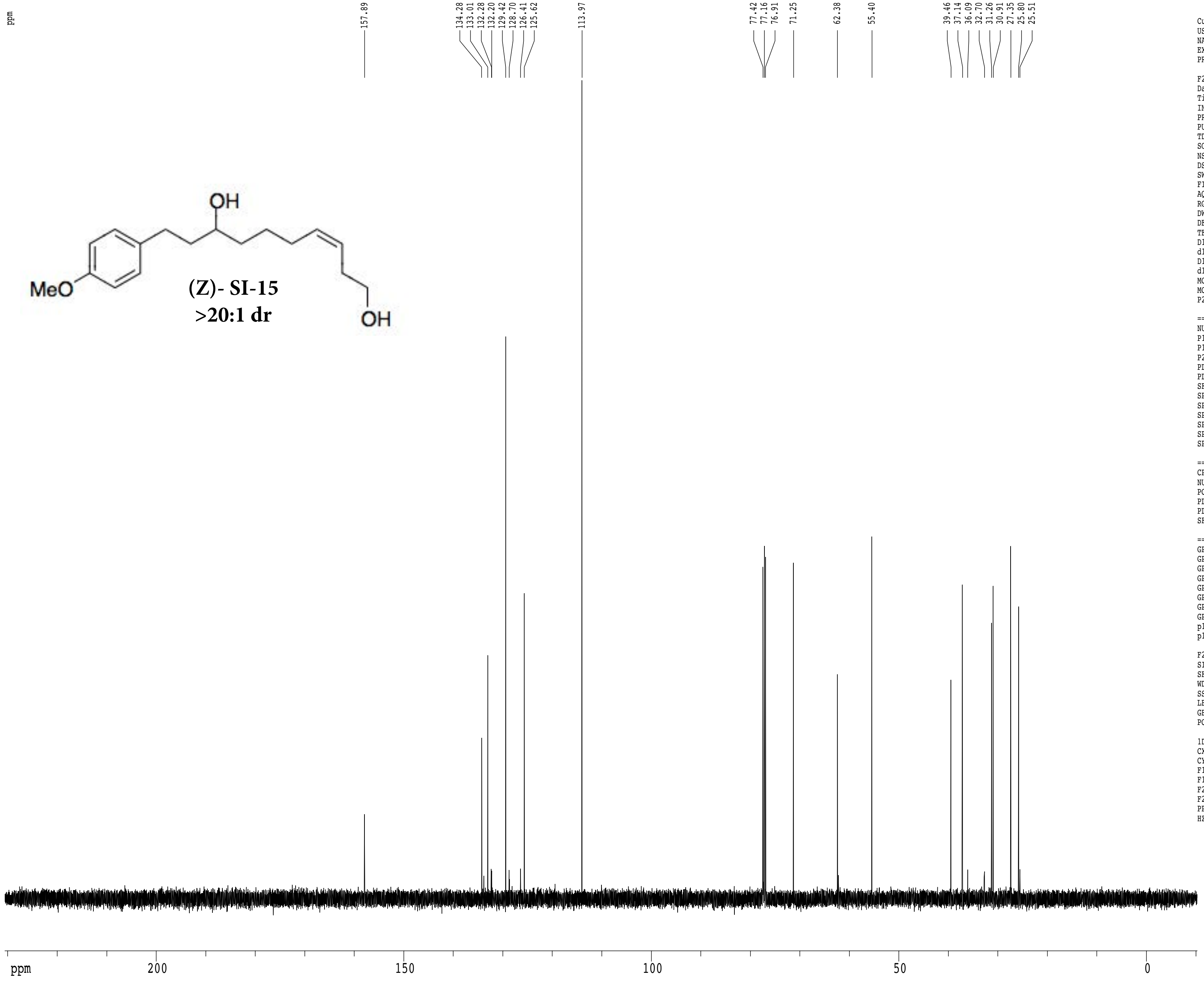


```
Current Data Parameters
USER          khewitt1
NAME     KAH-V-155-major-Z-Z
EXPNO                2
PROCNO               1

F2 - Acquisition Parameters
Date_          20211105
Time              11.42
INSTRUM         cryo500
PROBHD   5 mm CPTCI 1H-
PULPROG  SpinEchopg30gp2.prd
TD                65536
SOLVENT           CDCl3
NS                  184
DS                   16
SWH           30303.031 Hz
FIDRES         0.462388 Hz
AQ            1.0813940 sec
RG               1824.6
DW               16.500 usec
DE                 6.00 usec
TE                298.0 K
D1           0.25000000 sec
d11          0.03000000 sec
D16          0.00020000 sec
d17          0.00019600 sec
MCREST       0.00000000 sec
MCWRK        0.01500000 sec
P2                37.70 usec

======== CHANNEL f1 ========
NUC1                13C
P1                18.85 usec
P12             2000.00 usec
P20              500.00 usec
PL0              120.00 dB
PL1               -1.00 dB
SFO1        125.7942548 MHz
SP2                1.55 dB
SP4                1.55 dB
SPNAM2      Crp60comp.4
SPNAM4   Crp60,0.5,20.1
SPOFF2             0.00 Hz
SPOFF4             0.00 Hz

======== CHANNEL f2 ========
CPDPRG2         waltz16
NUC2                 1H
PCPD2            100.00 usec
PL2                1.60 dB
PL12              22.00 dB
SFO2        500.2225011 MHz

====== GRADIENT CHANNEL =====
GPNAM1         SINE.100
GPNAM2         SINE.100
GPX1               0.00 %
GPX2               0.00 %
GPY1               0.00 %
GPY2               0.00 %
GPZ1              30.00 %
GPZ2              50.00 %
p15              500.00 usec
p16             1000.00 usec

F2 - Processing parameters
SI                65536
SF          125.7804090 MHz
WDW                  no
SSB                   0
LB                 0.00 Hz
GB                    0
PC                 2.00

1D NMR plot parameters
CX                22.80 cm
CY                15.65 cm
F1P             230.637 ppm
F1             29009.68 Hz
F2P             -10.287 ppm
F2             -1293.96 Hz
PPMCM          10.56688 ppm/cm
HZCM         1329.10693 Hz/cm
```

1H spectrum

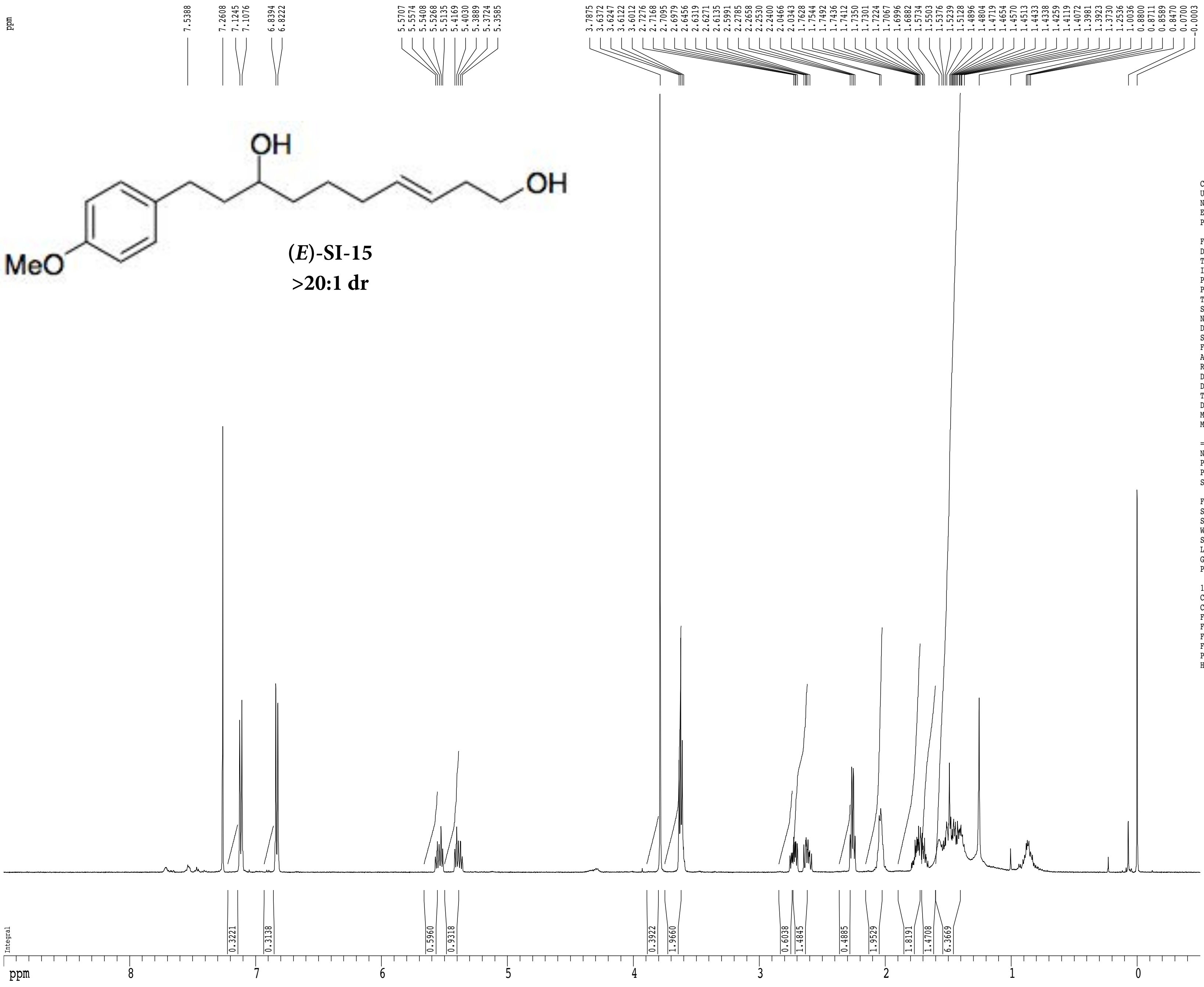

(*E*)-SI-15
>20:1 dr

```
Current Data Parameters
USER            khewitt1
NAME     KAH-V-155-minor-E-Z
EXPNO                  1
PROCNO                 1

F2 - Acquisition Parameters
Date_           20211105
Time               11.47
INSTRUM          cryo500
PROBHD   5 mm CPTCI 1H-
PULPROG             zg30
TD                 48074
SOLVENT          CDCl3T
NS                     8
DS                     2
SWH           8012.820 Hz
FIDRES        0.166677 Hz
AQ           2.9998677 sec
RG                     8
DW              62.400 usec
DE                6.00 usec
TE               298.0 K
D1          0.10000000 sec
MCREST      0.00000000 sec
MCWRK       0.01500000 sec

======== CHANNEL f1 ========
NUC1                  1H
P1                  9.75 usec
PL1                 1.60 dB
SFO1        500.2235015 MHz

F2 - Processing parameters
SI                 65536
SF           500.2200309 MHz
WDW                   no
SSB                    0
LB                  0.00 Hz
GB                     0
PC                  1.00

1D NMR plot parameters
CX                 22.80 cm
CY                 15.00 cm
F1P                9.000 ppm
F1               4501.98 Hz
F2P               -0.500 ppm
F2               -250.11 Hz
PPMCM            0.41667 ppm/cm
HZCM           208.42502 Hz/cm
```

Z-restored spin-echo 13C spectrum with 1H decoupling

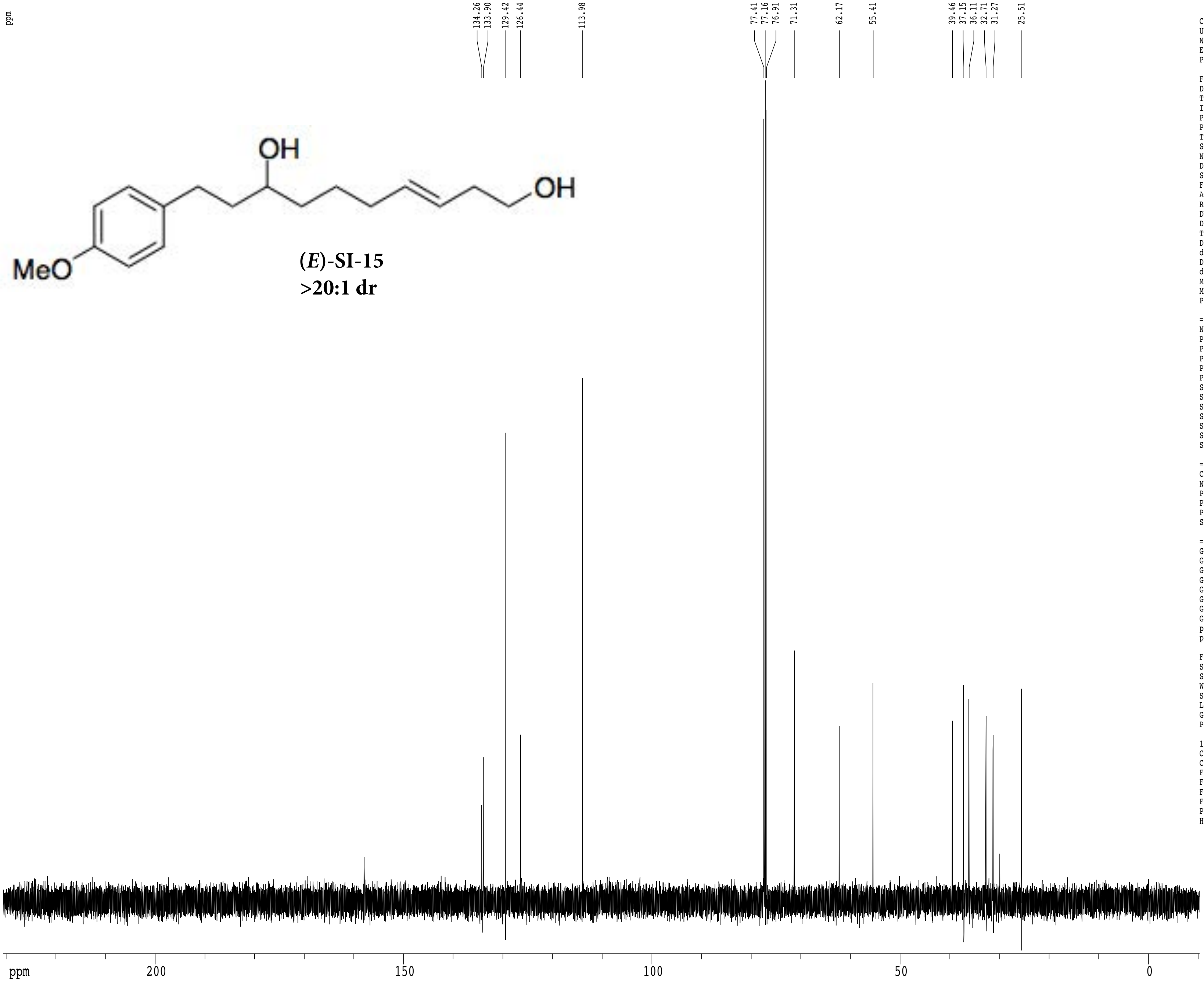

(*E*)-SI-15
>20:1 dr

Current Data Parameters
USER khewitt1
NAME KAH-V-155-minor-E-Z
EXPNO 2
PROCNO 1

F2 - Acquisition Parameters
Date_ 20211105
Time 11.50
INSTRUM cryo500
PROBHD 5 mm CPTCI 1H-
PULPROG SpinEchopg30gp2.prd
TD 65536
SOLVENT CDCl3
NS 280
DS 16
SWH 30303.031 Hz
FIDRES 0.462388 Hz
AQ 1.0813940 sec
RG 2298.8
DW 16.500 usec
DE 6.00 usec
TE 298.0 K
D1 0.25000000 sec
d11 0.03000000 sec
D16 0.00020000 sec
d17 0.00019600 sec
MCREST 0.00000000 sec
MCWRK 0.01500000 sec
P2 37.70 usec

======== CHANNEL f1 ========
NUC1 13C
P1 18.85 usec
P12 2000.00 usec
P20 500.00 usec
PL0 120.00 dB
PL1 -1.00 dB
SFO1 125.7942548 MHz
SP2 1.55 dB
SP4 1.55 dB
SPNAM2 Crp60comp.4
SPNAM4 Crp60,0.5,20.1
SPOFF2 0.00 Hz
SPOFF4 0.00 Hz

======== CHANNEL f2 ========
CPDPRG2 waltz16
NUC2 1H
PCPD2 100.00 usec
PL2 1.60 dB
PL12 22.00 dB
SFO2 500.2225011 MHz

====== GRADIENT CHANNEL =====
GPNAM1 SINE.100
GPNAM2 SINE.100
GPX1 0.00 %
GPX2 0.00 %
GPY1 0.00 %
GPY2 0.00 %
GPZ1 30.00 %
GPZ2 50.00 %
p15 500.00 usec
p16 1000.00 usec

F2 - Processing parameters
SI 65536
SF 125.7804076 MHz
WDW no
SSB 0
LB 0.00 Hz
GB 0
PC 2.00

1D NMR plot parameters
CX 22.80 cm
CY 15.65 cm
F1P 230.637 ppm
F1 29009.68 Hz
F2P -10.287 ppm
F2 -1293.96 Hz
PPMCM 10.56688 ppm/cm
HZCM 1329.10693 Hz/cm

1H spectrum

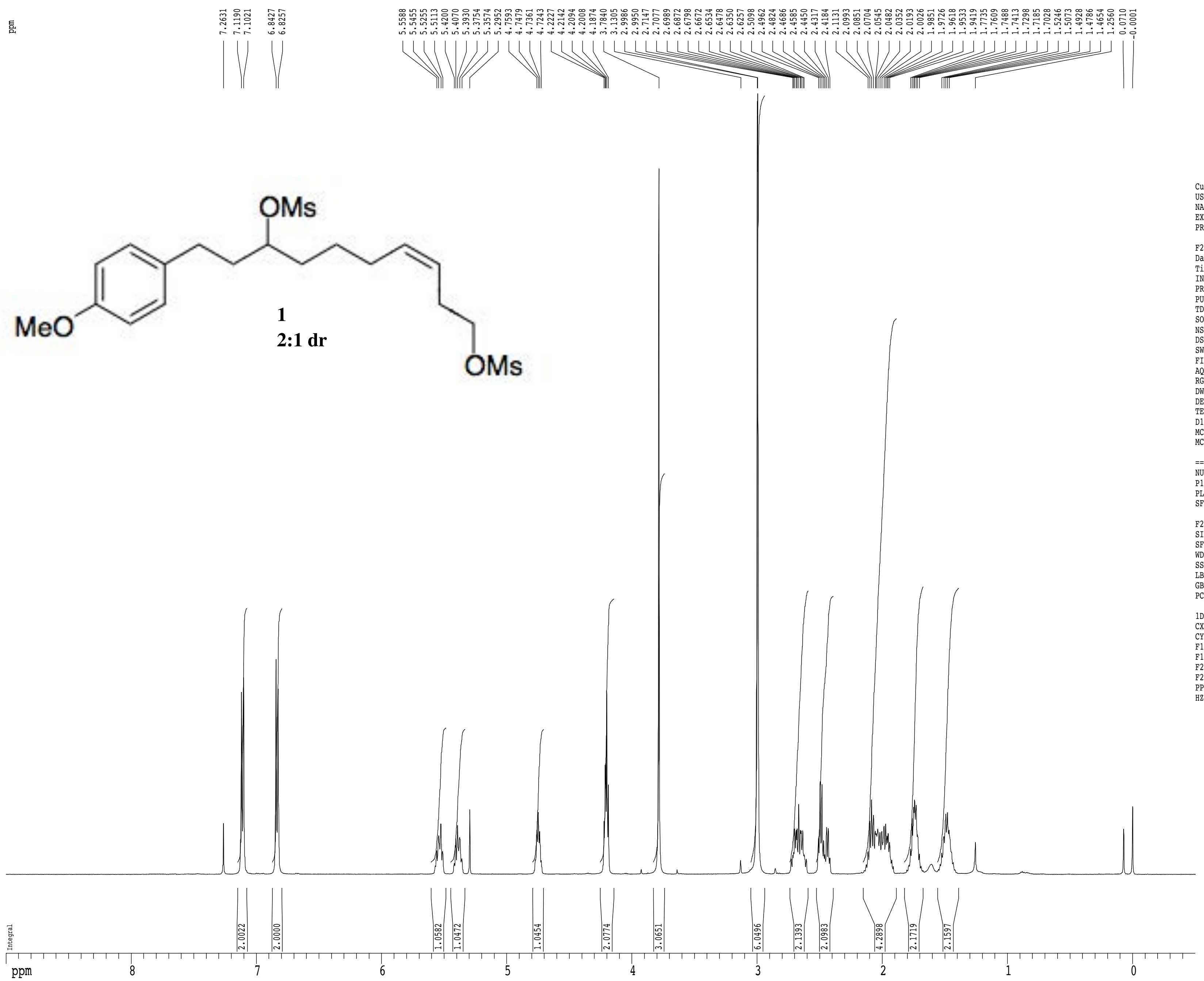


```
Current Data Parameters
USER            khewitt1
NAME        KAH-V-065-Z
EXPNO                 2
PROCNO                1

F2 - Acquisition Parameters
Date_          20210902
Time              16.32
INSTRUM         cryo500
PROBHD   5 mm CPTCI 1H-
PULPROG            zg30
TD                48074
SOLVENT           CDCl3
NS                    8
DS                    2
SWH            8012.820 Hz
FIDRES         0.166677 Hz
AQ            2.9998677 sec
RG                  5.7
DW               62.400 usec
DE                 6.00 usec
TE                298.0 K
D1           0.10000000 sec
MCREST       0.00000000 sec
MCWRK        0.01500000 sec

======== CHANNEL f1 ========
NUC1                 1H
P1                 9.75 usec
PL1                1.60 dB
SFO1        500.2235015 MHz

F2 - Processing parameters
SI                65536
SF          500.2200298 MHz
WDW                  no
SSB                   0
LB                 0.00 Hz
GB                    0
PC                 1.00

1D NMR plot parameters
CX                22.80 cm
CY                15.00 cm
F1P               9.000 ppm
F1              4501.98 Hz
F2P              -0.500 ppm
F2              -250.11 Hz
PPMCM           0.41667 ppm/cm
HZCM          208.42502 Hz/cm
```

Z-restored spin-echo 13C spectrum with 1H decoupling

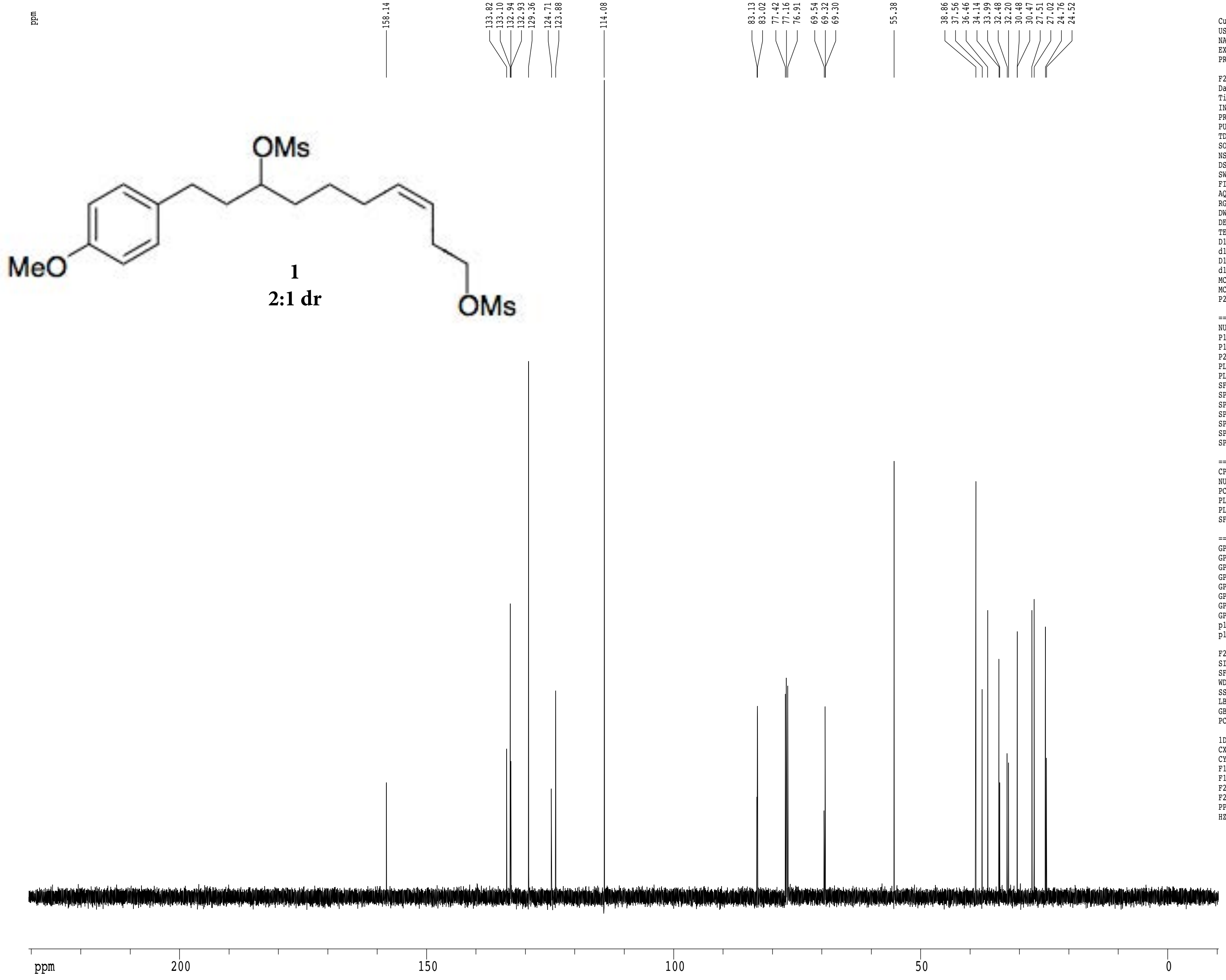

1
2:1 dr

Current Data Parameters

USER	khewitt1
NAME	KAH-V-065-Z
EXPNO	4
PROCNO	1

F2 - Acquisition Parameters

Date_	20210902
Time	16.46
INSTRUM	cryo500
PROBHD	5 mm CPTCI 1H-
PULPROG	SpinEchopg30gp2.prd
TD	65536
SOLVENT	CDCl3
NS	104
DS	16
SWH	30303.031 Hz
FIDRES	0.462388 Hz
AQ	1.0813940 sec
RG	2896.3
DW	16.500 usec
DE	6.00 usec
TE	298.0 K
D1	0.25000000 sec
d11	0.03000000 sec
D16	0.00020000 sec
d17	0.00019600 sec
MCREST	0.00000000 sec
MCWRK	0.01500000 sec
P2	37.70 usec

======== CHANNEL f1 ========

NUC1	13C
P1	18.85 usec
P12	2000.00 usec
P20	500.00 usec
PL0	120.00 dB
PL1	-1.00 dB
SFO1	125.7942548 MHz
SP2	1.55 dB
SP4	1.55 dB
SPNAM2	Crp60comp.4
SPNAM4	Crp60,0.5,20.1
SPOFF2	0.00 Hz
SPOFF4	0.00 Hz

======== CHANNEL f2 ========

CPDPRG2	waltz16
NUC2	1H
PCPD2	100.00 usec
PL2	1.60 dB
PL12	22.00 dB
SFO2	500.2225011 MHz

====== GRADIENT CHANNEL =====

GPNAM1	SINE.100
GPNAM2	SINE.100
GPX1	0.00 %
GPX2	0.00 %
GPY1	0.00 %
GPY2	0.00 %
GPZ1	30.00 %
GPZ2	50.00 %
p15	500.00 usec
p16	1000.00 usec

F2 - Processing parameters

SI	65536
SF	125.7804113 MHz
WDW	no
SSB	0
LB	0.00 Hz
GB	0
PC	2.00

1D NMR plot parameters

CX	22.80 cm
CY	15.65 cm
F1P	230.637 ppm
F1	29009.68 Hz
F2P	-10.287 ppm
F2	-1293.96 Hz
PPMCM	10.56688 ppm/cm
HZCM	1329.10693 Hz/cm

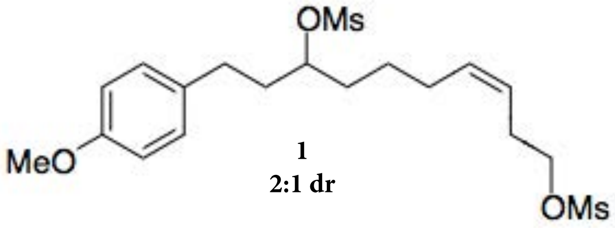

1
2:1 dr

gcosy60

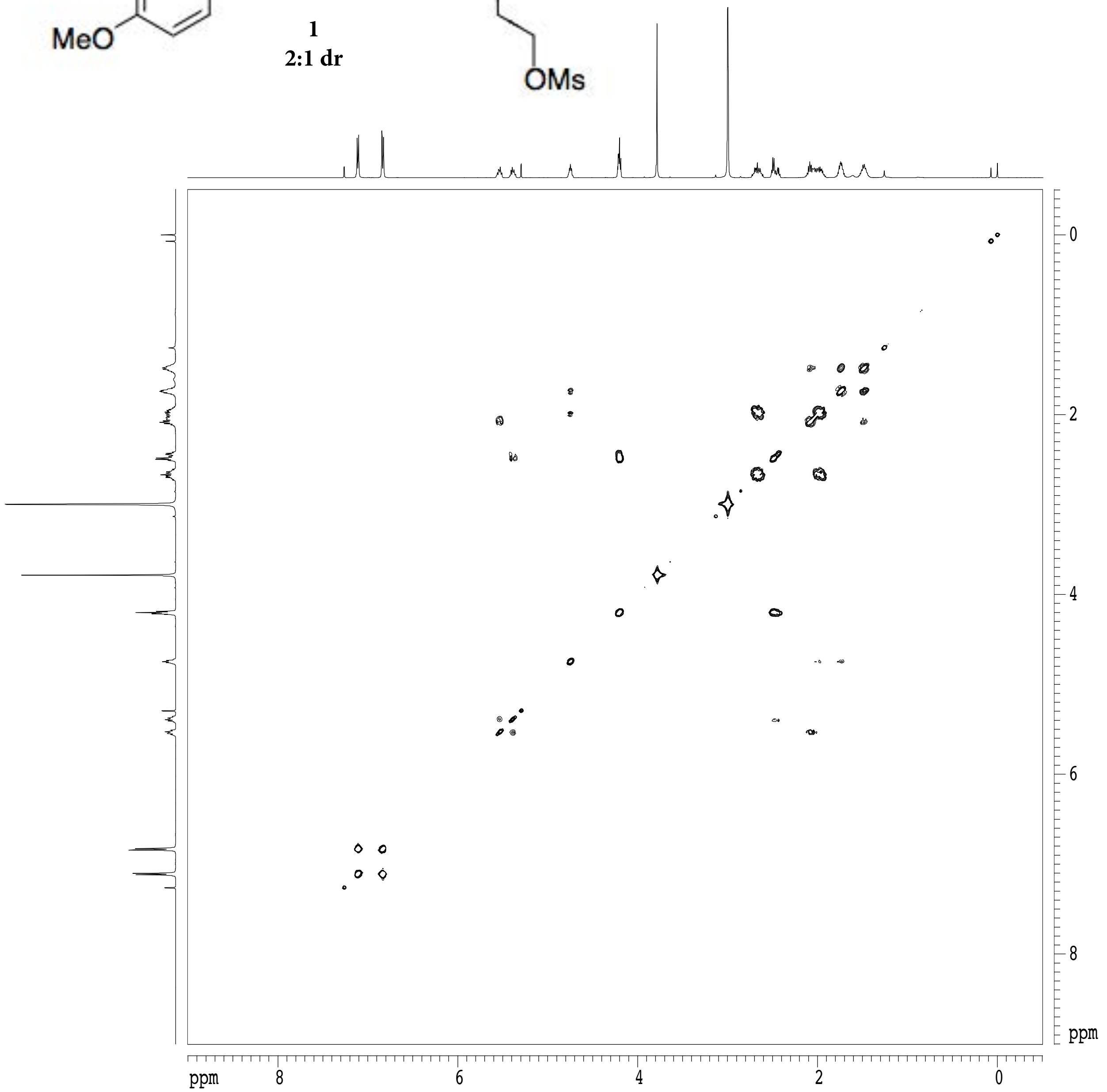

Current Data Parameters
USER khewitt1
NAME KAH-V-065-Z
EXPNO 3
PROCNO 1

F2 - Acquisition Parameters
Date_ 20210902
Time 16.34
INSTRUM cryo500
PROBHD 5 mm CPTCI 1H-
PULPROG cosygp60.prd
TD 2048
SOLVENT CDCl3
NS 1
DS 16
SWH 8012.820 Hz
FIDRES 3.912510 Hz
AQ 0.1278452 sec
RG 35.9
DW 62.400 usec
DE 6.00 usec
TE 298.0 K
d0 0.00000300 sec
D1 1.00000000 sec
d13 0.00000300 sec
D16 0.00020000 sec
IN0 0.00012480 sec

======== CHANNEL f1 ========
NUC1 1H
P1 9.75 usec
PL1 1.60 dB
SFO1 500.2235015 MHz

====== GRADIENT CHANNEL =====
GPNAM1 SMSQ10.100
GPNAM2 SMSQ10.100
GPX1 0.00 %
GPX2 0.00 %
GPY1 0.00 %
GPY2 0.00 %
GPZ1 17.00 %
GPZ2 17.00 %
P16 1000.00 usec

F1 - Acquisition parameters
ND0 1
TD 512
SFO1 500.2235 MHz
FIDRES 15.650040 Hz
SW 16.018 ppm
FnMODE QF

F2 - Processing parameters
SI 1024
SF 500.2200298 MHz
WDW SINE
SSB 0
LB 0.00 Hz
GB 0
PC 1.00

F1 - Processing parameters
SI 1024
MC2 QF
SF 500.2200298 MHz
WDW SINE
SSB 0
LB 0.00 Hz
GB 0

2D NMR plot parameters
CX2 15.00 cm
CX1 15.00 cm
F2PLO 9.005 ppm
F2LO 4504.64 Hz
F2PHI -0.506 ppm
F2HI -252.97 Hz
F1PLO 9.005 ppm
F1LO 4504.64 Hz
F1PHI -0.506 ppm
F1HI -252.97 Hz
F2PPMCM 0.63407 ppm/cm
F2HZCM 317.17413 Hz/cm
F1PPMCM 0.63407 ppm/cm
F1HZCM 317.17413 Hz/cm

1H spectrum

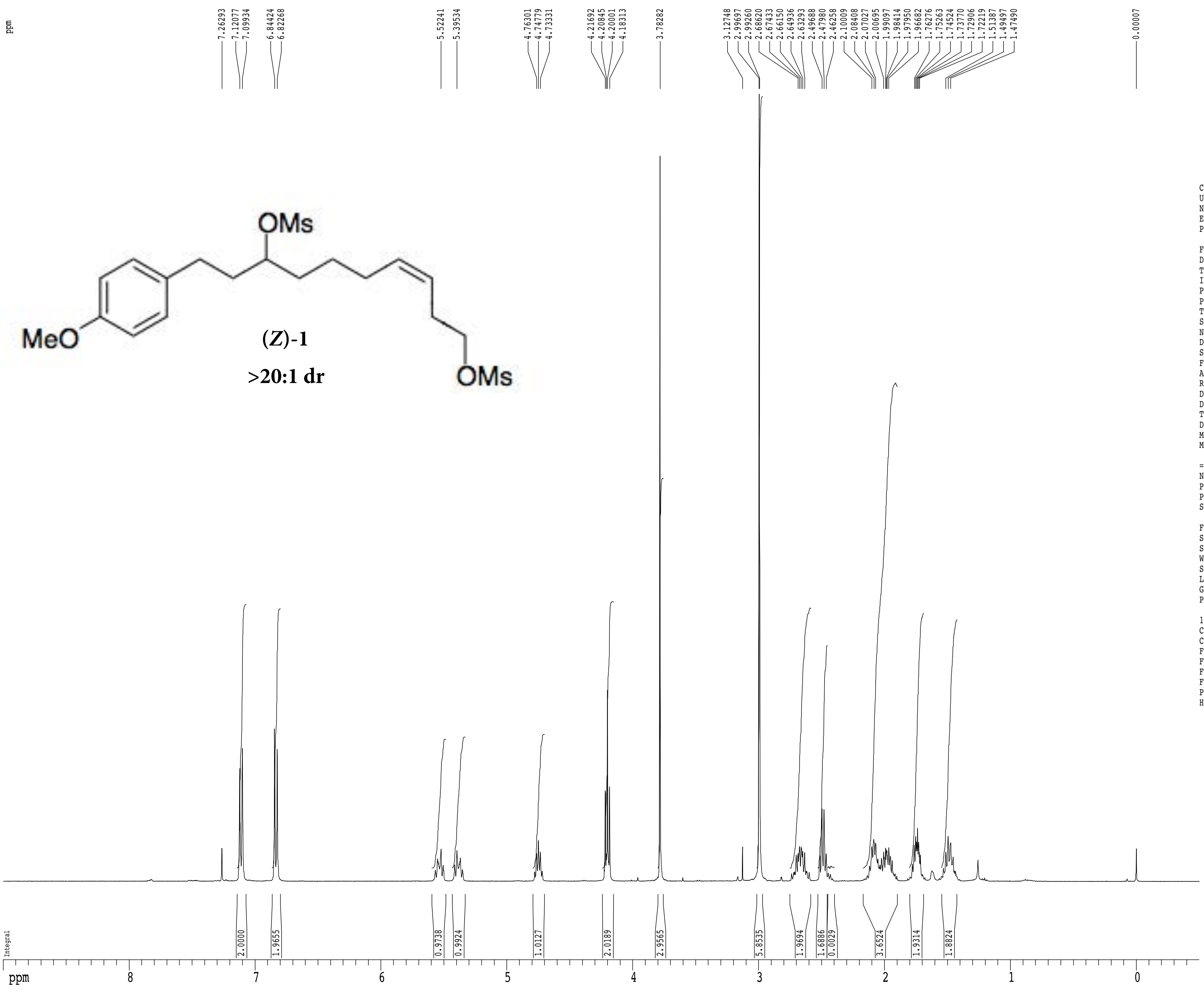

Current Data Parameters
USER khewitt1
NAME KAH-V-166-Z
EXPNO 1
PROCNO 1

F2 - Acquisition Parameters
Date_ 20211108
Time 9.18
INSTRUM drx400
PROBHD 5 mm QNP H/F/P
PULPROG zg30
TD 38460
SOLVENT CDCl3T
NS 8
DS 2
SWH 6410.256 Hz
FIDRES 0.166673 Hz
AQ 2.9999299 sec
RG 114
DW 78.000 usec
DE 4.50 usec
TE 298.1 K
D1 0.10000000 sec
MCREST 0.00000000 sec
MCWRK 0.01500000 sec

======== CHANNEL f1 ========
NUC1 1H
P1 12.00 usec
PL1 -0.90 dB
SFO1 400.1328009 MHz

F2 - Processing parameters
SI 65536
SF 400.1300202 MHz
WDW no
SSB 0
LB 0.00 Hz
GB 0
PC 2.00

1D NMR plot parameters
CX 22.80 cm
CY 15.00 cm
F1P 9.000 ppm
F1 3601.17 Hz
F2P -0.500 ppm
F2 -200.06 Hz
PPMCM 0.41667 ppm/cm
HZCM 166.72086 Hz/cm

13C spectrum with 1H decoupling

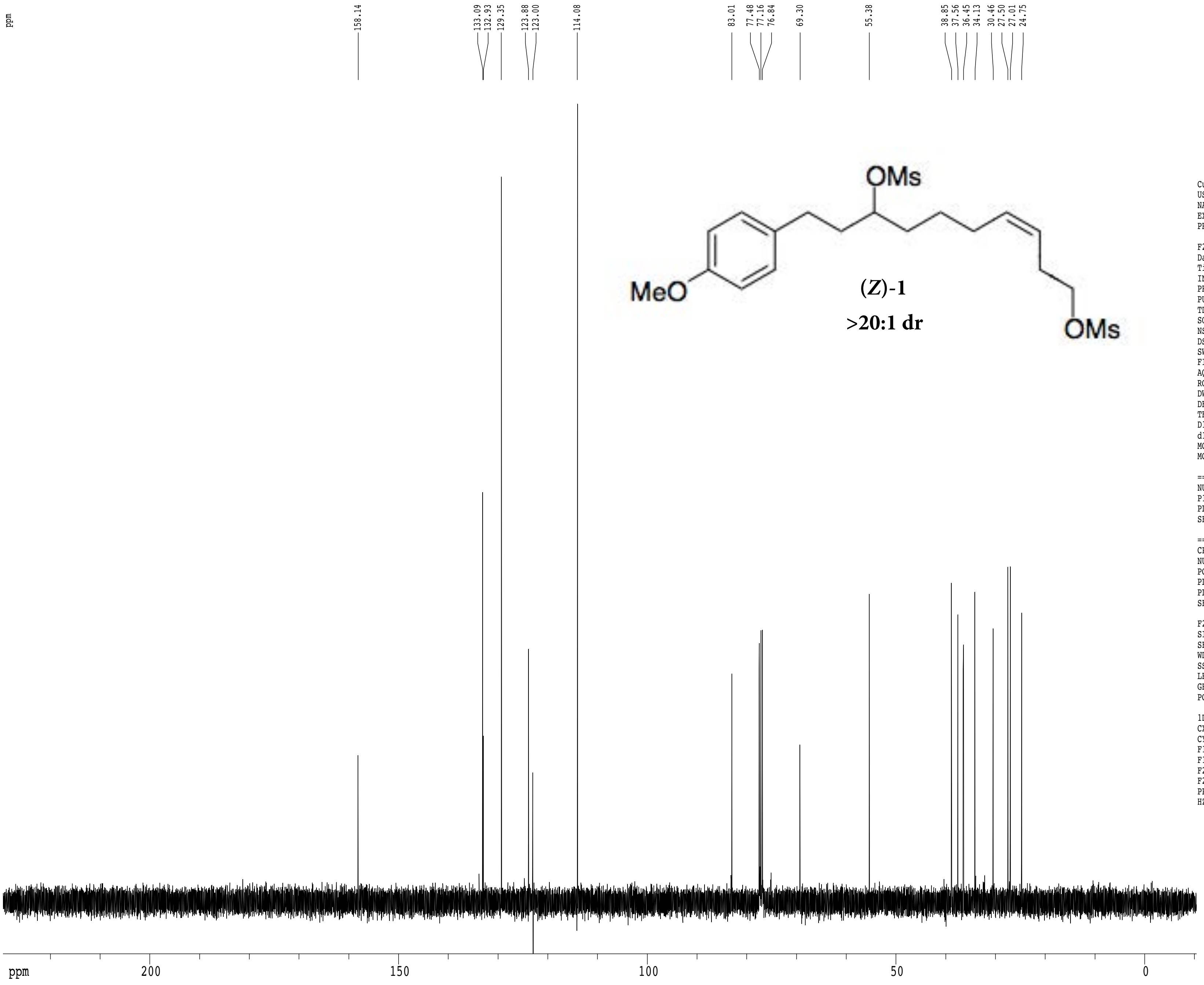

Current Data Parameters
USER khewitt1
NAME KAH-V-166-Z
EXPNO 2
PROCNO 1

F2 - Acquisition Parameters
Date_ 20211108
Time 9.25
INSTRUM drx400
PROBHD 5 mm QNP H/F/P
PULPROG zgdc30
TD 65536
SOLVENT CDCl3
NS 280
DS 4
SWH 24154.590 Hz
FIDRES 0.368570 Hz
AQ 1.3566452 sec
RG 8192
DW 20.700 usec
DE 20.39 usec
TE 298.1 K
D1 0.10000000 sec
d11 0.03000000 sec
MCREST 0.00000000 sec
MCWRK 0.01500000 sec

======== CHANNEL f1 ========
NUC1 13C
P1 7.90 usec
PL1 -3.00 dB
SFO1 100.6237964 MHz

======== CHANNEL f2 ========
CPDPRG2 waltz16
NUC2 1H
PCPD2 90.00 usec
PL2 -0.90 dB
PL12 17.00 dB
SFO2 400.1328009 MHz

F2 - Processing parameters
SI 65536
SF 100.6127621 MHz
WDW no
SSB 0
LB 0.00 Hz
GB 0
PC 1.00

1D NMR plot parameters
CX 22.80 cm
CY 15.50 cm
F1P 229.496 ppm
F1 23090.21 Hz
F2P -10.579 ppm
F2 -1064.37 Hz
PPMCM 10.52959 ppm/cm
HZCM 1059.41150 Hz/cm

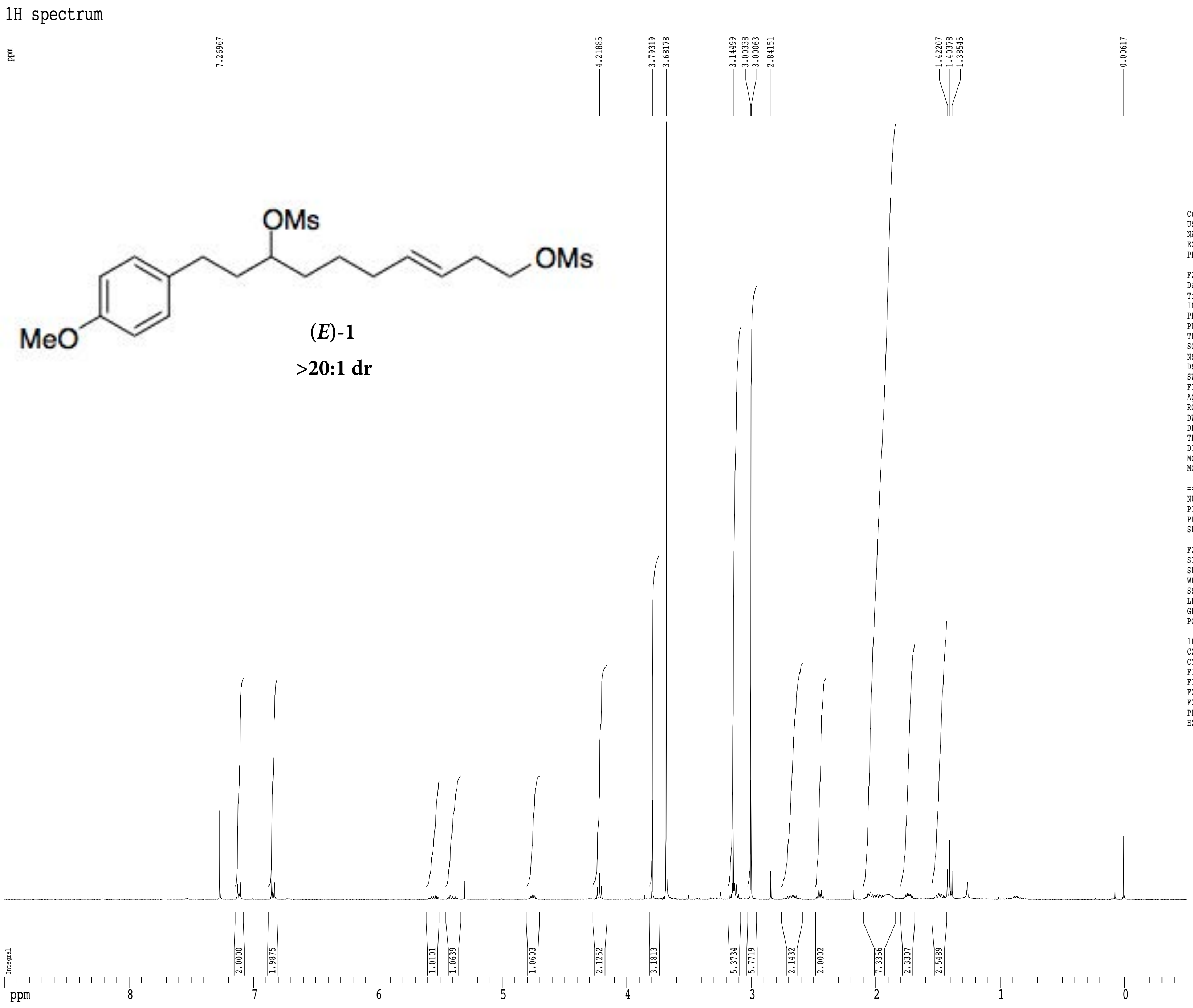


```
Current Data Parameters
USER            khewitt1
NAME         KAH-V-176-Z
EXPNO                  1
PROCNO                 1

F2 - Acquisition Parameters
Date_           20211113
Time               15.40
INSTRUM           drx400
PROBHD   5 mm QNP H/F/P
PULPROG             zg30
TD                 38460
SOLVENT            CDCl3T
NS                     8
DS                     2
SWH             6410.256 Hz
FIDRES          0.166673 Hz
AQ             2.9999299 sec
RG                   362
DW                78.000 usec
DE                  4.50 usec
TE                 298.0 K
D1            0.10000000 sec
MCREST        0.00000000 sec
MCWRK         0.01500000 sec

======== CHANNEL f1 ========
NUC1                  1H
P1                 12.00 usec
PL1                -0.90 dB
SFO1        400.1328009 MHz

F2 - Processing parameters
SI                 65536
SF          400.1300175 MHz
WDW                   no
SSB                    0
LB                  0.00 Hz
GB                     0
PC                  2.00

1D NMR plot parameters
CX                 22.80 cm
CY                 15.00 cm
F1P                9.000 ppm
F1               3601.17 Hz
F2P               -0.500 ppm
F2               -200.06 Hz
PPMCM            0.41667 ppm/cm
HZCM           166.72084 Hz/cm
```

Z-restored spin-echo 13C spectrum with 1H decoupling

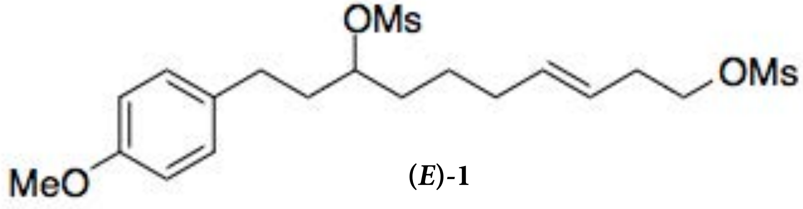

(*E*)-1

>20:1 dr

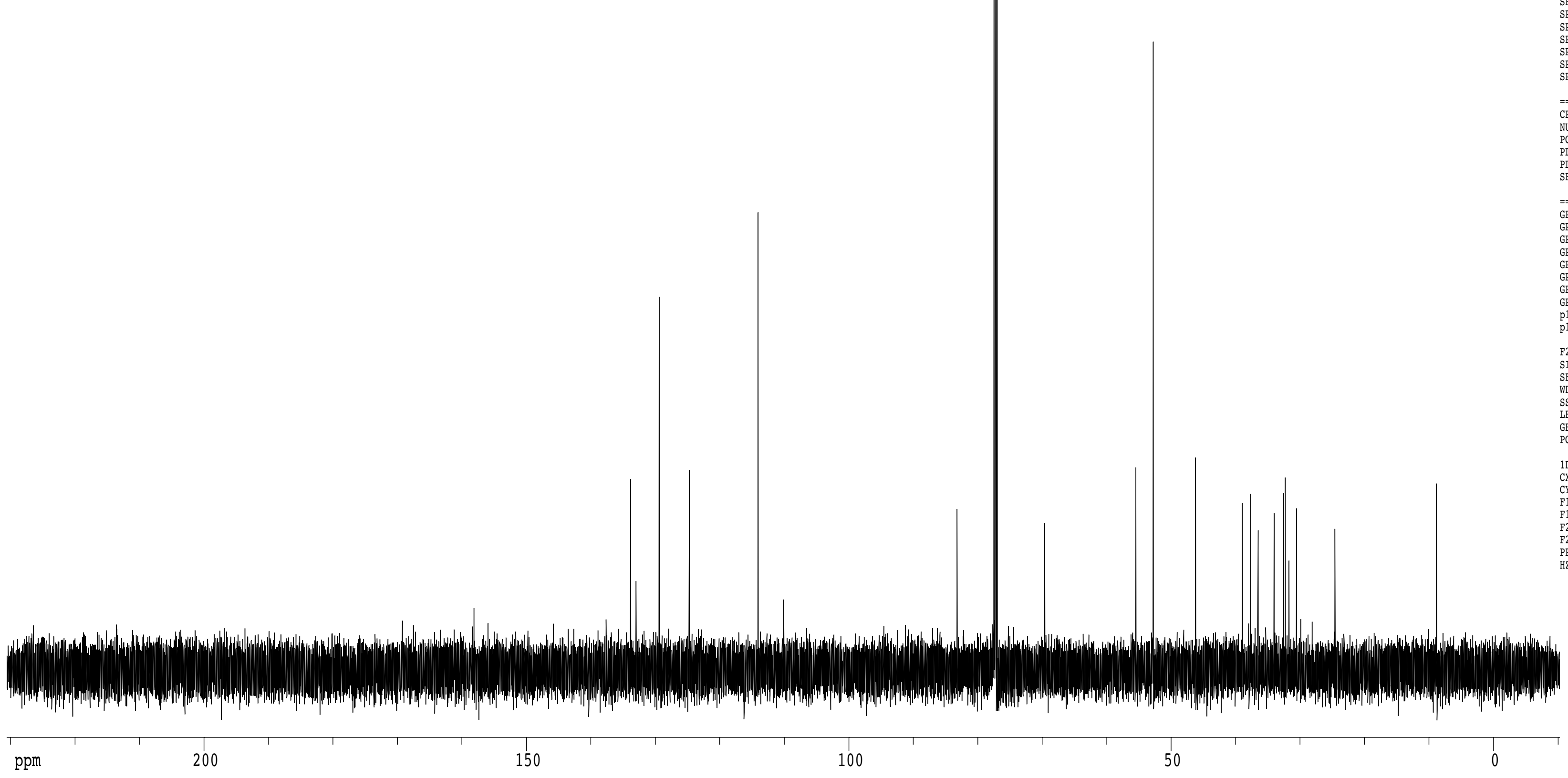

Current Data Parameters
USER khewitt1
NAME KAH-V-176-Z
EXPNO 3
PROCNO 1

F2 - Acquisition Parameters
Date_ 20211113
Time 15.59
INSTRUM cryo500
PROBHD 5 mm CPTCI 1H-
PULPROG SpinEchopg30gp2.prd
TD 65536
SOLVENT CDCl3T
NS 120
DS 16
SWH 30303.031 Hz
FIDRES 0.462388 Hz
AQ 1.0813940 sec
RG 2298.8
DW 16.500 usec
DE 6.00 usec
TE 298.0 K
D1 0.25000000 sec
d11 0.03000000 sec
D16 0.00020000 sec
d17 0.00019600 sec
MCREST 0.00000000 sec
MCWRK 0.01500000 sec
P2 37.70 usec

======== CHANNEL f1 ========
NUC1 13C
P1 18.85 usec
P12 2000.00 usec
P20 500.00 usec
PL0 120.00 dB
PL1 -1.00 dB
SFO1 125.7942548 MHz
SP2 1.55 dB
SP4 1.55 dB
SPNAM2 Crp60comp.4
SPNAM4 Crp60,0.5,20.1
SPOFF2 0.00 Hz
SPOFF4 0.00 Hz

======== CHANNEL f2 ========
CPDPRG2 waltz16
NUC2 1H
PCPD2 100.00 usec
PL2 1.60 dB
PL12 22.00 dB
SFO2 500.2225011 MHz

====== GRADIENT CHANNEL =====
GPNAM1 SINE.100
GPNAM2 SINE.100
GPX1 0.00 %
GPX2 0.00 %
GPY1 0.00 %
GPY2 0.00 %
GPZ1 30.00 %
GPZ2 50.00 %
p15 500.00 usec
p16 1000.00 usec

F2 - Processing parameters
SI 65536
SF 125.7804090 MHz
WDW no
SSB 0
LB 0.00 Hz
GB 0
PC 2.00

1D NMR plot parameters
CX 22.80 cm
CY 15.65 cm
F1P 230.637 ppm
F1 29009.68 Hz
F2P -10.287 ppm
F2 -1293.96 Hz
PPMCM 10.56688 ppm/cm
HZCM 1329.10693 Hz/cm

1H spectrum

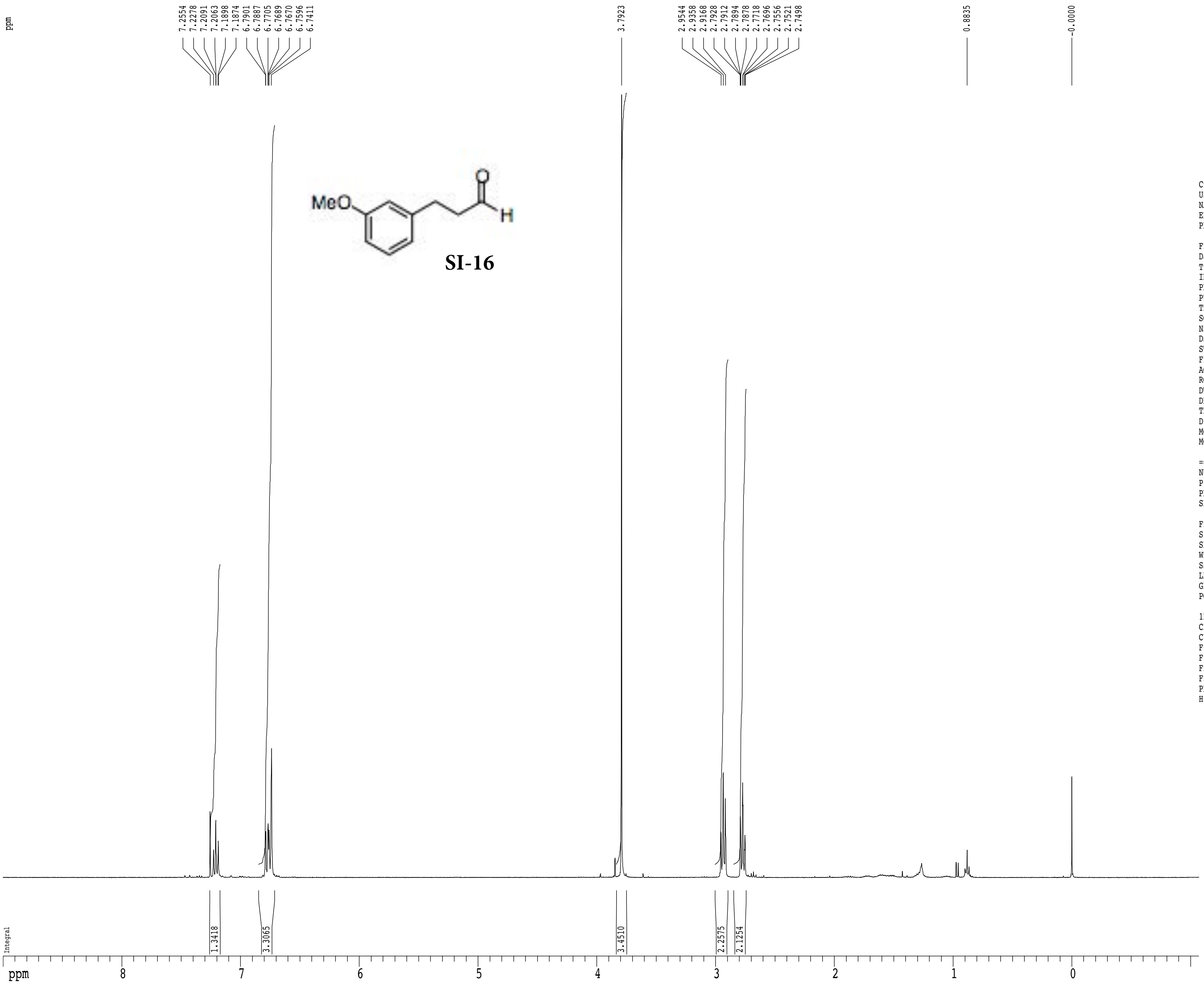


```
Current Data Parameters
USER              caherber
NAME              CAH-I-263
EXPNO                     1
PROCNO                    1

F2 - Acquisition Parameters
Date_              20210819
Time                   9.20
INSTRUM              drx400
PROBHD    5 mm QNP H/F/P
PULPROG                zg30
TD                    65536
SOLVENT               CDCl3
NS                        8
DS                        2
SWH              6410.256 Hz
FIDRES           0.097813 Hz
AQ              5.1118579 sec
RG                    322.5
DW                   78.000 usec
DE                     4.50 usec
TE                    298.0 K
D1               0.10000000 sec
MCREST           0.00000000 sec
MCWRK            0.01500000 sec

======== CHANNEL f1 ========
NUC1                     1H
P1                    12.00 usec
PL1                   -1.60 dB
SFO1           400.1328009 MHz

F2 - Processing parameters
SI                    65536
SF             400.1300232 MHz
WDW                      no
SSB                       0
LB                     0.00 Hz
GB                        0
PC                     2.00

1D NMR plot parameters
CX                    22.80 cm
CY                    15.00 cm
F1P                   9.000 ppm
F1                  3601.17 Hz
F2P                  -1.068 ppm
F2                  -427.44 Hz
PPMCM               0.44159 ppm/cm
HZCM              176.69353 Hz/cm
```

1H spectrum

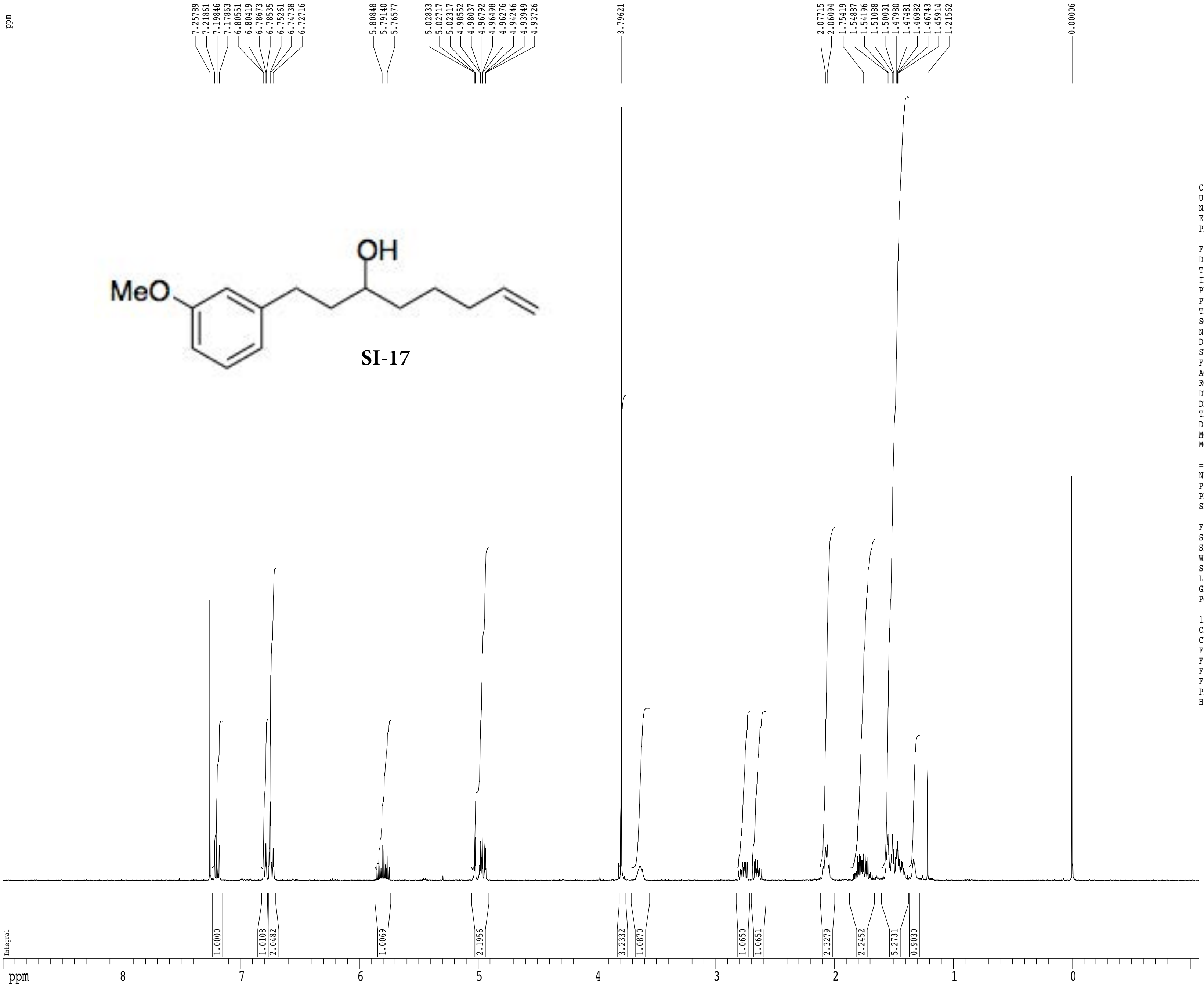


```
Current Data Parameters
USER            caherber
NAME           CAH-I-267
EXPNO                  1
PROCNO                 1

F2 - Acquisition Parameters
Date_           20210825
Time               10.12
INSTRUM           drx400
PROBHD   5 mm Multinucl
PULPROG             zg30
TD                 65536
SOLVENT            CDCl3
NS                     8
DS                     2
SWH             6410.256 Hz
FIDRES          0.097813 Hz
AQ             5.1118579 sec
RG                 645.1
DW                78.000 usec
DE                  4.50 usec
TE                 297.9 K
D1            0.10000000 sec
MCREST        0.00000000 sec
MCWRK         0.01500000 sec

======== CHANNEL f1 ========
NUC1                  1H
P1                 12.00 usec
PL1                -1.10 dB
SFO1       400.1328009 MHz

F2 - Processing parameters
SI                 65536
SF           400.1300224 MHz
WDW                   no
SSB                    0
LB                  0.00 Hz
GB                     0
PC                  2.00

1D NMR plot parameters
CX                 22.80 cm
CY                 15.00 cm
F1P                9.000 ppm
F1               3601.17 Hz
F2P               -1.066 ppm
F2               -426.66 Hz
PPMCM            0.44150 ppm/cm
HZCM           176.65921 Hz/cm
```

1H spectrum

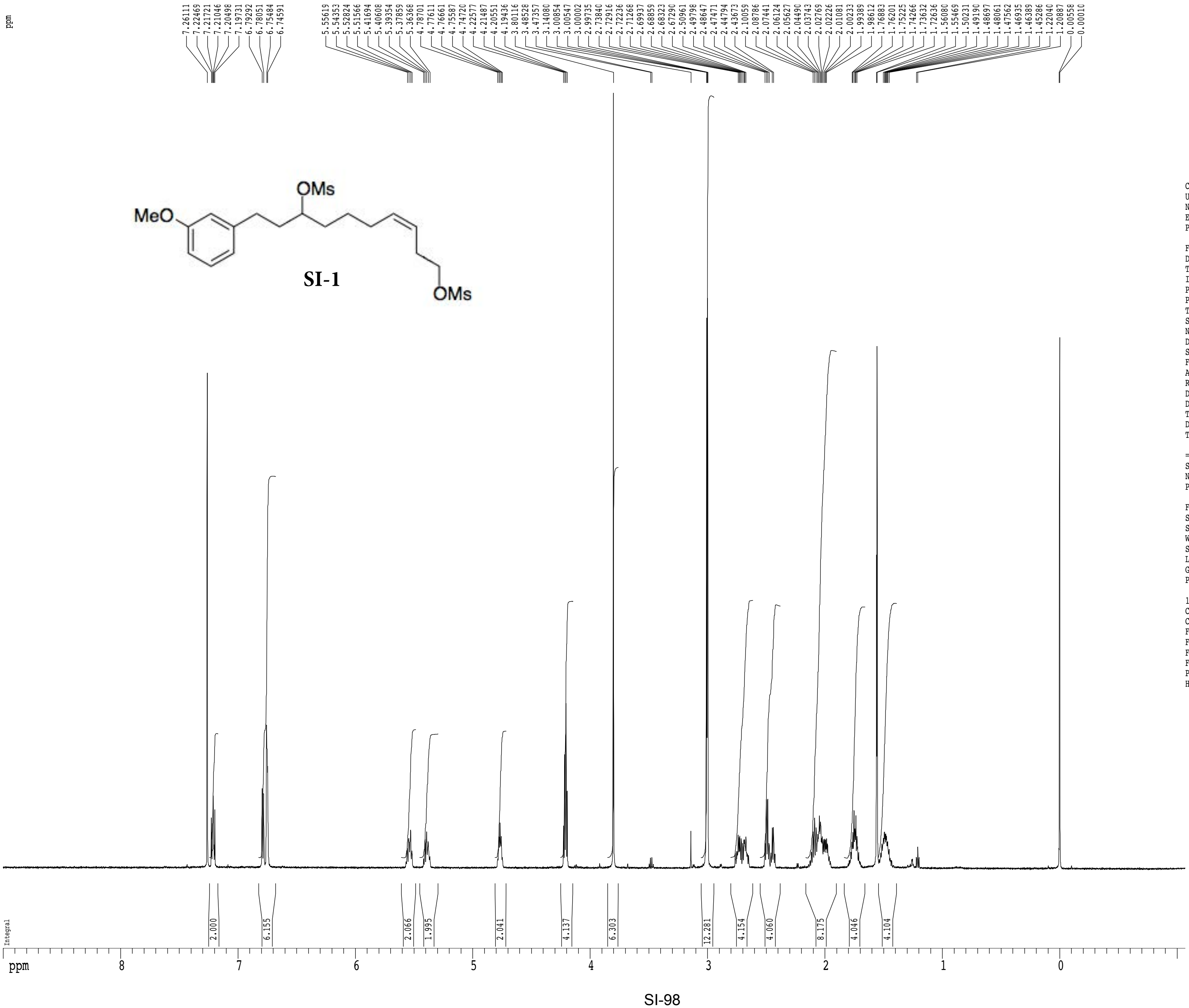


```
Current Data Parameters
USER          caherber
NAME     CAH-I-285-full
EXPNO                1
PROCNO               1

F2 - Acquisition Parameters
Date_          20210922
Time               9.48
INSTRUM           av600
PROBHD   5 mm CPBBO BB-
PULPROG            zg30
TD                98074
SOLVENT           CDCl3
NS                    8
DS                    2
SWH            9615.385 Hz
FIDRES         0.098042 Hz
AQ            5.0998979 sec
RG                 20.2
DW               52.000 usec
DE                14.23 usec
TE                298.0 K
D1           0.10000000 sec
TD0                   1

======== CHANNEL f1 ========
SFO1        600.1342009 MHz
NUC1                 1H
P1                 9.50 usec

F2 - Processing parameters
SI                65536
SF          600.1300345 MHz
WDW                  no
SSB                   0
LB                 0.00 Hz
GB                    0
PC                 1.00

1D NMR plot parameters
CX                22.80 cm
CY                15.00 cm
F1P               9.000 ppm
F1              5401.17 Hz
F2P              -1.069 ppm
F2              -641.31 Hz
PPMCM           0.44161 ppm/cm
HZCM          265.02106 Hz/cm
```

13C Spectrum

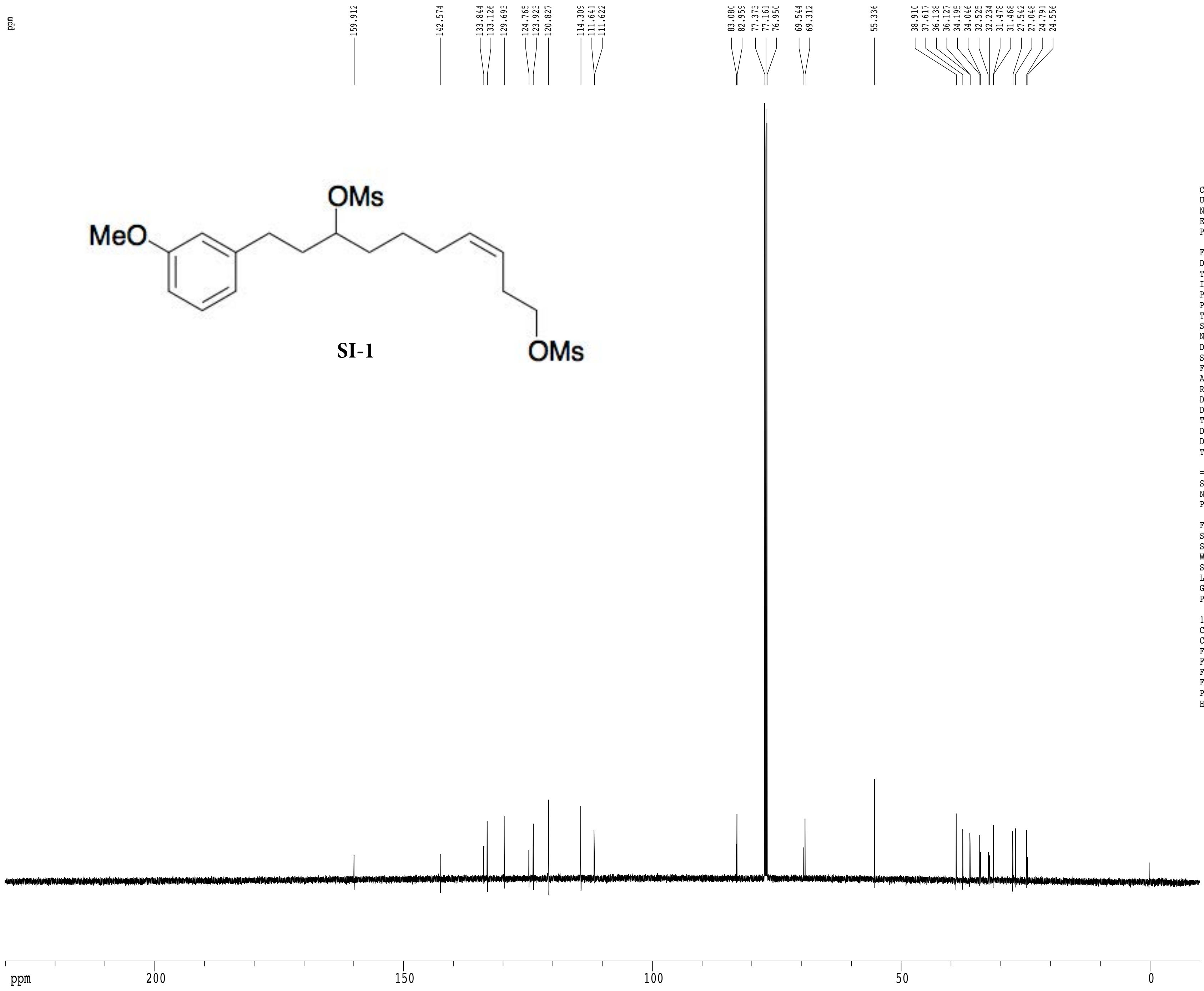

SI-1

Current Data Parameters
USER caherber
NAME CAH-I-285-full
EXPNO 2
PROCNO 1

F2 - Acquisition Parameters
Date_ 20210922
Time 9.59
INSTRUM av600
PROBHD 5 mm CPBBO BB-
PULPROG zgdc30
TD 65536
SOLVENT CDCl3
NS 246
DS 4
SWH 36231.883 Hz
FIDRES 0.552855 Hz
AQ 0.9044468 sec
RG 2050
DW 13.800 usec
DE 19.63 usec
TE 298.0 K
D1 0.40000001 sec
D11 0.03000000 sec
TD0 1

======== CHANNEL f1 ========
SFO1 150.9194080 MHz
NUC1 13C
P1 10.10 usec

F2 - Processing parameters
SI 65536
SF 150.9027936 MHz
WDW no
SSB 0
LB 0.00 Hz
GB 0
PC 1.00

1D NMR plot parameters
CX 22.80 cm
CY 15.00 cm
F1P 230.117 ppm
F1 34725.34 Hz
F2P -9.984 ppm
F2 -1506.54 Hz
PPMCM 10.53074 ppm/cm
HZCM 1589.11768 Hz/cm

1H spectrum

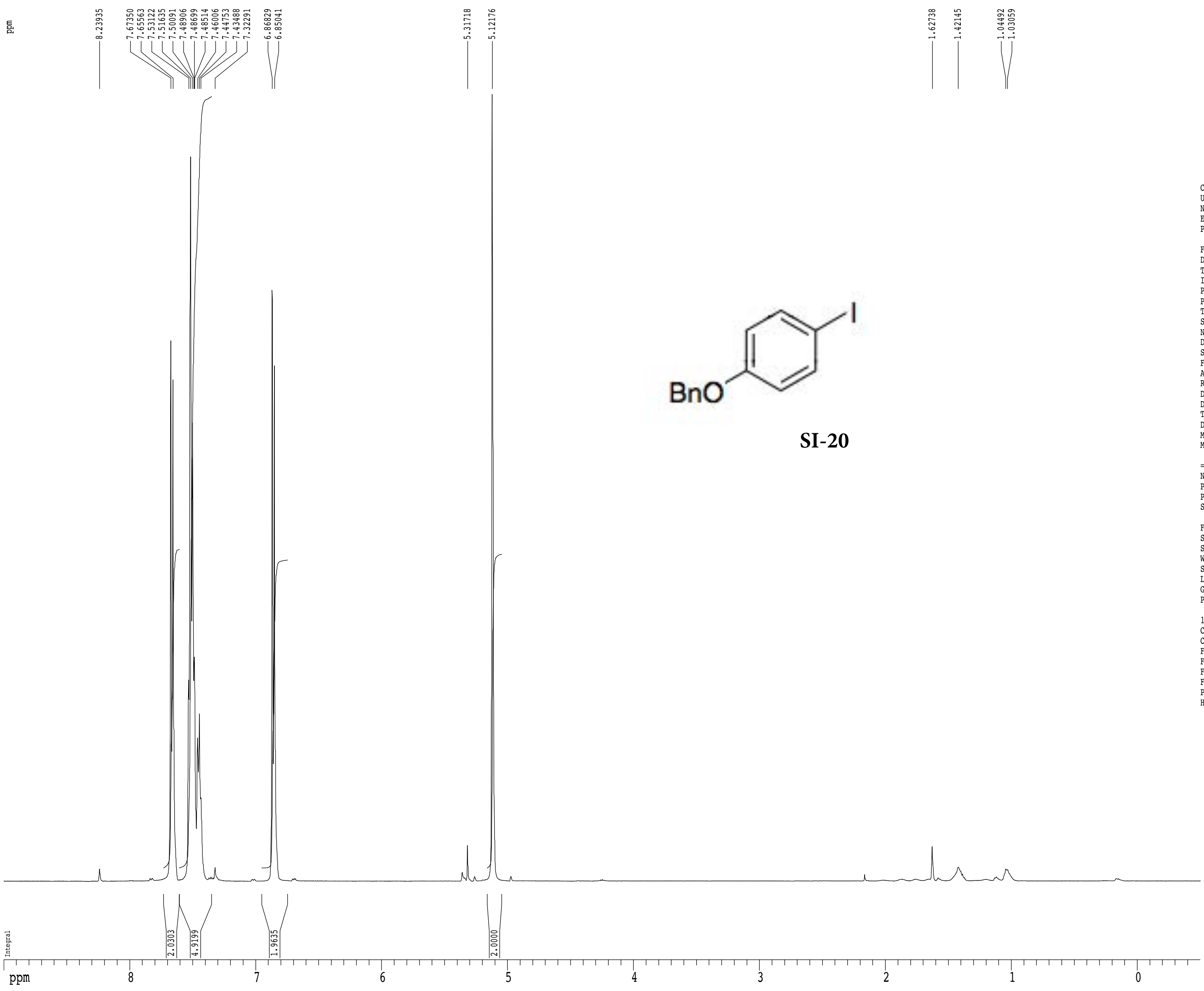

SI-20

```
Current Data Parameters
USER          khewitt1
NAME       KAH-IV-276-1
EXPNO                1
PROCNO               1

F2 - Acquisition Parameters
Date_         20210605
Time             14.11
INSTRUM        cryo500
PROBHD   5 mm CPTCI 1H-
PULPROG           zg30
TD               48074
SOLVENT         CDCl3T
NS                   8
DS                   2
SWH           8012.820 Hz
FIDRES        0.166677 Hz
AQ           2.9998677 sec
RG                 3.6
DW              62.400 usec
DE                6.00 usec
TE               298.0 K
D1          0.10000000 sec
MCREST      0.00000000 sec
MCWRK       0.01500000 sec

======== CHANNEL f1 ========
NUC1                1H
P1                9.75 usec
PL1               1.60 dB
SFO1       500.2235015 MHz

F2 - Processing parameters
SI               65536
SF         500.2200000 MHz
WDW                 no
SSB                  0
LB                0.00 Hz
GB                   0
PC                1.00

1D NMR plot parameters
CX               22.80 cm
CY               15.00 cm
F1P              9.000 ppm
F1             4501.98 Hz
F2P             -0.500 ppm
F2             -250.11 Hz
PPMCM          0.41667 ppm/cm
HZCM         208.42500 Hz/cm
```

1H spectrum

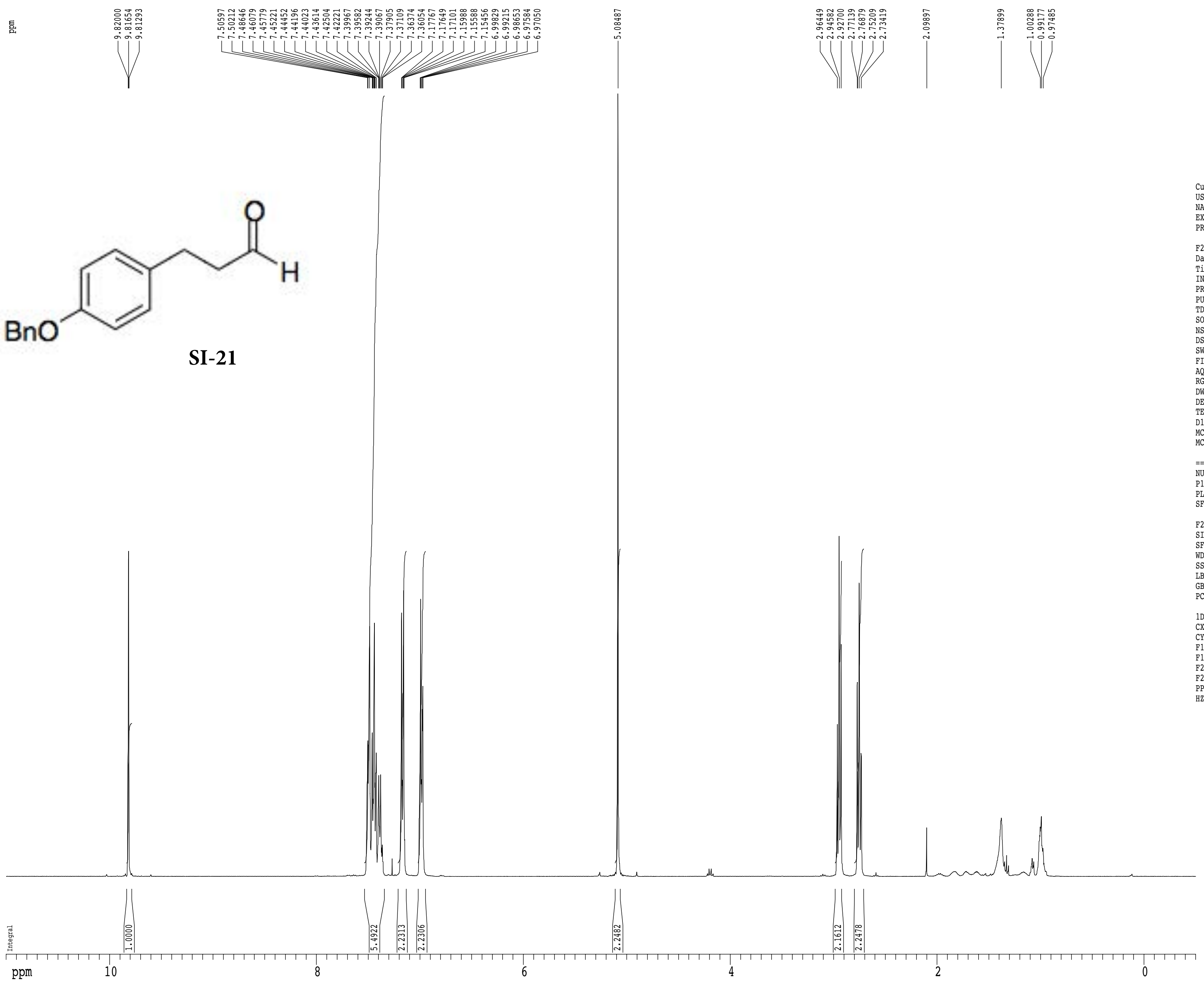

SI-21

```
Current Data Parameters
USER          khewitt1
NAME        KAH-V-265-1
EXPNO                1
PROCNO               1

F2 - Acquisition Parameters
Date_          20220205
Time              16.33
INSTRUM          drx400
PROBHD   5 mm QNP H/F/P
PULPROG            zg30
TD                65536
SOLVENT          CDCl3T
NS                    8
DS                    2
SWH            6410.256 Hz
FIDRES         0.097813 Hz
AQ            5.1118579 sec
RG                   32
DW               78.000 usec
DE                 4.50 usec
TE                298.0 K
D1           0.10000000 sec
MCREST       0.00000000 sec
MCWRK        0.01500000 sec

======== CHANNEL f1 ========
NUC1                 1H
P1                12.00 usec
PL1               -0.90 dB
SFO1        400.1328009 MHz

F2 - Processing parameters
SI                65536
SF          400.1300175 MHz
WDW                  no
SSB                   0
LB                 0.00 Hz
GB                    0
PC                 2.00

1D NMR plot parameters
CX                22.80 cm
CY                15.00 cm
F1P              11.000 ppm
F1              4401.43 Hz
F2P              -0.500 ppm
F2              -200.07 Hz
PPMCM           0.50439 ppm/cm
HZCM          201.81996 Hz/cm
```

13C spectrum with 1H decoupling

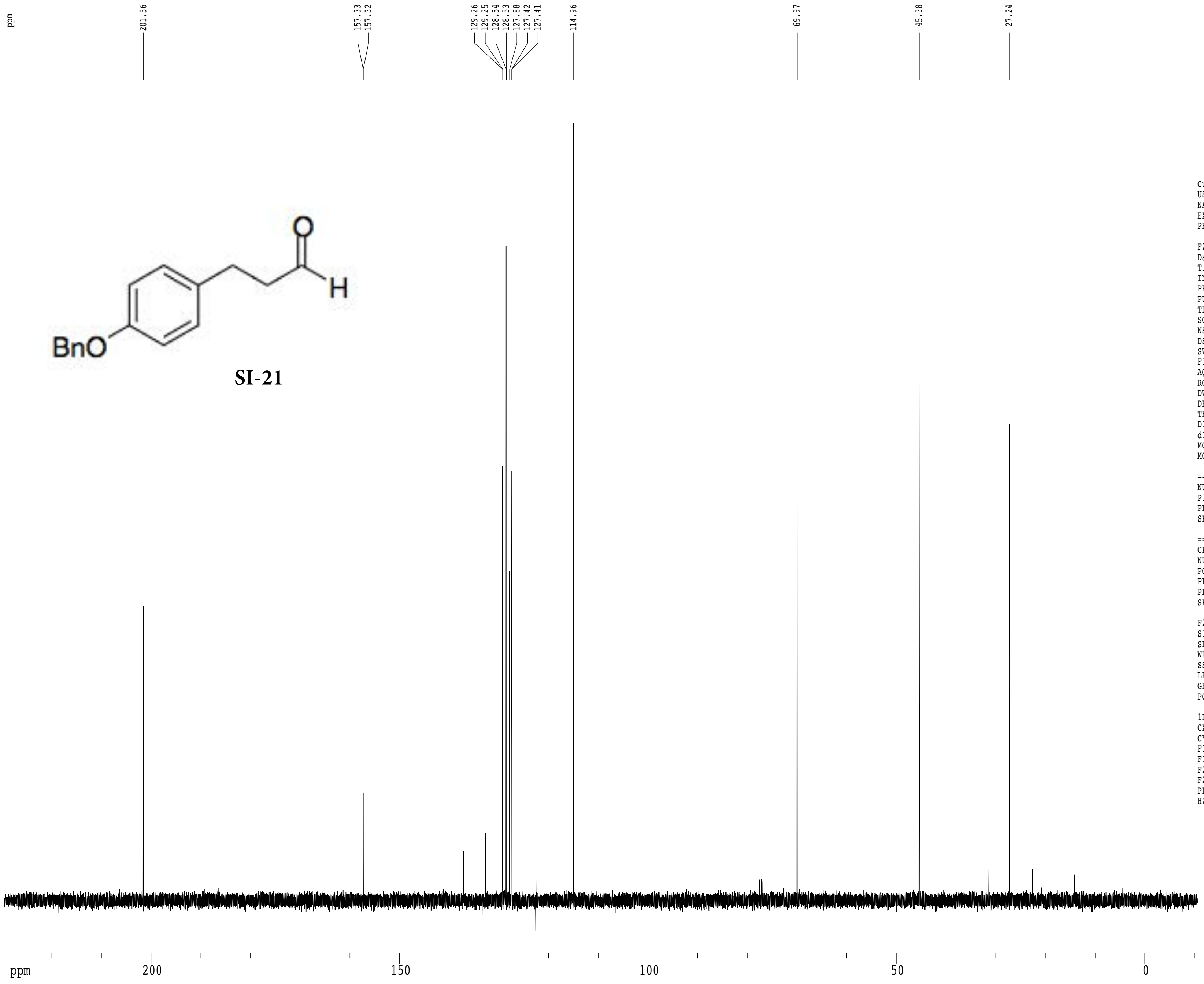

SI-21

```
Current Data Parameters
USER            khewitt1
NAME         KAH-V-265-1
EXPNO                  2
PROCNO                 1

F2 - Acquisition Parameters
Date_           20220205
Time               16.34
INSTRUM           drx400
PROBHD   5 mm QNP H/F/P
PULPROG          zgdc30
TD                 65536
SOLVENT            CDCl3
NS                    56
DS                     4
SWH           24154.590 Hz
FIDRES         0.368570 Hz
AQ            1.3566452 sec
RG                9195.2
DW                20.700 usec
DE                 20.39 usec
TE                 298.0 K
D1            0.10000000 sec
d11           0.03000000 sec
MCREST        0.00000000 sec
MCWRK         0.01500000 sec

======== CHANNEL f1 ========
NUC1                 13C
P1                  7.90 usec
PL1                -3.00 dB
SFO1         100.6237964 MHz

======== CHANNEL f2 ========
CPDPRG2          waltz16
NUC2                  1H
PCPD2              90.00 usec
PL2                -0.90 dB
PL12               17.00 dB
SFO2         400.1328009 MHz

F2 - Processing parameters
SI                 65536
SF           100.6127838 MHz
WDW                   no
SSB                    0
LB                  0.00 Hz
GB                     0
PC                  1.00

1D NMR plot parameters
CX                 22.80 cm
CY                 15.50 cm
F1P              229.496 ppm
F1              23090.22 Hz
F2P              -10.579 ppm
F2              -1064.37 Hz
PPMCM           10.52959 ppm/cm
HZCM          1059.41174 Hz/cm
```

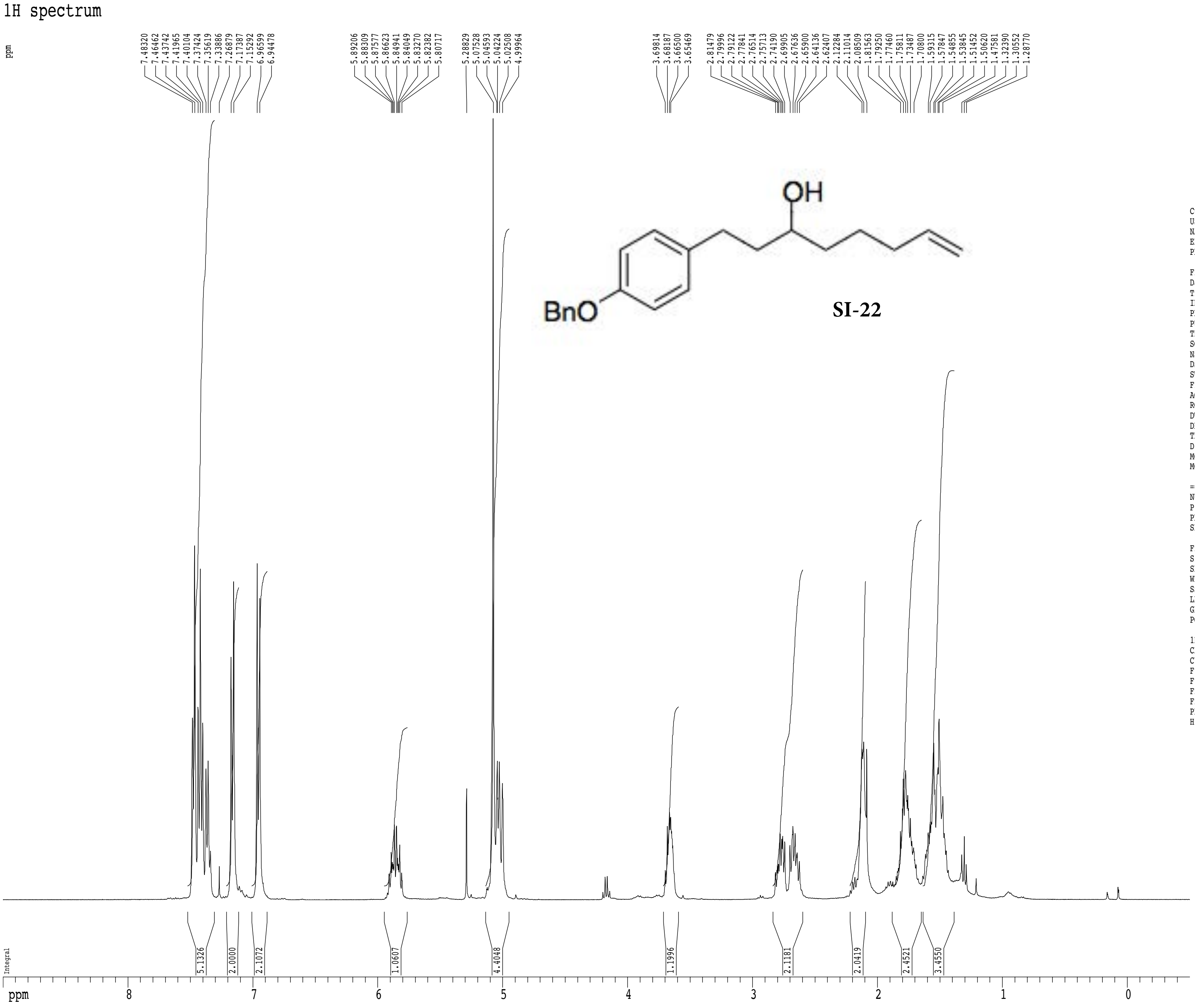

Current Data Parameters
USER khewitt1
NAME KAH-V-268-1
EXPNO 1
PROCNO 1

F2 - Acquisition Parameters
Date_ 20220207
Time 8.43
INSTRUM drx400
PROBHD 5 mm QNP H/F/P
PULPROG zg30
TD 38460
SOLVENT CDCl3T
NS 8
DS 2
SWH 6410.256 Hz
FIDRES 0.166673 Hz
AQ 2.9999299 sec
RG 32
DW 78.000 usec
DE 4.50 usec
TE 298.0 K
D1 0.10000000 sec
MCREST 0.00000000 sec
MCWRK 0.01500000 sec

======== CHANNEL f1 ========
NUC1 1H
P1 12.00 usec
PL1 -0.90 dB
SFO1 400.1328009 MHz

F2 - Processing parameters
SI 65536
SF 400.1300175 MHz
WDW no
SSB 0
LB 0.00 Hz
GB 0
PC 2.00

1D NMR plot parameters
CX 22.80 cm
CY 15.00 cm
F1P 9.000 ppm
F1 3601.17 Hz
F2P -0.500 ppm
F2 -200.06 Hz
PPMCM 0.41667 ppm/cm
HZCM 166.72084 Hz/cm

13C spectrum with 1H decoupling

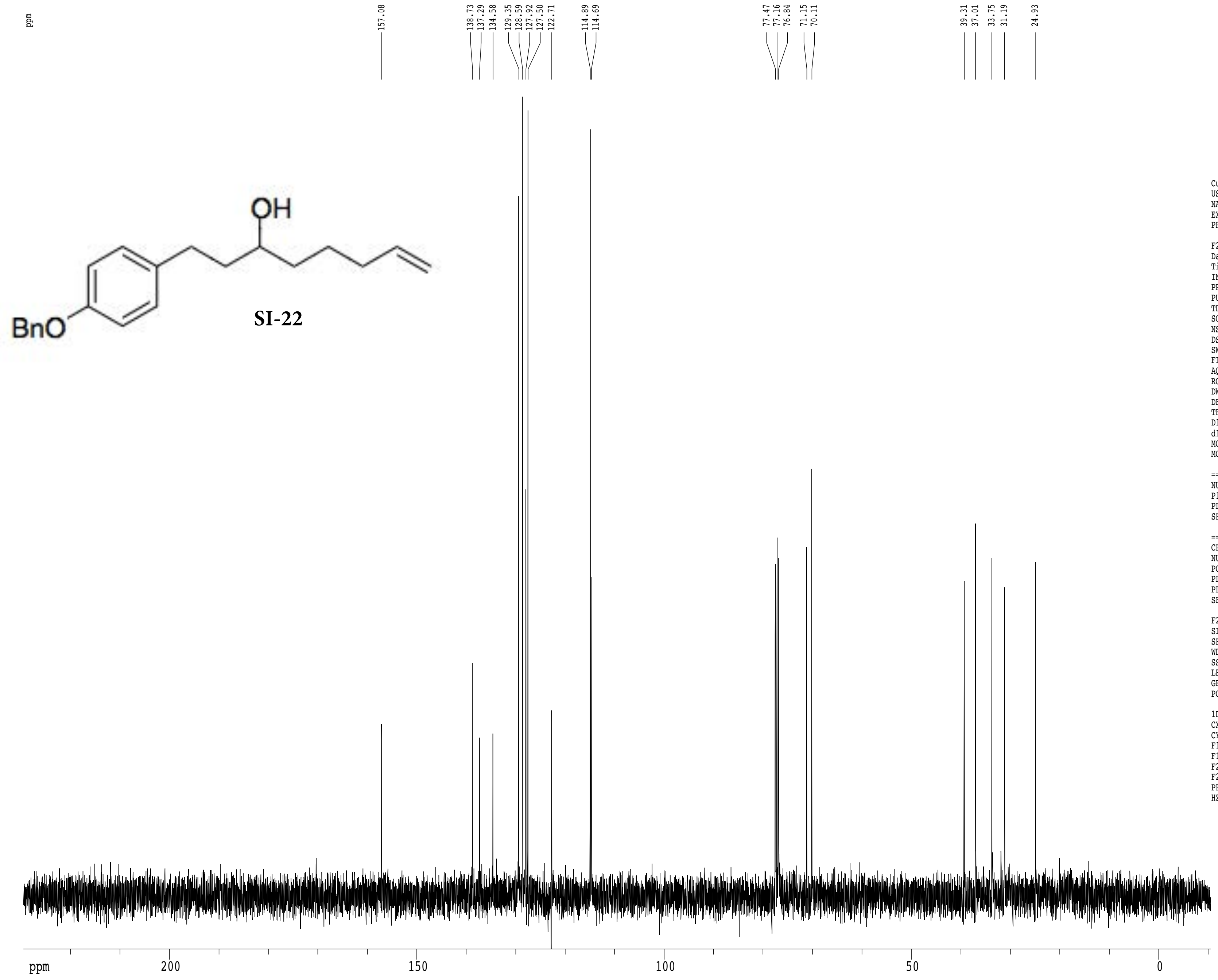

Current Data Parameters
USER khewitt1
NAME KAH-V-268-1
EXPNO 2
PROCNO 1

F2 - Acquisition Parameters
Date_ 20220207
Time 8.44
INSTRUM drx400
PROBHD 5 mm QNP H/F/P
PULPROG zgdc30
TD 65536
SOLVENT CDCl3
NS 32
DS 4
SWH 24154.590 Hz
FIDRES 0.368570 Hz
AQ 1.3566452 sec
RG 9195.2
DW 20.700 usec
DE 20.39 usec
TE 298.0 K
D1 0.10000000 sec
d11 0.03000000 sec
MCREST 0.00000000 sec
MCWRK 0.01500000 sec

======== CHANNEL f1 ========
NUC1 13C
P1 7.90 usec
PL1 -3.00 dB
SFO1 100.6237964 MHz

======== CHANNEL f2 ========
CPDPRG2 waltz16
NUC2 1H
PCPD2 90.00 usec
PL2 -0.90 dB
PL12 17.00 dB
SFO2 400.1328009 MHz

F2 - Processing parameters
SI 65536
SF 100.6127731 MHz
WDW EM
SSB 0
LB 1.00 Hz
GB 0
PC 1.00

1D NMR plot parameters
CX 22.80 cm
CY 15.50 cm
F1P 229.496 ppm
F1 23090.22 Hz
F2P -10.579 ppm
F2 -1064.37 Hz
PPMCM 10.52959 ppm/cm
HZCM 1059.41162 Hz/cm

1H spectrum

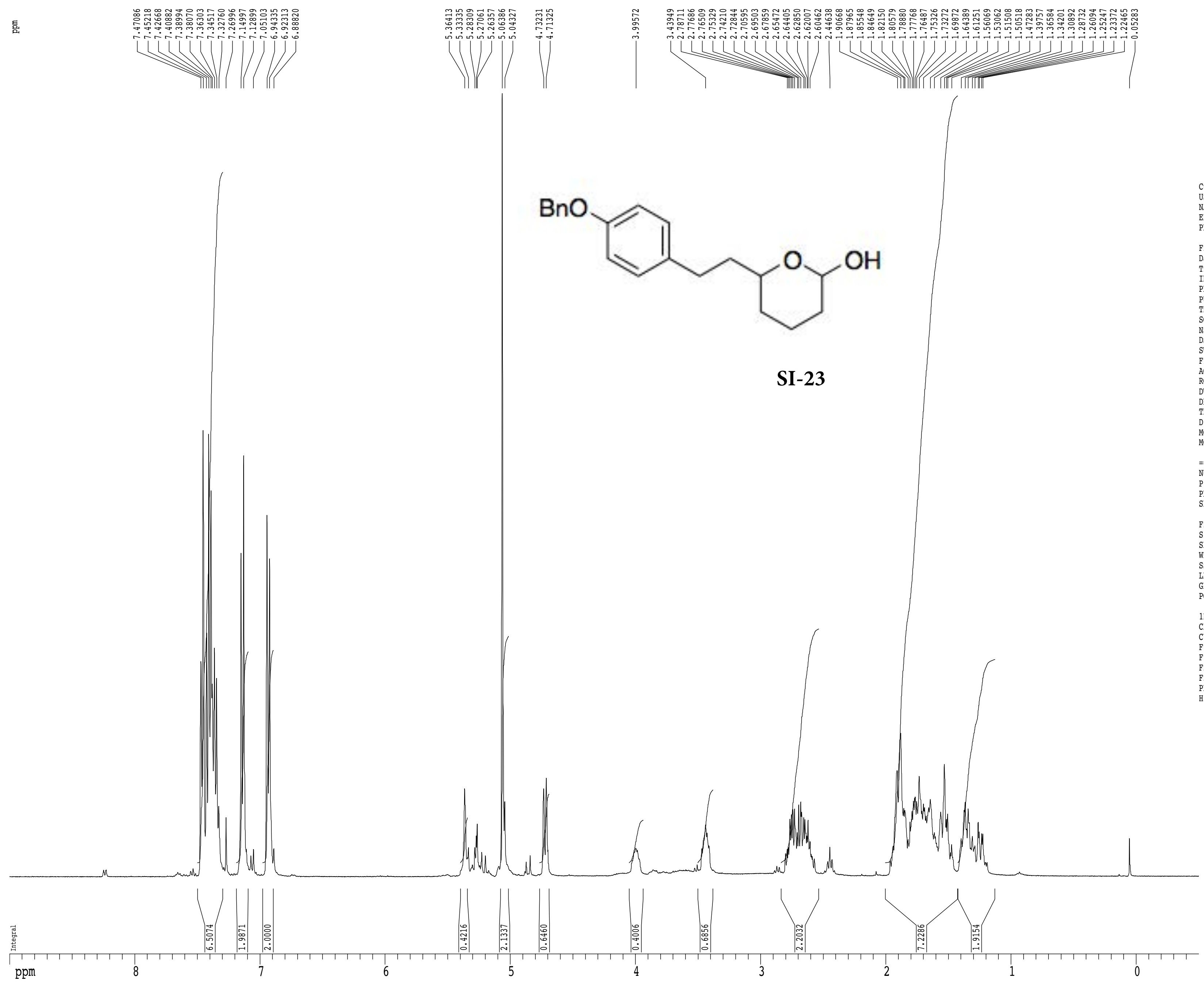

SI-23

```
Current Data Parameters
USER            khewitt1
NAME          KAH-V-287-Z
EXPNO                  1
PROCNO                 1

F2 - Acquisition Parameters
Date_           20220222
Time               16.43
INSTRUM           drx400
PROBHD   5 mm QNP H/F/P
PULPROG             zg30
TD                 38460
SOLVENT           CDCl3T
NS                     8
DS                     2
SWH             6410.256 Hz
FIDRES          0.166673 Hz
AQ             2.9999299 sec
RG                    57
DW                78.000 usec
DE                  4.50 usec
TE                 298.0 K
D1            0.10000000 sec
MCREST        0.00000000 sec
MCWRK         0.01500000 sec

======== CHANNEL f1 ========
NUC1                  1H
P1                 12.00 usec
PL1                -0.90 dB
SFO1         400.1328009 MHz

F2 - Processing parameters
SI                 65536
SF           400.1300175 MHz
WDW                   no
SSB                    0
LB                  0.00 Hz
GB                     0
PC                  2.00

1D NMR plot parameters
CX                 22.80 cm
CY                 15.00 cm
F1P                9.000 ppm
F1               3601.17 Hz
F2P               -0.500 ppm
F2               -200.06 Hz
PPMCM            0.41667 ppm/cm
HZCM           166.72084 Hz/cm
```

13C spectrum with 1H decoupling

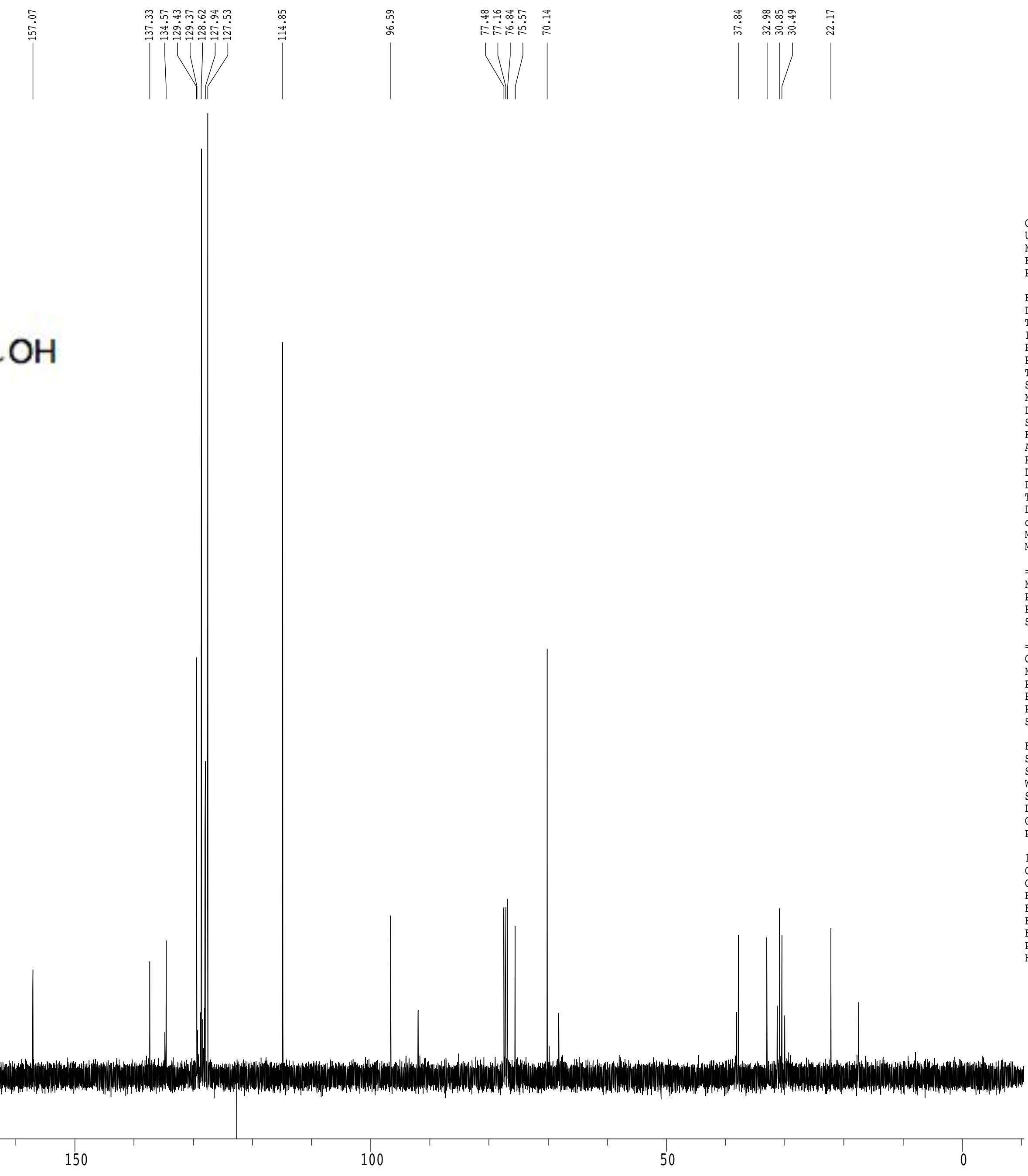

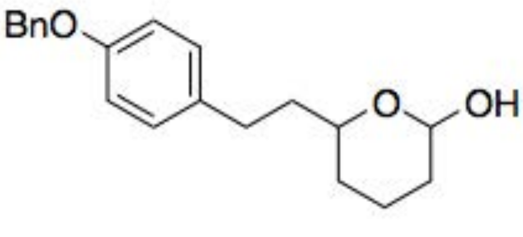

SI-23

```
Current Data Parameters
USER              khewitt1
NAME           KAH-V-287-Z
EXPNO                    2
PROCNO                   1

F2 - Acquisition Parameters
Date_             20220222
Time                 16.44
INSTRUM             drx400
PROBHD   5 mm QNP H/F/P
PULPROG            zgdc30
TD                   65536
SOLVENT              CDCl3
NS                      88
DS                       4
SWH              24154.590 Hz
FIDRES            0.368570 Hz
AQ               1.3566452 sec
RG                   13004
DW                  20.700 usec
DE                   20.39 usec
TE                   298.0 K
D1              0.10000000 sec
d11             0.03000000 sec
MCREST          0.00000000 sec
MCWRK           0.01500000 sec

======== CHANNEL f1 ========
NUC1                   13C
P1                    7.90 usec
PL1                  -3.00 dB
SFO1           100.6237964 MHz

======== CHANNEL f2 ========
CPDPRG2            waltz16
NUC2                    1H
PCPD2                90.00 usec
PL2                  -0.90 dB
PL12                 17.00 dB
SFO2           400.1328009 MHz

F2 - Processing parameters
SI                   65536
SF             100.6127687 MHz
WDW                     no
SSB                      0
LB                    0.00 Hz
GB                       0
PC                    1.00

1D NMR plot parameters
CX                   22.80 cm
CY                   15.50 cm
F1P                229.496 ppm
F1                23090.22 Hz
F2P                -10.579 ppm
F2                -1064.37 Hz
PPMCM             10.52959 ppm/cm
HZCM            1059.41162 Hz/cm
```

1H spectrum

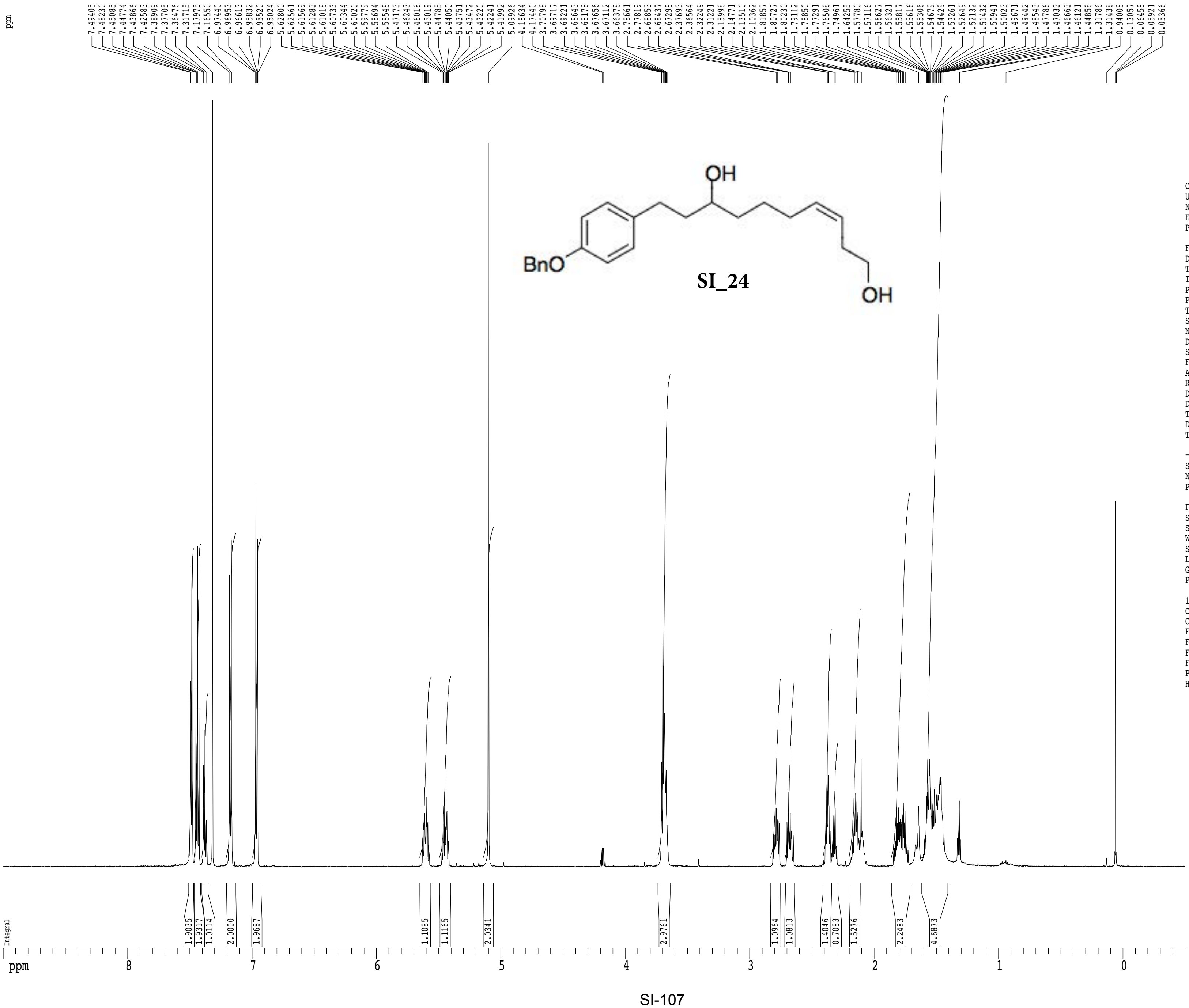


```
Current Data Parameters
USER          khewitt1
NAME     KAH-V-291-600-Z
EXPNO                  2
PROCNO                 1

F2 - Acquisition Parameters
Date_           20220225
Time               14.35
INSTRUM            av600
PROBHD   5 mm CPBBO BB-
PULPROG             zg30
TD                 98074
SOLVENT            CDCl3T
NS                     8
DS                     2
SWH            9615.385 Hz
FIDRES         0.098042 Hz
AQ            5.0998979 sec
RG                  25.4
DW                52.000 usec
DE                 14.23 usec
TE                 297.9 K
D1            0.10000000 sec
TD0                    1

======== CHANNEL f1 ========
SFO1        600.1342009 MHz
NUC1                  1H
P1                  9.50 usec

F2 - Processing parameters
SI                 65536
SF          600.1300000 MHz
WDW                   no
SSB                    0
LB                  0.00 Hz
GB                     0
PC                  1.00

1D NMR plot parameters
CX                 22.80 cm
CY                 15.00 cm
F1P                9.000 ppm
F1               5401.17 Hz
F2P               -0.500 ppm
F2               -300.06 Hz
PPMCM            0.41667 ppm/cm
HZCM           250.05418 Hz/cm
```

13C spectrum

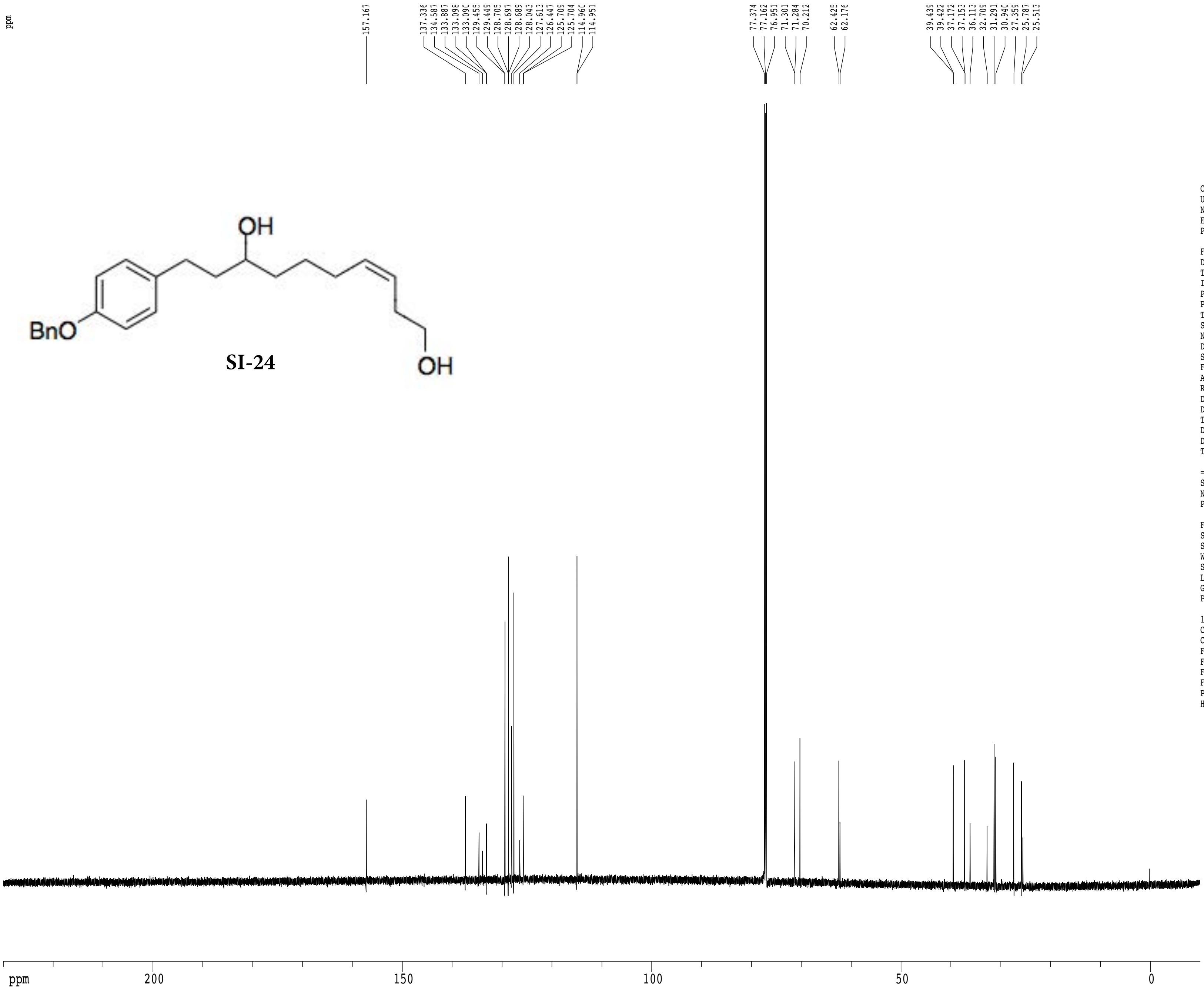

SI-24

Current Data Parameters
USER khewitt1
NAME KAH-V-291-600-Z
EXPNO 1
PROCNO 1

F2 - Acquisition Parameters
Date_ 20220225
Time 14.29
INSTRUM av600
PROBHD 5 mm CPBBO BB-
PULPROG zgdc30
TD 65536
SOLVENT CDCl3T
NS 180
DS 4
SWH 36231.883 Hz
FIDRES 0.552855 Hz
AQ 0.9044468 sec
RG 2050
DW 13.800 usec
DE 19.63 usec
TE 297.9 K
D1 0.40000001 sec
D11 0.03000000 sec
TD0 1

======== CHANNEL f1 ========
SFO1 150.9194080 MHz
NUC1 13C
P1 10.10 usec

F2 - Processing parameters
SI 65536
SF 150.9027942 MHz
WDW no
SSB 0
LB 0.00 Hz
GB 0
PC 1.00

1D NMR plot parameters
CX 22.80 cm
CY 15.00 cm
F1P 230.000 ppm
F1 34707.64 Hz
F2P -10.000 ppm
F2 -1509.03 Hz
PPMCM 10.52632 ppm/cm
HZCM 1588.45068 Hz/cm

1H spectrum

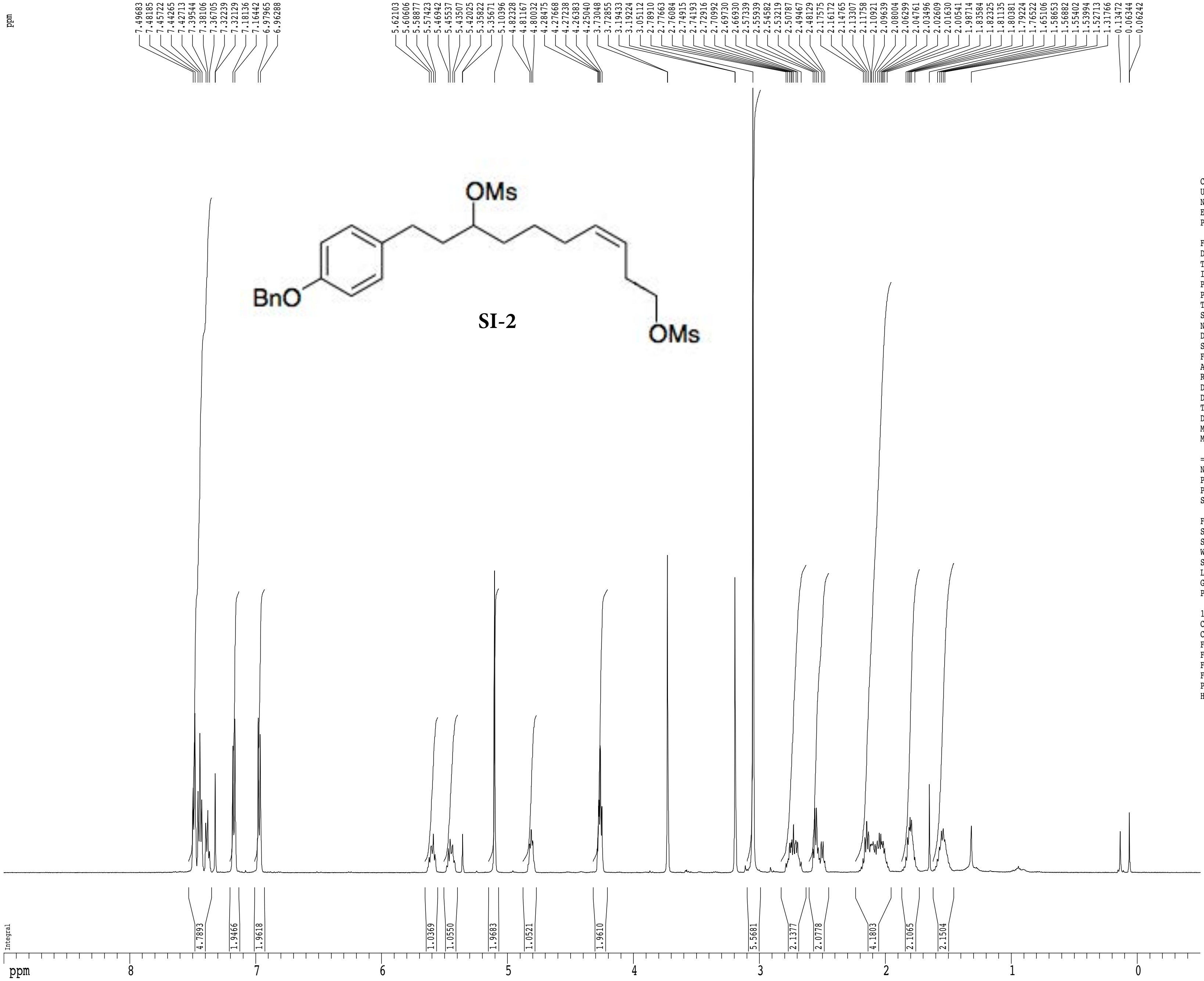

Current Data Parameters
USER khewitt1
NAME KAH-V-299-Z
EXPNO 1
PROCNO 1

F2 - Acquisition Parameters
Date_ 20220226
Time 16.13
INSTRUM cryo500
PROBHD 5 mm CPTCI 1H-
PULPROG zg30
TD 48074
SOLVENT CDCl3T
NS 8
DS 2
SWH 8012.820 Hz
FIDRES 0.166677 Hz
AQ 2.9998677 sec
RG 5
DW 62.400 usec
DE 6.00 usec
TE 298.0 K
D1 0.10000000 sec
MCREST 0.00000000 sec
MCWRK 0.01500000 sec

======== CHANNEL f1 ========
NUC1 1H
P1 9.75 usec
PL1 1.60 dB
SFO1 500.2235015 MHz

F2 - Processing parameters
SI 65536
SF 500.2200000 MHz
WDW no
SSB 0
LB 0.00 Hz
GB 0
PC 1.00

1D NMR plot parameters
CX 22.80 cm
CY 15.00 cm
F1P 9.000 ppm
F1 4501.98 Hz
F2P -0.500 ppm
F2 -250.11 Hz
PPMCM 0.41667 ppm/cm
HZCM 208.42500 Hz/cm

Z-restored spin-echo 13C spectrum with 1H decoupling

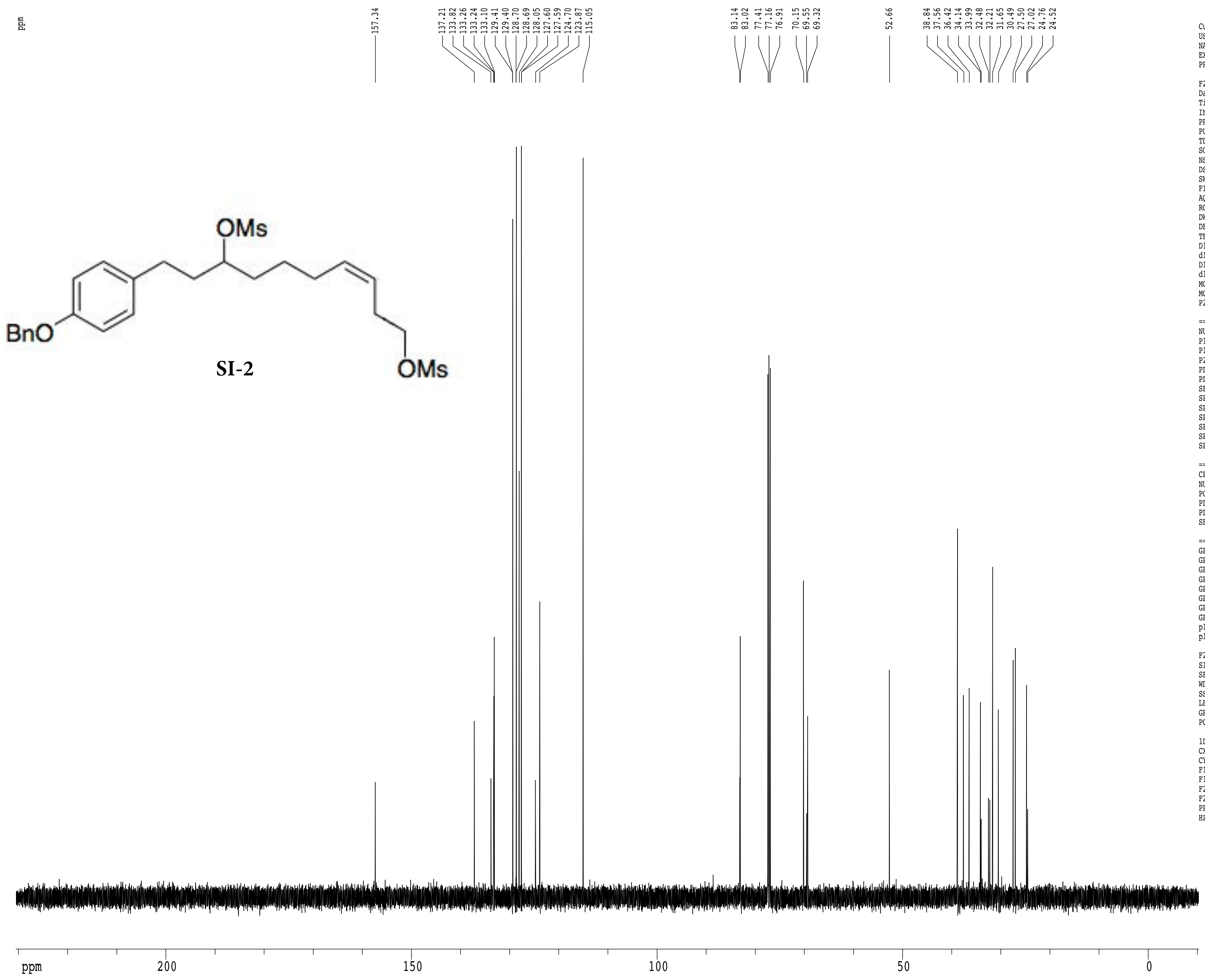


```
Current Data Parameters
USER            khewitt1
NAME         KAH-V-299-Z
EXPNO                  2
PROCNO                 1

F2 - Acquisition Parameters
Date_           20220226
Time               16.16
INSTRUM          cryo500
PROBHD   5 mm CPTCI 1H-
PULPROG  SpinEchopg30gp2.prd
TD                 65536
SOLVENT            CDCl3
NS                   144
DS                    16
SWH            30303.031 Hz
FIDRES          0.462388 Hz
AQ             1.0813940 sec
RG                  4096
DW                16.500 usec
DE                  6.00 usec
TE                 298.0 K
D1            0.25000000 sec
d11           0.03000000 sec
D16           0.00020000 sec
d17           0.00019600 sec
MCREST        0.00000000 sec
MCWRK         0.01500000 sec
P2                 37.70 usec

======== CHANNEL f1 ========
NUC1                 13C
P1                 18.85 usec
P12              2000.00 usec
P20               500.00 usec
PL0               120.00 dB
PL1                -1.00 dB
SFO1         125.7942548 MHz
SP2                 1.55 dB
SP4                 1.55 dB
SPNAM2       Crp60comp.4
SPNAM4    Crp60,0.5,20.1
SPOFF2              0.00 Hz
SPOFF4              0.00 Hz

======== CHANNEL f2 ========
CPDPRG2          waltz16
NUC2                  1H
PCPD2             100.00 usec
PL2                 1.60 dB
PL12               22.00 dB
SFO2         500.2225011 MHz

====== GRADIENT CHANNEL =====
GPNAM1          SINE.100
GPNAM2          SINE.100
GPX1                0.00 %
GPX2                0.00 %
GPY1                0.00 %
GPY2                0.00 %
GPZ1               30.00 %
GPZ2               50.00 %
p15               500.00 usec
p16              1000.00 usec

F2 - Processing parameters
SI                 65536
SF           125.7804122 MHz
WDW                   no
SSB                    0
LB                  0.00 Hz
GB                     0
PC                  2.00

1D NMR plot parameters
CX                 22.80 cm
CY                 15.65 cm
F1P              230.637 ppm
F1              29009.68 Hz
F2P              -10.287 ppm
F2              -1293.96 Hz
PPMCM           10.56688 ppm/cm
HZCM          1329.10693 Hz/cm
```

1H spectrum

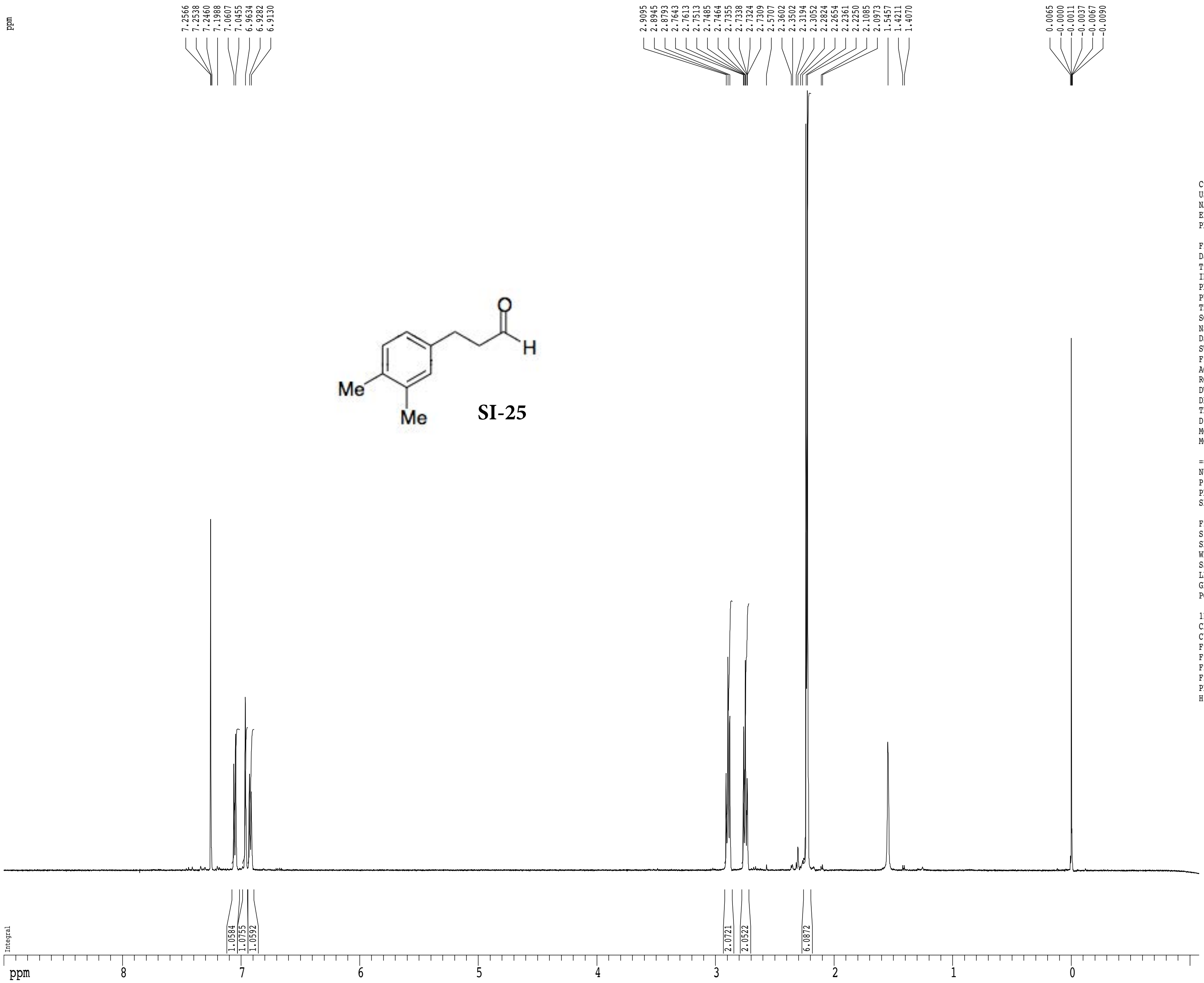

SI-25

```
Current Data Parameters
USER          caherber
NAME     CAH-I-276-full
EXPNO                1
PROCNO               1

F2 - Acquisition Parameters
Date_         20210921
Time              9.21
INSTRUM        cryo500
PROBHD   5 mm CPTCI 1H-
PULPROG           zg30
TD               81728
SOLVENT          CDCl3
NS                   8
DS                   2
SWH           8012.820 Hz
FIDRES        0.098043 Hz
AQ           5.0998774 sec
RG                 5.7
DW              62.400 usec
DE                6.00 usec
TE               298.0 K
D1          0.10000000 sec
MCREST      0.00000000 sec
MCWRK       0.01500000 sec

======== CHANNEL f1 ========
NUC1                1H
P1                9.75 usec
PL1               1.60 dB
SFO1       500.2235015 MHz

F2 - Processing parameters
SI               65536
SF         500.2200335 MHz
WDW                 no
SSB                  0
LB                0.00 Hz
GB                   0
PC                1.00

1D NMR plot parameters
CX               22.80 cm
CY               15.00 cm
F1P              9.000 ppm
F1             4501.98 Hz
F2P             -1.076 ppm
F2             -538.34 Hz
PPMCM          0.44194 ppm/cm
HZCM         221.06656 Hz/cm
```

Z-restored spin-echo 13C spectrum with 1H decoupling

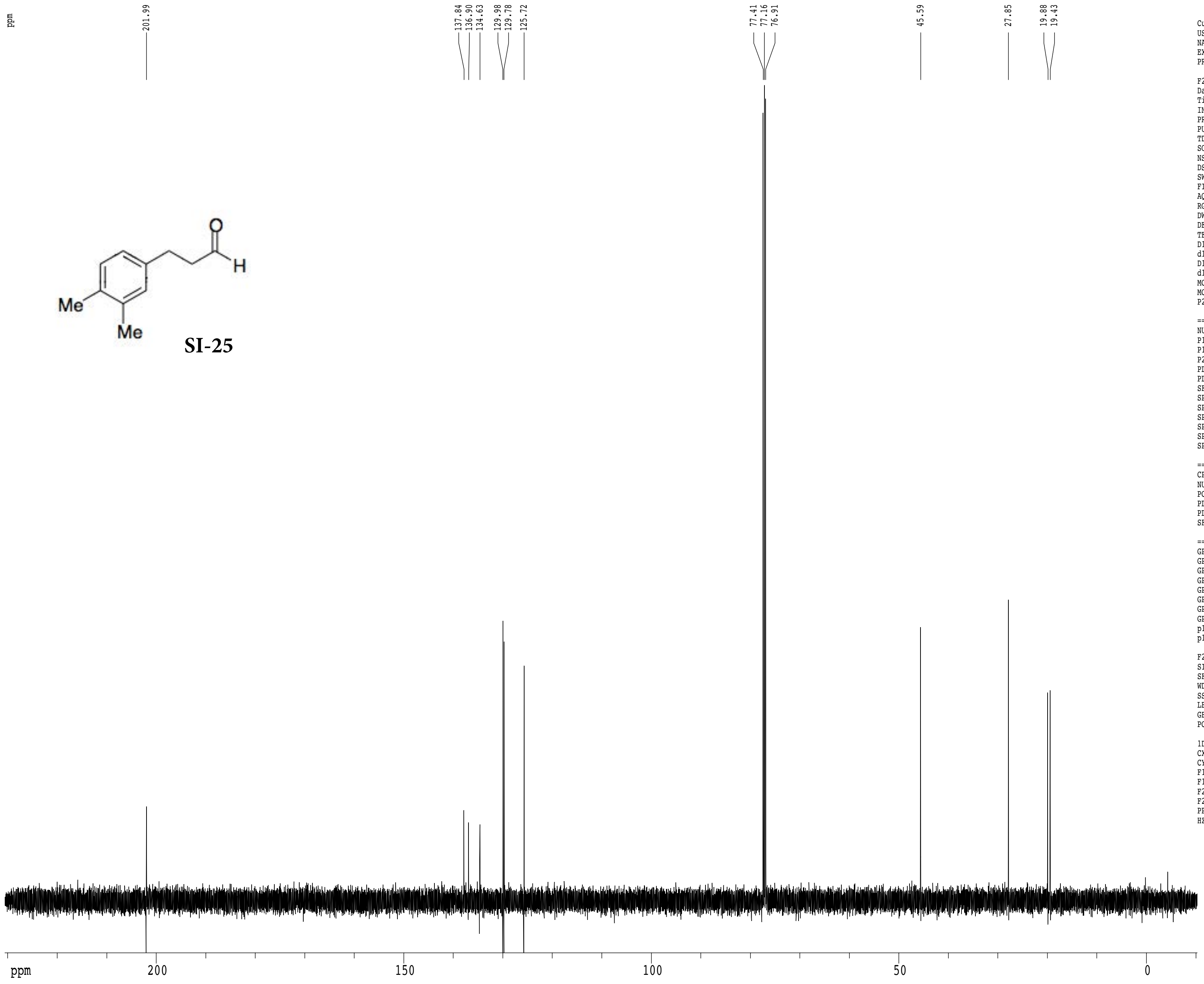

SI-25

```
Current Data Parameters
USER          caherber
NAME     CAH-I-276-full
EXPNO                 2
PROCNO                1

F2 - Acquisition Parameters
Date_          20210921
Time               9.28
INSTRUM         cryo500
PROBHD   5 mm CPTCI 1H-
PULPROG  SpinEchopg30gp2.prd
TD                65536
SOLVENT           CDCl3
NS                  240
DS                   16
SWH           30303.031 Hz
FIDRES         0.462388 Hz
AQ            1.0813940 sec
RG               7298.2
DW               16.500 usec
DE                 6.00 usec
TE                298.0 K
D1           0.25000000 sec
d11          0.03000000 sec
D16          0.00020000 sec
d17          0.00019600 sec
MCREST       0.00000000 sec
MCWRK        0.01500000 sec
P2                37.70 usec

======== CHANNEL f1 ========
NUC1                13C
P1                18.85 usec
P12             2000.00 usec
P20              500.00 usec
PL0              120.00 dB
PL1               -1.00 dB
SFO1        125.7942548 MHz
SP2                1.55 dB
SP4                1.55 dB
SPNAM2      Crp60comp.4
SPNAM4   Crp60,0.5,20.1
SPOFF2             0.00 Hz
SPOFF4             0.00 Hz

======== CHANNEL f2 ========
CPDPRG2         waltz16
NUC2                 1H
PCPD2            100.00 usec
PL2                1.60 dB
PL12              22.00 dB
SFO2        500.2225011 MHz

====== GRADIENT CHANNEL =====
GPNAM1         SINE.100
GPNAM2         SINE.100
GPX1               0.00 %
GPX2               0.00 %
GPY1               0.00 %
GPY2               0.00 %
GPZ1              30.00 %
GPZ2              50.00 %
p15              500.00 usec
p16             1000.00 usec

F2 - Processing parameters
SI                65536
SF          125.7804062 MHz
WDW                  no
SSB                   0
LB                 0.00 Hz
GB                    0
PC                 2.00

1D NMR plot parameters
CX                22.80 cm
CY                15.65 cm
F1P             230.637 ppm
F1             29009.68 Hz
F2P             -10.287 ppm
F2             -1293.96 Hz
PPMCM          10.56688 ppm/cm
HZCM         1329.10693 Hz/cm
```

1H spectrum

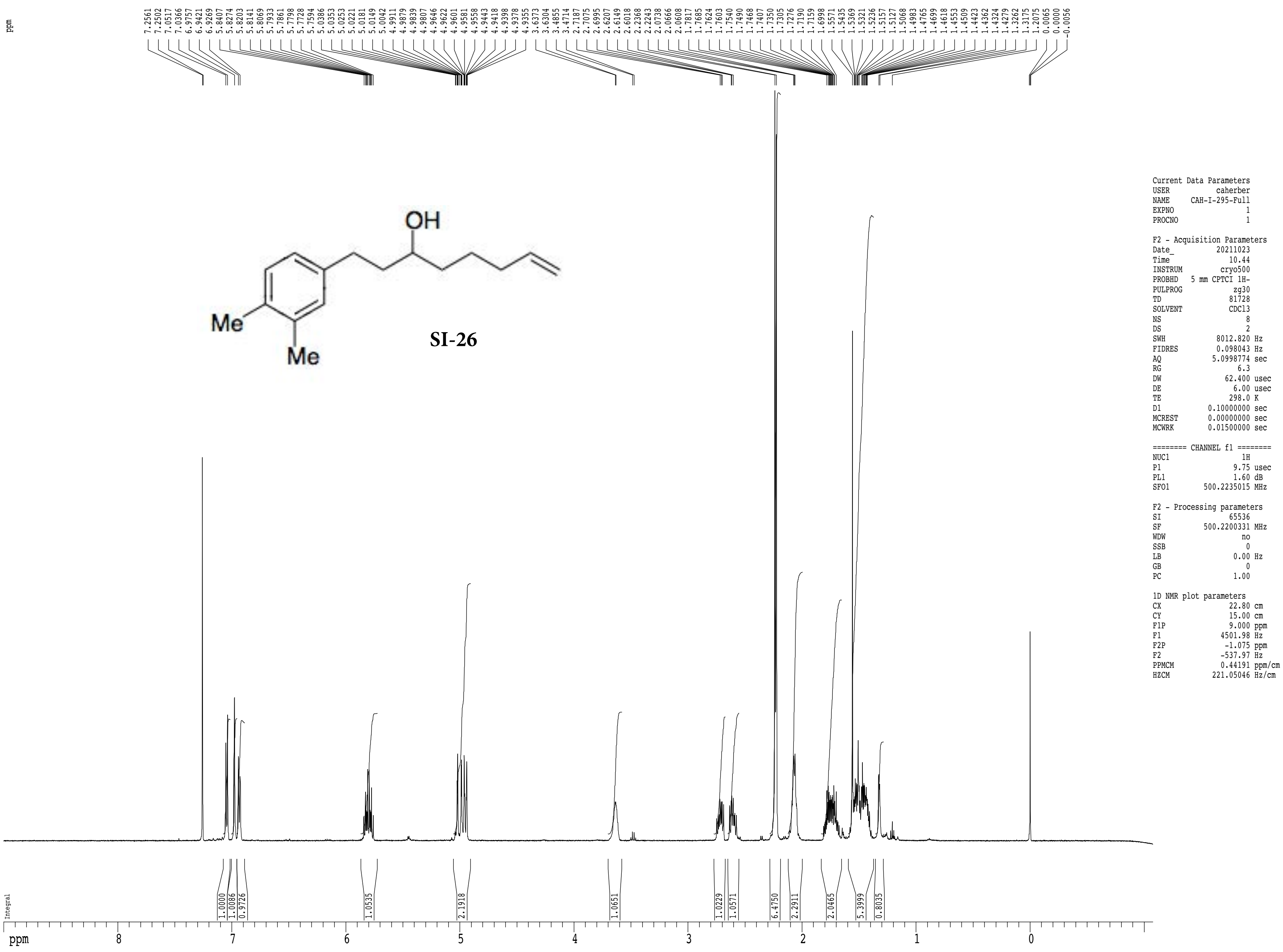
ppm
7.2561 7.2502 7.0517 7.0366 6.9757 6.9421 6.9269 5.8407 5.8274 5.8203 5.8141 5.8069 5.7933 5.7861 5.7798 5.7728 5.7594 5.0386 5.0353 5.0253 5.0221 5.0181 5.0149 5.0042 4.9911 4.9879 4.9839 4.9807 4.9646 4.9622 4.9601 4.9581 4.9558 4.9443 4.9418 4.9398 4.9378 4.9355 3.6373 3.6304 3.4855 3.4714 2.7187 2.7075 2.6995 2.6207 2.6149 2.6018 2.2368 2.2243 2.0738 2.0666 2.0608 1.7817 1.7685 1.7624 1.7603 1.7540 1.7490 1.7468 1.7407 1.7350 1.7305 1.7276 1.7190 1.7159 1.6998 1.5571 1.5435 1.5369 1.5321 1.5236 1.5157 1.5127 1.5068 1.4983 1.4765 1.4699 1.4618 1.4553 1.4509 1.4423 1.4362 1.4324 1.4279 1.3262 1.3175 1.2075 0.0065 0.0000 -0.0056
OH
Me
Me
SI-26
Current Data Parameters
USER caherber
NAME CAH-I-295-Full
EXPNO 1
PROCNO 1
F2 - Acquisition Parameters
Date_ 20211023
Time 10.44
INSTRUM cryo500
PROBHD 5 mm CPTCI 1H-
PULPROG zg30
TD 81728
SOLVENT CDCl3
NS 8
DS 2
SWH 8012.820 Hz
FIDRES 0.098043 Hz
AQ 5.0998774 sec
RG 6.3
DW 62.400 usec
DE 6.00 usec
TE 298.0 K
D1 0.10000000 sec
MCREST 0.00000000 sec
MCWRK 0.01500000 sec
======== CHANNEL f1 ========
NUC1 1H
P1 9.75 usec
PL1 1.60 dB
SFO1 500.2235015 MHz
F2 - Processing parameters
SI 65536
SF 500.2200331 MHz
WDW no
SSB 0
LB 0.00 Hz
GB 0
PC 1.00
1D NMR plot parameters
CX 22.80 cm
CY 15.00 cm
F1P 9.000 ppm
F1 4501.98 Hz
F2P -1.075 ppm
F2 -537.97 Hz
PPMCM 0.44191 ppm/cm
HZCM 221.05046 Hz/cm
Integral
1.0000 1.0086 0.9726 1.0535 2.1918 1.0651 1.0229 1.0571 6.4750 2.2911 2.0465 5.3999 0.8035
ppm 8 7 6 5 4 3 2 1 0

Z-restored spin-echo 13C spectrum with 1H decoupling

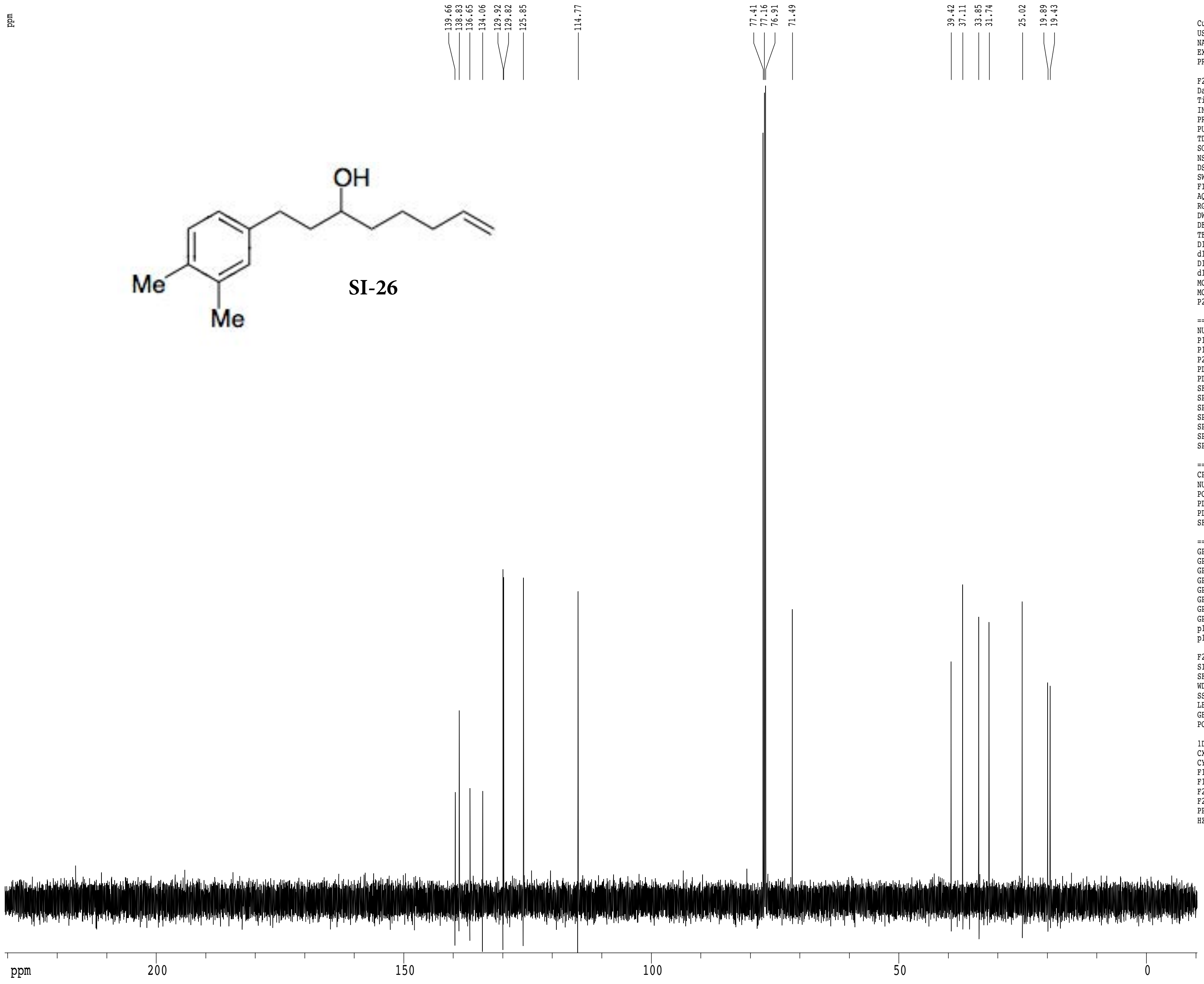


```
Current Data Parameters
USER          caherber
NAME     CAH-I-295-Full
EXPNO                 2
PROCNO                1

F2 - Acquisition Parameters
Date_          20211023
Time              10.49
INSTRUM         cryo500
PROBHD   5 mm CPTCI 1H-
PULPROG  SpinEchopg30gp2.prd
TD                65536
SOLVENT           CDCl3
NS                  216
DS                   16
SWH           30303.031 Hz
FIDRES         0.462388 Hz
AQ            1.0813940 sec
RG               2896.3
DW               16.500 usec
DE                 6.00 usec
TE                298.0 K
D1           0.25000000 sec
d11          0.03000000 sec
D16          0.00020000 sec
d17          0.00019600 sec
MCREST       0.00000000 sec
MCWRK        0.01500000 sec
P2                37.70 usec

======== CHANNEL f1 ========
NUC1                13C
P1                18.85 usec
P12             2000.00 usec
P20              500.00 usec
PL0              120.00 dB
PL1               -1.00 dB
SFO1        125.7942548 MHz
SP2                1.55 dB
SP4                1.55 dB
SPNAM2      Crp60comp.4
SPNAM4   Crp60,0.5,20.1
SPOFF2             0.00 Hz
SPOFF4             0.00 Hz

======== CHANNEL f2 ========
CPDPRG2         waltz16
NUC2                 1H
PCPD2            100.00 usec
PL2                1.60 dB
PL12              22.00 dB
SFO2        500.2225011 MHz

====== GRADIENT CHANNEL =====
GPNAM1         SINE.100
GPNAM2         SINE.100
GPX1               0.00 %
GPX2               0.00 %
GPY1               0.00 %
GPY2               0.00 %
GPZ1              30.00 %
GPZ2              50.00 %
p15              500.00 usec
p16             1000.00 usec

F2 - Processing parameters
SI                65536
SF          125.7804062 MHz
WDW                  no
SSB                   0
LB                 0.00 Hz
GB                    0
PC                 2.00

1D NMR plot parameters
CX                22.80 cm
CY                15.65 cm
F1P             230.637 ppm
F1             29009.68 Hz
F2P             -10.287 ppm
F2             -1293.96 Hz
PPMCM          10.56688 ppm/cm
HZCM         1329.10693 Hz/cm
```

1H spectrum

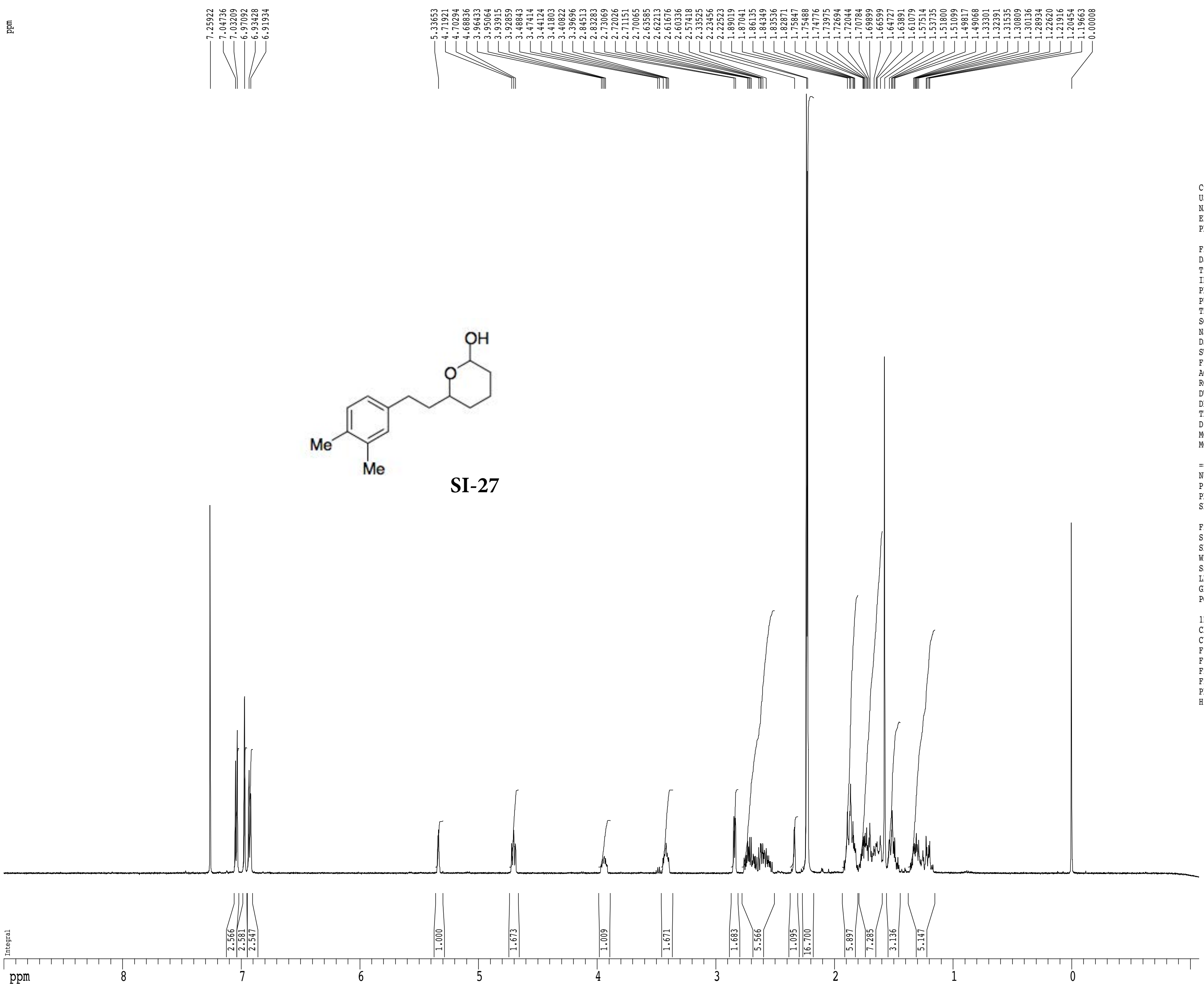


```
Current Data Parameters
USER          caherber
NAME     CAH-II-024-full
EXPNO                  1
PROCNO                 1

F2 - Acquisition Parameters
Date_          20211102
Time              14.54
INSTRUM         cryo500
PROBHD   5 mm CPTCI 1H-
PULPROG            zg30
TD                81728
SOLVENT           CDCl3
NS                    8
DS                    2
SWH            8012.820 Hz
FIDRES         0.098043 Hz
AQ            5.0998774 sec
RG                    5
DW               62.400 usec
DE                 6.00 usec
TE                298.0 K
D1           0.10000000 sec
MCREST       0.00000000 sec
MCWRK        0.01500000 sec

======== CHANNEL f1 ========
NUC1                 1H
P1                 9.75 usec
PL1                1.60 dB
SFO1        500.2235015 MHz

F2 - Processing parameters
SI                65536
SF          500.2200313 MHz
WDW                  no
SSB                   0
LB                 0.00 Hz
GB                    0
PC                 1.00

1D NMR plot parameters
CX                22.80 cm
CY                15.00 cm
F1P               9.000 ppm
F1              4501.98 Hz
F2P              -1.072 ppm
F2              -536.14 Hz
PPMCM           0.44175 ppm/cm
HZCM          220.97003 Hz/cm
```

Z-restored spin-echo 13C spectrum with 1H decoupling

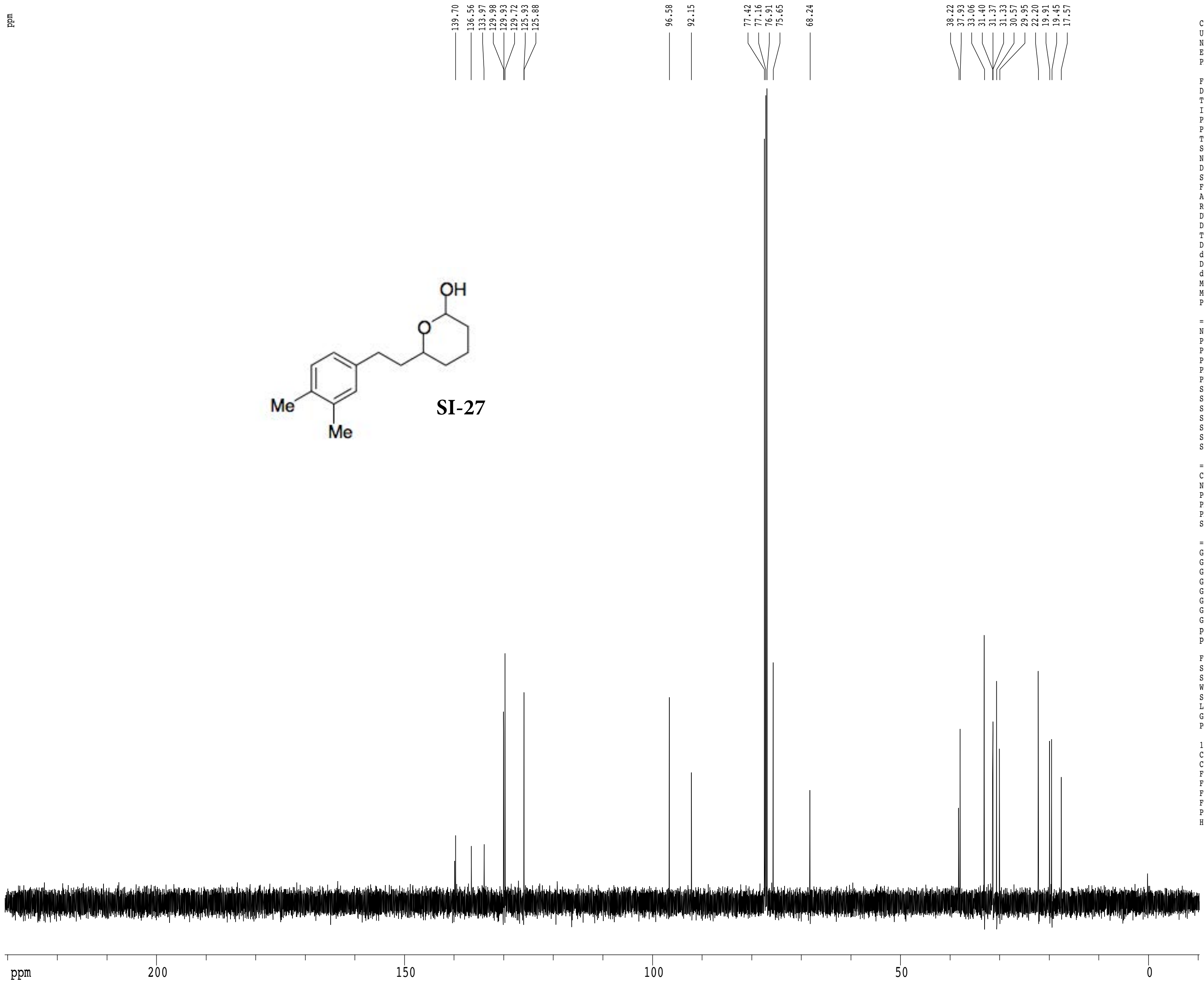

SI-27

Current Data Parameters
USER caherber
NAME CAH-II-024-full
EXPNO 2
PROCNO 1

F2 - Acquisition Parameters
Date_ 20211102
Time 15.06
INSTRUM cryo500
PROBHD 5 mm CPTCI 1H-
PULPROG SpinEchopg30gp2.prd
TD 65536
SOLVENT CDCl3
NS 520
DS 16
SWH 30303.031 Hz
FIDRES 0.462388 Hz
AQ 1.0813940 sec
RG 3251
DW 16.500 usec
DE 6.00 usec
TE 298.0 K
D1 0.25000000 sec
d11 0.03000000 sec
D16 0.00020000 sec
d17 0.00019600 sec
MCREST 0.00000000 sec
MCWRK 0.01500000 sec
P2 37.70 usec

======== CHANNEL f1 ========
NUC1 13C
P1 18.85 usec
P12 2000.00 usec
P20 500.00 usec
PL0 120.00 dB
PL1 -1.00 dB
SFO1 125.7942548 MHz
SP2 1.55 dB
SP4 1.55 dB
SPNAM2 Crp60comp.4
SPNAM4 Crp60,0.5,20.1
SPOFF2 0.00 Hz
SPOFF4 0.00 Hz

======== CHANNEL f2 ========
CPDPRG2 waltz16
NUC2 1H
PCPD2 100.00 usec
PL2 1.60 dB
PL12 22.00 dB
SFO2 500.2225011 MHz

====== GRADIENT CHANNEL =====
GPNAM1 SINE.100
GPNAM2 SINE.100
GPX1 0.00 %
GPX2 0.00 %
GPY1 0.00 %
GPY2 0.00 %
GPZ1 30.00 %
GPZ2 50.00 %
p15 500.00 usec
p16 1000.00 usec

F2 - Processing parameters
SI 65536
SF 125.7804076 MHz
WDW no
SSB 0
LB 0.00 Hz
GB 0
PC 2.00

1D NMR plot parameters
CX 22.80 cm
CY 15.65 cm
F1P 230.637 ppm
F1 29009.68 Hz
F2P -10.287 ppm
F2 -1293.96 Hz
PPMCM 10.56688 ppm/cm
HZCM 1329.10693 Hz/cm

1H spectrum

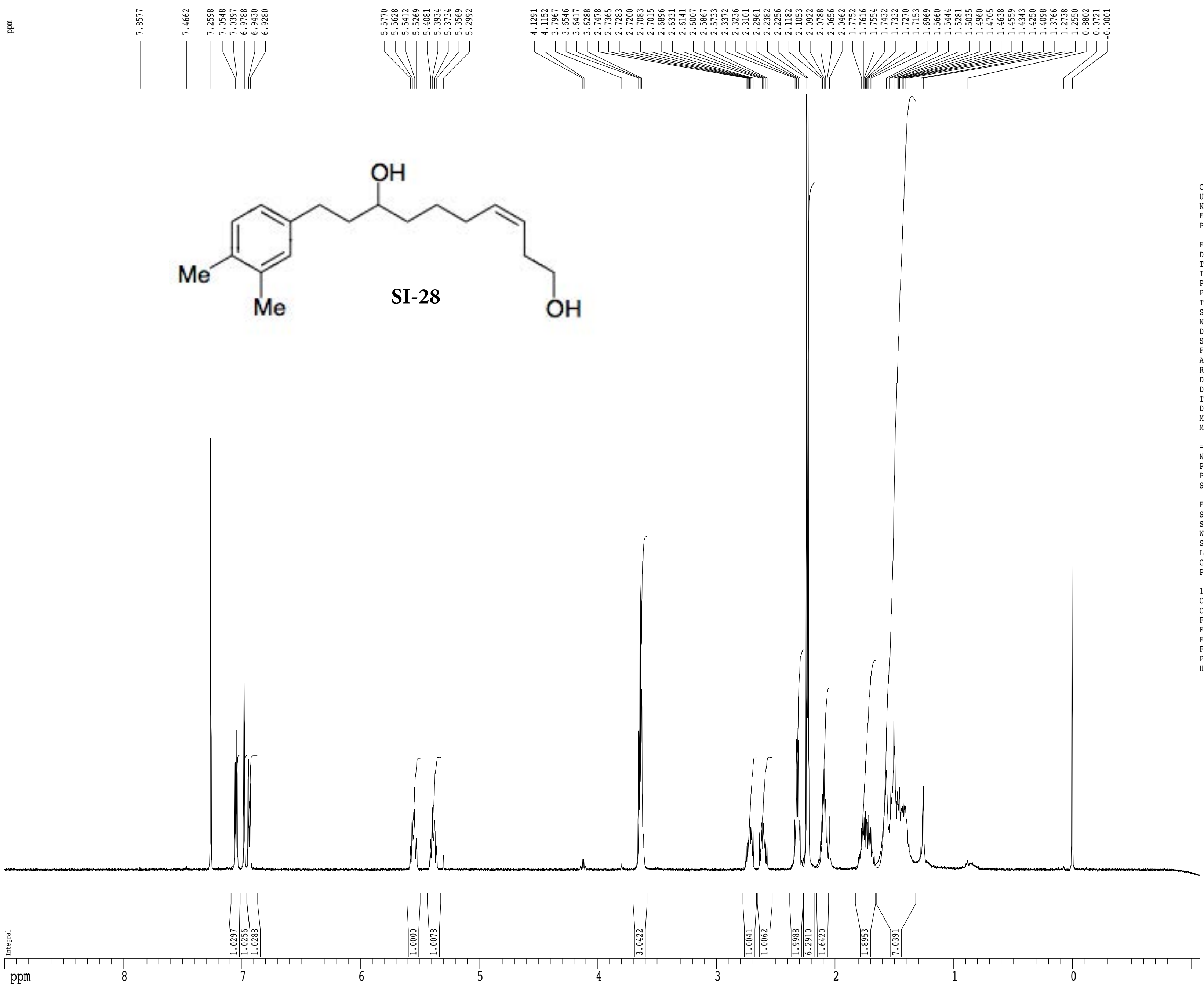

Current Data Parameters
USER caherber
NAME CAH-II-026-cis-full
EXPNO 1
PROCNO 1

F2 - Acquisition Parameters
Date_ 20211109
Time 8.47
INSTRUM cryo500
PROBHD 5 mm CPTCI 1H-
PULPROG zg30
TD 81728
SOLVENT CDCl3
NS 8
DS 2
SWH 8012.820 Hz
FIDRES 0.098043 Hz
AQ 5.0998774 sec
RG 5.7
DW 62.400 usec
DE 6.00 usec
TE 298.0 K
D1 0.10000000 sec
MCREST 0.00000000 sec
MCWRK 0.01500000 sec

======== CHANNEL f1 ========
NUC1 1H
P1 9.75 usec
PL1 1.60 dB
SFO1 500.2235015 MHz

F2 - Processing parameters
SI 65536
SF 500.2200309 MHz
WDW no
SSB 0
LB 0.00 Hz
GB 0
PC 1.00

1D NMR plot parameters
CX 22.80 cm
CY 15.00 cm
F1P 9.000 ppm
F1 4501.98 Hz
F2P -1.071 ppm
F2 -535.77 Hz
PPMCM 0.44171 ppm/cm
HZCM 220.95406 Hz/cm

Z-restored spin-echo 13C spectrum with 1H decoupling

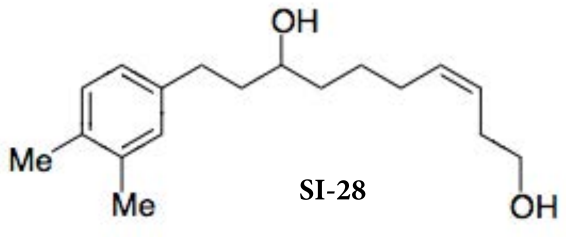

SI-28

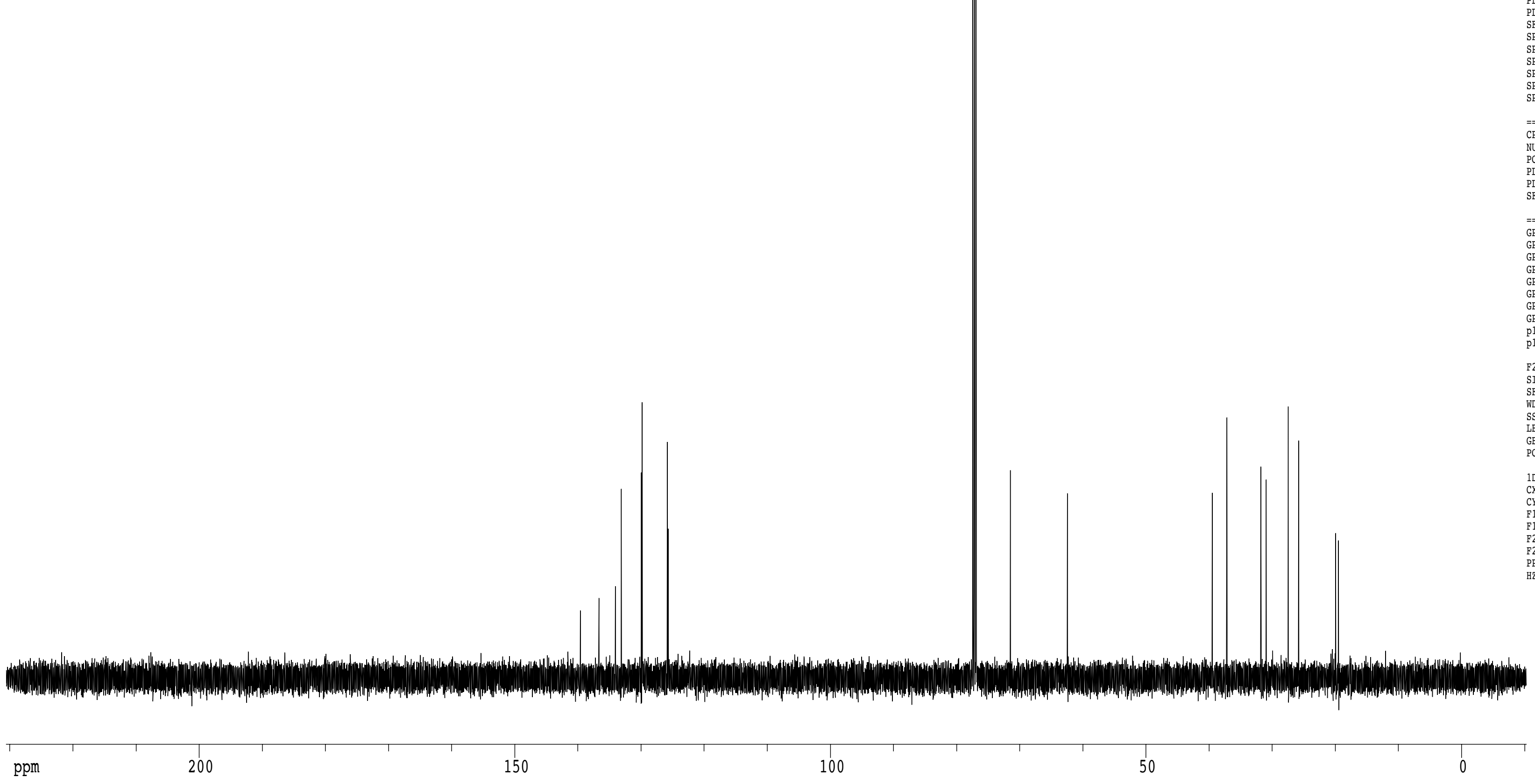

Current Data Parameters

USER	caherber
NAME	CAH-II-026-cis-full
EXPNO	2
PROCNO	1

F2 - Acquisition Parameters

Date_	20211109
Time	8.55
INSTRUM	cryo500
PROBHD	5 mm CPTCI 1H-
PULPROG	SpinEchopg30gp2.prd
TD	65536
SOLVENT	CDCl3
NS	424
DS	16
SWH	30303.031 Hz
FIDRES	0.462388 Hz
AQ	1.0813940 sec
RG	11585.2
DW	16.500 usec
DE	6.00 usec
TE	298.0 K
D1	0.25000000 sec
d11	0.03000000 sec
D16	0.00020000 sec
d17	0.00019600 sec
MCREST	0.00000000 sec
MCWRK	0.01500000 sec
P2	37.70 usec

======== CHANNEL f1 ========

NUC1	13C
P1	18.85 usec
P12	2000.00 usec
P20	500.00 usec
PL0	120.00 dB
PL1	-1.00 dB
SFO1	125.7942548 MHz
SP2	1.55 dB
SP4	1.55 dB
SPNAM2	Crp60comp.4
SPNAM4	Crp60,0.5,20.1
SPOFF2	0.00 Hz
SPOFF4	0.00 Hz

======== CHANNEL f2 ========

CPDPRG2	waltz16
NUC2	1H
PCPD2	100.00 usec
PL2	1.60 dB
PL12	22.00 dB
SFO2	500.2225011 MHz

====== GRADIENT CHANNEL =====

GPNAM1	SINE.100
GPNAM2	SINE.100
GPX1	0.00 %
GPX2	0.00 %
GPY1	0.00 %
GPY2	0.00 %
GPZ1	30.00 %
GPZ2	50.00 %
p15	500.00 usec
p16	1000.00 usec

F2 - Processing parameters

SI	65536
SF	125.7804076 MHz
WDW	no
SSB	0
LB	0.00 Hz
GB	0
PC	2.00

1D NMR plot parameters

CX	22.80 cm
CY	15.65 cm
F1P	230.637 ppm
F1	29009.68 Hz
F2P	-10.287 ppm
F2	-1293.96 Hz
PPMCM	10.56688 ppm/cm
HZCM	1329.10693 Hz/cm

1H spectrum

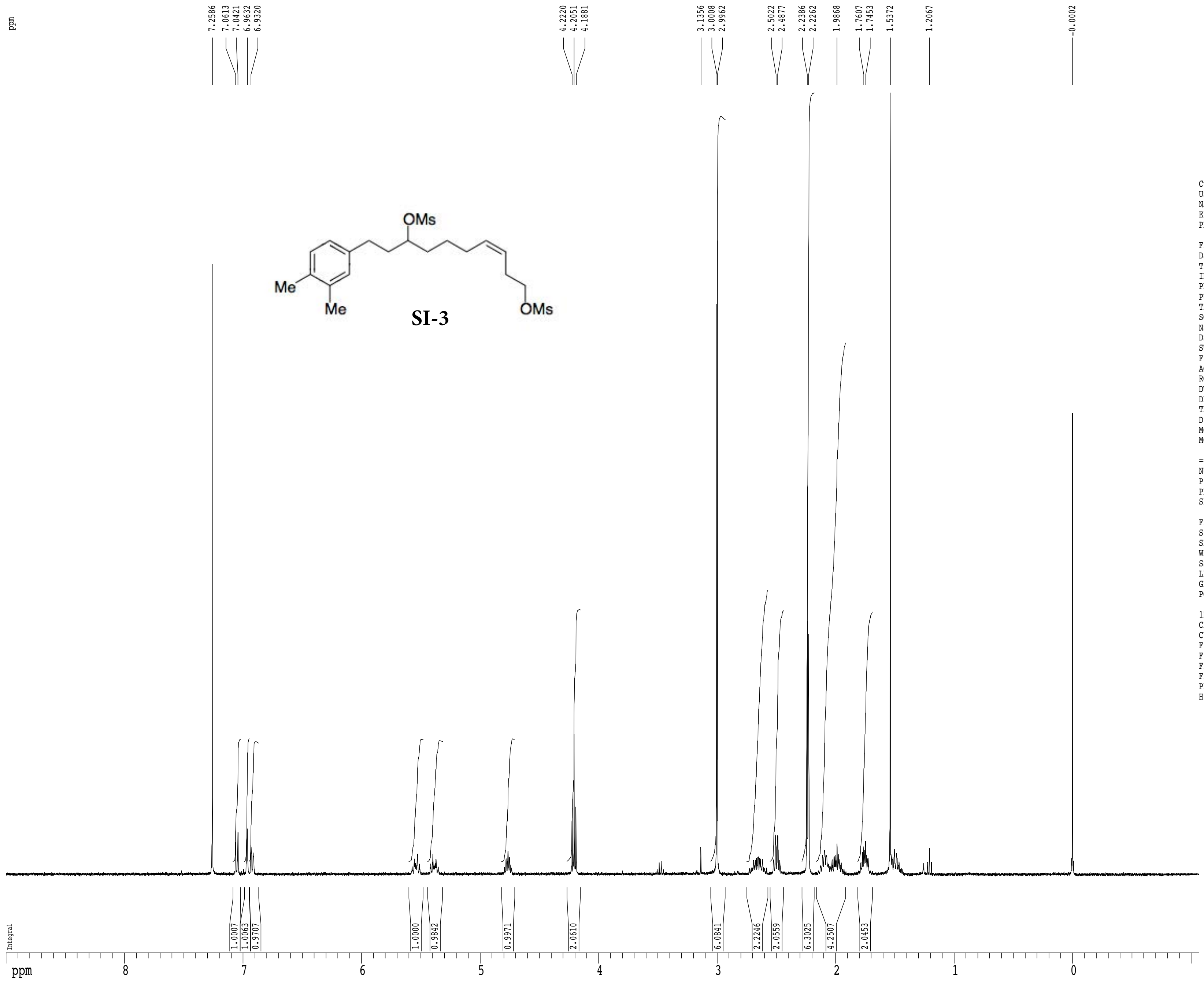


```
Current Data Parameters
USER            caherber
NAME          CAH-II-028
EXPNO                  1
PROCNO                 1

F2 - Acquisition Parameters
Date_           20211110
Time                8.57
INSTRUM           drx400
PROBHD   5 mm QNP H/F/P
PULPROG             zg30
TD                 65536
SOLVENT            CDCl3
NS                     8
DS                     2
SWH             6410.256 Hz
FIDRES          0.097813 Hz
AQ             5.1118579 sec
RG                 812.7
DW                78.000 usec
DE                  4.50 usec
TE                 298.0 K
D1            0.10000000 sec
MCREST        0.00000000 sec
MCWRK         0.01500000 sec

======== CHANNEL f1 ========
NUC1                  1H
P1                 12.00 usec
PL1                -0.90 dB
SFO1         400.1328009 MHz

F2 - Processing parameters
SI                 65536
SF           400.1300220 MHz
WDW                   no
SSB                    0
LB                  0.00 Hz
GB                     0
PC                  2.00

1D NMR plot parameters
CX                 22.80 cm
CY                 15.00 cm
F1P                9.000 ppm
F1               3601.17 Hz
F2P               -1.065 ppm
F2               -426.17 Hz
PPMCM            0.44145 ppm/cm
HZCM           176.63780 Hz/cm
```

13C spectrum with 1H decoupling

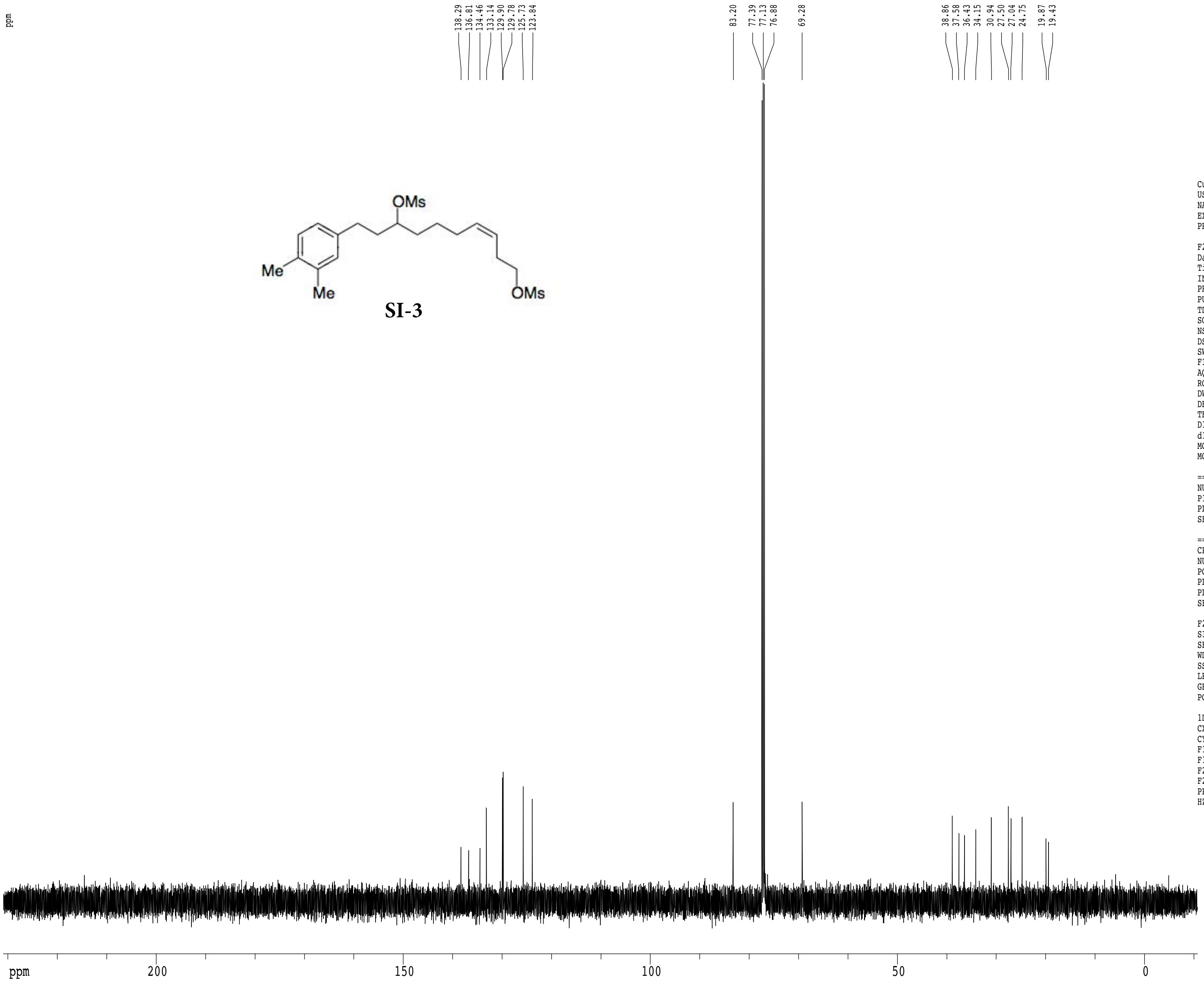


```
Current Data Parameters
USER          caherber
NAME     CAH-II-029-13C
EXPNO                2
PROCNO               1

F2 - Acquisition Parameters
Date_          20220114
Time              13.50
INSTRUM           gn500
PROBHD   5 mm broadband
PULPROG          zgdc30
TD                65536
SOLVENT           CDCl3
NS                  608
DS                    4
SWH           30303.031 Hz
FIDRES         0.462388 Hz
AQ            1.0813940 sec
RG                46341
DW               16.500 usec
DE                 6.00 usec
TE                293.1 K
D1           0.25000000 sec
d11          0.03000000 sec
MCREST       0.00000000 sec
MCWRK        0.01500000 sec

======== CHANNEL f1 ========
NUC1                13C
P1                14.20 usec
PL1               -6.00 dB
SFO1        125.3994349 MHz

======== CHANNEL f2 ========
CPDPRG2         waltz16
NUC2                 1H
PCPD2            100.00 usec
PL2               -6.00 dB
PL12              12.30 dB
SFO2        498.6524933 MHz

F2 - Processing parameters
SI                65536
SF          125.3856343 MHz
WDW                  no
SSB                   0
LB                 0.00 Hz
GB                    0
PC                 2.00

1D NMR plot parameters
CX                22.80 cm
CY                15.65 cm
F1P             230.956 ppm
F1             28958.52 Hz
F2P             -10.723 ppm
F2             -1344.51 Hz
PPMCM          10.59994 ppm/cm
HZCM         1329.08032 Hz/cm
```

1H spectrum

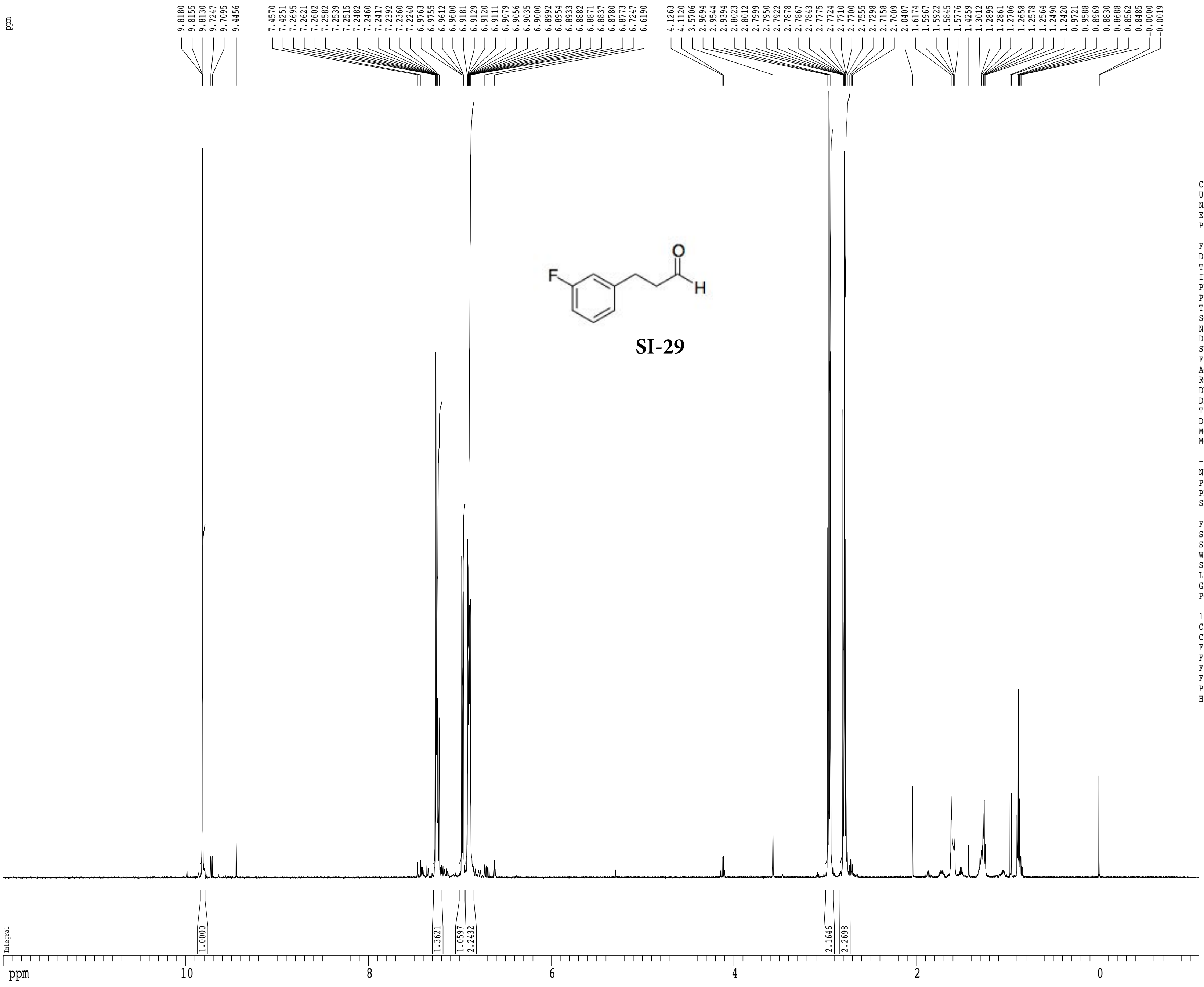

SI-29

Current Data Parameters
USER caherber
NAME CAH-I-250
EXPNO 1
PROCNO 1

F2 - Acquisition Parameters
Date_ 20210720
Time 15.44
INSTRUM gn500
PROBHD 5 mm broadband
PULPROG zg30
TD 81728
SOLVENT CDCl3
NS 8
DS 2
SWH 8012.820 Hz
FIDRES 0.098043 Hz
AQ 5.0998774 sec
RG 574.7
DW 62.400 usec
DE 6.00 usec
TE 297.9 K
D1 0.10000000 sec
MCREST 0.00000000 sec
MCWRK 0.01500000 sec

======== CHANNEL f1 ========
NUC1 1H
P1 12.00 usec
PL1 -6.00 dB
SFO1 498.6534906 MHz

F2 - Processing parameters
SI 65536
SF 498.6500290 MHz
WDW no
SSB 0
LB 0.00 Hz
GB 0
PC 1.00

1D NMR plot parameters
CX 22.80 cm
CY 15.00 cm
F1P 12.000 ppm
F1 5983.80 Hz
F2P -1.093 ppm
F2 -544.82 Hz
PPMCM 0.57424 ppm/cm
HZCM 286.34293 Hz/cm

1H spectrum

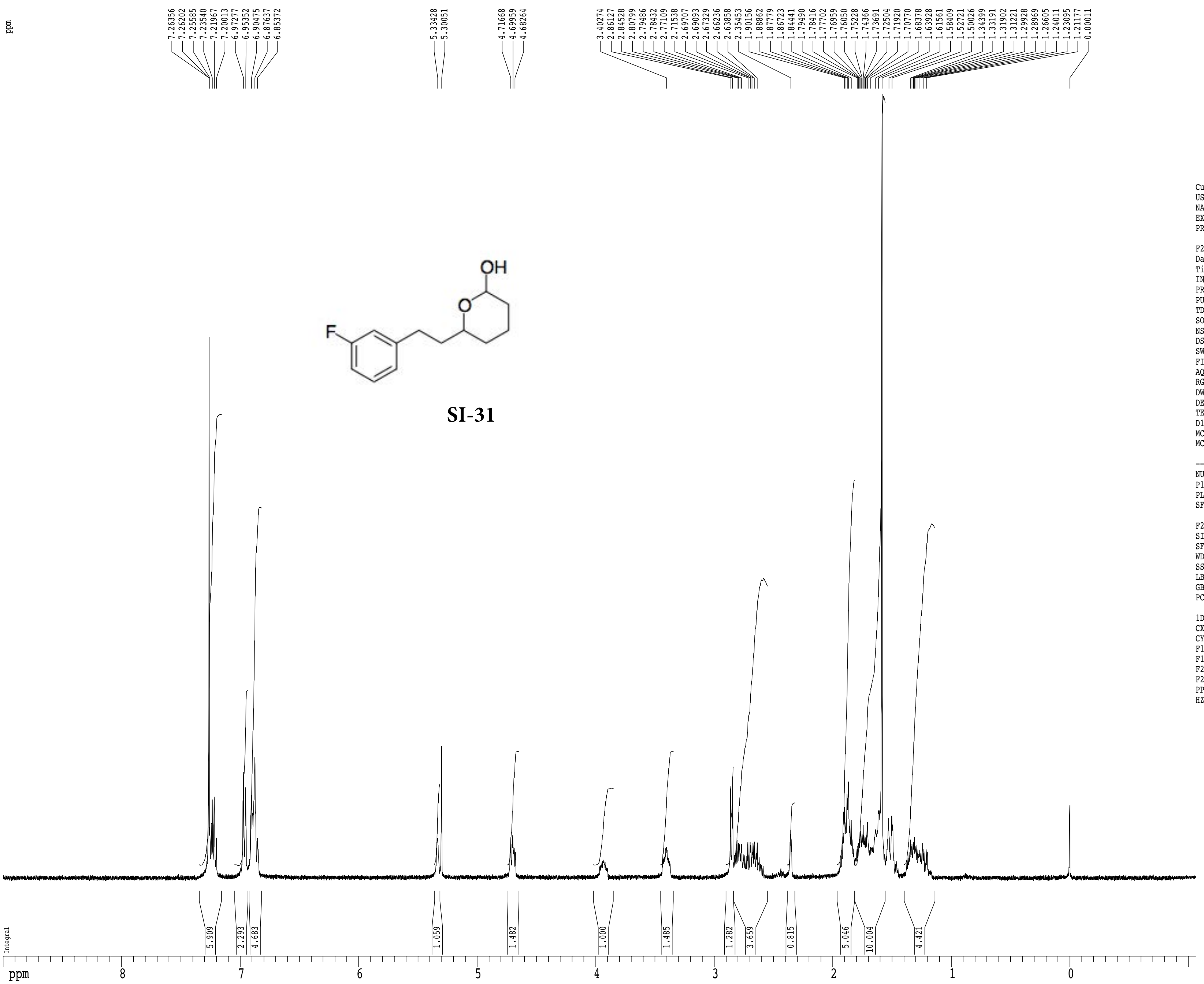

SI-31

```
Current Data Parameters
USER          caherber
NAME          CAH-I-254
EXPNO                1
PROCNO               1

F2 - Acquisition Parameters
Date_         20210726
Time             15.04
INSTRUM         drx400
PROBHD   5 mm QNP H/F/P
PULPROG           zg30
TD               65536
SOLVENT          CDCl3
NS                   8
DS                   2
SWH           6410.256 Hz
FIDRES        0.097813 Hz
AQ           5.1118579 sec
RG               724.1
DW              78.000 usec
DE                4.50 usec
TE               293.7 K
D1          0.10000000 sec
MCREST      0.00000000 sec
MCWRK       0.01500000 sec

======== CHANNEL f1 ========
NUC1                1H
P1               12.00 usec
PL1              -1.60 dB
SFO1       400.1328009 MHz

F2 - Processing parameters
SI               65536
SF         400.1300202 MHz
WDW                 no
SSB                  0
LB                0.00 Hz
GB                   0
PC                2.00

1D NMR plot parameters
CX               22.80 cm
CY               15.00 cm
F1P              9.000 ppm
F1             3601.17 Hz
F2P             -1.061 ppm
F2             -424.41 Hz
PPMCM          0.44126 ppm/cm
HZCM         176.56059 Hz/cm
```

13C Spectrum

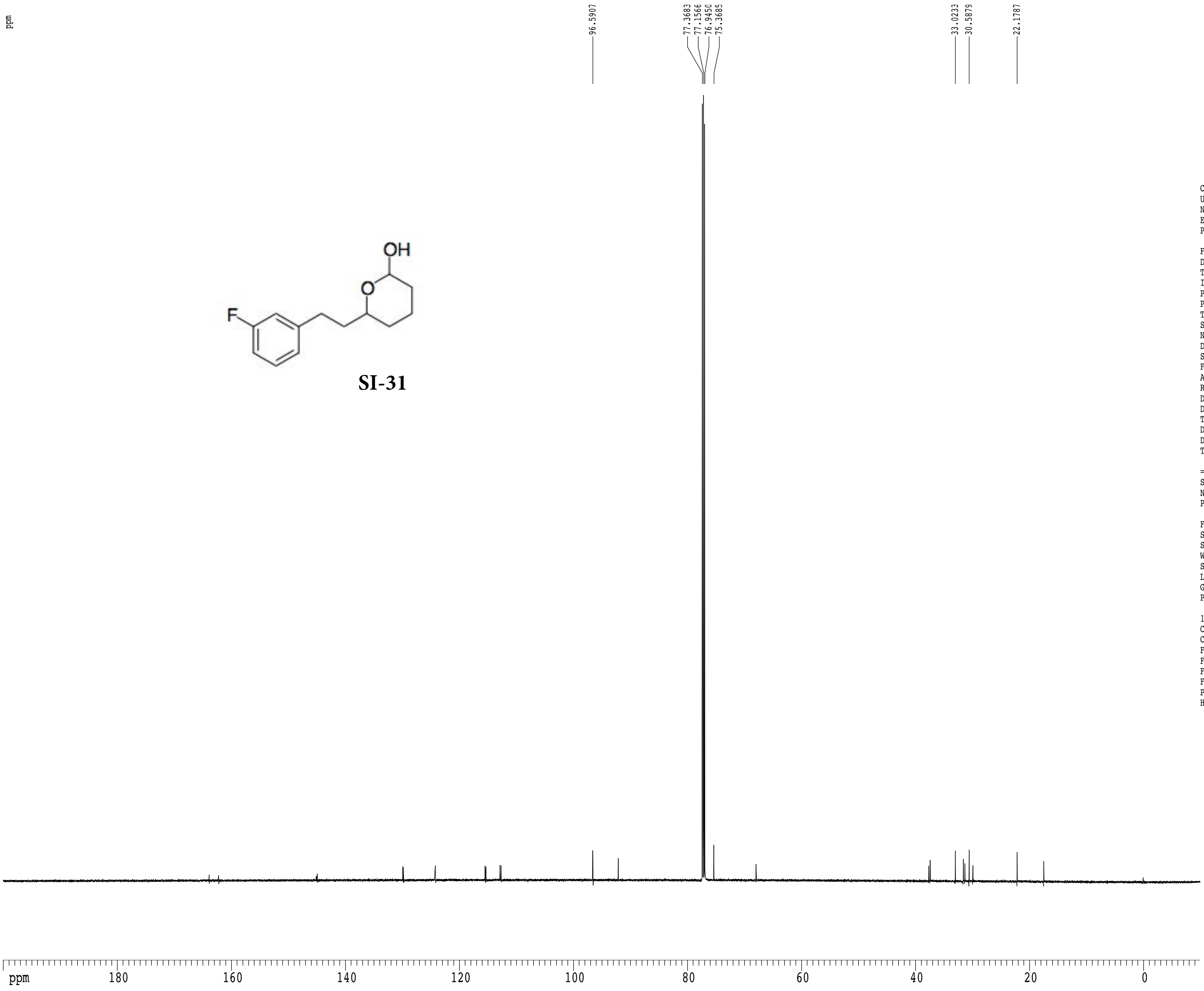

SI-31

```
Current Data Parameters
USER            caherber
NAME     CAH-I-254-full
EXPNO                  3
PROCNO                 1

F2 - Acquisition Parameters
Date_           20220421
Time                9.54
INSTRUM            av600
PROBHD   5 mm CPBBO BB-
PULPROG           zgdc30
TD                 65536
SOLVENT            CDCl3
NS                   912
DS                     4
SWH            36231.883 Hz
FIDRES          0.552855 Hz
AQ             0.9044468 sec
RG                  2050
DW                13.800 usec
DE                 19.63 usec
TE                 298.0 K
D1            0.40000001 sec
D11           0.03000000 sec
TD0                    1

======== CHANNEL f1 ========
SFO1         150.9194080 MHz
NUC1                 13C
P1                 10.10 usec

F2 - Processing parameters
SI                 65536
SF           150.9027936 MHz
WDW                   no
SSB                    0
LB                  0.00 Hz
GB                     0
PC                  1.00

1D NMR plot parameters
CX                 22.80 cm
CY                 15.00 cm
F1P              200.000 ppm
F1              30180.56 Hz
F2P               -9.950 ppm
F2              -1501.56 Hz
PPMCM            9.20835 ppm/cm
HZCM          1389.56641 Hz/cm
```

19F spectrum

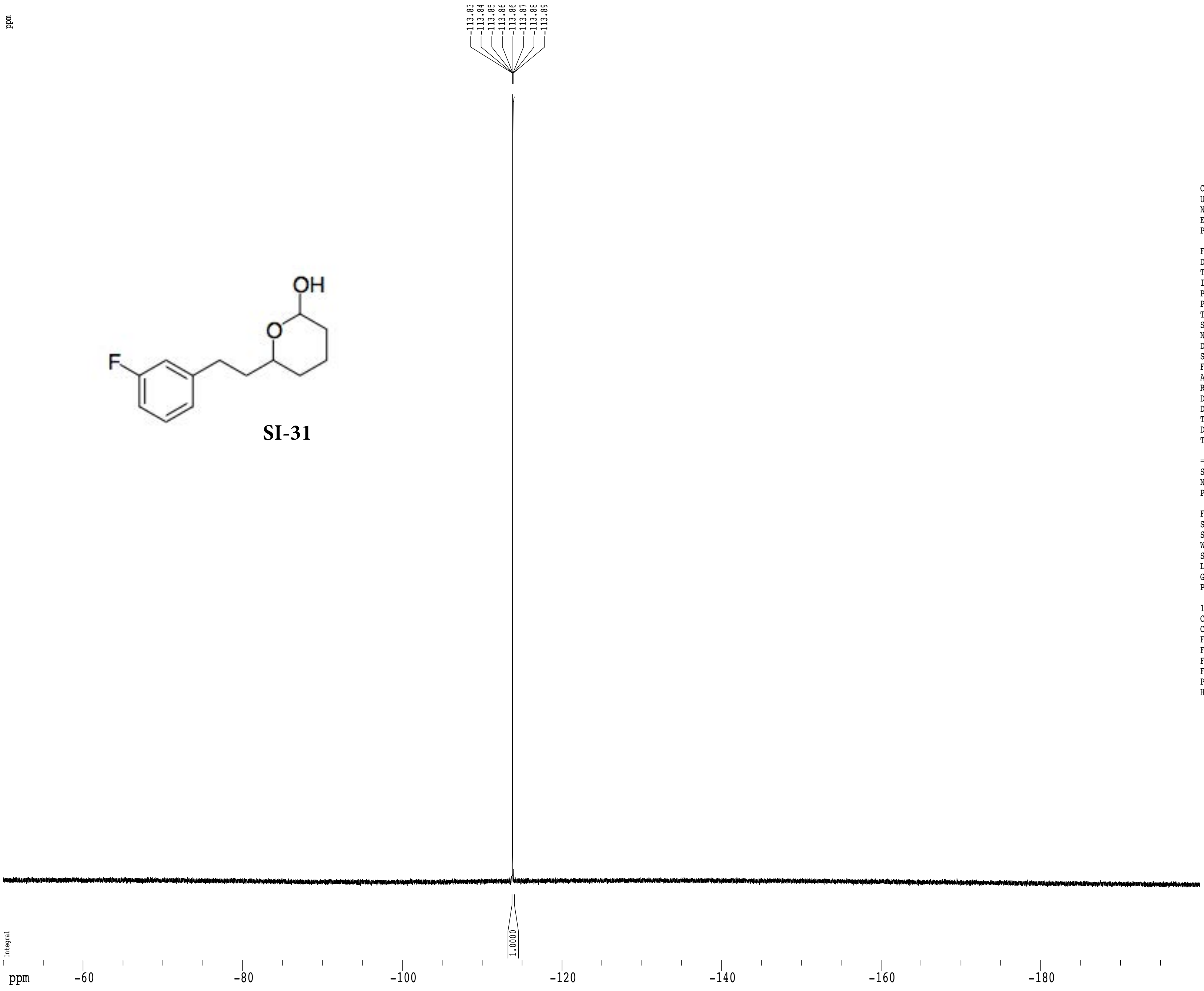

SI-31

Current Data Parameters
USER caherber
NAME CAH-I-254-full
EXPNO 2
PROCNO 1

F2 - Acquisition Parameters
Date_ 20220421
Time 9.41
INSTRUM av600
PROBHD 5 mm CPBBO BB-
PULPROG zgflqn30
TD 131072
SOLVENT CDCl3
NS 16
DS 2
SWH 178571.422 Hz
FIDRES 1.362392 Hz
AQ 0.3670516 sec
RG 575
DW 2.800 usec
DE 18.00 usec
TE 297.9 K
D1 3.00000000 sec
TD0 1

======== CHANNEL f1 ========
SFO1 564.6299196 MHz
NUC1 19F
P1 18.25 usec

F2 - Processing parameters
SI 131072
SF 564.6863882 MHz
WDW no
SSB 0
LB 0.00 Hz
GB 0
PC 1.00

1D NMR plot parameters
CX 22.80 cm
CY 15.00 cm
F1P -50.000 ppm
F1 -28234.32 Hz
F2P -200.000 ppm
F2 -112937.28 Hz
PPMCM 6.57895 ppm/cm
HZCM 3715.04224 Hz/cm

1H spectrum

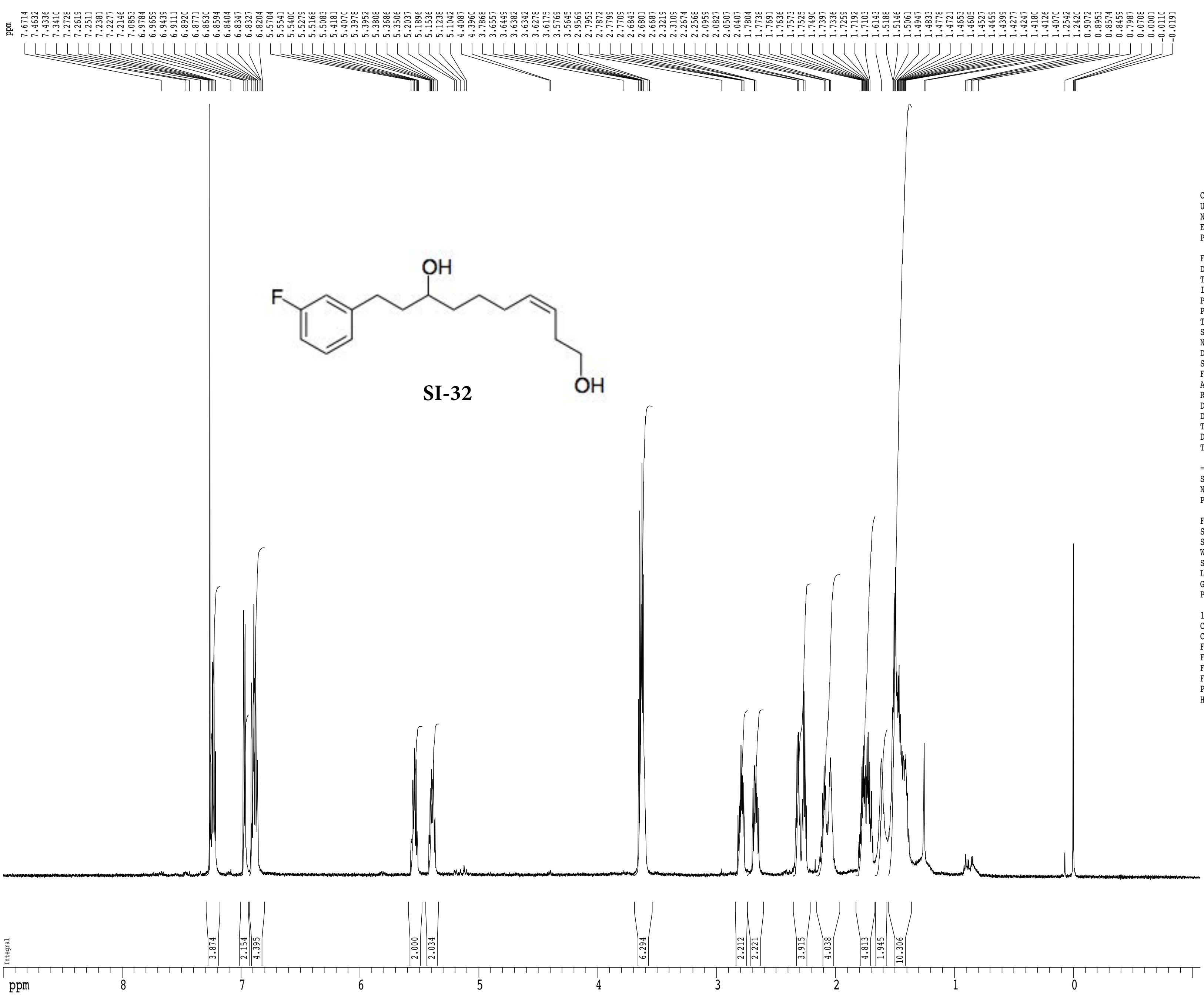

SI-32

Current Data Parameters
USER caherber
NAME CAH-I-255-full
EXPNO 1
PROCNO 1

F2 - Acquisition Parameters
Date_ 20220411
Time 13.38
INSTRUM av600
PROBHD 5 mm CPBBO BB-
PULPROG zg30
TD 98074
SOLVENT CDCl3
NS 8
DS 2
SWH 9615.385 Hz
FIDRES 0.098042 Hz
AQ 5.0998979 sec
RG 10
DW 52.000 usec
DE 14.23 usec
TE 298.0 K
D1 0.10000000 sec
TD0 1

======== CHANNEL f1 ========
SFO1 600.1342009 MHz
NUC1 1H
P1 9.50 usec

F2 - Processing parameters
SI 65536
SF 600.1300335 MHz
WDW no
SSB 0
LB 0.00 Hz
GB 0
PC 1.00

1D NMR plot parameters
CX 22.80 cm
CY 15.00 cm
F1P 9.000 ppm
F1 5401.17 Hz
F2P -1.067 ppm
F2 -640.28 Hz
PPMCM 0.44153 ppm/cm
HZCM 264.97598 Hz/cm

13C spectrum

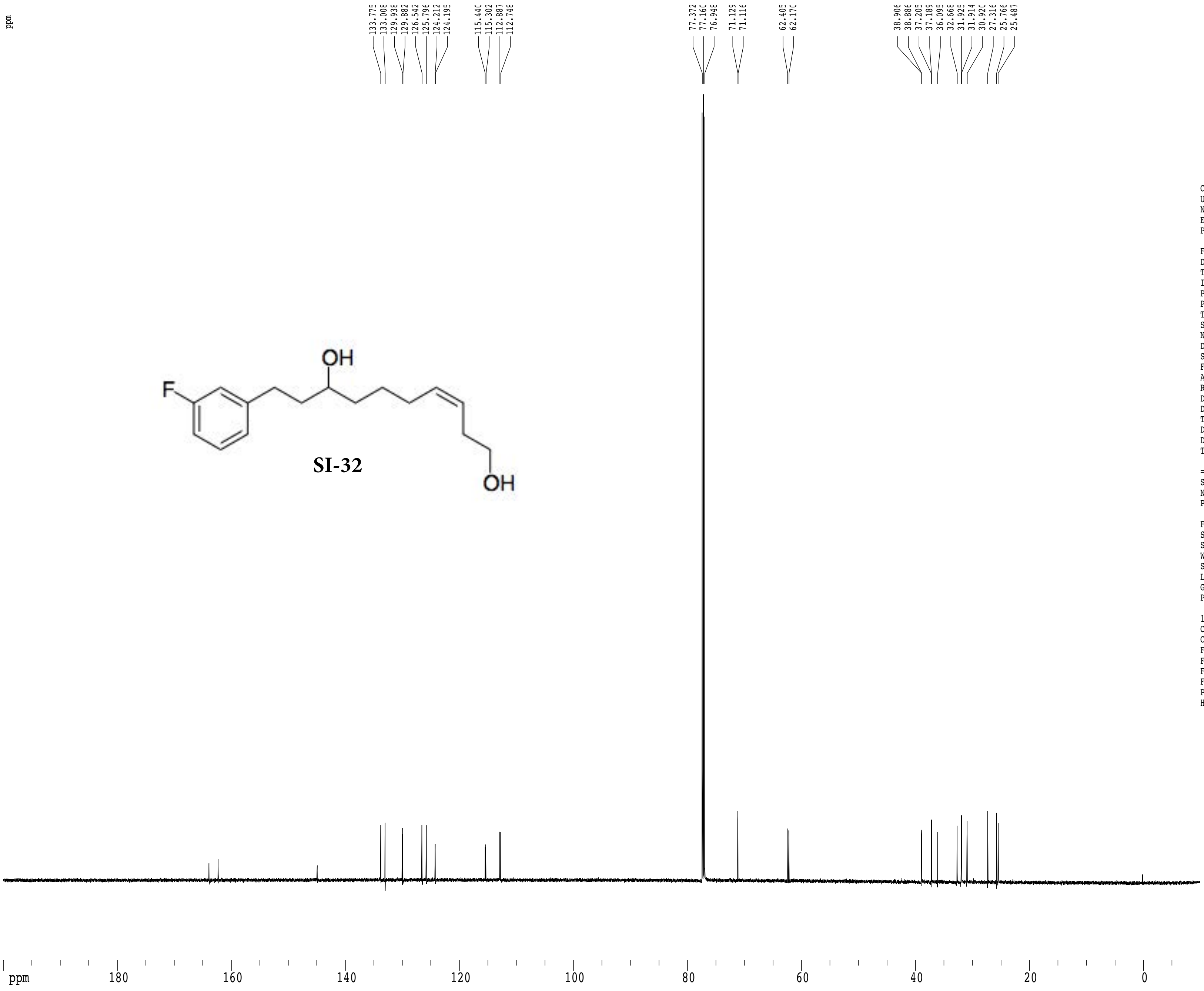

SI-32

```
Current Data Parameters
USER          caherber
NAME     CAH-I-255-full
EXPNO                 3
PROCNO                1

F2 - Acquisition Parameters
Date_          20220411
Time              13.56
INSTRUM           av600
PROBHD   5 mm CPBBO BB-
PULPROG          zgdc30
TD                65536
SOLVENT           CDCl3
NS                  410
DS                    4
SWH          36231.883 Hz
FIDRES        0.552855 Hz
AQ           0.9044468 sec
RG                 2050
DW               13.800 usec
DE                19.63 usec
TE                298.0 K
D1           0.40000001 sec
D11          0.03000000 sec
TD0                   1

======== CHANNEL f1 ========
SFO1       150.9194080 MHz
NUC1                13C
P1                10.10 usec

F2 - Processing parameters
SI                65536
SF         150.9027942 MHz
WDW                  no
SSB                   0
LB                 0.00 Hz
GB                    0
PC                 1.00

1D NMR plot parameters
CX                22.80 cm
CY                15.00 cm
F1P             200.000 ppm
F1             30180.56 Hz
F2P              -9.954 ppm
F2             -1502.12 Hz
PPMCM           9.20852 ppm/cm
HZCM         1389.59119 Hz/cm
```

19F spectrum

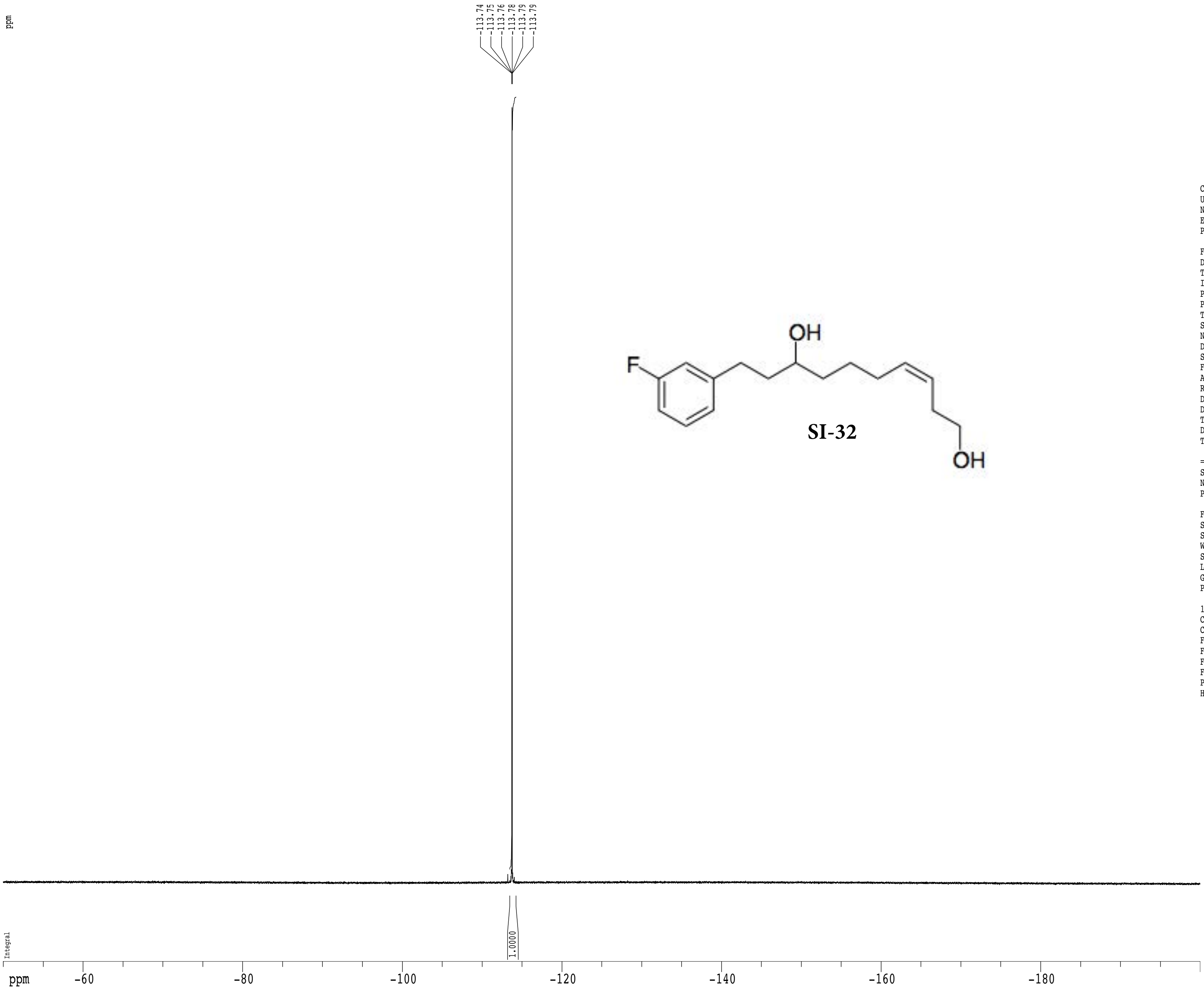

SI-32

```
Current Data Parameters
USER            caherber
NAME      CAH-I-255-full
EXPNO                  2
PROCNO                 1

F2 - Acquisition Parameters
Date_           20220411
Time               13.44
INSTRUM            av600
PROBHD   5 mm CPBBO BB-
PULPROG         zgflqn30
TD                131072
SOLVENT            CDCl3
NS                    16
DS                     2
SWH           178571.422 Hz
FIDRES          1.362392 Hz
AQ             0.3670516 sec
RG                   575
DW                 2.800 usec
DE                 18.00 usec
TE                 298.0 K
D1            3.00000000 sec
TD0                    1

======== CHANNEL f1 ========
SFO1         564.6299196 MHz
NUC1                 19F
P1                 18.25 usec

F2 - Processing parameters
SI                131072
SF           564.6863882 MHz
WDW                   no
SSB                    0
LB                  0.00 Hz
GB                     0
PC                  1.00

1D NMR plot parameters
CX                 22.80 cm
CY                 15.00 cm
F1P              -50.000 ppm
F1             -28234.32 Hz
F2P             -200.000 ppm
F2            -112937.28 Hz
PPMCM            6.57895 ppm/cm
HZCM          3715.04224 Hz/cm
```

h1.c

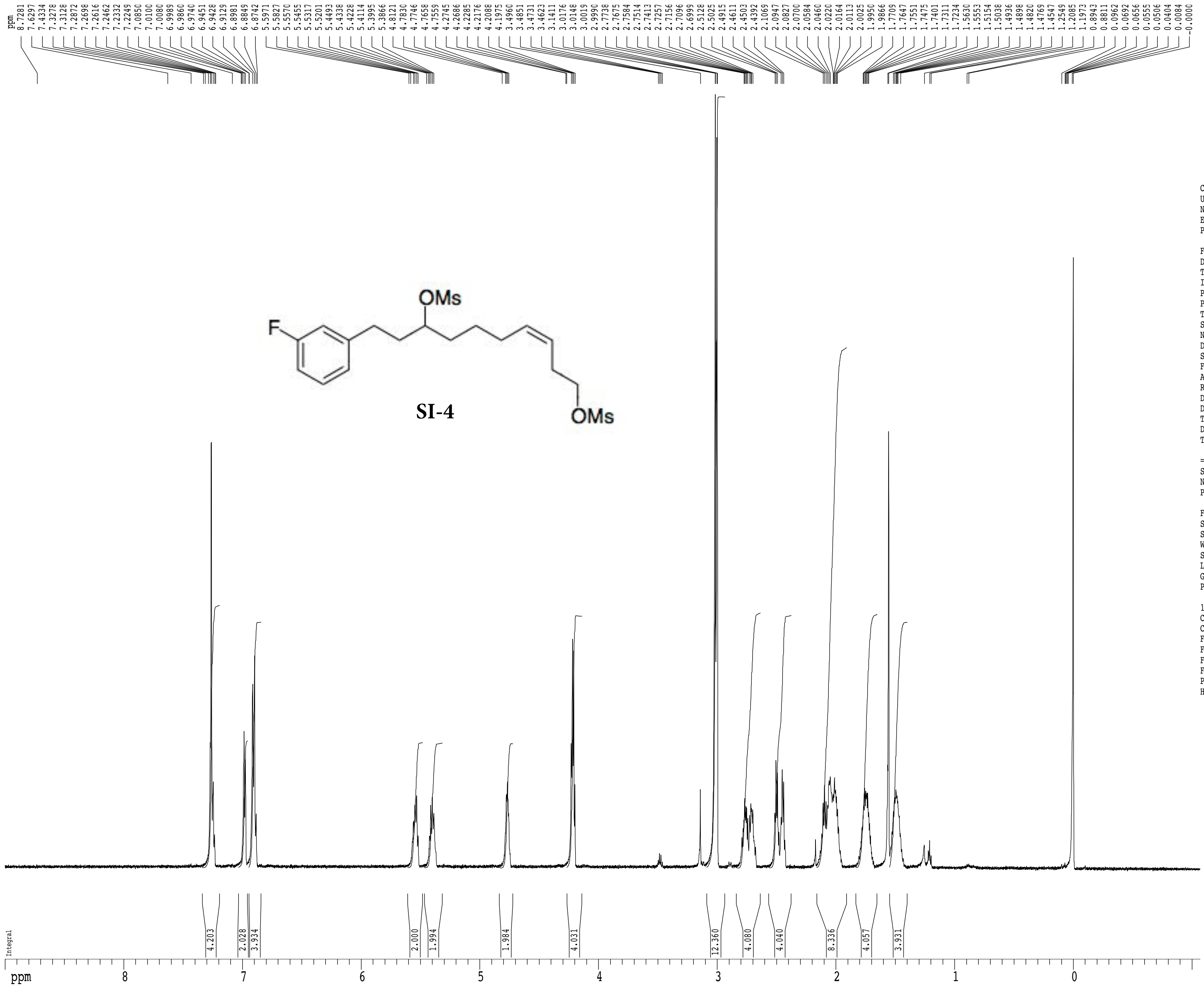

SI-4

Current Data Parameters
USER caherber
NAME CAH-I-257-full
EXPNO 1
PROCNO 1

F2 - Acquisition Parameters
Date_ 20210803
Time 8.35
INSTRUM av600
PROBHD 5 mm CPBBO BB-
PULPROG zg30
TD 98074
SOLVENT CDCl3
NS 8
DS 2
SWH 9615.385 Hz
FIDRES 0.098042 Hz
AQ 5.0998979 sec
RG 10
DW 52.000 usec
DE 14.23 usec
TE 298.0 K
D1 0.10000000 sec
TD0 1

======== CHANNEL f1 ========
SFO1 600.1342009 MHz
NUC1 1H
P1 9.50 usec

F2 - Processing parameters
SI 65536
SF 600.1300345 MHz
WDW no
SSB 0
LB 0.00 Hz
GB 0
PC 1.00

1D NMR plot parameters
CX 22.80 cm
CY 15.00 cm
F1P 9.000 ppm
F1 5401.17 Hz
F2P -1.069 ppm
F2 -641.31 Hz
PPMCM 0.44161 ppm/cm
HZCM 265.02106 Hz/cm

Z-restored spin-echo 13C spectrum with 1H decoupling

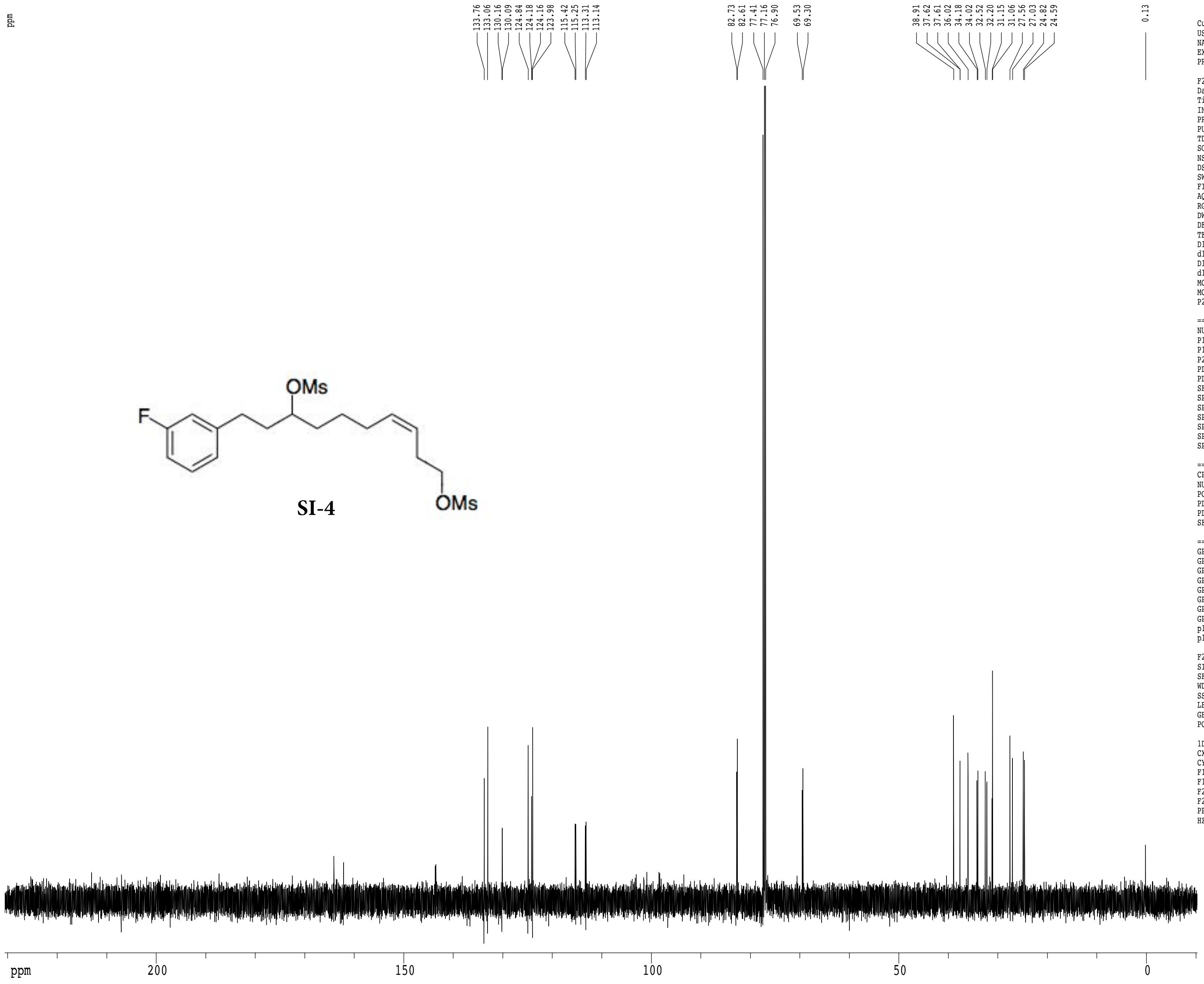

SI-4

```
Current Data Parameters
USER            caherber
NAME       CAH-I-257-C13
EXPNO                  1
PROCNO                 1

F2 - Acquisition Parameters
Date_           20210804
Time               12.10
INSTRUM          cryo500
PROBHD   5 mm CPTCI 1H-
PULPROG  SpinEchopg30gp2.prd
TD                 65536
SOLVENT            CDCl3
NS                   200
DS                    16
SWH            30303.031 Hz
FIDRES          0.462388 Hz
AQ             1.0813940 sec
RG                7298.2
DW                16.500 usec
DE                  6.00 usec
TE                 298.0 K
D1            0.25000000 sec
d11           0.03000000 sec
D16           0.00020000 sec
d17           0.00019600 sec
MCREST        0.00000000 sec
MCWRK         0.01500000 sec
P2                 37.70 usec

======== CHANNEL f1 ========
NUC1                 13C
P1                 18.85 usec
P12              2000.00 usec
P20               500.00 usec
PL0               120.00 dB
PL1                -1.00 dB
SFO1         125.7942548 MHz
SP2                 1.55 dB
SP4                 1.55 dB
SPNAM2       Crp60comp.4
SPNAM4    Crp60,0.5,20.1
SPOFF2              0.00 Hz
SPOFF4              0.00 Hz

======== CHANNEL f2 ========
CPDPRG2          waltz16
NUC2                  1H
PCPD2             100.00 usec
PL2                 1.60 dB
PL12               22.00 dB
SFO2         500.2225011 MHz

====== GRADIENT CHANNEL =====
GPNAM1          SINE.100
GPNAM2          SINE.100
GPX1                0.00 %
GPX2                0.00 %
GPY1                0.00 %
GPY2                0.00 %
GPZ1               30.00 %
GPZ2               50.00 %
p15               500.00 usec
p16              1000.00 usec

F2 - Processing parameters
SI                 65536
SF           125.7804071 MHz
WDW                   no
SSB                    0
LB                  0.00 Hz
GB                     0
PC                  2.00

1D NMR plot parameters
CX                 22.80 cm
CY                 15.65 cm
F1P              230.637 ppm
F1              29009.68 Hz
F2P              -10.287 ppm
F2              -1293.96 Hz
PPMCM           10.56688 ppm/cm
HZCM          1329.10693 Hz/cm
```

f19.c

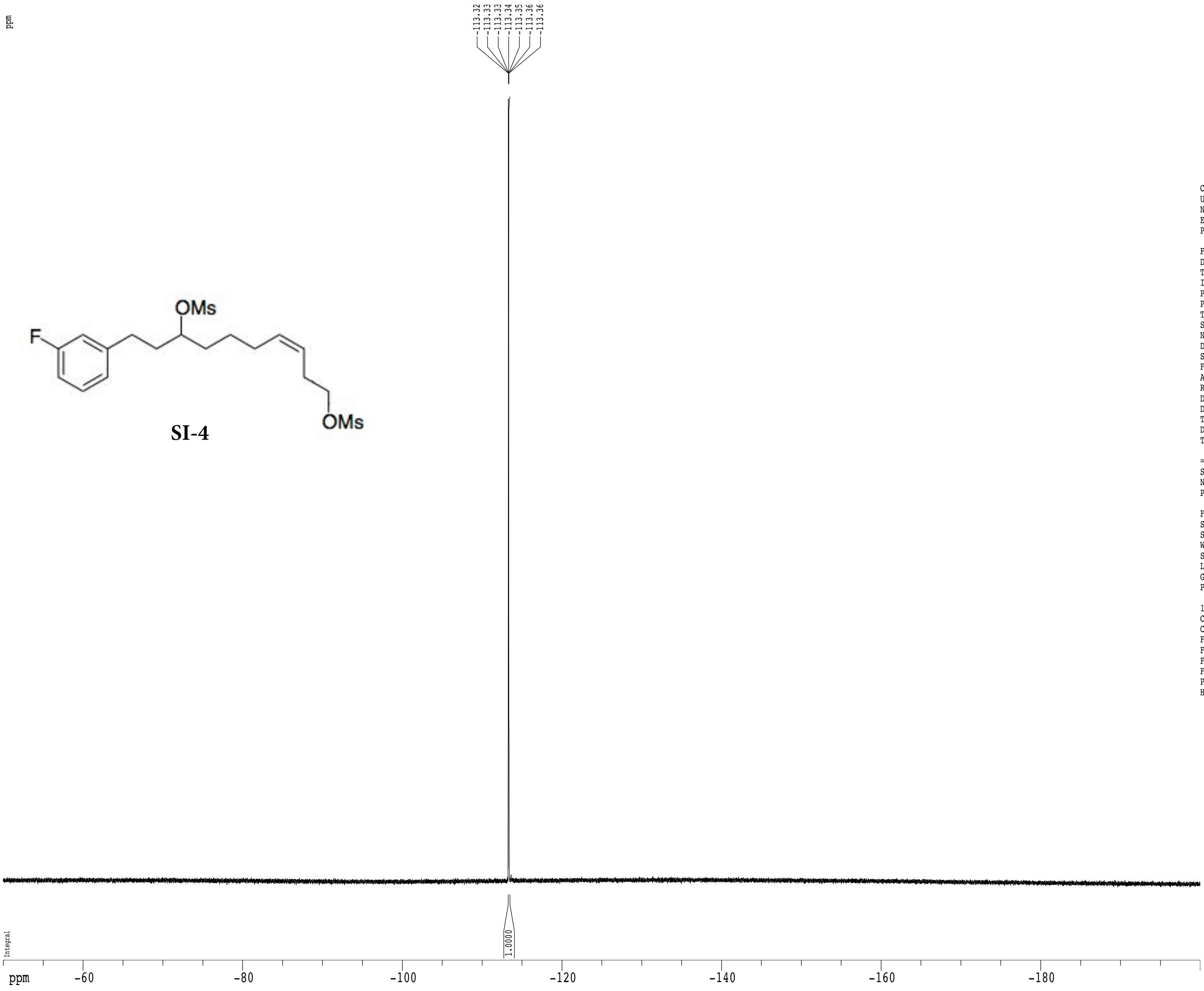

SI-4

```
Current Data Parameters
USER          caherber
NAME     CAH-I-257-full
EXPNO                3
PROCNO               1

F2 - Acquisition Parameters
Date_          20210803
Time               8.59
INSTRUM           av600
PROBHD   5 mm CPBBO BB-
PULPROG        zgflqn30
TD               131072
SOLVENT           CDCl3
NS                   16
DS                    2
SWH          178571.422 Hz
FIDRES         1.362392 Hz
AQ            0.3670516 sec
RG                  575
DW                2.800 usec
DE                18.00 usec
TE                298.0 K
D1           3.00000000 sec
TD0                   1

======== CHANNEL f1 ========
SFO1        564.6299196 MHz
NUC1                19F
P1                18.25 usec

F2 - Processing parameters
SI               131072
SF          564.6863882 MHz
WDW                  no
SSB                   0
LB                 0.00 Hz
GB                    0
PC                 1.00

1D NMR plot parameters
CX                22.80 cm
CY                15.00 cm
F1P             -50.000 ppm
F1            -28234.32 Hz
F2P            -200.000 ppm
F2           -112937.28 Hz
PPMCM           6.57895 ppm/cm
HZCM         3715.04224 Hz/cm
```

1H spectrum

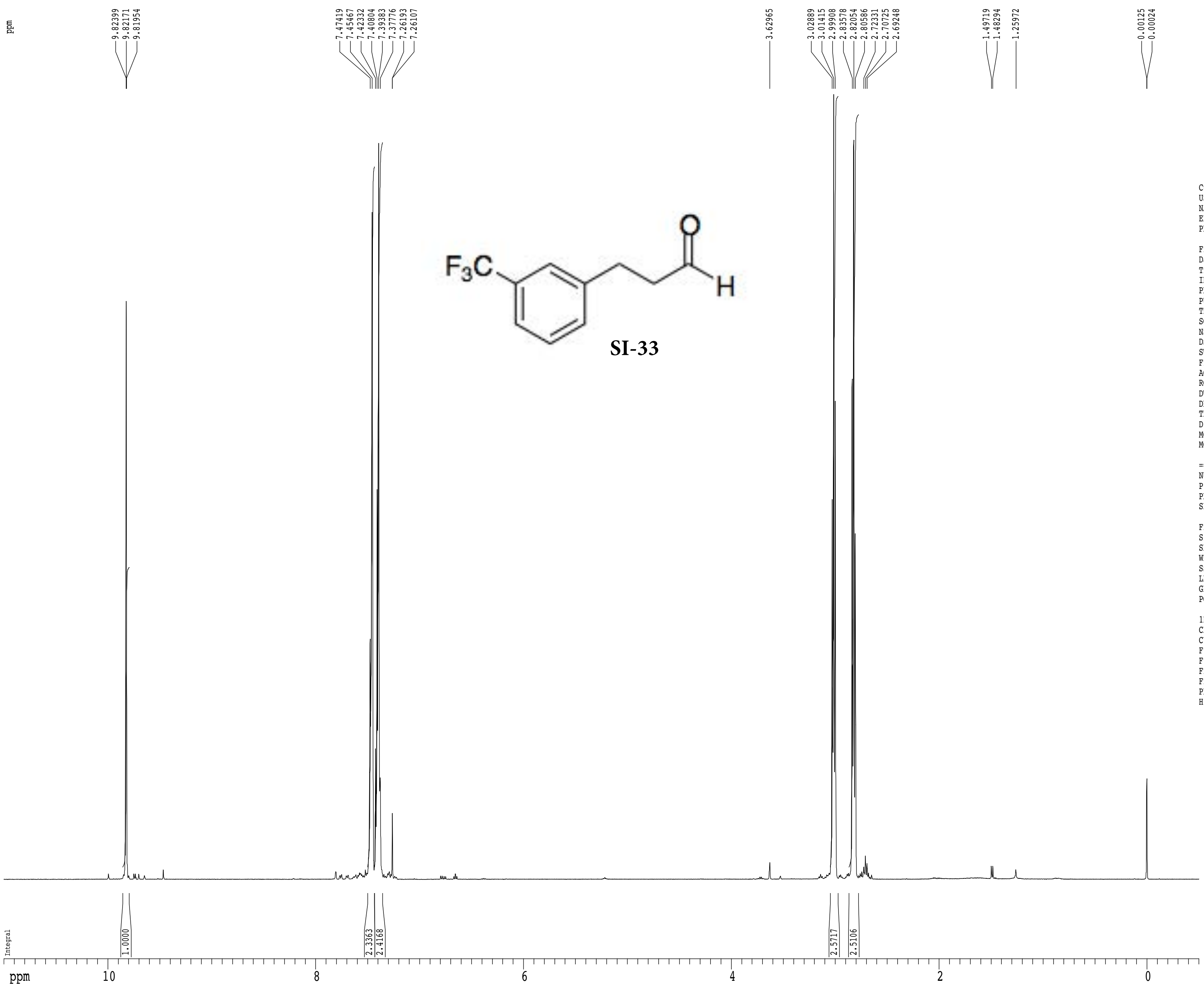

SI-33

Current Data Parameters
USER khewitt1
NAME KAH-IV-289-Z
EXPNO 1
PROCNO 1

F2 - Acquisition Parameters
Date_ 20210621
Time 14.12
INSTRUM cryo500
PROBHD 5 mm CPTCI 1H-
PULPROG zg30
TD 48074
SOLVENT CDCl3T
NS 8
DS 2
SWH 8012.820 Hz
FIDRES 0.166677 Hz
AQ 2.9998677 sec
RG 6.3
DW 62.400 usec
DE 6.00 usec
TE 298.0 K
D1 0.10000000 sec
MCREST 0.00000000 sec
MCWRK 0.01500000 sec

======== CHANNEL f1 ========
NUC1 1H
P1 9.75 usec
PL1 1.60 dB
SFO1 500.2235015 MHz

F2 - Processing parameters
SI 65536
SF 500.2200305 MHz
WDW no
SSB 0
LB 0.00 Hz
GB 0
PC 1.00

1D NMR plot parameters
CX 22.80 cm
CY 15.00 cm
F1P 11.000 ppm
F1 5502.42 Hz
F2P -0.500 ppm
F2 -250.11 Hz
PPMCM 0.50439 ppm/cm
HZCM 252.30397 Hz/cm

Z-restored spin-echo 13C spectrum with 1H decoupling

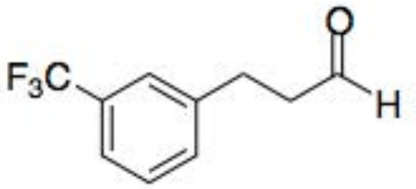

SI-33

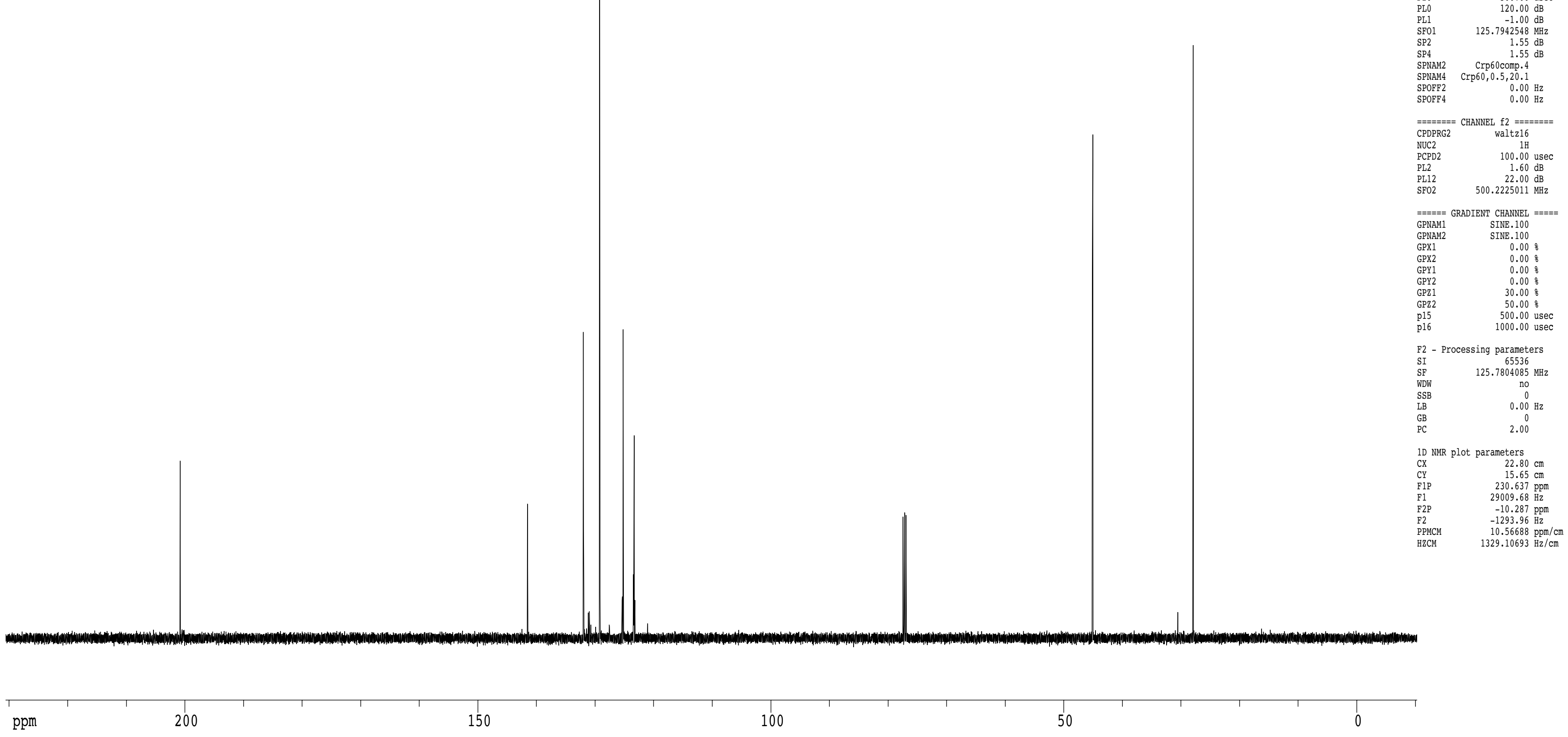

f19.c

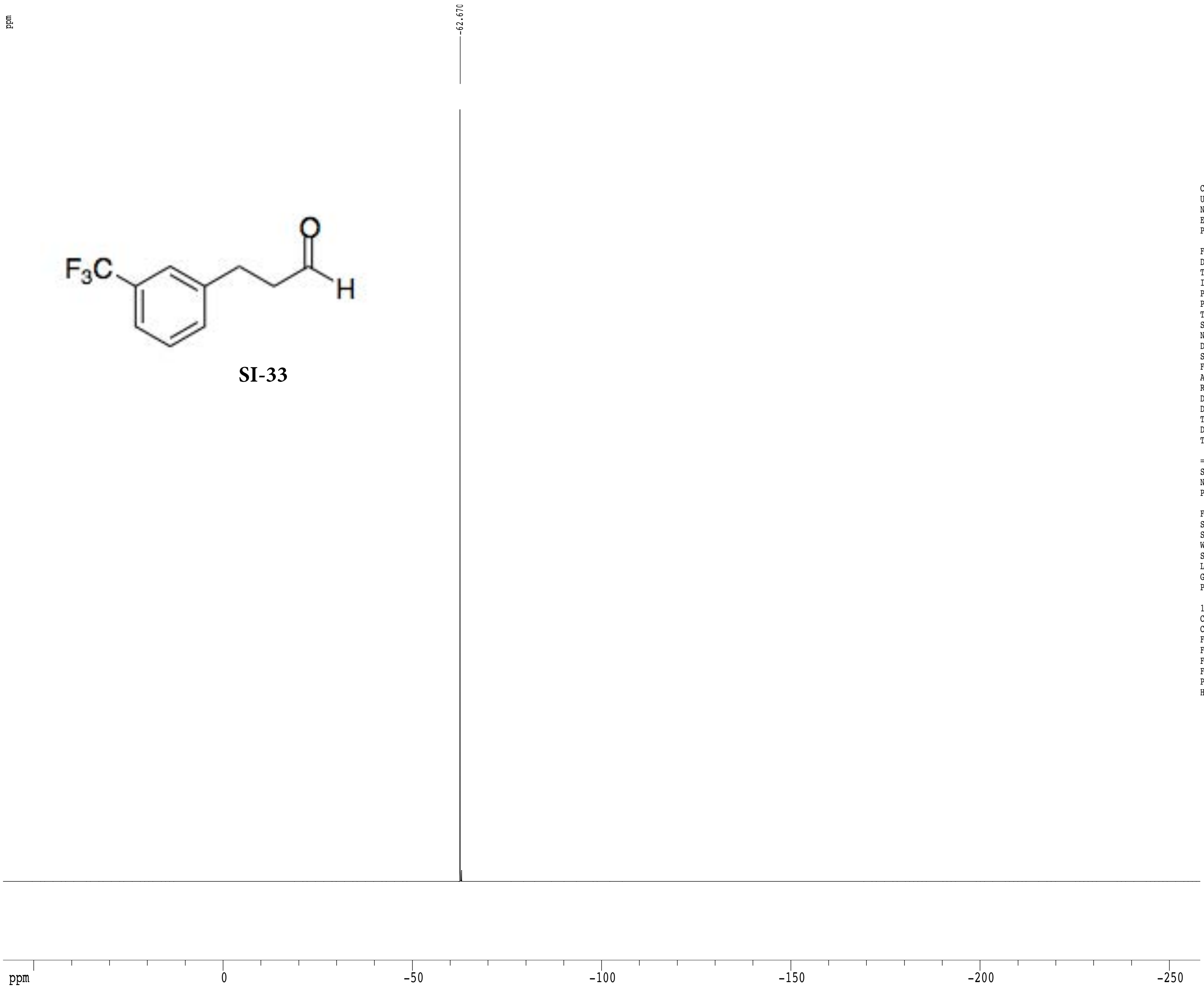

SI-33

Current Data Parameters
USER khewitt1
NAME KAH-IV-289-F
EXPNO 2
PROCNO 1

F2 - Acquisition Parameters
Date_ 20210621
Time 14.54
INSTRUM av600
PROBHD 5 mm CPBBO BB-
PULPROG zgflqn30
TD 131072
SOLVENT CDCl3T
NS 16
DS 2
SWH 178571.422 Hz
FIDRES 1.362392 Hz
AQ 0.3670516 sec
RG 362
DW 2.800 usec
DE 18.00 usec
TE 298.0 K
D1 3.00000000 sec
TD0 1

======== CHANNEL f1 ========
SFO1 564.6299196 MHz
NUC1 19F
P1 18.25 usec

F2 - Processing parameters
SI 131072
SF 564.6864196 MHz
WDW no
SSB 0
LB 0.00 Hz
GB 0
PC 1.00

1D NMR plot parameters
CX 22.80 cm
CY 15.00 cm
F1P 58.060 ppm
F1 32785.75 Hz
F2P -258.171 ppm
F2 -145785.67 Hz
PPMCM 13.86979 ppm/cm
HZCM 7832.08008 Hz/cm

h1.c

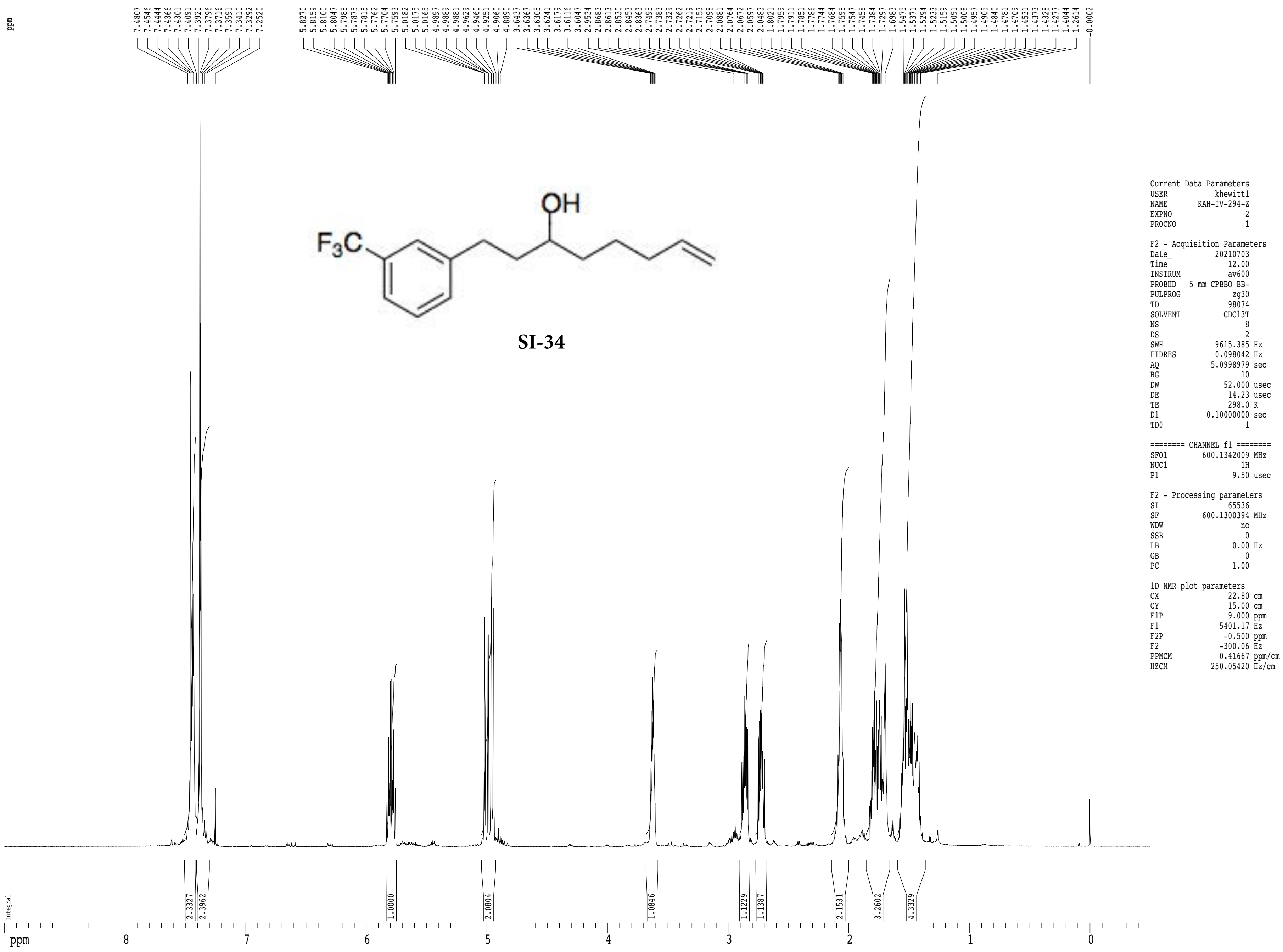

SI-34

```
Current Data Parameters
USER          khewitt1
NAME       KAH-IV-294-Z
EXPNO                 2
PROCNO                1

F2 - Acquisition Parameters
Date_          20210703
Time              12.00
INSTRUM           av600
PROBHD   5 mm CPBBO BB-
PULPROG            zg30
TD                98074
SOLVENT          CDCl3T
NS                    8
DS                    2
SWH            9615.385 Hz
FIDRES         0.098042 Hz
AQ            5.0998979 sec
RG                   10
DW               52.000 usec
DE                14.23 usec
TE                298.0 K
D1           0.10000000 sec
TD0                   1

======== CHANNEL f1 ========
SFO1        600.1342009 MHz
NUC1                 1H
P1                 9.50 usec

F2 - Processing parameters
SI                65536
SF          600.1300394 MHz
WDW                  no
SSB                   0
LB                 0.00 Hz
GB                    0
PC                 1.00

1D NMR plot parameters
CX                22.80 cm
CY                15.00 cm
F1P               9.000 ppm
F1              5401.17 Hz
F2P              -0.500 ppm
F2              -300.06 Hz
PPMCM           0.41667 ppm/cm
HZCM          250.05420 Hz/cm
```

13C.c

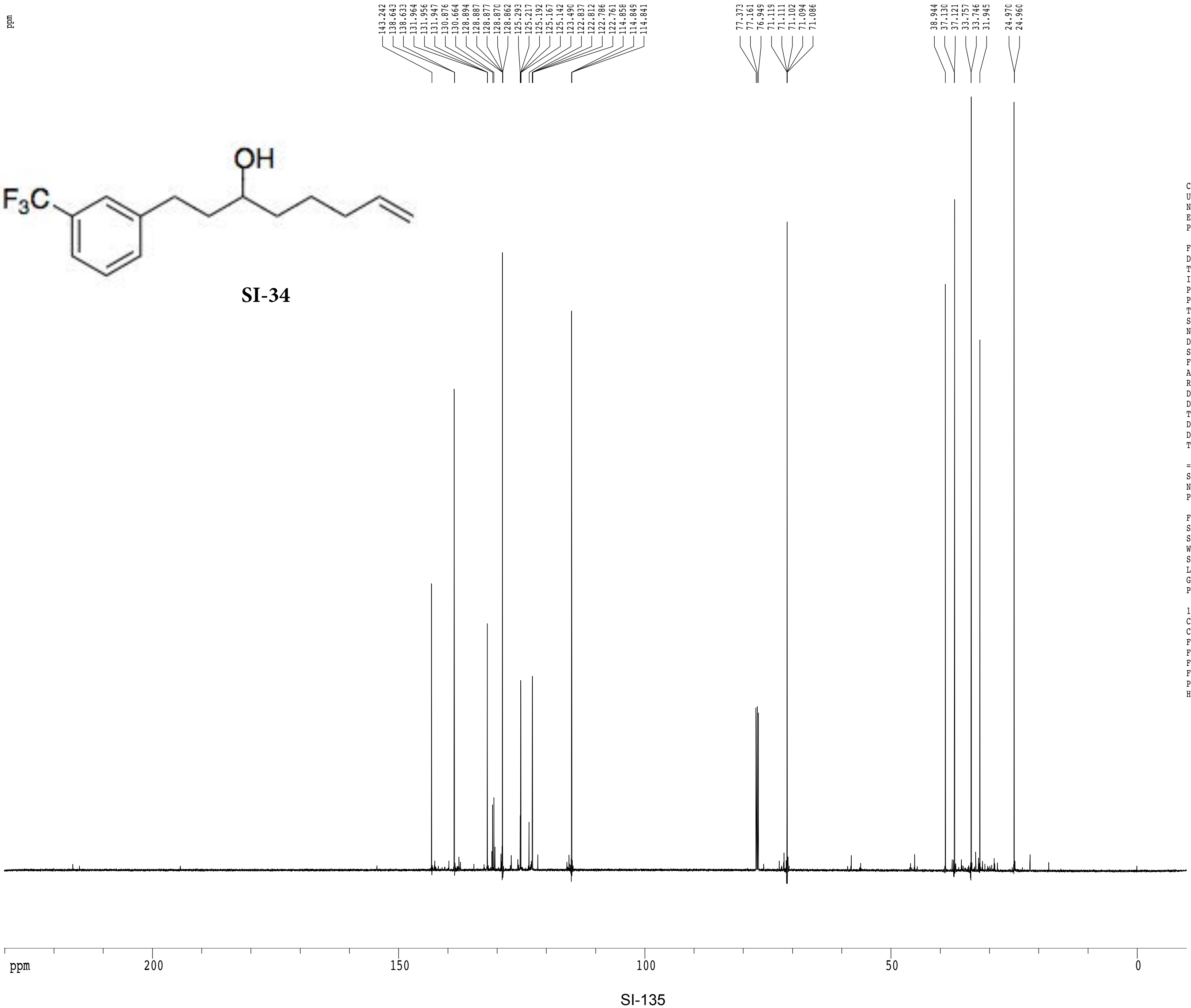

SI-34

Current Data Parameters
USER khewitt1
NAME KAH-IV-294-Z
EXPNO 1
PROCNO 1

F2 - Acquisition Parameters
Date_ 20210703
Time 11.56
INSTRUM av600
PROBHD 5 mm CPBBO BB-
PULPROG zgdc30
TD 65536
SOLVENT CDCl3T
NS 157
DS 4
SWH 36231.883 Hz
FIDRES 0.552855 Hz
AQ 0.9044468 sec
RG 2050
DW 13.800 usec
DE 19.63 usec
TE 298.0 K
D1 0.40000001 sec
D11 0.03000000 sec
TD0 1

======== CHANNEL f1 ========
SFO1 150.9194080 MHz
NUC1 13C
P1 10.10 usec

F2 - Processing parameters
SI 65536
SF 150.9027986 MHz
WDW no
SSB 0
LB 0.00 Hz
GB 0
PC 1.00

1D NMR plot parameters
CX 22.80 cm
CY 15.00 cm
F1P 230.117 ppm
F1 34725.34 Hz
F2P -9.984 ppm
F2 -1506.54 Hz
PPMCM 10.53074 ppm/cm
HZCM 1589.11780 Hz/cm

f19.c

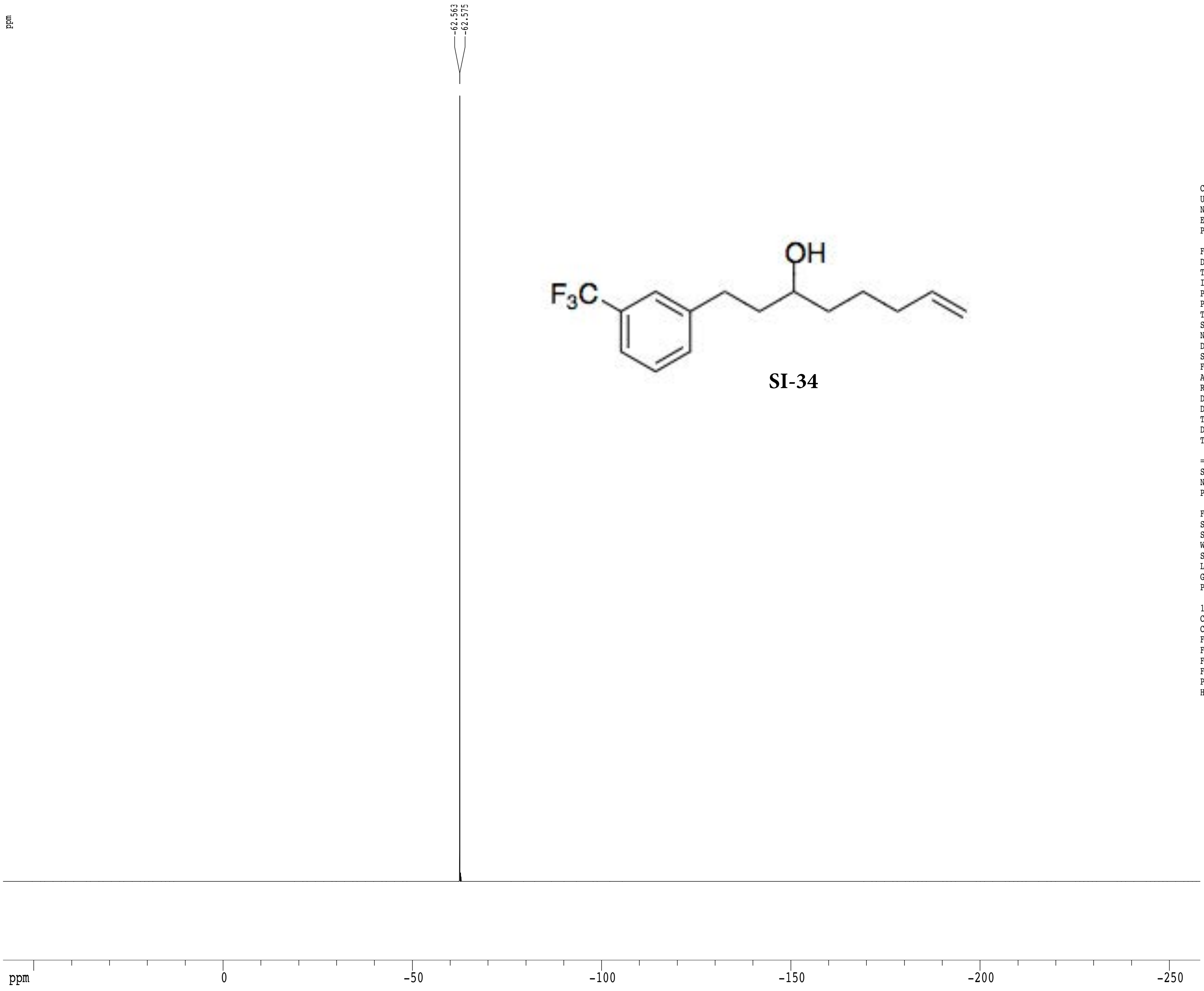

SI-34

Current Data Parameters
USER khewitt1
NAME KAH-IV-294-Z
EXPNO 3
PROCNO 1

F2 - Acquisition Parameters
Date_ 20210703
Time 12.04
INSTRUM av600
PROBHD 5 mm CPBBO BB-
PULPROG zgflqn30
TD 131072
SOLVENT CDCl3T
NS 16
DS 2
SWH 178571.422 Hz
FIDRES 1.362392 Hz
AQ 0.3670516 sec
RG 256
DW 2.800 usec
DE 18.00 usec
TE 298.0 K
D1 3.00000000 sec
TD0 1

======== CHANNEL f1 ========
SFO1 564.6299196 MHz
NUC1 19F
P1 18.25 usec

F2 - Processing parameters
SI 131072
SF 564.6864253 MHz
WDW no
SSB 0
LB 0.00 Hz
GB 0
PC 1.00

1D NMR plot parameters
CX 22.80 cm
CY 15.00 cm
F1P 58.050 ppm
F1 32780.04 Hz
F2P -258.181 ppm
F2 -145791.39 Hz
PPMCM 13.86979 ppm/cm
HZCM 7832.08008 Hz/cm

1H spectrum

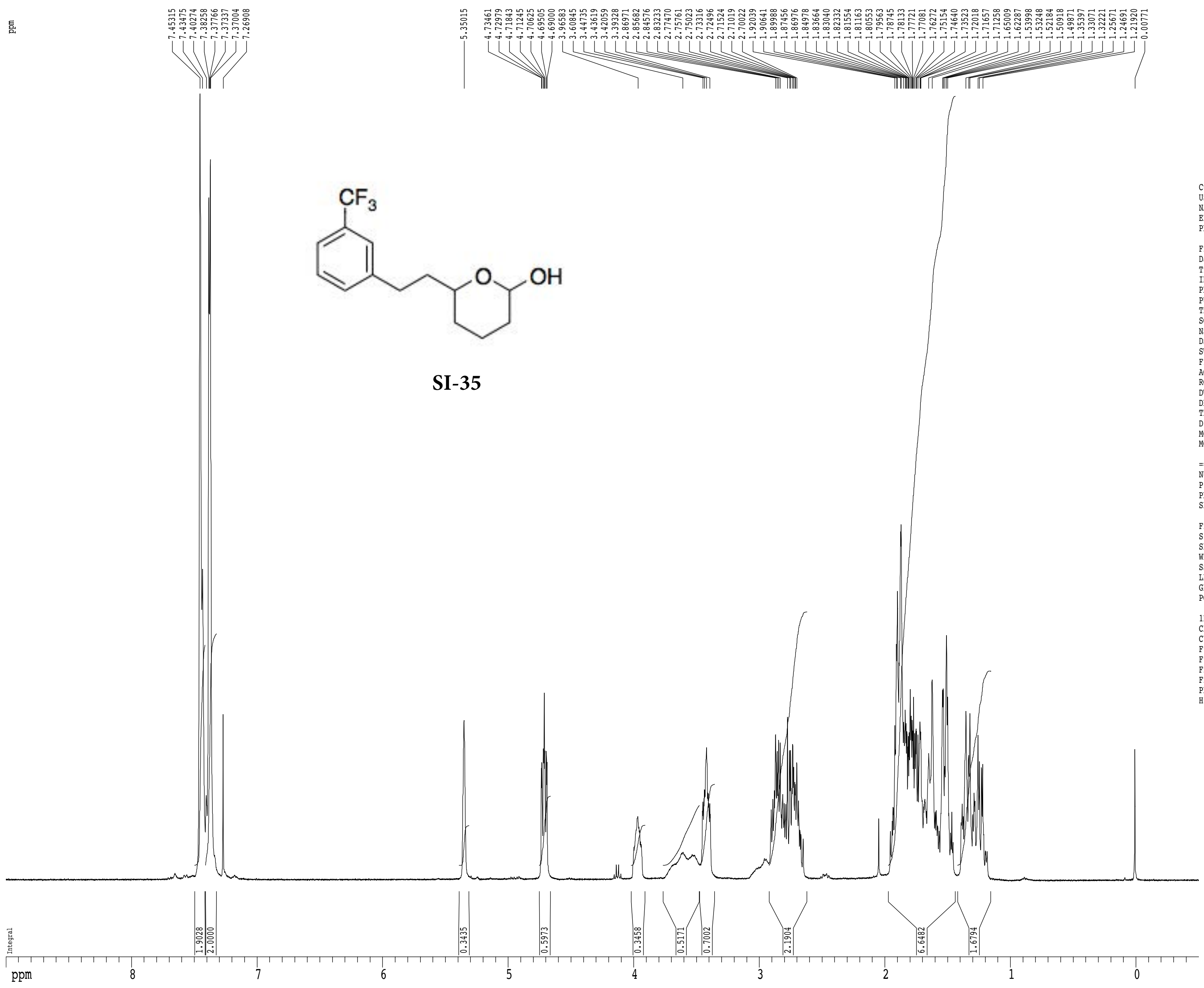

SI-35

Current Data Parameters
USER khewitt1
NAME KAH-IV-303-chk
EXPNO 1
PROCNO 1

F2 - Acquisition Parameters
Date_ 20210707
Time 14.13
INSTRUM drx400
PROBHD 5 mm QNP H/F/P
PULPROG zg30
TD 65536
SOLVENT CDCl3T
NS 8
DS 2
SWH 6410.256 Hz
FIDRES 0.097813 Hz
AQ 5.1118579 sec
RG 101.6
DW 78.000 usec
DE 4.50 usec
TE 298.0 K
D1 0.10000000 sec
MCREST 0.00000000 sec
MCWRK 0.01500000 sec

======== CHANNEL f1 ========
NUC1 1H
P1 12.00 usec
PL1 -1.60 dB
SFO1 400.1328009 MHz

F2 - Processing parameters
SI 65536
SF 400.1300175 MHz
WDW no
SSB 0
LB 0.00 Hz
GB 0
PC 2.00

1D NMR plot parameters
CX 22.80 cm
CY 15.00 cm
F1P 9.000 ppm
F1 3601.17 Hz
F2P -0.500 ppm
F2 -200.06 Hz
PPMCM 0.41667 ppm/cm
HZCM 166.72084 Hz/cm

C13.c

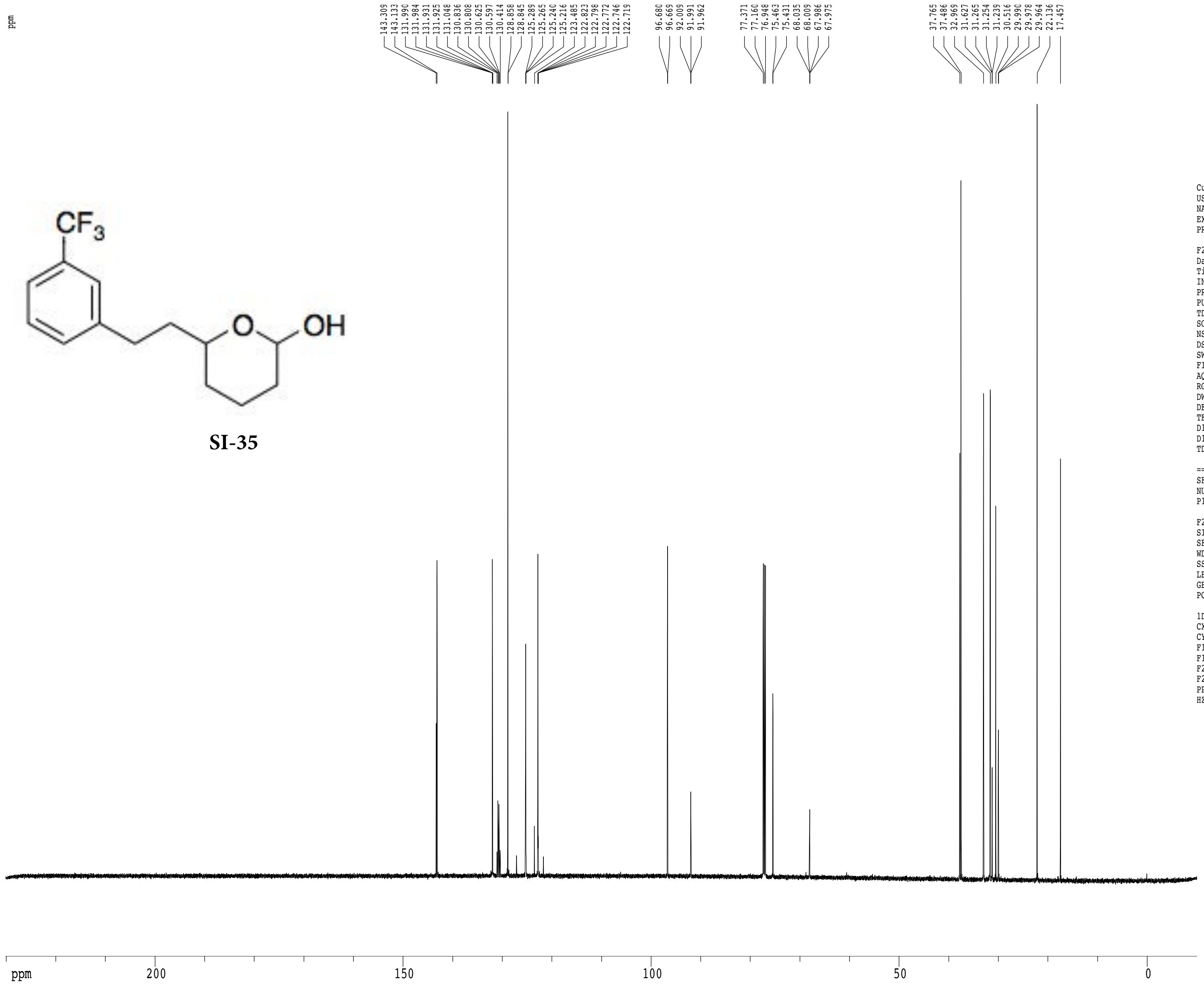

SI-35

Current Data Parameters
USER khewitt1
NAME KAH-IV-303-Z
EXPNO 1
PROCNO 1

F2 - Acquisition Parameters
Date_ 20210707
Time 15.42
INSTRUM av600
PROBHD 5 mm CPBBO BB-
PULPROG zgdc30
TD 65536
SOLVENT CDCl3T
NS 471
DS 4
SWH 36231.883 Hz
FIDRES 0.552855 Hz
AQ 0.9044468 sec
RG 2050
DW 13.800 usec
DE 19.63 usec
TE 298.0 K
D1 0.40000001 sec
D11 0.03000000 sec
TD0 1

======== CHANNEL f1 ========
SFO1 150.9194080 MHz
NUC1 13C
P1 10.10 usec

F2 - Processing parameters
SI 65536
SF 150.9027975 MHz
WDW no
SSB 0
LB 0.00 Hz
GB 0
PC 1.00

1D NMR plot parameters
CX 22.80 cm
CY 15.00 cm
F1P 230.125 ppm
F1 34726.45 Hz
F2P -9.976 ppm
F2 -1505.43 Hz
PPMCM 10.53074 ppm/cm
HZCM 1589.11780 Hz/cm

f19.c

ppm

-62.560
-62.572

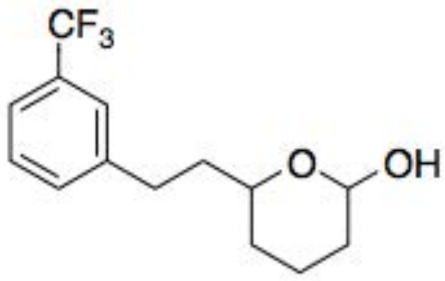

SI-35

Current Data Parameters
USER khewitt1
NAME KAH-IV-303-Z
EXPNO 3
PROCNO 1

F2 - Acquisition Parameters
Date_ 20210707
Time 15.59
INSTRUM av600
PROBHD 5 mm CPBBO BB-
PULPROG zgflqn30
TD 131072
SOLVENT CDCl3T
NS 16
DS 2
SWH 178571.422 Hz
FIDRES 1.362392 Hz
AQ 0.3670516 sec
RG 362
DW 2.800 usec
DE 18.00 usec
TE 298.0 K
D1 3.00000000 sec
TD0 1

======== CHANNEL f1 ========
SFO1 564.6299196 MHz
NUC1 19F
P1 18.25 usec

F2 - Processing parameters
SI 131072
SF 564.6864190 MHz
WDW no
SSB 0
LB 0.00 Hz
GB 0
PC 1.00

1D NMR plot parameters
CX 22.80 cm
CY 15.00 cm
F1P 58.061 ppm
F1 32786.34 Hz
F2P -258.170 ppm
F2 -145785.09 Hz
PPMCM 13.86979 ppm/cm
HZCM 7832.08057 Hz/cm

ppm 0 -50 -100 -150 -200 -250

h1.c

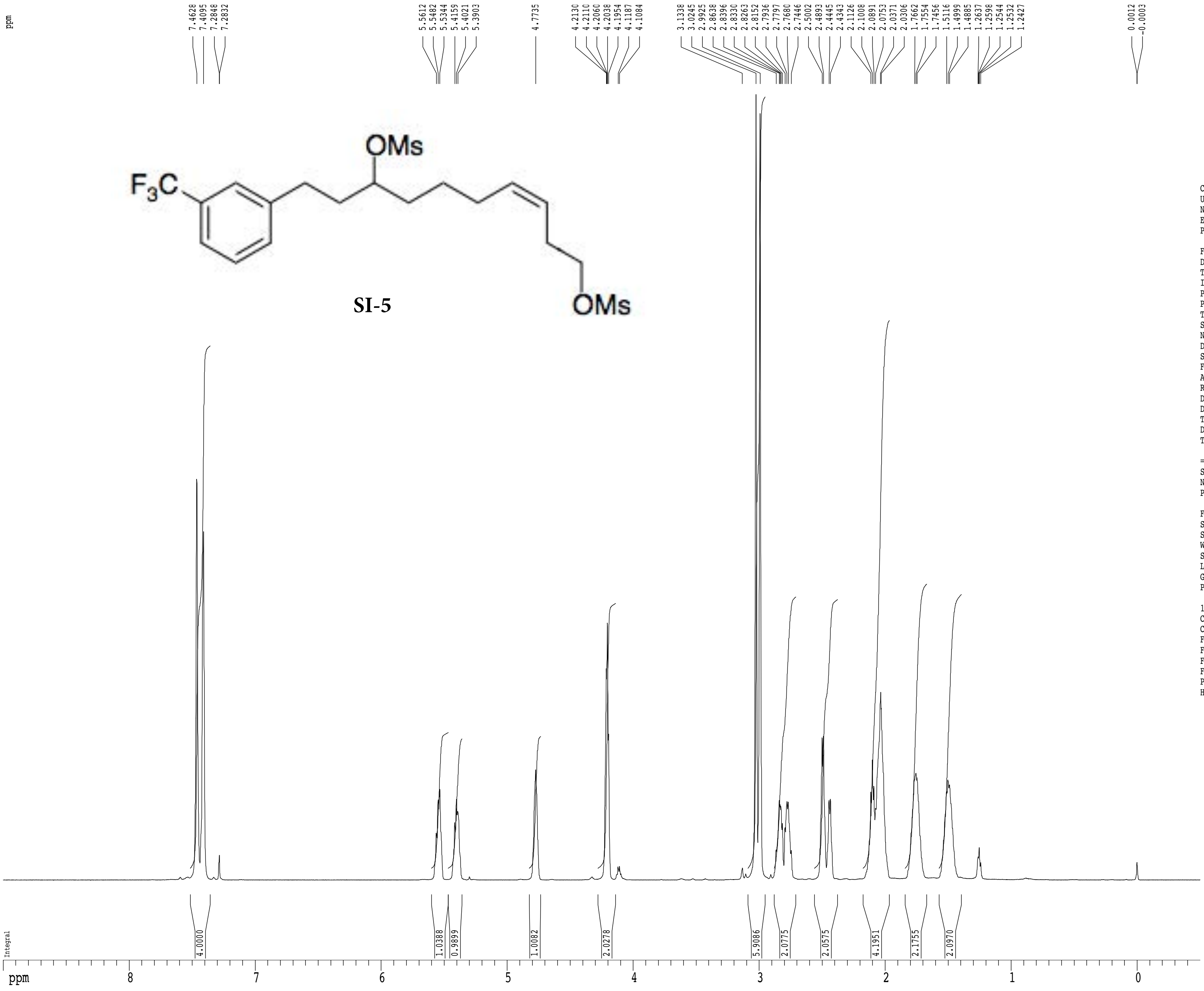

SI-5

Current Data Parameters
USER khewitt1
NAME KAH-V-025-Z
EXPNO 3
PROCNO 1

F2 - Acquisition Parameters
Date_ 20210713
Time 14.04
INSTRUM av600
PROBHD 5 mm CPBBO BB-
PULPROG zg30
TD 98074
SOLVENT CDCl3T
NS 8
DS 2
SWH 9615.385 Hz
FIDRES 0.098042 Hz
AQ 5.0998979 sec
RG 10
DW 52.000 usec
DE 14.23 usec
TE 298.0 K
D1 0.10000000 sec
TD0 1

======== CHANNEL f1 ========
SFO1 600.1342009 MHz
NUC1 1H
P1 9.50 usec

F2 - Processing parameters
SI 65536
SF 600.1300203 MHz
WDW no
SSB 0
LB 0.00 Hz
GB 0
PC 1.00

1D NMR plot parameters
CX 22.80 cm
CY 15.00 cm
F1P 9.000 ppm
F1 5401.17 Hz
F2P -0.500 ppm
F2 -300.06 Hz
PPMCM 0.41667 ppm/cm
HZCM 250.05418 Hz/cm

c13.c

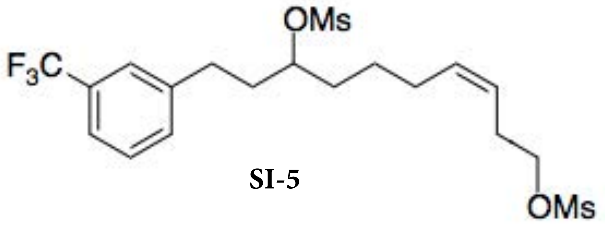

SI-5

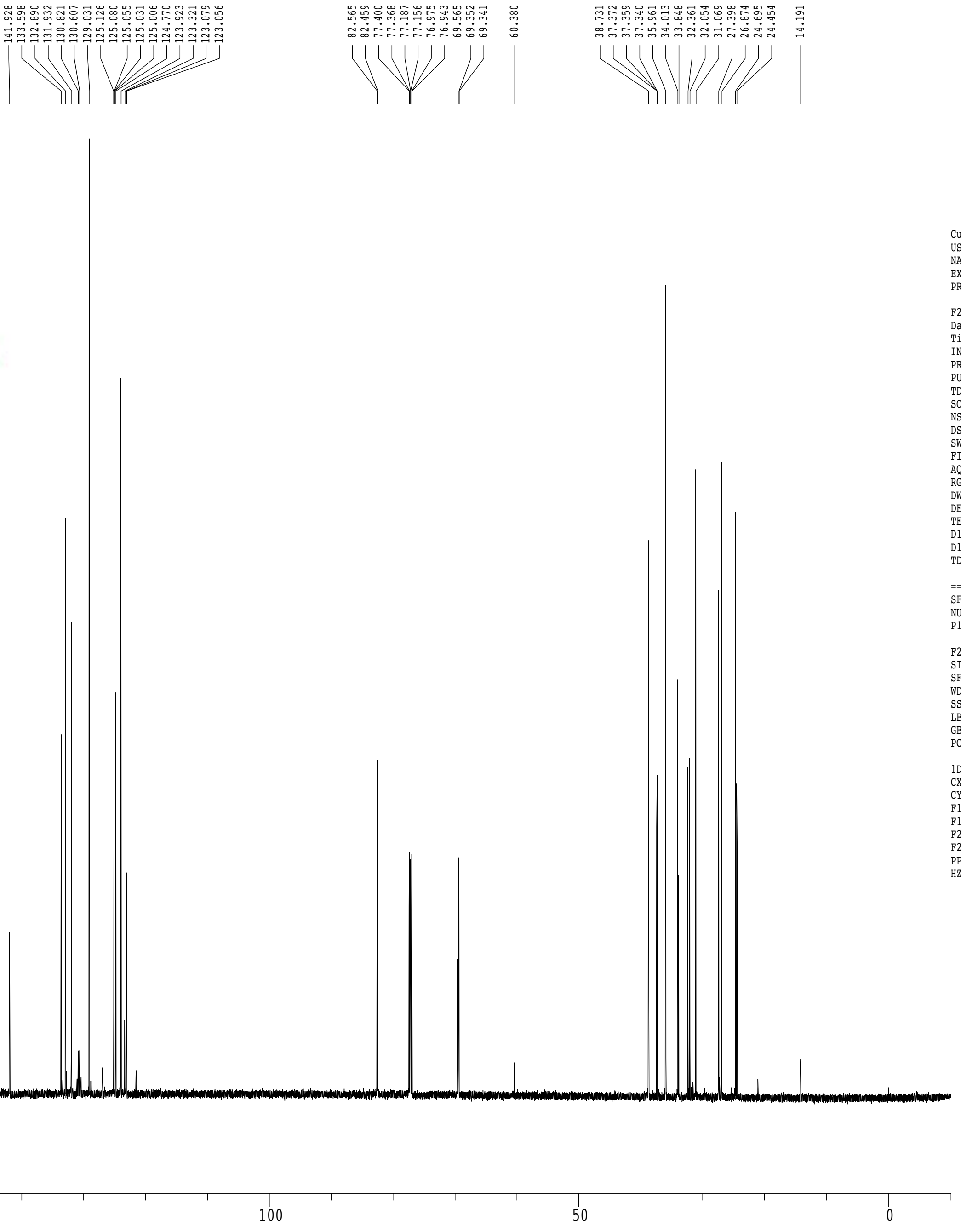

Current Data Parameters
USER khewitt1
NAME KAH-V-025-Z
EXPNO 1
PROCNO 1

F2 - Acquisition Parameters
Date_ 20210713
Time 13.57
INSTRUM av600
PROBHD 5 mm CPBBO BB-
PULPROG zgdc30
TD 65536
SOLVENT CDCl3T
NS 128
DS 4
SWH 36231.883 Hz
FIDRES 0.552855 Hz
AQ 0.9044468 sec
RG 2050
DW 13.800 usec
DE 19.63 usec
TE 298.0 K
D1 0.40000001 sec
D11 0.03000000 sec
TD0 1

======== CHANNEL f1 ========
SFO1 150.9194080 MHz
NUC1 13C
P1 10.10 usec

F2 - Processing parameters
SI 65536
SF 150.9028113 MHz
WDW no
SSB 0
LB 0.00 Hz
GB 0
PC 1.00

1D NMR plot parameters
CX 22.80 cm
CY 15.00 cm
F1P 230.033 ppm
F1 34712.62 Hz
F2P -10.068 ppm
F2 -1519.26 Hz
PPMCM 10.53074 ppm/cm
HZCM 1589.11768 Hz/cm

19F Spectrum

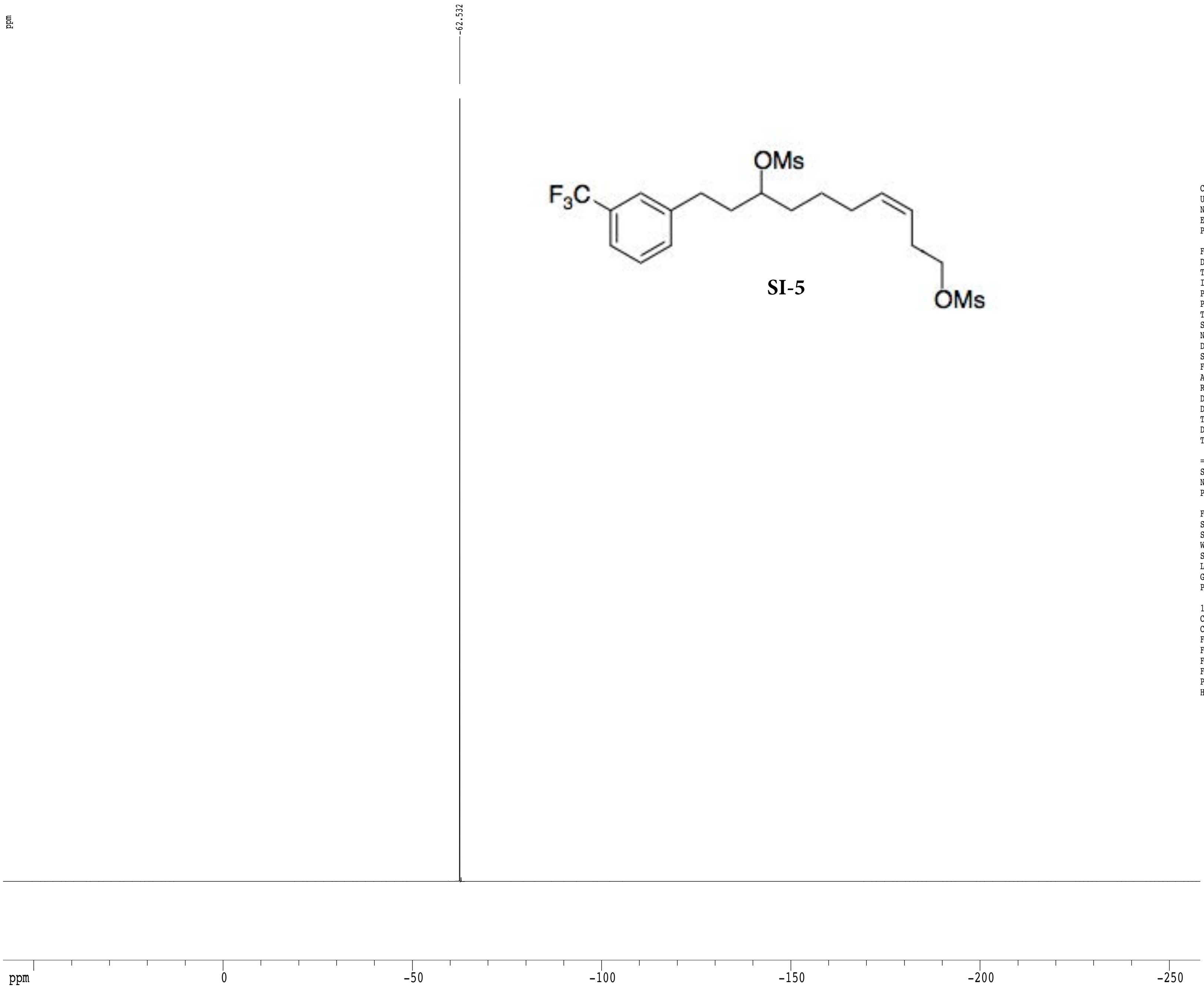

SI-5

Current Data Parameters
USER khewitt1
NAME KAH-V-025-F
EXPNO 2
PROCNO 1

F2 - Acquisition Parameters
Date_ 20220117
Time 17.13
INSTRUM av600
PROBHD 5 mm CPBBO BB-
PULPROG zgflqn30
TD 131072
SOLVENT CDCl3T
NS 16
DS 2
SWH 178571.422 Hz
FIDRES 1.362392 Hz
AQ 0.3670516 sec
RG 575
DW 2.800 usec
DE 18.00 usec
TE 298.0 K
D1 3.00000000 sec
TD0 1

======== CHANNEL f1 ========
SFO1 564.6299196 MHz
NUC1 19F
P1 18.25 usec

F2 - Processing parameters
SI 131072
SF 564.6864101 MHz
WDW no
SSB 0
LB 0.00 Hz
GB 0
PC 1.00

1D NMR plot parameters
CX 22.80 cm
CY 15.00 cm
F1P 58.077 ppm
F1 32795.21 Hz
F2P -258.154 ppm
F2 -145776.22 Hz
PPMCM 13.86979 ppm/cm
HZCM 7832.08057 Hz/cm

1H spectrum

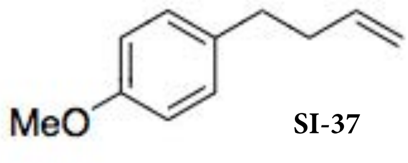

SI-37

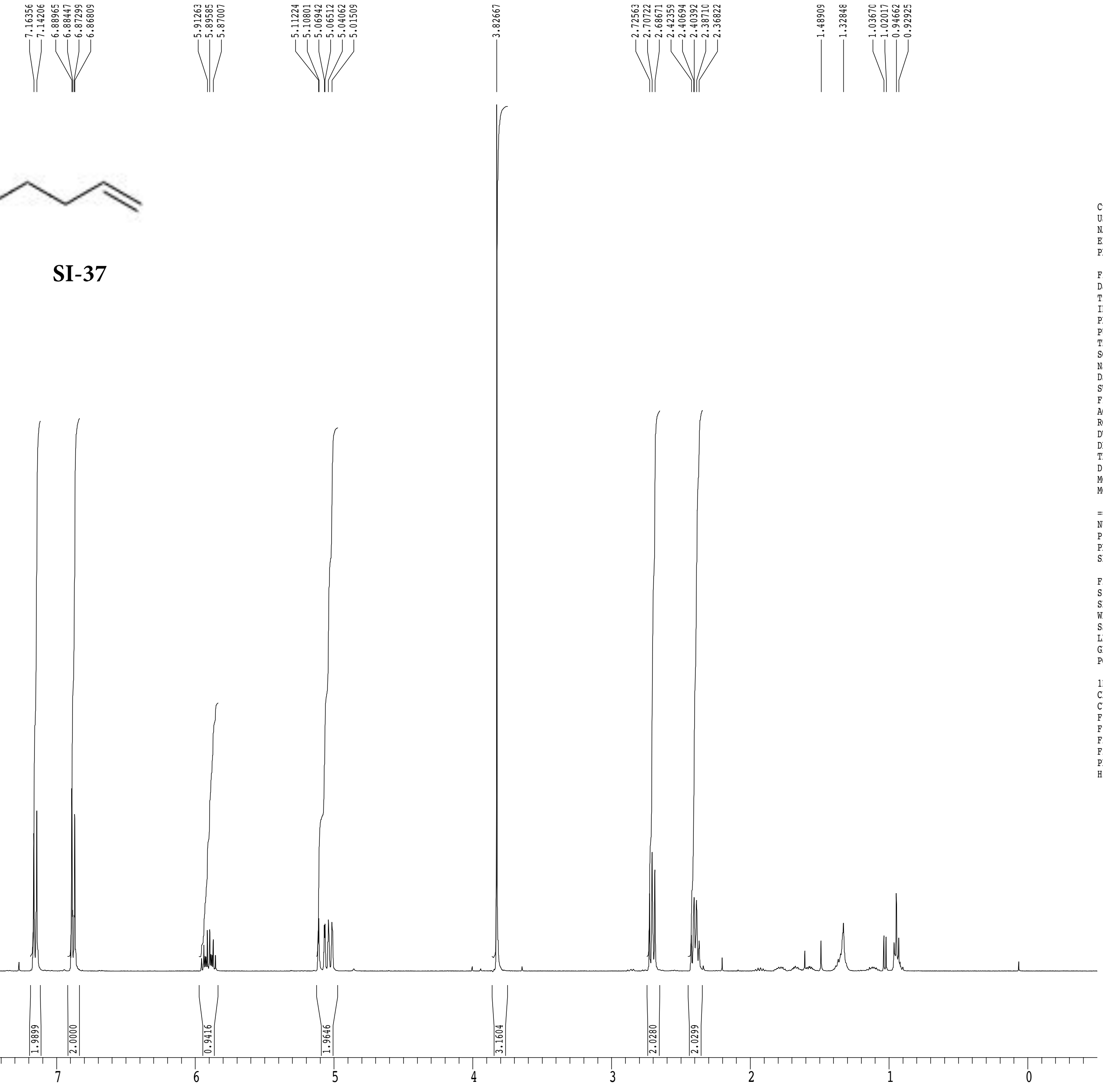

Current Data Parameters
USER khewitt1
NAME KAH-V-116-1
EXPNO 1
PROCNO 1

F2 - Acquisition Parameters
Date_ 20210929
Time 18.03
INSTRUM drx400
PROBHD 5 mm Multinucl
PULPROG zg30
TD 38460
SOLVENT CDCl3T
NS 8
DS 2
SWH 6410.256 Hz
FIDRES 0.166673 Hz
AQ 2.9999299 sec
RG 71.8
DW 78.000 usec
DE 4.50 usec
TE 297.9 K
D1 0.10000000 sec
MCREST 0.00000000 sec
MCWRK 0.01500000 sec

======== CHANNEL f1 ========
NUC1 1H
P1 12.00 usec
PL1 -1.10 dB
SFO1 400.1328009 MHz

F2 - Processing parameters
SI 65536
SF 400.1300175 MHz
WDW no
SSB 0
LB 0.00 Hz
GB 0
PC 2.00

1D NMR plot parameters
CX 22.80 cm
CY 15.00 cm
F1P 9.000 ppm
F1 3601.17 Hz
F2P -0.500 ppm
F2 -200.06 Hz
PPMCM 0.41667 ppm/cm
HZCM 166.72084 Hz/cm

13C spectrum with 1H decoupling

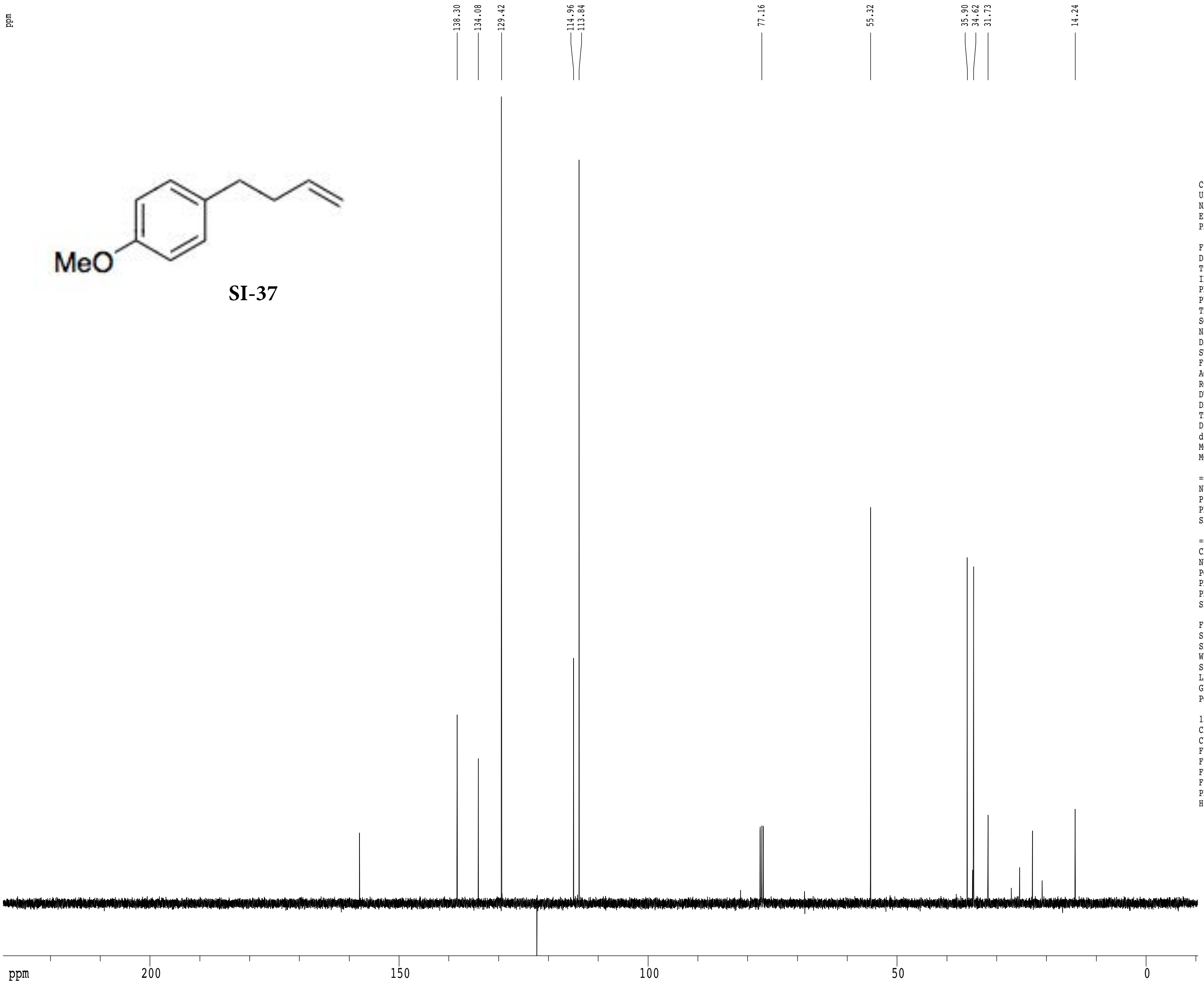


```
Current Data Parameters
USER            khewitt1
NAME          KAH-V-116-1
EXPNO                  2
PROCNO                 1

F2 - Acquisition Parameters
Date_           20210929
Time               18.05
INSTRUM           drx400
PROBHD   5 mm Multinucl
PULPROG          zgdc30
TD                 65536
SOLVENT            CDCl3
NS                   136
DS                     4
SWH           24154.590 Hz
FIDRES         0.368570 Hz
AQ            1.3566452 sec
RG                9195.2
DW               20.700 usec
DE                20.39 usec
TE                297.9 K
D1           0.10000000 sec
d11          0.03000000 sec
MCREST       0.00000000 sec
MCWRK        0.01500000 sec

======== CHANNEL f1 ========
NUC1                 13C
P1                  9.00 usec
PL1                -2.50 dB
SFO1         100.6237964 MHz

======== CHANNEL f2 ========
CPDPRG2          waltz16
NUC2                  1H
PCPD2              80.00 usec
PL2                -1.10 dB
PL12               15.60 dB
SFO2         400.1328009 MHz

F2 - Processing parameters
SI                 65536
SF           100.6127632 MHz
WDW                   no
SSB                    0
LB                  0.00 Hz
GB                     0
PC                  1.00

1D NMR plot parameters
CX                 22.80 cm
CY                 15.50 cm
F1P              229.496 ppm
F1              23090.21 Hz
F2P              -10.579 ppm
F2              -1064.37 Hz
PPMCM           10.52959 ppm/cm
HZCM          1059.41150 Hz/cm
```

1H spectrum

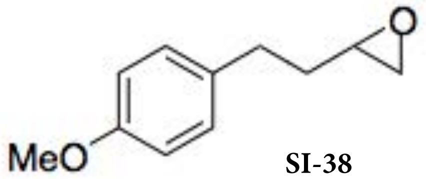

SI-38

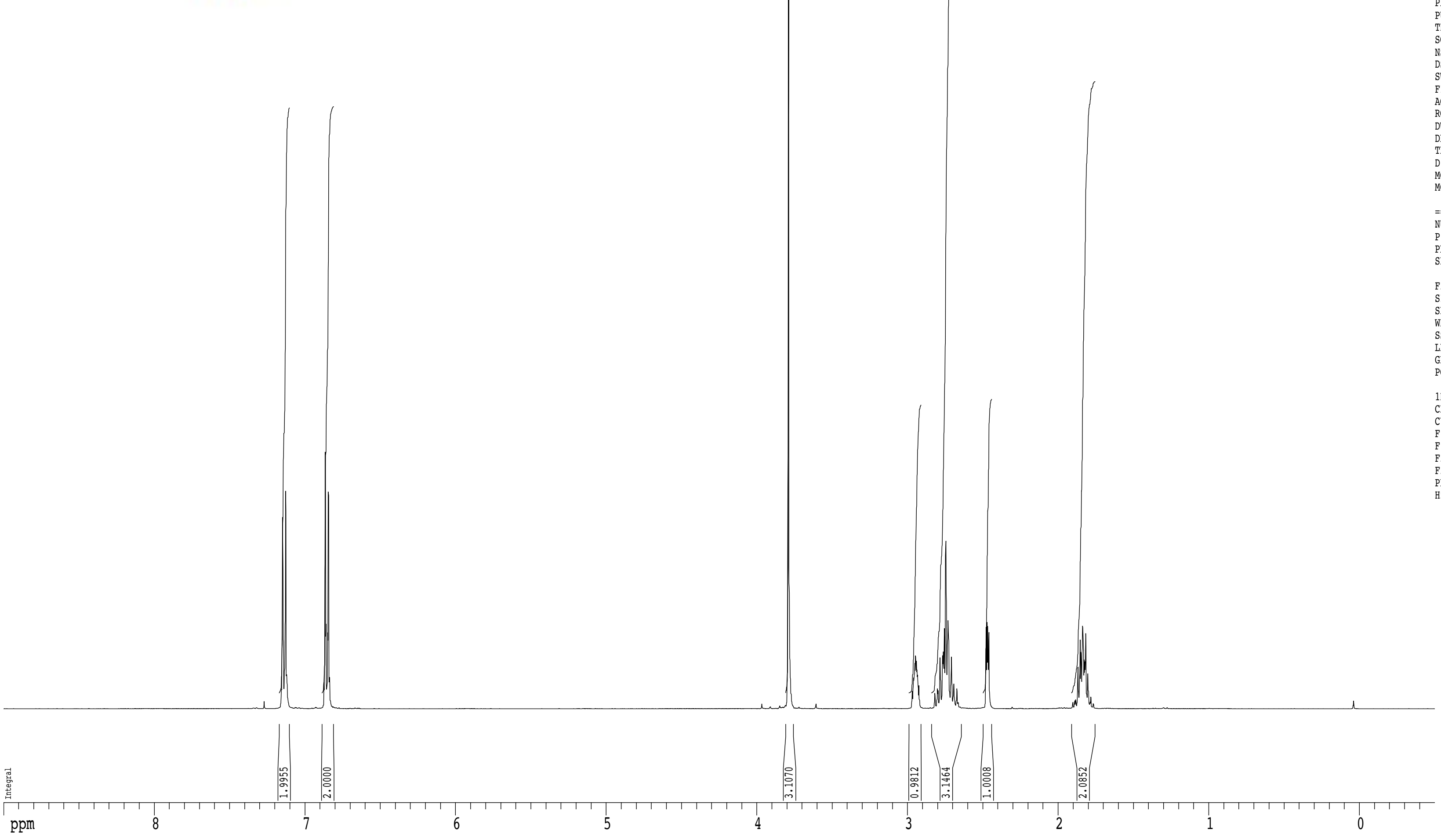

Current Data Parameters
USER khewitt1
NAME KAH-V-117-Z
EXPNO 1
PROCNO 1

F2 - Acquisition Parameters
Date_ 20211004
Time 14.24
INSTRUM drx400
PROBHD 5 mm Multinucl
PULPROG zg30
TD 38460
SOLVENT CDCl3T
NS 8
DS 2
SWH 6410.256 Hz
FIDRES 0.166673 Hz
AQ 2.9999299 sec
RG 45.3
DW 78.000 usec
DE 4.50 usec
TE 298.0 K
D1 0.10000000 sec
MCREST 0.00000000 sec
MCWRK 0.01500000 sec

======== CHANNEL f1 ========
NUC1 1H
P1 12.00 usec
PL1 -1.10 dB
SFO1 400.1328009 MHz

F2 - Processing parameters
SI 65536
SF 400.1300175 MHz
WDW no
SSB 0
LB 0.00 Hz
GB 0
PC 2.00

1D NMR plot parameters
CX 22.80 cm
CY 15.00 cm
F1P 9.000 ppm
F1 3601.17 Hz
F2P -0.500 ppm
F2 -200.06 Hz
PPMCM 0.41667 ppm/cm
HZCM 166.72084 Hz/cm

13C spectrum with 1H decoupling

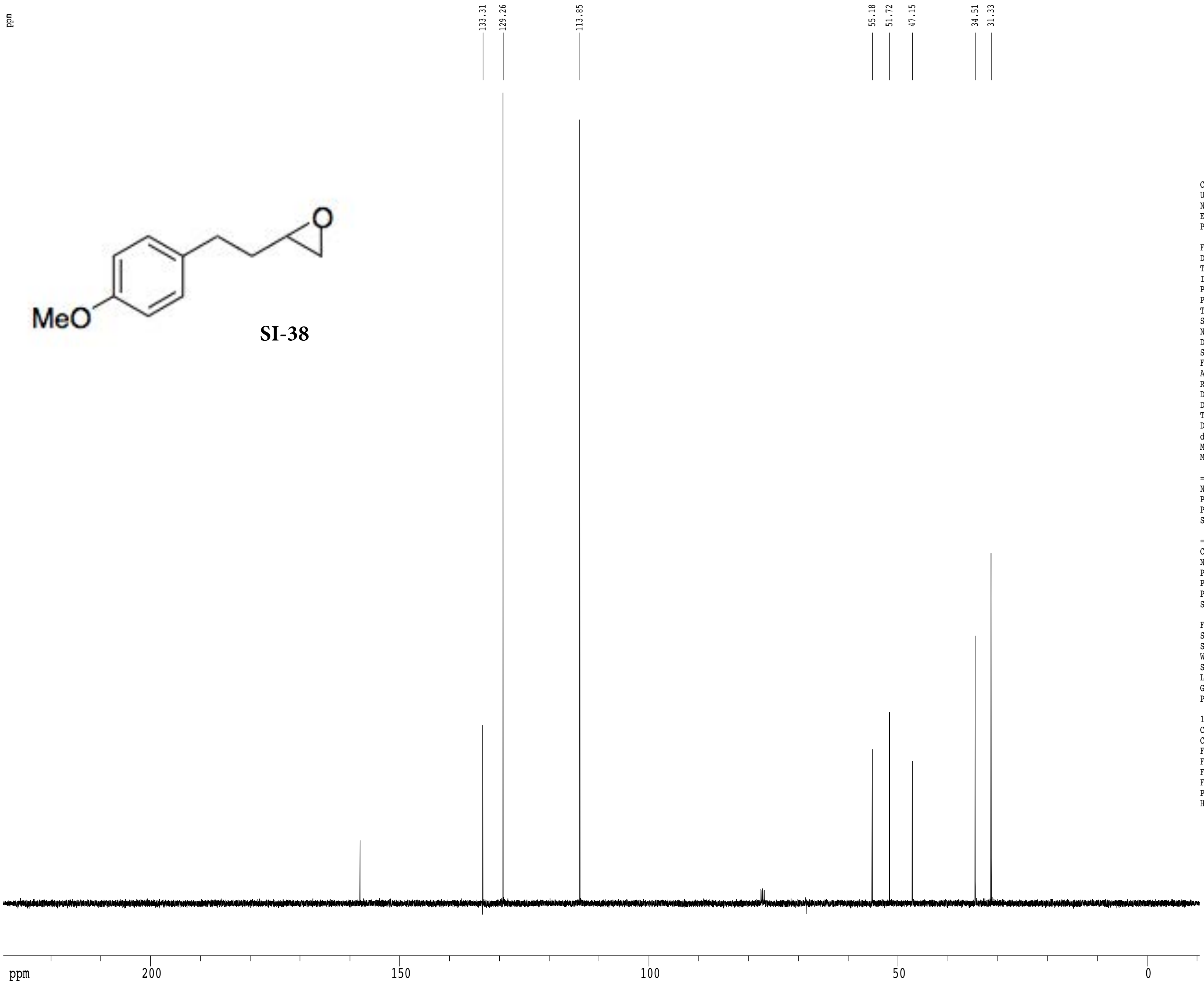

SI-38


```
Current Data Parameters
USER            khewitt1
NAME         KAH-V-117-Z
EXPNO                  2
PROCNO                 1

F2 - Acquisition Parameters
Date_           20211004
Time               14.26
INSTRUM           drx400
PROBHD   5 mm Multinucl
PULPROG          zgdc30
TD                 65536
SOLVENT            CDCl3
NS                    88
DS                     4
SWH           24154.590 Hz
FIDRES         0.368570 Hz
AQ            1.3566452 sec
RG                 13004
DW                20.700 usec
DE                 20.39 usec
TE                 298.0 K
D1           0.10000000 sec
d11          0.03000000 sec
MCREST       0.00000000 sec
MCWRK        0.01500000 sec

======== CHANNEL f1 ========
NUC1                 13C
P1                  9.00 usec
PL1                -2.50 dB
SFO1        100.6237964 MHz

======== CHANNEL f2 ========
CPDPRG2          waltz16
NUC2                  1H
PCPD2              80.00 usec
PL2                -1.10 dB
PL12               15.60 dB
SFO2        400.1328009 MHz

F2 - Processing parameters
SI                 65536
SF           100.6127750 MHz
WDW                   no
SSB                    0
LB                  0.00 Hz
GB                     0
PC                  1.00

1D NMR plot parameters
CX                 22.80 cm
CY                 15.50 cm
F1P              229.496 ppm
F1              23090.22 Hz
F2P              -10.579 ppm
F2              -1064.37 Hz
PPMCM           10.52959 ppm/cm
HZCM          1059.41174 Hz/cm
```

1H spectrum

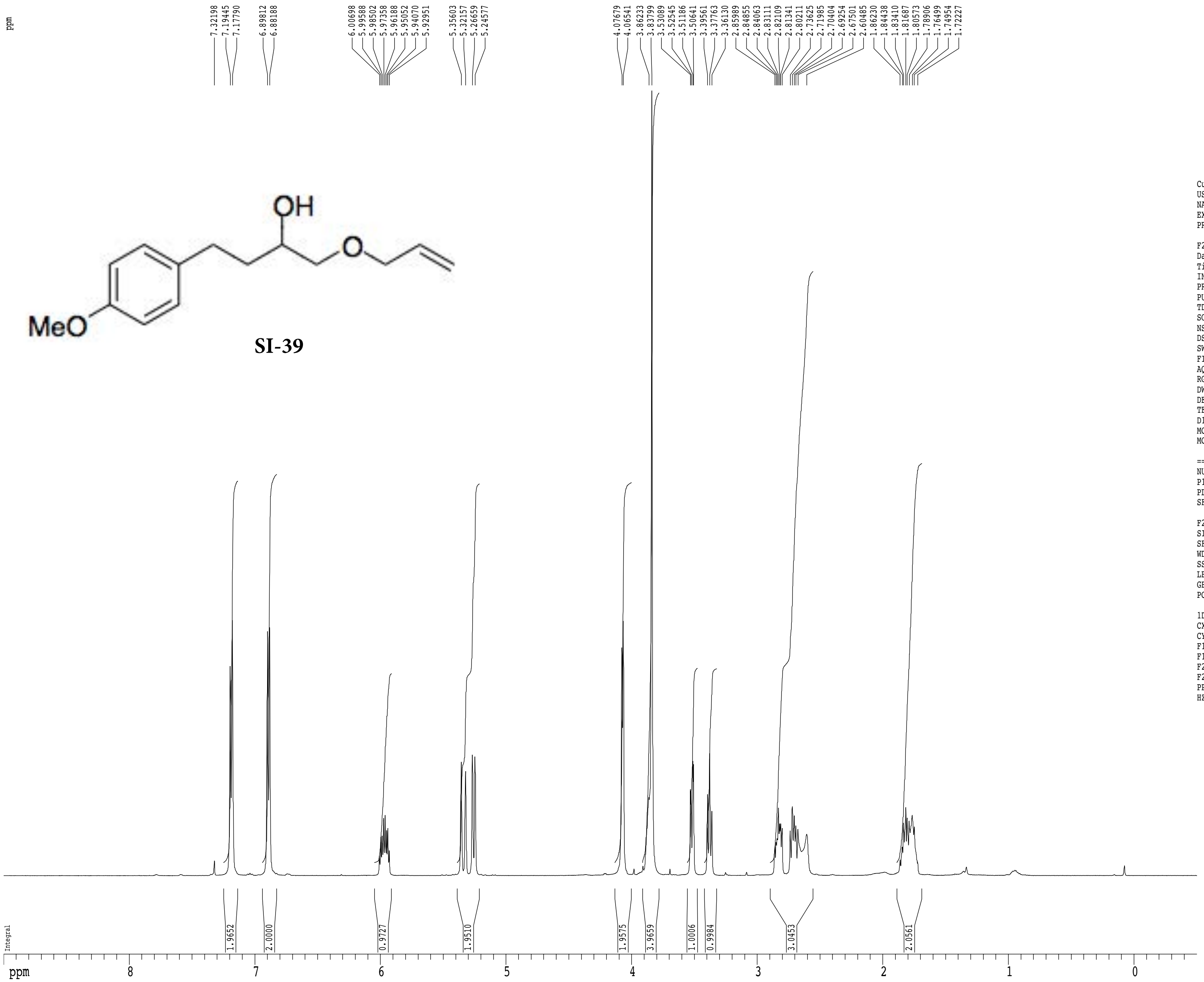

SI-39

Current Data Parameters
USER khewitt1
NAME KAH-V-122-Z
EXPNO 1
PROCNO 1

F2 - Acquisition Parameters
Date_ 20211008
Time 14.43
INSTRUM cryo500
PROBHD 5 mm CPTCI 1H-
PULPROG zg30
TD 81728
SOLVENT CDCl3
NS 8
DS 2
SWH 8012.820 Hz
FIDRES 0.098043 Hz
AQ 5.0998774 sec
RG 4.5
DW 62.400 usec
DE 6.00 usec
TE 298.0 K
D1 0.10000000 sec
MCREST 0.00000000 sec
MCWRK 0.01500000 sec

======== CHANNEL f1 ========
NUC1 1H
P1 9.75 usec
PL1 1.60 dB
SFO1 500.2235015 MHz

F2 - Processing parameters
SI 65536
SF 500.2200000 MHz
WDW no
SSB 0
LB 0.00 Hz
GB 0
PC 1.00

1D NMR plot parameters
CX 22.80 cm
CY 15.00 cm
F1P 9.000 ppm
F1 4501.98 Hz
F2P -0.500 ppm
F2 -250.11 Hz
PPMCM 0.41667 ppm/cm
HZCM 208.42500 Hz/cm

Z-restored spin-echo 13C spectrum with 1H decoupling

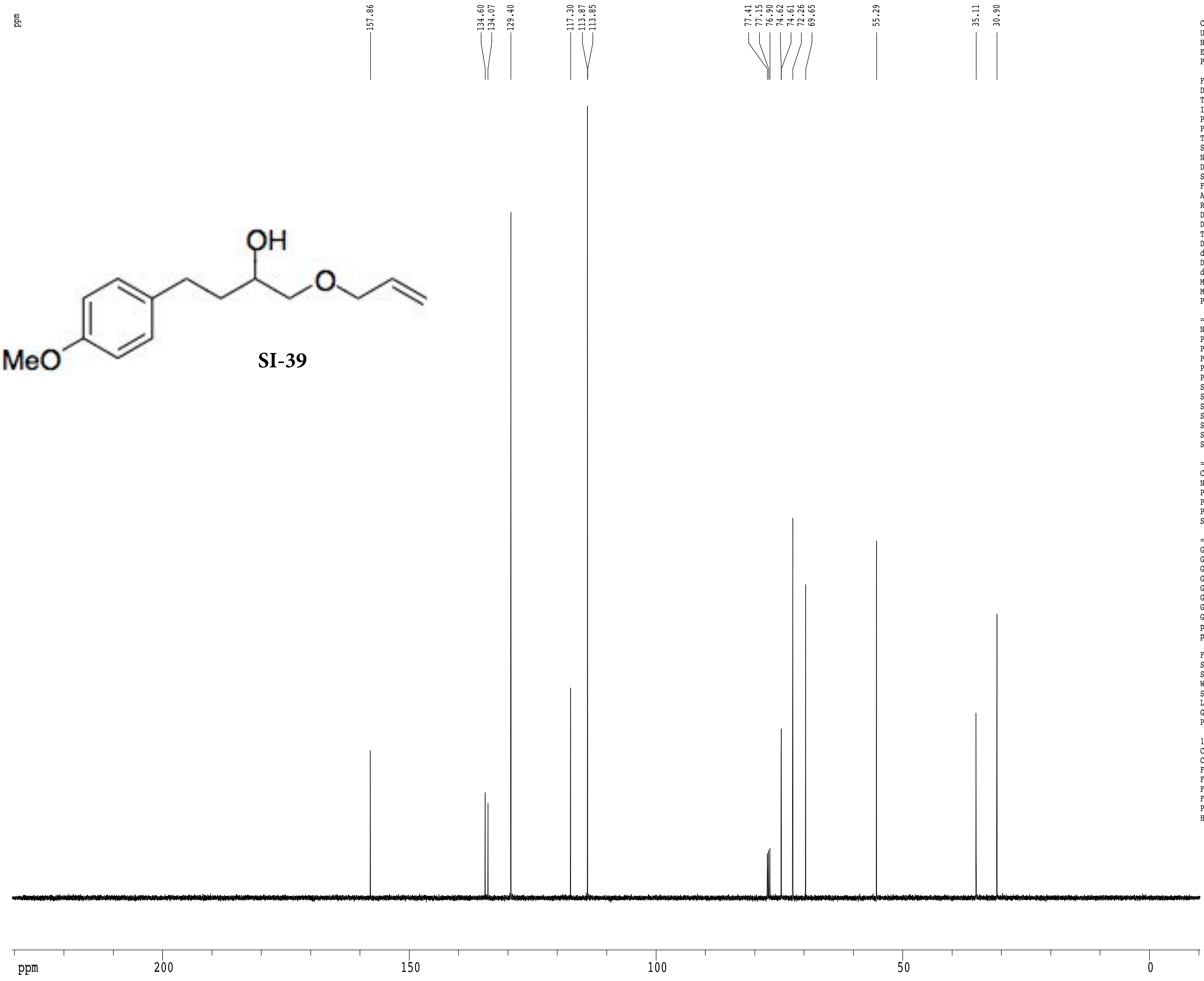

SI-39

```
Current Data Parameters
USER            khewitt1
NAME          KAH-V-122-Z
EXPNO                  2
PROCNO                 1

F2 - Acquisition Parameters
Date_           20211008
Time               14.46
INSTRUM          cryo500
PROBHD   5 mm CPTCI 1H-
PULPROG  SpinEchopg30gp2.prd
TD                 65536
SOLVENT            CDCl3
NS                    88
DS                    16
SWH            30303.031 Hz
FIDRES          0.462388 Hz
AQ             1.0813940 sec
RG                7298.2
DW                16.500 usec
DE                  6.00 usec
TE                 298.0 K
D1            0.25000000 sec
d11           0.03000000 sec
D16           0.00020000 sec
d17           0.00019600 sec
MCREST        0.00000000 sec
MCWRK         0.01500000 sec
P2                 37.70 usec

======== CHANNEL f1 ========
NUC1                 13C
P1                 18.85 usec
P12              2000.00 usec
P20               500.00 usec
PL0               120.00 dB
PL1                -1.00 dB
SFO1         125.7942548 MHz
SP2                 1.55 dB
SP4                 1.55 dB
SPNAM2       Crp60comp.4
SPNAM4   Crp60,0.5,20.1
SPOFF2              0.00 Hz
SPOFF4              0.00 Hz

======== CHANNEL f2 ========
CPDPRG2          waltz16
NUC2                  1H
PCPD2             100.00 usec
PL2                 1.60 dB
PL12               22.00 dB
SFO2         500.2225011 MHz

====== GRADIENT CHANNEL =====
GPNAM1          SINE.100
GPNAM2          SINE.100
GPX1                0.00 %
GPX2                0.00 %
GPY1                0.00 %
GPY2                0.00 %
GPZ1               30.00 %
GPZ2               50.00 %
p15               500.00 usec
p16              1000.00 usec

F2 - Processing parameters
SI                 65536
SF           125.7804190 MHz
WDW                   no
SSB                    0
LB                  0.00 Hz
GB                     0
PC                  2.00

1D NMR plot parameters
CX                 22.80 cm
CY                 15.65 cm
F1P              230.637 ppm
F1              29009.68 Hz
F2P              -10.287 ppm
F2              -1293.96 Hz
PPMCM           10.56688 ppm/cm
HZCM          1329.10706 Hz/cm
```

1H spectrum

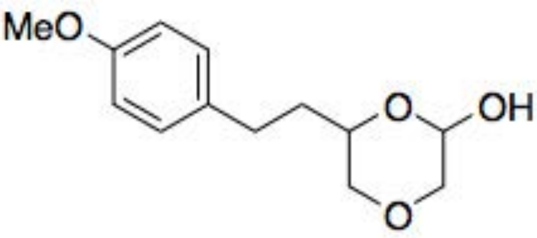

SI-40

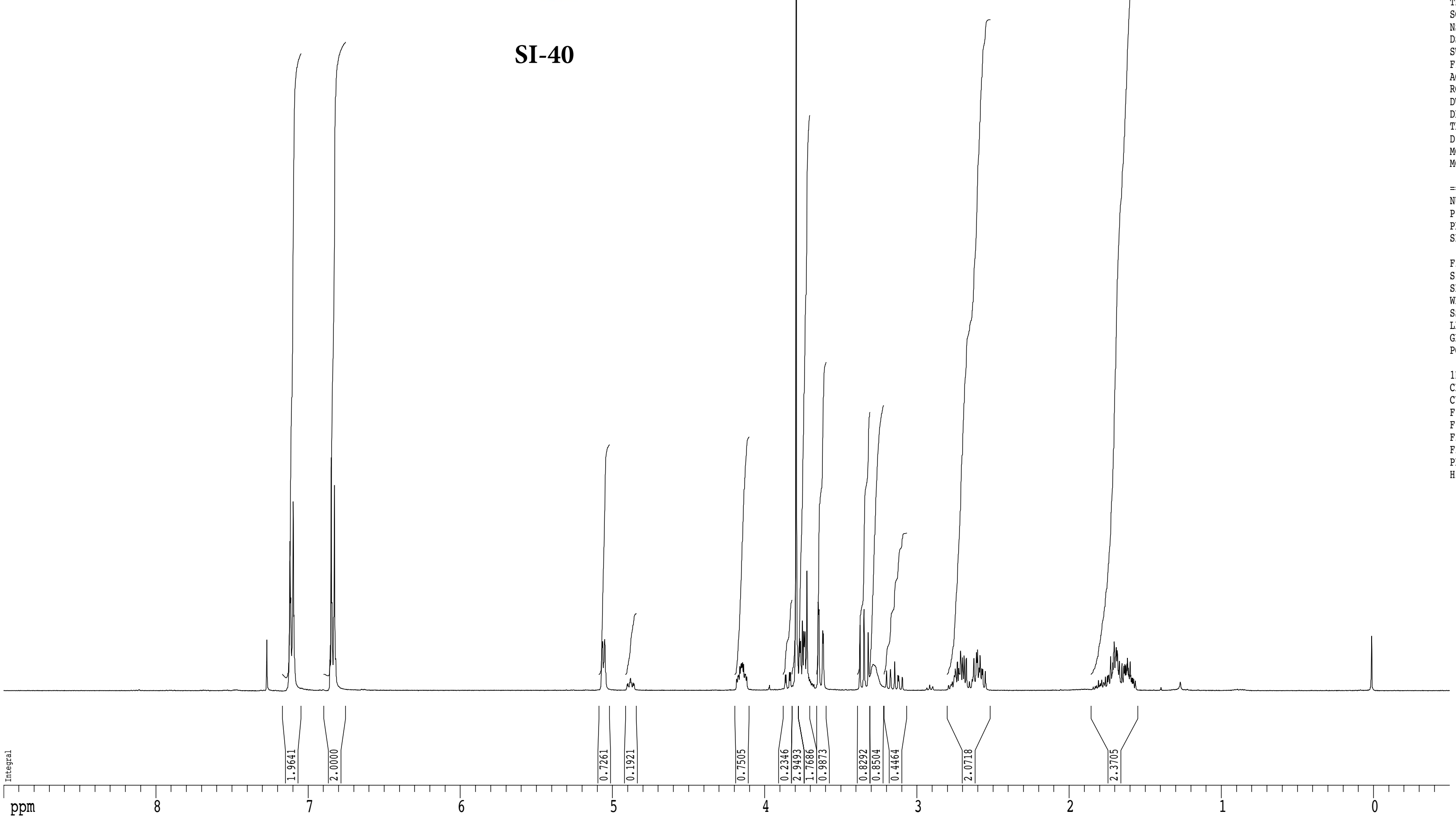


```
Current Data Parameters
USER            khewitt1
NAME          KAH-V-153-Z
EXPNO                  1
PROCNO                 1

F2 - Acquisition Parameters
Date_           20211027
Time               15.15
INSTRUM           drx400
PROBHD   5 mm QNP H/F/P
PULPROG             zg30
TD                 38460
SOLVENT           CDCl3T
NS                     8
DS                     2
SWH             6410.256 Hz
FIDRES          0.166673 Hz
AQ             2.9999299 sec
RG                 161.3
DW                78.000 usec
DE                  4.50 usec
TE                 298.1 K
D1           0.10000000 sec
MCREST       0.00000000 sec
MCWRK        0.01500000 sec

======== CHANNEL f1 ========
NUC1                  1H
P1                 12.00 usec
PL1                -0.90 dB
SFO1        400.1328009 MHz

F2 - Processing parameters
SI                 65536
SF           400.1300175 MHz
WDW                   no
SSB                    0
LB                  0.00 Hz
GB                     0
PC                  2.00

1D NMR plot parameters
CX                 22.80 cm
CY                 15.00 cm
F1P                9.000 ppm
F1               3601.17 Hz
F2P               -0.500 ppm
F2               -200.06 Hz
PPMCM            0.41667 ppm/cm
HZCM           166.72084 Hz/cm
```

13C spectrum with 1H decoupling

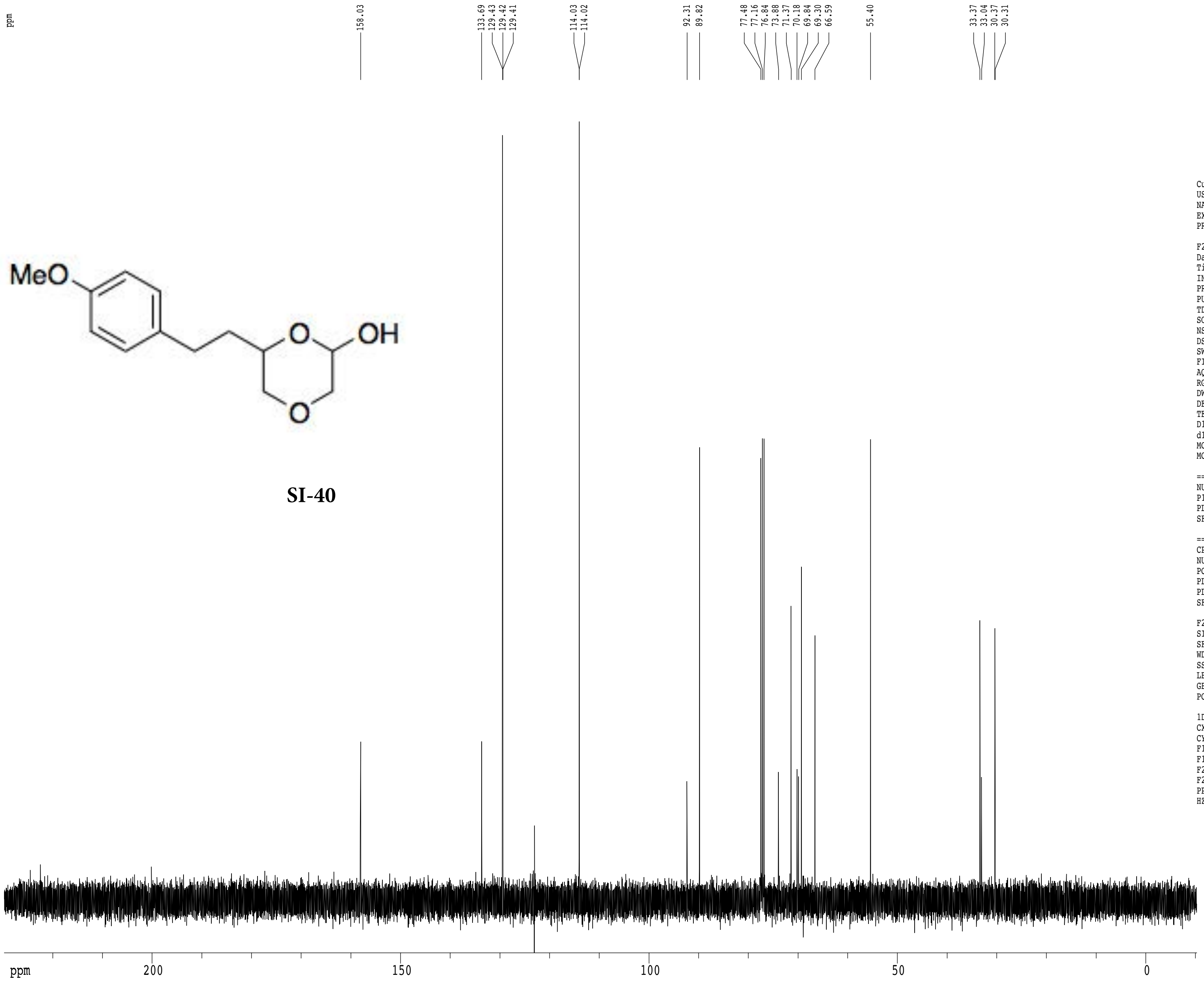

SI-40

```
Current Data Parameters
USER            khewitt1
NAME        KAH-V-153-Z
EXPNO                  2
PROCNO                 1

F2 - Acquisition Parameters
Date_           20211027
Time               15.17
INSTRUM           drx400
PROBHD   5 mm QNP H/F/P
PULPROG           zgdc30
TD                 65536
SOLVENT            CDCl3
NS                   240
DS                     4
SWH            24154.590 Hz
FIDRES          0.368570 Hz
AQ             1.3566452 sec
RG                5160.6
DW                20.700 usec
DE                 20.39 usec
TE                 298.1 K
D1            0.10000000 sec
d11           0.03000000 sec
MCREST        0.00000000 sec
MCWRK         0.01500000 sec

======== CHANNEL f1 ========
NUC1                 13C
P1                  7.90 usec
PL1                -3.00 dB
SFO1         100.6237964 MHz

======== CHANNEL f2 ========
CPDPRG2          waltz16
NUC2                  1H
PCPD2              90.00 usec
PL2                -0.90 dB
PL12               17.00 dB
SFO2         400.1328009 MHz

F2 - Processing parameters
SI                 65536
SF           100.6127595 MHz
WDW                   no
SSB                    0
LB                  0.00 Hz
GB                     0
PC                  1.00

1D NMR plot parameters
CX                 22.80 cm
CY                 15.50 cm
F1P              229.829 ppm
F1              23123.72 Hz
F2P              -10.246 ppm
F2              -1030.88 Hz
PPMCM           10.52960 ppm/cm
HZCM          1059.41199 Hz/cm
```

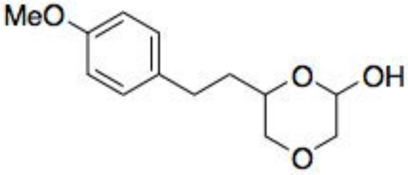

SI-40

gcosy60

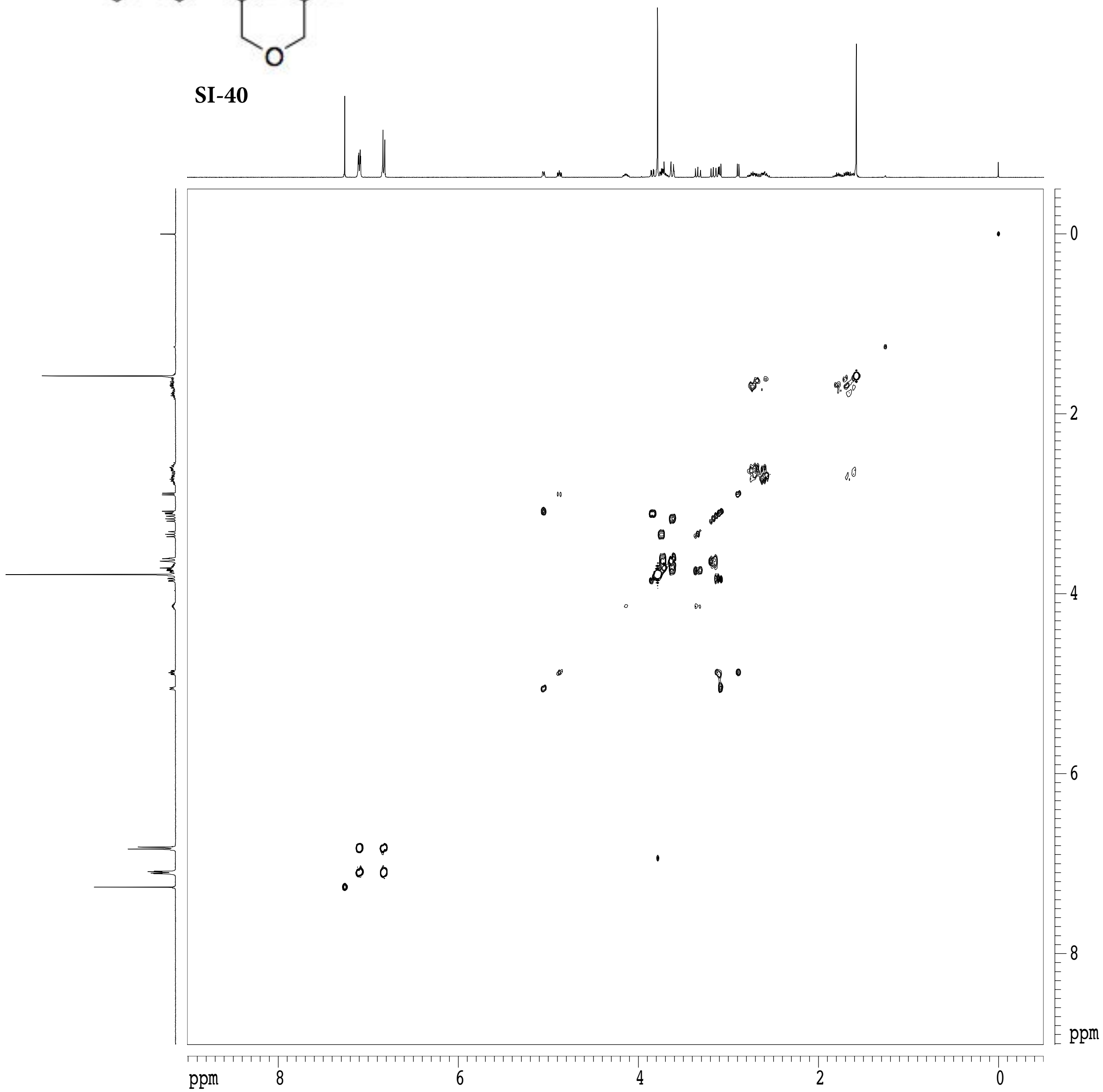


```
Current Data Parameters
USER          khewitt1
NAME     KAH-V-164-03-Z
EXPNO                 2
PROCNO                1

F2 - Acquisition Parameters
Date_          20211111
Time              14.38
INSTRUM          drx400
PROBHD   5 mm QNP H/F/P
PULPROG    cosygp60.prd
TD                 2048
SOLVENT           CDCl3
NS                    1
DS                   16
SWH            6410.256 Hz
FIDRES         3.130008 Hz
AQ            0.1597940 sec
RG                 2048
DW               78.000 usec
DE                 4.50 usec
TE                298.0 K
d0           0.00000300 sec
D1           1.00000000 sec
d13          0.00000300 sec
D16          0.00100000 sec
IN0          0.00015600 sec

======== CHANNEL f1 ========
NUC1                 1H
P1                12.00 usec
PL1               -0.90 dB
SFO1        400.1328009 MHz

====== GRADIENT CHANNEL =====
GPNAM1       SMSQ10.100
GPNAM2       SMSQ10.100
GPX1               0.00 %
GPX2               0.00 %
GPY1               0.00 %
GPY2               0.00 %
GPZ1              10.00 %
GPZ2              10.00 %
P16             1000.00 usec

F1 - Acquisition parameters
ND0                   1
TD                  256
SFO1           400.1328 MHz
FIDRES        25.040064 Hz
SW               16.020 ppm
FnMODE               QF

F2 - Processing parameters
SI                 1024
SF          400.1300214 MHz
WDW                SINE
SSB                   0
LB                 0.00 Hz
GB                    0
PC                 2.00

F1 - Processing parameters
SI                 1024
MC2                  QF
SF          400.1300214 MHz
WDW                SINE
SSB                   0
LB                 0.00 Hz
GB                    0

2D NMR plot parameters
CX2               15.00 cm
CX1               15.00 cm
F2PLO             9.012 ppm
F2LO            3605.86 Hz
F2PHI            -0.500 ppm
F2HI            -200.23 Hz
F1PLO             9.012 ppm
F1LO            3605.86 Hz
F1PHI            -0.500 ppm
F1HI            -200.23 Hz
F2PPMCM         0.63414 ppm/cm
F2HZCM        253.73933 Hz/cm
F1PPMCM         0.63414 ppm/cm
F1HZCM        253.73933 Hz/cm
```

1H spectrum

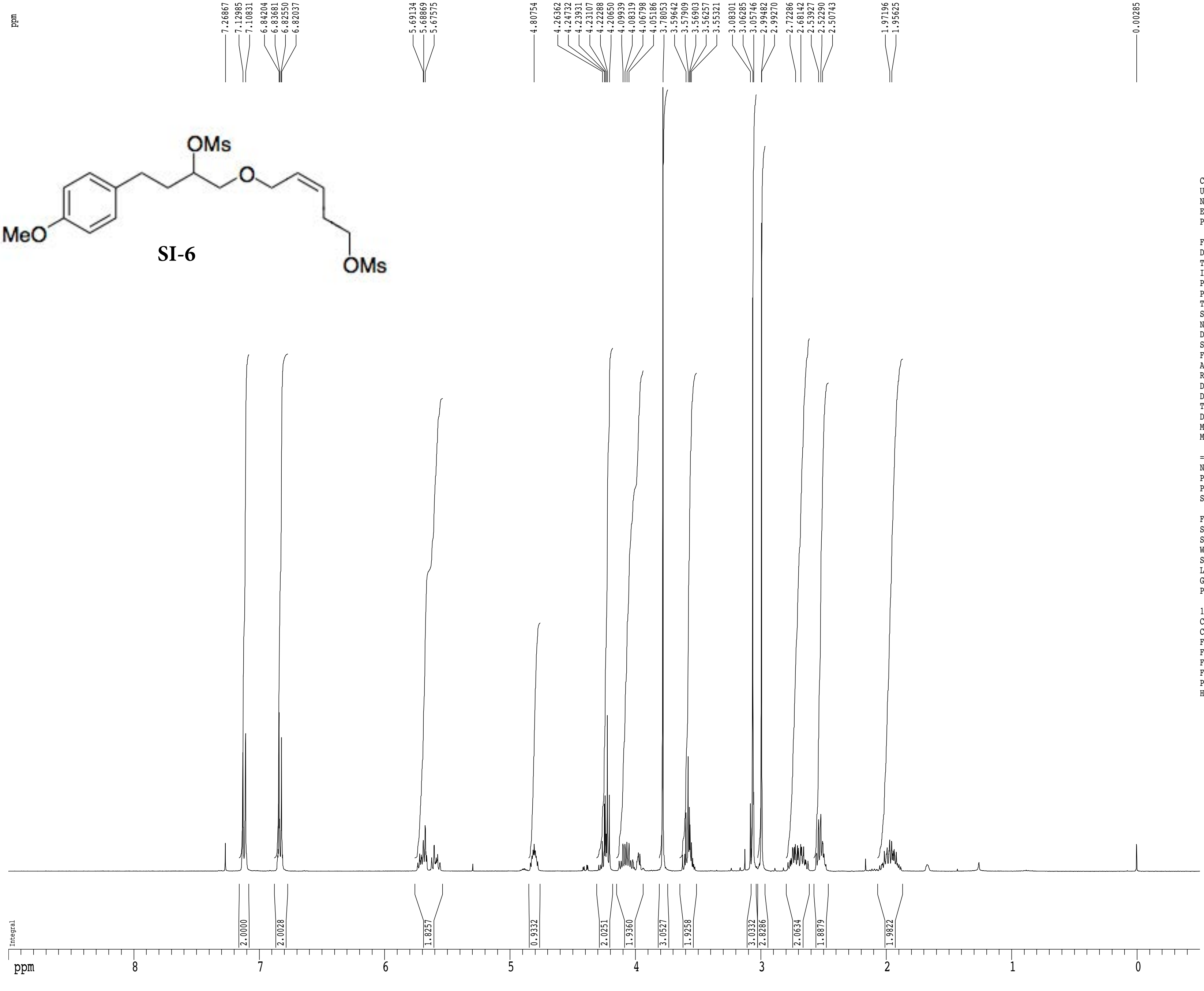


```
Current Data Parameters
USER            khewitt1
NAME         KAH-V-173-Z
EXPNO                  3
PROCNO                 1

F2 - Acquisition Parameters
Date_           20211112
Time               11.54
INSTRUM           drx400
PROBHD   5 mm QNP H/F/P
PULPROG             zg30
TD                 38460
SOLVENT           CDCl3T
NS                     8
DS                     2
SWH             6410.256 Hz
FIDRES          0.166673 Hz
AQ             2.9999299 sec
RG                  90.5
DW                78.000 usec
DE                  4.50 usec
TE                 298.0 K
D1            0.10000000 sec
MCREST        0.00000000 sec
MCWRK         0.01500000 sec

======== CHANNEL f1 ========
NUC1                  1H
P1                 12.00 usec
PL1                -0.90 dB
SFO1         400.1328009 MHz

F2 - Processing parameters
SI                 65536
SF           400.1300175 MHz
WDW                   no
SSB                    0
LB                  0.00 Hz
GB                     0
PC                  2.00

1D NMR plot parameters
CX                 22.80 cm
CY                 15.00 cm
F1P                9.000 ppm
F1               3601.17 Hz
F2P               -0.500 ppm
F2               -200.06 Hz
PPMCM            0.41667 ppm/cm
HZCM           166.72084 Hz/cm
```

13C spectrum with 1H decoupling

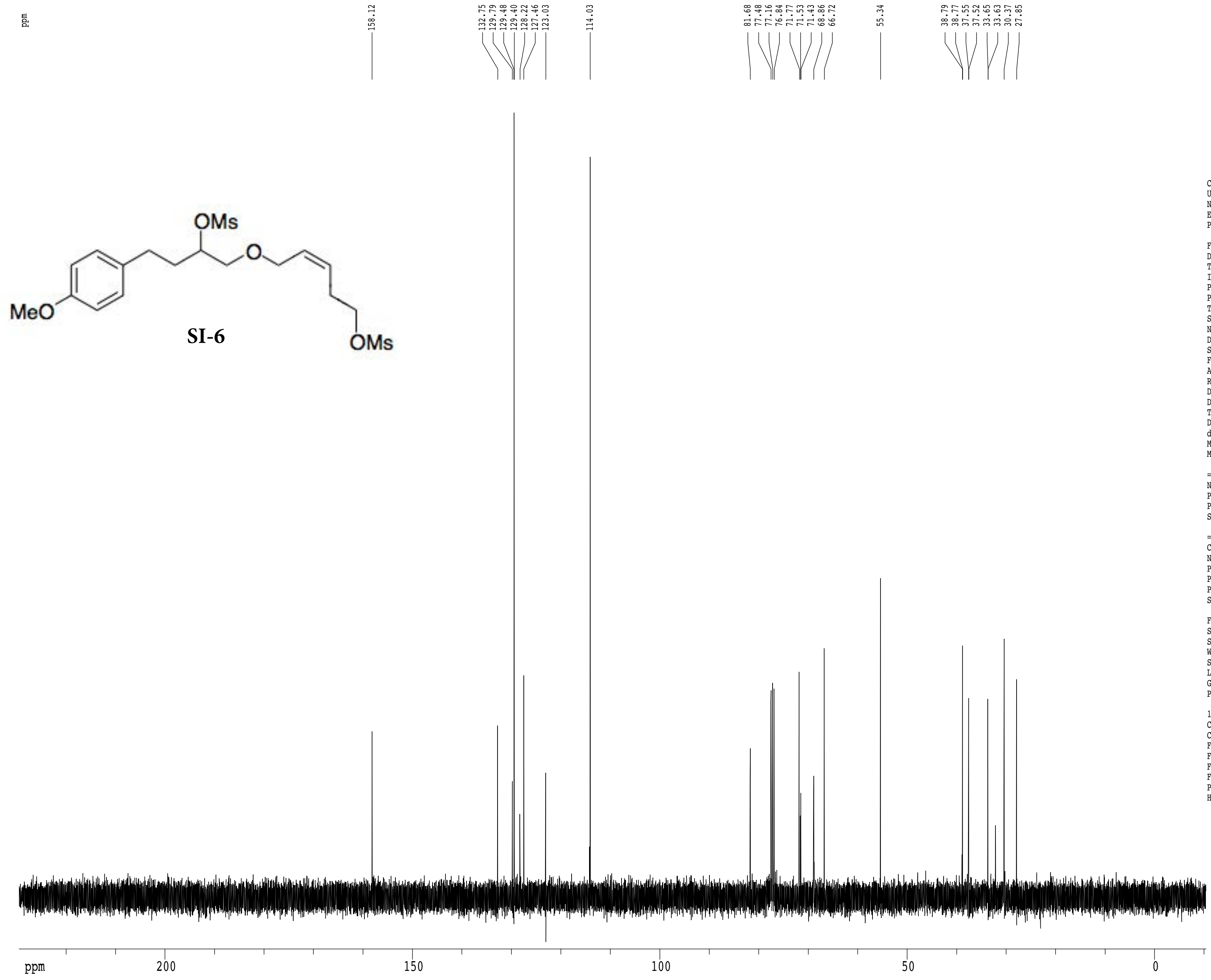

Current Data Parameters
USER khewitt1
NAME KAH-V-173-Z
EXPNO 4
PROCNO 1

F2 - Acquisition Parameters
Date_ 20211112
Time 11.57
INSTRUM drx400
PROBHD 5 mm QNP H/F/P
PULPROG zgdc30
TD 65536
SOLVENT CDCl3
NS 112
DS 4
SWH 24154.590 Hz
FIDRES 0.368570 Hz
AQ 1.3566452 sec
RG 14596.5
DW 20.700 usec
DE 20.39 usec
TE 298.0 K
D1 0.10000000 sec
d11 0.03000000 sec
MCREST 0.00000000 sec
MCWRK 0.01500000 sec

======== CHANNEL f1 ========
NUC1 13C
P1 7.90 usec
PL1 -3.00 dB
SFO1 100.6237964 MHz

======== CHANNEL f2 ========
CPDPRG2 waltz16
NUC2 1H
PCPD2 90.00 usec
PL2 -0.90 dB
PL12 17.00 dB
SFO2 400.1328009 MHz

F2 - Processing parameters
SI 65536
SF 100.6127650 MHz
WDW no
SSB 0
LB 0.00 Hz
GB 0
PC 1.00

1D NMR plot parameters
CX 22.80 cm
CY 15.50 cm
F1P 229.496 ppm
F1 23090.21 Hz
F2P -10.579 ppm
F2 -1064.37 Hz
PPMCM 10.52959 ppm/cm
HZCM 1059.41150 Hz/cm

1H spectrum

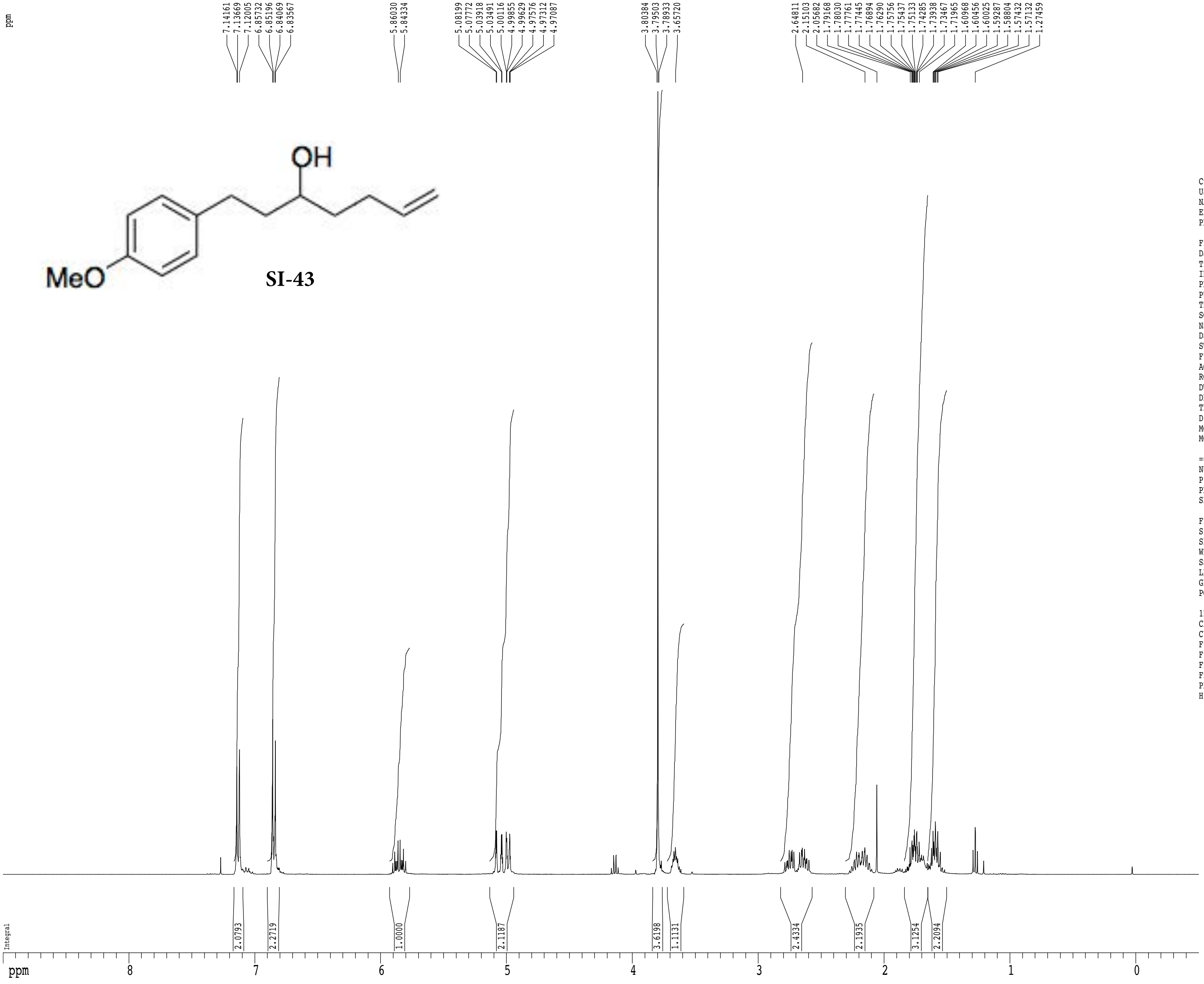

SI-43

```
Current Data Parameters
USER          khewitt1
NAME        KAH-V-248-1
EXPNO                1
PROCNO               1

F2 - Acquisition Parameters
Date_         20220126
Time             18.34
INSTRUM         drx400
PROBHD   5 mm QNP H/F/P
PULPROG           zg30
TD               38460
SOLVENT         CDCl3T
NS                   8
DS                   2
SWH           6410.256 Hz
FIDRES        0.166673 Hz
AQ           2.9999299 sec
RG                  57
DW              78.000 usec
DE                4.50 usec
TE               298.0 K
D1          0.10000000 sec
MCREST      0.00000000 sec
MCWRK       0.01500000 sec

======== CHANNEL f1 ========
NUC1                1H
P1               12.00 usec
PL1              -0.90 dB
SFO1       400.1328009 MHz

F2 - Processing parameters
SI               65536
SF         400.1300175 MHz
WDW                 no
SSB                  0
LB                0.00 Hz
GB                   0
PC                2.00

1D NMR plot parameters
CX               22.80 cm
CY               15.00 cm
F1P              9.000 ppm
F1             3601.17 Hz
F2P             -0.500 ppm
F2             -200.06 Hz
PPMCM          0.41667 ppm/cm
HZCM         166.72084 Hz/cm
```

13C spectrum with 1H decoupling

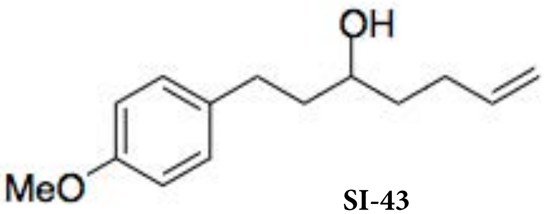

SI-43

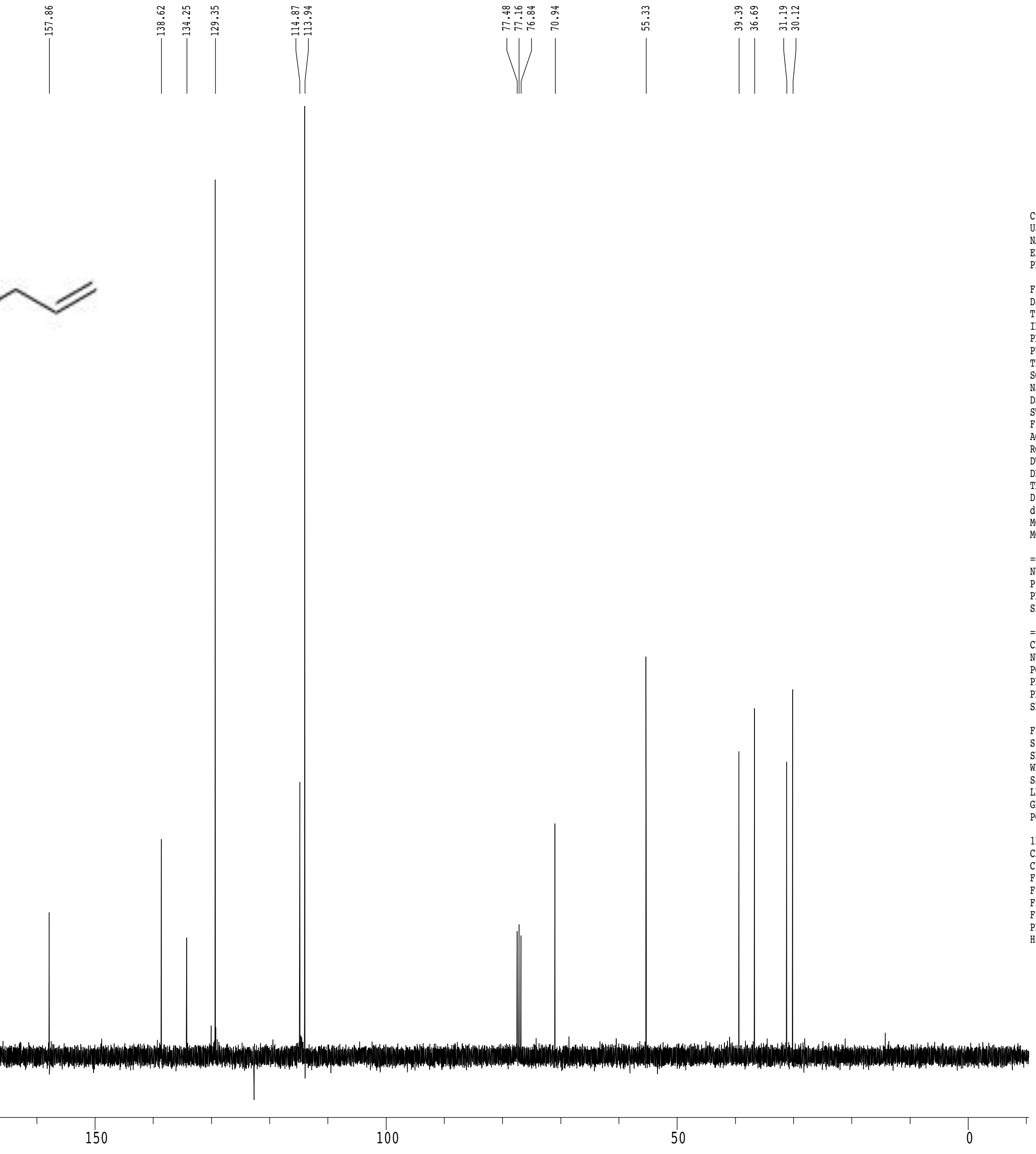


```
Current Data Parameters
USER          khewitt1
NAME        KAH-V-248-1
EXPNO              2
PROCNO             1

F2 - Acquisition Parameters
Date_          20220126
Time             18.37
INSTRUM         drx400
PROBHD   5 mm QNP H/F/P
PULPROG         zgdc30
TD               65536
SOLVENT          CDCl3
NS                  64
DS                   4
SWH          24154.590 Hz
FIDRES        0.368570 Hz
AQ           1.3566452 sec
RG              9195.2
DW              20.700 usec
DE               20.39 usec
TE               298.0 K
D1          0.10000000 sec
d11         0.03000000 sec
MCREST      0.00000000 sec
MCWRK       0.01500000 sec

======== CHANNEL f1 ========
NUC1               13C
P1                7.90 usec
PL1              -3.00 dB
SFO1       100.6237964 MHz

======== CHANNEL f2 ========
CPDPRG2         waltz16
NUC2                1H
PCPD2            90.00 usec
PL2              -0.90 dB
PL12             17.00 dB
SFO2       400.1328009 MHz

F2 - Processing parameters
SI               65536
SF         100.6127658 MHz
WDW                 no
SSB                  0
LB                0.00 Hz
GB                   0
PC                1.00

1D NMR plot parameters
CX               22.80 cm
CY               15.50 cm
F1P            229.496 ppm
F1            23090.21 Hz
F2P            -10.579 ppm
F2            -1064.37 Hz
PPMCM         10.52959 ppm/cm
HZCM        1059.41150 Hz/cm
```

1H spectrum

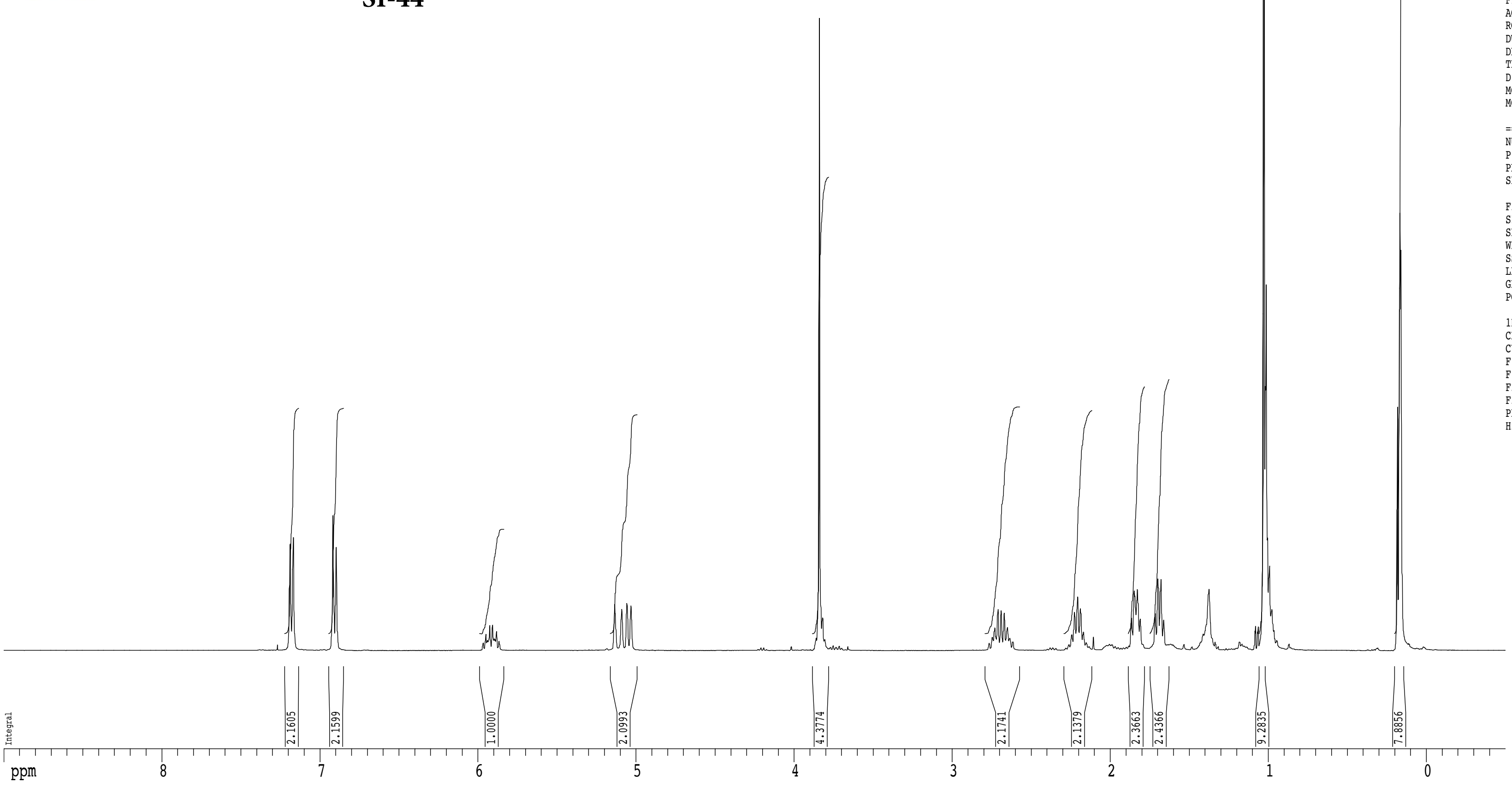

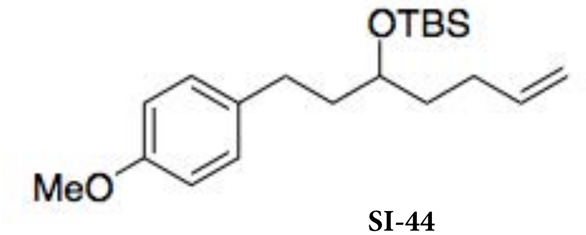

SI-44

Current Data Parameters
USER khewitt1
NAME KAH-V-159-1
EXPNO 1
PROCNO 1

F2 - Acquisition Parameters
Date_ 20211028
Time 18.42
INSTRUM drx400
PROBHD 5 mm QNP H/F/P
PULPROG zg30
TD 38460
SOLVENT CDCl3T
NS 8
DS 2
SWH 6410.256 Hz
FIDRES 0.166673 Hz
AQ 2.9999299 sec
RG 11.3
DW 78.000 usec
DE 4.50 usec
TE 298.0 K
D1 0.10000000 sec
MCREST 0.00000000 sec
MCWRK 0.01500000 sec

======== CHANNEL f1 ========
NUC1 1H
P1 12.00 usec
PL1 -0.90 dB
SFO1 400.1328009 MHz

F2 - Processing parameters
SI 65536
SF 400.1300175 MHz
WDW no
SSB 0
LB 0.00 Hz
GB 0
PC 2.00

1D NMR plot parameters
CX 22.80 cm
CY 15.00 cm
F1P 9.000 ppm
F1 3601.17 Hz
F2P -0.500 ppm
F2 -200.06 Hz
PPMCM 0.41667 ppm/cm
HZCM 166.72084 Hz/cm

13C spectrum with 1H decoupling

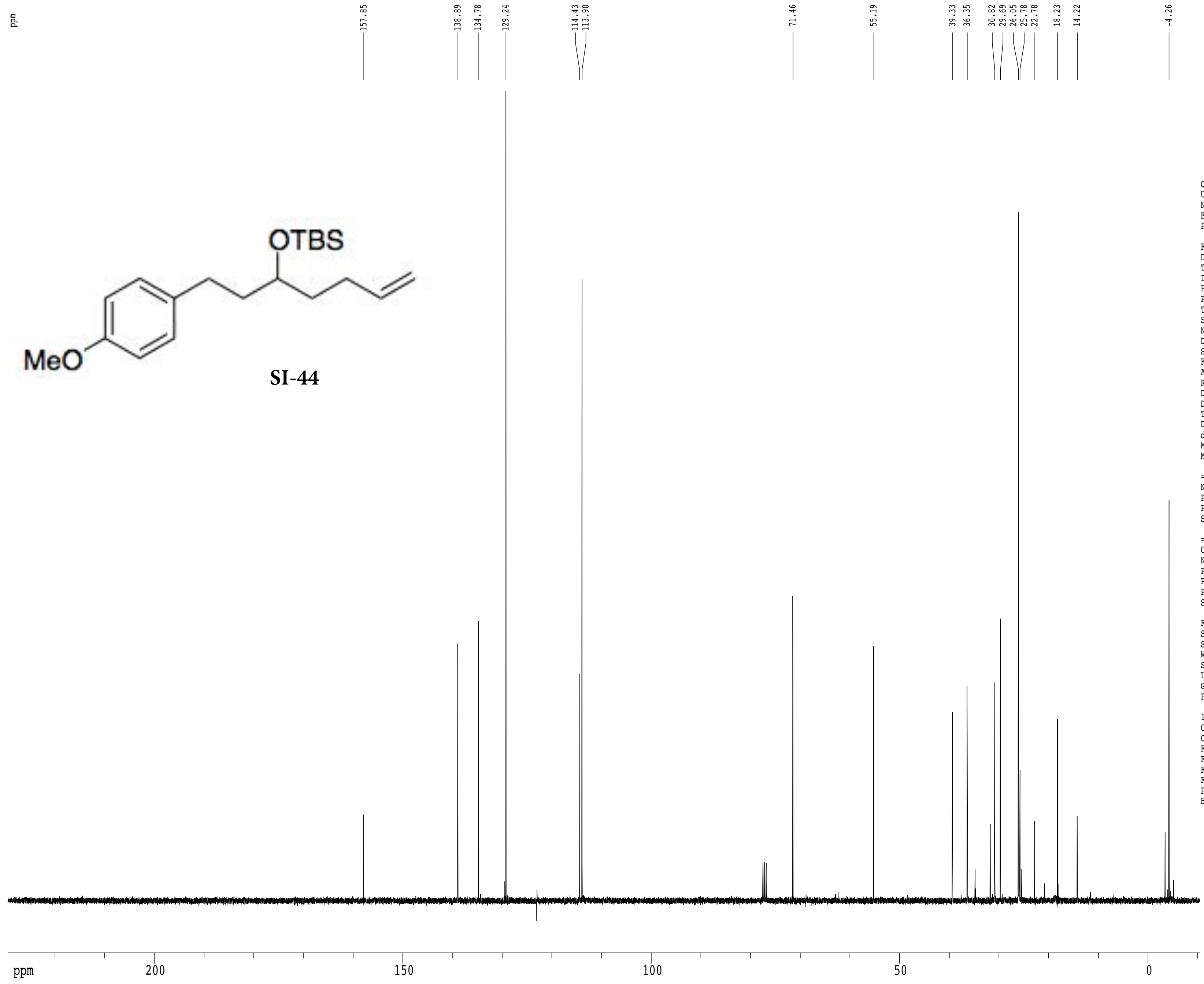

Current Data Parameters
USER khewitt1
NAME KAH-V-159-1
EXPNO 2
PROCNO 1

F2 - Acquisition Parameters
Date_ 20211028
Time 18.44
INSTRUM drx400
PROBHD 5 mm QNP H/F/P
PULPROG zgdc30
TD 65536
SOLVENT CDCl3
NS 80
DS 4
SWH 24154.590 Hz
FIDRES 0.368570 Hz
AQ 1.3566452 sec
RG 9195.2
DW 20.700 usec
DE 20.39 usec
TE 298.1 K
D1 0.10000000 sec
d11 0.03000000 sec
MCREST 0.00000000 sec
MCWRK 0.01500000 sec

======== CHANNEL f1 ========
NUC1 13C
P1 7.90 usec
PL1 -3.00 dB
SFO1 100.6237964 MHz

======== CHANNEL f2 ========
CPDPRG2 waltz16
NUC2 1H
PCPD2 90.00 usec
PL2 -0.90 dB
PL12 17.00 dB
SFO2 400.1328009 MHz

F2 - Processing parameters
SI 65536
SF 100.6127680 MHz
WDW no
SSB 0
LB 0.00 Hz
GB 0
PC 1.00

1D NMR plot parameters
CX 22.80 cm
CY 15.50 cm
F1P 229.496 ppm
F1 23090.22 Hz
F2P -10.579 ppm
F2 -1064.37 Hz
PPMCM 10.52959 ppm/cm
HZCM 1059.41162 Hz/cm

1H spectrum

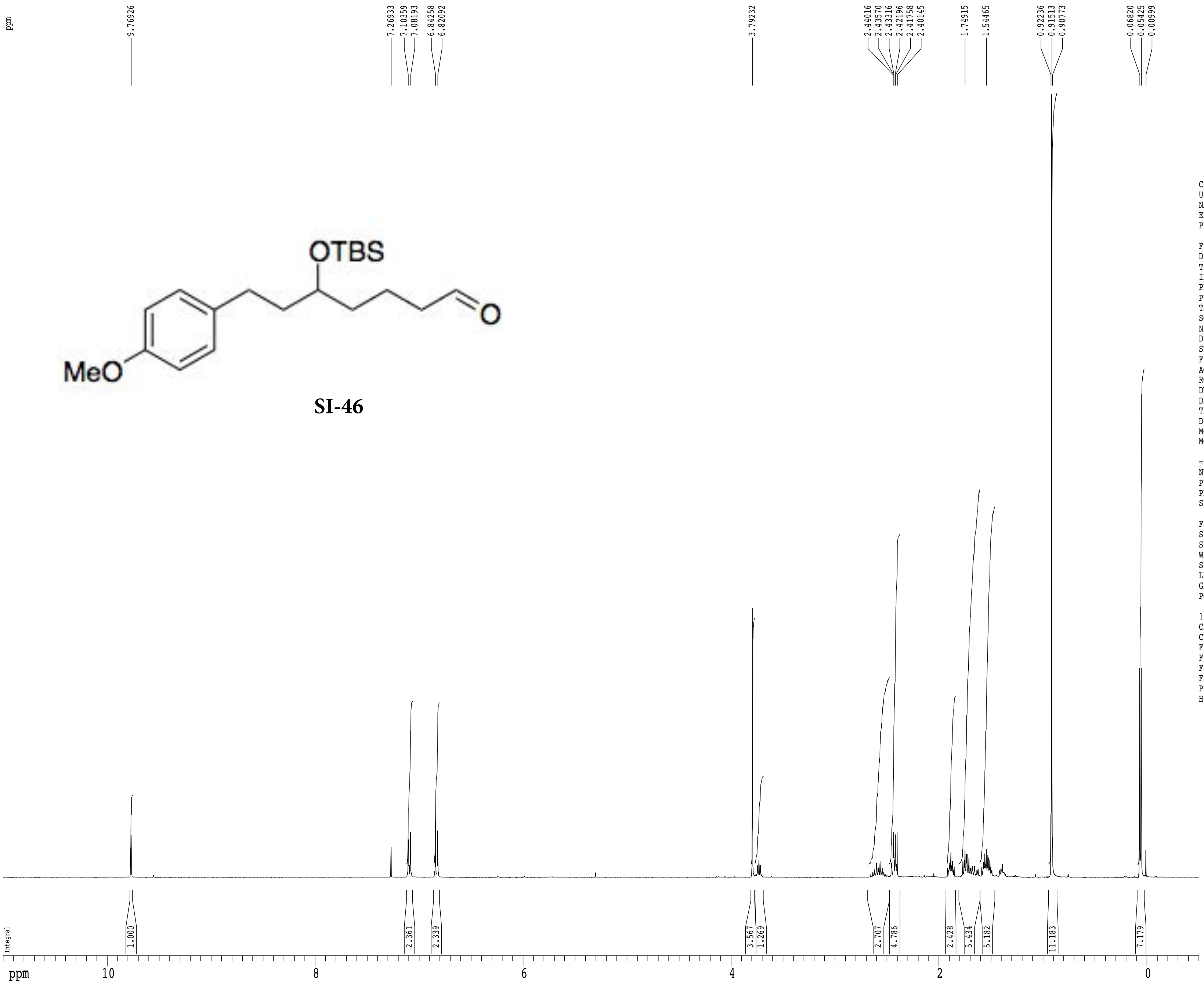

SI-46

```
Current Data Parameters
USER            khewitt1
NAME         KAH-V-258-1
EXPNO                  1
PROCNO                 1

F2 - Acquisition Parameters
Date_           20220202
Time               13.52
INSTRUM           drx400
PROBHD   5 mm QNP H/F/P
PULPROG             zg30
TD                 38460
SOLVENT           CDCl3T
NS                     8
DS                     2
SWH             6410.256 Hz
FIDRES          0.166673 Hz
AQ             2.9999299 sec
RG                   114
DW                78.000 usec
DE                  4.50 usec
TE                 298.0 K
D1            0.10000000 sec
MCREST        0.00000000 sec
MCWRK         0.01500000 sec

======== CHANNEL f1 ========
NUC1                  1H
P1                 12.00 usec
PL1                -0.90 dB
SFO1         400.1328009 MHz

F2 - Processing parameters
SI                 65536
SF           400.1300175 MHz
WDW                   no
SSB                    0
LB                  0.00 Hz
GB                     0
PC                  2.00

1D NMR plot parameters
CX                 22.80 cm
CY                 15.00 cm
F1P               11.000 ppm
F1               4401.43 Hz
F2P               -0.500 ppm
F2               -200.07 Hz
PPMCM            0.50439 ppm/cm
HZCM           201.81996 Hz/cm
```

13C spectrum with 1H decoupling

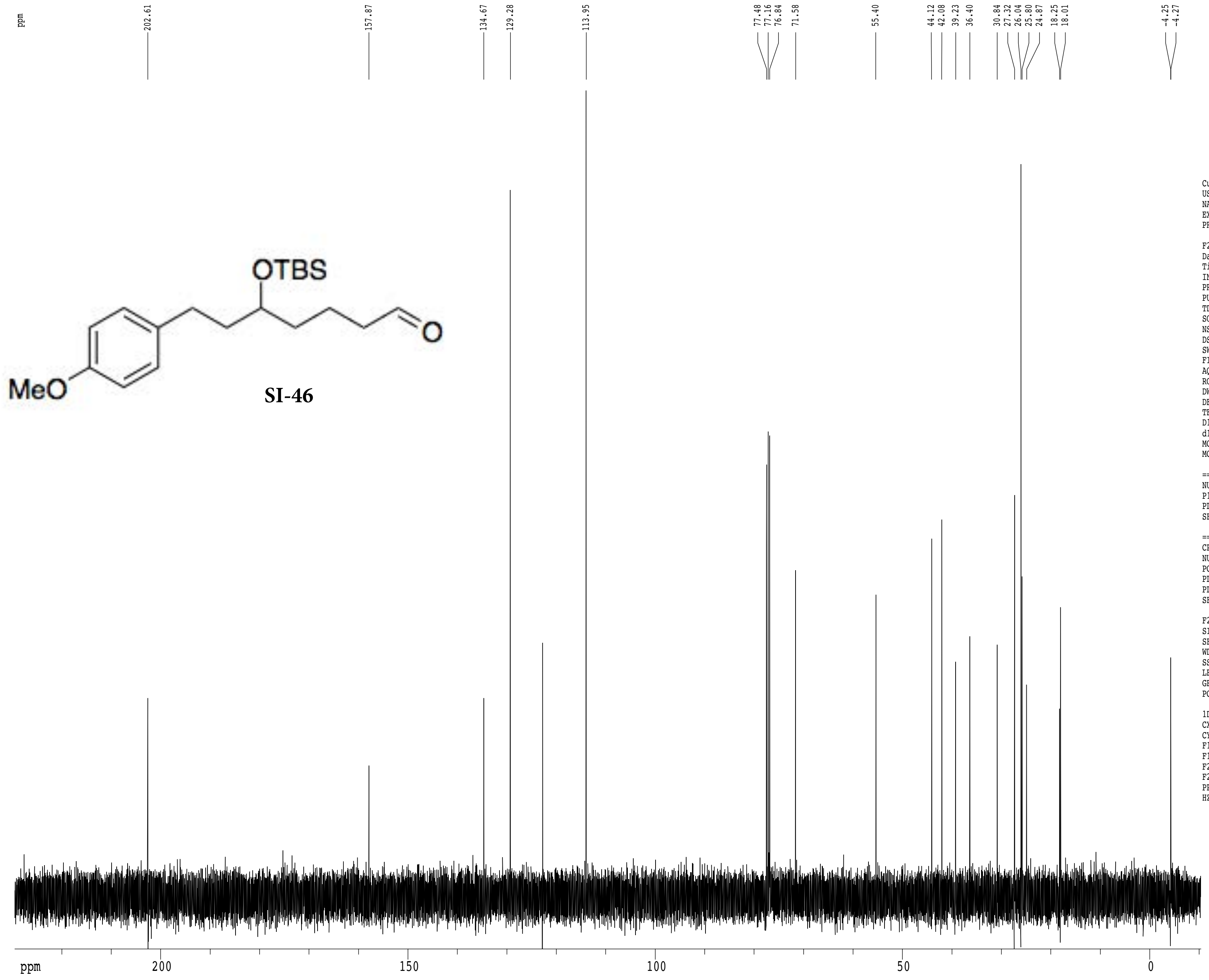

SI-46

```
Current Data Parameters
USER              khewitt1
NAME           KAH-V-258-1
EXPNO                    2
PROCNO                   1

F2 - Acquisition Parameters
Date_             20220202
Time                 13.56
INSTRUM             drx400
PROBHD   5 mm QNP H/F/P
PULPROG            zgdc30
TD                   65536
SOLVENT              CDCl3
NS                      96
DS                       4
SWH          24154.590 Hz
FIDRES        0.368570 Hz
AQ           1.3566452 sec
RG                 10321.3
DW              20.700 usec
DE               20.39 usec
TE                 298.0 K
D1          0.10000000 sec
d11         0.03000000 sec
MCREST      0.00000000 sec
MCWRK       0.01500000 sec

======== CHANNEL f1 ========
NUC1                   13C
P1                7.90 usec
PL1               -3.00 dB
SFO1        100.6237964 MHz

======== CHANNEL f2 ========
CPDPRG2            waltz16
NUC2                    1H
PCPD2            90.00 usec
PL2               -0.90 dB
PL12              17.00 dB
SFO2        400.1328009 MHz

F2 - Processing parameters
SI                   65536
SF          100.6127584 MHz
WDW                     no
SSB                      0
LB                 0.00 Hz
GB                       0
PC                    1.00

1D NMR plot parameters
CX                22.80 cm
CY                15.50 cm
F1P             229.496 ppm
F1             23090.21 Hz
F2P             -10.579 ppm
F2             -1064.37 Hz
PPMCM         10.52959 ppm/cm
HZCM        1059.41150 Hz/cm
```

1H spectrum

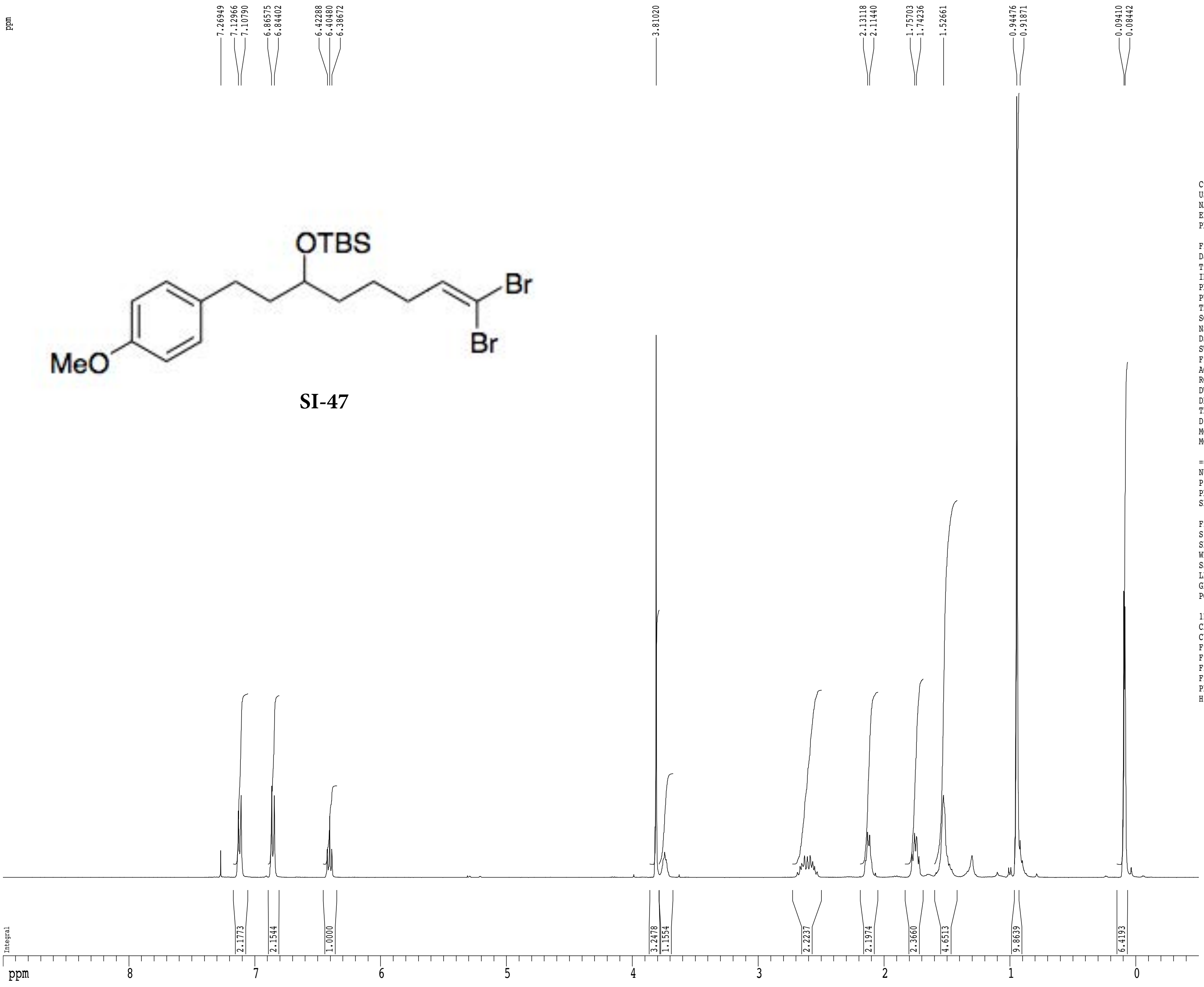

SI-47

```
Current Data Parameters
USER            khewitt1
NAME       KAH-V-262-c3
EXPNO                 1
PROCNO                1

F2 - Acquisition Parameters
Date_          20220203
Time              17.53
INSTRUM          drx400
PROBHD   5 mm QNP H/F/P
PULPROG            zg30
TD                38460
SOLVENT          CDCl3T
NS                    8
DS                    2
SWH            6410.256 Hz
FIDRES         0.166673 Hz
AQ            2.9999299 sec
RG                   57
DW               78.000 usec
DE                 4.50 usec
TE                298.0 K
D1           0.10000000 sec
MCREST       0.00000000 sec
MCWRK        0.01500000 sec

======== CHANNEL f1 ========
NUC1                 1H
P1                12.00 usec
PL1               -0.90 dB
SFO1        400.1328009 MHz

F2 - Processing parameters
SI                65536
SF          400.1300175 MHz
WDW                  no
SSB                   0
LB                 0.00 Hz
GB                    0
PC                 2.00

1D NMR plot parameters
CX                22.80 cm
CY                15.00 cm
F1P               9.000 ppm
F1              3601.17 Hz
F2P              -0.500 ppm
F2              -200.06 Hz
PPMCM           0.41667 ppm/cm
HZCM          166.72084 Hz/cm
```

13C spectrum with 1H decoupling

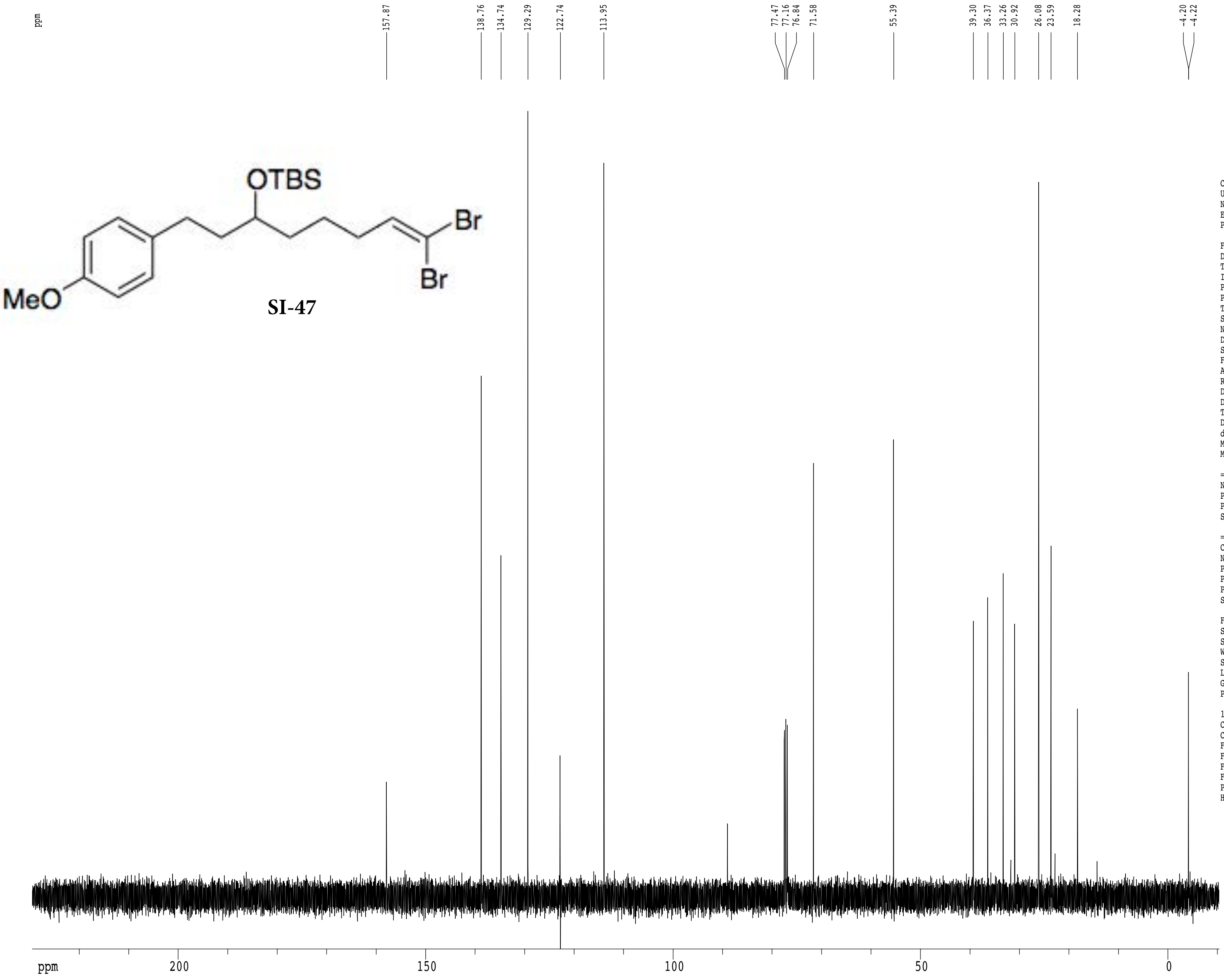


```
Current Data Parameters
USER            khewitt1
NAME        KAH-V-262-c3
EXPNO                  2
PROCNO                 1

F2 - Acquisition Parameters
Date_           20220203
Time               17.55
INSTRUM           drx400
PROBHD   5 mm QNP H/F/P
PULPROG           zgdc30
TD                 65536
SOLVENT            CDCl3
NS                    96
DS                     4
SWH         24154.590 Hz
FIDRES       0.368570 Hz
AQ          1.3566452 sec
RG                9195.2
DW            20.700 usec
DE             20.39 usec
TE             298.0 K
D1        0.10000000 sec
d11       0.03000000 sec
MCREST    0.00000000 sec
MCWRK     0.01500000 sec

======== CHANNEL f1 ========
NUC1                 13C
P1              7.90 usec
PL1             -3.00 dB
SFO1      100.6237964 MHz

======== CHANNEL f2 ========
CPDPRG2          waltz16
NUC2                  1H
PCPD2          90.00 usec
PL2             -0.90 dB
PL12            17.00 dB
SFO2      400.1328009 MHz

F2 - Processing parameters
SI                 65536
SF        100.6127602 MHz
WDW                   no
SSB                    0
LB               0.00 Hz
GB                     0
PC                  1.00

1D NMR plot parameters
CX              22.80 cm
CY              15.50 cm
F1P           229.496 ppm
F1           23090.21 Hz
F2P           -10.579 ppm
F2           -1064.37 Hz
PPMCM      10.52959 ppm/cm
HZCM     1059.41150 Hz/cm
```

1H spectrum

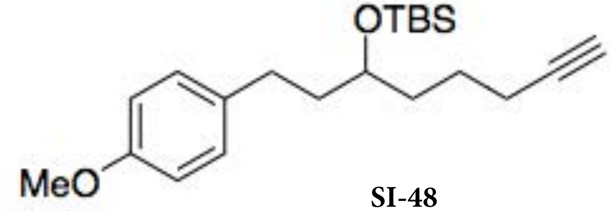

SI-48

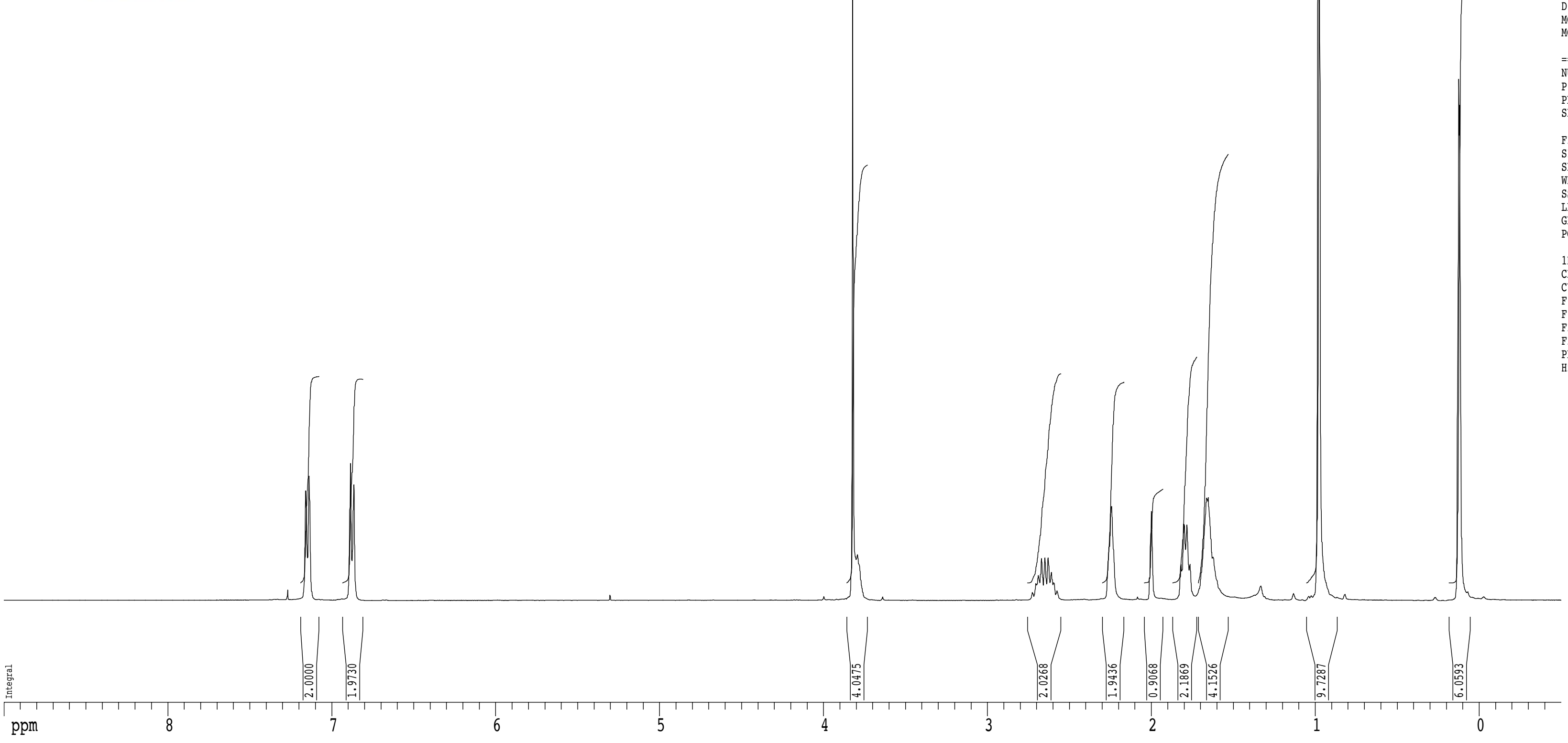


```
Current Data Parameters
USER              khewitt1
NAME           KAH-V-185-1
EXPNO                    1
PROCNO                   1

F2 - Acquisition Parameters
Date_             20211202
Time                 17.05
INSTRUM             drx400
PROBHD   5 mm QNP H/F/P
PULPROG               zg30
TD                   38460
SOLVENT              CDCl3T
NS                       8
DS                       2
SWH               6410.256 Hz
FIDRES            0.166673 Hz
AQ               2.9999299 sec
RG                      16
DW                  78.000 usec
DE                    4.50 usec
TE                   298.0 K
D1              0.10000000 sec
MCREST          0.00000000 sec
MCWRK           0.01500000 sec

======== CHANNEL f1 ========
NUC1                    1H
P1                   12.00 usec
PL1                  -0.90 dB
SFO1         400.1328009 MHz

F2 - Processing parameters
SI                   65536
SF           400.1300175 MHz
WDW                     no
SSB                      0
LB                    0.00 Hz
GB                       0
PC                    2.00

1D NMR plot parameters
CX                   22.80 cm
CY                   15.00 cm
F1P                  9.000 ppm
F1                 3601.17 Hz
F2P                 -0.500 ppm
F2                 -200.06 Hz
PPMCM              0.41667 ppm/cm
HZCM             166.72084 Hz/cm
```

13C spectrum with 1H decoupling

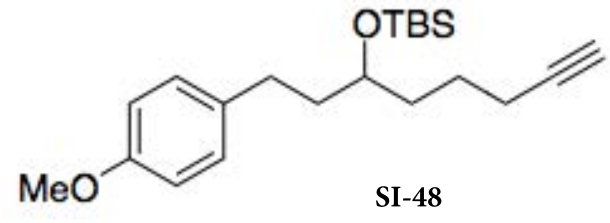

SI-48

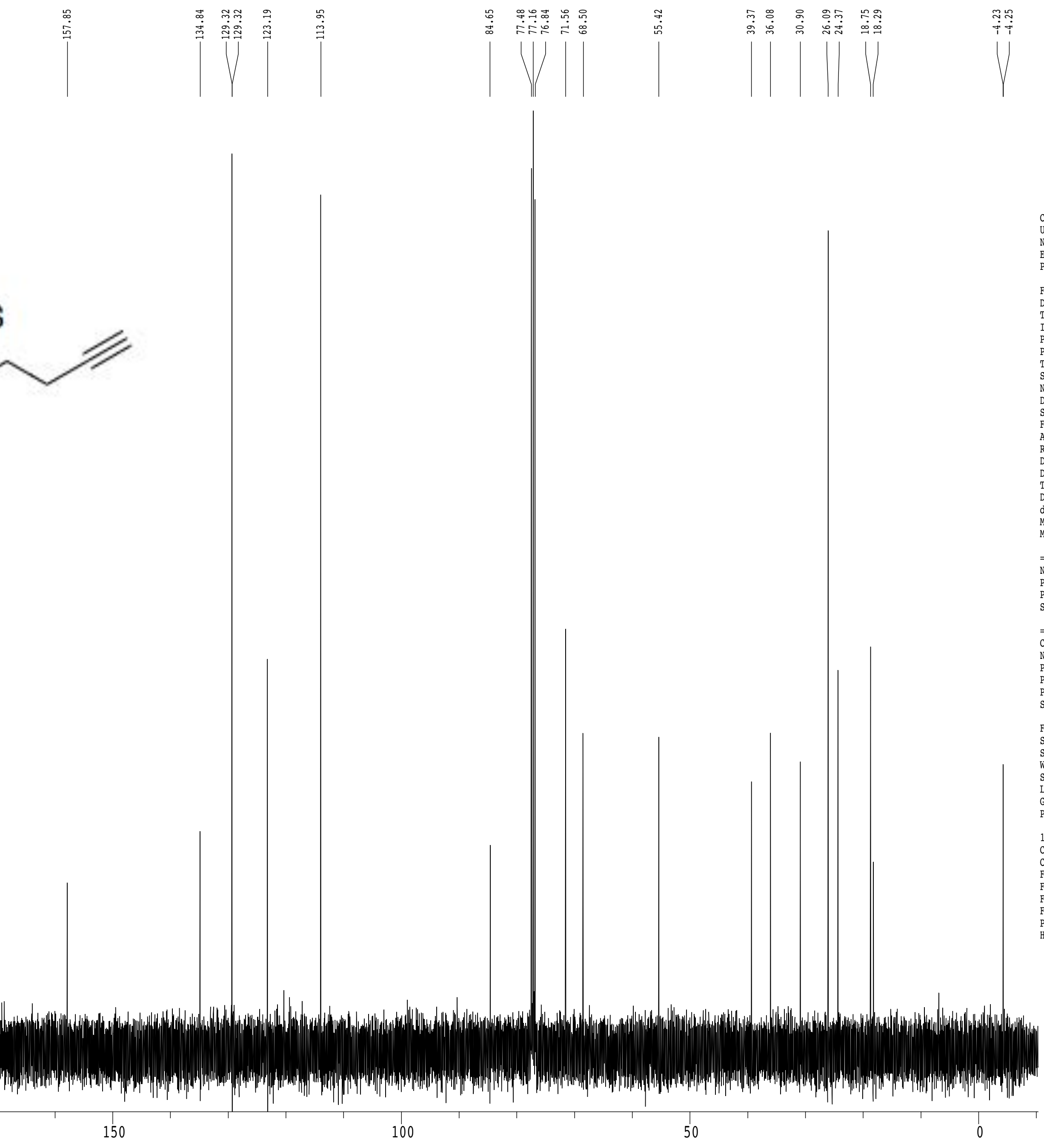


```
Current Data Parameters
USER            khewitt1
NAME         KAH-V-175-1
EXPNO                  2
PROCNO                 1

F2 - Acquisition Parameters
Date_           20211122
Time               16.48
INSTRUM           drx400
PROBHD   5 mm QNP H/F/P
PULPROG          zgdc30
TD                 65536
SOLVENT            CDCl3
NS                   224
DS                     4
SWH         24154.590 Hz
FIDRES       0.368570 Hz
AQ         1.3566452 sec
RG                9195.2
DW            20.700 usec
DE             20.39 usec
TE               298.0 K
D1        0.10000000 sec
d11       0.03000000 sec
MCREST    0.00000000 sec
MCWRK     0.01500000 sec

======== CHANNEL f1 ========
NUC1                 13C
P1               7.90 usec
PL1              -3.00 dB
SFO1       100.6237964 MHz

======== CHANNEL f2 ========
CPDPRG2          waltz16
NUC2                  1H
PCPD2           90.00 usec
PL2              -0.90 dB
PL12             17.00 dB
SFO2       400.1328009 MHz

F2 - Processing parameters
SI                 65536
SF         100.6127569 MHz
WDW                   no
SSB                    0
LB               0.00 Hz
GB                     0
PC                  1.00

1D NMR plot parameters
CX               22.80 cm
CY               15.50 cm
F1P            229.496 ppm
F1            23090.21 Hz
F2P            -10.579 ppm
F2            -1064.37 Hz
PPMCM        10.52959 ppm/cm
HZCM       1059.41150 Hz/cm
```

1H spectrum

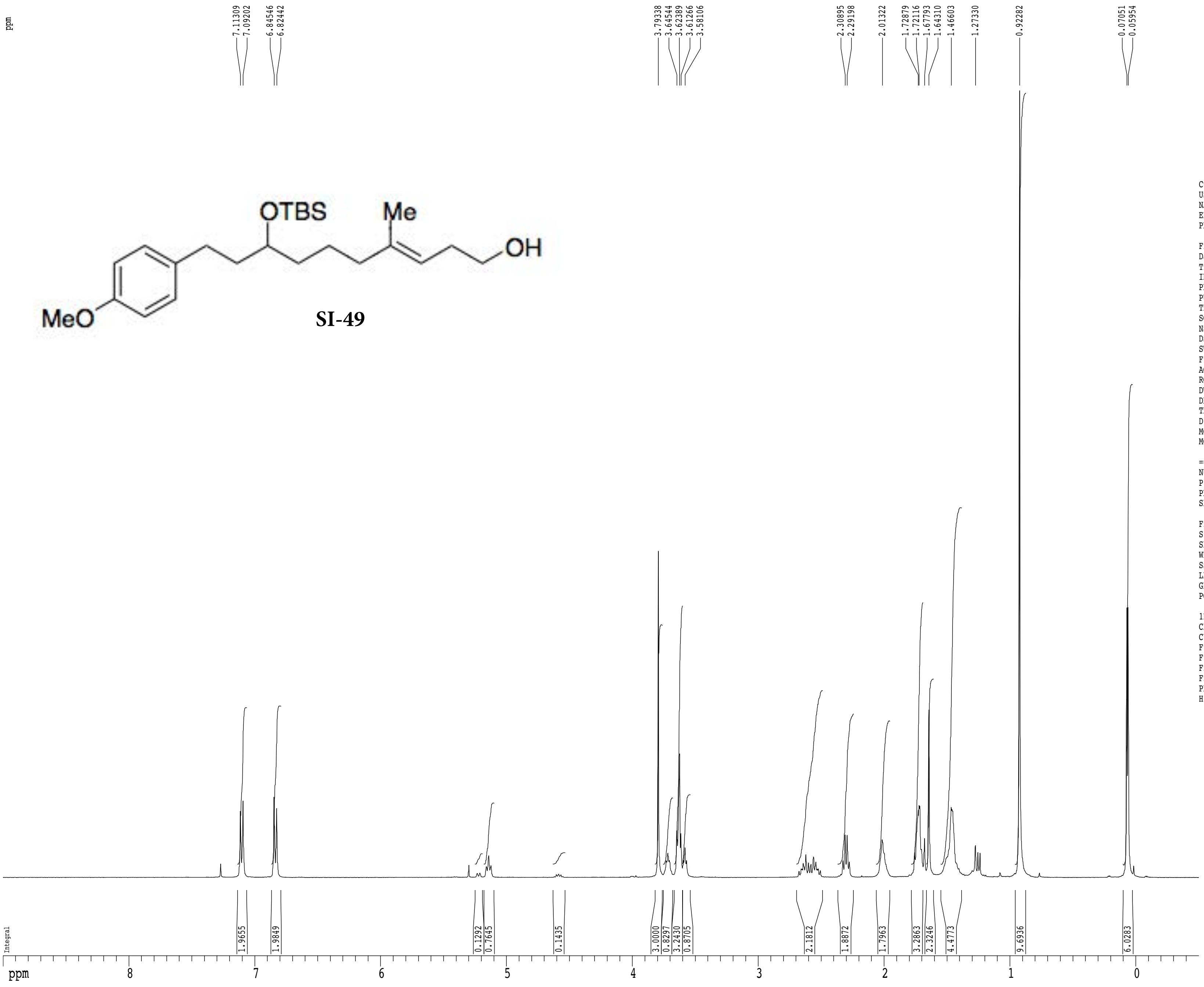


```
Current Data Parameters
USER            khewitt1
NAME       KAH-V-193-chk
EXPNO                  1
PROCNO                 1

F2 - Acquisition Parameters
Date_           20211209
Time               16.41
INSTRUM           drx400
PROBHD   5 mm QNP H/F/P
PULPROG             zg30
TD                 38460
SOLVENT           CDCl3T
NS                     8
DS                     2
SWH             6410.256 Hz
FIDRES          0.166673 Hz
AQ             2.9999299 sec
RG                  50.8
DW                78.000 usec
DE                  4.50 usec
TE                 298.0 K
D1            0.10000000 sec
MCREST        0.00000000 sec
MCWRK         0.01500000 sec

======== CHANNEL f1 ========
NUC1                  1H
P1                 12.00 usec
PL1                -0.90 dB
SFO1         400.1328009 MHz

F2 - Processing parameters
SI                 65536
SF           400.1300175 MHz
WDW                   no
SSB                    0
LB                  0.00 Hz
GB                     0
PC                  2.00

1D NMR plot parameters
CX                 22.80 cm
CY                 15.00 cm
F1P                9.000 ppm
F1               3601.17 Hz
F2P               -0.500 ppm
F2               -200.06 Hz
PPMCM            0.41667 ppm/cm
HZCM           166.72084 Hz/cm
```

13C spectrum with 1H decoupling

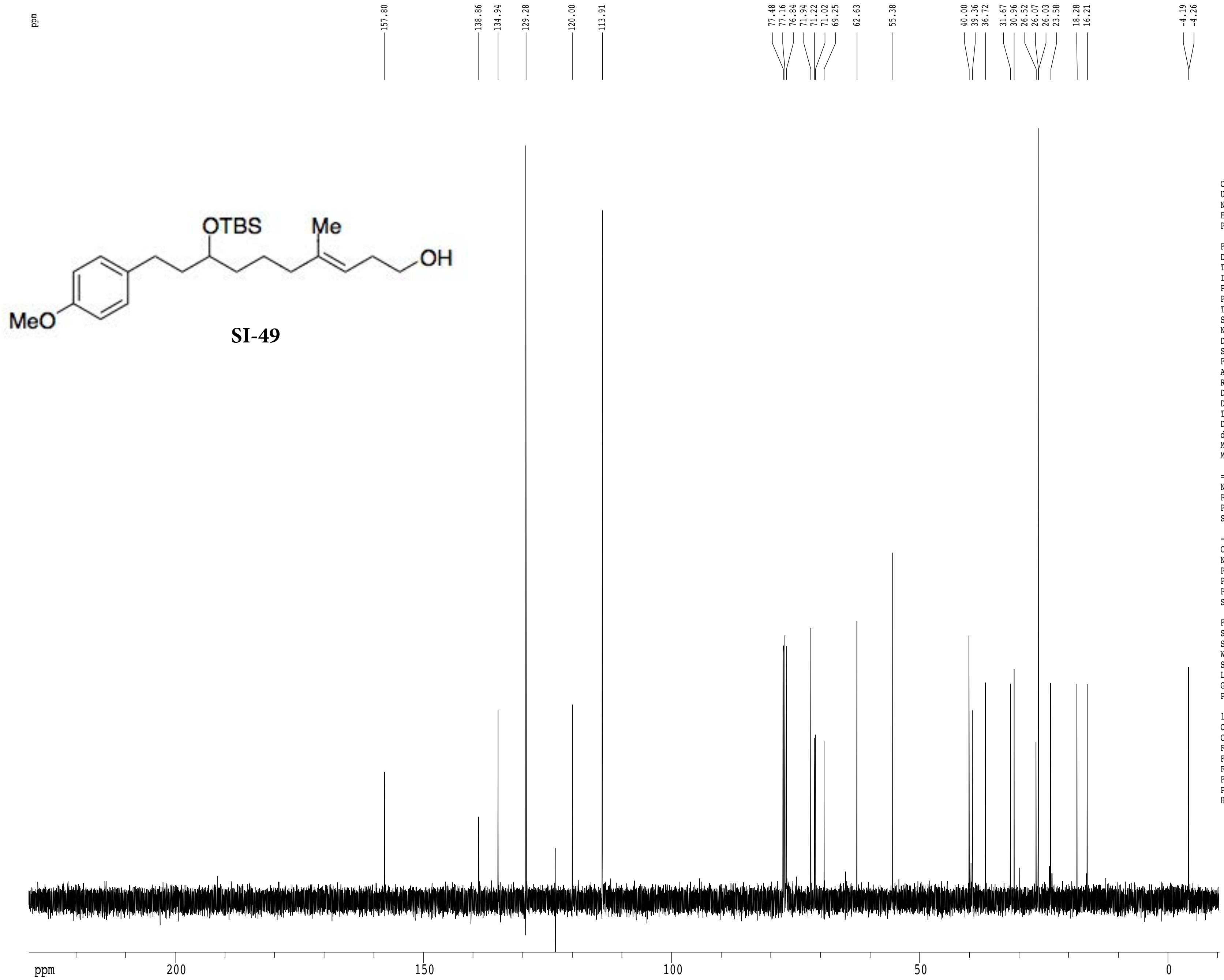

SI-49


```
Current Data Parameters
USER          khewitt1
NAME      KAH-V-193-chk
EXPNO                2
PROCNO               1

F2 - Acquisition Parameters
Date_         20211209
Time             16.47
INSTRUM         drx400
PROBHD   5 mm QNP H/F/P
PULPROG         zgdc30
TD               65536
SOLVENT          CDCl3
NS                 200
DS                   4
SWH          24154.590 Hz
FIDRES        0.368570 Hz
AQ           1.3566452 sec
RG              9195.2
DW              20.700 usec
DE               20.39 usec
TE               298.0 K
D1          0.10000000 sec
d11         0.03000000 sec
MCREST      0.00000000 sec
MCWRK       0.01500000 sec

======== CHANNEL f1 ========
NUC1               13C
P1                7.90 usec
PL1              -3.00 dB
SFO1       100.6237964 MHz

======== CHANNEL f2 ========
CPDPRG2        waltz16
NUC2                1H
PCPD2            90.00 usec
PL2              -0.90 dB
PL12             17.00 dB
SFO2       400.1328009 MHz

F2 - Processing parameters
SI               65536
SF         100.6127599 MHz
WDW                 no
SSB                  0
LB                0.00 Hz
GB                   0
PC                1.00

1D NMR plot parameters
CX               22.80 cm
CY               15.50 cm
F1P            229.496 ppm
F1            23090.21 Hz
F2P            -10.579 ppm
F2            -1064.37 Hz
PPMCM         10.52959 ppm/cm
HZCM        1059.41150 Hz/cm
```

1H spectrum

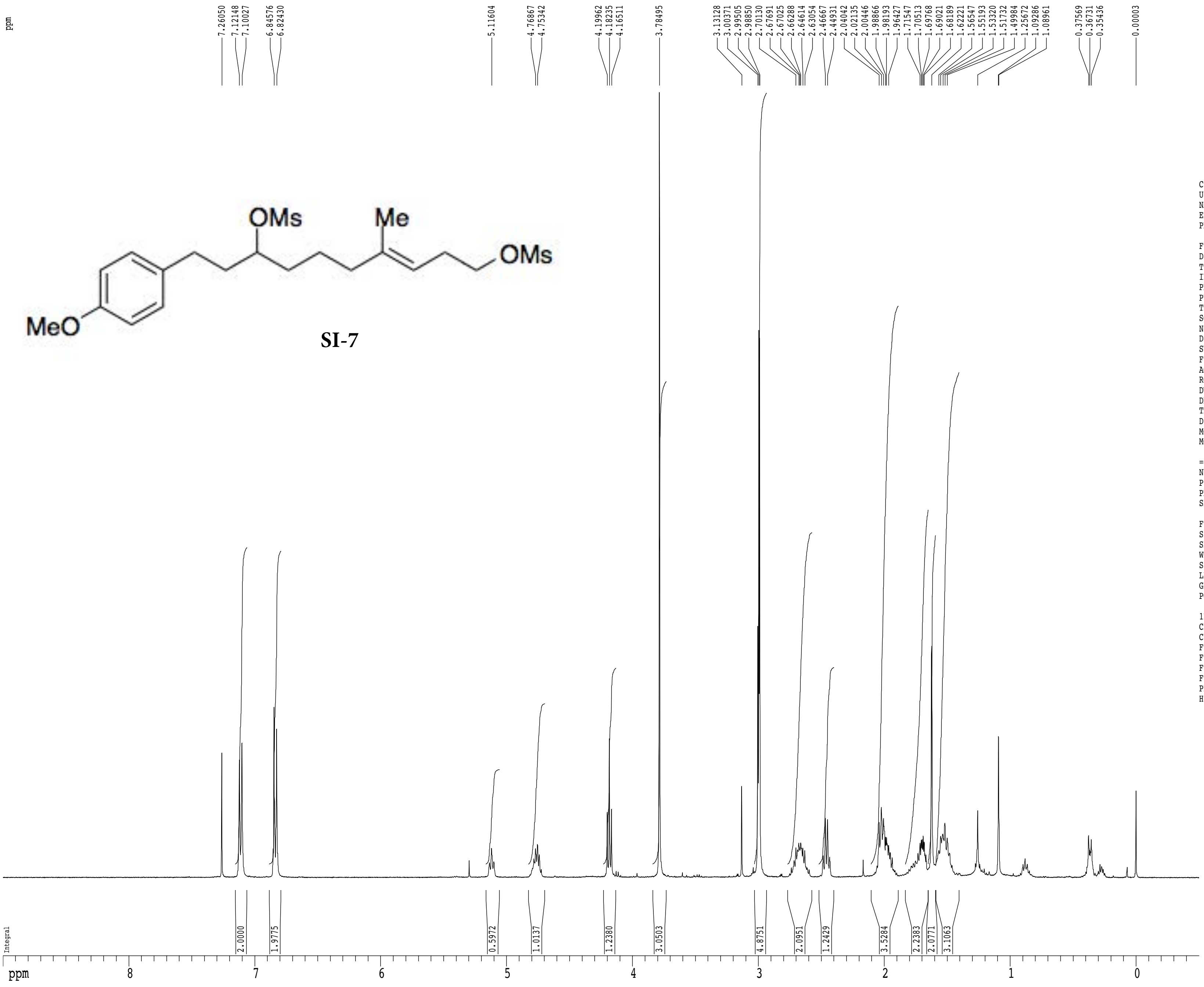

SI-7

```
Current Data Parameters
USER            khewitt1
NAME       KAH-V-203-chk
EXPNO                  1
PROCNO                 1

F2 - Acquisition Parameters
Date_           20211215
Time               16.58
INSTRUM           drx400
PROBHD   5 mm QNP H/F/P
PULPROG             zg30
TD                 38460
SOLVENT          CDCl3T
NS                     8
DS                     2
SWH          6410.256 Hz
FIDRES       0.166673 Hz
AQ          2.9999299 sec
RG                   181
DW            78.000 usec
DE              4.50 usec
TE               298.0 K
D1        0.10000000 sec
MCREST    0.00000000 sec
MCWRK     0.01500000 sec

======== CHANNEL f1 ========
NUC1                  1H
P1             12.00 usec
PL1              -0.90 dB
SFO1     400.1328009 MHz

F2 - Processing parameters
SI                 65536
SF        400.1300211 MHz
WDW                   no
SSB                    0
LB               0.00 Hz
GB                     0
PC                  2.00

1D NMR plot parameters
CX              22.80 cm
CY              15.00 cm
F1P             9.000 ppm
F1            3601.17 Hz
F2P            -0.500 ppm
F2            -200.06 Hz
PPMCM       0.41667 ppm/cm
HZCM       166.72086 Hz/cm
```

13C spectrum with 1H decoupling

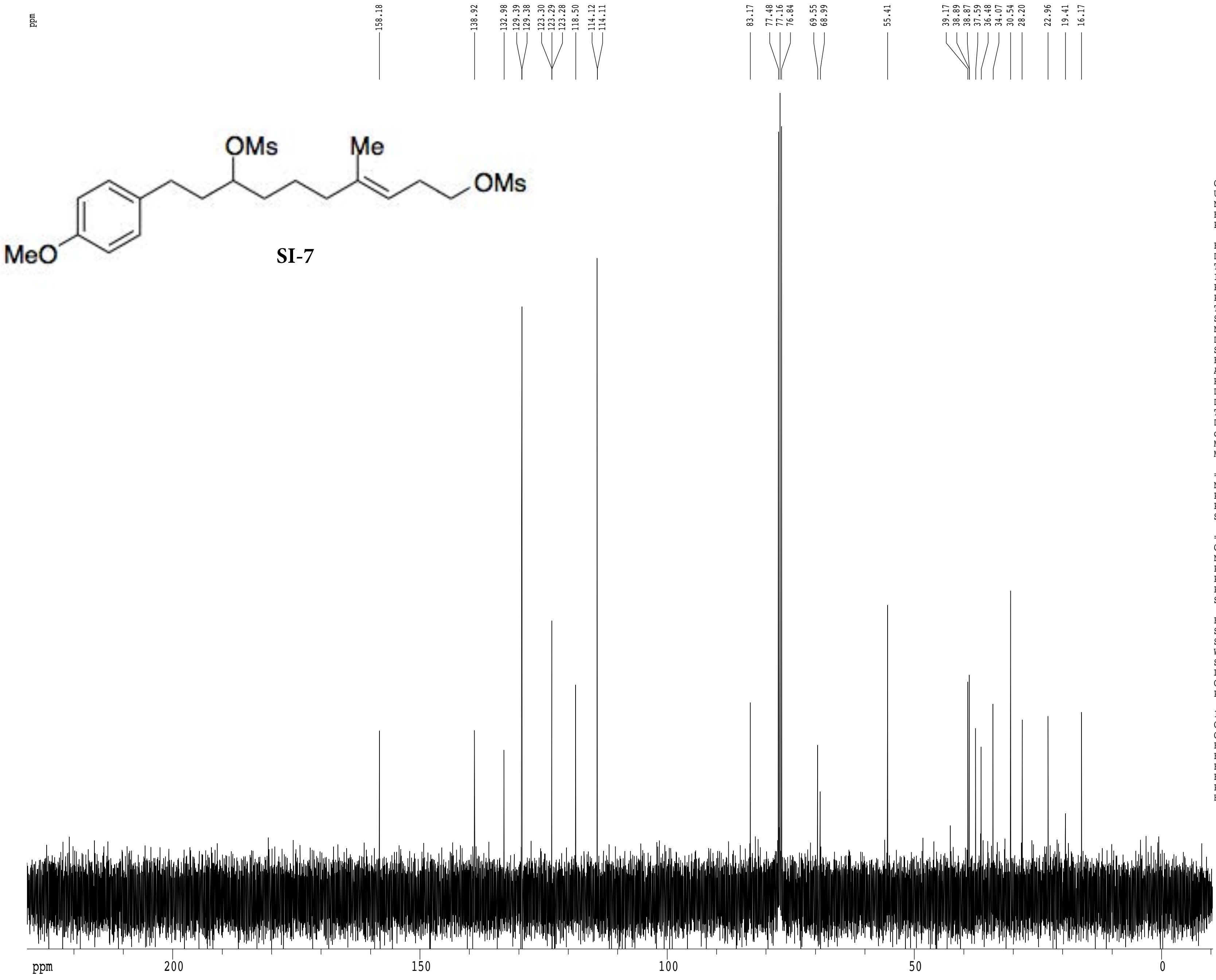

SI-7

```
Current Data Parameters
USER          khewitt1
NAME      KAH-V-203-chk
EXPNO                2
PROCNO               1

F2 - Acquisition Parameters
Date_         20211215
Time             17.01
INSTRUM         drx400
PROBHD   5 mm QNP H/F/P
PULPROG         zgdc30
TD               65536
SOLVENT          CDCl3
NS                 200
DS                   4
SWH          24154.590 Hz
FIDRES        0.368570 Hz
AQ           1.3566452 sec
RG               16384
DW              20.700 usec
DE               20.39 usec
TE               298.0 K
D1          0.10000000 sec
d11         0.03000000 sec
MCREST      0.00000000 sec
MCWRK       0.01500000 sec

======== CHANNEL f1 ========
NUC1               13C
P1                7.90 usec
PL1              -3.00 dB
SFO1       100.6237964 MHz

======== CHANNEL f2 ========
CPDPRG2        waltz16
NUC2                1H
PCPD2            90.00 usec
PL2              -0.90 dB
PL12             17.00 dB
SFO2       400.1328009 MHz

F2 - Processing parameters
SI               65536
SF         100.6127587 MHz
WDW                 no
SSB                  0
LB                0.00 Hz
GB                   0
PC                1.00

1D NMR plot parameters
CX               22.80 cm
CY               15.50 cm
F1P            229.496 ppm
F1            23090.21 Hz
F2P            -10.579 ppm
F2            -1064.37 Hz
PPMCM         10.52959 ppm/cm
HZCM        1059.41150 Hz/cm
```

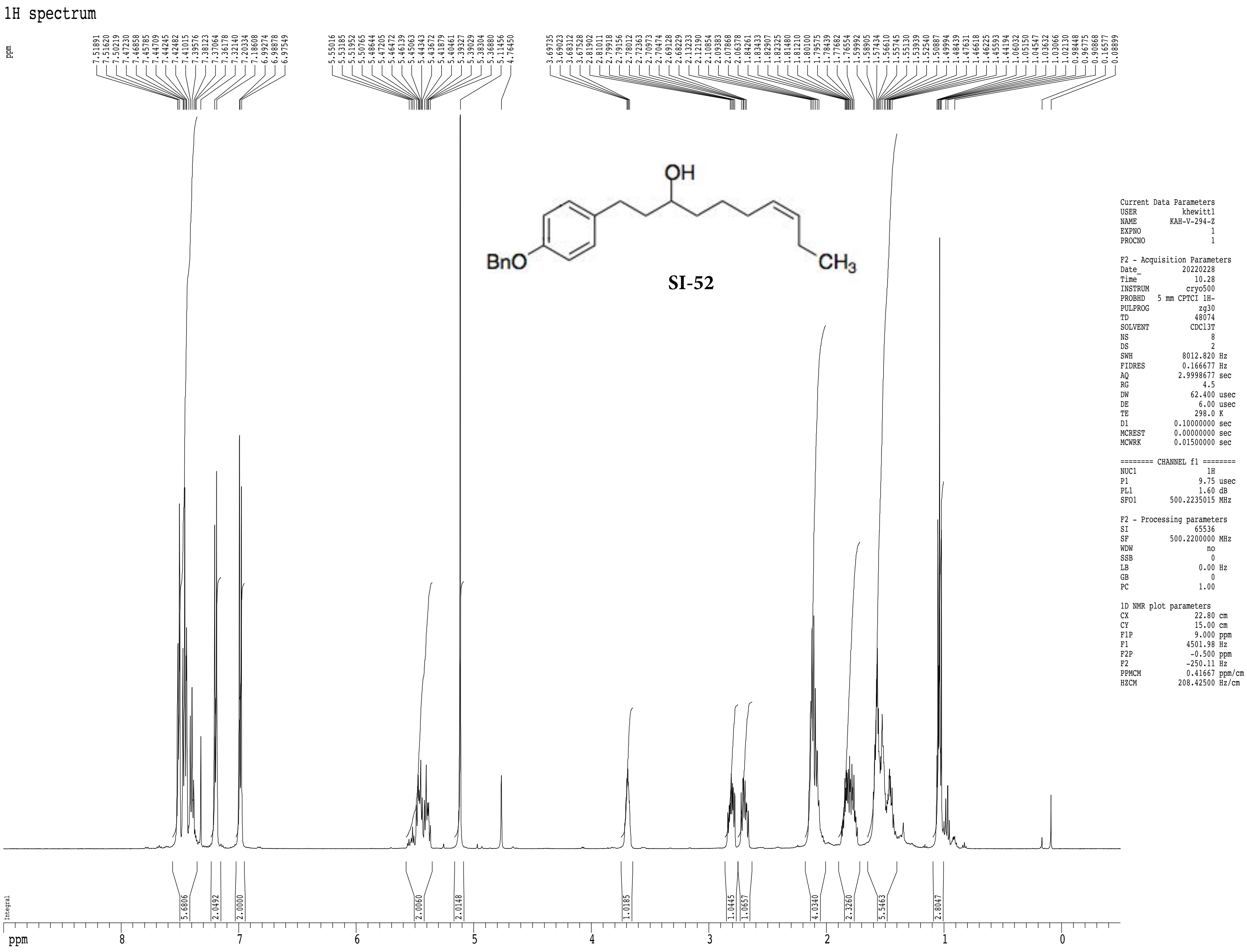
1H spectrum
OH
BnO
CH3
SI-52
Current Data Parameters
USER khewitt1
NAME KAH-V-294-Z
EXPNO 1
PROCNO 1
F2 - Acquisition Parameters
Date_ 20220228
Time 10.28
INSTRUM cryo500
PROBHD 5 mm CPTCI 1H-
PULPROG zg30
TD 48074
SOLVENT CDCl3T
NS 8
DS 2
SWH 8012.820 Hz
FIDRES 0.166677 Hz
AQ 2.9998677 sec
RG 4.5
DW 62.400 usec
DE 6.00 usec
TE 298.0 K
D1 0.10000000 sec
MCREST 0.00000000 sec
MCWRK 0.01500000 sec
======== CHANNEL f1 ========
NUC1 1H
P1 9.75 usec
PL1 1.60 dB
SFO1 500.2235015 MHz
F2 - Processing parameters
SI 65536
SF 500.2200000 MHz
WDW no
SSB 0
LB 0.00 Hz
GB 0
PC 1.00
1D NMR plot parameters
CX 22.80 cm
CY 15.00 cm
F1P 9.000 ppm
F1 4501.98 Hz
F2P -0.500 ppm
F2 -250.11 Hz
PPMCM 0.41667 ppm/cm
HZCM 208.42500 Hz/cm
5.6806
2.0492
2.0000
2.0060
2.0148
1.0185
1.0445
1.0657
4.0340
2.3260
5.5463
2.8047
ppm
8
7
6
5
4
3
2
1
0

Z-restored spin-echo 13C spectrum with 1H decoupling

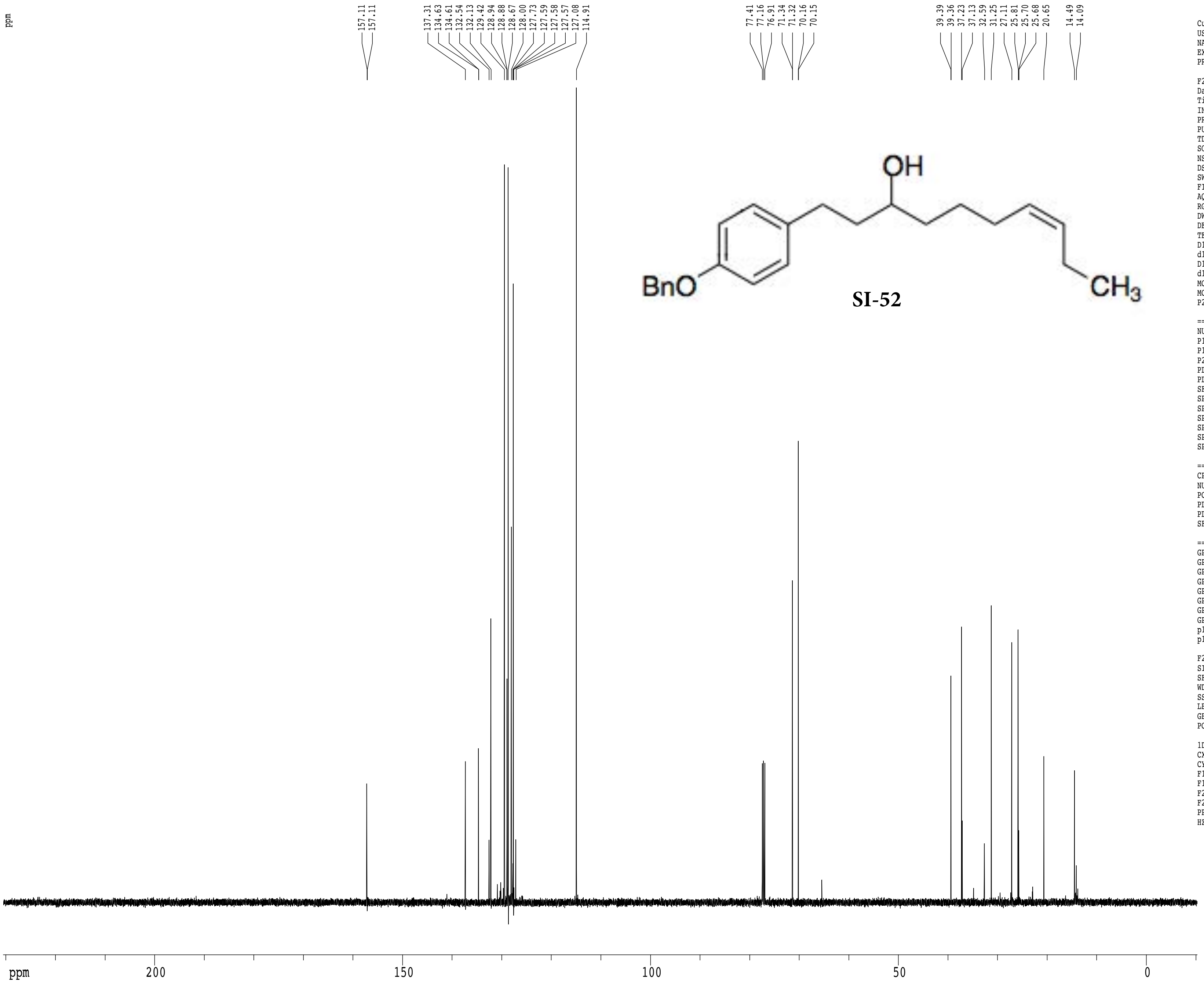

SI-52

Current Data Parameters
USER khewitt1
NAME KAH-V-294-Z
EXPNO 2
PROCNO 1

F2 - Acquisition Parameters
Date_ 20220228
Time 10.30
INSTRUM cryo500
PROBHD 5 mm CPTCI 1H-
PULPROG SpinEchopg30gp2.prd
TD 65536
SOLVENT CDCl3
NS 168
DS 16
SWH 30303.031 Hz
FIDRES 0.462388 Hz
AQ 1.0813940 sec
RG 7298.2
DW 16.500 usec
DE 6.00 usec
TE 298.0 K
D1 0.25000000 sec
d11 0.03000000 sec
D16 0.00020000 sec
d17 0.00019600 sec
MCREST 0.00000000 sec
MCWRK 0.01500000 sec
P2 37.70 usec

======== CHANNEL f1 ========
NUC1 13C
P1 18.85 usec
P12 2000.00 usec
P20 500.00 usec
PL0 120.00 dB
PL1 -1.00 dB
SFO1 125.7942548 MHz
SP2 1.55 dB
SP4 1.55 dB
SPNAM2 Crp60comp.4
SPNAM4 Crp60,0.5,20.1
SPOFF2 0.00 Hz
SPOFF4 0.00 Hz

======== CHANNEL f2 ========
CPDPRG2 waltz16
NUC2 1H
PCPD2 100.00 usec
PL2 1.60 dB
PL12 22.00 dB
SFO2 500.2225011 MHz

====== GRADIENT CHANNEL =====
GPNAM1 SINE.100
GPNAM2 SINE.100
GPX1 0.00 %
GPX2 0.00 %
GPY1 0.00 %
GPY2 0.00 %
GPZ1 30.00 %
GPZ2 50.00 %
p15 500.00 usec
p16 1000.00 usec

F2 - Processing parameters
SI 65536
SF 125.7804150 MHz
WDW no
SSB 0
LB 0.00 Hz
GB 0
PC 2.00

1D NMR plot parameters
CX 22.80 cm
CY 15.65 cm
F1P 230.637 ppm
F1 29009.68 Hz
F2P -10.287 ppm
F2 -1293.96 Hz
PPMCM 10.56688 ppm/cm
HZCM 1329.10706 Hz/cm

1H spectrum

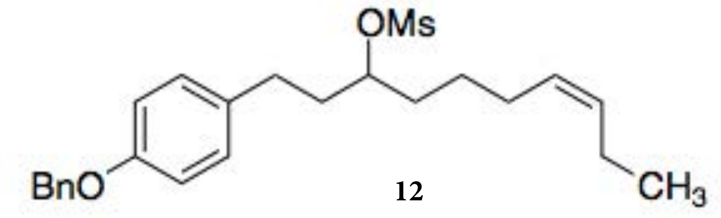

12

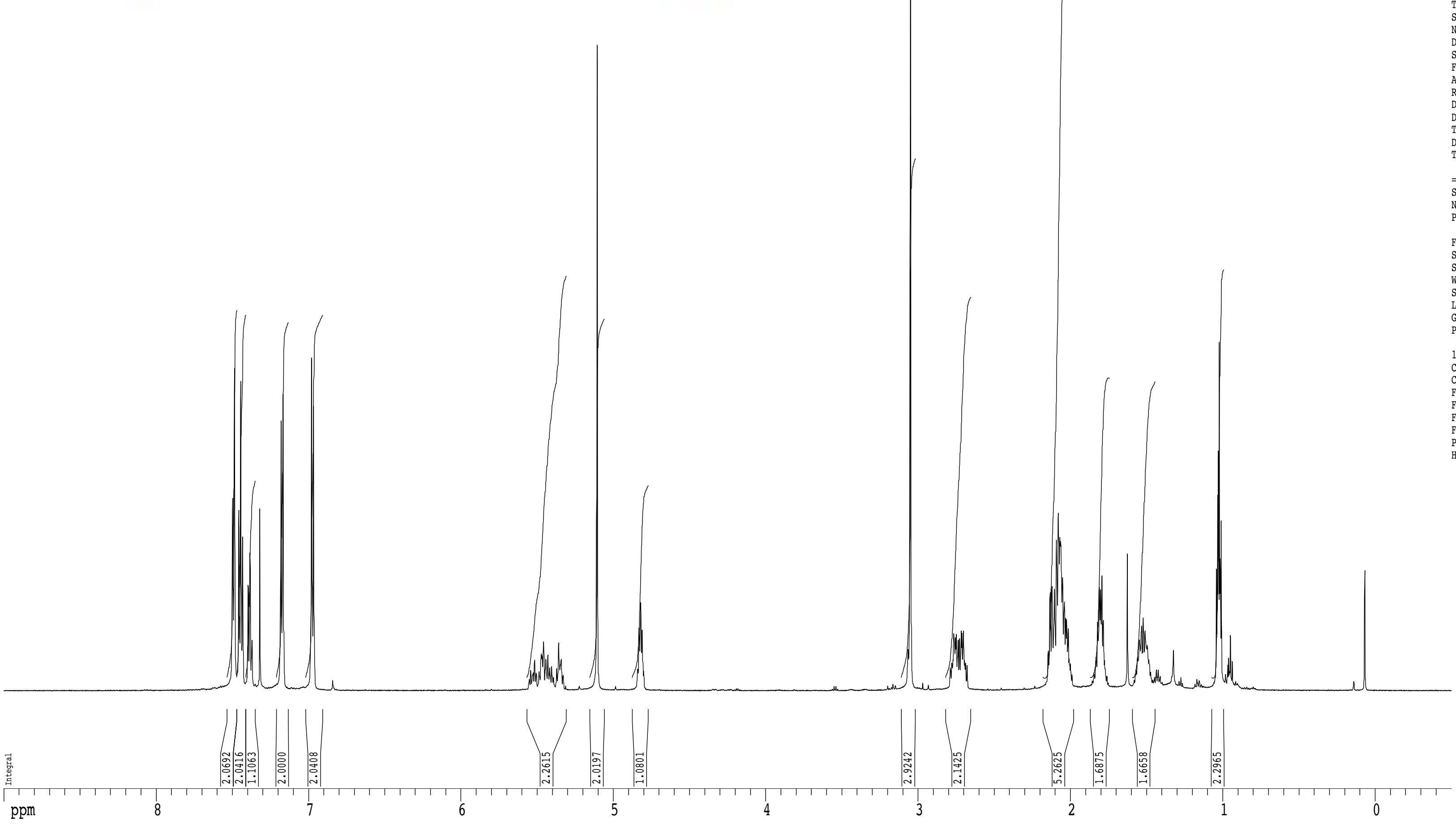


```
Current Data Parameters
USER            khewitt1
NAME     KAH-VI-059-Z-600
EXPNO                  1
PROCNO                 1

F2 - Acquisition Parameters
Date_           20220409
Time               12.38
INSTRUM            av600
PROBHD   5 mm CPBBO BB-
PULPROG             zg30
TD                 98074
SOLVENT            CDCl3T
NS                     8
DS                     2
SWH            9615.385 Hz
FIDRES         0.098042 Hz
AQ            5.0998979 sec
RG                    10
DW               52.000 usec
DE                14.12 usec
TE                298.0 K
D1           0.10000000 sec
TD0                    1

======== CHANNEL f1 ========
SFO1       600.1342009 MHz
NUC1                  1H
P1                 10.00 usec

F2 - Processing parameters
SI                 65536
SF           600.1300000 MHz
WDW                   no
SSB                    0
LB                  0.00 Hz
GB                     0
PC                  1.00

1D NMR plot parameters
CX                 22.80 cm
CY                 15.00 cm
F1P                9.000 ppm
F1               5401.17 Hz
F2P               -0.500 ppm
F2               -300.06 Hz
PPMCM            0.41667 ppm/cm
HZCM            250.05418 Hz/cm
```

13C spectrum

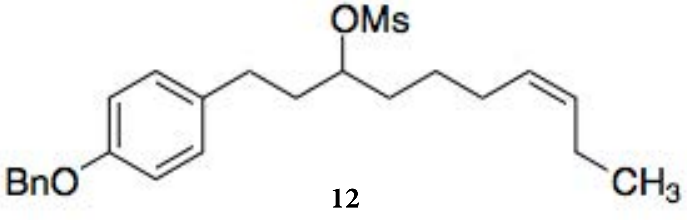

12

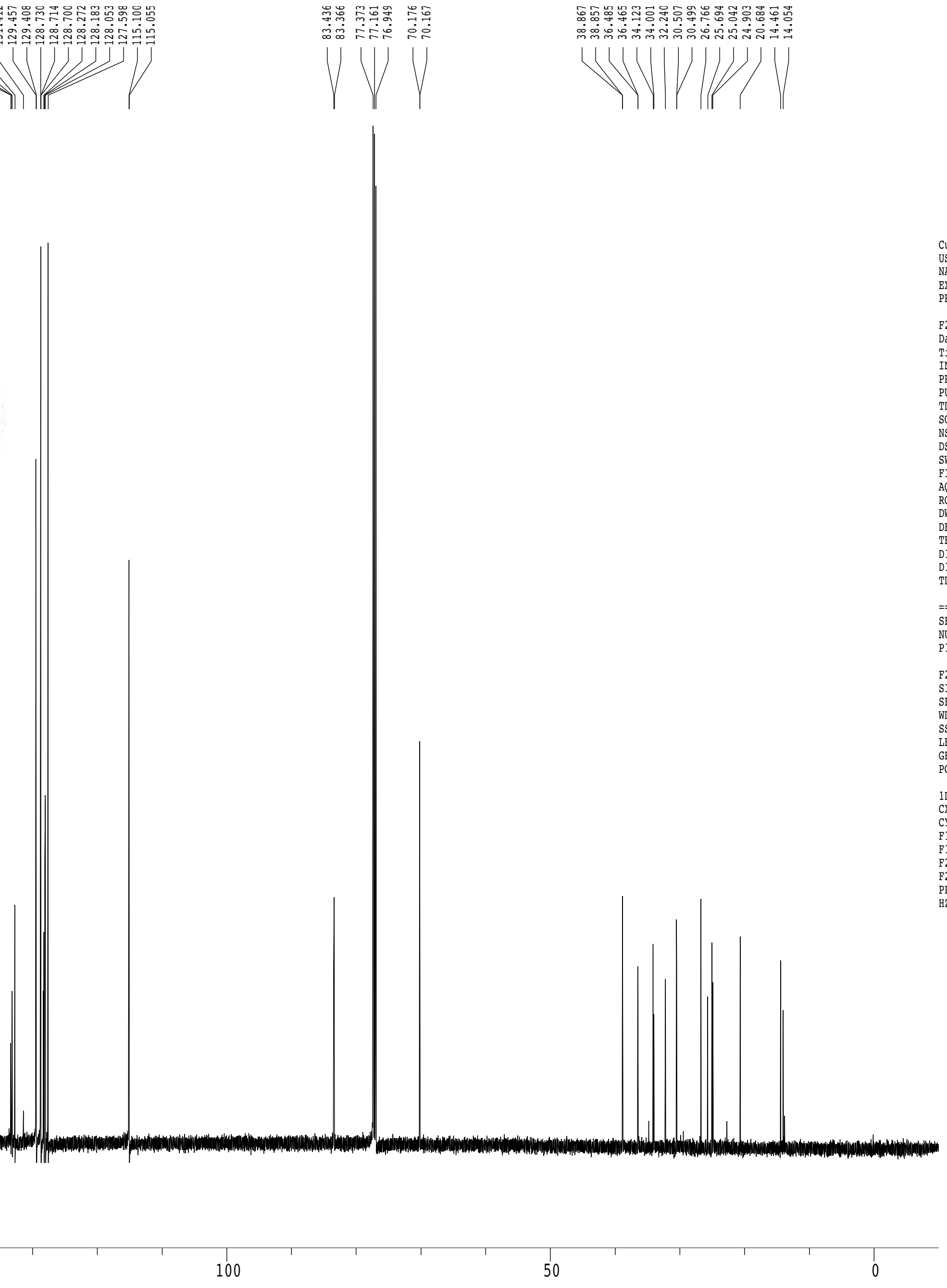

Current Data Parameters
USER khewitt1
NAME KAH-VI-059-Z-600
EXPNO 2
PROCNO 1

F2 - Acquisition Parameters
Date_ 20220409
Time 12.43
INSTRUM av600
PROBHD 5 mm CPBBO BB-
PULPROG zgdc30
TD 65536
SOLVENT CDCl3T
NS 86
DS 4
SWH 36231.883 Hz
FIDRES 0.552855 Hz
AQ 0.9044468 sec
RG 2050
DW 13.800 usec
DE 19.63 usec
TE 298.0 K
D1 0.40000001 sec
D11 0.03000000 sec
TD0 1

======== CHANNEL f1 ========
SFO1 150.9194080 MHz
NUC1 13C
P1 10.10 usec

F2 - Processing parameters
SI 65536
SF 150.9027975 MHz
WDW no
SSB 0
LB 0.00 Hz
GB 0
PC 1.00

1D NMR plot parameters
CX 22.80 cm
CY 15.00 cm
F1P 230.125 ppm
F1 34726.45 Hz
F2P -9.976 ppm
F2 -1505.44 Hz
PPMCM 10.53074 ppm/cm
HZCM 1589.11780 Hz/cm

1H spectrum

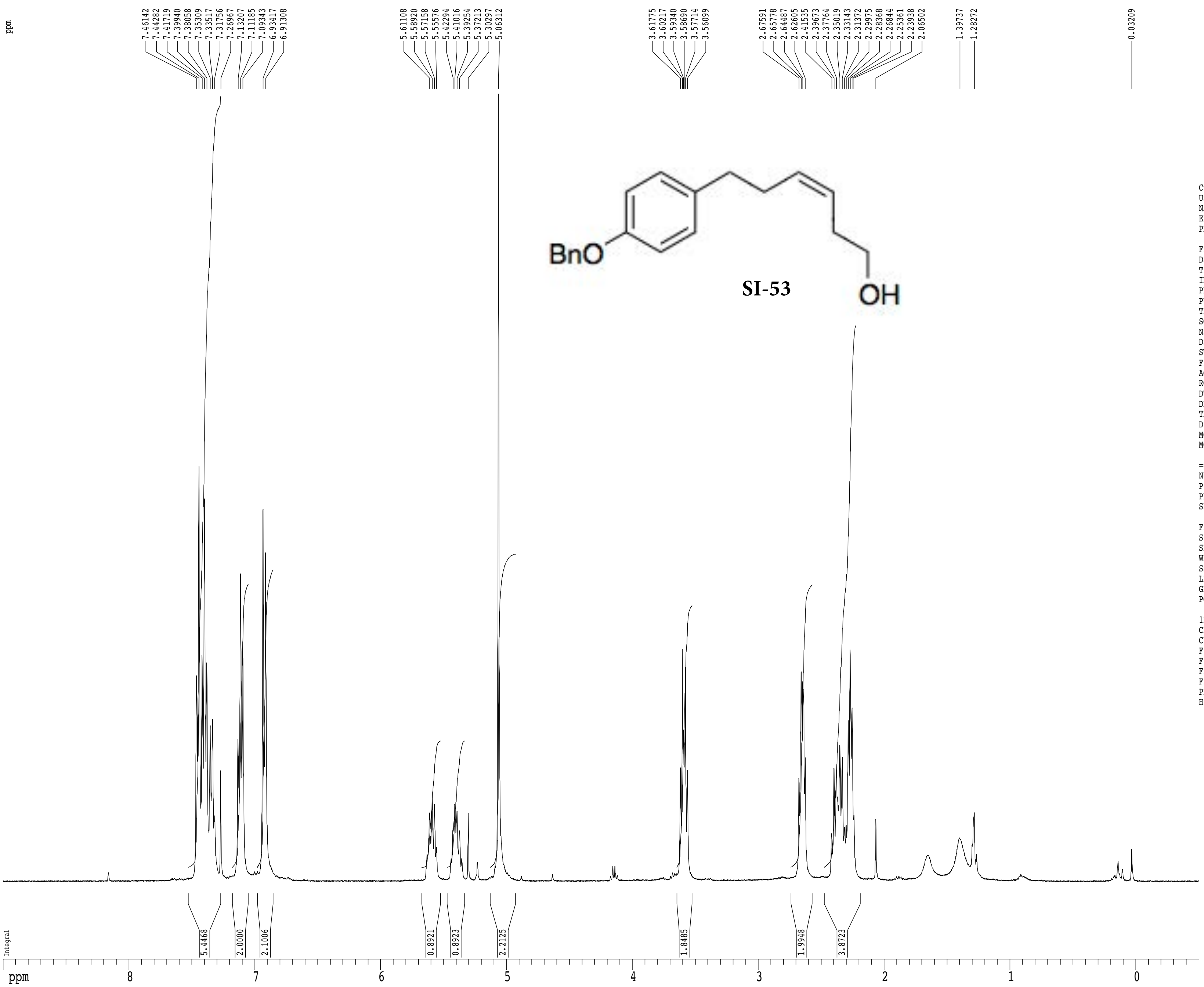

SI-53

```
Current Data Parameters
USER          khewitt1
NAME       KAH-VI-040-Z
EXPNO                 1
PROCNO                1

F2 - Acquisition Parameters
Date_          20220330
Time              16.33
INSTRUM          drx400
PROBHD   5 mm QNP H/F/P
PULPROG            zg30
TD                38460
SOLVENT          CDCl3T
NS                    8
DS                    2
SWH            6410.256 Hz
FIDRES         0.166673 Hz
AQ            2.9999299 sec
RG                  128
DW               78.000 usec
DE                 4.50 usec
TE                298.0 K
D1           0.10000000 sec
MCREST       0.00000000 sec
MCWRK        0.01500000 sec

======== CHANNEL f1 ========
NUC1                 1H
P1                12.00 usec
PL1               -0.90 dB
SFO1        400.1328009 MHz

F2 - Processing parameters
SI                65536
SF          400.1300175 MHz
WDW                  no
SSB                   0
LB                 0.00 Hz
GB                    0
PC                 2.00

1D NMR plot parameters
CX                22.80 cm
CY                15.00 cm
F1P               9.000 ppm
F1              3601.17 Hz
F2P              -0.500 ppm
F2              -200.06 Hz
PPMCM           0.41667 ppm/cm
HZCM          166.72084 Hz/cm
```

13C spectrum with 1H decoupling

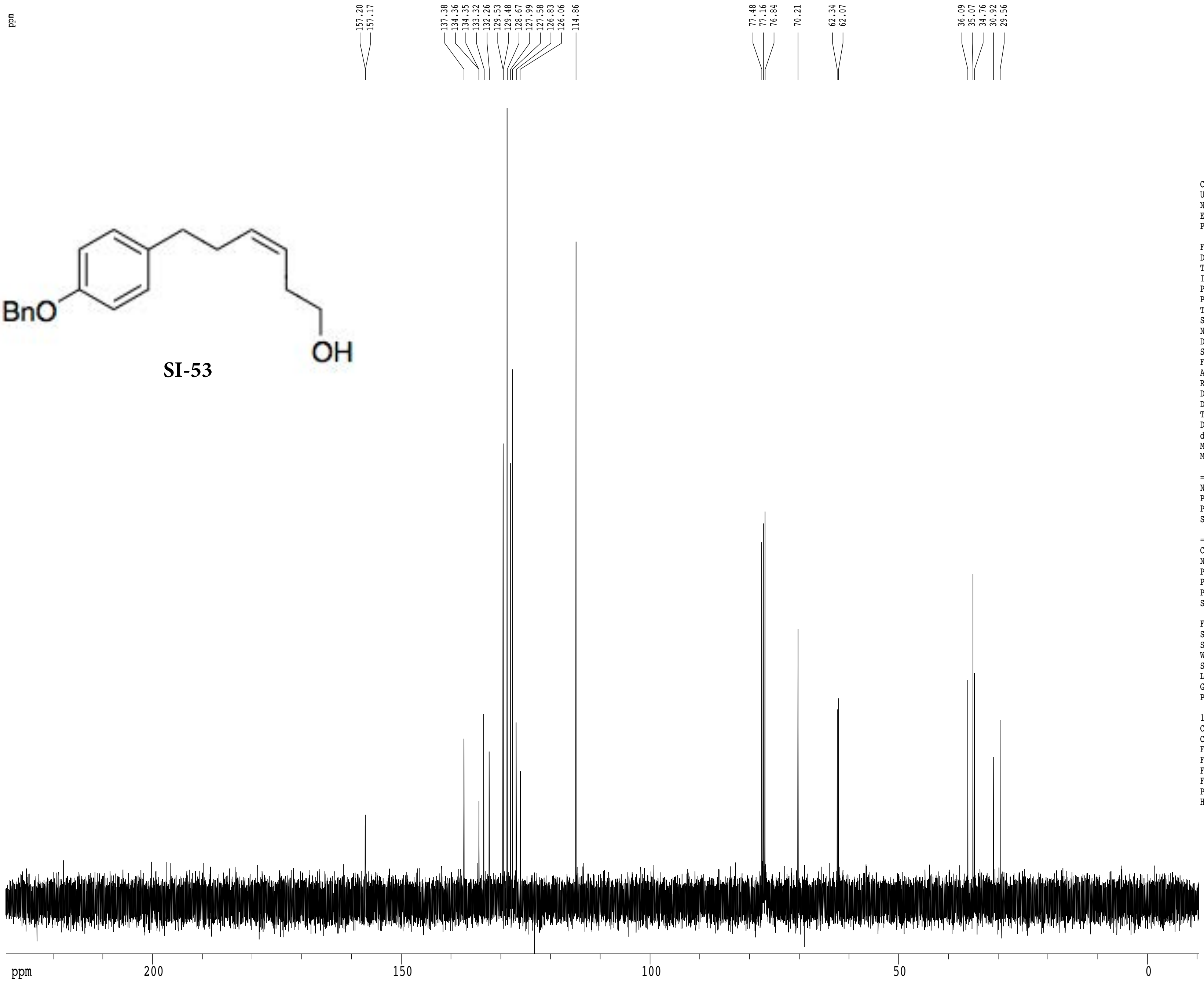

SI-53

```
Current Data Parameters
USER            khewitt1
NAME        KAH-VI-040-Z
EXPNO                  2
PROCNO                 1

F2 - Acquisition Parameters
Date_           20220330
Time               16.35
INSTRUM           drx400
PROBHD   5 mm QNP H/F/P
PULPROG           zgdc30
TD                 65536
SOLVENT            CDCl3
NS                   175
DS                     4
SWH            24154.590 Hz
FIDRES          0.368570 Hz
AQ             1.3566452 sec
RG                9195.2
DW                20.700 usec
DE                 20.39 usec
TE                 298.0 K
D1            0.10000000 sec
d11           0.03000000 sec
MCREST        0.00000000 sec
MCWRK         0.01500000 sec

======== CHANNEL f1 ========
NUC1                 13C
P1                  7.90 usec
PL1                -3.00 dB
SFO1         100.6237964 MHz

======== CHANNEL f2 ========
CPDPRG2           waltz16
NUC2                   1H
PCPD2               90.00 usec
PL2                 -0.90 dB
PL12                17.00 dB
SFO2          400.1328009 MHz

F2 - Processing parameters
SI                  65536
SF            100.6127610 MHz
WDW                    no
SSB                     0
LB                   0.00 Hz
GB                      0
PC                   1.00

1D NMR plot parameters
CX                  22.80 cm
CY                  15.50 cm
F1P               229.496 ppm
F1               23090.21 Hz
F2P               -10.579 ppm
F2               -1064.37 Hz
PPMCM            10.52959 ppm/cm
HZCM           1059.41150 Hz/cm
```

1H spectrum

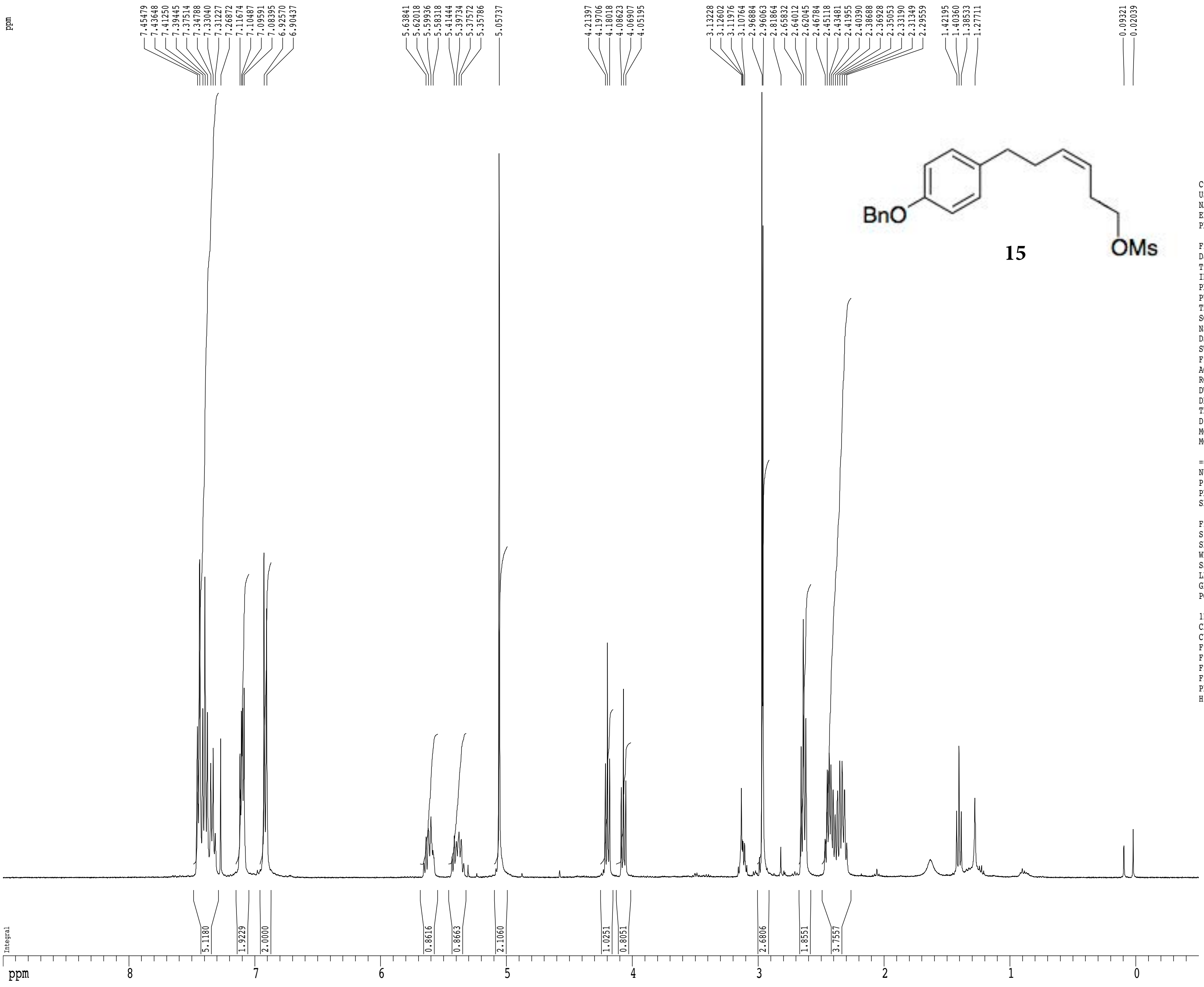

15

```
Current Data Parameters
USER            khewitt1
NAME        KAH-VI-041-1
EXPNO                  1
PROCNO                 1

F2 - Acquisition Parameters
Date_           20220331
Time               10.31
INSTRUM           drx400
PROBHD   5 mm QNP H/F/P
PULPROG             zg30
TD                 38460
SOLVENT           CDCl3T
NS                     8
DS                     2
SWH             6410.256 Hz
FIDRES          0.166673 Hz
AQ             2.9999299 sec
RG                 161.3
DW                78.000 usec
DE                  4.50 usec
TE                 298.0 K
D1            0.10000000 sec
MCREST        0.00000000 sec
MCWRK         0.01500000 sec

======== CHANNEL f1 ========
NUC1                  1H
P1                 12.00 usec
PL1                -0.90 dB
SFO1         400.1328009 MHz

F2 - Processing parameters
SI                 65536
SF           400.1300175 MHz
WDW                   no
SSB                    0
LB                  0.00 Hz
GB                     0
PC                  2.00

1D NMR plot parameters
CX                 22.80 cm
CY                 15.00 cm
F1P                9.000 ppm
F1               3601.17 Hz
F2P               -0.500 ppm
F2               -200.06 Hz
PPMCM            0.41667 ppm/cm
HZCM           166.72084 Hz/cm
```

13C spectrum with 1H decoupling

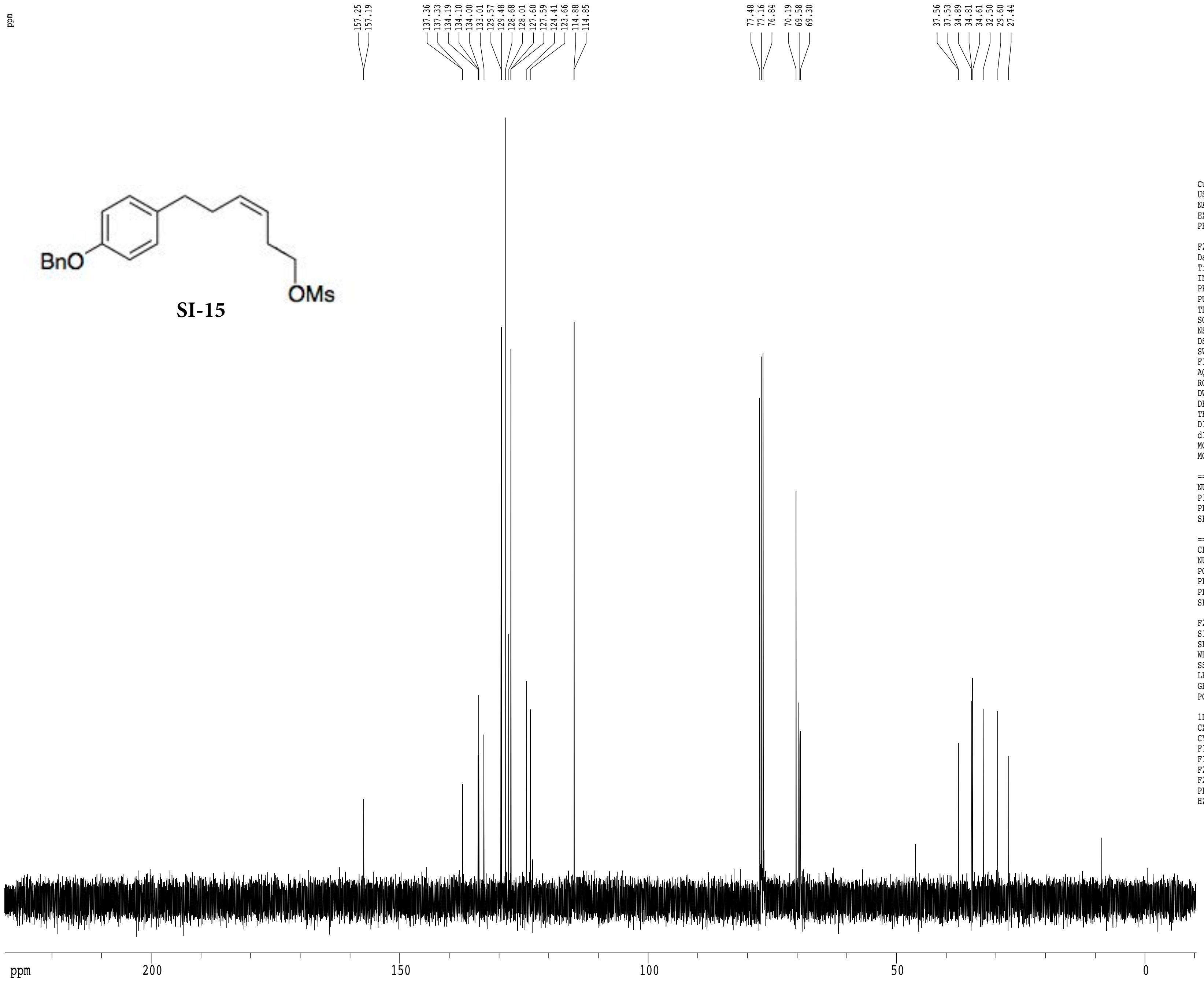

Current Data Parameters
USER khewitt1
NAME KAH-VI-041-1
EXPNO 2
PROCNO 1

F2 - Acquisition Parameters
Date_ 20220331
Time 10.33
INSTRUM drx400
PROBHD 5 mm QNP H/F/P
PULPROG zgdc30
TD 65536
SOLVENT CDCl3
NS 288
DS 4
SWH 24154.590 Hz
FIDRES 0.368570 Hz
AQ 1.3566452 sec
RG 9195.2
DW 20.700 usec
DE 20.39 usec
TE 298.0 K
D1 0.10000000 sec
d11 0.03000000 sec
MCREST 0.00000000 sec
MCWRK 0.01500000 sec

======== CHANNEL f1 ========
NUC1 13C
P1 7.90 usec
PL1 -3.00 dB
SFO1 100.6237964 MHz

======== CHANNEL f2 ========
CPDPRG2 waltz16
NUC2 1H
PCPD2 90.00 usec
PL2 -0.90 dB
PL12 17.00 dB
SFO2 400.1328009 MHz

F2 - Processing parameters
SI 65536
SF 100.6127606 MHz
WDW no
SSB 0
LB 0.00 Hz
GB 0
PC 1.00

1D NMR plot parameters
CX 22.80 cm
CY 15.50 cm
F1P 229.496 ppm
F1 23090.21 Hz
F2P -10.579 ppm
F2 -1064.37 Hz
PPMCM 10.52959 ppm/cm
HZCM 1059.41150 Hz/cm
